# Loss of orf3b in the circulating SARS-CoV-2 strains

**DOI:** 10.1080/22221751.2020.1852892

**Published:** 2020-12-24

**Authors:** Joy-Yan Lam, Chun-Kit Yuen, Jonathan Daniel Ip, Wan-Man Wong, Kelvin Kai-Wang To, Kwok-Yung Yuen, Kin-Hang Kok

**Affiliations:** a Department of Microbiology, Li Ka Shing Faculty of Medicine, The University of Hong Kong, Pokfulam, Hong Kong Special Administrative Region, People’s Republic of China.; b State Key Laboratory of Emerging Infectious Diseases, The University of Hong Kong, Pokfulam, Hong Kong Special Administrative Region, People’s Republic of China

**Keywords:** SARS-CoV-2, orf3b, orf3a, Q57H, D614G

## Abstract

The newly emerged betacoronavirus, SARS-CoV-2, causes the COVID-19 pandemic since December 2019 with more than 35 million laboratory confirmed human infections and over one million deaths within nine months. The genome of SARS-CoV-2 continues to evolve during the global transmission with the notable emergence of the spike D614G substitution that enhances infectivity. Some of these viral adaptations may alter not only the infectivity but also viral pathogenesis. Continuous phylogenomic analysis of circulating viral strains and functional investigation of new non-synonymous substitutions may help to understand the evolution of virus, its virulence and transmissibility. Here we describe a loss of an accessory protein orf3b (57 amino acids) in current circulating SARS-CoV-2 strains, contributing around 24% of more than 100,000 complete viral genomes analysed. The loss of 3b is caused by the presence of an early stop codon which is created by an orf3a Q57H substitution. There is an increasing trend in the loss of orf3b which has reached 32% in May 2020. Geographically, loss of 3b is more prevalent in certain countries including Colombia (46%), USA (48%), South Korea (51%), France (66%), Saudi Arabia (72%), Finland (76%) and Egypt (77%). Interestingly, the loss of 3b coincides with the emergence of spike D614G substitution. In addition, we found that truncated orf3b has lost the interferon antagonism compared to the full-length orf3b, suggesting a loss of function by the newly adapted virus. Further investigation of orf3b deletion and spike D614G substitution on virulence and infectivity respectively will provide important insights into SARS-CoV-2 evolution.

## Introduction

In late 2019, the newly emerged severe acute respiratory syndrome-coronavirus-2 (SARS-CoV-2) causes a global pandemic of COVID-19 in an unprecedented scale. According to the statistics from World Health Organization (WHO) released on 19 September 2020, there are over 35 million total confirmed cases and over one million deaths globally (https://covid19.who.int/). Several determinants contribute to this large-scale pandemic including the presence of large number of asymptomatic and mildly symptomatic patients, effective human-to-human transmission and immunomodulatory characteristic of SARS-CoV-2 [1–3]. Current efforts have been made for developing antivirals, immunomodulatory agents for alleviating disease severity, safe and effective vaccines, as well as rapid and accurate diagnostic tools by the unprecedented close collaborations between governments, pharmaceutical companies and various institutions. To tackle this novel emerging viral infectious disease, understanding the viral genome and evolution, viral protein function and structure, viral pathogenesis and transmissibility, is critical for the successful development of accurate diagnostic assays, effective therapeutics and vaccination.

Continuous phylogenetic analysis on the bank of open-source whole genome sequences of SARS-CoV-2 not only provides valuable information on its genome structures, but also allows in-depth analysis on the evolution of the virus, which serves as the basis of therapeutics developments. SARS-CoV-2 belongs to the subgenus of Sarbecovirus under the genus of betacoronavirus, and is closely related to SARS-CoV and SARS-related-CoVs [4]. Its genome encodes 27 proteins, in which the structural proteins, non-structural proteins and the accessory proteins are highly similar to its closely related betacoronaviruses, except the spike, orf3b, and orf8. Naturally, sporadic mutations arise frequently during the SARS-CoV-2 transmission which are considered useful genetic markers to visualize the transmission path, understand the observed changes in pathogenesis and give clues for the control of this pandemic. For instance, spike D614G substitution that arose in January has enhanced the virus transmissibility and infectivity [5, 6]. The spike G614 genotype soon became the dominant species. Regarding the accessory proteins, both orf6 and orf8 deletions have been reported [7, 8]. In particular, the 27-nucleotide deletion in orf6 will possibly alter protein folding, and the 382-nucleotide deletions in orf8 will very likely cause the loss of protein expression. Albeit seemingly minor, these deletion mutations will likely cause a change in the virus phenotype. For example, milder infections were reported in orf8-deleted virus infected patients [9]. Such alterations in the virus genome at genes unique to SARS-CoV-2 may also affect the targets for serological tracking and also the development of accurate diagnostic assays.

Previously, orf3b has been first proposed as a SARS-CoV-2 protein by our genome analysis published during early outbreak of COVID-19 [4]. It is translated from the frame 3 of orf3a gene (25524-25697 nt; 174 bp) and encodes a protein with 57 amino acids. A proteomic study identified STOML2 as its binding partner [10] and it may play a role in mitochondrial homeostasis [11]. More importantly, a recent serological study demonstrated a strong antibody response against orf3b protein in a cohort of COVID-19 patients and the anti-orf3b antibodies have been proposed for the use as the serological markers of early and late infection [12]. It is noted that a recent paper studying a putative orf3 protein (a 22 amino acids peptide predicted from frame 3 of orf3a (25814-25882; 69 bp)) and named it as orf3b [13]. Its amino acid sequence is totally different from the orf3b described in this and other studies [4, 10, 12].

Here, we report the increasing dominance of orf3b-deleted virus strains amongst the over 100,000 publicly available SARS-CoV-2 genome sequences. We showed that the Q57H amino acid substitution in SARS-CoV-2 orf3a gives rise to a premature stop codon in the reading frame for orf3b, and such genotype contributes to 23.82% of analysed sequences. There was an increasing number of viruses with loss of orf3b that has reached over 31.86% in May 2020, suggesting an increasing prevalence of its circulation. Moreover, several locations have increased prevalence of viruses with loss of orf3b, including Colombia (46%), USA (48%), South Korea (51%), France (66%), Saudi Arabia (72%), Finland (76%) and Egypt (77%), suggesting that significant host serological differences and immune pressure may exist which leads to the virus escape from such immune pressure of human population by the loss of orf3b protein. Furthermore, we performed structural prediction on both full-length orf3b and truncated-orf3b and determine that the truncated mutant will very likely lead to the loss of orf3b protein. Our previous report identified four most potent SARS-CoV-2 interferon antagonists including NSP13, NSP14, NSP15 and orf6 [14]. Orf3b has been shown to inhibit interferon production, but to a lesser extent when compared to orf6. Therefore, we have examined the interferon antagonism of wild-type and truncated-orf3b and confirmed that truncated orf3b loses its activity of interferon antagonism. Further in-vivo and in-vitro study of orf3b may reveal additional functions on viral pathogenesis and modulation of host cell signalling pathways, hinting a functional impact on this virus on its host adaptation or evolution. Taken together, our study indicates that there is increasing circulation of SARS-CoV-2 with the loss of orf3b in the ongoing pandemic, which coincides with the emergence of spike D614G virus strains. More attention on the loss of orf3b is required for the future surveillance, design of serological tests, development of vaccines and diagnostic tools for COVID-19.

## Materials and methods

### Cell culture and transfection

293FT cells (Thermo Fisher Scientific) was cultured in Dulbecco's Modified Eagle Medium (DMEM) supplemented with 10% Fetal bovine serum (FBS). GeneJuice (Novagen) was used for transfection according to manufacturer’s instruction.

### Plasmids

Gene fragments encoding SARS-CoV-2 orf3a and full-length orf3b were obtained in previous study [14]. The fragment containing orf3a with H57 genotype was generated by PCR, while the fragment for truncated orf3b with 13 amino acids were synthesized as short oligos (BGI). All gene fragments were cloned into pCAGEN expression vector with c-terminal FLAG-tag and confirmed by Sanger sequencing.

### Dual luciferase assay

293FT cells were transfected in triplicate with IFNβ-luc firefly luciferase reporter and pRL-TK Renilla luciferase reporter (Promega), RIG-IN [15], together with the indicated expression plasmids. Cells were lysed at 24 h post-transfection using 1X passive lysis buffer (Promega). Firefly and Renilla luciferase signals were quantified using Dual Luciferase Reporter Assay System (Promega), represented as relative luciferase activity by dividing firefly luciferase signal with Renilla luciferase signal. Statistical analysis was performed by student’s t test.

### RNA isolation and RT-qPCR

Similar to luciferase assay, 293FT cells were transfected with RIG-IN and indicated expression plasmids. Cells were lysed at 24 h post-transfection directly with RNAiso (Takara) and cellular total RNA was extracted following manufacturer’s instruction. Reverse transcription was performed with PrimeScript RT reagent kit with gDNA eraser (Takara) using the kit primer mix. IFNβ mRNA transcript expression was quantified by quantitative PCR (qPCR) using SYBR Premix Ex Taq (Takara). GAPDH was used as the housekeeping gene to determine relative transcript expression. Statistical analysis was performed by student’s t test.

### Phylogenetic analysis and sequence analysis

SARS-CoV-2 sequences and sequence author data were downloaded from GISAID depository and NextStrain.org [16]. Acknowledgment table was listed in Supplementary Table S1. Multiple alignment of 106 representative SARS-CoV-2 sequences was performed using MAFFT online server (https://mafft.cbrc.jp/alignment/server/) using default parameters, followed by phylogenetic tree analysis by MEGA X programme [17]. Phylogenetic tree was generated with Maximum Likelihood with bootstrapping value equals to 1000 and evolutionary analysis was conducted in MEGA X programme by using Tamura-Nei model. The tree was drawn to scale, with branch lengths representing evolutionary distances. For sequence analysis, an in-house python script was used to process 163,333 whole genome sequences. Low coverage sequences, sequences with gaps or ambiguous bases, sequences with no collection dates were filtered out, resulting in 150,198 sequences for formal analysis. Changes in respective nucleotide positions of interest were recorded and exported.

### Protein structure prediction

Orf3b structural prediction was performed using comparative modelling with RosettaCM on Robetta protein structure prediction server [18]. The structure was modelled by the analysis of alignment clusters using HHSEARCH, SPARKS, and Raptor. The predicted structure was visualized and presented using PyMOL (DeLano Scientific LLC).

#### Luciferase Immunoprecipitation System (LIPS) assay

LIPS assay (Figure S1) was performed according to a protocol previously described by Burbelo et al. [19] with modification. *Renilla* antigen was prepared by transfecting 293FT cells with *Renilla* luciferase-orf3b expression plasmid, followed by lysis at 48 h post-transfection. Heat-inactivated patient sera were 1:100 diluted and incubated with 1×10^7^ light units of *Renilla* antigen on a rotary shaker. 1.5 µL of Protein A/G resin (Thermo Fisher Scientific, USA) diluted in PBS was then added to each sample and incubated to capture the antibody–antigen complex. Following three washes, the beads were transferred to an opaque 96-well plate. Renilla substrate (Promega, USA) was then added and luciferase signal was measured using the Wallac Victor3 Multilabel Counter (PerkinElmer, USA).

## Results

### Q57H substitution in SARS-CoV-2 orf3a contributes to orf3b truncation

**Figure 2.**
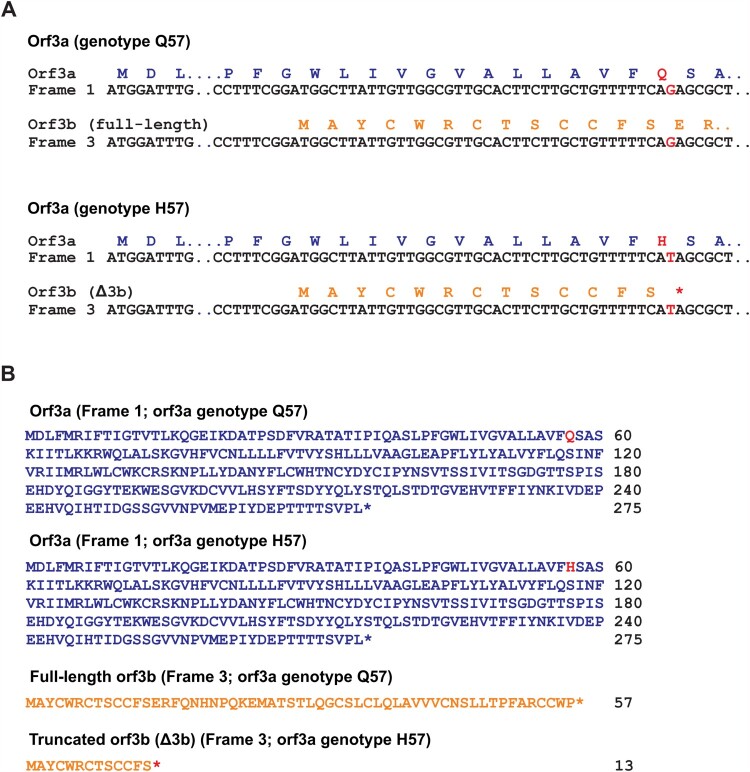
SARS-CoV-2 orf3a Q57H substitution creates early stop codon of orf3b. (A) Highlight of the Q57H substitution in orf3a and the introduction of premature stop codon in orf3b. The reading frame for orf3a is denoted as frame 1 and that of orf3b as frame 3. Amino acid changes at position 57 in orf3a and the respective site in orf3b were highlighted in red. (B) Complete amino acid sequence of orf3a Q57, orf3a H57, full-length orf3b and Δ3b were shown. Likewise, orf3a position 57 was highlighted in red. Respective peptide length was labelled as shown.

The SARS-CoV-2 virus has continued to evolve since its first appearance in late 2019. For instance, viruses with D614G mutation in Spike protein have acquired enhanced transmissibility and infectivity and become the dominant circulating strain [6, 20]. In order to better understand the evolution of SARS-CoV-2, we performed phylogenetic analysis of publicly available SARS-CoV-2 sequences. Consistent with published findings [10, 21], we identified a distinct clade that represents the G25563T nucleotide mutation, which results in Q57H amino acid substitution in orf3a (red box in Figure 1A, and red lines in Figure 1B). In our analysis, spike D614G substitution was also included as a reference since it signifies the evolutionary phases of SARS-CoV-2 (blue box in Figure 1A, and blue lines in Figure 1C). All H57 strains clustered within the clades with spike G614, indicating that this mutation has co-evolved with the D614G mutation (blue box in Figure 1A and coloured as blue in Figure 1C). While some spike G614 strains contain the orf3a Q57H mutation, all orf3a H57 strains contain the spike D614G mutation. Orf3a shares overlapping genome position at amino acid 44–102 with orf3b, which is a 57 amino acid open reading frame transcribed in frame 3 relative to orf3a (frame 1) (Figure 2A). The G25563T mutation gives rise to orf3a Q57H substitution and also introduces an early stop codon to orf3b after amino acid 13 (Δ3b), causing a large truncation of 44 amino acids to the protein (Figure 2B). This implies that there are currently two co-circulating orf3b genotypes, one contains full-length orf3b, and the other has orf3b truncated. Also, the Δ3b viruses contain both the orf3a Q57H substitution and D614G mutation. Figure 1.Recent emergence of SARS-CoV-2 orf3a Q57H and spike D614G genotype. (A) Multiple alignment of 106 representative SARS-CoV-2 sequences extracted from GISAID were performed using MAFFT online server (https://mafft.cbrc.jp/alignment/server/). Phylogenetic tree was constructed by Maximum Likelihood using MEGA X (17) programme with 1000 bootstrap value. The tree was rooted with hCoV-19/Wuhan/IPBCAMS-WH-01/2019 (EPI ISL 402123), which is the earliest available sequence on GSIAID. Specific genotypes were highlighted in colours: orange for orf3a Q57, red for orf3a H57, green for spike D614, blue for spike G614. The distinct clade of orf3a H57 was also highlighted inside the red box, and the population for spike G614 in blue box. (B-C) Radial display of the phylogenetic analysis. Respective clade for orf3a and spike was labelled the same as the previous display.

**Figure:**
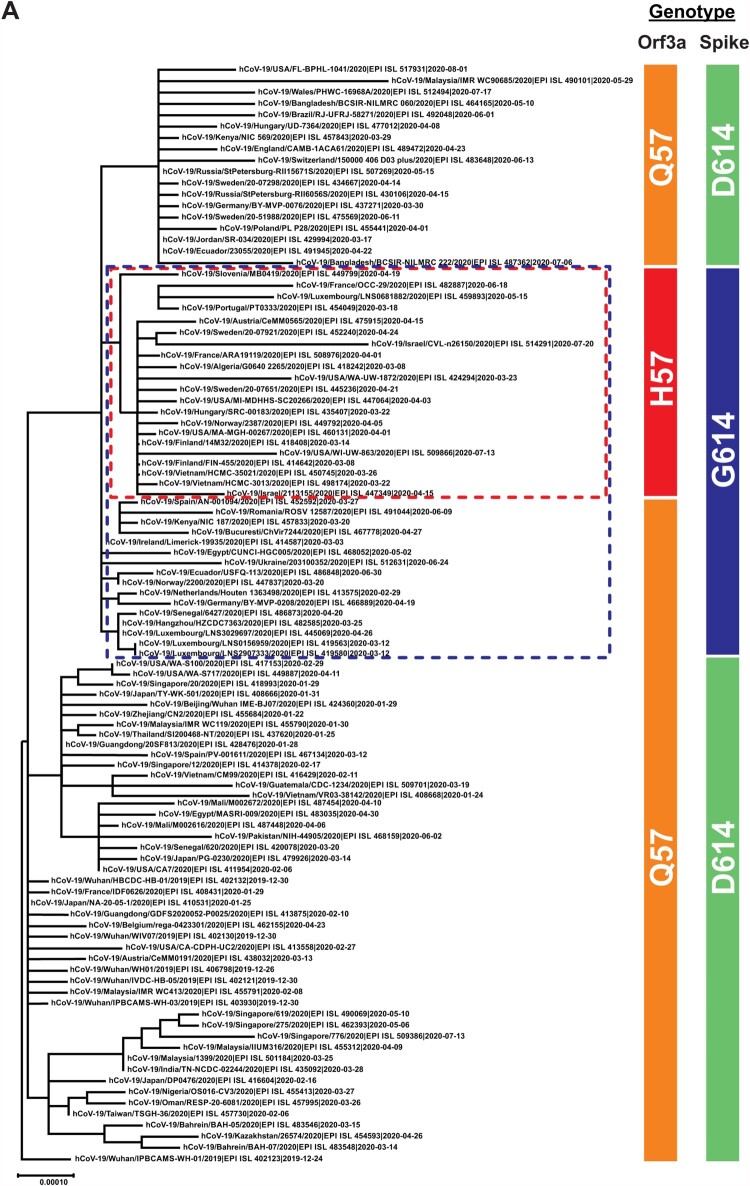

**Figure:**
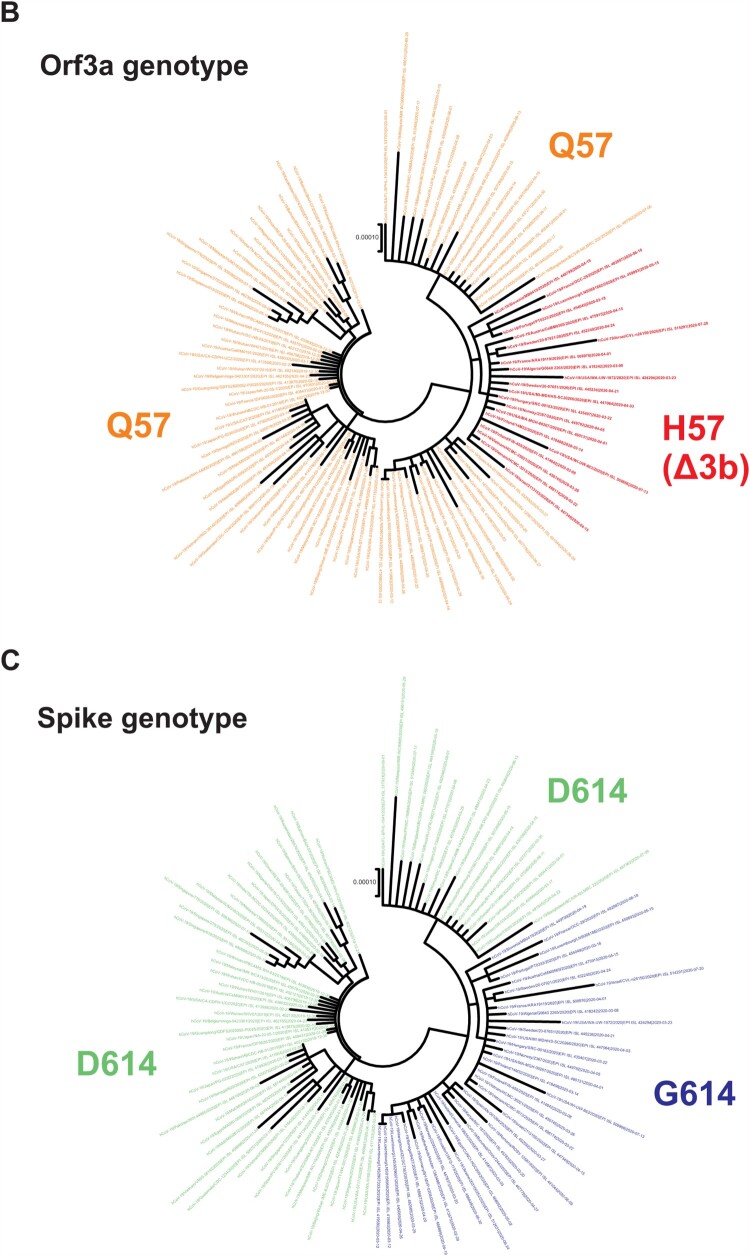

### Recent sequence data highlights the emergence and circulation of Δ3b viruses

**Figure 3.**
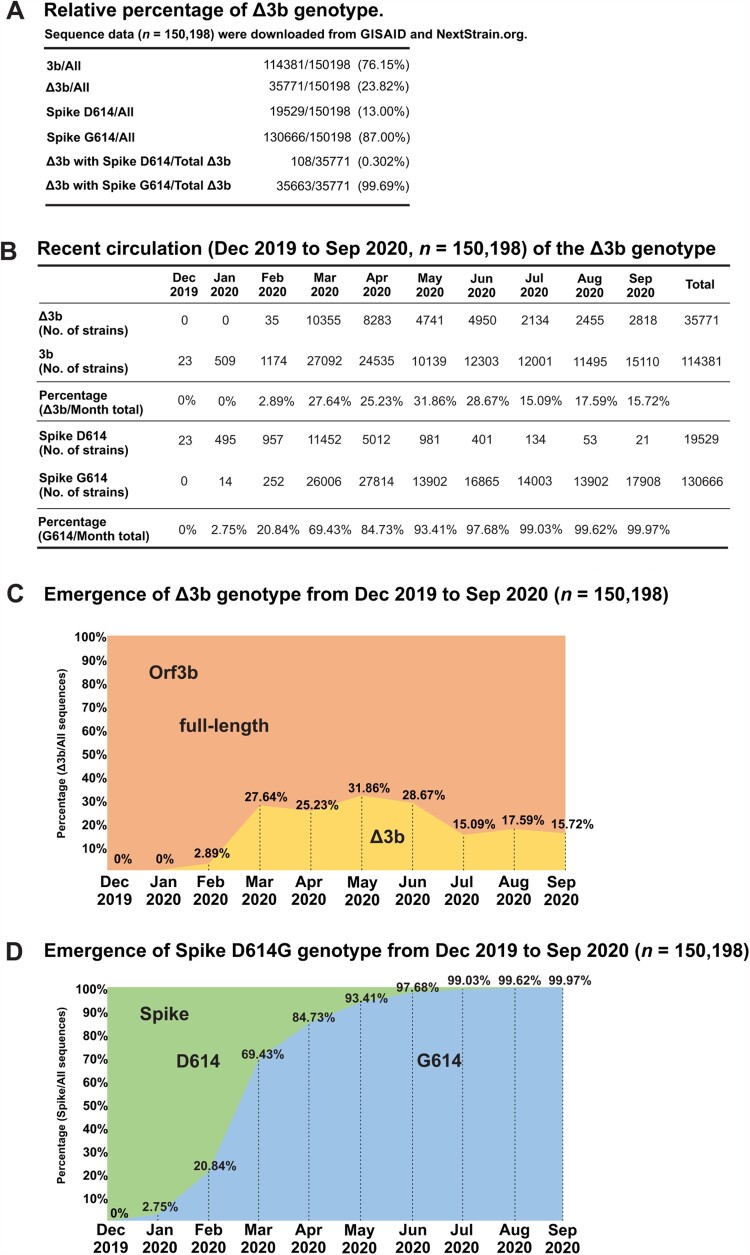
Circulation of SARS-CoV-2 strains with Δ3b. (A) Summary of total sequences with wild type orf3b, Δ3b, D614 and G614, and statistics for the proportions of Δ3b with spike D614 or G614. (B) Monthly summary of sequences with wild type orf3b, Δ3b, D614 and G614. **(**C) Visualization of the percentage of full-length orf3b and Δ3b sequences by month. (D) Visualization of the percentage of spike D614 and G614 sequences by month.

To better visualize current circulation of the two distinct orf3b genotypes, we analysed sequence statistics of available resources on NextStrain.org [16] and GISAID sequence depository (Supplementary Table S1). The sequence data were analysed and presented in Figure 3(A). Based on the sequence information of 150,198 SARS-CoV-2 genome sequences collected from December 2019 to September 2020, we found that 23.82% (35771/150198) of SARS-CoV-2 genome sequences carry the Δ3b genotype. Notably, almost all (99.69%) genome sequences with Δ3b genotype contain spike D614G substitution. This is consistent with our phylogenetic analysis (Figure 1) and further suggests the co-evolution of Δ3b genotype and spike D614G substitution.

The sequence data set was further analysed by sorting them into respective months of sample collection and into their respective geographical locations in order to investigate the circulation pattern of Δ3b virus. The number and percentage of sequences with full length orf3b or Δ3b genotype were summarized in Figure 3(B). Consistent with our phylogenetic analysis, SARS-CoV-2 strains carrying Δ3b genotype began to circulate in February (Figure 3C), where the spike G614 genotype coincidentally also began to rise in proportion (Figure 3D). The number of Δ3b virus strains persistently increases in circulation throughout the course of the SARS-CoV-2 pandemic. The proportion of Δ3b genotype has reached 31.86% (4741/14880) in May 2020 and maintained 15–28% from June to September. This suggests that the Δ3b genotype has stably propagated for 8 months (February to September), with up to 20% of circulating viruses lacking the expression of full-length orf3b.

**Figure 4.**
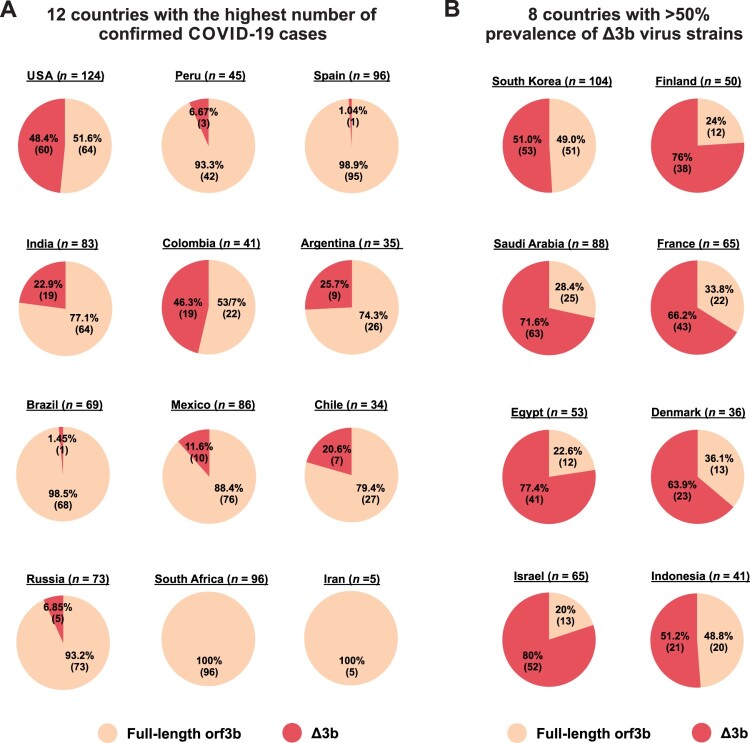
Geographic prevalence of Δ3b viruses. Relative percentage of SARS-CoV-2 virus strains with full-length orf3b and Δ3b genotype was shown. Sequence data from Dec 2019 to Aug 2020 was obtained from NextStrain. The percentage and the number of available sequences for (A) 12 countries with the highest number of confirmed COVID-19 cases based on WHO (https://covid19.who.int/); and (B) 8 countries with more than 50% prevalence of Δ3b virus strains were shown. Portion of full-length orf3b was coloured orange, and portions of Δ3b was coloured red.

Geographically, Δ3b viruses are present in 10 out of 12 countries with the highest number of confirmed COVID-19 cases listed in WHO records (Figure 4A). Particularly, almost half of the available sequences from the USA (48.4%) and Colombia (46.3%) are Δ3b viruses, further confirming that Δ3b persists in the virus circulation. Furthermore, 8 other countries have more than 50% prevalence of Δ3b virus (Figure 4B), including Israel (80%), Egypt (77.4%), Finland (76%), Saudi Arabia (71.6%), France (66.2%), Denmark (63.9%), Indonesia (51.2%), and South Korea (51.0%). The extraordinary prevalence of Δ3b in these countries may suggest a significant serological difference among geographical locations. Our result indicates that precautions should be taken for using orf3b as a target for diagnostic or serological tests.

### Orf3b truncation leads to its loss of interferon antagonism

**Figure 5.**
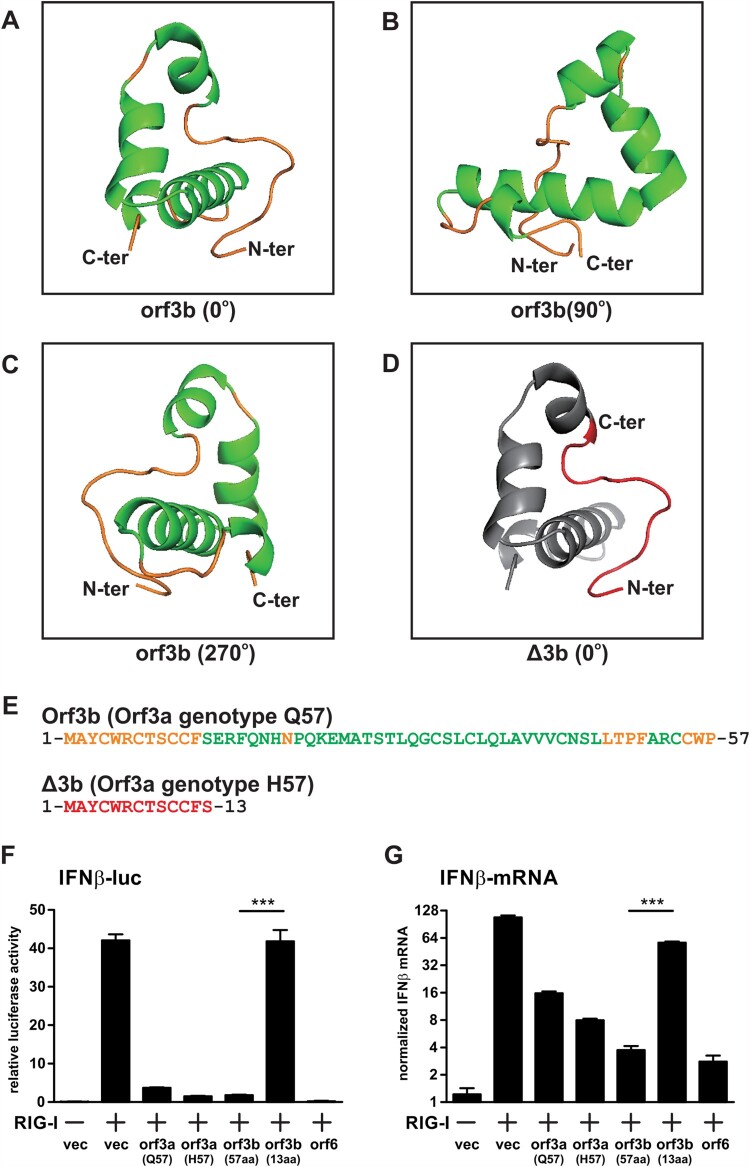
Structure prediction and interferon antagonism of SARS-CoV-2 orf3b and Δ3b. (A-C) In silico structural prediction was performed using Robetta server. Visualization at 0°, 90°, and 270° was shown. Helices were coloured in green and loops were coloured in yellow. **(D)** Visualization of the 13 amino acids correspond to Δ3b in the predicted model. The short peptide was coloured in red and the remaining model in grey for contrast. **(E)** Amino acid sequences of full-length orf3b and Δ3b, coloured with respect to the structure prediction above. **(F-G)** Interferon antagonism by orf3b and Δ3b using **(F)** dual luciferase reporter assay and **(G)** RT-qPCR. vec: empty vector control. Data were statistically analysed by unpaired student’s t-test. *** = *p*<0.0001.

Spontaneous mutations are generated during erroneous replication of viruses. Occasionally, these mutations can result in gain or loss of function of viral proteins, imposing phenotypic changes to the virus. In the case of SARS-CoV-2 orf3b, the two genotypes, full-length orf3b (57a.a.) and Δ3b (13a.a.) respectively, give rise to two significantly different viral protein isoforms. *In silico* structural prediction shows that the full-length SARS-CoV-2 orf3b contains a long, flexible loop at N-terminus and three alpha helices (Figure 5A–C). The three alpha helices fold around to form a compact protein with ring-like structure. In stark contrast, Δ3b only contains the N-terminus flexible loop (Figure 5D; highlighted in red), which is difficult for computational structural analysis. Figure 5(E) highlights the amino acid sequence of full-length orf3b and Δ3b with corresponding colours to their predicted structures.

Based on such drastic change in encoded protein length and structure, we therefore hypothesized that the premature stop codon imposed by Q57H substitution of orf3a would contribute to a loss of function of orf3b in these circulating strains. Our previous findings have showed that both SARS-CoV-2 orf3a and orf3b are able to antagonize type-I interferon activation [14]. Thus, we performed similar dual luciferase assay to determine the interferon antagonism of orf3a with Q57 and H57, as well as full-length orf3b and Δ3b. 293FT cells were co-transfected with interferon β (IFNβ) luciferase reporter, Renilla control luciferase reporter, expression plasmids for an active form of RIG-I (RIG-IN) and expression plasmids for the viral proteins. Orf6 was used as a control for interferon antagonism. Consistent with our published result, SARS-CoV-2 orf3a and full-length orf3b can significantly inhibit IFNβ activation, though not as potent as orf6 (Figure 5F). While H57 orf3a retained its interferon-antagonizing function, the 13a.a. truncated form of orf3b (Δ3b) has lost the ability to inhibit interferon induction. Similar result was also observed when IFNβ transcripts were quantitated by RT-qPCR (Figure 5G). These data reflect that the Q57H substitution resulted in a loss of function of orf3b by introducing an early stop codon. It is highly likely that the phenotype of SARS-CoV-2 strains with Δ3b is altered to some extent.

## Discussion

SARS-CoV-2 belongs to sarbecovirus and its overall genomic structure is similar to that of SARS-CoV. Like other betacoronaviruses, the sarbecovirus genome contains a 5’ untranslated region (UTR); orf1ab encoding a long polypeptide that gives rise to 16 mature non-structure proteins (NSP 1-16); structural genes encoding surface spike glycoprotein, envelope and membrane protein; accessory genes including orf3a, orf3b, 6, 7a, 7b, 8 and 9b; nucleoprotein gene and 3’UTR [4]. Within the orf3 genomic region, the full length orf3 sequences (frame 1) of both SARS-CoV and SARS-CoV-2 encode the orf3a protein with high similarity [21]. The SARS-CoV orf3a has been shown to inhibit interferon signalling while the SARS-CoV-2 orf3a has been characterized as a proapoptotic factor [22]. For the SARS-CoV-2 orf3a, more than 50 non-synonymous mutations have been identified and the most frequent and dominant one is Q57H [21]. In an early proteomic study, Gordon et al. briefly mentioned a premature stop codon in SARS-CoV-2 orf3b at position 14 (E14) which is corresponding to the Q57H mutation in orf3a in their Extended Data Figure 1(b) [10]. Since then, no further detailed analysis on the prevalence of orf3a Q57H and truncation of orf3b has been reported.

In the past nine months, SARS-CoV-2 has evolved rapidly and accumulated a considerable amount of mutations throughout the pandemic. While some of them are sporadic, certain mutations become dominant and such substitutions may have functional impacts on virus life cycle and infected hosts such as altered viral infectivity, replication, dissemination, and pathogenesis. One of the well-studied examples is spike protein D614G substitution, of which the G614 genotype is now dominant in circulating strains with higher infectivity [6]. Another example is the truncation/deletion of SARS-CoV-2 orf8 protein [9, 23]. These weaken orf8-deleted viruses which have not dominated or persisted in the human population were only found in certain locations such as Singapore, Taiwan, Australia, Bangladesh, and Spain [9]. We have recently characterized the SARS-CoV-2 orf8 as a highly immunologic secreted protein [24]. It will be of great interest to investigate the molecular mechanism of loss of orf8 on the viral replication and pathogenesis.

In this study, we have identified the global circulation of a persistent orf3b-truncated SARS-CoV-2 population, the Δ3b viruses, in the current pandemic. We analysed over 150,000 sequences extracted from GISAID depository and constructed a phylogenetic tree with 106 representative sequences (Figure 1). We have demonstrated a distinct clade where all sequences contain orf3a Q57H substitution. It is noted that this substitution of orf3a has introduced a premature stop codon to orf3b, which utilizes a different reading frame (Figure 2). This creates a truncated form of orf3b, Δ3b, which only encodes 13 amino acids, contrast to the full-length orf3b with 57 amino acids. Our phylogenetic analysis in this study demonstrated that Δ3b virus strains cluster together to form a distinct population.

To determine the extent of circulation of Δ3b viruses, we compiled and analysed sequence data from GISAID and NextStrain.org by month (Figure 3). We found that 23.82% of circulating sequences carry Δ3b in average. Most importantly, not only does the number of Δ3b sequences increases during the pandemic, but its emergence also coincides with the spike D614G viruses. Moreover, unlike truncations/mutations in other accessory proteins such as above-mentioned orf8 protein, SARS-CoV-2 Δ3b genotype is not localized in certain areas. In our geographic analysis, Δ3b viruses exist worldwide, including ten of the twelve countries with highest confirmed COVID-19 cases (Figure 4), such as the USA (48.4%) and Colombia (46.3%). Also, more than 50% of available genome sequences in some locations are Δ3b, such as Israel (80%), Egypt (77.4%) and Saudi Arabia (71.6%). This shows that not only Δ3b is circulating globally with considerable prevalence, but also significant serological differences may exist. Thus, our findings provide invaluable information regarding the development of accurate diagnostic tools for COVID-19. Recently, human antibodies against SARS-CoV-2 orf3b has been detected in patient serum and orf3b was proposed as a novel serological marker for early and late infection [12] However, based on the differences we observed, the effectiveness of utilizing orf3b for diagnosis is doubtful as Δ3b viruses are persistent globally, with increasing prevalence in different locations. Nevertheless, the 13 amino acid of Δ3b may still be immunogenic, but whether it can be expressed as a proper immunogen or elicit an immune response comparable to full-length orf3b is still not known. More information such as orf3b epitope mapping and the differential expression of full-length orf3b and Δ3b during viral infection is essential for the consideration of using SARS-CoV-2 orf3b as a diagnostic marker. There are currently only two H57 cases reported in Hong Kong. To further testify the hypothesis that Δ3b may not able to induce the production orf3b-specific antibodies, we applied the Luciferase Immunoprecipitation System (LIPS) assay for the quantification of anti-orf3b antibody in serum samples of COVID-19 patients. Similar to the previous study on the use of orf3b and orf8 for the COVID-19 diagnosis [12], around 70% of COVID-19 positive sera showed orf3b-positive signals (Figure S1), indicating the presence of anti-orf3b antibodies. However, no positive readings were obtained using the serum samples of two Hong Kong H57 cases.

We have also performed structural analysis on orf3b in order to predict changes in function for Δ3b (Figure 5A–E). Δ3b exists as a short peptide that is difficult for in silico structure prediction, and we hypothesized that it can hardly be expressed into a functional protein. As we have previously identified the SARS-CoV-2 orf3b as an interferon antagonist, we further tested the hypothesis by looking at its interferon antagonism through dual luciferase assay and RT-qPCR (Figure 5F, G). Indeed, Δ3b has completely lost the interferon antagonism when compared to full-length orf3b. As shown in our phylogenetic and sequence statistical analysis, all Δ3b carry G614 genotype. This implies that Δ3b viruses have increased infectivity and possibly reduced disease severity. The virus also persists in 15-28% of circulation. As such, the potential for SARS-CoV-2 to become an endemic cannot be neglected. Subtle changes such as Δ3b prevalence should be carefully monitored and must be coupled with molecular findings of the relevant viral proteins. However, to elucidate the detailed function of orf3b and the effect of Δ3b on SARS-CoV-2 replication, more sophisticated methods, such as recombinant infectious clones, should be applied. Moreover, clinical specimens of full-length orf3b and Δ3b (virus isolates with orf3a Q57 and H57 substitutions) could be a valuable tool to evaluate the clinical relevance of Δ3b variants. We have attempted to grow the only two Δ3b clinical samples found in Hong Kong, but no virus could be successfully isolated. Whether the Δ3b genotype will introduce a strong change in the infectivity and pathogenesis similar to spike G614 remains unknown, as our knowledge of the function of orf3b protein as well as clinical correlations are lacking. But the finding of the increasing global dominance Δ3b variant viruses suggests that the virus fitness is unlikely to be compromised despite the functional loss of interferon antagonism by orf3b which is likely to be compensated by NSP13, NSP14, NSP15 and orf6. Ultimately, further *in vivo* studies using mice or hamster model can shed light on the function of orf3b on the pathogenesis and transmissibility of SARS-CoV-2.

One of the key questions is whether SARS-CoV-2 evolves into a less lethal virus that adapts and circulates within our human population for a long period of time. Regarding to the evolution, virus may tend to decrease its virulence and increase its transmissibility for the better human adaptation and circulation. It will be of interest to understand whether loss of orf3b decreases the virulence and spike D614G substitution increases the transmissibility simultaneously. It is also important to analyse any correlation of Δ3b/D614G genotype and clinical manifestation of COVID-19 patients.

In conclusion, our study has demonstrated the increasing trend of loss of orf3b in the current circulating SARS-CoV-2. Phylogenetic analysis shows that Δ3b viruses form a distinct clade and its emergence coincide with spike G614. The Δ3b virus variants persist in the circulation, with increasing trend throughout the pandemic, and may result in serological differences among countries. We also showed that Δ3b has lost its interferon antagonism, which reflects that Δ3b genotype can bring about a loss of function phenotype in SARS-CoV-2. Finally, careful considerations should be taken for using orf3b as a diagnostic marker for COVID-19 due to the global emergence of Δ3b variants.

## Supplementary Material

Table_S1_cc_updated.docx

Figure_S2_updated.docx

Figure_S1_updated.docx
