# Supplementary material for: Loss of orf3b in the circulating SARS-CoV-2 strains
Source: Emerg Microbes Infect. 2020 Dec 24;9(1):2685–96. doi: 10.1080/22221751.2020.1852892 (PMC7782295; doi:10.1080/22221751.2020.1852892)
Supplement: Table_S1_cc_updated.docx [file TEMI_A_1852892_SM1186.docx]

|  |  |  |  |
| --- | --- | --- | --- |
| !"#$#%&'())!"#$#%&'()&!"#$#%&'(&*!"#$#%&'(&+ | , | , | - . ,/0$12" 34 ! 1" 56,32 6$74 3 7 . 4 6. 0 3 1 0 ! 3 , 14 $ /3 0 4$3 3 75 8 . 710 |
| !"#$#%&'(&( | , | , | - . ,/0$12" 34 ! 1" 56,32 6$74 |


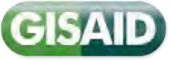
 3 7 . 4 3 1 0 ! 3 , 14 $ /3 0 4$3 370 715 8 . !"#$#%&'(&9!"#$#%&'(&%!"#$#%&'(&'!"#$#%&'(&:!"#$#%&'(&)!"#$#%&'9**!"#$#%&'9*+!"#$#%&'9*;!"#$#%&'9*(!"#$#%&'9*%!"#$#%&'9*'!"#$#%&'9*:!"#$#%&'9*)!"#$#%&'9*&

|  | , | , | - . ,/0$12" 34 ! 1" 56,32 6$74 3 7 . 4 6. 0 3 1 0 ! 3 , 14 $ /3 0 4$3 3 75 8 . 710 |
| --- | --- | --- | --- |
| !"#$#%&'9+* | , | , | - . ,/0$12" 34 ! 1" 56,32 6$74  3 7 . 4 3 1 0 ! 3 , 14 $ /3 0 4$3 370 715 8 . |
| !"#$#%&'9+%!"#$#%&'9+' | , | , | - . ,/0$12" 34 ! 1" 56,32 6$74 3 7 . 4 6. 0 3 1 0 ! 3 , 14 $ /3 0 4$3 3 75 8 . 710 |
| !"#$#%&'9+:!"#$#%&'9+)!"#$#%&'9+&!"#$#%&'9;* | , | , | - . ,/0$12" 34 ! 1" 56,32 6$74  3 7 . 4 3 1 0 ! 3 , 14 $ /3 0 4$3 370 715 8 . |
| !"#$#%&'9;+!"#$#%&'9;;!"#$#%&'9;(!"#$#%&'9;9 | , | , | - . ,/0$12" 34 ! 1" 56,32 6$74 3 7 . 4 6. 0 3 1 0 ! 3 , 14 $ /3 0 4$3 3 75 8 . 710 |
| !"#$#%&'9;'!"#$#%&'9;:!"#$#%&'9;)!"#$#%&'9;& !"#$#%&'9(*!"#$#%&'9(+!"#$#%&'9(;!"#$#%&'9(9 !"#$#%&'9(%!"#$#%&'9(' | , | , | - . ,/0$12" 34 ! 1" 56,32 6$74  3 7 . 4 3 1 0 ! 3 , 14 $ /3 0 4$3 370 715 8 . |
| !"#$#%&'9(:!"#$#%&'9(& | , | , | - . ,/0$12" 34 ! 1" 56,32 6$74 3 7 . 4 6. 0 3 1 0 ! 3 , 14 $ /3 0 4$3 3 75 8 . 710 |
| !"#$#%&'99*!"#$#%&'99+!"#$#%&'99;!"#$#%&'99(  !"#$#%&'999!"#$#%&'99% | , | , | - . ,/0$12" 34 ! 1" 56,32 6$74  3 7 . 4 3 1 0 ! 3 , 14 $ /3 0 4$3 370 715 8 . |
| !"#$#%&'99' | , | , | - . ,/0$12" 34 ! 1" 56,32 6$74 3 7 . 4 6. 0 3 1 0 ! 3 , 14 $ /3 0 4$3 3 75 8 . 710 |
| !"#$#%&'99:!"#$#%&'99) | -3<5 $ | $ - $1 | .=.$3 . 633 7 6 05 3 !" . |
| !"#$#%&'99& | 3 4 4  .- 5 35 3 | 3 4  4.- 5 35 3 | 06 63=>>74 |
| !"#$#%&'9%+!"#$#%&'9%; | " 36 5 7?3 | 4.?  4 | >,3/3620 // 3/ 0 /5 / 0 //  3/ 4/-2 66/30/5-2 |
| !"#$#%&'9%( | 1 @ A  " 5 7?3 7 | 4.?  4 | >,3/3620 // 3/ 0 36/366/30/  5-2 |
| !"#$#%&'9%9 | 7 34.  47 >?3 > | 4.?  4 | >,3/3620 // 3/ 0 !635=66/3  0/5-2 |
| !"#$#%&'9%% | " 36 5 7?3 | 4.?  4 | >,3/3620 // 3/ 0 /5 / 0 //  3/ 4/-2 66/30/5-2 |

!"#$#%&'9%'!"#$#%&'9%:!"#$#%&'9%)!"#$#%&'9%&!"#$#%&'9'*!"#$#%&'9'+!"#$#%&'9';!"#$#%&'9'(!"#$#%&'9'9!"#$#%&'9'%!"#$#%&'9''!"#$#%&'9':!"#$#%&'9')!"#$#%&'9'&!"#$#%&'9:*!"#$#%&'9:+!"#$#%&'9:;!"#$#%&'9:(!"#$#%&'9:9!"#$#%&'9:%!"#$#%&'9:'!"#$#%&'9::!"#$#%&'9:)!"#$#%&'9:& !"#$#%&'9)*!"#$#%&'9)+!"#$#%&'9);!"#$#%&'9)(!"#$#%&'9)9!"#$#%&'9)%!"#$#%&'9)'!"#$#%&'9):!"#$#%&'9))!"#$#%&'9)&!"#$#%&'9&*!"#$#%&'9&+!"#$#%&'9&;!"#$#%&'9&(!"#$#%&'9&9!"#$#%&'9&%!"#$#%&'9&'!"#$#%&'9&:

- " 5 $- . - " 5 $- . 7/33 >> $. 47/"$

!"#$#%&'%**!"#$#%&'%*+!"#$#%&'%*;!"#$#%&'%*(!"#$#%&'%*9!"#$#%&'%*%!"#$#%&'%*'!"#$#%&'%*:!"#$#%&'%*)!"#$#%&'%*&!"#$#%&'%+*!"#$#%&'%++!"#$#%&'%+;!"#$#%&'%+(!"#$#%&'%+9!"#$#%&'%+%!"#$#%&'%+'!"#$#%&'%+:!"#$#%&'%+)!"#$#%&'%+&!"#$#%&'%;*!"#$#%&'%;+!"#$#%&'%;(!"#$#%&'%;% !"#$#%&'%;'!"#$#%&'%;:!"#$#%&'%;)!"#$#%&'%;&!"#$#%&'%(*!"#$#%&'%(+!"#$#%&'%(;!"#$#%&'%((!"#$#%&'%(9!"#$#%&'%(%!"#$#%&'%('!"#$#%&'%(:!"#$#%&'%()!"#$#%&'%(&!"#$#%&'%9*!"#$#%&'%9+!"#$#%&'%9;!"#$#%&'%9(!"#$#%&'%99!"#$#%&'%9%!"#$#%&'%9:!"#$#%&'%9)!"#$#%&'%9&!"#$#%&'%%* !"#$#%&'%%+!"#$#%&'%%;!"#$#%&'%%(!"#$#%&'%%9!"#$#%&'%%%!"#$#%&'%%'!"#$#%&'%%:!"#$#%&'%%)!"#$#%&'%%&!"#$#%&'%'*!"#$#%&'%'+!"#$#%&'%';!"#$#%&'%'(!"#$#%&'%'9!"#$#%&'%'%!"#$#%&'%''!"#$#%&'%':!"#$#%&'%')

" 35 3 $ - @ > 455 -223 4 /B-22,1 <$

!"#$#%&'%'&!"#$#%&'%:*!"#$#%&'%:+!"#$#%&'%:;!"#$#%&'%:(!"#$#%&'%:9!"#$#%&'%:%!"#$#%&'%:'!"#$#%&'%::!"#$#%&'%:)!"#$#%&'%:&!"#$#%&'%)*!"#$#%&'%)+!"#$#%&'%);!"#$#%&'%)(!"#$#%&'%)9!"#$#%&'%)%!"#$#%&'%)'!"#$#%&'%):!"#$#%&'%))!"#$#%&'%)&!"#$#%&'%&*!"#$#%&'%&+!"#$#%&'%&; !"#$#%&'%&(!"#$#%&'%&9!"#$#%&'%&%!"#$#%&'%&'!"#$#%&'%&:!"#$#%&'%&)!"#$#%&'%&&!"#$#%&''**!"#$#%&''*+!"#$#%&''*;!"#$#%&''*(!"#$#%&''*9!"#$#%&''*%!"#$#%&''*'!"#$#%&''*:!"#$#%&''*)!"#$#%&''*&!"#$#%&''+*!"#$#%&''++!"#$#%&''+;!"#$#%&''+(!"#$#%&''+9!"#$#%&''+%!"#$#%&''+'

!"#$#%&''+:!"#$#%&''+)!"#$#%&''+&!"#$#%&'';*!"#$#%&'';+!"#$#%&'';;!"#$#%&'';(!"#$#%&'';9!"#$#%&'';%!"#$#%&'';'

?3. 3 $ $ $?3 A

3

!"#$#%&'';)!"#$#%&'';&!"#$#%&''(*!"#$#%&''(+!"#$#%&''(;!"#$#%&''((!"#$#%&''(9!"#$#%&''(%!"#$#%&''('!"#$#%&''(:!"#$#%&''()!"#$#%&''(&!"#$#%&''9*!"#$#%&''9+!"#$#%&''9;!"#$#%&''9%!"#$#%&''9'!"#$#%&''9:!"#$#%&''9)!"#$#%&''9&!"#$#%&''%*!"#$#%&''%+!"#$#%&''%;!"#$#%&''%( !"#$#%&''%9!"#$#%&''%%!"#$#%&''%'!"#$#%&''%:!"#$#%&''%)!"#$#%&''%&!"#$#%&'''*!"#$#%&'''+!"#$#%&''';!"#$#%&'''(!"#$#%&'''9!"#$#%&'''%!"#$#%&''''!"#$#%&''':!"#$#%&''')!"#$#%&'''&!"#$#%&'':*!"#$#%&'':;!"#$#%&'':(!"#$#%&'':9!"#$#%&'':%!"#$#%&'':'

AC?5 AC?5 3$ 3-3 ? =,/ / / /. " $, D ,6

!"#$#%&''::!"#$#%&'':)!"#$#%&'':&!"#$#%&'')*!"#$#%&'')+!"#$#%&'');!"#$#%&'')(!"#$#%&'')9!"#$#%&'')%!"#$#%&'')'!"#$#%&''):!"#$#%&''))!"#$#%&'')&!"#$#%&''&*!"#$#%&''&+!"#$#%&''&;!"#$#%&''&(!"#$#%&''&9!"#$#%&''&%!"#$#%&''&'!"#$#%&''&:!"#$#%&''&)!"#$#%&''&&!"#$#%&':**

!"#$#%&':*+!"#$#%&':*;!"#$#%&':*(!"#$#%&':*9!"#$#%&':*%!"#$#%&':*'!"#$#%&':*:!"#$#%&':*)!"#$#%&':*&!"#$#%&':+*!"#$#%&':++!"#$#%&':+;!"#$#%&':+(!"#$#%&':+9!"#$#%&':+%!"#$#%&':+'!"#$#%&':+:!"#$#%&':+)!"#$#%&':+&!"#$#%&':;*!"#$#%&':;+!"#$#%&':;;!"#$#%&':;(!"#$#%&':;9

!"#$#%&':;%!"#$#%&':;'!"#$#%&':;:!"#$#%&':;)!"#$#%&':;&!"#$#%&':(*!"#$#%&':(+!"#$#%&':(;!"#$#%&':((!"#$#%&':(9!"#$#%&':('!"#$#%&':(:!"#$#%&':()!"#$#%&':(&!"#$#%&':9*!"#$#%&':9+!"#$#%&':9;!"#$#%&':9(!"#$#%&':99!"#$#%&':9%!"#$#%&':9'!"#$#%&':9:!"#$#%&':9)!"#$#%&':9&

!"#$#%&':%*!"#$#%&':%+!"#$#%&':%;!"#$#%&':%(!"#$#%&':%9!"#$#%&':%%!"#$#%&':%'!"#$#%&':%:!"#$#%&':%)!"#$#%&':%&!"#$#%&':'*!"#$#%&':'+!"#$#%&':';!"#$#%&':'(!"#$#%&')+&!"#$#%&');*!"#$#%&');+!"#$#%&');;!"#$#%&');(!"#$#%&');9!"#$#%&');%!"#$#%&');'!"#$#%&');:!"#$#%&');)

!"#$#%&');&!"#$#%&')(*!"#$#%&')(+!"#$#%&')(;!"#$#%&')((!"#$#%&')(9!"#$#%&')(%!"#$#%&')('!"#$#%&')(:!"#$#%&')()!"#$#%&')(&!"#$#%&')9*!"#$#%&')9+!"#$#%&')9;!"#$#%&')9(!"#$#%&')99!"#$#%&')9%!"#$#%&')9'!"#$#%&')9:!"#$#%&')9)!"#$#%&')9&!"#$#%&')%*!"#$#%&')%+!"#$#%&')%;

!"#$#%&')%(!"#$#%&')%9!"#$#%&')%%!"#$#%&')%'!"#$#%&')%:!"#$#%&')%)!"#$#%&')%&!"#$#%&')'*!"#$#%&')'+!"#$#%&')';!"#$#%&')'(!"#$#%&')'9!"#$#%&')'%!"#$#%&')''!"#$#%&')':!"#$#%&')')

"$3 "$33 "$33 ?

?

!"#$#%&'))&!"#$#%&')&*!"#$#%&')&+!"#$#%&')&(!"#$#%&')&9!"#$#%&')&%!"#$#%&')&'!"#$#%&')&:!"#$#%&')&)!"#$#%&')&&!"#$#%&'&**!"#$#%&'&*+!"#$#%&'&*;!"#$#%&'&*(!"#$#%&'&*9!"#$#%&'&*%!"#$#%&'&*'!"#$#%&'&*:!"#$#%&'&*)!"#$#%&'&*&!"#$#%&'&+*!"#$#%&'&++!"#$#%&'&+;!"#$#%&'&+( !"#$#%&'&+9!"#$#%&'&+%!"#$#%&'&+'!"#$#%&'&+:!"#$#%&'&+)!"#$#%&'&+&!"#$#%&'&;*!"#$#%&'&;+!"#$#%&'&;;!"#$#%&'&;(!"#$#%&'&;9!"#$#%&'&;%!"#$#%&'&;'!"#$#%&'&;:

- A 4$ - A 4$ 3 . / /0 5. ,

!"#$#%&'&;)!"#$#%&'&;&!"#$#%&'&(*!"#$#%&'&(+!"#$#%&'&(;!"#$#%&'&((!"#$#%&'&(9!"#$#%&'&(%!"#$#%&'&('!"#$#%&'&()!"#$#%&'&(&!"#$#%&'&9*!"#$#%&'&9+!"#$#%&'&9;!"#$#%&'&9(!"#$#%&'&99!"#$#%&'&9%!"#$#%&'&9'!"#$#%&'&9:!"#$#%&'&9)!"#$#%&'&9&!"#$#%&'&%*!"#$#%&'&%+!"#$#%&'&%; !"#$#%&'&%(!"#$#%&'&%9!"#$#%&'&%%!"#$#%&'&%'!"#$#%&'&%:!"#$#%&'&%)!"#$#%&'&%&!"#$#%&'&'*!"#$#%&'&'+!"#$#%&'&';!"#$#%&'&'(!"#$#%&'&'9!"#$#%&'&'%!"#$#%&'&''!"#$#%&'&':!"#$#%&'&')!"#$#%&'&:*!"#$#%&'&:+!"#$#%&'&:;!"#$#%&'&:(!"#$#%&'&:9!"#$#%&'&:%!"#$#%&'&:'!"#$#%&'&::

!"#$#%&'&:)!"#$#%&'&:&!"#$#%&'&)*!"#$#%&'&)+!"#$#%&'&);!"#$#%&'&)(!"#$#%&'&)9!"#$#%&'&)%!"#$#%&'&)'!"#$#%&'&):!"#$#%&'&))!"#$#%&'&)&!"#$#%&'&&*!"#$#%&'&&+!"#$#%&'&&;!"#$#%&'&&(!"#$#%&'&&9!"#$#%&'&&%!"#$#%&'&&'!"#$#%&'&&:!"#$#%&'&&)!"#$#%&'&&&!"#$#%&:***!"#$#%&:**+

!"#$#%&:**;!"#$#%&:**(!"#$#%&:**9!"#$#%&:**%!"#$#%&:**'!"#$#%&:**:!"#$#%&:**)!"#$#%&:**&!"#$#%&:*+*!"#$#%&:*++!"#$#%&:*+;!"#$#%&:*+(!"#$#%&:*+9!"#$#%&:*+%!"#$#%&:*+'!"#$#%&:*+:!"#$#%&:*+)!"#$#%&:*+&!"#$#%&:*;*!"#$#%&:*;+!"#$#%&:*;;!"#$#%&:*;(!"#$#%&:*;9!"#$#%&:*;%

!"#$#%&:*;'!"#$#%&:*;:!"#$#%&:*;)!"#$#%&:*;&!"#$#%&:*(*!"#$#%&:*(+!"#$#%&:*(;!"#$#%&:*((!"#$#%&:*(9!"#$#%&:*(%!"#$#%&:*('!"#$#%&:*(:!"#$#%&:*()!"#$#%&:*(&!"#$#%&:*9*!"#$#%&:*9+!"#$#%&:*9;!"#$#%&:*9(!"#$#%&:*99!"#$#%&:*9%!"#$#%&:*9'!"#$#%&:*9:!"#$#%&:*9)!"#$#%&:*9&

!"#$#%&:*%*!"#$#%&:*%+!"#$#%&:*%;!"#$#%&:*%(!"#$#%&:*%9!"#$#%&:*%%!"#$#%&:*%'!"#$#%&:*%:!"#$#%&:*%)!"#$#%&:*%&!"#$#%&:*'*!"#$#%&:*'+!"#$#%&:*';!"#$#%&:*'(!"#$#%&:*'9!"#$#%&:*''!"#$#%&:*':!"#$#%&:*')!"#$#%&:*'&!"#$#%&:*:*!"#$#%&:*:+!"#$#%&:*:;!"#$#%&:*:(!"#$#%&:*:%

!"#$#%&:*:'!"#$#%&:*::!"#$#%&:*:)!"#$#%&:*:&!"#$#%&:*)*!"#$#%&:*)+!"#$#%&:*);!"#$#%&:*)(!"#$#%&:*)9!"#$#%&:*)%!"#$#%&:*)'!"#$#%&:*):!"#$#%&:*))!"#$#%&:*)&!"#$#%&:*&*!"#$#%&:*&+!"#$#%&:*&;!"#$#%&:*&(!"#$#%&:*&9!"#$#%&:*&%!"#$#%&:*&'!"#$#%&:*&:!"#$#%&:*&)!"#$#%&:+**

!"#$#%&:+*+!"#$#%&:+*;!"#$#%&:+*(!"#$#%&:+*9!"#$#%&:+*%!"#$#%&:+*'!"#$#%&:+*:!"#$#%&:+*)!"#$#%&:+*&!"#$#%&:++*!"#$#%&:+++!"#$#%&:++;!"#$#%&:++(!"#$#%&:++9!"#$#%&:++%!"#$#%&:++'!"#$#%&:++:!"#$#%&:++)!"#$#%&:++&!"#$#%&:+;*!"#$#%&:+;+!"#$#%&:+;;!"#$#%&:+;(!"#$#%&:+;9

!"#$#%&:+;%!"#$#%&:+;'!"#$#%&:+;:!"#$#%&:+;)!"#$#%&:+;&!"#$#%&:+(*!"#$#%&:+(+!"#$#%&:+(;!"#$#%&:+((!"#$#%&:+(9!"#$#%&:+(%!"#$#%&:+('!"#$#%&:+(:!"#$#%&:+()!"#$#%&:+(&!"#$#%&:+9*!"#$#%&:+9+!"#$#%&:+9(!"#$#%&:+99!"#$#%&:+9%!"#$#%&:+9'!"#$#%&:+9:!"#$#%&:+9)!"#$#%&:+9&

!"#$#%&:+%*!"#$#%&:+%+!"#$#%&:+%;!"#$#%&:+%(!"#$#%&:+%9!"#$#%&:+%%!"#$#%&:+%'!"#$#%&:+%:!"#$#%&:+%)!"#$#%&:+%&!"#$#%&:+'*!"#$#%&:+'+!"#$#%&:+';!"#$#%&:+'(!"#$#%&:+'9!"#$#%&:+'%!"#$#%&:+''!"#$#%&:+':!"#$#%&:+')!"#$#%&:+'&!"#$#%&:+:*!"#$#%&:+:+!"#$#%&:+:;!"#$#%&:+:( !"#$#%&:+:9!"#$#%&:+:%!"#$#%&:+:'!"#$#%&:+::!"#$#%&:+:)!"#$#%&:+:&!"#$#%&:+)*!"#$#%&:+)+!"#$#%&:+);!"#$#%&:+)(!"#$#%&:+)9!"#$#%&:+)%!"#$#%&:+)'!"#$#%&:+):!"#$#%&:+))!"#$#%&:+&*!"#$#%&:+&+!"#$#%&:+&;!"#$#%&:+&(!"#$#%&:+&9!"#$#%&:+&%!"#$#%&:+&'!"#$#%&:+&:!"#$#%&:+&& !"#$#%&:;**!"#$#%&:;*+!"#$#%&:;*;!"#$#%&:;*(!"#$#%&:;*9!"#$#%&:;*%!"#$#%&:;*'!"#$#%&:;*:!"#$#%&:;*)!"#$#%&:;*&!"#$#%&:;+*!"#$#%&:;++!"#$#%&:;+;!"#$#%&:;+(!"#$#%&:;+9!"#$#%&:;+%!"#$#%&:;+'!"#$#%&:;+:!"#$#%&:;+)!"#$#%&:;+&!"#$#%&:;;*!"#$#%&:;;+!"#$#%&:;;;!"#$#%&:;;( !"#$#%&:;;9!"#$#%&:;;%!"#$#%&:;;'!"#$#%&:;;:!"#$#%&:;;)!"#$#%&:;;&!"#$#%&:;(*!"#$#%&:;(+!"#$#%&:;(;!"#$#%&:;((

!"#$#%&:((+ $ $. .A=+&

?6.

|  | $ $. |  | 457$ $. < 4 !56 0 06 . $0 .A=+& 7EFGG G=H |
| --- | --- | --- | --- |
| !"#$#%&:;(9!"#$#%&:;(%!"#$#%&:;(' | $ $ |  | 5A 8 6. . / < 4 !56 0 0  6 . $0 .A=+& 7EFGG G=H |
| !"#$#%&:;(:!"#$#%&:;()!"#$#%&:;(& | $ $. |  | 457$ $. < 4 !56 0 06 . $0 .A=+& 7EFGG G=H |
| !"#$#%&:;9* | $ $ |  | 5A 8 6. . / < 4 !56 0 0  6 . $0 .A=+& 7EFGG G=H |
| !"#$#%&:;9+ | $ $. |  | 457$ $. < 4 !56 0 06 . $0 .A=+& 7EFGG G=H |
| !"#$#%&:;9; | $ $ |  | 5A 8 6. . / < 4 !56 0 0  6 . $0 .A=+& 7EFGG G=H |
| !"#$#%&:;9( | $ $. |  | 457$ $. < 4 !56 0 06 . $0 .A=+& 7EFGG G=H |
| !"#$#%&:;99!"#$#%&:;9% | $ $ | .A=+&  ?6. | 5A 8 6. . / < 4 !56 0 0  6 . $0 .A=+& 7EFGG G=H |
| !"#$#%&:;9'!"#$#%&:;9:!"#$#%&:;9) | $ $. | .A=+&  ?6. | 457$ $. < 4 !56 0 06 . $0 .A=+& 7EFGG G=H |
| !"#$#%&:;9& | $ $ | .A=+&  ?6. | 5A 8 6. . / < 4 !56 0 0  6 . $0 .A=+& 7EFGG G=H |
| !"#$#%&:;%*!"#$#%&:;%+ | $ $. | .A=+&  ?6. | 457$ $. < 4 !56 0 06 . $0 .A=+& 7EFGG G=H |
| !"#$#%&:;%; | $ $ | .A=+&  ?6. | 5A 8 6. . / < 4 !56 0 0  6 . $0 .A=+& 7EFGG G=H |
| !"#$#%&:;%(!"#$#%&:;%9!"#$#%&:;%%!"#$#%&:;%' !"#$#%&:;%:!"#$#%&:;%)!"#$#%&:;%& | $ $. | .A=+&  ?6. | 457$ $. < 4 !56 0 06 . $0 .A=+& 7EFGG G=H |
| !"#$#%&:;'*!"#$#%&:;'+ | $ $ | .A=+&  ?6. | 5A 8 6. . / < 4 !56 0 0  6 . $0 .A=+& 7EFGG G=H |
| !"#$#%&:;'; | $ $. | .A=+&  ?6. | 457$ $. < 4 !56 0 06 . $0 .A=+& 7EFGG G=H |
| !"#$#%&:;'( | $ $ | .A=+&  ?6. | 5A 8 6. . / < 4 !56 0 0  6 . $0 .A=+& 7EFGG G=H |
| !"#$#%&:;'9!"#$#%&:;'%!"#$#%&:;'' | $ $. | .A=+&  ?6. | 457$ $. < 4 !56 0 06 . $0 .A=+& 7EFGG G=H |
| !"#$#%&:;':!"#$#%&:;') | $ $ | .A=+&  ?6. | 5A 8 6. . / < 4 !56 0 0  6 . $0 .A=+& 7EFGG G=H |
| !"#$#%&:;'&!"#$#%&:;:*!"#$#%&:;:+ | $ $. | .A=+&  ?6. | 457$ $. < 4 !56 0 06 . $0 .A=+& 7EFGG G=H |
| !"#$#%&:;:; | $ $ | .A=+&  ?6. | 5A 8 6. . / < 4 !56 0 0  6 . $0 .A=+& 7EFGG G=H |
| !"#$#%&:;:(!"#$#%&:;:9 | $ $. | .A=+&  ?6. | 457$ $. < 4 !56 0 06 . $0 .A=+& 7EFGG G=H |
| !"#$#%&:;:%!"#$#%&:;:'!"#$#%&:;::!"#$#%&:;:) | $ $ | .A=+&  ?6. | 5A 8 6. . / < 4 !56 0 0  6 . $0 .A=+& 7EFGG G=H |
| !"#$#%&:;:& | $ $. | .A=+&  ?6. | 457$ $. < 4 !56 0 06 . $0 .A=+& 7EFGG G=H |
| !"#$#%&:;)*!"#$#%&:;)+ | $ $ | .A=+&  ?6. | 5A 8 6. . / < 4 !56 0 0  6 . $0 .A=+& 7EFGG G=H |
| !"#$#%&:;);!"#$#%&:;)(!"#$#%&:;)9!"#$#%&:;)% !"#$#%&:;)'!"#$#%&:;): | $ $. | .A=+&  ?6. | 457$ $. < 4 !56 0 06 . $0 .A=+& 7EFGG G=H |
| !"#$#%&:;)) | $ $ | .A=+&  ?6. | 5A 8 6. . / < 4 !56 0 0  6 . $0 .A=+& 7EFGG G=H |
| !"#$#%&:;)& | $ $. | .A=+&  ?6. | 457$ $. < 4 !56 0 06 . $0 .A=+& 7EFGG G=H |
| !"#$#%&:;&*!"#$#%&:;&+!"#$#%&:;&; | $ $ | .A=+&  ?6. | 5A 8 6. . / < 4 !56 0 0  6 . $0 .A=+& 7EFGG G=H |
| !"#$#%&:;&(!"#$#%&:;&9!"#$#%&:;&% | $ $. | .A=+&  ?6. | 457$ $. < 4 !56 0 06 . $0 .A=+& 7EFGG G=H |
| !"#$#%&:;&' | $ $ | .A=+&  ?6. | 5A 8 6. . / < 4 !56 0 0  6 . $0 .A=+& 7EFGG G=H |
| !"#$#%&:;&: | $ $. | .A=+&  ?6. | 457$ $. < 4 !56 0 06 . $0 .A=+& 7EFGG G=H |
| !"#$#%&:;&)!"#$#%&:;&& | $ $ | .A=+&  ?6. | 5A 8 6. . / < 4 !56 0 0  6 . $0 .A=+& 7EFGG G=H |
| !"#$#%&:(**!"#$#%&:(*+ | $ $. | .A=+&  ?6. | 457$ $. < 4 !56 0 06 . $0 .A=+& 7EFGG G=H |
| !"#$#%&:(*;!"#$#%&:(*( | $ $ | .A=+&  ?6. | 5A 8 6. . / < 4 !56 0 0  6 . $0 .A=+& 7EFGG G=H |
| !"#$#%&:(*9!"#$#%&:(*%!"#$#%&:(*'!"#$#%&:(*: !"#$#%&:(*)!"#$#%&:(*&!"#$#%&:(+* | $ $. | .A=+&  ?6. | 457$ $. < 4 !56 0 06 . $0 .A=+& 7EFGG G=H |
| !"#$#%&:(++!"#$#%&:(+;!"#$#%&:(+(!"#$#%&:(+9  !"#$#%&:(+% | $ $ | .A=+&  ?6. | 5A 8 6. . / < 4 !56 0 0  6 . $0 .A=+& 7EFGG G=H |
| !"#$#%&:(+'!"#$#%&:(+: | $ $. | .A=+&  ?6. | 457$ $. < 4 !56 0 06 . $0 .A=+& 7EFGG G=H |
| !"#$#%&:(+) | $ $ | .A=+&  ?6. | 5A 8 6. . / < 4 !56 0 0  6 . $0 .A=+& 7EFGG G=H |
| !"#$#%&:(+&!"#$#%&:(;*!"#$#%&:(;+ | $ $. | .A=+&  ?6. | 457$ $. < 4 !56 0 06 . $0 .A=+& 7EFGG G=H |
| !"#$#%&:(;; | $ $ | .A=+&  ?6. | 5A 8 6. . / < 4 !56 0 0  6 . $0 .A=+& 7EFGG G=H |
| !"#$#%&:(;(!"#$#%&:(;9!"#$#%&:(;% | $ $. | .A=+&  ?6. | 457$ $. < 4 !56 0 06 . $0 .A=+& 7EFGG G=H |
| !"#$#%&:(;' | $ $ | .A=+&  ?6. | 5A 8 6. . / < 4 !56 0 0  6 . $0 .A=+& 7EFGG G=H |
| !"#$#%&:(;: | $ $. | .A=+&  ?6. | 457$ $. < 4 !56 0 06 . $0 .A=+& 7EFGG G=H |
| !"#$#%&:(;)!"#$#%&:(;&!"#$#%&:((* | $ $ | .A=+&  ?6. | 5A 8 6. . / < 4 !56 0 0  6 . $0 .A=+& 7EFGG G=H |

457$ $. < 4 !56 0 06 . $0

.A=+& 7EFGG G=H

!"#$#%&:9((!"#$#%&:9(9 $ $ .A=+&

?6.

| !"#$#%&:((;!"#$#%&:(((!"#$#%&:((9 | $ $ |  | 5A 8 6. . / < 4 !56 0 0  6 . $0 .A=+& 7EFGG G=H |
| --- | --- | --- | --- |
| !"#$#%&:((% | $ $. |  | 457$ $. < 4 !56 0 06 . $0 .A=+& 7EFGG G=H |
| !"#$#%&:((' | $ $ |  | 5A 8 6. . / < 4 !56 0 0  6 . $0 .A=+& 7EFGG G=H |
| !"#$#%&:((: | $ $. |  | 457$ $. < 4 !56 0 06 . $0 .A=+& 7EFGG G=H |
| !"#$#%&:(()!"#$#%&:((& | $ $ |  | 5A 8 6. . / < 4 !56 0 0  6 . $0 .A=+& 7EFGG G=H |
| !"#$#%&:(9*!"#$#%&:(9+!"#$#%&:(9;!"#$#%&:(9( !"#$#%&:(99!"#$#%&:(9%!"#$#%&:(9' | $ $. |  | 457$ $. < 4 !56 0 06 . $0 .A=+& 7EFGG G=H |
| !"#$#%&:(9:!"#$#%&:(9) | $ $ |  | 5A 8 6. . / < 4 !56 0 0  6 . $0 .A=+& 7EFGG G=H |
| !"#$#%&:(9&!"#$#%&:(%* | $ $. | .A=+&  ?6. | 457$ $. < 4 !56 0 06 . $0 .A=+& 7EFGG G=H |
| !"#$#%&:(%+!"#$#%&:(%;!"#$#%&:(%( | $ $ | .A=+&  ?6. | 5A 8 6. . / < 4 !56 0 0  6 . $0 .A=+& 7EFGG G=H |
| !"#$#%&:(%9 | $ $. | .A=+&  ?6. | 457$ $. < 4 !56 0 06 . $0 .A=+& 7EFGG G=H |
| !"#$#%&:(%%!"#$#%&:(%'!"#$#%&:(%: | $ $ | .A=+&  ?6. | 5A 8 6. . / < 4 !56 0 0  6 . $0 .A=+& 7EFGG G=H |
| !"#$#%&:(%) | $ $. | .A=+&  ?6. | 457$ $. < 4 !56 0 06 . $0 .A=+& 7EFGG G=H |
| !"#$#%&:(%&!"#$#%&:('* | $ $ | .A=+&  ?6. | 5A 8 6. . / < 4 !56 0 0  6 . $0 .A=+& 7EFGG G=H |
| !"#$#%&:('+ | $ $. | .A=+&  ?6. | 457$ $. < 4 !56 0 06 . $0 .A=+& 7EFGG G=H |
| !"#$#%&:(';!"#$#%&:('(!"#$#%&:('9 | $ $ | .A=+&  ?6. | 5A 8 6. . / < 4 !56 0 0  6 . $0 .A=+& 7EFGG G=H |
| !"#$#%&:('%!"#$#%&:(''!"#$#%&:(':!"#$#%&:(') | $ $. | .A=+&  ?6. | 457$ $. < 4 !56 0 06 . $0 .A=+& 7EFGG G=H |
| !"#$#%&:('&!"#$#%&:(:* | $ $ | .A=+&  ?6. | 5A 8 6. . / < 4 !56 0 0  6 . $0 .A=+& 7EFGG G=H |
| !"#$#%&:(:+!"#$#%&:(:;!"#$#%&:(:(!"#$#%&:(:9  !"#$#%&:(:% | $ $. | .A=+&  ?6. | 457$ $. < 4 !56 0 06 . $0 .A=+& 7EFGG G=H |
| !"#$#%&:(:' | $ $ | .A=+&  ?6. | 5A 8 6. . / < 4 !56 0 0  6 . $0 .A=+& 7EFGG G=H |
| !"#$#%&:(::!"#$#%&:(:) | $ $. | .A=+&  ?6. | 457$ $. < 4 !56 0 06 . $0 .A=+& 7EFGG G=H |
| !"#$#%&:(:& | $ $ | .A=+&  ?6. | 5A 8 6. . / < 4 !56 0 0  6 . $0 .A=+& 7EFGG G=H |
| !"#$#%&:()*!"#$#%&:()+ | $ $. | .A=+&  ?6. | 457$ $. < 4 !56 0 06 . $0 .A=+& 7EFGG G=H |
| !"#$#%&:();!"#$#%&:()(!"#$#%&:()9!"#$#%&:()% !"#$#%&:()'!"#$#%&:(): | $ $ | .A=+&  ?6. | 5A 8 6. . / < 4 !56 0 0  6 . $0 .A=+& 7EFGG G=H |
| !"#$#%&:()) | $ $. | .A=+&  ?6. | 457$ $. < 4 !56 0 06 . $0 .A=+& 7EFGG G=H |
| !"#$#%&:()& | $ $ | .A=+&  ?6. | 5A 8 6. . / < 4 !56 0 0  6 . $0 .A=+& 7EFGG G=H |
| !"#$#%&:(&* | $ $. | .A=+&  ?6. | 457$ $. < 4 !56 0 06 . $0 .A=+& 7EFGG G=H |
| !"#$#%&:(&+ | $ $ | .A=+&  ?6. | 5A 8 6. . / < 4 !56 0 0  6 . $0 .A=+& 7EFGG G=H |
| !"#$#%&:(&; | $ $. | .A=+&  ?6. | 457$ $. < 4 !56 0 06 . $0 .A=+& 7EFGG G=H |
| !"#$#%&:(&(!"#$#%&:(&9!"#$#%&:(&% | $ $ | .A=+&  ?6. | 5A 8 6. . / < 4 !56 0 0  6 . $0 .A=+& 7EFGG G=H |
| !"#$#%&:(&'!"#$#%&:(&:!"#$#%&:(&)!"#$#%&:(&& | $ $. | .A=+&  ?6. | 457$ $. < 4 !56 0 06 . $0 .A=+& 7EFGG G=H |
| !"#$#%&:9** | $ $ | .A=+&  ?6. | 5A 8 6. . / < 4 !56 0 0  6 . $0 .A=+& 7EFGG G=H |
| !"#$#%&:9*+!"#$#%&:9*; | $ $. | .A=+&  ?6. | 457$ $. < 4 !56 0 06 . $0 .A=+& 7EFGG G=H |
| !"#$#%&:9*9 | $ $ | .A=+&  ?6. | 5A 8 6. . / < 4 !56 0 0  6 . $0 .A=+& 7EFGG G=H |
| !"#$#%&:9*% | $ $. | .A=+&  ?6. | 457$ $. < 4 !56 0 06 . $0 .A=+& 7EFGG G=H |
| !"#$#%&:9*'!"#$#%&:9*:!"#$#%&:9*)!"#$#%&:9*&  !"#$#%&:9+*!"#$#%&:9++ | $ $ | .A=+&  ?6. | 5A 8 6. . / < 4 !56 0 0  6 . $0 .A=+& 7EFGG G=H |
| !"#$#%&:9+; | $ $. | .A=+&  ?6. | 457$ $. < 4 !56 0 06 . $0 .A=+& 7EFGG G=H |
| !"#$#%&:9+( | $ $ | .A=+&  ?6. | 5A 8 6. . / < 4 !56 0 0  6 . $0 .A=+& 7EFGG G=H |
| !"#$#%&:9+9 | $ $. | .A=+&  ?6. | 457$ $. < 4 !56 0 06 . $0 .A=+& 7EFGG G=H |
| !"#$#%&:9+%!"#$#%&:9+'!"#$#%&:9+: | $ $ | .A=+&  ?6. | 5A 8 6. . / < 4 !56 0 0  6 . $0 .A=+& 7EFGG G=H |
| !"#$#%&:9+) | $ $. | .A=+&  ?6. | 457$ $. < 4 !56 0 06 . $0 .A=+& 7EFGG G=H |
| !"#$#%&:9+&!"#$#%&:9;*!"#$#%&:9;+!"#$#%&:9;;  !"#$#%&:9;( | $ $ | .A=+&  ?6. | 5A 8 6. . / < 4 !56 0 0  6 . $0 .A=+& 7EFGG G=H |
| !"#$#%&:9;9!"#$#%&:9;%!"#$#%&:9;'!"#$#%&:9;: | $ $. | .A=+&  ?6. | 457$ $. < 4 !56 0 06 . $0 .A=+& 7EFGG G=H |
| !"#$#%&:9;&!"#$#%&:9(*!"#$#%&:9(+ | $ $ | .A=+&  ?6. | 5A 8 6. . / < 4 !56 0 0  6 . $0 .A=+& 7EFGG G=H |
| !"#$#%&:9(; | $ $. | .A=+&  ?6. | 457$ $. < 4 !56 0 06 . $0 .A=+& 7EFGG G=H |

5A 8 6. . / < 4 !56 0 06 . $0 .A=+& 7EFGG G=H

| !"#$#%&:9(% | $ $. |  | 457$ $. < 4 !56 0 06 . $0 .A=+& 7EFGG G=H |
| --- | --- | --- | --- |
| !"#$#%&:9(' | $ $ |  | 5A 8 6. . / < 4 !56 0 0  6 . $0 .A=+& 7EFGG G=H |
| !"#$#%&:9(:!"#$#%&:9() | $ $. |  | 457$ $. < 4 !56 0 06 . $0 .A=+& 7EFGG G=H |
| !"#$#%&:9(&!"#$#%&:99* | $ $ |  | 5A 8 6. . / < 4 !56 0 0  6 . $0 .A=+& 7EFGG G=H |
| !"#$#%&:99+ | $ $. |  | 457$ $. < 4 !56 0 06 . $0 .A=+& 7EFGG G=H |
| !"#$#%&:99;!"#$#%&:99(!"#$#%&:999 | $ $ |  | 5A 8 6. . / < 4 !56 0 0  6 . $0 .A=+& 7EFGG G=H |
| !"#$#%&:99%!"#$#%&:99' | $ $. |  | 457$ $. < 4 !56 0 06 . $0 .A=+& 7EFGG G=H |
| !"#$#%&:99:!"#$#%&:99) | $ $ | .A=+&  ?6. | 5A 8 6. . / < 4 !56 0 0  6 . $0 .A=+& 7EFGG G=H |
| !"#$#%&:99&!"#$#%&:9%*!"#$#%&:9%+ | $ $. | .A=+&  ?6. | 457$ $. < 4 !56 0 06 . $0 .A=+& 7EFGG G=H |
| !"#$#%&:9%; | $ $ | .A=+&  ?6. | 5A 8 6. . / < 4 !56 0 0  6 . $0 .A=+& 7EFGG G=H |
| !"#$#%&:9%( | $ $. | .A=+&  ?6. | 457$ $. < 4 !56 0 06 . $0 .A=+& 7EFGG G=H |
| !"#$#%&:9%9!"#$#%&:9%%!"#$#%&:9%'!"#$#%&:9%: | $ $ | .A=+&  ?6. | 5A 8 6. . / < 4 !56 0 0  6 . $0 .A=+& 7EFGG G=H |
| !"#$#%&:9%)!"#$#%&:9%&!"#$#%&:9'*!"#$#%&:9'+  !"#$#%&:9'; | $ $. | .A=+&  ?6. | 457$ $. < 4 !56 0 06 . $0 .A=+& 7EFGG G=H |
| !"#$#%&:9'( | $ $ | .A=+&  ?6. | 5A 8 6. . / < 4 !56 0 0  6 . $0 .A=+& 7EFGG G=H |
| !"#$#%&:9'9 | $ $. | .A=+&  ?6. | 457$ $. < 4 !56 0 06 . $0 .A=+& 7EFGG G=H |
| !"#$#%&:9'% | $ $ | .A=+&  ?6. | 5A 8 6. . / < 4 !56 0 0  6 . $0 .A=+& 7EFGG G=H |
| !"#$#%&:9''!"#$#%&:9':!"#$#%&:9')!"#$#%&:9'&  !"#$#%&:9:* | $ $. | .A=+&  ?6. | 457$ $. < 4 !56 0 06 . $0 .A=+& 7EFGG G=H |
| !"#$#%&:9:+ | $ $ | .A=+&  ?6. | 5A 8 6. . / < 4 !56 0 0  6 . $0 .A=+& 7EFGG G=H |
| !"#$#%&:9:;!"#$#%&:9:( | $ $. | .A=+&  ?6. | 457$ $. < 4 !56 0 06 . $0 .A=+& 7EFGG G=H |
| !"#$#%&:9:9!"#$#%&:9:% | $ $ | .A=+&  ?6. | 5A 8 6. . / < 4 !56 0 0  6 . $0 .A=+& 7EFGG G=H |
| !"#$#%&:9:'!"#$#%&:9::!"#$#%&:9:)!"#$#%&:9:&  !"#$#%&:9)* | $ $. | .A=+&  ?6. | 457$ $. < 4 !56 0 06 . $0 .A=+& 7EFGG G=H |
| !"#$#%&:9)+!"#$#%&:9); | $ $ | .A=+&  ?6. | 5A 8 6. . / < 4 !56 0 0  6 . $0 .A=+& 7EFGG G=H |
| !"#$#%&:9)( | $ $. | .A=+&  ?6. | 457$ $. < 4 !56 0 06 . $0 .A=+& 7EFGG G=H |
| !"#$#%&:9)9!"#$#%&:9)%!"#$#%&:9)' | $ $ | .A=+&  ?6. | 5A 8 6. . / < 4 !56 0 0  6 . $0 .A=+& 7EFGG G=H |
| !"#$#%&:9):!"#$#%&:9))!"#$#%&:9)&!"#$#%&:9&* | $ $. | .A=+&  ?6. | 457$ $. < 4 !56 0 06 . $0 .A=+& 7EFGG G=H |
| !"#$#%&:9&+!"#$#%&:9&;!"#$#%&:9&( | $ $ | .A=+&  ?6. | 5A 8 6. . / < 4 !56 0 0  6 . $0 .A=+& 7EFGG G=H |
| !"#$#%&:9&9!"#$#%&:9&% | $ $. | .A=+&  ?6. | 457$ $. < 4 !56 0 06 . $0 .A=+& 7EFGG G=H |
| !"#$#%&:9&' | $ $ | .A=+&  ?6. | 5A 8 6. . / < 4 !56 0 0  6 . $0 .A=+& 7EFGG G=H |
| !"#$#%&:9&: | $ $. | .A=+&  ?6. | 457$ $. < 4 !56 0 06 . $0 .A=+& 7EFGG G=H |
| !"#$#%&:9&)!"#$#%&:9&&!"#$#%&:%**!"#$#%&:%*+ | $ $ | .A=+&  ?6. | 5A 8 6. . / < 4 !56 0 0  6 . $0 .A=+& 7EFGG G=H |
| !"#$#%&:%*; | $ $. | .A=+&  ?6. | 457$ $. < 4 !56 0 06 . $0 .A=+& 7EFGG G=H |
| !"#$#%&:%*( | $ $ | .A=+&  ?6. | 5A 8 6. . / < 4 !56 0 0  6 . $0 .A=+& 7EFGG G=H |
| !"#$#%&:%*9!"#$#%&:%*% | $ $. | .A=+&  ?6. | 457$ $. < 4 !56 0 06 . $0 .A=+& 7EFGG G=H |
| !"#$#%&:%*' | $ $ | .A=+&  ?6. | 5A 8 6. . / < 4 !56 0 0  6 . $0 .A=+& 7EFGG G=H |
| !"#$#%&:%*:!"#$#%&:%*) | $ $. | .A=+&  ?6. | 457$ $. < 4 !56 0 06 . $0 .A=+& 7EFGG G=H |
| !"#$#%&:%*& | $ $ | .A=+&  ?6. | 5A 8 6. . / < 4 !56 0 0  6 . $0 .A=+& 7EFGG G=H |
| !"#$#%&:%+* | $ $. | .A=+&  ?6. | 457$ $. < 4 !56 0 06 . $0 .A=+& 7EFGG G=H |
| !"#$#%&:%++ | $ $ | .A=+&  ?6. | 5A 8 6. . / < 4 !56 0 0  6 . $0 .A=+& 7EFGG G=H |
| !"#$#%&:%+; | $ $. | .A=+&  ?6. | 457$ $. < 4 !56 0 06 . $0 .A=+& 7EFGG G=H |
| !"#$#%&:%+(!"#$#%&:%+9!"#$#%&:%+%!"#$#%&:%+' | $ $ | .A=+&  ?6. | 5A 8 6. . / < 4 !56 0 0  6 . $0 .A=+& 7EFGG G=H |
| !"#$#%&:%+: | $ $. | .A=+&  ?6. | 457$ $. < 4 !56 0 06 . $0 .A=+& 7EFGG G=H |
| !"#$#%&:%+) | $ $ | .A=+&  ?6. | 5A 8 6. . / < 4 !56 0 0  6 . $0 .A=+& 7EFGG G=H |
| !"#$#%&:%+&!"#$#%&:%;* | $ $. | .A=+&  ?6. | 457$ $. < 4 !56 0 06 . $0 .A=+& 7EFGG G=H |
| !"#$#%&:%;+!"#$#%&:%;; | $ $ | .A=+&  ?6. | 5A 8 6. . / < 4 !56 0 0  6 . $0 .A=+& 7EFGG G=H |

!"#$#%&:%;(!"#$#%&:%;9!"#$#%&:%;%!"#$#%&:%;'!"#$#%&:%;:!"#$#%&:%;)!"#$#%&:%;&!"#$#%&:%(*!"#$#%&:%(+!"#$#%&:%(;!"#$#%&:%((!"#$#%&:%(9!"#$#%&:%(%!"#$#%&:%('!"#$#%&:%(:!"#$#%&:%()!"#$#%&:%(&!"#$#%&:%9*!"#$#%&:%9+!"#$#%&:%9;!"#$#%&:%9(!"#$#%&:%99!"#$#%&:%9%!"#$#%&:%9'

!"#$#%&:%9:!"#$#%&:%9)!"#$#%&:%9&!"#$#%&:%%*!"#$#%&:%%+!"#$#%&:%%;!"#$#%&:%%(!"#$#%&:%%9!"#$#%&:%%%!"#$#%&:%%'!"#$#%&:%%:!"#$#%&:%%)!"#$#%&:%%&!"#$#%&:%'*!"#$#%&:%'+!"#$#%&:%';!"#$#%&:%'(!"#$#%&:%'9!"#$#%&:%'%!"#$#%&:%''!"#$#%&:%':!"#$#%&:%')!"#$#%&:%'&!"#$#%&:%:*

!"#$#%&:%:+!"#$#%&:%:;!"#$#%&:%:(!"#$#%&:%:9!"#$#%&:%:'!"#$#%&:%::!"#$#%&:%:)!"#$#%&:%:&!"#$#%&:%)*!"#$#%&:%)+!"#$#%&:%);!"#$#%&:%)(!"#$#%&:%)9!"#$#%&:%)%!"#$#%&:%)'!"#$#%&:%):!"#$#%&:%))!"#$#%&:%)&!"#$#%&:%&*!"#$#%&:%&+!"#$#%&:%&;!"#$#%&:%&(!"#$#%&:%&9!"#$#%&:%&% !"#$#%&:%&'!"#$#%&:%&:!"#$#%&:%&)!"#$#%&:%&&!"#$#%&:'**!"#$#%&:'*+!"#$#%&:'*;!"#$#%&:'*(!"#$#%&:'*9!"#$#%&:'*%!"#$#%&:'*'!"#$#%&:'*:!"#$#%&:'*)!"#$#%&:'*&!"#$#%&:'+*!"#$#%&:'++!"#$#%&:'+;!"#$#%&:'+(!"#$#%&:'+9!"#$#%&:'+%!"#$#%&:'+'!"#$#%&:'+:!"#$#%&:'+)!"#$#%&:'+&

!"#$#%&:';*!"#$#%&:';+!"#$#%&:';;!"#$#%&:';(!"#$#%&:';9!"#$#%&:';%!"#$#%&:';'!"#$#%&:';:!"#$#%&:';)!"#$#%&:';&!"#$#%&:'(*!"#$#%&:'(;!"#$#%&:'(%!"#$#%&:'('!"#$#%&:'(:!"#$#%&:'()!"#$#%&:'(&!"#$#%&:'9*!"#$#%&:'9+!"#$#%&:'9;!"#$#%&:'9(!"#$#%&:'99!"#$#%&:'9%!"#$#%&:'9'

!"#$#%&:'9:!"#$#%&:'9)!"#$#%&:'9&!"#$#%&:'%*!"#$#%&:'%+!"#$#%&:'%;!"#$#%&:'%(!"#$#%&:'%9!"#$#%&:'%%!"#$#%&:'%'!"#$#%&:'%:!"#$#%&:'%)!"#$#%&:'%&!"#$#%&:''*!"#$#%&:''+!"#$#%&:'';!"#$#%&:''(!"#$#%&:''9!"#$#%&:''%!"#$#%&:'''!"#$#%&:'':!"#$#%&:'')!"#$#%&:''&!"#$#%&:':*

!"#$#%&:':+!"#$#%&:':;!"#$#%&:':(!"#$#%&:':9!"#$#%&:':%!"#$#%&:':'!"#$#%&:'::!"#$#%&:':)!"#$#%&:':&!"#$#%&:')*!"#$#%&:')+!"#$#%&:');!"#$#%&:')(!"#$#%&:')9!"#$#%&:')%!"#$#%&:')'!"#$#%&:'):!"#$#%&:'))!"#$#%&:')&!"#$#%&:'&*!"#$#%&:'&+!"#$#%&:'&;!"#$#%&:'&(!"#$#%&:'&9

!"#$#%&:'&%!"#$#%&:'&'!"#$#%&:'&:!"#$#%&:'&)!"#$#%&:'&&!"#$#%&::**!"#$#%&::*+!"#$#%&::*;!"#$#%&::*(!"#$#%&::*9!"#$#%&::*%!"#$#%&::*'!"#$#%&::*:!"#$#%&::*)!"#$#%&::*&!"#$#%&::+*!"#$#%&::++!"#$#%&::+;!"#$#%&::+(!"#$#%&::+9!"#$#%&::+%!"#$#%&::+'!"#$#%&::+:!"#$#%&::+)

!"#$#%&::+&!"#$#%&::;*!"#$#%&::;+!"#$#%&::;;!"#$#%&::;(!"#$#%&::;9!"#$#%&::;%!"#$#%&::;'!"#$#%&::;:!"#$#%&::;)!"#$#%&::;&!"#$#%&::(*!"#$#%&::(+!"#$#%&::(;!"#$#%&::((!"#$#%&::(9!"#$#%&::(%!"#$#%&::('!"#$#%&::(:!"#$#%&::()!"#$#%&::(&!"#$#%&::9*!"#$#%&::9+!"#$#%&::9;

!"#$#%&::9(!"#$#%&::99!"#$#%&::9%!"#$#%&::9'!"#$#%&::9:!"#$#%&::9)!"#$#%&::9&!"#$#%&::%*!"#$#%&::%+!"#$#%&::%;!"#$#%&::%(!"#$#%&::%9!"#$#%&::%%!"#$#%&::%'!"#$#%&::%:!"#$#%&::%)!"#$#%&::%&!"#$#%&::'*!"#$#%&::'+!"#$#%&::';!"#$#%&::'(!"#$#%&::'9!"#$#%&::'%!"#$#%&::''

!"#$#%&::':!"#$#%&::')!"#$#%&::'&!"#$#%&:::*!"#$#%&:::+!"#$#%&:::;!"#$#%&:::(!"#$#%&:::9!"#$#%&:::%!"#$#%&:::'!"#$#%&::::!"#$#%&:::)!"#$#%&:::&!"#$#%&::)*!"#$#%&::)+!"#$#%&::);!"#$#%&::)(!"#$#%&::)9!"#$#%&::)%!"#$#%&::)'!"#$#%&::):!"#$#%&::))!"#$#%&::)&!"#$#%&::&*

!"#$#%&::&+!"#$#%&::&;!"#$#%&::&(!"#$#%&::&9!"#$#%&::&%!"#$#%&::&'!"#$#%&::&:!"#$#%&::&)!"#$#%&::&&!"#$#%&:)**!"#$#%&:)*+!"#$#%&:)*;!"#$#%&:)*(!"#$#%&:)*9!"#$#%&:)*%!"#$#%&:)*'!"#$#%&:)*:!"#$#%&:)*)!"#$#%&:)*&!"#$#%&:)+*!"#$#%&:)++!"#$#%&:)+;!"#$#%&:)+(!"#$#%&:)+9

!"#$#%&:)+%!"#$#%&:)+'!"#$#%&:)+:!"#$#%&:)+)!"#$#%&:)+&!"#$#%&:);*!"#$#%&:);+!"#$#%&:);;!"#$#%&:);(!"#$#%&:);9!"#$#%&:);%!"#$#%&:);'!"#$#%&:);:!"#$#%&:);)!"#$#%&:);&!"#$#%&:)(*!"#$#%&:)(+!"#$#%&:)(;!"#$#%&:)((!"#$#%&:)(9!"#$#%&:)(%!"#$#%&:)('!"#$#%&:)(:!"#$#%&:)() !"#$#%&:)(&!"#$#%&:)9*!"#$#%&:)9+!"#$#%&:)9;!"#$#%&:)9(!"#$#%&:)99!"#$#%&:)9%!"#$#%&:)9'!"#$#%&:)9:!"#$#%&:)9)!"#$#%&:)9&!"#$#%&:)%*!"#$#%&:)%+!"#$#%&:)%;!"#$#%&:)%(!"#$#%&:)%9!"#$#%&:)%%!"#$#%&:)%'!"#$#%&:)%:!"#$#%&:)%)!"#$#%&:)%&!"#$#%&:)'*!"#$#%&:)'+!"#$#%&:)'; !"#$#%&:)'(!"#$#%&:)'9!"#$#%&:)'%!"#$#%&:)''!"#$#%&:)':!"#$#%&:)')!"#$#%&:)'&!"#$#%&:):*!"#$#%&:):+!"#$#%&:):;!"#$#%&:):(!"#$#%&:):9!"#$#%&:):%!"#$#%&:):'!"#$#%&:)::!"#$#%&:):)!"#$#%&:):&!"#$#%&:))*!"#$#%&:))+!"#$#%&:));!"#$#%&:))(!"#$#%&:))9!"#$#%&:))%!"#$#%&:))'

!"#$#%&:)):!"#$#%&:)))!"#$#%&:))&!"#$#%&:)&*!"#$#%&:)&+!"#$#%&:)&;!"#$#%&:)&(!"#$#%&:)&9!"#$#%&:)&%!"#$#%&:)&'!"#$#%&:)&:!"#$#%&:)&)!"#$#%&:)&&!"#$#%&:&**!"#$#%&:&*+!"#$#%&:&*;!"#$#%&:&*(!"#$#%&:&*9!"#$#%&:&*%!"#$#%&:&*'!"#$#%&:&*:!"#$#%&:&*)!"#$#%&:&*&!"#$#%&:&+*

!"#$#%&:&++!"#$#%&:&+;!"#$#%&:&+(!"#$#%&:&+9!"#$#%&:&+%!"#$#%&:&+'!"#$#%&:&+:!"#$#%&:&+)!"#$#%&:&+&!"#$#%&:&;*!"#$#%&:&;+!"#$#%&:&;;!"#$#%&:&;(!"#$#%&:&;9!"#$#%&:&;%!"#$#%&:&;'!"#$#%&:&;:!"#$#%&:&;)!"#$#%&:&;&!"#$#%&:&(*!"#$#%&:&(+!"#$#%&:&(;!"#$#%&:&((!"#$#%&:&(9

!"#$#%&:&(%!"#$#%&:&('!"#$#%&:&(:!"#$#%&:&()!"#$#%&:&(&!"#$#%&:&9*!"#$#%&:&9+!"#$#%&:&9;!"#$#%&:&9(!"#$#%&:&99!"#$#%&:&9%!"#$#%&:&9'!"#$#%&:&9:!"#$#%&:&9)!"#$#%&:&9&!"#$#%&:&%*!"#$#%&:&%+!"#$#%&:&%;!"#$#%&:&%(!"#$#%&:&%9!"#$#%&:&%%!"#$#%&:&%'!"#$#%&:&%:!"#$#%&:&%)

!"#$#%&:&%&!"#$#%&:&'*!"#$#%&:&'+!"#$#%&:&';!"#$#%&:&'(!"#$#%&:&'9!"#$#%&:&'%!"#$#%&:&''!"#$#%&:&':!"#$#%&:&')!"#$#%&:&'&!"#$#%&:&:*!"#$#%&:&:+!"#$#%&:&:;!"#$#%&:&:(!"#$#%&:&:9!"#$#%&:&:%!"#$#%&:&:'!"#$#%&:&::!"#$#%&:&:)!"#$#%&:&:&!"#$#%&:&)*!"#$#%&:&)+!"#$#%&:&);

!"#$#%&:&)(!"#$#%&:&)%!"#$#%&:&)'!"#$#%&:&):!"#$#%&:&))!"#$#%&:&)&!"#$#%&:&&*!"#$#%&:&&+!"#$#%&:&&;!"#$#%&:&&(!"#$#%&:&&9!"#$#%&:&&%!"#$#%&:&&'!"#$#%&:&&:!"#$#%&:&&)!"#$#%&:&&&!"#$#%&)***!"#$#%&)**;!"#$#%&)**(!"#$#%&)**9!"#$#%&)**%!"#$#%&)**'!"#$#%&)**:!"#$#%&)**)

!"#$#%&)**&!"#$#%&)*+*!"#$#%&)*++!"#$#%&)*+;!"#$#%&)*+(!"#$#%&)*+9!"#$#%&)*+%!"#$#%&)*+'!"#$#%&)*+:!"#$#%&)*+)!"#$#%&)*+&!"#$#%&)*;*!"#$#%&)*;+!"#$#%&)*;;!"#$#%&)*;(!"#$#%&)*;9!"#$#%&)*;%!"#$#%&)*;'!"#$#%&)*;:!"#$#%&)*;)!"#$#%&)*;&!"#$#%&)*(*!"#$#%&)*(+!"#$#%&)*(; !"#$#%&)*((!"#$#%&)*(9!"#$#%&)*(%!"#$#%&)*('!"#$#%&)*(:!"#$#%&)*()!"#$#%&)*(&!"#$#%&)*9*!"#$#%&)*9+!"#$#%&)*9;!"#$#%&)*9(!"#$#%&)*99!"#$#%&)*9%!"#$#%&)*9'!"#$#%&)*9:!"#$#%&)*9)!"#$#%&)*9&!"#$#%&)*%*!"#$#%&)*%+!"#$#%&)*%;!"#$#%&)*%(!"#$#%&)*%9!"#$#%&)*%%!"#$#%&)*%'

!"#$#%&)*%:!"#$#%&)*%)!"#$#%&)*%&!"#$#%&)*'*!"#$#%&)*'+!"#$#%&)*';!"#$#%&)*'(!"#$#%&)*'9!"#$#%&)*'%!"#$#%&)*''!"#$#%&)*':!"#$#%&)*')!"#$#%&)*'&!"#$#%&)*:*!"#$#%&)*:+!"#$#%&)*:;!"#$#%&)*:(!"#$#%&)*:9!"#$#%&)*:%!"#$#%&)*:'!"#$#%&)*::!"#$#%&)*:)!"#$#%&)*:&!"#$#%&)*)*

!"#$#%&)*)+!"#$#%&)*);!"#$#%&)*)(!"#$#%&)*)9!"#$#%&)*)%!"#$#%&)*)'!"#$#%&)*):!"#$#%&)*))!"#$#%&)*)&!"#$#%&)*&*!"#$#%&)*&+!"#$#%&)*&;!"#$#%&)*&(!"#$#%&)*&9!"#$#%&)*&%!"#$#%&)*&'!"#$#%&)*&:!"#$#%&)*&)!"#$#%&)*&&!"#$#%&)+**!"#$#%&)+*+!"#$#%&)+*;!"#$#%&)+*(!"#$#%&)+*9

!"#$#%&)+*%!"#$#%&)+*'!"#$#%&)+*:!"#$#%&)+*)!"#$#%&)+*&!"#$#%&)++*!"#$#%&)+++!"#$#%&)++;!"#$#%&)++(!"#$#%&)++9!"#$#%&)++%!"#$#%&)++'!"#$#%&)++:!"#$#%&)++)!"#$#%&)++&!"#$#%&)+;*!"#$#%&)+;+!"#$#%&)+;;!"#$#%&)+;(!"#$#%&)+;9!"#$#%&)+;%!"#$#%&)+;'!"#$#%&)+;:!"#$#%&)+;) !"#$#%&)+;&!"#$#%&)+(*!"#$#%&)+(+!"#$#%&)+(;!"#$#%&)+((!"#$#%&)+(9!"#$#%&)+(%!"#$#%&)+('!"#$#%&)+(:!"#$#%&)+()!"#$#%&)+(&!"#$#%&)+9*!"#$#%&)+9+!"#$#%&)+9;!"#$#%&)+9(!"#$#%&)+99!"#$#%&)+9'!"#$#%&)+9:!"#$#%&)+9)!"#$#%&)+9&!"#$#%&)+%*!"#$#%&)+%+!"#$#%&)+%;!"#$#%&)+%( !"#$#%&)+%9!"#$#%&)+%%!"#$#%&)+%'!"#$#%&)+%:!"#$#%&)+%)!"#$#%&)+%&!"#$#%&)+'*!"#$#%&)+'+!"#$#%&)+';!"#$#%&)+'9!"#$#%&)+'%!"#$#%&)+''!"#$#%&)+':!"#$#%&)+')!"#$#%&)+'&!"#$#%&)+:*!"#$#%&)+:+!"#$#%&)+:;!"#$#%&)+:(!"#$#%&)+:9!"#$#%&)+:%!"#$#%&)+:'!"#$#%&)+::!"#$#%&)+:) !"#$#%&)+:&!"#$#%&)+)*!"#$#%&)+)+!"#$#%&)+);!"#$#%&)+)(!"#$#%&)+)9!"#$#%&)+)%!"#$#%&)+)'!"#$#%&)+):!"#$#%&)+))!"#$#%&)+)&!"#$#%&)+&*!"#$#%&)+&+!"#$#%&)+&;!"#$#%&)+&(!"#$#%&)+&9!"#$#%&)+&%!"#$#%&)+&'!"#$#%&)+&:!"#$#%&)+&)!"#$#%&)+&&!"#$#%&);**!"#$#%&);*+!"#$#%&);*;

!"#$#%&);*(!"#$#%&);*9!"#$#%&);*%!"#$#%&);*'!"#$#%&);*:!"#$#%&);*)!"#$#%&);*&!"#$#%&);+*!"#$#%&);++!"#$#%&);+;!"#$#%&);+(!"#$#%&);+9!"#$#%&);+%!"#$#%&);+'!"#$#%&);+:!"#$#%&);+)!"#$#%&);+&!"#$#%&);;*!"#$#%&);;+!"#$#%&);;;!"#$#%&);;(!"#$#%&);;9!"#$#%&);;%!"#$#%&);;' !"#$#%&);;:!"#$#%&);;)!"#$#%&);;&!"#$#%&);(*!"#$#%&);(+!"#$#%&);(;!"#$#%&);((!"#$#%&);(9!"#$#%&);(%!"#$#%&);('!"#$#%&);(:!"#$#%&);()!"#$#%&);(&!"#$#%&);9*!"#$#%&);9+!"#$#%&);9;!"#$#%&);9(!"#$#%&);99!"#$#%&);9%!"#$#%&);9'!"#$#%&);9:!"#$#%&);9)

$ $ " .A=+& 0B 35 7$ $ " < 4 !56 0 06 .

?6. $0 .A=+& 7EFGG G=H

!"#$#%&);%*!"#$#%&);%+!"#$#%&);%;!"#$#%&);%(!"#$#%&);%9!"#$#%&);%%!"#$#%&);%'!"#$#%&);%:!"#$#%&);%)!"#$#%&);%&!"#$#%&);'*!"#$#%&);'+!"#$#%&);';!"#$#%&);'(!"#$#%&);'9!"#$#%&);'%!"#$#%&);''!"#$#%&);':!"#$#%&);')!"#$#%&);'&!"#$#%&);:*!"#$#%&);:+!"#$#%&);:;!"#$#%&);:( !"#$#%&);:9!"#$#%&);:%!"#$#%&);:'!"#$#%&);::!"#$#%&);:)!"#$#%&);:&!"#$#%&);)*!"#$#%&);)+!"#$#%&););!"#$#%&);)(!"#$#%&);)9!"#$#%&);)%!"#$#%&);)'!"#$#%&);):!"#$#%&);))!"#$#%&);)&!"#$#%&);&*!"#$#%&);&+!"#$#%&);&;!"#$#%&);&(!"#$#%&);&9!"#$#%&);&%!"#$#%&);&'!"#$#%&);&:

!"#$#%&);&)!"#$#%&);&&!"#$#%&)(**!"#$#%&)(*+!"#$#%&)(*;!"#$#%&)(*(!"#$#%&)(*9!"#$#%&)(*'!"#$#%&)(*:!"#$#%&)(*)!"#$#%&)(*&!"#$#%&)(+*!"#$#%&)(++!"#$#%&)(+;!"#$#%&)(+(!"#$#%&)(+9!"#$#%&)(+%!"#$#%&)(+'!"#$#%&)(+:!"#$#%&)(+)!"#$#%&)(+&!"#$#%&)(;*!"#$#%&)(;+!"#$#%&)(;;

!"#$#%&)(;(!"#$#%&)(;9!"#$#%&)(;%!"#$#%&)(;'!"#$#%&)(;:!"#$#%&)(;)!"#$#%&)(;&!"#$#%&)((;!"#$#%&)(((!"#$#%&)((9!"#$#%&)((%!"#$#%&)(('!"#$#%&)((:!"#$#%&)(()!"#$#%&)((&!"#$#%&)(9*!"#$#%&)(9+!"#$#%&)(9;!"#$#%&)(9(!"#$#%&)(99!"#$#%&)(9%!"#$#%&)(9'!"#$#%&)(9:!"#$#%&)(9)

!"#$#%&)(9&!"#$#%&)(%*!"#$#%&)(%+!"#$#%&)(%;!"#$#%&)(%(!"#$#%&)(%9!"#$#%&)(%%!"#$#%&)(%'!"#$#%&)(%:!"#$#%&)(%)!"#$#%&)(%&!"#$#%&)('*!"#$#%&)(';!"#$#%&)('(!"#$#%&)('9!"#$#%&)('%!"#$#%&)(''!"#$#%&)(':!"#$#%&)(')!"#$#%&)('&!"#$#%&)(:*!"#$#%&)(:+!"#$#%&)(:;!"#$#%&)(:(

!"#$#%&)(:9!"#$#%&)(:%!"#$#%&)(:'!"#$#%&)(::!"#$#%&)(:)!"#$#%&)(:&!"#$#%&)()*!"#$#%&)()+!"#$#%&)();!"#$#%&)()(!"#$#%&)()9!"#$#%&)()%!"#$#%&)()'!"#$#%&)():!"#$#%&)())!"#$#%&)()&!"#$#%&)(&*!"#$#%&)(&+!"#$#%&)(&;!"#$#%&)(&(!"#$#%&)(&9!"#$#%&)(&%!"#$#%&)(&'!"#$#%&)(&:

!"#$#%&)(&)!"#$#%&)(&&!"#$#%&)9**!"#$#%&)9*+!"#$#%&)9*;!"#$#%&)9*(!"#$#%&)9*9!"#$#%&)9*%!"#$#%&)9*'!"#$#%&)9*:!"#$#%&)9*&!"#$#%&)9+*!"#$#%&)9++!"#$#%&)9+;!"#$#%&)9+(!"#$#%&)9+9!"#$#%&)9+%!"#$#%&)9+'!"#$#%&)9+:!"#$#%&)9+)!"#$#%&)9+&!"#$#%&)9;*!"#$#%&)9;+!"#$#%&)9;;

!"#$#%&)9;(!"#$#%&)9;9!"#$#%&)9;%!"#$#%&)9;'!"#$#%&)9;:!"#$#%&)9;)!"#$#%&)9;&!"#$#%&)9(*!"#$#%&)9(+!"#$#%&)9(;!"#$#%&)9(9!"#$#%&)9(%!"#$#%&)9(:!"#$#%&)9()!"#$#%&)9(&!"#$#%&)99*!"#$#%&)99+!"#$#%&)99;!"#$#%&)99(!"#$#%&)999!"#$#%&)99%!"#$#%&)99'!"#$#%&)99:!"#$#%&)99)

!"#$#%&)99&!"#$#%&)9%*!"#$#%&)9%+!"#$#%&)9%;!"#$#%&)9%(!"#$#%&)9%9!"#$#%&)9%%!"#$#%&)9%'!"#$#%&)9%:!"#$#%&)9%)!"#$#%&)9%&!"#$#%&)9'*!"#$#%&)9'+!"#$#%&)9';!"#$#%&)9'(!"#$#%&)9'9!"#$#%&)9'%!"#$#%&)9''!"#$#%&)9':!"#$#%&)9')!"#$#%&)9'&!"#$#%&)9:*!"#$#%&)9:+!"#$#%&)9:;

!"#$#%&)9:(!"#$#%&)9:9!"#$#%&)9:%!"#$#%&)9:'!"#$#%&)9::!"#$#%&)9:)!"#$#%&)9:&!"#$#%&)9)*!"#$#%&)9)+!"#$#%&)9);!"#$#%&)9)(!"#$#%&)9)9!"#$#%&)9)%!"#$#%&)9)'!"#$#%&)9):!"#$#%&)9))!"#$#%&)9&*!"#$#%&)9&+!"#$#%&)9&;!"#$#%&)9&(!"#$#%&)9&9!"#$#%&)9&%!"#$#%&)9&'!"#$#%&)9&:

!"#$#%&)9&)!"#$#%&)9&&!"#$#%&)%**!"#$#%&)%*+!"#$#%&)%*;!"#$#%&)%*(!"#$#%&)%*9!"#$#%&)%*%!"#$#%&)%*'!"#$#%&)%*:!"#$#%&)%*)!"#$#%&)%*&!"#$#%&)%+*!"#$#%&)%++!"#$#%&)%+;!"#$#%&)%+(!"#$#%&)%+%!"#$#%&)%+'!"#$#%&)%+:!"#$#%&)%+)!"#$#%&)%+&!"#$#%&)%;*!"#$#%&)%;+!"#$#%&)%;; !"#$#%&)%;(!"#$#%&)%;9!"#$#%&)%;%!"#$#%&)%;'!"#$#%&)%;:!"#$#%&)%;)!"#$#%&)%;&!"#$#%&)%(*!"#$#%&)%(+!"#$#%&)%(;!"#$#%&)%((!"#$#%&)%(9!"#$#%&)%(%!"#$#%&)%('!"#$#%&)%(:!"#$#%&)%()!"#$#%&)%(&!"#$#%&)%9*!"#$#%&)%9+!"#$#%&)%9;!"#$#%&)%9(!"#$#%&)%99!"#$#%&)%9'!"#$#%&)%9: !"#$#%&)%9)!"#$#%&)%9&!"#$#%&)%%*!"#$#%&)%%+!"#$#%&)%%;!"#$#%&)%%(!"#$#%&)%%9!"#$#%&)%%%!"#$#%&)%%'!"#$#%&)%%:!"#$#%&)%%)!"#$#%&)%%&!"#$#%&)%'*!"#$#%&)%'+!"#$#%&)%';!"#$#%&)%'(!"#$#%&)%'%!"#$#%&)%''!"#$#%&)%':!"#$#%&)%')!"#$#%&)%'&!"#$#%&)%:*!"#$#%&)%:+!"#$#%&)%:;

!"#$#%&)%:(!"#$#%&)%:9!"#$#%&)%:%!"#$#%&)%:'!"#$#%&)%::!"#$#%&)%:)!"#$#%&)%:&!"#$#%&)%)*!"#$#%&)%)+!"#$#%&)%);!"#$#%&)%)(!"#$#%&)%)9!"#$#%&)%)%!"#$#%&)%)'!"#$#%&)%):!"#$#%&)%))!"#$#%&)%)&!"#$#%&)%&*!"#$#%&)%&+!"#$#%&)%&;!"#$#%&)%&(!"#$#%&)%&9!"#$#%&)%&%!"#$#%&)%&'

!"#$#%&)%&:!"#$#%&)%&)!"#$#%&)%&&!"#$#%&)'**!"#$#%&)'*+!"#$#%&)'*;!"#$#%&)'*(!"#$#%&)'*9!"#$#%&)'*%!"#$#%&)'*:!"#$#%&)'*)!"#$#%&)'*&!"#$#%&)'+*!"#$#%&)'++!"#$#%&)'+;!"#$#%&)'+(!"#$#%&)'+9!"#$#%&)'+%!"#$#%&)'+'!"#$#%&)'+:!"#$#%&)'+)!"#$#%&)'+&!"#$#%&)';*!"#$#%&)';+

!"#$#%&)';;!"#$#%&)';(!"#$#%&)';9!"#$#%&)';%!"#$#%&)';'!"#$#%&)';:!"#$#%&)';)!"#$#%&)';&!"#$#%&)'(*!"#$#%&)'(+!"#$#%&)'(;!"#$#%&)'((!"#$#%&)'(9!"#$#%&)'(%!"#$#%&)'('!"#$#%&)'(:!"#$#%&)'(&!"#$#%&)'9*!"#$#%&)'9+!"#$#%&)'9;!"#$#%&)'9(!"#$#%&)'99!"#$#%&)'9%!"#$#%&)'9'

!"#$#%&)'9:!"#$#%&)'9)!"#$#%&)'9&!"#$#%&)'%*!"#$#%&)'%+!"#$#%&)'%;!"#$#%&)'%(!"#$#%&)'%9!"#$#%&)'%%!"#$#%&)'%'!"#$#%&)'%:!"#$#%&)'%)!"#$#%&)'%&!"#$#%&)''*!"#$#%&)''+!"#$#%&)'';!"#$#%&)''(!"#$#%&)''9!"#$#%&)''%!"#$#%&)'''!"#$#%&)'')!"#$#%&)''&!"#$#%&)':*!"#$#%&)':+

!"#$#%&)':;!"#$#%&)':(!"#$#%&)':9!"#$#%&)':%!"#$#%&)':'!"#$#%&)'::!"#$#%&)':)!"#$#%&)':&!"#$#%&)')*!"#$#%&)')+!"#$#%&)');!"#$#%&)')(!"#$#%&)')9!"#$#%&)')%!"#$#%&)')'!"#$#%&)'):!"#$#%&)'))!"#$#%&)')&!"#$#%&)'&*!"#$#%&)'&+!"#$#%&)'&;!"#$#%&)'&(!"#$#%&)'&9!"#$#%&)'&%

!"#$#%&)'&'!"#$#%&)'&:!"#$#%&)'&)!"#$#%&)'&&!"#$#%&):**!"#$#%&):*+!"#$#%&):*;!"#$#%&):*(!"#$#%&):*9!"#$#%&):*%!"#$#%&):*'!"#$#%&):*:!"#$#%&):*)!"#$#%&):*&!"#$#%&):+*!"#$#%&):++!"#$#%&):+;!"#$#%&):+(!"#$#%&):+9!"#$#%&):+%!"#$#%&):+'!"#$#%&):+:!"#$#%&):+)!"#$#%&):+& !"#$#%&):;*!"#$#%&):;+!"#$#%&):;;!"#$#%&):;(!"#$#%&):;9!"#$#%&):;%!"#$#%&):;'!"#$#%&):;:!"#$#%&):;)!"#$#%&):;&!"#$#%&):(*!"#$#%&):(+!"#$#%&):(;!"#$#%&):((!"#$#%&):(9!"#$#%&):(%!"#$#%&):('!"#$#%&):(:!"#$#%&):()!"#$#%&):(&!"#$#%&):9*!"#$#%&):9+!"#$#%&):9;!"#$#%&):9(

!"#$#%&):99!"#$#%&):9%!"#$#%&):9:!"#$#%&):9)!"#$#%&):9&!"#$#%&):%*!"#$#%&):%+!"#$#%&):%;!"#$#%&):%(!"#$#%&):%9!"#$#%&):%%!"#$#%&):%'!"#$#%&):%:!"#$#%&):%)!"#$#%&):%&!"#$#%&):'*!"#$#%&):'+!"#$#%&):';!"#$#%&):'(!"#$#%&):'9!"#$#%&):''!"#$#%&):':!"#$#%&):')!"#$#%&):'&

!"#$#%&)::*!"#$#%&)::+!"#$#%&)::;!"#$#%&)::(!"#$#%&)::9!"#$#%&)::%!"#$#%&)::'!"#$#%&):::!"#$#%&)::)!"#$#%&)::&!"#$#%&):)*!"#$#%&):)+!"#$#%&):);!"#$#%&):)(!"#$#%&):)9!"#$#%&):)%!"#$#%&):)'!"#$#%&):):!"#$#%&):))!"#$#%&):)&!"#$#%&):&*!"#$#%&):&+!"#$#%&):&;!"#$#%&):&(

!"#$#%&):&9!"#$#%&):&%!"#$#%&):&'!"#$#%&):&:!"#$#%&):&)!"#$#%&):&&!"#$#%&))**!"#$#%&))*+!"#$#%&))*;!"#$#%&))*(!"#$#%&))*9!"#$#%&))*%!"#$#%&))*'!"#$#%&))*:!"#$#%&))*)!"#$#%&))*&!"#$#%&))+*!"#$#%&))++!"#$#%&))+;!"#$#%&))+(!"#$#%&))+9!"#$#%&))+%!"#$#%&))+'!"#$#%&))+)

!"#$#%&))+&!"#$#%&));*!"#$#%&));;!"#$#%&));(

|  | $ $3 6 | .A=+&  ?6. | 7$ $3 6 < 4 !56 0 06 . $0 .A=+& 7EFGG G=H |
| --- | --- | --- | --- |
| !"#$#%&));9!"#$#%&));%!"#$#%&));'!"#$#%&));: !"#$#%&));)!"#$#%&));&!"#$#%&))(* | $ $ " | .A=+&  ?6. | 0B 35 7$ $ " < 4 !56 0 06 .  $0 .A=+& 7EFGG G=H |

!"#$#%&))(+!"#$#%&))(;!"#$#%&))((!"#$#%&))(9!"#$#%&))(%!"#$#%&))('!"#$#%&))(:!"#$#%&))()!"#$#%&))(&!"#$#%&))9*!"#$#%&))9+!"#$#%&))9;!"#$#%&))9(!"#$#%&))99!"#$#%&))9%!"#$#%&))9'!"#$#%&))9:!"#$#%&))9)!"#$#%&))9&!"#$#%&))%*!"#$#%&))%+!"#$#%&))%;!"#$#%&))%(!"#$#%&))%9 !"#$#%&))%%!"#$#%&))%'!"#$#%&))%:!"#$#%&))%)!"#$#%&))%&!"#$#%&))'*!"#$#%&))'+!"#$#%&))';!"#$#%&))'(!"#$#%&))'9!"#$#%&))'%!"#$#%&))''!"#$#%&))':!"#$#%&))')!"#$#%&))'&!"#$#%&)):*!"#$#%&)):+!"#$#%&)):;!"#$#%&)):(!"#$#%&)):9!"#$#%&)):%!"#$#%&)):'!"#$#%&))::!"#$#%&)):)

!"#$#%&)):&!"#$#%&)))*!"#$#%&)))+!"#$#%&)));!"#$#%&)))(!"#$#%&)))9!"#$#%&)))%!"#$#%&)))'!"#$#%&))):!"#$#%&))))!"#$#%&)))&!"#$#%&))&*!"#$#%&))&+!"#$#%&))&;!"#$#%&))&(!"#$#%&))&9!"#$#%&))&%!"#$#%&))&'!"#$#%&))&:!"#$#%&))&)!"#$#%&))&&!"#$#%&)&**!"#$#%&)&*+!"#$#%&)&*;

!"#$#%&)&*(!"#$#%&)&*9!"#$#%&)&*%!"#$#%&)&*'!"#$#%&)&*:!"#$#%&)&*)!"#$#%&)&*&!"#$#%&)&+*!"#$#%&)&++!"#$#%&)&+;!"#$#%&)&+(!"#$#%&)&+9!"#$#%&)&+%!"#$#%&)&+'!"#$#%&)&+:!"#$#%&)&+)!"#$#%&)&+&!"#$#%&)&;*!"#$#%&)&;+!"#$#%&)&;;!"#$#%&)&;(!"#$#%&)&;9!"#$#%&)&;%!"#$#%&)&;'

!"#$#%&)&;:!"#$#%&)&;)!"#$#%&)&;&!"#$#%&)&(*!"#$#%&)&(+!"#$#%&)&(;!"#$#%&)&((!"#$#%&)&(9!"#$#%&)&(%!"#$#%&)&('!"#$#%&)&(:!"#$#%&)&()!"#$#%&)&(&!"#$#%&)&9*!"#$#%&)&9+!"#$#%&)&9;!"#$#%&)&9(!"#$#%&)&99!"#$#%&)&9%!"#$#%&)&9'!"#$#%&)&9:!"#$#%&)&9)!"#$#%&)&9&!"#$#%&)&%*

!"#$#%&)&%+!"#$#%&)&%;!"#$#%&)&%(!"#$#%&)&%9!"#$#%&)&%%!"#$#%&)&%'!"#$#%&)&%:!"#$#%&)&%)!"#$#%&)&%&!"#$#%&)&'*!"#$#%&)&'+!"#$#%&)&';!"#$#%&)&'(!"#$#%&)&'9!"#$#%&)&'%!"#$#%&)&''!"#$#%&)&':!"#$#%&)&')!"#$#%&)&'&!"#$#%&)&:+!"#$#%&)&:;!"#$#%&)&:(!"#$#%&)&:9!"#$#%&)&:% !"#$#%&)&:'!"#$#%&)&::!"#$#%&)&:)!"#$#%&)&:&!"#$#%&)&)*!"#$#%&)&)+!"#$#%&)&);!"#$#%&)&)(!"#$#%&)&)9!"#$#%&)&)%!"#$#%&)&)'!"#$#%&)&):!"#$#%&)&))!"#$#%&)&)&!"#$#%&)&&*!"#$#%&)&&+!"#$#%&)&&;!"#$#%&)&&(!"#$#%&)&&9!"#$#%&)&&%!"#$#%&)&&'!"#$#%&)&&:!"#$#%&)&&)!"#$#%&)&&& !"#$#%&&***!"#$#%&&**+!"#$#%&&**;!"#$#%&&**(!"#$#%&&**9!"#$#%&&**%!"#$#%&&**'!"#$#%&&**:!"#$#%&&**)!"#$#%&&**&!"#$#%&&*++!"#$#%&&*+;!"#$#%&&*+(!"#$#%&&*+9!"#$#%&&*+%!"#$#%&&*+'!"#$#%&&*+:!"#$#%&&*+)!"#$#%&&*+&!"#$#%&&*;*!"#$#%&&*;+!"#$#%&&*;;!"#$#%&&*;(!"#$#%&&*;9

!"#$#%&&*;%!"#$#%&&*;'!"#$#%&&*;:!"#$#%&&*;)!"#$#%&&*;&!"#$#%&&*(*!"#$#%&&*(+!"#$#%&&*(;!"#$#%&&*((!"#$#%&&*(9!"#$#%&&*(%!"#$#%&&*('!"#$#%&&*(:!"#$#%&&*()!"#$#%&&*(&!"#$#%&&*9*!"#$#%&&*9+!"#$#%&&*9;!"#$#%&&*9(!"#$#%&&*99!"#$#%&&*9%!"#$#%&&*9'!"#$#%&&*9:!"#$#%&&*9)

!"#$#%&&*9&!"#$#%&&*%*!"#$#%&&*%+!"#$#%&&*%;!"#$#%&&*%(!"#$#%&&*%9!"#$#%&&*%%!"#$#%&&*%'!"#$#%&&*%:!"#$#%&&*%)!"#$#%&&*%&!"#$#%&&*'*!"#$#%&&*'+!"#$#%&&*';!"#$#%&&*'(!"#$#%&&*'9!"#$#%&&*'%!"#$#%&&*''!"#$#%&&*':!"#$#%&&*')!"#$#%&&*'&!"#$#%&&*:*!"#$#%&&*:+!"#$#%&&*:;

!"#$#%&&*:(!"#$#%&&*:9!"#$#%&&*:%!"#$#%&&*:'!"#$#%&&*:)!"#$#%&&*:&!"#$#%&&*)*!"#$#%&&*)+!"#$#%&&*);!"#$#%&&*)(!"#$#%&&*)9!"#$#%&&*)%!"#$#%&&*)'!"#$#%&&*):!"#$#%&&*))!"#$#%&&*)&!"#$#%&&*&*!"#$#%&&*&+!"#$#%&&*&;!"#$#%&&*&(!"#$#%&&*&9!"#$#%&&*&%!"#$#%&&*&'!"#$#%&&*&:

!"#$#%&&*&)!"#$#%&&*&&!"#$#%&&+**!"#$#%&&+*+!"#$#%&&+*;!"#$#%&&+*(!"#$#%&&+*9!"#$#%&&+*%!"#$#%&&+*'!"#$#%&&+*:!"#$#%&&+*)!"#$#%&&+*&!"#$#%&&++*!"#$#%&&+++!"#$#%&&++;!"#$#%&&++(!"#$#%&&++9!"#$#%&&++%!"#$#%&&++'!"#$#%&&++:!"#$#%&&++)!"#$#%&&++&!"#$#%&&+;*!"#$#%&&+;+

!"#$#%&&+;;!"#$#%&&+;(!"#$#%&&+;9!"#$#%&&+;%!"#$#%&&+;'!"#$#%&&+;:!"#$#%&&+;)!"#$#%&&+;&!"#$#%&&+(*!"#$#%&&+(+!"#$#%&&+(;!"#$#%&&+((!"#$#%&&+(9!"#$#%&&+(%!"#$#%&&+('!"#$#%&&+(:!"#$#%&&+()!"#$#%&&+(&!"#$#%&&+9*!"#$#%&&+9+!"#$#%&&+9;!"#$#%&&+9(!"#$#%&&+99!"#$#%&&+9%

!"#$#%&&+9'!"#$#%&&+9:!"#$#%&&+9)!"#$#%&&+9&!"#$#%&&+%*!"#$#%&&+%+!"#$#%&&+%;!"#$#%&&+%(!"#$#%&&+%9!"#$#%&&+%%!"#$#%&&+%'!"#$#%&&+%:!"#$#%&&+%)!"#$#%&&+%&!"#$#%&&+'*!"#$#%&&+'+!"#$#%&&+';!"#$#%&&+'(!"#$#%&&+'9!"#$#%&&+':!"#$#%&&+')!"#$#%&&+'&!"#$#%&&+:*!"#$#%&&+:+

!"#$#%&&+:;!"#$#%&&+:(!"#$#%&&+:9!"#$#%&&+:%!"#$#%&&+:'!"#$#%&&+::!"#$#%&&+:)!"#$#%&&+:&!"#$#%&&+)*!"#$#%&&+)+!"#$#%&&+);!"#$#%&&+)9!"#$#%&&+)%!"#$#%&&+)'!"#$#%&&+):!"#$#%&&+))!"#$#%&&+)&!"#$#%&&+&*!"#$#%&&+&+!"#$#%&&+&;!"#$#%&&+&(!"#$#%&&+&%!"#$#%&&+&'!"#$#%&&+&:

!"#$#%&&+&)!"#$#%&&+&&!"#$#%&&;**!"#$#%&&;*;!"#$#%&&;*(!"#$#%&&;*9!"#$#%&&;*'!"#$#%&&;*:!"#$#%&&;*)!"#$#%&&;*&!"#$#%&&;+*!"#$#%&&;++!"#$#%&&;+;!"#$#%&&;+(!"#$#%&&;+9!"#$#%&&;+%!"#$#%&&;+'!"#$#%&&;+:!"#$#%&&;+)!"#$#%&&;+&!"#$#%&&;;*!"#$#%&&;;+!"#$#%&&;;;!"#$#%&&;;( !"#$#%&&;;9!"#$#%&&;;%!"#$#%&&;;'!"#$#%&&;;:!"#$#%&&;;&!"#$#%&&;(*!"#$#%&&;(+!"#$#%&&;(;!"#$#%&&;((!"#$#%&&;(9!"#$#%&&;(%!"#$#%&&;('!"#$#%&&;(:!"#$#%&&;()!"#$#%&&;(&!"#$#%&&;9*!"#$#%&&;9+!"#$#%&&;9;!"#$#%&&;9(!"#$#%&&;99!"#$#%&&;9%!"#$#%&&;9'!"#$#%&&;9:!"#$#%&&;9) !"#$#%&&;9&!"#$#%&&;%*!"#$#%&&;%+!"#$#%&&;%;!"#$#%&&;%(!"#$#%&&;%9!"#$#%&&;%%!"#$#%&&;%'!"#$#%&&;%:!"#$#%&&;%)!"#$#%&&;%&!"#$#%&&;'*!"#$#%&&;'+!"#$#%&&;';!"#$#%&&;'(!"#$#%&&;'9!"#$#%&&;'%!"#$#%&&;''!"#$#%&&;':!"#$#%&&;')!"#$#%&&;'&!"#$#%&&;:+!"#$#%&&;:;!"#$#%&&;:(

!"#$#%&&;:9!"#$#%&&;:%!"#$#%&&;:'!"#$#%&&;::!"#$#%&&;:)!"#$#%&&;:&!"#$#%&&;)*!"#$#%&&;)+!"#$#%&&;);!"#$#%&&;)(!"#$#%&&;)9!"#$#%&&;)%!"#$#%&&;)'!"#$#%&&;):!"#$#%&&;))!"#$#%&&;)&!"#$#%&&;&*!"#$#%&&;&+!"#$#%&&;&;!"#$#%&&;&(!"#$#%&&;&9!"#$#%&&;&%!"#$#%&&;&'!"#$#%&&;&:

!"#$#%&&;&)!"#$#%&&;&&!"#$#%&&(**!"#$#%&&(*+!"#$#%&&(*;!"#$#%&&(*(!"#$#%&&(*9!"#$#%&&(*%!"#$#%&&(*'!"#$#%&&(*:!"#$#%&&(*)!"#$#%&&(*&!"#$#%&&(+*!"#$#%&&(++!"#$#%&&(+;!"#$#%&&(+(!"#$#%&&(+%!"#$#%&&(+'!"#$#%&&(+:!"#$#%&&(+)!"#$#%&&(+&!"#$#%&&(;*!"#$#%&&(;+!"#$#%&&(;;

!"#$#%&&(;(!"#$#%&&(;9!"#$#%&&(;%!"#$#%&&(;'!"#$#%&&(;:!"#$#%&&(;)!"#$#%&&(;&!"#$#%&&((*!"#$#%&&((+!"#$#%&&((;!"#$#%&&(((!"#$#%&&((9!"#$#%&&((%!"#$#%&&(('!"#$#%&&((:!"#$#%&&(()!"#$#%&&((&!"#$#%&&(9*!"#$#%&&(9+!"#$#%&&(9;!"#$#%&&(9(!"#$#%&&(99!"#$#%&&(9%!"#$#%&&(9'

!"#$#%&&(9:!"#$#%&&(9)!"#$#%&&(9&!"#$#%&&(%*!"#$#%&&(%+!"#$#%&&(%;!"#$#%&&(%(!"#$#%&&(%9!"#$#%&&(%%!"#$#%&&(%'!"#$#%&&(%:!"#$#%&&(%)!"#$#%&&(%&!"#$#%&&('*!"#$#%&&('+!"#$#%&&(';!"#$#%&&('(!"#$#%&&('9!"#$#%&&('%!"#$#%&&(''!"#$#%&&(':!"#$#%&&(')!"#$#%&&('&!"#$#%&&(:*

!"#$#%&&(:+!"#$#%&&(:;!"#$#%&&(:(!"#$#%&&(:9!"#$#%&&(:%!"#$#%&&(:'!"#$#%&&(::!"#$#%&&(:)!"#$#%&&(:&!"#$#%&&()*!"#$#%&&()+!"#$#%&&();!"#$#%&&()(!"#$#%&&()9!"#$#%&&()%!"#$#%&&()'!"#$#%&&():!"#$#%&&())!"#$#%&&()&!"#$#%&&(&*!"#$#%&&(&+!"#$#%&&(&;!"#$#%&&(&(!"#$#%&&(&9

!"#$#%&&(&'!"#$#%&&(&:!"#$#%&&(&)!"#$#%&&(&&!"#$#%&&9**!"#$#%&&9*+!"#$#%&&9*;!"#$#%&&9*(!"#$#%&&9*9!"#$#%&&9*%!"#$#%&&9*'!"#$#%&&9*:!"#$#%&&9*)!"#$#%&&9*&!"#$#%&&9+*!"#$#%&&9++!"#$#%&&9+;!"#$#%&&9+(!"#$#%&&9+9!"#$#%&&9+%!"#$#%&&9+'!"#$#%&&9+:!"#$#%&&9+)!"#$#%&&9+& !"#$#%&&9;*!"#$#%&&9;+!"#$#%&&9;;!"#$#%&&9;(!"#$#%&&9;9!"#$#%&&9;%!"#$#%&&9;'!"#$#%&&9;:!"#$#%&&9;)!"#$#%&&9;&!"#$#%&&9(*!"#$#%&&9(+!"#$#%&&9(;!"#$#%&&9((!"#$#%&&9(9!"#$#%&&9(%!"#$#%&&9('!"#$#%&&9(:!"#$#%&&9()!"#$#%&&9(&!"#$#%&&99*!"#$#%&&99+!"#$#%&&99;!"#$#%&&99(

!"#$#%&&999!"#$#%&&99%!"#$#%&&99'!"#$#%&&99:!"#$#%&&99)!"#$#%&&99&!"#$#%&&9%*!"#$#%&&9%+!"#$#%&&9%;!"#$#%&&9%(!"#$#%&&9%9!"#$#%&&9%%!"#$#%&&9%'!"#$#%&&9%:!"#$#%&&9%)!"#$#%&&9%&!"#$#%&&9'*!"#$#%&&9'+!"#$#%&&9';!"#$#%&&9'(!"#$#%&&9'9!"#$#%&&9'%!"#$#%&&9''!"#$#%&&9':

!"#$#%&&9')!"#$#%&&9'&!"#$#%&&9:*!"#$#%&&9:+!"#$#%&&9:;!"#$#%&&9:(!"#$#%&&9:9!"#$#%&&9:%!"#$#%&&9:'!"#$#%&&9::!"#$#%&&9:&!"#$#%&&9)*!"#$#%&&9)+!"#$#%&&9);!"#$#%&&9)(!"#$#%&&9)9!"#$#%&&9)%!"#$#%&&9)'!"#$#%&&9):!"#$#%&&9))!"#$#%&&9)&!"#$#%&&9&+!"#$#%&&9&;!"#$#%&&9&(

!"#$#%&&9&9!"#$#%&&9&%!"#$#%&&9&'!"#$#%&&9&:!"#$#%&&9&)!"#$#%&&9&&!"#$#%&&%**!"#$#%&&%*+!"#$#%&&%*;!"#$#%&&%*(!"#$#%&&%*9!"#$#%&&%*%!"#$#%&&%*'!"#$#%&&%*)!"#$#%&&%*&!"#$#%&&%+*!"#$#%&&%++!"#$#%&&%+;!"#$#%&&%+(!"#$#%&&%+9!"#$#%&&%+%!"#$#%&&%+:!"#$#%&&%+)!"#$#%&&%+&

!"#$#%&&%;*!"#$#%&&%;+!"#$#%&&%;;!"#$#%&&%;(!"#$#%&&%;9!"#$#%&&%;%!"#$#%&&%;'!"#$#%&&%;:!"#$#%&&%;)!"#$#%&&%;&!"#$#%&&%(*!"#$#%&&%(+!"#$#%&&%(;!"#$#%&&%((!"#$#%&&%(9!"#$#%&&%(%!"#$#%&&%('!"#$#%&&%(:!"#$#%&&%()!"#$#%&&%(&!"#$#%&&%9*!"#$#%&&%9+!"#$#%&&%9;!"#$#%&&%9(

!"#$#%&&%99!"#$#%&&%9%!"#$#%&&%9'!"#$#%&&%9:!"#$#%&&%9)!"#$#%&&%9&!"#$#%&&%%*!"#$#%&&%%+!"#$#%&&%%;!"#$#%&&%%(!"#$#%&&%%%!"#$#%&&%%'!"#$#%&&%%:!"#$#%&&%%&!"#$#%&&%'*!"#$#%&&%'+!"#$#%&&%';!"#$#%&&%'(!"#$#%&&%'%!"#$#%&&%''!"#$#%&&%':!"#$#%&&%')!"#$#%&&%'&!"#$#%&&%:*

!"#$#%&&%:+!"#$#%&&%:;!"#$#%&&%:(!"#$#%&&%:9!"#$#%&&%:%!"#$#%&&%:'!"#$#%&&%::!"#$#%&&%:)!"#$#%&&%:&!"#$#%&&%)*!"#$#%&&%)+!"#$#%&&%);!"#$#%&&%)(!"#$#%&&%)9!"#$#%&&%)%!"#$#%&&%)'!"#$#%&&%))!"#$#%&&%)&!"#$#%&&%&+!"#$#%&&%&;!"#$#%&&%&(!"#$#%&&%&9!"#$#%&&%&'!"#$#%&&%&: !"#$#%&&%&)!"#$#%&&%&&!"#$#%&&'**!"#$#%&&'*+!"#$#%&&'*;!"#$#%&&'*(!"#$#%&&'*%!"#$#%&&'*'!"#$#%&&'*:!"#$#%&&'*)!"#$#%&&'*&!"#$#%&&'+*!"#$#%&&'++!"#$#%&&'+;!"#$#%&&'+(!"#$#%&&'+9!"#$#%&&'+%!"#$#%&&'+'!"#$#%&&'+:!"#$#%&&'+)!"#$#%&&'+&!"#$#%&&';*!"#$#%&&';+!"#$#%&&';;

!"#$#%&&';(!"#$#%&&';9!"#$#%&&';%!"#$#%&&';'!"#$#%&&';&!"#$#%&&'(*!"#$#%&&'(+!"#$#%&&'(;!"#$#%&&'((!"#$#%&&'(9!"#$#%&&'(%!"#$#%&&'('!"#$#%&&'(:!"#$#%&&'()!"#$#%&&'(&!"#$#%&&'9*!"#$#%&&'9+!"#$#%&&'9;!"#$#%&&'9(!"#$#%&&'99!"#$#%&&'9%!"#$#%&&'9'!"#$#%&&'9:!"#$#%&&'9& !"#$#%&&'%*!"#$#%&&'%+!"#$#%&&'%;!"#$#%&&'%(!"#$#%&&'%9!"#$#%&&'%%!"#$#%&&'%'!"#$#%&&'%:!"#$#%&&'%)!"#$#%&&'%&!"#$#%&&''*!"#$#%&&''+!"#$#%&&'';!"#$#%&&''9!"#$#%&&''%!"#$#%&&'''!"#$#%&&'':!"#$#%&&'')!"#$#%&&''&!"#$#%&&':*!"#$#%&&':+!"#$#%&&':;!"#$#%&&':(!"#$#%&&':9

!"#$#%&&':%!"#$#%&&':'!"#$#%&&'::!"#$#%&&':)!"#$#%&&':&!"#$#%&&')*!"#$#%&&')+!"#$#%&&');!"#$#%&&')(!"#$#%&&')9!"#$#%&&')%!"#$#%&&')'!"#$#%&&'):!"#$#%&&'))!"#$#%&&')&!"#$#%&&'&*!"#$#%&&'&+!"#$#%&&'&;!"#$#%&&'&(!"#$#%&&'&9!"#$#%&&'&%!"#$#%&&'&'!"#$#%&&'&:!"#$#%&&'&)

!"#$#%&&'&&!"#$#%&&:**!"#$#%&&:*+!"#$#%&&:*;!"#$#%&&:*(!"#$#%&&:*9!"#$#%&&:*%!"#$#%&&:*'!"#$#%&&:*:!"#$#%&&:*)!"#$#%&&:*&!"#$#%&&:+*!"#$#%&&:++!"#$#%&&:+;!"#$#%&&:+(!"#$#%&&:+9!"#$#%&&:+%!"#$#%&&:+'!"#$#%&&:+:!"#$#%&&:+)!"#$#%&&:+&!"#$#%&&:;*!"#$#%&&:;+!"#$#%&&:;;

!"#$#%&&:;(!"#$#%&&:;9!"#$#%&&:;%!"#$#%&&:;'!"#$#%&&:;:!"#$#%&&:;)!"#$#%&&:;&!"#$#%&&:(*!"#$#%&&:(;!"#$#%&&:((!"#$#%&&:(9!"#$#%&&:(%!"#$#%&&:('!"#$#%&&:(:!"#$#%&&:()!"#$#%&&:(&!"#$#%&&:9*!"#$#%&&:9+!"#$#%&&:9;!"#$#%&&:9(!"#$#%&&:99!"#$#%&&:9%!"#$#%&&:9'!"#$#%&&:9:

!"#$#%&&:9)!"#$#%&&:9&!"#$#%&&:%*!"#$#%&&:%+!"#$#%&&:%;!"#$#%&&:%(!"#$#%&&:%9!"#$#%&&:%%!"#$#%&&:%'!"#$#%&&:%:!"#$#%&&:%)!"#$#%&&:%&!"#$#%&&:'*!"#$#%&&:'+!"#$#%&&:';!"#$#%&&:'9!"#$#%&&:''!"#$#%&&:':!"#$#%&&:')!"#$#%&&:'&!"#$#%&&::*!"#$#%&&::+!"#$#%&&::;!"#$#%&&::(

!"#$#%&&::9!"#$#%&&::%!"#$#%&&::'!"#$#%&&:::!"#$#%&&::)!"#$#%&&::&!"#$#%&&:)*!"#$#%&&:)+!"#$#%&&:);!"#$#%&&:)9!"#$#%&&:)%!"#$#%&&:)'!"#$#%&&:):!"#$#%&&:))!"#$#%&&:)&!"#$#%&&:&*!"#$#%&&:&+!"#$#%&&:&;!"#$#%&&:&(!"#$#%&&:&9!"#$#%&&:&%!"#$#%&&:&'!"#$#%&&:&:!"#$#%&&:&) !"#$#%&&:&&!"#$#%&&)**!"#$#%&&)*+!"#$#%&&)*;!"#$#%&&)*(!"#$#%&&)*9!"#$#%&&)*%!"#$#%&&)*'!"#$#%&&)*:!"#$#%&&)*)!"#$#%&&)*&!"#$#%&&)+*!"#$#%&&)+(!"#$#%&&)+9!"#$#%&&)+%!"#$#%&&)+:

$ $ .A=+& 5A 8 6. . / < 4 !56 0 0

?6. 6 . $0 .A=+& 7EFGG G=H

!"#$#%&&)+)!"#$#%&&)+&!"#$#%&&);*!"#$#%&&);+!"#$#%&&);;!"#$#%&&);(!"#$#%&&);9!"#$#%&&);%!"#$#%&&);'!"#$#%&&);:!"#$#%&&);)!"#$#%&&);&!"#$#%&&)(*!"#$#%&&)(+!"#$#%&&)((!"#$#%&&)(%!"#$#%&&)('!"#$#%&&)(:!"#$#%&&)()!"#$#%&&)(&!"#$#%&&)9*!"#$#%&&)9+!"#$#%&&)9;!"#$#%&&)9( !"#$#%&&)99!"#$#%&&)9'!"#$#%&&)9:!"#$#%&&)9)!"#$#%&&)9&!"#$#%&&)%*!"#$#%&&)%+!"#$#%&&)%;!"#$#%&&)%(!"#$#%&&)%9!"#$#%&&)%%!"#$#%&&)%'!"#$#%&&)%:!"#$#%&&)%)!"#$#%&&)%&!"#$#%&&)'*!"#$#%&&)'+!"#$#%&&)';!"#$#%&&)'(!"#$#%&&)'9!"#$#%&&)'%!"#$#%&&)''!"#$#%&&)':!"#$#%&&)')

!"#$#%&&)'&!"#$#%&&):*!"#$#%&&):+!"#$#%&&):;!"#$#%&&):(!"#$#%&&):9!"#$#%&&):%!"#$#%&&):'!"#$#%&&)::!"#$#%&&):)!"#$#%&&):&!"#$#%&&))*!"#$#%&&))+!"#$#%&&));!"#$#%&&))(!"#$#%&&))9!"#$#%&&))%!"#$#%&&))'!"#$#%&&)):!"#$#%&&))&!"#$#%&&)&*!"#$#%&&)&+!"#$#%&&)&;!"#$#%&&)&(

!"#$#%&&)&9!"#$#%&&)&%!"#$#%&&)&'!"#$#%&&)&:!"#$#%&&)&)!"#$#%&&)&&!"#$#%&&&**!"#$#%&&&*+!"#$#%&&&*;!"#$#%&&&*(!"#$#%&&&*%!"#$#%&&&*'!"#$#%&&&*:!"#$#%&&&*)!"#$#%&&&*&!"#$#%&&&+*!"#$#%&&&++!"#$#%&&&+;!"#$#%&&&+9!"#$#%&&&+%!"#$#%&&&+'!"#$#%&&&+:!"#$#%&&&+)!"#$#%&&&+&

!"#$#%&&&;*!"#$#%&&&;+!"#$#%&&&;;!"#$#%&&&;(!"#$#%&&&;9!"#$#%&&&;%!"#$#%&&&;'!"#$#%&&&;:!"#$#%&&&;)!"#$#%&&&;&!"#$#%&&&(*!"#$#%&&&(+!"#$#%&&&(;!"#$#%&&&((!"#$#%&&&(9!"#$#%&&&(%!"#$#%&&&('!"#$#%&&&(:!"#$#%&&&()!"#$#%&&&(&!"#$#%&&&9*!"#$#%&&&9+!"#$#%&&&9;!"#$#%&&&9(

!"#$#%&&&99!"#$#%&&&9%!"#$#%&&&9'!"#$#%&&&9:!"#$#%&&&9&!"#$#%&&&%*!"#$#%&&&%+!"#$#%&&&%;!"#$#%&&&%(!"#$#%&&&%9!"#$#%&&&%%!"#$#%&&&%'!"#$#%&&&%:!"#$#%&&&%)!"#$#%&&&%&!"#$#%&&&'+!"#$#%&&&';!"#$#%&&&'(!"#$#%&&&'9!"#$#%&&&'%!"#$#%&&&''!"#$#%&&&':!"#$#%&&&')!"#$#%&&&'&

!"#$#%&&&:*!"#$#%&&&:+!"#$#%&&&:;!"#$#%&&&:9!"#$#%&&&:%!"#$#%&&&:'!"#$#%&&&::!"#$#%&&&:)!"#$#%&&&:&!"#$#%&&&)*!"#$#%&&&)+!"#$#%&&&);!"#$#%&&&)(!"#$#%&&&)9!"#$#%&&&)%!"#$#%&&&)'!"#$#%&&&):!"#$#%&&&))!"#$#%&&&)&!"#$#%&&&&*!"#$#%&&&&+!"#$#%&&&&;!"#$#%&&&&(!"#$#%&&&&9 !"#$#%&&&&'!"#$#%&&&&:!"#$#%&&&&)!"#$#%&&&&&!"#$#'*****!"#$#'****+!"#$#'****;!"#$#'****(!"#$#'****9!"#$#'****%!"#$#'****'!"#$#'****:!"#$#'****)!"#$#'****&!"#$#'***+*!"#$#'***++!"#$#'***+;!"#$#'***+(!"#$#'***+9!"#$#'***+%!"#$#'***+'!"#$#'***+:!"#$#'***+)!"#$#'***+& !"#$#'***;*!"#$#'***;+!"#$#'***;;!"#$#'***;(!"#$#'***;9!"#$#'***;%!"#$#'***;'!"#$#'***;:!"#$#'***;)!"#$#'***;&!"#$#'***(*!"#$#'***(+!"#$#'***(;!"#$#'***((!"#$#'***(9!"#$#'***(%!"#$#'***('!"#$#'***(:!"#$#'***()!"#$#'***(&!"#$#'***9*!"#$#'***9+!"#$#'***9;!"#$#'***9(

!"#$#'***99!"#$#'***9%!"#$#'***9'!"#$#'***9:!"#$#'***9)!"#$#'***9&!"#$#'***%*!"#$#'***%+!"#$#'***%;!"#$#'***%(!"#$#'***%9!"#$#'***%%!"#$#'***%:!"#$#'***%)!"#$#'***%&!"#$#'***'*!"#$#'***'+!"#$#'***';!"#$#'***'(!"#$#'***'9!"#$#'***'%!"#$#'***''!"#$#'***':!"#$#'***')

!"#$#'***'&!"#$#'***:*!"#$#'***:+!"#$#'***:;!"#$#'***:(!"#$#'***:9!"#$#'***:%!"#$#'***:'!"#$#'***::!"#$#'***:)!"#$#'***:&!"#$#'***)*!"#$#'***)+!"#$#'***);!"#$#'***)9!"#$#'***)%!"#$#'***)'!"#$#'***):!"#$#'***))!"#$#'***)&!"#$#'***&*!"#$#'***&;!"#$#'***&(!"#$#'***&9

!"#$#'***&%!"#$#'***&'!"#$#'***&:!"#$#'***&)!"#$#'***&&!"#$#'**+**!"#$#'**+*+!"#$#'**+*;!"#$#'**+*(!"#$#'**+*9!"#$#'**+*%!"#$#'**+*'!"#$#'**+*)!"#$#'**+*&!"#$#'**++*!"#$#'**+++!"#$#'**++;!"#$#'**++(!"#$#'**++9!"#$#'**++%!"#$#'**++'!"#$#'**++:!"#$#'**++)!"#$#'**++& !"#$#'**+;*!"#$#'**+;+!"#$#'**+;;!"#$#'**+;(!"#$#'**+;9!"#$#'**+;%!"#$#'**+;'!"#$#'**+;:

$ $3 6 .A=+& 7$ $3 6 < 4 !56 0 06 . $0

?6. .A=+& 7EFGG G=H

!"#$#'**+;)!"#$#'**+;&!"#$#'**+(*!"#$#'**+(+!"#$#'**+(;!"#$#'**+((!"#$#'**+(9!"#$#'**+(%!"#$#'**+('!"#$#'**+(:!"#$#'**+()!"#$#'**+9*!"#$#'**+9+!"#$#'**+9;!"#$#'**+9(!"#$#'**+99!"#$#'**+9%!"#$#'**+9'!"#$#'**+9:!"#$#'**+9)!"#$#'**+9&!"#$#'**+%*!"#$#'**+%+!"#$#'**+%; !"#$#'**+%(!"#$#'**+%9!"#$#'**+%%!"#$#'**+%'!"#$#'**+%:!"#$#'**+%)!"#$#'**+%&!"#$#'**+'*!"#$#'**+'+!"#$#'**+';!"#$#'**+'(!"#$#'**+'9!"#$#'**+'%!"#$#'**+''!"#$#'**+':!"#$#'**+')!"#$#'**+'&!"#$#'**+:*!"#$#'**+:+!"#$#'**+:(!"#$#'**+:9!"#$#'**+:%!"#$#'**+:'!"#$#'**+:: !"#$#'**+:)!"#$#'**+:&!"#$#'**+)*!"#$#'**+)+!"#$#'**+);!"#$#'**+)(!"#$#'**+)9!"#$#'**+)%!"#$#'**+)'!"#$#'**+):!"#$#'**+))!"#$#'**+)&!"#$#'**+&*!"#$#'**+&+!"#$#'**+&;!"#$#'**+&(!"#$#'**+&9!"#$#'**+&%!"#$#'**+&'!"#$#'**+&:!"#$#'**+&)!"#$#'**+&&!"#$#'**;**!"#$#'**;*+ !"#$#'**;*;!"#$#'**;*(!"#$#'**;*9!"#$#'**;*%!"#$#'**;*'!"#$#'**;*:!"#$#'**;*)!"#$#'**;*&!"#$#'**;+*!"#$#'**;++!"#$#'**;+;!"#$#'**;+(!"#$#'**;+9!"#$#'**;+%!"#$#'**;+'!"#$#'**;+:!"#$#'**;+)!"#$#'**;+&!"#$#'**;;*!"#$#'**;;;!"#$#'**;;(!"#$#'**;;9!"#$#'**;;%!"#$#'**;;' !"#$#'**;;:!"#$#'**;;)!"#$#'**;;&!"#$#'**;(*!"#$#'**;(+!"#$#'**;(;!"#$#'**;((!"#$#'**;(9!"#$#'**;(%!"#$#'**;('!"#$#'**;()!"#$#'**;(&!"#$#'**;9*!"#$#'**;9+!"#$#'**;9;!"#$#'**;9(!"#$#'**;99!"#$#'**;9%!"#$#'**;9'!"#$#'**;9:!"#$#'**;9)!"#$#'**;9&!"#$#'**;%*!"#$#'**;%+

!"#$#'**;%;!"#$#'**;%9!"#$#'**;%%!"#$#'**;%'!"#$#'**;%:!"#$#'**;%)!"#$#'**;%&!"#$#'**;'*!"#$#'**;'+!"#$#'**;';!"#$#'**;'(!"#$#'**;'9!"#$#'**;'%!"#$#'**;''!"#$#'**;':!"#$#'**;')!"#$#'**;'&!"#$#'**;:*!"#$#'**;:+!"#$#'**;:;!"#$#'**;:(!"#$#'**;:9!"#$#'**;:%!"#$#'**;:'

!"#$#'**;::!"#$#'**;:)!"#$#'**;:&!"#$#'**;)*!"#$#'**;)+!"#$#'**;);!"#$#'**;)(!"#$#'**;)9!"#$#'**;)%!"#$#'**;)'!"#$#'**;):!"#$#'**;))!"#$#'**;)&!"#$#'**;&*!"#$#'**;&+!"#$#'**;&;!"#$#'**;&(!"#$#'**;&9!"#$#'**;&%!"#$#'**;&'!"#$#'**;&:!"#$#'**;&)!"#$#'**;&&!"#$#'**(**

!"#$#'**(*+!"#$#'**(*(!"#$#'**(*9!"#$#'**(*%!"#$#'**(*'!"#$#'**(*:!"#$#'**(*)!"#$#'**(*&!"#$#'**(+*!"#$#'**(++!"#$#'**(+;!"#$#'**(+(!"#$#'**(+9!"#$#'**(+%!"#$#'**(+'!"#$#'**(+:!"#$#'**(+)!"#$#'**(+&!"#$#'**(;*!"#$#'**(;+!"#$#'**(;;!"#$#'**(;(!"#$#'**(;9!"#$#'**(;%

!"#$#'**(;'!"#$#'**(;:!"#$#'**(;)!"#$#'**(;&!"#$#'**((*!"#$#'**((+!"#$#'**((;!"#$#'**(((!"#$#'**((9!"#$#'**((%!"#$#'**(('!"#$#'**((:!"#$#'**(()!"#$#'**((&!"#$#'**(9*!"#$#'**(9+!"#$#'**(9;!"#$#'**(9(!"#$#'**(99!"#$#'**(9%!"#$#'**(9'!"#$#'**(9:!"#$#'**(9)!"#$#'**(9&

!"#$#'**(%*!"#$#'**(%+!"#$#'**(%;!"#$#'**(%(!"#$#'**(%9!"#$#'**(%%!"#$#'**(%'!"#$#'**(%:!"#$#'**(%)!"#$#'**(%&!"#$#'**('*!"#$#'**('+!"#$#'**(';!"#$#'**('(!"#$#'**('9!"#$#'**('%!"#$#'**(''!"#$#'**(':!"#$#'**(')!"#$#'**('&!"#$#'**(:*!"#$#'**(:+!"#$#'**(:;!"#$#'**(:(

!"#$#'**(:9!"#$#'**(:%!"#$#'**(:'!"#$#'**(:)!"#$#'**(:&!"#$#'**()*!"#$#'**()+!"#$#'**();!"#$#'**()(!"#$#'**()9!"#$#'**()%!"#$#'**():!"#$#'**())!"#$#'**()&!"#$#'**(&*!"#$#'**(&+!"#$#'**(&;!"#$#'**(&(!"#$#'**(&9!"#$#'**(&%!"#$#'**(&'!"#$#'**(&:!"#$#'**(&)!"#$#'**(&&

!"#$#'**9**!"#$#'**9*+!"#$#'**9*;!"#$#'**9*(!"#$#'**9*9!"#$#'**9*'!"#$#'**9*:!"#$#'**9*)!"#$#'**9*&!"#$#'**9+*!"#$#'**9++!"#$#'**9+;!"#$#'**9+(!"#$#'**9+9!"#$#'**9+%!"#$#'**9+'!"#$#'**9+:!"#$#'**9+)!"#$#'**9+&!"#$#'**9;*!"#$#'**9;+!"#$#'**9;;!"#$#'**9;(!"#$#'**9;9 !"#$#'**9;%!"#$#'**9;'!"#$#'**9;:

|  | $ $ | .A=+&  ?6. | 5A 8 6. . / < 4 !56 0 0  6 . $0 .A=+& 7EFGG G=H |
| --- | --- | --- | --- |
| !"#$#'**9(&!"#$#'**999 | ! . 4 | 5 | $ . 3 543 3 /= = , @7 |

!"#$#'**99:!"#$#'**99)!"#$#'**9%*!"#$#'**9%;!"#$#'**9%(!"#$#'**9%%!"#$#'**9%:!"#$#'**9%)!"#$#'**9'*!"#$#'**9';!"#$#'**9'9!"#$#'**9'%!"#$#'**9':!"#$#'**9'&!"#$#'**9:*!"#$#'**9:;!"#$#'**9:9!"#$#'**9:%

|  | $ $ | .A=+&  ?6. | 5A 8 6. . / < 4 !56 0 0  6 . $0 .A=+& 7EFGG G=H |
| --- | --- | --- | --- |
| !"#$#'**9:' | ! . 4 | 5 | $ . 3 543 3 /= = , @7 |
| !"#$#'**9:: | $ $ | .A=+&  ?6. | 5A 8 6. . / < 4 !56 0 0  6 . $0 .A=+& 7EFGG G=H |
| !"#$#'**9:) | ! . 4 | 5 | $ . 3 543 3 /= = , @7 |
| !"#$#'**9:& | $ $ | .A=+&  ?6. | 5A 8 6. . / < 4 !56 0 0  6 . $0 .A=+& 7EFGG G=H |
| !"#$#'**9)* | ! . 4 | 5 | $ . 3 543 3 /= = , @7 |
| !"#$#'**9)+!"#$#'**9);!"#$#'**9)(!"#$#'**9)% !"#$#'**9)'!"#$#'**9))!"#$#'**9&*!"#$#'**9&+ | $ $ | .A=+&  ?6. | 5A 8 6. . / < 4 !56 0 0  6 . $0 .A=+& 7EFGG G=H |
| !"#$#'**9&; | ! . 4 | 5 | $ . 3 543 3 /= = , @7 |
| !"#$#'**9&(!"#$#'**9&9 | $ $ | .A=+&  ?6. | 5A 8 6. . / < 4 !56 0 0  6 . $0 .A=+& 7EFGG G=H |
| !"#$#'**9&% | ! . 4 | 5 | $ . 3 543 3 /= = , @7 |
| !"#$#'**9&'!"#$#'**9&: | $ $ | .A=+&  ?6. | 5A 8 6. . / < 4 !56 0 0  6 . $0 .A=+& 7EFGG G=H |
| !"#$#'**9&) | ! . 4 | 5 | $ . 3 543 3 /= = , @7 |

!"#$#'**9&&!"#$#'**%*+!"#$#'**%*(!"#$#'**%*9!"#$#'**%*'!"#$#'**%*)!"#$#'**%*&!"#$#'**%++!"#$#'**%+(!"#$#'**%+9!"#$#'**%+'!"#$#'**%+)!"#$#'**%;*

|  | $ $ | .A=+&  ?6. | 5A 8 6. . / < 4 !56 0 0  6 . $0 .A=+& 7EFGG G=H |
| --- | --- | --- | --- |
| !"#$#'**%;+!"#$#'**%;( | $ $ " | .A=+&  ?6. | 0B 35 7$ $ " < 4 !56 0 06 .  $0 .A=+& 7EFGG G=H |
| !"#$#'**%;9 | ! . 4 | 5 | $ . 3 543 3 /= = , @7 |
| !"#$#'**%;%!"#$#'**%;'!"#$#'**%;)!"#$#'**%;&  !"#$#'**%(+!"#$#'**%(;!"#$#'**%(9!"#$#'**%(' !"#$#'**%(: | $ $ " | .A=+&  ?6. | 0B 35 7$ $ " < 4 !56 0 06 .  $0 .A=+& 7EFGG G=H |

!"#$#'**%(&!"#$#'**%9*!"#$#'**%9;!"#$#'**%99!"#$#'**%9%!"#$#'**%9:!"#$#'**%9)!"#$#'**%%*!"#$#'**%%+!"#$#'**%%(!"#$#'**%%%!"#$#'**%%'!"#$#'**%%)!"#$#'**%'*!"#$#'**%'+!"#$#'**%'(!"#$#'**%'%!"#$#'**%''!"#$#'**%')!"#$#'**%'&!"#$#'**%:+!"#$#'**%:;!"#$#'**%:(!"#$#'**%:9 !"#$#'**%:%!"#$#'**%:'!"#$#'**%::!"#$#'**%:)!"#$#'**%:&!"#$#'**%)*!"#$#'**%)+!"#$#'**%);!"#$#'**%)(!"#$#'**%)9!"#$#'**%)%!"#$#'**%)'!"#$#'**%):!"#$#'**%))!"#$#'**%)&!"#$#'**%&*!"#$#'**%&+!"#$#'**%&;!"#$#'**%&9!"#$#'**%&%!"#$#'**%&'!"#$#'**%&:!"#$#'**%&)!"#$#'**%&& !"#$#'**'**!"#$#'**'*+!"#$#'**'*;!"#$#'**'*(!"#$#'**'*9!"#$#'**'*'!"#$#'**'*:!"#$#'**'*)!"#$#'**'*&!"#$#'**'+*!"#$#'**'++!"#$#'**'+;!"#$#'**'+(!"#$#'**'+9!"#$#'**'+%!"#$#'**'+'!"#$#'**'+:!"#$#'**'+)!"#$#'**'+&!"#$#'**';*!"#$#'**';+!"#$#'**';;!"#$#'**';(!"#$#'**';9

!"#$#'**';%!"#$#'**';'!"#$#'**';:!"#$#'**';)!"#$#'**';&!"#$#'**'(*!"#$#'**'(+!"#$#'**'(;!"#$#'**'((!"#$#'**'(9!"#$#'**'('!"#$#'**'(:!"#$#'**'()!"#$#'**'(&!"#$#'**'9*!"#$#'**'9+!"#$#'**'9;!"#$#'**'99!"#$#'**'9%!"#$#'**'9'!"#$#'**'9:!"#$#'**'9)!"#$#'**'9&!"#$#'**'%*

!"#$#'**'%+!"#$#'**'%;!"#$#'**'%(!"#$#'**'%9!"#$#'**'%%!"#$#'**'%'!"#$#'**'%:!"#$#'**'%)!"#$#'**'%&!"#$#'**''*!"#$#'**''+!"#$#'**'';!"#$#'**''(!"#$#'**''9!"#$#'**''%!"#$#'**'''!"#$#'**'':!"#$#'**'')!"#$#'**''&!"#$#'**':*!"#$#'**':+!"#$#'**':;!"#$#'**':(!"#$#'**':9

!"#$#'**':%!"#$#'**':'!"#$#'**'::!"#$#'**':)!"#$#'**':&!"#$#'**')*!"#$#'**')+!"#$#'**');!"#$#'**')(!"#$#'**')9!"#$#'**')%!"#$#'**')'!"#$#'**'):!"#$#'**'))!"#$#'**')&!"#$#'**'&*!"#$#'**'&+!"#$#'**'&;!"#$#'**'&(!"#$#'**'&9!"#$#'**'&%!"#$#'**'&'!"#$#'**'&:!"#$#'**'&) !"#$#'**'&&!"#$#'**:**!"#$#'**:*+!"#$#'**:*;!"#$#'**:*(!"#$#'**:*9!"#$#'**:*%!"#$#'**:*'!"#$#'**:*:!"#$#'**:*)!"#$#'**:*&!"#$#'**:+*!"#$#'**:++!"#$#'**:+;!"#$#'**:+(!"#$#'**:+9!"#$#'**:+%!"#$#'**:+'!"#$#'**:+:!"#$#'**:+)!"#$#'**:+&!"#$#'**:;*!"#$#'**:;+!"#$#'**:;; !"#$#'**:;(!"#$#'**:;9!"#$#'**:;%!"#$#'**:;'!"#$#'**:;:!"#$#'**:;)!"#$#'**:;&!"#$#'**:(*!"#$#'**:(+!"#$#'**:(;!"#$#'**:((!"#$#'**:(9!"#$#'**:(%!"#$#'**:('!"#$#'**:(:!"#$#'**:()!"#$#'**:(&!"#$#'**:9*!"#$#'**:9+!"#$#'**:9;!"#$#'**:9(!"#$#'**:99!"#$#'**:9%!"#$#'**:9'

!"#$#'**:9:!"#$#'**:9)!"#$#'**:9&!"#$#'**:%*!"#$#'**:%+!"#$#'**:%;!"#$#'**:%(!"#$#'**:%9!"#$#'**:%%!"#$#'**:%'!"#$#'**:%:!"#$#'**:%)!"#$#'**:%&!"#$#'**:'*!"#$#'**:'+!"#$#'**:';!"#$#'**:'(!"#$#'**:'9!"#$#'**:'%!"#$#'**:''!"#$#'**:':!"#$#'**:')!"#$#'**:'&!"#$#'**::*

!"#$#'**::+!"#$#'**::;!"#$#'**::(!"#$#'**::9!"#$#'**::%!"#$#'**::'!"#$#'**:::!"#$#'**::)!"#$#'**::&!"#$#'**:)*!"#$#'**:)+!"#$#'**:);!"#$#'**:)(!"#$#'**:)%!"#$#'**:)'!"#$#'**:):!"#$#'**:))!"#$#'**:)&!"#$#'**:&*!"#$#'**:&+!"#$#'**:&;!"#$#'**:&(!"#$#'**:&9!"#$#'**:&%

!"#$#'**:&'!"#$#'**:&:!"#$#'**:&)!"#$#'**:&&!"#$#'**)**!"#$#'**)*+!"#$#'**)*;!"#$#'**)*(!"#$#'**)*9!"#$#'**)*%!"#$#'**)*'!"#$#'**)*:!"#$#'**)*)!"#$#'**)*&!"#$#'**)+*!"#$#'**)++!"#$#'**)+;!"#$#'**)+(!"#$#'**)+9!"#$#'**)+%!"#$#'**)+'!"#$#'**)+:!"#$#'**)+)!"#$#'**)+&

!"#$#'**);*!"#$#'**);+!"#$#'**);;!"#$#'**);(!"#$#'**);%!"#$#'**);'!"#$#'**);:!"#$#'**);)!"#$#'**);&!"#$#'**)(*!"#$#'**)(+!"#$#'**)(;!"#$#'**)((!"#$#'**)(9!"#$#'**)(%!"#$#'**)('!"#$#'**)(:!"#$#'**)()!"#$#'**)(&!"#$#'**)9*!"#$#'**)9+!"#$#'**)9;!"#$#'**)9(!"#$#'**)99

!"#$#'**)9%!"#$#'**)9'!"#$#'**)9:!"#$#'**)9)!"#$#'**)9&!"#$#'**)%*!"#$#'**)%;!"#$#'**)%(!"#$#'**)%9!"#$#'**)%%!"#$#'**)%'!"#$#'**)%:!"#$#'**)%)!"#$#'**)%&!"#$#'**)'*!"#$#'**)'+!"#$#'**)';!"#$#'**)'(!"#$#'**)'9!"#$#'**)'%!"#$#'**)''!"#$#'**)':!"#$#'**)')!"#$#'**)'& !"#$#'**):*!"#$#'**):+!"#$#'**):;!"#$#'**):(!"#$#'**):9!"#$#'**):%!"#$#'**):'!"#$#'**)::!"#$#'**):)!"#$#'**):&!"#$#'**))*!"#$#'**))+!"#$#'**));!"#$#'**))(!"#$#'**))9!"#$#'**))%!"#$#'**))'!"#$#'**)):!"#$#'**)))!"#$#'**))&!"#$#'**)&*!"#$#'**)&+

|  | $ $ | .A=+&  ?6. | 5A 8 6. . / < 4 !56 0 0  6 . $0 .A=+& 7EFGG G=H |
| --- | --- | --- | --- |
| !"#$#'**)&; | $ $3 6 | .A=+&  ?6. | 7$ $3 6 < 4 !56 0 06 . $0 .A=+& 7EFGG G=H |
| !"#$#'**)&(!"#$#'**)&9!"#$#'**)&%!"#$#'**)&' !"#$#'**)&:!"#$#'**)&)!"#$#'**)&&!"#$#'**&** | $ $ | .A=+&  ?6. | 5A 8 6. . / < 4 !56 0 0  6 . $0 .A=+& 7EFGG G=H |
| !"#$#'**&*+ | $ $3 6 | .A=+&  ?6. | 7$ $3 6 < 4 !56 0 06 . $0 .A=+& 7EFGG G=H |
| !"#$#'**&*;!"#$#'**&*(!"#$#'**&*%!"#$#'**&*' !"#$#'**&*:!"#$#'**&*)!"#$#'**&*&!"#$#'**&+* !"#$#'**&++ | $ $ | .A=+&  ?6. | 5A 8 6. . / < 4 !56 0 0  6 . $0 .A=+& 7EFGG G=H |
| !"#$#'**&+;!"#$#'**&+( | $ $3 6 | .A=+&  ?6. | 7$ $3 6 < 4 !56 0 06 . $0 .A=+& 7EFGG G=H |
| !"#$#'**&+9 | $ $ | .A=+&  ?6. | 5A 8 6. . / < 4 !56 0 0  6 . $0 .A=+& 7EFGG G=H |
| !"#$#'**&+% | $ $3 6 | .A=+&  ?6. | 7$ $3 6 < 4 !56 0 06 . $0 .A=+& 7EFGG G=H |
| !"#$#'**&+'!"#$#'**&+:!"#$#'**&+)!"#$#'**&+& !"#$#'**&;*!"#$#'**&;+!"#$#'**&;; | $ $ | .A=+&  ?6. | 5A 8 6. . / < 4 !56 0 0  6 . $0 .A=+& 7EFGG G=H |
| !"#$#'**&;( | $ $3 6 | .A=+&  ?6. | 7$ $3 6 < 4 !56 0 06 . $0 .A=+& 7EFGG G=H |
| !"#$#'**&;9 | $ $ | .A=+&  ?6. | 5A 8 6. . / < 4 !56 0 0  6 . $0 .A=+& 7EFGG G=H |
| !"#$#'**&;%!"#$#'**&;'!"#$#'**&;:!"#$#'**&;) !"#$#'**&;&!"#$#'**&(*!"#$#'**&(+ | $ $3 6 | .A=+&  ?6. | 7$ $3 6 < 4 !56 0 06 . $0 .A=+& 7EFGG G=H |

!"#$#'**&(;!"#$#'**&((!"#$#'**&(9!"#$#'**&(%!"#$#'**&('!"#$#'**&(:!"#$#'**&()!"#$#'**&(&!"#$#'**&9*!"#$#'**&9+!"#$#'**&9;!"#$#'**&9(!"#$#'**&99!"#$#'**&9%!"#$#'**&9'!"#$#'**&9:!"#$#'**&9)!"#$#'**&9&!"#$#'**&%*!"#$#'**&%+!"#$#'**&%;!"#$#'**&%(!"#$#'**&%9!"#$#'**&%% !"#$#'**&%'!"#$#'**&%:!"#$#'**&%)!"#$#'**&%&!"#$#'**&'*!"#$#'**&'+!"#$#'**&';!"#$#'**&'(!"#$#'**&'9!"#$#'**&'%!"#$#'**&''!"#$#'**&':!"#$#'**&')!"#$#'**&'&!"#$#'**&:*!"#$#'**&:+!"#$#'**&:;!"#$#'**&:(!"#$#'**&:9!"#$#'**&:%!"#$#'**&:'!"#$#'**&::!"#$#'**&:)!"#$#'**&:&

!"#$#'**&)*!"#$#'**&)+!"#$#'**&);!"#$#'**&)(!"#$#'**&)9!"#$#'**&)%!"#$#'**&)'!"#$#'**&):!"#$#'**&))!"#$#'**&)&!"#$#'**&&*!"#$#'**&&+!"#$#'**&&;!"#$#'**&&(!"#$#'**&&9!"#$#'**&&%!"#$#'**&&'!"#$#'**&&:!"#$#'**&&)!"#$#'**&&&!"#$#'*+***!"#$#'*+**+!"#$#'*+**;!"#$#'*+**( !"#$#'*+**9!"#$#'*+**%!"#$#'*+**'!"#$#'*+**:!"#$#'*+**)!"#$#'*+**&!"#$#'*+*+*!"#$#'*+*++!"#$#'*+*+;!"#$#'*+*+(!"#$#'*+*+9!"#$#'*+*+%!"#$#'*+*+'!"#$#'*+*+:!"#$#'*+*+)!"#$#'*+*+&!"#$#'*+*;*!"#$#'*+*;+!"#$#'*+*;(!"#$#'*+*;9!"#$#'*+*;%!"#$#'*+*;'!"#$#'*+*;:!"#$#'*+*;) !"#$#'*+*;&!"#$#'*+*(*!"#$#'*+*(+!"#$#'*+*(;!"#$#'*+*((!"#$#'*+*(9!"#$#'*+*(%!"#$#'*+*('!"#$#'*+*(:!"#$#'*+*()!"#$#'*+*(&!"#$#'*+*9*!"#$#'*+*9+!"#$#'*+*9;!"#$#'*+*9(!"#$#'*+*99!"#$#'*+*9%!"#$#'*+*9'!"#$#'*+*9)!"#$#'*+*9&!"#$#'*+*%*!"#$#'*+*%+!"#$#'*+*%;!"#$#'*+*%(

!"#$#'*+*%9!"#$#'*+*%%!"#$#'*+*%'!"#$#'*+*%:!"#$#'*+*%)!"#$#'*+*%&!"#$#'*+*'*!"#$#'*+*';!"#$#'*+*'(!"#$#'*+*'9!"#$#'*+*'%!"#$#'*+*''!"#$#'*+*':!"#$#'*+*')!"#$#'*+*'&!"#$#'*+*:*!"#$#'*+*:;!"#$#'*+*:(!"#$#'*+*:9!"#$#'*+*:%!"#$#'*+*:'!"#$#'*+*::!"#$#'*+*:)!"#$#'*+*:&

!"#$#'*+*)*!"#$#'*+*)+!"#$#'*+*);!"#$#'*+*)(!"#$#'*+*)9!"#$#'*+*)%!"#$#'*+*)'!"#$#'*+*):!"#$#'*+*))!"#$#'*+*)&!"#$#'*+*&*!"#$#'*+*&+!"#$#'*+*&;!"#$#'*+*&(!"#$#'*+*&9!"#$#'*+*&%!"#$#'*+*&'!"#$#'*+*&:!"#$#'*+*&)!"#$#'*+*&&!"#$#'*++**!"#$#'*++*+!"#$#'*++*;!"#$#'*++*(

!"#$#'*++*9!"#$#'*++*%!"#$#'*++*'!"#$#'*++*:!"#$#'*++*)!"#$#'*++*&!"#$#'*+++*!"#$#'*++++!"#$#'*+++;!"#$#'*+++(!"#$#'*+++9!"#$#'*+++%!"#$#'*+++'!"#$#'*+++:!"#$#'*+++)!"#$#'*+++&!"#$#'*++;*!"#$#'*++;+!"#$#'*++;;!"#$#'*++;(!"#$#'*++;9!"#$#'*++;%!"#$#'*++;'!"#$#'*++;:

!"#$#'*++;)!"#$#'*++;&!"#$#'*++(*!"#$#'*++(+!"#$#'*++(;!"#$#'*++((!"#$#'*++(9!"#$#'*++(%!"#$#'*++('!"#$#'*++(:!"#$#'*++()!"#$#'*++(&!"#$#'*++9*!"#$#'*++9+!"#$#'*++9;!"#$#'*++9(!"#$#'*++99!"#$#'*++9%!"#$#'*++9'!"#$#'*++9:!"#$#'*++9)!"#$#'*++9&!"#$#'*++%+!"#$#'*++%;

!"#$#'*++%(!"#$#'*++%9!"#$#'*++%%!"#$#'*++%'!"#$#'*++%:!"#$#'*++%)!"#$#'*++%&!"#$#'*++'*!"#$#'*++'+!"#$#'*++';!"#$#'*++'(!"#$#'*++'9!"#$#'*++'%!"#$#'*++''!"#$#'*++':!"#$#'*++')!"#$#'*++'&!"#$#'*++:*!"#$#'*++:+!"#$#'*++:;!"#$#'*++:(!"#$#'*++:9!"#$#'*++:%!"#$#'*++:'

!"#$#'*++::!"#$#'*++:)!"#$#'*++:&!"#$#'*++)*!"#$#'*++)+!"#$#'*++);!"#$#'*++)(!"#$#'*++)9!"#$#'*++)%!"#$#'*++)'!"#$#'*++):!"#$#'*++))!"#$#'*++)&!"#$#'*++&*!"#$#'*++&+!"#$#'*++&(!"#$#'*++&9!"#$#'*++&%!"#$#'*++&'!"#$#'*++&:!"#$#'*++&)!"#$#'*++&&!"#$#'*+;**!"#$#'*+;*+

!"#$#'*+;*;!"#$#'*+;*(!"#$#'*+;*9!"#$#'*+;*%!"#$#'*+;*'!"#$#'*+;*:!"#$#'*+;*)!"#$#'*+;*&!"#$#'*+;+*!"#$#'*+;++!"#$#'*+;+;!"#$#'*+;+(!"#$#'*+;+%!"#$#'*+;+'!"#$#'*+;+:!"#$#'*+;+)!"#$#'*+;+&!"#$#'*+;;*!"#$#'*+;;+!"#$#'*+;;;!"#$#'*+;;(

|  | $ $ | .A=+&  ?6. | 5A 8 6. . / < 4 !56 0 0  6 . $0 .A=+& 7EFGG G=H |
| --- | --- | --- | --- |
| !"#$#'*+;;9 | $ $3 6 | .A=+&  ?6. | 7$ $3 6 < 4 !56 0 06 . $0 .A=+& 7EFGG G=H |
| !"#$#'*+;;%!"#$#'*+;;'!"#$#'*+;;:!"#$#'*+;;) !"#$#'*+;;&!"#$#'*+;(*!"#$#'*+;(+!"#$#'*+;(; !"#$#'*+;(( | $ $ | .A=+&  ?6. | 5A 8 6. . / < 4 !56 0 0  6 . $0 .A=+& 7EFGG G=H |
| !"#$#'*+;(9!"#$#'*+;(% | $ $3 6 | .A=+&  ?6. | 7$ $3 6 < 4 !56 0 06 . $0 .A=+& 7EFGG G=H |
| !"#$#'*+;('!"#$#'*+;(:!"#$#'*+;() | $ $ | .A=+&  ?6. | 5A 8 6. . / < 4 !56 0 0  6 . $0 .A=+& 7EFGG G=H |
| !"#$#'*+;(&!"#$#'*+;9* | $ $3 6 | .A=+&  ?6. | 7$ $3 6 < 4 !56 0 06 . $0 .A=+& 7EFGG G=H |
| !"#$#'*+;9+ | $ $ | .A=+&  ?6. | 5A 8 6. . / < 4 !56 0 0  6 . $0 .A=+& 7EFGG G=H |
| !"#$#'*+;9;!"#$#'*+;9( | $ $3 6 | .A=+&  ?6. | 7$ $3 6 < 4 !56 0 06 . $0 .A=+& 7EFGG G=H |
| !"#$#'*+;99!"#$#'*+;9' | $ $ | .A=+&  ?6. | 5A 8 6. . / < 4 !56 0 0  6 . $0 .A=+& 7EFGG G=H |
| !"#$#'*+;9:!"#$#'*+;9)!"#$#'*+;9& | $ $3 6 | .A=+&  ?6. | 7$ $3 6 < 4 !56 0 06 . $0 .A=+& 7EFGG G=H |
| !"#$#'*+;%*!"#$#'*+;%+!"#$#'*+;%;!"#$#'*+;%(  !"#$#'*+;%9!"#$#'*+;%% | $ $ | .A=+&  ?6. | 5A 8 6. . / < 4 !56 0 0  6 . $0 .A=+& 7EFGG G=H |
| !"#$#'*+;%' | $ $3 6 | .A=+&  ?6. | 7$ $3 6 < 4 !56 0 06 . $0 .A=+& 7EFGG G=H |
| !"#$#'*+;%: | $ $ | .A=+&  ?6. | 5A 8 6. . / < 4 !56 0 0  6 . $0 .A=+& 7EFGG G=H |
| !"#$#'*+;%) | $ $3 6 | .A=+&  ?6. | 7$ $3 6 < 4 !56 0 06 . $0 .A=+& 7EFGG G=H |
| !"#$#'*+;%&!"#$#'*+;'*!"#$#'*+;'+ | $ $ | .A=+&  ?6. | 5A 8 6. . / < 4 !56 0 0  6 . $0 .A=+& 7EFGG G=H |
| !"#$#'*+;'(!"#$#'*+;'9 | $ $3 6 | .A=+&  ?6. | 7$ $3 6 < 4 !56 0 06 . $0 .A=+& 7EFGG G=H |
| !"#$#'*+;'%!"#$#'*+;'' | $ $ | .A=+&  ?6. | 5A 8 6. . / < 4 !56 0 0  6 . $0 .A=+& 7EFGG G=H |
| !"#$#'*+;':!"#$#'*+;') | $ $3 6 | .A=+&  ?6. | 7$ $3 6 < 4 !56 0 06 . $0 .A=+& 7EFGG G=H |
| !"#$#'*+;'& | $ $ | .A=+&  ?6. | 5A 8 6. . / < 4 !56 0 0  6 . $0 .A=+& 7EFGG G=H |
| !"#$#'*+;:* | $ $3 6 | .A=+&  ?6. | 7$ $3 6 < 4 !56 0 06 . $0 .A=+& 7EFGG G=H |
| !"#$#'*+;:+ | $ $ | .A=+&  ?6. | 5A 8 6. . / < 4 !56 0 0  6 . $0 .A=+& 7EFGG G=H |
| !"#$#'*+;:;!"#$#'*+;:( | $ $3 6 | .A=+&  ?6. | 7$ $3 6 < 4 !56 0 06 . $0 .A=+& 7EFGG G=H |
| !"#$#'*+;:9 | $ $ | .A=+&  ?6. | 5A 8 6. . / < 4 !56 0 0  6 . $0 .A=+& 7EFGG G=H |
| !"#$#'*+;:% | $ $3 6 | .A=+&  ?6. | 7$ $3 6 < 4 !56 0 06 . $0 .A=+& 7EFGG G=H |
| !"#$#'*+;:' | $ $ | .A=+&  ?6. | 5A 8 6. . / < 4 !56 0 0  6 . $0 .A=+& 7EFGG G=H |
| !"#$#'*+;::!"#$#'*+;:) | $ $3 6 | .A=+&  ?6. | 7$ $3 6 < 4 !56 0 06 . $0 .A=+& 7EFGG G=H |
| !"#$#'*+;:& | $ $ | .A=+&  ?6. | 5A 8 6. . / < 4 !56 0 0  6 . $0 .A=+& 7EFGG G=H |
| !"#$#'*+;)*!"#$#'*+;)+!"#$#'*+;);!"#$#'*+;)( | $ $3 6 | .A=+&  ?6. | 7$ $3 6 < 4 !56 0 06 . $0 .A=+& 7EFGG G=H |
| !"#$#'*+;)9!"#$#'*+;)%!"#$#'*+;)' | $ $ | .A=+&  ?6. | 5A 8 6. . / < 4 !56 0 0  6 . $0 .A=+& 7EFGG G=H |
| !"#$#'*+;): | $ $3 6 | .A=+&  ?6. | 7$ $3 6 < 4 !56 0 06 . $0 .A=+& 7EFGG G=H |
| !"#$#'*+;)) | $ $ | .A=+&  ?6. | 5A 8 6. . / < 4 !56 0 0  6 . $0 .A=+& 7EFGG G=H |
| !"#$#'*+;)&!"#$#'*+;&+!"#$#'*+;&;!"#$#'*+;&(  !"#$#'*+;&9 | $ $3 6 | .A=+&  ?6. | 7$ $3 6 < 4 !56 0 06 . $0 .A=+& 7EFGG G=H |
| !"#$#'*+;&%!"#$#'*+;&' | $ $ | .A=+&  ?6. | 5A 8 6. . / < 4 !56 0 0  6 . $0 .A=+& 7EFGG G=H |
| !"#$#'*+;&:!"#$#'*+;&)!"#$#'*+;&& | $ $3 6 | .A=+&  ?6. | 7$ $3 6 < 4 !56 0 06 . $0 .A=+& 7EFGG G=H |
| !"#$#'*+(** | $ $ | .A=+&  ?6. | 5A 8 6. . / < 4 !56 0 0  6 . $0 .A=+& 7EFGG G=H |
| !"#$#'*+(*+!"#$#'*+(*;!"#$#'*+(*(!"#$#'*+(*9 !"#$#'*+(*%!"#$#'*+(*'!"#$#'*+(*: | $ $3 6 | .A=+&  ?6. | 7$ $3 6 < 4 !56 0 06 . $0 .A=+& 7EFGG G=H |
| !"#$#'*+(*)!"#$#'*+(*& | $ $ | .A=+&  ?6. | 5A 8 6. . / < 4 !56 0 0  6 . $0 .A=+& 7EFGG G=H |
| !"#$#'*+(+*!"#$#'*+(++!"#$#'*+(+;!"#$#'*+(+(  !"#$#'*+(+9 | $ $3 6 | .A=+&  ?6. | 7$ $3 6 < 4 !56 0 06 . $0 .A=+& 7EFGG G=H |
| !"#$#'*+(+% | $ $ | .A=+&  ?6. | 5A 8 6. . / < 4 !56 0 0  6 . $0 .A=+& 7EFGG G=H |
| !"#$#'*+(+'!"#$#'*+(+: | $ $3 6 | .A=+&  ?6. | 7$ $3 6 < 4 !56 0 06 . $0 .A=+& 7EFGG G=H |

!"#$#'*+(+)!"#$#'*+(+& $ $ .A=+&

?6.

5A 8 6. . / < 4 !56 0 06 . $0 .A=+& 7EFGG G=H

!"#$#'*+(;*!"#$#'*+(;+!"#$#'*+(;;

|  |  |  | .A=+& 7EFGG G=H |
| --- | --- | --- | --- |
| !"#$#'*+(;( | $ $ |  | 5A 8 6. . / < 4 !56 0 0  6 . $0 .A=+& 7EFGG G=H |
| !"#$#'*+(;9!"#$#'*+(;%!"#$#'*+(;'!"#$#'*+(;: | $ $3 6 |  | 7$ $3 6 < 4 !56 0 06 . $0 .A=+& 7EFGG G=H |
| !"#$#'*+(;) | $ $ |  | 5A 8 6. . / < 4 !56 0 0  6 . $0 .A=+& 7EFGG G=H |
| !"#$#'*+(;&!"#$#'*+((* | $ $3 6 |  | 7$ $3 6 < 4 !56 0 06 . $0 .A=+& 7EFGG G=H |
| !"#$#'*+((+!"#$#'*+((;!"#$#'*+(((!"#$#'*+((9  !"#$#'*+((%!"#$#'*+((' | $ $ |  | 5A 8 6. . / < 4 !56 0 0  6 . $0 .A=+& 7EFGG G=H |
| !"#$#'*+((:!"#$#'*+(() | $ $3 6 | .A=+&  ?6. | 7$ $3 6 < 4 !56 0 06 . $0 .A=+& 7EFGG G=H |
| !"#$#'*+((& | $ $ | .A=+&  ?6. | 5A 8 6. . / < 4 !56 0 0  6 . $0 .A=+& 7EFGG G=H |
| !"#$#'*+(9* | $ $3 6 | .A=+&  ?6. | 7$ $3 6 < 4 !56 0 06 . $0 .A=+& 7EFGG G=H |
| !"#$#'*+(9+!"#$#'*+(9; | $ $ | .A=+&  ?6. | 5A 8 6. . / < 4 !56 0 0  6 . $0 .A=+& 7EFGG G=H |
| !"#$#'*+(9( | $ $3 6 | .A=+&  ?6. | 7$ $3 6 < 4 !56 0 06 . $0 .A=+& 7EFGG G=H |
| !"#$#'*+(99 | $ $ | .A=+&  ?6. | 5A 8 6. . / < 4 !56 0 0  6 . $0 .A=+& 7EFGG G=H |
| !"#$#'*+(9%!"#$#'*+(9' | $ $3 6 | .A=+&  ?6. | 7$ $3 6 < 4 !56 0 06 . $0 .A=+& 7EFGG G=H |
| !"#$#'*+(9:!"#$#'*+(9) | $ $ | .A=+&  ?6. | 5A 8 6. . / < 4 !56 0 0  6 . $0 .A=+& 7EFGG G=H |
| !"#$#'*+(9&!"#$#'*+(%* | $ $3 6 | .A=+&  ?6. | 7$ $3 6 < 4 !56 0 06 . $0 .A=+& 7EFGG G=H |
| !"#$#'*+(%+!"#$#'*+(%;!"#$#'*+(%( | $ $ | .A=+&  ?6. | 5A 8 6. . / < 4 !56 0 0  6 . $0 .A=+& 7EFGG G=H |
| !"#$#'*+(%9 | $ $3 6 | .A=+&  ?6. | 7$ $3 6 < 4 !56 0 06 . $0 .A=+& 7EFGG G=H |
| !"#$#'*+(%%!"#$#'*+(%'!"#$#'*+(%:!"#$#'*+(%) | $ $ | .A=+&  ?6. | 5A 8 6. . / < 4 !56 0 0  6 . $0 .A=+& 7EFGG G=H |
| !"#$#'*+(%&!"#$#'*+('* | $ $3 6 | .A=+&  ?6. | 7$ $3 6 < 4 !56 0 06 . $0 .A=+& 7EFGG G=H |
| !"#$#'*+('+!"#$#'*+('; | $ $ | .A=+&  ?6. | 5A 8 6. . / < 4 !56 0 0  6 . $0 .A=+& 7EFGG G=H |
| !"#$#'*+('( | $ $3 6 | .A=+&  ?6. | 7$ $3 6 < 4 !56 0 06 . $0 .A=+& 7EFGG G=H |
| !"#$#'*+('9 | $ $ | .A=+&  ?6. | 5A 8 6. . / < 4 !56 0 0  6 . $0 .A=+& 7EFGG G=H |
| !"#$#'*+('%!"#$#'*+(':!"#$#'*+(')!"#$#'*+('&  !"#$#'*+(:*!"#$#'*+(:+ | $ $3 6 | .A=+&  ?6. | 7$ $3 6 < 4 !56 0 06 . $0 .A=+& 7EFGG G=H |
| !"#$#'*+(:; | $ $ | .A=+&  ?6. | 5A 8 6. . / < 4 !56 0 0  6 . $0 .A=+& 7EFGG G=H |
| !"#$#'*+(:( | $ $3 6 | .A=+&  ?6. | 7$ $3 6 < 4 !56 0 06 . $0 .A=+& 7EFGG G=H |
| !"#$#'*+(:9 | $ $ | .A=+&  ?6. | 5A 8 6. . / < 4 !56 0 0  6 . $0 .A=+& 7EFGG G=H |
| !"#$#'*+(:%!"#$#'*+(:' | $ $3 6 | .A=+&  ?6. | 7$ $3 6 < 4 !56 0 06 . $0 .A=+& 7EFGG G=H |
| !"#$#'*+(:: | $ $ | .A=+&  ?6. | 5A 8 6. . / < 4 !56 0 0  6 . $0 .A=+& 7EFGG G=H |
| !"#$#'*+(:)!"#$#'*+(:& | $ $3 6 | .A=+&  ?6. | 7$ $3 6 < 4 !56 0 06 . $0 .A=+& 7EFGG G=H |
| !"#$#'*+()* | $ $ | .A=+&  ?6. | 5A 8 6. . / < 4 !56 0 0  6 . $0 .A=+& 7EFGG G=H |
| !"#$#'*+()+!"#$#'*+();!"#$#'*+()( | $ $3 6 | .A=+&  ?6. | 7$ $3 6 < 4 !56 0 06 . $0 .A=+& 7EFGG G=H |
| !"#$#'*+()9 | $ $ | .A=+&  ?6. | 5A 8 6. . / < 4 !56 0 0  6 . $0 .A=+& 7EFGG G=H |
| !"#$#'*+()% | $ $3 6 | .A=+&  ?6. | 7$ $3 6 < 4 !56 0 06 . $0 .A=+& 7EFGG G=H |
| !"#$#'*+()'!"#$#'*+(): | $ $ | .A=+&  ?6. | 5A 8 6. . / < 4 !56 0 0  6 . $0 .A=+& 7EFGG G=H |
| !"#$#'*+()) | $ $3 6 | .A=+&  ?6. | 7$ $3 6 < 4 !56 0 06 . $0 .A=+& 7EFGG G=H |
| !"#$#'*+()& | $ $ | .A=+&  ?6. | 5A 8 6. . / < 4 !56 0 0  6 . $0 .A=+& 7EFGG G=H |
| !"#$#'*+(&* | $ $3 6 | .A=+&  ?6. | 7$ $3 6 < 4 !56 0 06 . $0 .A=+& 7EFGG G=H |
| !"#$#'*+(&+ | $ $ | .A=+&  ?6. | 5A 8 6. . / < 4 !56 0 0  6 . $0 .A=+& 7EFGG G=H |
| !"#$#'*+(&; | $ $3 6 | .A=+&  ?6. | 7$ $3 6 < 4 !56 0 06 . $0 .A=+& 7EFGG G=H |
| !"#$#'*+(&( | $ $ | .A=+&  ?6. | 5A 8 6. . / < 4 !56 0 0  6 . $0 .A=+& 7EFGG G=H |
| !"#$#'*+(&9!"#$#'*+(&'!"#$#'*+(&:!"#$#'*+(&) | $ $3 6 | .A=+&  ?6. | 7$ $3 6 < 4 !56 0 06 . $0 .A=+& 7EFGG G=H |
| !"#$#'*+9**!"#$#'*+9*+!"#$#'*+9*;!"#$#'*+9*( | $ $ | .A=+&  ?6. | 5A 8 6. . / < 4 !56 0 0  6 . $0 .A=+& 7EFGG G=H |
| !"#$#'*+9*9 | $ $3 6 | .A=+&  ?6. | 7$ $3 6 < 4 !56 0 06 . $0 .A=+& 7EFGG G=H |
| !"#$#'*+9*% | $ $ | .A=+&  ?6. | 5A 8 6. . / < 4 !56 0 0  6 . $0 .A=+& 7EFGG G=H |

!"#$#'*+9*' $ $3 6 .A=+&

?6.

7$ $3 6 < 4 !56 0 06 . $0

.A=+& 7EFGG G=H

!"#$#'*+%*+ $ $. .A=+&

?6.

| !"#$#'*+9*:!"#$#'*+9*) | $ $ |  | 5A 8 6. . / < 4 !56 0 0  6 . $0 .A=+& 7EFGG G=H |
| --- | --- | --- | --- |
| !"#$#'*+9*&!"#$#'*+9+* | $ $3 6 |  | 7$ $3 6 < 4 !56 0 06 . $0 .A=+& 7EFGG G=H |
| !"#$#'*+9++ | $ $ |  | 5A 8 6. . / < 4 !56 0 0  6 . $0 .A=+& 7EFGG G=H |
| !"#$#'*+9+; | $ $3 6 |  | 7$ $3 6 < 4 !56 0 06 . $0 .A=+& 7EFGG G=H |
| !"#$#'*+9+(!"#$#'*+9+9 | $ $ |  | 5A 8 6. . / < 4 !56 0 0  6 . $0 .A=+& 7EFGG G=H |
| !"#$#'*+9+% | $ $3 6 |  | 7$ $3 6 < 4 !56 0 06 . $0 .A=+& 7EFGG G=H |
| !"#$#'*+9+' | $ $ | .A=+&  ?6. | 5A 8 6. . / < 4 !56 0 0  6 . $0 .A=+& 7EFGG G=H |
| !"#$#'*+9+:!"#$#'*+9+)!"#$#'*+9+&!"#$#'*+9;* | $ $3 6 | .A=+&  ?6. | 7$ $3 6 < 4 !56 0 06 . $0 .A=+& 7EFGG G=H |
| !"#$#'*+9;+ | $ $ | .A=+&  ?6. | 5A 8 6. . / < 4 !56 0 0  6 . $0 .A=+& 7EFGG G=H |
| !"#$#'*+9;; | $ $3 6 | .A=+&  ?6. | 7$ $3 6 < 4 !56 0 06 . $0 .A=+& 7EFGG G=H |
| !"#$#'*+9;(!"#$#'*+9;9!"#$#'*+9;%!"#$#'*+9;' | $ $ | .A=+&  ?6. | 5A 8 6. . / < 4 !56 0 0  6 . $0 .A=+& 7EFGG G=H |
| !"#$#'*+9;:!"#$#'*+9;) | $ $3 6 | .A=+&  ?6. | 7$ $3 6 < 4 !56 0 06 . $0 .A=+& 7EFGG G=H |
| !"#$#'*+9;&!"#$#'*+9(* | $ $ | .A=+&  ?6. | 5A 8 6. . / < 4 !56 0 0  6 . $0 .A=+& 7EFGG G=H |
| !"#$#'*+9(+ | $ $3 6 | .A=+&  ?6. | 7$ $3 6 < 4 !56 0 06 . $0 .A=+& 7EFGG G=H |
| !"#$#'*+9(;!"#$#'*+9(( | $ $ | .A=+&  ?6. | 5A 8 6. . / < 4 !56 0 0  6 . $0 .A=+& 7EFGG G=H |
| !"#$#'*+9(9 | $ $3 6 | .A=+&  ?6. | 7$ $3 6 < 4 !56 0 06 . $0 .A=+& 7EFGG G=H |
| !"#$#'*+9(%!"#$#'*+9(' | $ $ | .A=+&  ?6. | 5A 8 6. . / < 4 !56 0 0  6 . $0 .A=+& 7EFGG G=H |
| !"#$#'*+9(:!"#$#'*+9()!"#$#'*+9(&!"#$#'*+99* !"#$#'*+99+!"#$#'*+99;!"#$#'*+99(!"#$#'*+999 | $ $3 6 | .A=+&  ?6. | 7$ $3 6 < 4 !56 0 06 . $0 .A=+& 7EFGG G=H |
| !"#$#'*+99%!"#$#'*+99' | $ $ | .A=+&  ?6. | 5A 8 6. . / < 4 !56 0 0  6 . $0 .A=+& 7EFGG G=H |
| !"#$#'*+99:!"#$#'*+99)!"#$#'*+99&!"#$#'*+9%*  !"#$#'*+9%+!"#$#'*+9%; | $ $3 6 | .A=+&  ?6. | 7$ $3 6 < 4 !56 0 06 . $0 .A=+& 7EFGG G=H |
| !"#$#'*+9%(!"#$#'*+9%9 | $ $ | .A=+&  ?6. | 5A 8 6. . / < 4 !56 0 0  6 . $0 .A=+& 7EFGG G=H |
| !"#$#'*+9%%!"#$#'*+9%'!"#$#'*+9%:!"#$#'*+9%) | $ $3 6 | .A=+&  ?6. | 7$ $3 6 < 4 !56 0 06 . $0 .A=+& 7EFGG G=H |
| !"#$#'*+9%&!"#$#'*+9'*!"#$#'*+9'+!"#$#'*+9'; | $ $ | .A=+&  ?6. | 5A 8 6. . / < 4 !56 0 0  6 . $0 .A=+& 7EFGG G=H |
| !"#$#'*+9'(!"#$#'*+9'9!"#$#'*+9'% | $ $3 6 | .A=+&  ?6. | 7$ $3 6 < 4 !56 0 06 . $0 .A=+& 7EFGG G=H |
| !"#$#'*+9''!"#$#'*+9': | $ $ | .A=+&  ?6. | 5A 8 6. . / < 4 !56 0 0  6 . $0 .A=+& 7EFGG G=H |
| !"#$#'*+9') | $ $3 6 | .A=+&  ?6. | 7$ $3 6 < 4 !56 0 06 . $0 .A=+& 7EFGG G=H |
| !"#$#'*+9'& | $ $ | .A=+&  ?6. | 5A 8 6. . / < 4 !56 0 0  6 . $0 .A=+& 7EFGG G=H |
| !"#$#'*+9:* | $ $3 6 | .A=+&  ?6. | 7$ $3 6 < 4 !56 0 06 . $0 .A=+& 7EFGG G=H |
| !"#$#'*+9:+ | $ $ | .A=+&  ?6. | 5A 8 6. . / < 4 !56 0 0  6 . $0 .A=+& 7EFGG G=H |
| !"#$#'*+9:;!"#$#'*+9:(!"#$#'*+9:9!"#$#'*+9:%  !"#$#'*+9:' | $ $3 6 | .A=+&  ?6. | 7$ $3 6 < 4 !56 0 06 . $0 .A=+& 7EFGG G=H |
| !"#$#'*+9:: | $ $ | .A=+&  ?6. | 5A 8 6. . / < 4 !56 0 0  6 . $0 .A=+& 7EFGG G=H |
| !"#$#'*+9:) | $ $3 6 | .A=+&  ?6. | 7$ $3 6 < 4 !56 0 06 . $0 .A=+& 7EFGG G=H |
| !"#$#'*+9:&!"#$#'*+9)+!"#$#'*+9);!"#$#'*+9)( | $ $ | .A=+&  ?6. | 5A 8 6. . / < 4 !56 0 0  6 . $0 .A=+& 7EFGG G=H |
| !"#$#'*+9)% | $ $3 6 | .A=+&  ?6. | 7$ $3 6 < 4 !56 0 06 . $0 .A=+& 7EFGG G=H |
| !"#$#'*+9)' | $ $ | .A=+&  ?6. | 5A 8 6. . / < 4 !56 0 0  6 . $0 .A=+& 7EFGG G=H |
| !"#$#'*+9): | $ $3 6 | .A=+&  ?6. | 7$ $3 6 < 4 !56 0 06 . $0 .A=+& 7EFGG G=H |
| !"#$#'*+9))!"#$#'*+9)& | $ $ | .A=+&  ?6. | 5A 8 6. . / < 4 !56 0 0  6 . $0 .A=+& 7EFGG G=H |
| !"#$#'*+9&*!"#$#'*+9&+!"#$#'*+9&; | $ $3 6 | .A=+&  ?6. | 7$ $3 6 < 4 !56 0 06 . $0 .A=+& 7EFGG G=H |
| !"#$#'*+9&( | $ $. | .A=+&  ?6. | 457$ $. < 4 !56 0 06 . $0 .A=+& 7EFGG G=H |
| !"#$#'*+9&9 | $ $3 6 | .A=+&  ?6. | 7$ $3 6 < 4 !56 0 06 . $0 .A=+& 7EFGG G=H |
| !"#$#'*+9&% | $ $. | .A=+&  ?6. | 457$ $. < 4 !56 0 06 . $0 .A=+& 7EFGG G=H |
| !"#$#'*+9&'!"#$#'*+9&:!"#$#'*+9&) | $ $3 6 | .A=+&  ?6. | 7$ $3 6 < 4 !56 0 06 . $0 .A=+& 7EFGG G=H |
| !"#$#'*+9&& | $ $. | .A=+&  ?6. | 457$ $. < 4 !56 0 06 . $0 .A=+& 7EFGG G=H |
| !"#$#'*+%** | $ $3 6 | .A=+&  ?6. | 7$ $3 6 < 4 !56 0 06 . $0 .A=+& 7EFGG G=H |

457$ $. < 4 !56 0 06 . $0

.A=+& 7EFGG G=H

| !"#$#'*+%*;!"#$#'*+%*(!"#$#'*+%*9!"#$#'*+%*%  .A=+& 7EFGG G=H   | !"#$#'*+%*' |  |  | .A=+& 7EFGG G=H | | --- | --- | --- | --- | | !"#$#'*+%*)!"#$#'*+%*&!"#$#'*+%+*!"#$#'*+%++ !"#$#'*+%+;!"#$#'*+%+(!"#$#'*+%+9 |  |  | 7$ $3 6 < 4 !56 0 06 . $0 .A=+& 7EFGG G=H | | !"#$#'*+%+% |  |  | 457$ $. < 4 !56 0 06 . $0 .A=+& 7EFGG G=H | | !"#$#'*+%+'!"#$#'*+%+:!"#$#'*+%+)!"#$#'*+%+&  !"#$#'*+%;*!"#$#'*+%;+ |  |  | 7$ $3 6 < 4 !56 0 06 . $0 .A=+& 7EFGG G=H | | !"#$#'*+%;; |  |  | 457$ $. < 4 !56 0 06 . $0 .A=+& 7EFGG G=H | | !"#$#'*+%;(!"#$#'*+%;9!"#$#'*+%;%!"#$#'*+%;' !"#$#'*+%;:!"#$#'*+%;)!"#$#'*+%;& |  | .A=+&  ?6. | 7$ $3 6 < 4 !56 0 06 . $0 .A=+& 7EFGG G=H | | !"#$#'*+%(* | $ $. | .A=+&  ?6. | 457$ $. < 4 !56 0 06 . $0 .A=+& 7EFGG G=H | | !"#$#'*+%(+!"#$#'*+%(;!"#$#'*+%((!"#$#'*+%(9  !"#$#'*+%(%!"#$#'*+%('!"#$#'*+%(:!"#$#'*+%() !"#$#'*+%(&!"#$#'*+%9* | $ $3 6 | .A=+&  ?6. | 7$ $3 6 < 4 !56 0 06 . $0 .A=+& 7EFGG G=H | | !"#$#'*+%9+ | $ $. | .A=+&  ?6. | 457$ $. < 4 !56 0 06 . $0 .A=+& 7EFGG G=H | | !"#$#'*+%9;!"#$#'*+%9(!"#$#'*+%99!"#$#'*+%9%  !"#$#'*+%9' | $ $3 6 | .A=+&  ?6. | 7$ $3 6 < 4 !56 0 06 . $0 .A=+& 7EFGG G=H | | !"#$#'*+%9&!"#$#'*+%%* | $ $. | .A=+&  ?6. | 457$ $. < 4 !56 0 06 . $0 .A=+& 7EFGG G=H | | !"#$#'*+%%+!"#$#'*+%%;!"#$#'*+%%(!"#$#'*+%%9 | $ $3 6 | .A=+&  ?6. | 7$ $3 6 < 4 !56 0 06 . $0 .A=+& 7EFGG G=H | | !"#$#'*+%%% | $ $. | .A=+&  ?6. | 457$ $. < 4 !56 0 06 . $0 .A=+& 7EFGG G=H | | !"#$#'*+%%'!"#$#'*+%%:!"#$#'*+%%)!"#$#'*+%%&!"#$#'*+%'*!"#$#'*+%'+!"#$#'*+%';!"#$#'*+%'(!"#$#'*+%'9!"#$#'*+%'%!"#$#'*+%'' | | | |  | $ $3 6 | .A=+&  ?6. | 7$ $3 6 < 4 !56 0 06 . $0 .A=+& 7EFGG G=H | | !"#$#'*+%': | $ $. | .A=+&  ?6. | 457$ $. < 4 !56 0 06 . $0 .A=+& 7EFGG G=H | | !"#$#'*+%') | $ $3 6 | .A=+&  ?6. | 7$ $3 6 < 4 !56 0 06 . $0 .A=+& 7EFGG G=H | | !"#$#'*+%'& | $ $. | .A=+&  ?6. | 457$ $. < 4 !56 0 06 . $0 .A=+& 7EFGG G=H | | !"#$#'*+%:* | $ $3 6 | .A=+&  ?6. | 7$ $3 6 < 4 !56 0 06 . $0 .A=+& 7EFGG G=H | | !"#$#'*+%:+ | $ $. | .A=+&  ?6. | 457$ $. < 4 !56 0 06 . $0 .A=+& 7EFGG G=H | | !"#$#'*+%:;!"#$#'*+%:(!"#$#'*+%:9!"#$#'*+%:% !"#$#'*+%:'!"#$#'*+%:: | $ $3 6 | .A=+&  ?6. | 7$ $3 6 < 4 !56 0 06 . $0 .A=+& 7EFGG G=H | | !"#$#'*+%:)!"#$#'*+%:&!"#$#'*+%)* | $ $. | .A=+&  ?6. | 457$ $. < 4 !56 0 06 . $0 .A=+& 7EFGG G=H | | !"#$#'*+%)+!"#$#'*+%);!"#$#'*+%)(!"#$#'*+%)9 | $ $3 6 | .A=+&  ?6. | 7$ $3 6 < 4 !56 0 06 . $0 .A=+& 7EFGG G=H | | !"#$#'*+%)% | $ $. | .A=+&  ?6. | 457$ $. < 4 !56 0 06 . $0 .A=+& 7EFGG G=H | | !"#$#'*+%)'!"#$#'*+%): | $ $3 6 | .A=+&  ?6. | 7$ $3 6 < 4 !56 0 06 . $0 .A=+& 7EFGG G=H | | !"#$#'*+%))!"#$#'*+%)&!"#$#'*+%&+ | $ $. | .A=+&  ?6. | 457$ $. < 4 !56 0 06 . $0 .A=+& 7EFGG G=H | | !"#$#'*+%&;!"#$#'*+%&( | $ $3 6 | .A=+&  ?6. | 7$ $3 6 < 4 !56 0 06 . $0 .A=+& 7EFGG G=H | | !"#$#'*+%&9 | $ $. | .A=+&  ?6. | 457$ $. < 4 !56 0 06 . $0 .A=+& 7EFGG G=H | | !"#$#'*+%&%!"#$#'*+%&'!"#$#'*+%&:!"#$#'*+%&)  !"#$#'*+%&&!"#$#'*+'**!"#$#'*+'*+!"#$#'*+'*; !"#$#'*+'*( | $ $3 6 | .A=+&  ?6. | 7$ $3 6 < 4 !56 0 06 . $0 .A=+& 7EFGG G=H | | !"#$#'*+'*9 | $ $. | .A=+&  ?6. | 457$ $. < 4 !56 0 06 . $0 .A=+& 7EFGG G=H | | !"#$#'*+'*% | $ $3 6 | .A=+&  ?6. | 7$ $3 6 < 4 !56 0 06 . $0 .A=+& 7EFGG G=H | | !"#$#'*+'*' | $ $. | .A=+&  ?6. | 457$ $. < 4 !56 0 06 . $0 .A=+& 7EFGG G=H | | !"#$#'*+'*:!"#$#'*+'*)!"#$#'*+'*&!"#$#'*+'+* | $ $3 6 | .A=+&  ?6. | 7$ $3 6 < 4 !56 0 06 . $0 .A=+& 7EFGG G=H | | !"#$#'*+'++ | $ $. | .A=+&  ?6. | 457$ $. < 4 !56 0 06 . $0 .A=+& 7EFGG G=H | | !"#$#'*+'+;!"#$#'*+'+(!"#$#'*+'+9 | $ $3 6 | .A=+&  ?6. | 7$ $3 6 < 4 !56 0 06 . $0 .A=+& 7EFGG G=H | | !"#$#'*+'+% | $ $. | .A=+&  ?6. | 457$ $. < 4 !56 0 06 . $0 .A=+& 7EFGG G=H | | !"#$#'*+'+' | $ $3 6 | .A=+&  ?6. | 7$ $3 6 < 4 !56 0 06 . $0 .A=+& 7EFGG G=H | | !"#$#'*+'+:!"#$#'*+'+)!"#$#'*+'+& | $ $. | .A=+&  ?6. | 457$ $. < 4 !56 0 06 . $0 .A=+& 7EFGG G=H | | !"#$#'*+';*!"#$#'*+';+ | $ $3 6 | .A=+&  ?6. | 7$ $3 6 < 4 !56 0 06 . $0 .A=+& 7EFGG G=H | | !"#$#'*+';; | $ $. | .A=+&  ?6. | 457$ $. < 4 !56 0 06 . $0 .A=+& 7EFGG G=H | | !"#$#'*+';( | $ $3 6 | .A=+&  ?6. | 7$ $3 6 < 4 !56 0 06 . $0 .A=+& 7EFGG G=H | | !"#$#'*+';9 | $ $. | .A=+&  ?6. | 457$ $. < 4 !56 0 06 . $0 .A=+& 7EFGG G=H | | !"#$#'*+';%!"#$#'*+';' | $ $3 6 | .A=+&  ?6. | 7$ $3 6 < 4 !56 0 06 . $0 .A=+& 7EFGG G=H | |
| --- | --- | --- | --- | --- | --- | --- | --- | --- | --- | --- | --- | --- | --- | --- | --- | --- | --- | --- | --- | --- | --- | --- | --- | --- | --- | --- | --- | --- | --- | --- | --- | --- | --- | --- | --- | --- | --- | --- | --- | --- | --- | --- | --- | --- | --- | --- | --- | --- | --- | --- | --- | --- | --- | --- | --- | --- | --- | --- | --- | --- | --- | --- | --- | --- | --- | --- | --- | --- | --- | --- | --- | --- | --- | --- | --- | --- | --- | --- | --- | --- | --- | --- | --- | --- | --- | --- | --- | --- | --- | --- | --- | --- | --- | --- | --- | --- | --- | --- | --- | --- | --- | --- | --- | --- | --- | --- | --- | --- | --- | --- | --- | --- | --- | --- | --- | --- | --- | --- | --- | --- | --- | --- | --- | --- | --- | --- | --- | --- | --- | --- | --- | --- | --- | --- | --- | --- | --- | --- | --- | --- | --- | --- | --- | --- | --- | --- | --- | --- | --- | --- | --- | --- | --- | --- | --- | --- | --- | --- | --- | --- | --- | --- | --- | --- | --- | --- | --- | --- | --- | --- | --- |

!"#$#'*+';:!"#$#'*+';) $ $. .A=+&

?6.

457$ $. < 4 !56 0 06 . $0

.A=+& 7EFGG G=H

| !"#$#'*+';&!"#$#'*+'(*!"#$#'*+'(+!"#$#'*+'(; |  |  |  |
| --- | --- | --- | --- |
| !"#$#'*+'((  !"#$#'*+'(9 |  |  | .A=+& 7EFGG G=H  .A=+& 7EFGG G=H |
| !"#$#'*+'(%!"#$#'*+'('!"#$#'*+'(:!"#$#'*+'() |  |  | 7$ $3 6 < 4 !56 0 06 . $0 .A=+& 7EFGG G=H |
| !"#$#'*+'(& |  |  | 457$ $. < 4 !56 0 06 . $0 .A=+& 7EFGG G=H |
| !"#$#'*+'9*!"#$#'*+'9;!"#$#'*+'9(!"#$#'*+'99 !"#$#'*+'9%!"#$#'*+'9'!"#$#'*+'9: |  |  | 7$ $3 6 < 4 !56 0 06 . $0 .A=+& 7EFGG G=H |
| !"#$#'*+'9) |  |  | 457$ $. < 4 !56 0 06 . $0 .A=+& 7EFGG G=H |
| !"#$#'*+'9& |  | .A=+&  ?6. | 7$ $3 6 < 4 !56 0 06 . $0 .A=+& 7EFGG G=H |
| !"#$#'*+'%* | $ $. | .A=+&  ?6. | 457$ $. < 4 !56 0 06 . $0 .A=+& 7EFGG G=H |
| !"#$#'*+'%+!"#$#'*+'%(!"#$#'*+'%9!"#$#'*+'%%  !"#$#'*+'%' | $ $3 6 | .A=+&  ?6. | 7$ $3 6 < 4 !56 0 06 . $0 .A=+& 7EFGG G=H |
| !"#$#'*+'%: | $ $. | .A=+&  ?6. | 457$ $. < 4 !56 0 06 . $0 .A=+& 7EFGG G=H |
| !"#$#'*+'%)!"#$#'*+'%&!"#$#'*+''*!"#$#'*+''+  !"#$#'*+''; | $ $3 6 | .A=+&  ?6. | 7$ $3 6 < 4 !56 0 06 . $0 .A=+& 7EFGG G=H |
| !"#$#'*+''(!"#$#'*+''9 | $ $. | .A=+&  ?6. | 457$ $. < 4 !56 0 06 . $0 .A=+& 7EFGG G=H |
| !"#$#'*+''% | $ $3 6 | .A=+&  ?6. | 7$ $3 6 < 4 !56 0 06 . $0 .A=+& 7EFGG G=H |
| !"#$#'*+'''!"#$#'*+'') | $ $. | .A=+&  ?6. | 457$ $. < 4 !56 0 06 . $0 .A=+& 7EFGG G=H |
| !"#$#'*+''&!"#$#'*+':*!"#$#'*+':+!"#$#'*+':; | $ $3 6 | .A=+&  ?6. | 7$ $3 6 < 4 !56 0 06 . $0 .A=+& 7EFGG G=H |
| !"#$#'*+':( | $ $. | .A=+&  ?6. | 457$ $. < 4 !56 0 06 . $0 .A=+& 7EFGG G=H |
| !"#$#'*+':9!"#$#'*+':%!"#$#'*+':'!"#$#'*+':: !"#$#'*+':)!"#$#'*+':&!"#$#'*+')*!"#$#'*+')+ | $ $3 6 | .A=+&  ?6. | 7$ $3 6 < 4 !56 0 06 . $0 .A=+& 7EFGG G=H |
| !"#$#'*+'); | $ $. | .A=+&  ?6. | 457$ $. < 4 !56 0 06 . $0 .A=+& 7EFGG G=H |
| !"#$#'*+')(!"#$#'*+')9 | $ $3 6 | .A=+&  ?6. | 7$ $3 6 < 4 !56 0 06 . $0 .A=+& 7EFGG G=H |
| !"#$#'*+')% | $ $. | .A=+&  ?6. | 457$ $. < 4 !56 0 06 . $0 .A=+& 7EFGG G=H |
| !"#$#'*+')'!"#$#'*+'):!"#$#'*+'))!"#$#'*+')& | $ $3 6 | .A=+&  ?6. | 7$ $3 6 < 4 !56 0 06 . $0 .A=+& 7EFGG G=H |
| !"#$#'*+'&*!"#$#'*+'&+ | $ $. | .A=+&  ?6. | 457$ $. < 4 !56 0 06 . $0 .A=+& 7EFGG G=H |
| !"#$#'*+'&;!"#$#'*+'&(!"#$#'*+'&9!"#$#'*+'&% !"#$#'*+'&'!"#$#'*+'&:!"#$#'*+'&)!"#$#'*+'&& | $ $3 6 | .A=+&  ?6. | 7$ $3 6 < 4 !56 0 06 . $0 .A=+& 7EFGG G=H |
| !"#$#'*+:** | $ $. | .A=+&  ?6. | 457$ $. < 4 !56 0 06 . $0 .A=+& 7EFGG G=H |
| !"#$#'*+:*+!"#$#'*+:*;!"#$#'*+:*(!"#$#'*+:*% !"#$#'*+:*'!"#$#'*+:*:!"#$#'*+:*) | $ $3 6 | .A=+&  ?6. | 7$ $3 6 < 4 !56 0 06 . $0 .A=+& 7EFGG G=H |
| !"#$#'*+:*& | $ $. | .A=+&  ?6. | 457$ $. < 4 !56 0 06 . $0 .A=+& 7EFGG G=H |
| !"#$#'*+:+*!"#$#'*+:++!"#$#'*+:+;!"#$#'*+:+(  !"#$#'*+:+9 | $ $3 6 | .A=+&  ?6. | 7$ $3 6 < 4 !56 0 06 . $0 .A=+& 7EFGG G=H |
| !"#$#'*+:+% | $ $. | .A=+&  ?6. | 457$ $. < 4 !56 0 06 . $0 .A=+& 7EFGG G=H |
| !"#$#'*+:+'!"#$#'*+:+:!"#$#'*+:+)!"#$#'*+:+&!"#$#'*+:;*!"#$#'*+:;;!"#$#'*+:;(!"#$#'*+:;9!"#$#'*+:;%!"#$#'*+:;'!"#$#'*+:;: | | |
| $ $3 6 .A=+&  ?6. | | | 7$ $3 6 < 4 !56 0 06 . $0 .A=+& 7EFGG G=H |
| !"#$#'*+:;) $ $. .A=+&  ?6. | | | 457$ $. < 4 !56 0 06 . $0 .A=+& 7EFGG G=H |
| !"#$#'*+:;& $ $3 6 .A=+&  ?6. | | | 7$ $3 6 < 4 !56 0 06 . $0 .A=+& 7EFGG G=H |
| !"#$#'*+:(* $ $. .A=+&  ?6. | | | 457$ $. < 4 !56 0 06 . $0 .A=+& 7EFGG G=H |
| !"#$#'*+:(+!"#$#'*+:(; $ $3 6 .A=+&  ?6. | | | 7$ $3 6 < 4 !56 0 06 . $0 .A=+& 7EFGG G=H |
| !"#$#'*+:(( $ $. .A=+&  ?6. | | | 457$ $. < 4 !56 0 06 . $0 .A=+& 7EFGG G=H |
| !"#$#'*+:(9!"#$#'*+:(%!"#$#'*+:('!"#$#'*+:(: $ $3 6 .A=+&  !"#$#'*+:()!"#$#'*+:(& ?6. | | | 7$ $3 6 < 4 !56 0 06 . $0 .A=+& 7EFGG G=H |
| !"#$#'*+:9* $ $. .A=+&  ?6. | | | 457$ $. < 4 !56 0 06 . $0 .A=+& 7EFGG G=H |
| !"#$#'*+:9+!"#$#'*+:9;!"#$#'*+:9(!"#$#'*+:99 $ $3 6 .A=+&  !"#$#'*+:9%!"#$#'*+:9' ?6. | | | 7$ $3 6 < 4 !56 0 06 . $0 .A=+& 7EFGG G=H |
| !"#$#'*+:9:!"#$#'*+:9) $ $. .A=+&  ?6. | | | 457$ $. < 4 !56 0 06 . $0 .A=+& 7EFGG G=H |

!"#$#'*+:9&!"#$#'*+:%*!"#$#'*+:%+!"#$#'*+:%;!"#$#'*+:%(!"#$#'*+:%9!"#$#'*+:%%!"#$#'*+:%'!"#$#'*+:%:!"#$#'*+:%)!"#$#'*+:%&!"#$#'*+:'*!"#$#'*+:'+!"#$#'*+:';!"#$#'*+:'(

|  |  | $ $3 6 | .A=+&  ?6. | 7$ $3 6 < 4 !56 0 06 . $0 .A=+& 7EFGG G=H |
| --- | --- | --- | --- | --- |
|  | !"#$#'*+:'9!"#$#'*+:'' | $ $. | .A=+&  ?6. | 457$ $. < 4 !56 0 06 . $0 .A=+& 7EFGG G=H |
|  | !"#$#'*+:': | $ $3 6 | .A=+&  ?6. | 7$ $3 6 < 4 !56 0 06 . $0 .A=+& 7EFGG G=H |
|  | !"#$#'*+:') | $ $. | .A=+&  ?6. | 457$ $. < 4 !56 0 06 . $0 .A=+& 7EFGG G=H |
|  | !"#$#'*+:'&!"#$#'*+::* | $ $3 6 | .A=+&  ?6. | 7$ $3 6 < 4 !56 0 06 . $0 .A=+& 7EFGG G=H |
|  | !"#$#'*+::+ | $ $. | .A=+&  ?6. | 457$ $. < 4 !56 0 06 . $0 .A=+& 7EFGG G=H |
| !"#$#'*+::;!"#$#'*+::(!"#$#'*+::9!"#$#'*+::% | | |  |  |
| !"#$#'*+::'!"#$#'*+:::  !"#$#'*+::) | | |  | .A=+& 7EFGG G=H  .A=+& 7EFGG G=H |
| !"#$#'*+::&!"#$#'*+:)*!"#$#'*+:)+!"#$#'*+:); | | |  | 7$ $3 6 < 4 !56 0 06 . $0 .A=+& 7EFGG G=H |
| !"#$#'*+:)( | | |  | 457$ $. < 4 !56 0 06 . $0 .A=+& 7EFGG G=H |
| !"#$#'*+:)9 | | |  | 7$ $3 6 < 4 !56 0 06 . $0 .A=+& 7EFGG G=H |
| !"#$#'*+:)% | | |  | 457$ $. < 4 !56 0 06 . $0 .A=+& 7EFGG G=H |
| !"#$#'*+:)' | | | .A=+& | 7$ $3 6 < 4 !56 0 06 . $0 |

?6. .A=+& 7EFGG G=H

!"#$#'*+:):!"#$#'*+:))!"#$#'*+:)&!"#$#'*+:&*!"#$#'*+:&+!"#$#'*+:&;!"#$#'*+:&(!"#$#'*+:&9!"#$#'*+:&%!"#$#'*+:&'!"#$#'*+:&)!"#$#'*+:&&!"#$#'*+)**!"#$#'*+)*+!"#$#'*+)*;!"#$#'*+)*(!"#$#'*+)*9!"#$#'*+)*%!"#$#'*+)*'!"#$#'*+)*:!"#$#'*+)*)!"#$#'*+)*&!"#$#'*+)+*!"#$#'*+)++ !"#$#'*+)+;!"#$#'*+)+(!"#$#'*+)+9!"#$#'*+)+%!"#$#'*+)+'!"#$#'*+)+:!"#$#'*+)+)!"#$#'*+);*!"#$#'*+);+!"#$#'*+);;!"#$#'*+);(!"#$#'*+);9!"#$#'*+);%!"#$#'*+);'!"#$#'*+);:!"#$#'*+);)!"#$#'*+);&!"#$#'*+)(*!"#$#'*+)(+!"#$#'*+)(;!"#$#'*+)((!"#$#'*+)(%!"#$#'*+)('!"#$#'*+)(: !"#$#'*+)()!"#$#'*+)(&!"#$#'*+)9*!"#$#'*+)9+!"#$#'*+)9;!"#$#'*+)9(!"#$#'*+)99!"#$#'*+)9%!"#$#'*+)9'!"#$#'*+)9:!"#$#'*+)9)!"#$#'*+)9&!"#$#'*+)%*!"#$#'*+)%+!"#$#'*+)%;!"#$#'*+)%(!"#$#'*+)%9!"#$#'*+)%%!"#$#'*+)%'!"#$#'*+)%:!"#$#'*+)%)!"#$#'*+)%&!"#$#'*+)'*!"#$#'*+)'+

!"#$#'*+)';!"#$#'*+)'(!"#$#'*+)'%!"#$#'*+)''!"#$#'*+)':!"#$#'*+)')!"#$#'*+)'&!"#$#'*+):*!"#$#'*+):+!"#$#'*+):;!"#$#'*+):(!"#$#'*+):9!"#$#'*+):%!"#$#'*+):'!"#$#'*+)::!"#$#'*+):)!"#$#'*+):&!"#$#'*+))*!"#$#'*+))+!"#$#'*+));!"#$#'*+))(!"#$#'*+))9!"#$#'*+))%!"#$#'*+))'

!"#$#'*+)):!"#$#'*+)))!"#$#'*+))&!"#$#'*+)&*!"#$#'*+)&+!"#$#'*+)&;!"#$#'*+)&(!"#$#'*+)&9!"#$#'*+)&%!"#$#'*+)&'!"#$#'*+)&:!"#$#'*+)&)!"#$#'*+)&&!"#$#'*+&**!"#$#'*+&*+!"#$#'*+&*;!"#$#'*+&*(!"#$#'*+&*9!"#$#'*+&*%!"#$#'*+&*'!"#$#'*+&*:!"#$#'*+&*)!"#$#'*+&*&!"#$#'*+&+*

!"#$#'*+&++!"#$#'*+&+;!"#$#'*+&+(!"#$#'*+&+9!"#$#'*+&+%!"#$#'*+&+'!"#$#'*+&+:!"#$#'*+&+)!"#$#'*+&+&!"#$#'*+&;*!"#$#'*+&;+!"#$#'*+&;;!"#$#'*+&;(!"#$#'*+&;%!"#$#'*+&;'!"#$#'*+&;:!"#$#'*+&;)!"#$#'*+&;&!"#$#'*+&(*!"#$#'*+&(+!"#$#'*+&(;!"#$#'*+&((!"#$#'*+&(9!"#$#'*+&(%

!"#$#'*+&('!"#$#'*+&(:!"#$#'*+&()!"#$#'*+&(&!"#$#'*+&9*!"#$#'*+&9+!"#$#'*+&9;!"#$#'*+&9(!"#$#'*+&99!"#$#'*+&9%!"#$#'*+&9'!"#$#'*+&9:!"#$#'*+&9)!"#$#'*+&9&!"#$#'*+&%+!"#$#'*+&%;!"#$#'*+&%(!"#$#'*+&%9!"#$#'*+&%%!"#$#'*+&%'!"#$#'*+&%:!"#$#'*+&%)!"#$#'*+&%&!"#$#'*+&'*

!"#$#'*+&'+!"#$#'*+&';!"#$#'*+&'(!"#$#'*+&'9!"#$#'*+&'%!"#$#'*+&''!"#$#'*+&':!"#$#'*+&')!"#$#'*+&'&!"#$#'*+&:*!"#$#'*+&:+!"#$#'*+&:;!"#$#'*+&:(!"#$#'*+&:9!"#$#'*+&:%!"#$#'*+&:'!"#$#'*+&::!"#$#'*+&:)!"#$#'*+&:&!"#$#'*+&)*!"#$#'*+&)+!"#$#'*+&);!"#$#'*+&)(!"#$#'*+&)9 !"#$#'*+&)%!"#$#'*+&)'!"#$#'*+&):!"#$#'*+&))!"#$#'*+&)&!"#$#'*+&&*!"#$#'*+&&+!"#$#'*+&&;!"#$#'*+&&9!"#$#'*+&&%!"#$#'*+&&'!"#$#'*+&&:!"#$#'*+&&)!"#$#'*+&&&!"#$#'*;**+!"#$#'*;**;!"#$#'*;**(!"#$#'*;**9!"#$#'*;**%!"#$#'*;**:!"#$#'*;**)!"#$#'*;**&!"#$#'*;*+*!"#$#'*;*++ !"#$#'*;*+;!"#$#'*;*+(!"#$#'*;*+9!"#$#'*;*+'!"#$#'*;*+:!"#$#'*;*+)!"#$#'*;*+&!"#$#'*;*;*!"#$#'*;*;+!"#$#'*;*;;!"#$#'*;*;(!"#$#'*;*;9!"#$#'*;*;%!"#$#'*;*;'

|  | $ $ | .A=+&  ?6. | 5A 8 6. . / < 4 !56 0 0  6 . $0 .A=+& 7EFGG G=H |
| --- | --- | --- | --- |
| !"#$#'*;*;:!"#$#'*;*;)!"#$#'*;*;& | $ $3 6 | .A=+& | 7$ $3 6 < 4 !56 0 06 . $0 |

?6. .A=+& 7EFGG G=H

!"#$#'*;*(*!"#$#'*;*(+!"#$#'*;*(;!"#$#'*;*((!"#$#'*;*(9!"#$#'*;*(%!"#$#'*;*('!"#$#'*;*(:!"#$#'*;*()!"#$#'*;*(&!"#$#'*;*9*!"#$#'*;*9+!"#$#'*;*9;!"#$#'*;*9(!"#$#'*;*99!"#$#'*;*9%!"#$#'*;*9'!"#$#'*;*9:!"#$#'*;*9)!"#$#'*;*9&!"#$#'*;*%*!"#$#'*;*%+!"#$#'*;*%;!"#$#'*;*%(

!"#$#'*;*%9!"#$#'*;*%%!"#$#'*;*%'!"#$#'*;*%:!"#$#'*;*%)!"#$#'*;*%&!"#$#'*;*'*!"#$#'*;*'+!"#$#'*;*';!"#$#'*;*'(!"#$#'*;*'9!"#$#'*;*'%!"#$#'*;*''!"#$#'*;*':!"#$#'*;*')!"#$#'*;*'&!"#$#'*;*:*!"#$#'*;*:+!"#$#'*;*:;!"#$#'*;*:(!"#$#'*;*:9!"#$#'*;*:%!"#$#'*;*:'!"#$#'*;*:)

!"#$#'*;*:&!"#$#'*;*)*!"#$#'*;*);!"#$#'*;*)9!"#$#'*;*)%!"#$#'*;*)'!"#$#'*;*):!"#$#'*;*))!"#$#'*;*)&!"#$#'*;*&;!"#$#'*;*&(!"#$#'*;*&9!"#$#'*;*&%!"#$#'*;*&'!"#$#'*;*&:!"#$#'*;*&)!"#$#'*;*&&!"#$#'*;+**!"#$#'*;+*+!"#$#'*;+*9!"#$#'*;+*%!"#$#'*;+*:!"#$#'*;+*)!"#$#'*;++*

!"#$#'*;+++!"#$#'*;++;!"#$#'*;++(!"#$#'*;++9!"#$#'*;++%!"#$#'*;++'!"#$#'*;++:!"#$#'*;++)!"#$#'*;++&!"#$#'*;+;*!"#$#'*;+;+!"#$#'*;+;(!"#$#'*;+;9!"#$#'*;+;%!"#$#'*;+;'!"#$#'*;+;:!"#$#'*;+;)!"#$#'*;+;&!"#$#'*;+(*!"#$#'*;+(+!"#$#'*;+(;!"#$#'*;+((!"#$#'*;+(9!"#$#'*;+(%

!"#$#'*;+('!"#$#'*;+(:!"#$#'*;+()!"#$#'*;+(&!"#$#'*;+9*!"#$#'*;+9+!"#$#'*;+9;!"#$#'*;+9(!"#$#'*;+99

|  | $ $ | .A=+&  ?6. | 5A 8 6. . / < 4 !56 0 0  6 . $0 .A=+& 7EFGG G=H |
| --- | --- | --- | --- |
| !"#$#'*;+9%!"#$#'*;+9'!"#$#'*;+9:!"#$#'*;+9)  !"#$#'*;+9&!"#$#'*;+%*!"#$#'*;+%+!"#$#'*;+%; !"#$#'*;+%( | $ $3 6 | .A=+&  ?6. | 7$ $3 6 < 4 !56 0 06 . $0 .A=+& 7EFGG G=H |
| !"#$#'*;+%9 | $ $. | .A=+&  ?6. | 457$ $. < 4 !56 0 06 . $0 .A=+& 7EFGG G=H |
| !"#$#'*;+%% | $ $ " | .A=+&  ?6. | 0B 35 7$ $ " < 4 !56 0 06 .  $0 .A=+& 7EFGG G=H |
| !"#$#'*;+%'!"#$#'*;+%: | $ $ | .A=+&  ?6. | 5A 8 6. . / < 4 !56 0 0  6 . $0 .A=+& 7EFGG G=H |
| !"#$#'*;+%)!"#$#'*;+%&!"#$#'*;+'+ | $ $ " | .A=+&  ?6. | 0B 35 7$ $ " < 4 !56 0 06 .  $0 .A=+& 7EFGG G=H |

!"#$#'*;+';!"#$#'*;+'(!"#$#'*;+'9!"#$#'*;+'%!"#$#'*;+''!"#$#'*;+':!"#$#'*;+')!"#$#'*;+'&!"#$#'*;+:*!"#$#'*;+:+!"#$#'*;+:;!"#$#'*;+:(!"#$#'*;+:9!"#$#'*;+:%!"#$#'*;+:'!"#$#'*;+::!"#$#'*;+:)!"#$#'*;+:&!"#$#'*;+)*!"#$#'*;+)+!"#$#'*;+);!"#$#'*;+)(!"#$#'*;+)9!"#$#'*;+)% !"#$#'*;+)'!"#$#'*;+):!"#$#'*;+))!"#$#'*;+)&!"#$#'*;+&*!"#$#'*;+&+!"#$#'*;+&;!"#$#'*;+&(!"#$#'*;+&9!"#$#'*;+&%!"#$#'*;+&'!"#$#'*;+&:!"#$#'*;+&)!"#$#'*;+&&!"#$#'*;;**!"#$#'*;;*+!"#$#'*;;*;!"#$#'*;;*(!"#$#'*;;*9!"#$#'*;;*%!"#$#'*;;*'!"#$#'*;;*:

-3<5 $ -3<5 $ ! 0=,C! 3

!"#$#'*;;*)!"#$#'*;;*& -3<5 $ $ - $1 .=.$3 . 633 7 6 05 3 !" .

!"#$#'*;;+*!"#$#'*;;++!"#$#'*;;+;!"#$#'*;;+(!"#$#'*;;+'!"#$#'*;;+:!"#$#'*;;+)!"#$#'*;;+&!"#$#'*;;;*!"#$#'*;;;(!"#$#'*;;;9!"#$#'*;;;%!"#$#'*;;;'!"#$#'*;;;)!"#$#'*;;(+!"#$#'*;;((!"#$#'*;;(9!"#$#'*;;(%!"#$#'*;;(:!"#$#'*;;()!"#$#'*;;(&!"#$#'*;;9*!"#$#'*;;9+!"#$#'*;;9; !"#$#'*;;9(!"#$#'*;;99!"#$#'*;;9%!"#$#'*;;9'!"#$#'*;;9&!"#$#'*;;%(!"#$#'*;;%%

7I5 7I5 47 10>3 4" 36. 4 $6 3 " /

!"#$#'*;;%)!"#$#'*;;%&!"#$#'*;;'*!"#$#'*;;'+!"#$#'*;;';!"#$#'*;;'(!"#$#'*;;'9!"#$#'*;;'%!"#$#'*;;''!"#$#'*;;':!"#$#'*;;')!"#$#'*;;'&!"#$#'*;;:*!"#$#'*;;:+!"#$#'*;;:;!"#$#'*;;:(!"#$#'*;;:9!"#$#'*;;:%!"#$#'*;;:'!"#$#'*;;::

3"5$ 3"5$ 3 5 $

!"#$#'*;;:)!"#$#'*;;:&!"#$#'*;;)*!"#$#'*;;)+ -3<5 $ $ - $1 .=.$3 . 633 7 6 05 3 !" .

!"#$#'*;;);!"#$#'*;;)(!"#$#'*;;)9!"#$#'*;;)%!"#$#'*;;)'!"#$#'*;;):!"#$#'*;;))!"#$#'*;;)&!"#$#'*;;&*!"#$#'*;;&+!"#$#'*;;&;!"#$#'*;;&(!"#$#'*;;&9!"#$#'*;;&%!"#$#'*;;&'!"#$#'*;;&:!"#$#'*;;&)!"#$#'*;;&&!"#$#'*;(**!"#$#'*;(*+!"#$#'*;(*;!"#$#'*;(*(

! 6 1 J$ / 1 ? 171 3 5 0K6 $=06 . </3 2A

3 5

!"#$#'*;(*9 > 3// ? A L >3 3 /A$. ,

!"#$#'*;(*'!"#$#'*;(*:!"#$#'*;(*)!"#$#'*;(*&!"#$#'*;(+*!"#$#'*;(++!"#$#'*;(+;!"#$#'*;(+(!"#$#'*;(+9!"#$#'*;(+%!"#$#'*;(+'!"#$#'*;(+:!"#$#'*;(+)!"#$#'*;(+&!"#$#'*;(;*!"#$#'*;(;+!"#$#'*;(;;!"#$#'*;(;(!"#$#'*;(;9!"#$#'*;(;%!"#$#'*;(;'!"#$#'*;(;:!"#$#'*;(;)

?3 5 $ ?3 5 ! 3 =. "" 4 /3"

$

!"#$#'*;(;&!"#$#'*;((*!"#$#'*;((+!"#$#'*;((;!"#$#'*;(((!"#$#'*;((9!"#$#'*;((%!"#$#'*;(('!"#$#'*;((:!"#$#'*;(()!"#$#'*;((&!"#$#'*;(9*!"#$#'*;(9+!"#$#'*;(9;!"#$#'*;(9(!"#$#'*;(99!"#$#'*;(9%!"#$#'*;(9'!"#$#'*;(9:!"#$#'*;(9)!"#$#'*;(9&!"#$#'*;(%*!"#$#'*;(%+!"#$#'*;(%; !"#$#'*;(%(!"#$#'*;(%9!"#$#'*;(%%!"#$#'*;(%'!"#$#'*;(%:!"#$#'*;(%)!"#$#'*;(%&!"#$#'*;('*!"#$#'*;('+!"#$#'*;(';!"#$#'*;('(!"#$#'*;('9!"#$#'*;('%!"#$#'*;(''!"#$#'*;(':!"#$#'*;(')!"#$#'*;('&!"#$#'*;(:*!"#$#'*;(:+!"#$#'*;(:;!"#$#'*;(:(!"#$#'*;(:9!"#$#'*;(:%!"#$#'*;(:' !"#$#'*;(::!"#$#'*;(:)!"#$#'*;(:&!"#$#'*;()*!"#$#'*;()+!"#$#'*;();!"#$#'*;()(!"#$#'*;()9!"#$#'*;()%!"#$#'*;()'!"#$#'*;():!"#$#'*;())!"#$#'*;()&!"#$#'*;(&*!"#$#'*;(&+!"#$#'*;(&;!"#$#'*;(&(!"#$#'*;(&9!"#$#'*;(&%!"#$#'*;(&'!"#$#'*;(&:!"#$#'*;(&)!"#$#'*;(&&!"#$#'*;9**

!"#$#'*;9*+!"#$#'*;9*;!"#$#'*;9*(!"#$#'*;9*9!"#$#'*;9*%!"#$#'*;9*'!"#$#'*;9*:!"#$#'*;9*)!"#$#'*;9*&!"#$#'*;9+*!"#$#'*;9++

|  | 5!$I$$. | 5- /.4 , | 6,661/ |
| --- | --- | --- | --- |
| !"#$#'*;9+;!"#$#'*;9+(!"#$#'*;9+9!"#$#'*;9+% | 5- /.4 , | 5- /.4 , | 6,661/ |

!"#$#'*;9+'

!"#$#'*;9+:!"#$#'*;9+)!"#$#'*;9+&!"#$#'*;9;*!"#$#'*;9;+!"#$#'*;9;;!"#$#'*;9;(!"#$#'*;9;9!"#$#'*;9;%!"#$#'*;9;'!"#$#'*;9;:!"#$#'*;9;)!"#$#'*;9;&!"#$#'*;9(*!"#$#'*;9(+!"#$#'*;9(;!"#$#'*;9((!"#$#'*;9(9!"#$#'*;9(%!"#$#'*;9('!"#$#'*;9(:!"#$#'*;9()!"#$#'*;9(&!"#$#'*;99* !"#$#'*;99+!"#$#'*;99;!"#$#'*;99(!"#$#'*;999!"#$#'*;99%!"#$#'*;99'!"#$#'*;99:!"#$#'*;99)!"#$#'*;99&!"#$#'*;9%*!"#$#'*;9%+!"#$#'*;9%;!"#$#'*;9%(!"#$#'*;9%9!"#$#'*;9%%!"#$#'*;9%'!"#$#'*;9%:!"#$#'*;9%)!"#$#'*;9%&!"#$#'*;9'*!"#$#'*;9'+

5!$I$$. 5- /.4 , 6,661/

!"#$#'*;9'(!"#$#'*;9'9!"#$#'*;9'%!"#$#'*;9''!"#$#'*;9':!"#$#'*;9')!"#$#'*;9'&!"#$#'*;9:*!"#$#'*;9:+!"#$#'*;9:;!"#$#'*;9:(!"#$#'*;9:9!"#$#'*;9:%!"#$#'*;9:'!"#$#'*;9::!"#$#'*;9:)!"#$#'*;9:&!"#$#'*;9)*!"#$#'*;9)+!"#$#'*;9);!"#$#'*;9)(!"#$#'*;9)9!"#$#'*;9)%!"#$#'*;9): !"#$#'*;9))!"#$#'*;9)&!"#$#'*;9&*!"#$#'*;9&+!"#$#'*;9&;!"#$#'*;9&(!"#$#'*;9&9!"#$#'*;9&%!"#$#'*;9&'!"#$#'*;9&:!"#$#'*;9&)!"#$#'*;9&&!"#$#'*;%**!"#$#'*;%*+!"#$#'*;%*;!"#$#'*;%*(!"#$#'*;%*9!"#$#'*;%*%!"#$#'*;%*'!"#$#'*;%*:!"#$#'*;%*)!"#$#'*;%*&

A ?5 ! .3 3 A 5 ! ? 3< / < 75 $5J 3 6A -$J 0- 6 "

5? 0K7 7

!"#$#'*;%+*!"#$#'*;%++!"#$#'*;%+;!"#$#'*;%+( .3 3 A 5 5 .3 3 A 5 3< / < 75 $5J 3 6A 3 6 -$J 0- 6 "5 0K

!"#$#'*;%+9!"#$#'*;%+%!"#$#'*;%+'!"#$#'*;%+: ? 5? 7 7

!"#$#'*;%+)!"#$#'*;%+&!"#$#'*;%;*!"#$#'*;%;+!"#$#'*;%;;!"#$#'*;%;(!"#$#'*;%;9!"#$#'*;%;%!"#$#'*;%;'!"#$#'*;%;:!"#$#'*;%;)!"#$#'*;%;&!"#$#'*;%(*!"#$#'*;%(+!"#$#'*;%(;

A ?5 ! .3 3 A 5 ! ? 3< / < 75 $5J 3 6A -$J 0- 6 "

5? 0K7 7

!"#$#'*;%((!"#$#'*;%(9!"#$#'*;%(%!"#$#'*;%('!"#$#'*;%(:!"#$#'*;%()!"#$#'*;%(&!"#$#'*;%9*!"#$#'*;%9+!"#$#'*;%9(!"#$#'*;%99!"#$#'*;%9%!"#$#'*;%9:!"#$#'*;%9&!"#$#'*;%%*!"#$#'*;%%+!"#$#'*;%%;

|  | .3 3 A 5 5  ? | .3 3 A 5 5? | 3< / < 75 3 6A 3 6 -$J 0- 6 "5 0K7 7 |
| --- | --- | --- | --- |
| !"#$#'*;%'9 | . 4  3 3 | . 4 | .00 4$ 2 3 6 - 3 |
| !"#$#'*;%'% | . 4  3 3 | . 4 3 3 | .00 4$ 4 2 - 3 |
| !"#$#'*;%''!"#$#'*;%:9!"#$#'*;%:' | . 4  3 3 | . 4 3 3 | .00 $ 4 - 3 |

!"#$#'*;'(;!"#$#'*;'((!"#$#'*;'(9!"#$#'*;'(%!"#$#'*;'('!"#$#'*;'(:!"#$#'*;'()!"#$#'*;'(&!"#$#'*;'9*!"#$#'*;'9+!"#$#'*;'9;!"#$#'*;'9(!"#$#'*;'99!"#$#'*;'9%!"#$#'*;'9'!"#$#'*;'9:!"#$#'*;'9)!"#$#'*;'9&!"#$#'*;'%*!"#$#'*;'%+!"#$#'*;'%;!"#$#'*;'%(!"#$#'*;'%9!"#$#'*;'%% !"#$#'*;'%'!"#$#'*;'%:!"#$#'*;'%)!"#$#'*;'%&!"#$#'*;''*!"#$#'*;''+!"#$#'*;'';!"#$#'*;''(!"#$#'*;''9!"#$#'*;''%!"#$#'*;'''!"#$#'*;'':!"#$#'*;'')!"#$#'*;''&!"#$#'*;':*!"#$#'*;':+!"#$#'*;':;!"#$#'*;':(!"#$#'*;':9!"#$#'*;':%!"#$#'*;':'!"#$#'*;'::!"#$#'*;':)!"#$#'*;':&

!"#$#'*;')*!"#$#'*;')+!"#$#'*;');!"#$#'*;')(!"#$#'*;')9!"#$#'*;')%!"#$#'*;')'!"#$#'*;'):!"#$#'*;'))!"#$#'*;')&!"#$#'*;'&*!"#$#'*;'&+!"#$#'*;'&;!"#$#'*;'&(!"#$#'*;'&9!"#$#'*;'&%!"#$#'*;'&'!"#$#'*;'&:!"#$#'*;'&)!"#$#'*;:**!"#$#'*;:*+!"#$#'*;:*(!"#$#'*;:*9!"#$#'*;:*%

!"#$#'*;:*'!"#$#'*;:*:!"#$#'*;:*)!"#$#'*;:*&!"#$#'*;:+*!"#$#'*;:++!"#$#'*;:+;!"#$#'*;:+(!"#$#'*;:+9!"#$#'*;:+%!"#$#'*;:+'!"#$#'*;:+:!"#$#'*;:+)!"#$#'*;:+&!"#$#'*;:;*!"#$#'*;:;+!"#$#'*;:;;!"#$#'*;:;(!"#$#'*;:;9!"#$#'*;:;%!"#$#'*;:;'!"#$#'*;:;:!"#$#'*;:;)!"#$#'*;:;&

!"#$#'*;:(*!"#$#'*;:(+!"#$#'*;:(;!"#$#'*;:((!"#$#'*;:(9!"#$#'*;:(%!"#$#'*;:('!"#$#'*;:(:!"#$#'*;:()!"#$#'*;:(&!"#$#'*;:9*!"#$#'*;:9+!"#$#'*;:9;!"#$#'*;:9(!"#$#'*;:99!"#$#'*;:9%!"#$#'*;:9'!"#$#'*;:9:!"#$#'*;:9)!"#$#'*;:9&!"#$#'*;:%*!"#$#'*;:%+!"#$#'*;:%;!"#$#'*;:%(

!"#$#'*;:%9!"#$#'*;:%%!"#$#'*;:%'!"#$#'*;:%:!"#$#'*;:%)!"#$#'*;:%&!"#$#'*;:'*!"#$#'*;:'+!"#$#'*;:';!"#$#'*;:'(!"#$#'*;:'9!"#$#'*;:'%!"#$#'*;:''!"#$#'*;:':!"#$#'*;:')!"#$#'*;:'&!"#$#'*;::*!"#$#'*;::+!"#$#'*;::;!"#$#'*;::(!"#$#'*;::9!"#$#'*;::%!"#$#'*;::'!"#$#'*;:::

!"#$#'*;::)!"#$#'*;::&!"#$#'*;:)*!"#$#'*;:)+!"#$#'*;:);!"#$#'*;:)(!"#$#'*;:)9!"#$#'*;:)%!"#$#'*;:)'!"#$#'*;:):!"#$#'*;:))!"#$#'*;:)&!"#$#'*;:&*!"#$#'*;:&+!"#$#'*;:&(!"#$#'*;:&%!"#$#'*;:&'!"#$#'*;:&:!"#$#'*;:&)!"#$#'*;:&&!"#$#'*;)**!"#$#'*;)*+!"#$#'*;)*;!"#$#'*;)*(

!"#$#'*;)*9!"#$#'*;)*'!"#$#'*;)*:!"#$#'*;)*)!"#$#'*;)*&!"#$#'*;)+*!"#$#'*;)++!"#$#'*;)+;!"#$#'*;)+(!"#$#'*;)+9!"#$#'*;)+%!"#$#'*;)+'!"#$#'*;)+:!"#$#'*;)+)!"#$#'*;)+&!"#$#'*;);*!"#$#'*;);+!"#$#'*;);;!"#$#'*;);(!"#$#'*;);9!"#$#'*;);%!"#$#'*;);'!"#$#'*;);)!"#$#'*;);&

!"#$#'*;)(*!"#$#'*;)(+!"#$#'*;)(;!"#$#'*;)((!"#$#'*;)(9!"#$#'*;)(%!"#$#'*;)('!"#$#'*;)(:!"#$#'*;)()!"#$#'*;)(&!"#$#'*;)9*!"#$#'*;)9+!"#$#'*;)9;!"#$#'*;)9(!"#$#'*;)99!"#$#'*;)9%!"#$#'*;)9'!"#$#'*;)9:!"#$#'*;)9)!"#$#'*;)9&!"#$#'*;)%*!"#$#'*;)%+!"#$#'*;)%(!"#$#'*;)%9

!"#$#'*;)%%!"#$#'*;)%'!"#$#'*;)%:!"#$#'*;)%)!"#$#'*;)%&!"#$#'*;)'*!"#$#'*;)'+!"#$#'*;)';!"#$#'*;)'(!"#$#'*;)'9!"#$#'*;)'%!"#$#'*;)''!"#$#'*;)':!"#$#'*;)')!"#$#'*;)'&!"#$#'*;):*!"#$#'*;):+!"#$#'*;):;!"#$#'*;):(!"#$#'*;):9!"#$#'*;):%!"#$#'*;):'!"#$#'*;)::!"#$#'*;):)

!"#$#'*;):&!"#$#'*;))*!"#$#'*;))+!"#$#'*;))(!"#$#'*;))9!"#$#'*;))%!"#$#'*;))'!"#$#'*;)):!"#$#'*;)))!"#$#'*;))&!"#$#'*;)&*!"#$#'*;)&+!"#$#'*;)&;!"#$#'*;)&9!"#$#'*;)&%!"#$#'*;)&'!"#$#'*;)&:!"#$#'*;)&)!"#$#'*;)&&!"#$#'*;&**!"#$#'*;&*+!"#$#'*;&*;!"#$#'*;&*(!"#$#'*;&*9 !"#$#'*;&*%!"#$#'*;&*'!"#$#'*;&*:!"#$#'*;&*)!"#$#'*;&*&!"#$#'*;&+*!"#$#'*;&++!"#$#'*;&+;!"#$#'*;&+(!"#$#'*;&+9!"#$#'*;&+'!"#$#'*;&+:!"#$#'*;&+)!"#$#'*;&+&!"#$#'*;&;*!"#$#'*;&;;!"#$#'*;&;(!"#$#'*;&;9!"#$#'*;&;'!"#$#'*;&;)!"#$#'*;&;&!"#$#'*;&(*

-5$=$.5 64"6>-4B 0" $ 43 68 67 5 ! 7

"

!"#$#'*;&(+!"#$#'*;&(9!"#$#'*;&(%!"#$#'*;&('!"#$#'*;&(:!"#$#'*;&9;!"#$#'*;&99!"#$#'*;&9'!"#$#'*;&9:!"#$#'*;&9)!"#$#'*;&9&!"#$#'*;&%*!"#$#'*;&%+!"#$#'*;&%;!"#$#'*;&%9!"#$#'*;&%%

?" 5 $ ?" 5 $ !8 6

| !"#$#'*;%::!"#$#'*;%:&!"#$#'*;%)*!"#$#'*;%)+ | " | " | $<$0 . 6 $3 7 6615 |
| --- | --- | --- | --- |
| !"#$#'*;';; | 54= | 64"6>-4B  " | / 6,. 0" 7 5 ! 7 |
| !"#$#'*;';9!"#$#'*;';%!"#$#'*;';'!"#$#'*;';: !"#$#'*;';)!"#$#'*;';&!"#$#'*;'(* | 54= | 64"6>-4B  " | / 6,. 0" 7 5 ! 7 |

| !"#$#'*(+;(!"#$#'*(+;9!"#$#'*(+;%!"#$#'*(+;'  !"#$#'*(+;:!"#$#'*(+;) | " | " | $<$0 . 6 $3 7 6615 |
| --- | --- | --- | --- |
| !"#$#'*(+(: | -3$// /4.. | -3$// /4.. | .! 3 34 11 ,3! $.34 . |
| !"#$#'*(+() | -3$// /4.. | -3$// /4.. | 34 ,311 .! 3 ! $.34 . |

!"#$#'*;&%'!"#$#'*;&%:!"#$#'*;&%)!"#$#'*;&%&!"#$#'*;&'*!"#$#'*;&'+!"#$#'*;&';!"#$#'*;&'(!"#$#'*;&'9!"#$#'*;&'%!"#$#'*;&''!"#$#'*;&':!"#$#'*;&')!"#$#'*;&'&!"#$#'*;&:*!"#$#'*;&:+!"#$#'*;&:;!"#$#'*;&:(!"#$#'*;&:9!"#$#'*;&:%!"#$#'*;&:'!"#$#'*;&::!"#$#'*;&:)!"#$#'*;&:& !"#$#'*;&)*!"#$#'*;&)+!"#$#'*;&);!"#$#'*;&)(!"#$#'*;&)9!"#$#'*;&)%!"#$#'*;&)'!"#$#'*;&):!"#$#'*;&))!"#$#'*;&)&!"#$#'*;&&*!"#$#'*;&&+!"#$#'*;&&;!"#$#'*;&&(!"#$#'*;&&9!"#$#'*;&&%!"#$#'*;&&'!"#$#'*;&&:!"#$#'*;&&)!"#$#'*;&&&

|  | 35 " 5 $ | 35 " 5  $ | 3" 0 <$/I |
| --- | --- | --- | --- |
| !"#$#'*(***!"#$#'*(**+!"#$#'*(**;!"#$#'*(**( | ?3 . | 35 " 5  $ | 3" 0 <$/I |
| !"#$#'*(**9!"#$#'*(**% | !5 = 3C3 . | 35 " 5  $ | 3" 0 <$/I |
| !"#$#'*(**'!"#$#'*(**:!"#$#'*(**)!"#$#'*(**& | 35 " 5 $ | 35 " 5  $ | 3" 0 <$/I |
| !"#$#'*(*+*!"#$#'*(*++ | 3. M3. $ | 35 " 5  $ | 3" 0 <$/I |
| !"#$#'*(*+;!"#$#'*(*+(!"#$#'*(*+9 | !5 = 3C3 . | 35 " 5  $ | 3" 0 <$/I |
| !"#$#'*(*+% | 35 " 5 $ | 35 " 5  $ | 3" 0 <$/I |
| !"#$#'*(*+'!"#$#'*(*+: | !5 = 3C3 . | 35 " 5  $ | 3" 0 <$/I |
| !"#$#'*(*+)!"#$#'*(*+&!"#$#'*(*;* | 35 " 5 $ | 35 " 5  $ | 3" 0 <$/I |
| !"#$#'*(*;+ | " ..- | $ / " .  $ | . 7. 4N !A 46 4 . |
| !"#$#'*(*;; | A OLP | $ / " .  $ | . 7. 4N !A 46 4 . |
| !"#$#'*(*;( | A OPA | $ / " .  $ | . 7. 4N !A 46 4 . |
| !"#$#'*(*;9 | .3N | $ / " .  $ | . 7. 4N !A 46 4 . |
| !"#$#'*(*;% | ?". . | $ / " .  $ | . 7. 4N !A 46 4 . |
| !"#$#'*(*;' | .3Q"" | $ / " .  $ | . 7. 4N !A 46 4 . |
| !"#$#'*(*;: | .3N | $ / " .  $ | . 7. 4N !A 46 4 . |
| !"#$#'*(*;) | 5 3 | $ / " .  $ | . 7. 4N !A 46 4 . |
| !"#$#'*(*;& | 5 3 3R | $ / " .  $ | . 7. 4N !A 46 4 . |
| !"#$#'*(*(* | 5 $. "" | $ / " .  $ | . 7. 4N !A 46 4 . |
| !"#$#'*(*(+ | ."!R | $ / " .  $ | . 7. 4N !A 46 4 . |
| !"#$#'*(*(; | .3Q"" | $ / " .  $ | . 7. 4N !A 46 4 . |
| !"#$#'*(*(( | A ! S1. | $ / " .  $ | . 7. 4N !A 46 4 . |
| !"#$#'*(*(9 | A OLP | $ / " .  $ | . 7. 4N !A 46 4 . |
| !"#$#'*(*(% | 3 P | $ / " .  $ | . 7. 4N !A 46 4 . |
| !"#$#'*(*(' | 5 | $ / " .  $ | . 7. 4N !A 46 4 . |
| !"#$#'*(*(: | 5 " | $ / " .  $ | . 7. 4N !A 46 4 . |
| !"#$#'*(*() | .3N | $ / " .  $ | . 7. 4N !A 46 4 . |
| !"#$#'*(*(& | 5 3 3R | $ / " .  $ | . 7. 4N !A 46 4 . |
| !"#$#'*(*9*!"#$#'*(*9+!"#$#'*(*9(!"#$#'*(*99 !"#$#'*(*9'!"#$#'*(*9:!"#$#'*(*9) | 3?="5$7" | 3?="5$7" | 7 . $ 3 /3 1 0 - |
| !"#$#'*(*9&!"#$#'*(*%*!"#$#'*(*%+!"#$#'*(*%; | ?" 5 $?" 5 $ | ?" 5 $?" 5 $ | 8 ! $ 6 |

!"#$#'*(*);!"#$#'*(*)(!"#$#'*(*)9!"#$#'*(*)%!"#$#'*(*)'!"#$#'*(*):!"#$#'*(*))!"#$#'*(*)&!"#$#'*(*&*!"#$#'*(*&+!"#$#'*(*&;!"#$#'*(*&(!"#$#'*(*&9!"#$#'*(*&%!"#$#'*(*&'!"#$#'*(*&:!"#$#'*(*&)!"#$#'*(*&&!"#$#'*(+**!"#$#'*(+*+!"#$#'*(+*;!"#$#'*(+*(!"#$#'*(+*9!"#$#'*(+*% !"#$#'*(+*'!"#$#'*(+*:!"#$#'*(+*)!"#$#'*(+*&!"#$#'*(++*!"#$#'*(+++!"#$#'*(++;!"#$#'*(++(!"#$#'*(++9!"#$#'*(++%!"#$#'*(++'!"#$#'*(++:!"#$#'*(++)!"#$#'*(+;*!"#$#'*(+;;

$ ?5 5 $ ?5 3 $ > T " 6 7 $ " A$ A

$3

| !"#$#'*(+(& | -3$// /4.. | -3$// /4.. | 34 .! 3 11 ,3! $.34 . |
| --- | --- | --- | --- |
| !"#$#'*(+9* | -3$// /4.. | -3$// /4.. | 34 .! 3 ,311 ! $.34 . |
| !"#$#'*(+9+ | -3$// /4.. | -3$// /4.. | ,3.! 3 34 11 ! $34 .. |
| !"#$#'*(+9; | -3$// /4.. | -3$// /4.. | 11 .! 3 ,334 $! 34 .. |
| !"#$#'*(+9( | -3$// /4.. | -3$// /4.. | 34 ,3.! 3 11 ! $.34 . |
| !"#$#'*(+99 | -3$// /4.. | -3$// /4.. | .! 3 34 ,311 ! $34 .. |
| !"#$#'*(+9% | -3$// /4.. | -3$// /4.. | ,3.! 3 34 11 ! $.34 . |
| !"#$#'*(+9' | -3$// /4.. | -3$// /4.. | .! 3 34 11 ! ,3$34 .. |
| !"#$#'*(+9: | -3$// /4.. | -3$// /4.. | .! 3 11 ,334 $! .34 . |
| !"#$#'*(+9) | -3$// /4.. | -3$// /4.. | .! 3 ,334 $! 11 34 .. |
| !"#$#'*(+9& | -3$// /4.. | -3$// /4.. | ,311 .! 3 34 $! .34 . |
| !"#$#'*(+%* | -3$// /4.. | -3$// /4.. | 11 ,334 .! 3 $! 34 .. |
| !"#$#'*(+%+ | -3$// /4.. | -3$// /4.. | ,311 .! 3 34 ! $34 .. |
| !"#$#'*(+%; | -3$// /4.. | -3$// /4.. | 11 .! 3 ,334 ! $.34 . |
| !"#$#'*(+%( | -3$// /4.. | -3$// /4.. | ,3.! 3 11 $! 34 .34 . |
| !"#$#'*(+%9 | -3$// /4.. | -3$// /4.. | 34 ,3.! 3 11 ! $.34 . |
| !"#$#'*(+%% | -3$// /4.. | -3$// /4.. | 34 ,311 .! 3 $! 34 .. |
| !"#$#'*(+%' | -3$// /4.. | -3$// /4.. | 11 ,3.! 3 34 ! $34 .. |
| !"#$#'*(+%: | -3$// /4.. | -3$// /4.. | ,334 11 .! 3 $! .34 . |
| !"#$#'*(+%) | -3$// /4.. | -3$// /4.. | 11 .! 3 ,334 ! $34 .. |
| !"#$#'*(+%& | -3$// /4.. | -3$// /4.. | 11 ,3.! 3 34 $! 34 .. |
| !"#$#'*(+'* | -3$// /4.. | -3$// /4.. | 11 $! ,3.! 3 34 34 .. |
| !"#$#'*(+'+ | -3$// /4.. | -3$// /4.. | .! 3 11 ,334 ! $.34 . |
| !"#$#'*(+'; | -3$// /4.. | -3$// /4.. | .! 3 ,334 11 ! $.34 . |
| !"#$#'*(+'( | -3$// /4.. | -3$// /4.. | .! 3 34 ! $,311 34 .. |
| !"#$#'*(+'9 | -3$// /4.. | -3$// /4.. | .! 3 ,334 11 ! $.34 . |
| !"#$#'*(+'% | -3$// /4.. | -3$// /4.. | 34 ,311 .! 3 ! $34 .. |
| !"#$#'*(+'' | -3$// /4.. | -3$// /4.. | 34 .! 3 11 ,3$! 34 .. |
| !"#$#'*(+': | -3$// /4.. | -3$// /4.. | 34 .! 3 ,311 $! 34 .. |
| !"#$#'*(+') | -3$// /4.. | -3$// /4.. | 34 11 .! 3 $! ,3.34 . |
| !"#$#'*(+'& | -3$// /4.. | -3$// /4.. | ,311 .! 3 34 $! 34 .. |
| !"#$#'*(+:* | -3$// /4.. | -3$// /4.. | 34 11 .! 3 ,3$! 34 .. |
| !"#$#'*(+:+ | -3$// /4.. | -3$// /4.. | 11 .! 3 ,334 ! $.34 . |
| !"#$#'*(+:; | -3$// /4.. | -3$// /4.. | .! 3 34 11 $,3! .34 . |
| !"#$#'*(+:( | -3$// /4.. | -3$// /4.. | 11 .! 3 34 ,3$! 34 .. |
| !"#$#'*(+:9 | -3$// /4.. | -3$// /4.. | ,311 34 ! $.! 3 34 .. |
| !"#$#'*(+:% | -3$// /4.. | -3$// /4.. | 11 .! 3 $! ,334 .34 . |
| !"#$#'*(+:' | -3$// /4.. | -3$// /4.. | .! 3 11 ,334 $! .34 . |
| !"#$#'*(+:: | -3$// /4.. | -3$// /4.. | 11 ,3! 34 .! 3 $34 .. |
| !"#$#'*(+:) | -3$// /4.. | -3$// /4.. | 34 ,3.! 3 11 $! .34 . |
| !"#$#'*(+:& | -3$// /4.. | -3$// /4.. | .! 3 11 ,334 ! $.34 . |
| !"#$#'*(+)* | -3$// /4.. | -3$// /4.. | 11 .! 3 ,3$34 ! 34 .. |
| !"#$#'*(+)+ | -3$// /4.. | -3$// /4.. | ,3.! 3 11 34 $! 34 .. |
| !"#$#'*(+); | -3$// /4.. | -3$// /4.. | 34 11 .! 3 ,3! $34 .. |
| !"#$#'*(+)( | -3$// /4.. | -3$// /4.. | 11 ,334 .! 3 ! $34 .. |
| !"#$#'*(+)9 | -3$// /4.. | -3$// /4.. | 34 11 ,3.! 3 ! $34 .. |
| !"#$#'*(+)% | -3$// /4.. | -3$// /4.. | .! 3 34 11 ,3$! 34 .. |
| !"#$#'*(+)' | -3$// /4.. | -3$// /4.. | ,334 11 .! 3 ! $34 .. |
| !"#$#'*(+): | -3$// /4.. | -3$// /4.. | .! 3 34 ,311 $! .34 . |

!"#$#'*(+))!"#$#'*(+)&!"#$#'*(+&*!"#$#'*(+&+!"#$#'*(+&;!"#$#'*(+&(!"#$#'*(+&9!"#$#'*(+&%!"#$#'*(+&'!"#$#'*(+&:!"#$#'*(+&)!"#$#'*(+&&!"#$#'*(;**!"#$#'*(;*+!"#$#'*(;*;!"#$#'*(;*(!"#$#'*(;*9!"#$#'*(;*%!"#$#'*(;*'!"#$#'*(;*:!"#$#'*(;*)!"#$#'*(;*&!"#$#'*(;+*!"#$#'*(;++

!"#$#'*(;+;!"#$#'*(;+(!"#$#'*(;+9!"#$#'*(;+%

|  | 4A ?3 . "  5 ! | 4A ?3 .  " 5 ! | "5!.B 7 |
| --- | --- | --- | --- |
| !"#$#'*(;+'!"#$#'*(;+:!"#$#'*(;+)!"#$#'*(;+&  !"#$#'*(;;* | .5?" =$A = ,DD  1 | .5?" =$A = ,DD  1 | $ 0 4- 3 .4 5 1" 7 <" /0 |
| !"#$#'*(;;+!"#$#'*(;;; | - $34 . | 4$1.4 | 33 3?//!3 5 72 1 3 3 5 103 57-3 3 3 3 86 6 3 //3"3 3 53 6 |
| !"#$#'*(;;(!"#$#'*(;;9!"#$#'*(;;% | - $34 . | 4$1.4 | 3 3 5 33 3?//!3 5 72 1 103 57-3 3 3 3 86 6 3 //3"3 3 53 6 |
| !"#$#'*(;()!"#$#'*(;(& | - $34 . | 4$1.4 | 33 3?//!3 572 1 3 3 5 103 57-3 3 3 3 86 6 3 //3"3 3 53 6 |
| !"#$#'*(;9*!"#$#'*(;9+ | - $34 . | 4$1.4 | 72 1 33 3?//!3 5 3 3 5 103 57-3 3 3 3 86 6 3 //3"3 3 53 6 |
| !"#$#'*(;9;!"#$#'*(;9( | - $34 . | 4$1.4 | 1 33 3?//!3 5 72 1 3 3 5 03 57-3 3 3 3 86 6 3 //3"3 3 53 6 |
| !"#$#'*(;99!"#$#'*(;9% | - $34 . | 4$1.4 | 0 33 3?//!3 5 72 1 3 3 5 13 57-3 3 3 3 86 6 3 //3"3 3 53 6 |
| !"#$#'*(;9'!"#$#'*(;9: | - $34 . | 4$1.4 | 3 5 33 3?//!3 5 72 1 3 3 5 1 07-3 3 3 3 86 6 3 //3"3 3 53 6 |
| !"#$#'*(;9) | 11 "3 | 11 "3 | 7106 $ " 5. 4 . ,/12" 3 0 4 - .3 $ $3 5 8 . 0 |
| !"#$#'*(;9&!"#$#'*(;%* | - $34 . | 4$1.4 | 3 5 33 3?//! 72 1 3 3 5 103 57-3 3 3 3 86 6 3 //3"3 3 53 6 |
| !"#$#'*(;%+!"#$#'*(;%;!"#$#'*(;%(!"#$#'*(;%9 | ?A $ | ?A $ | "4 5I$3=$5 640 < |

!"#$#'*(;%%

!"#$#'*(;':!"#$#'*(;')!"#$#'*(;'&!"#$#'*(;:*!"#$#'*(;:;!"#$#'*(;:(!"#$#'*(;:9!"#$#'*(;:%!"#$#'*(;:'!"#$#'*(;::!"#$#'*(;:)!"#$#'*(;:&!"#$#'*(;)*!"#$#'*(;)+!"#$#'*(;);!"#$#'*(;)(!"#$#'*(;)9!"#$#'*(;)%!"#$#'*(;):!"#$#'*(;))!"#$#'*(;)&!"#$#'*(;&*!"#$#'*(;&+!"#$#'*(;&; !"#$#'*(;&(!"#$#'*(;&9!"#$#'*(;&%!"#$#'*(;&'!"#$#'*(;&:!"#$#'*(;&)!"#$#'*(;&&!"#$#'*((**!"#$#'*((*+!"#$#'*((*;!"#$#'*((*(!"#$#'*((*%!"#$#'*((*'!"#$#'*((*)!"#$#'*((*&!"#$#'*((+*!"#$#'*((++!"#$#'*((+;!"#$#'*((+(!"#$#'*((+9!"#$#'*((+%!"#$#'*((+'!"#$#'*((+)!"#$#'*((+&

!"#$#'*((;*!"#$#'*((;+!"#$#'*((;;!"#$#'*((;(!"#$#'*((;9!"#$#'*((;%!"#$#'*((;:!"#$#'*((;)!"#$#'*(((*!"#$#'*(((+!"#$#'*(((;!"#$#'*((((!"#$#'*(((9!"#$#'*(((%!"#$#'*((('!"#$#'*(((:!"#$#'*((()!"#$#'*(((&!"#$#'*((9*!"#$#'*((9+!"#$#'*((9;!"#$#'*((9(!"#$#'*((99!"#$#'*((9%

!"#$#'*((9'!"#$#'*((9)!"#$#'*((%*!"#$#'*((%+!"#$#'*((%;!"#$#'*((%(!"#$#'*((%9!"#$#'*((%%!"#$#'*((%:!"#$#'*((%)!"#$#'*((%&!"#$#'*(('*!"#$#'*(('+!"#$#'*((';!"#$#'*(('(!"#$#'*(('9!"#$#'*(('%!"#$#'*((''!"#$#'*((':!"#$#'*((')!"#$#'*(('&!"#$#'*((:*!"#$#'*((:+!"#$#'*((:;

!"#$#'*((:(!"#$#'*((:9!"#$#'*((:%!"#$#'*((:'!"#$#'*((::!"#$#'*((:)!"#$#'*((:&!"#$#'*(()*!"#$#'*(()+!"#$#'*(();!"#$#'*(()(!"#$#'*(()9!"#$#'*(()%!"#$#'*(()'!"#$#'*(():!"#$#'*(())!"#$#'*(()&!"#$#'*((&*!"#$#'*((&+!"#$#'*((&;!"#$#'*((&(!"#$#'*((&9!"#$#'*((&%!"#$#'*((&'

!"#$#'*((&:!"#$#'*((&)!"#$#'*((&&!"#$#'*(9**!"#$#'*(9*+!"#$#'*(9*;!"#$#'*(9*(!"#$#'*(9*9!"#$#'*(9*%!"#$#'*(9*'!"#$#'*(9*:!"#$#'*(9*)!"#$#'*(9*&!"#$#'*(9+*!"#$#'*(9++!"#$#'*(9+;!"#$#'*(9+(!"#$#'*(9+9!"#$#'*(9+%!"#$#'*(9+'!"#$#'*(9+:!"#$#'*(9+)!"#$#'*(9+&!"#$#'*(9;* !"#$#'*(9;+!"#$#'*(9;;!"#$#'*(9;(!"#$#'*(9;9!"#$#'*(9;%!"#$#'*(9;'!"#$#'*(9;:!"#$#'*(9;)!"#$#'*(9;&!"#$#'*(9(*!"#$#'*(9(+!"#$#'*(9(;!"#$#'*(9((!"#$#'*(9(9!"#$#'*(9(%!"#$#'*(9('!"#$#'*(9(:!"#$#'*(9()!"#$#'*(9(&!"#$#'*(99*!"#$#'*(99+!"#$#'*(99;!"#$#'*(99(!"#$#'*(999 !"#$#'*(99%!"#$#'*(99'!"#$#'*(99:!"#$#'*(99)!"#$#'*(99&!"#$#'*(9%*!"#$#'*(9%+!"#$#'*(9%;!"#$#'*(9%(!"#$#'*(9%9!"#$#'*(9%%!"#$#'*(9%'!"#$#'*(9%:!"#$#'*(9%)!"#$#'*(9%&!"#$#'*(9'*!"#$#'*(9'+!"#$#'*(9';!"#$#'*(9'(!"#$#'*(9'9!"#$#'*(9'%!"#$#'*(9''!"#$#'*(9')!"#$#'*(9'&

!"#$#'*(9:*!"#$#'*(9:+!"#$#'*(9:;!"#$#'*(9:(!"#$#'*(9:9!"#$#'*(9:%!"#$#'*(9:'!"#$#'*(9::!"#$#'*(9:)!"#$#'*(9:&!"#$#'*(9)*!"#$#'*(9)+!"#$#'*(9);!"#$#'*(9)(!"#$#'*(9)9!"#$#'*(9)%!"#$#'*(9)'!"#$#'*(9))!"#$#'*(9)&!"#$#'*(9&*!"#$#'*(9&+!"#$#'*(9&;!"#$#'*(9&(!"#$#'*(9&9 !"#$#'*(9&%!"#$#'*(9&'!"#$#'*(9&:!"#$#'*(9&)!"#$#'*(9&&!"#$#'*(%**!"#$#'*(%*+!"#$#'*(%*;!"#$#'*(%*(!"#$#'*(%*9!"#$#'*(%*%!"#$#'*(%*'!"#$#'*(%*:!"#$#'*(%*)!"#$#'*(%*&!"#$#'*(%+*!"#$#'*(%++!"#$#'*(%+;!"#$#'*(%+(!"#$#'*(%+9!"#$#'*(%+%!"#$#'*(%+'!"#$#'*(%+:!"#$#'*(%+) !"#$#'*(%+&!"#$#'*(%;*!"#$#'*(%;+!"#$#'*(%;;!"#$#'*(%;(!"#$#'*(%;9!"#$#'*(%;%!"#$#'*(%;'!"#$#'*(%;:!"#$#'*(%;&!"#$#'*(%(+!"#$#'*(%((!"#$#'*(%(9!"#$#'*(%('!"#$#'*(%(:!"#$#'*(%()!"#$#'*(%(&!"#$#'*(%9*!"#$#'*(%9+!"#$#'*(%9;!"#$#'*(%9(!"#$#'*(%99!"#$#'*(%9%!"#$#'*(%9:

!"#$#'*(%9)!"#$#'*(%9&!"#$#'*(%%*!"#$#'*(%%+!"#$#'*(%%;!"#$#'*(%%(!"#$#'*(%%9!"#$#'*(%%%!"#$#'*(%%'!"#$#'*(%%:!"#$#'*(%%)!"#$#'*(%%&!"#$#'*(%'*!"#$#'*(%'+!"#$#'*(%';!"#$#'*(%'(!"#$#'*(%'9!"#$#'*(%'%!"#$#'*(%''!"#$#'*(%':!"#$#'*(%')!"#$#'*(%'&!"#$#'*(%:*!"#$#'*(%:+

!"#$#'*(%:;!"#$#'*(%:(!"#$#'*(%:9!"#$#'*(%:%!"#$#'*(%:'!"#$#'*(%::!"#$#'*(%:)!"#$#'*(%:&!"#$#'*(%)*!"#$#'*(%)+!"#$#'*(%);!"#$#'*(%)(!"#$#'*(%)9!"#$#'*(%)%!"#$#'*(%)'!"#$#'*(%):!"#$#'*(%))!"#$#'*(%)&!"#$#'*(%&*!"#$#'*(%&+!"#$#'*(%&;!"#$#'*(%&(!"#$#'*(%&9!"#$#'*(%&%

!"#$#'*(%&'!"#$#'*(%&:!"#$#'*(%&)!"#$#'*(%&&!"#$#'*('**!"#$#'*('*+!"#$#'*('*;!"#$#'*('*(!"#$#'*('*9!"#$#'*('*'!"#$#'*('*:!"#$#'*('*)!"#$#'*('*&!"#$#'*('+*!"#$#'*('++!"#$#'*('+;!"#$#'*('+(!"#$#'*('+9!"#$#'*('+%!"#$#'*('+'!"#$#'*('+:!"#$#'*('+)!"#$#'*('+&!"#$#'*(';*

!"#$#'*(';+!"#$#'*(';;!"#$#'*(';(!"#$#'*(';9!"#$#'*(';%!"#$#'*(';'!"#$#'*(';:!"#$#'*(';)!"#$#'*(';&!"#$#'*('(*!"#$#'*('(+!"#$#'*('(;!"#$#'*('((!"#$#'*('(9!"#$#'*('(%!"#$#'*('('!"#$#'*('(:!"#$#'*('()!"#$#'*('(&!"#$#'*('9*!"#$#'*('9+!"#$#'*('9;!"#$#'*('9(!"#$#'*('99

!"#$#'*('9%!"#$#'*('9'!"#$#'*('9:!"#$#'*('9)!"#$#'*('9&!"#$#'*('%*!"#$#'*('%+!"#$#'*('%;!"#$#'*('%(!"#$#'*('%9!"#$#'*('%%!"#$#'*('%'!"#$#'*('%:!"#$#'*('%)!"#$#'*('%&!"#$#'*(''*!"#$#'*(''+!"#$#'*('';!"#$#'*(''(!"#$#'*(''9!"#$#'*(''%!"#$#'*('''!"#$#'*('':!"#$#'*('')

!"#$#'*(''&!"#$#'*(':*!"#$#'*(':+!"#$#'*(':;!"#$#'*(':(!"#$#'*(':9!"#$#'*(':%!"#$#'*(':'!"#$#'*('::!"#$#'*(':)!"#$#'*(':&!"#$#'*(')*!"#$#'*(')+!"#$#'*(');!"#$#'*(')(!"#$#'*(')9!"#$#'*(')%!"#$#'*(')'!"#$#'*('):!"#$#'*('))!"#$#'*(')&!"#$#'*('&*!"#$#'*('&+!"#$#'*('&;

!"#$#'*('&(!"#$#'*('&9!"#$#'*('&%!"#$#'*('&'!"#$#'*('&:!"#$#'*('&)!"#$#'*('&&!"#$#'*(:**!"#$#'*(:*;!"#$#'*(:*(!"#$#'*(:*9!"#$#'*(:*%!"#$#'*(:*'!"#$#'*(:*:!"#$#'*(:*)!"#$#'*(:*&!"#$#'*(:+*!"#$#'*(:++!"#$#'*(:+;!"#$#'*(:+(!"#$#'*(:+9!"#$#'*(:+%!"#$#'*(:+'!"#$#'*(:+:

!"#$#'*(:+)!"#$#'*(:+&!"#$#'*(:;*!"#$#'*(:;+!"#$#'*(:;;!"#$#'*(:;(!"#$#'*(:;9!"#$#'*(:;%!"#$#'*(:;'!"#$#'*(:;:!"#$#'*(:;)!"#$#'*(:;&!"#$#'*(:(*!"#$#'*(:(+!"#$#'*(:(;!"#$#'*(:((!"#$#'*(:(9!"#$#'*(:(%!"#$#'*(:('!"#$#'*(:(:!"#$#'*(:()!"#$#'*(:(&!"#$#'*(:9*!"#$#'*(:9+ !"#$#'*(:9;!"#$#'*(:9(!"#$#'*(:99!"#$#'*(:9%!"#$#'*(:9'!"#$#'*(:9:!"#$#'*(:9)!"#$#'*(:9&!"#$#'*(:%*!"#$#'*(:%+!"#$#'*(:%;!"#$#'*(:%(!"#$#'*(:%9!"#$#'*(:%%!"#$#'*(:%'!"#$#'*(:%:!"#$#'*(:%)

A 1!!75 .1 - 7 "," 0 "=.D7U--! 1 .1

>J 3 4 6 .- - /- 1 72

!"#$#'*(:%&!"#$#'*(:'*!"#$#'*(:'+!"#$#'*(:';!"#$#'*(:'(!"#$#'*(:'9!"#$#'*(:'%!"#$#'*(:''!"#$#'*(:':!"#$#'*(:')!"#$#'*(:'&!"#$#'*(::*!"#$#'*(::+!"#$#'*(::;!"#$#'*(::(!"#$#'*(::9!"#$#'*(::%!"#$#'*(::'!"#$#'*(:::!"#$#'*(::)!"#$#'*(::&!"#$#'*(:)*!"#$#'*(:)+!"#$#'*(:); !"#$#'*(:)(!"#$#'*(:)9!"#$#'*(:)%!"#$#'*(:)'!"#$#'*(:):!"#$#'*(:))!"#$#'*(:)&!"#$#'*(:&*!"#$#'*(:&+!"#$#'*(:&;!"#$#'*(:&(!"#$#'*(:&9!"#$#'*(:&%!"#$#'*(:&'!"#$#'*(:&:!"#$#'*(:&)!"#$#'*(:&&!"#$#'*()**!"#$#'*()*+!"#$#'*()*;!"#$#'*()*(!"#$#'*()*9!"#$#'*()*%!"#$#'*()*'

!"#$#'*()*:!"#$#'*()*)!"#$#'*()*&!"#$#'*()+*!"#$#'*()++!"#$#'*()+;!"#$#'*()+(!"#$#'*()+9!"#$#'*()+%!"#$#'*()+'!"#$#'*()+:!"#$#'*()+)!"#$#'*()+&!"#$#'*();*!"#$#'*();+!"#$#'*();;!"#$#'*();(!"#$#'*();9!"#$#'*();%!"#$#'*();'!"#$#'*();:!"#$#'*();)!"#$#'*();&!"#$#'*()(*

!"#$#'*()(+!"#$#'*()(;!"#$#'*()((!"#$#'*()(9!"#$#'*()(%!"#$#'*()('!"#$#'*()(:!"#$#'*()()!"#$#'*()(&!"#$#'*()9*!"#$#'*()9+!"#$#'*()9;!"#$#'*()9(!"#$#'*()99!"#$#'*()9%!"#$#'*()9'!"#$#'*()9:!"#$#'*()9)!"#$#'*()9&!"#$#'*()%*!"#$#'*()%+!"#$#'*()%;!"#$#'*()%(!"#$#'*()%9

!"#$#'*()%%!"#$#'*()%'!"#$#'*()%:!"#$#'*()%)!"#$#'*()%&!"#$#'*()'*!"#$#'*()'+!"#$#'*()';!"#$#'*()'(!"#$#'*()'9!"#$#'*()'%!"#$#'*()''!"#$#'*()':!"#$#'*()')!"#$#'*()'&!"#$#'*():*!"#$#'*():+!"#$#'*():;!"#$#'*():(!"#$#'*():9!"#$#'*():%!"#$#'*():'!"#$#'*()::!"#$#'*():) !"#$#'*():&!"#$#'*())*!"#$#'*())+!"#$#'*());!"#$#'*())(!"#$#'*())9!"#$#'*())%!"#$#'*())'!"#$#'*()):!"#$#'*()))!"#$#'*())&!"#$#'*()&*!"#$#'*()&+!"#$#'*()&;!"#$#'*()&(!"#$#'*()&9!"#$#'*()&%!"#$#'*()&'!"#$#'*()&:!"#$#'*()&)!"#$#'*()&&!"#$#'*(&**!"#$#'*(&*+!"#$#'*(&*; !"#$#'*(&*(!"#$#'*(&*9!"#$#'*(&*%!"#$#'*(&*'!"#$#'*(&*:!"#$#'*(&*)!"#$#'*(&*&!"#$#'*(&+*!"#$#'*(&++!"#$#'*(&+;!"#$#'*(&+(!"#$#'*(&+9!"#$#'*(&+%!"#$#'*(&+'!"#$#'*(&+:!"#$#'*(&+)!"#$#'*(&+&!"#$#'*(&;*!"#$#'*(&;+!"#$#'*(&;;!"#$#'*(&;(!"#$#'*(&;9!"#$#'*(&;%!"#$#'*(&;' !"#$#'*(&;:!"#$#'*(&;)!"#$#'*(&;&!"#$#'*(&(*!"#$#'*(&(+!"#$#'*(&(;!"#$#'*(&((!"#$#'*(&(9!"#$#'*(&(%!"#$#'*(&('!"#$#'*(&(:!"#$#'*(&()!"#$#'*(&(&!"#$#'*(&9*!"#$#'*(&9+!"#$#'*(&9;!"#$#'*(&9(!"#$#'*(&99!"#$#'*(&9%!"#$#'*(&9'!"#$#'*(&9:!"#$#'*(&9)!"#$#'*(&9&!"#$#'*(&%*

!"#$#'*(&%+!"#$#'*(&%;!"#$#'*(&%(!"#$#'*(&%9!"#$#'*(&%%!"#$#'*(&%'!"#$#'*(&%:!"#$#'*(&%)!"#$#'*(&%&!"#$#'*(&'*!"#$#'*(&'+!"#$#'*(&';!"#$#'*(&'(!"#$#'*(&'9!"#$#'*(&'%!"#$#'*(&''!"#$#'*(&':!"#$#'*(&')!"#$#'*(&'&!"#$#'*(&:*!"#$#'*(&:+!"#$#'*(&:;!"#$#'*(&:(!"#$#'*(&:9 !"#$#'*(&:%!"#$#'*(&:'!"#$#'*(&::!"#$#'*(&:)!"#$#'*(&:&!"#$#'*(&)*!"#$#'*(&)+!"#$#'*(&);!"#$#'*(&)(!"#$#'*(&)9!"#$#'*(&)%!"#$#'*(&)'!"#$#'*(&):!"#$#'*(&))!"#$#'*(&)&!"#$#'*(&&*!"#$#'*(&&+!"#$#'*(&&;!"#$#'*(&&(!"#$#'*(&&9!"#$#'*(&&%!"#$#'*(&&'!"#$#'*(&&:!"#$#'*(&&) !"#$#'*(&&&!"#$#'*9***!"#$#'*9**+!"#$#'*9**;!"#$#'*9**(!"#$#'*9**9!"#$#'*9**%!"#$#'*9**'!"#$#'*9**:!"#$#'*9**)!"#$#'*9**&!"#$#'*9*+*!"#$#'*9*++!"#$#'*9*+;!"#$#'*9*+(!"#$#'*9*+9!"#$#'*9*+%!"#$#'*9*+'!"#$#'*9*+:!"#$#'*9*+)!"#$#'*9*+&!"#$#'*9*;*!"#$#'*9*;+!"#$#'*9*;;

!"#$#'*9*;(!"#$#'*9*;9!"#$#'*9*;%!"#$#'*9*;'!"#$#'*9*;:!"#$#'*9*;)!"#$#'*9*;&!"#$#'*9*(*!"#$#'*9*(+!"#$#'*9*(;!"#$#'*9*((!"#$#'*9*(9!"#$#'*9*(%!"#$#'*9*('!"#$#'*9*(:!"#$#'*9*()!"#$#'*9*(&!"#$#'*9*9*!"#$#'*9*9+!"#$#'*9*9;!"#$#'*9*9(!"#$#'*9*99!"#$#'*9*9%!"#$#'*9*9'

!"#$#'*9*9:!"#$#'*9*9)!"#$#'*9*9&!"#$#'*9*%*!"#$#'*9*%+!"#$#'*9*%;!"#$#'*9*%(!"#$#'*9*%9!"#$#'*9*%%!"#$#'*9*%'!"#$#'*9*%:!"#$#'*9*%)!"#$#'*9*%&!"#$#'*9*'*!"#$#'*9*'+!"#$#'*9*';!"#$#'*9*'(!"#$#'*9*'9!"#$#'*9*'%!"#$#'*9*''!"#$#'*9*':!"#$#'*9*')!"#$#'*9*'&!"#$#'*9*:*

!"#$#'*9*:+!"#$#'*9*:;!"#$#'*9*:(!"#$#'*9*:9!"#$#'*9*:%!"#$#'*9*:'!"#$#'*9*::!"#$#'*9*:)!"#$#'*9*:&!"#$#'*9*)*!"#$#'*9*)+!"#$#'*9*);!"#$#'*9*)(!"#$#'*9*)9!"#$#'*9*)%!"#$#'*9*)'!"#$#'*9*):!"#$#'*9*))!"#$#'*9*)&!"#$#'*9*&*!"#$#'*9*&+!"#$#'*9*&;!"#$#'*9*&(!"#$#'*9*&9

!"#$#'*9*&%!"#$#'*9*&'!"#$#'*9*&:!"#$#'*9*&)!"#$#'*9*&&!"#$#'*9+**!"#$#'*9+*+!"#$#'*9+*;!"#$#'*9+*(!"#$#'*9+*9!"#$#'*9+*%!"#$#'*9+*'!"#$#'*9+*:!"#$#'*9+*)!"#$#'*9+*&!"#$#'*9++*!"#$#'*9+++!"#$#'*9++;!"#$#'*9++(!"#$#'*9++9!"#$#'*9++%!"#$#'*9++'!"#$#'*9++:!"#$#'*9++)

!"#$#'*9++&!"#$#'*9+;*!"#$#'*9+;+!"#$#'*9+;;!"#$#'*9+;(!"#$#'*9+;9!"#$#'*9+;%!"#$#'*9+;'!"#$#'*9+;:!"#$#'*9+;)!"#$#'*9+;&!"#$#'*9+(*!"#$#'*9+(+!"#$#'*9+(;!"#$#'*9+((!"#$#'*9+(9!"#$#'*9+(%!"#$#'*9+('!"#$#'*9+(:!"#$#'*9+()!"#$#'*9+(&!"#$#'*9+9*!"#$#'*9+9+!"#$#'*9+9;

!"#$#'*9+9(!"#$#'*9+99!"#$#'*9+9%!"#$#'*9+9'!"#$#'*9+9:!"#$#'*9+9)!"#$#'*9+9&!"#$#'*9+%*!"#$#'*9+%+!"#$#'*9+%;!"#$#'*9+%(!"#$#'*9+%9!"#$#'*9+%%!"#$#'*9+%'!"#$#'*9+%:!"#$#'*9+%)!"#$#'*9+%&!"#$#'*9+'*!"#$#'*9+'+!"#$#'*9+';!"#$#'*9+'(!"#$#'*9+'9!"#$#'*9+'%!"#$#'*9+''

!"#$#'*9+':!"#$#'*9+')!"#$#'*9+'&!"#$#'*9+:*!"#$#'*9+:+!"#$#'*9+:;!"#$#'*9+:(!"#$#'*9+:9!"#$#'*9+:%!"#$#'*9+:'!"#$#'*9+::!"#$#'*9+:)!"#$#'*9+:&!"#$#'*9+)*!"#$#'*9+)+!"#$#'*9+);!"#$#'*9+)(!"#$#'*9+)9!"#$#'*9+)%!"#$#'*9+)'!"#$#'*9+):!"#$#'*9+))!"#$#'*9+)&!"#$#'*9+&*

!"#$#'*9+&+!"#$#'*9+&;!"#$#'*9+&(!"#$#'*9+&9!"#$#'*9+&%!"#$#'*9+&'!"#$#'*9+&:!"#$#'*9+&)!"#$#'*9+&&!"#$#'*9;**!"#$#'*9;*+!"#$#'*9;*;!"#$#'*9;*(!"#$#'*9;*9!"#$#'*9;*%!"#$#'*9;*'!"#$#'*9;*:!"#$#'*9;*)!"#$#'*9;*&!"#$#'*9;+*!"#$#'*9;++!"#$#'*9;+;!"#$#'*9;+(!"#$#'*9;+9

!"#$#'*9;+%!"#$#'*9;+'!"#$#'*9;+:!"#$#'*9;+)!"#$#'*9;+&!"#$#'*9;;*!"#$#'*9;;+!"#$#'*9;;;!"#$#'*9;;(!"#$#'*9;;9!"#$#'*9;;%!"#$#'*9;;'!"#$#'*9;;:!"#$#'*9;;)!"#$#'*9;;&!"#$#'*9;(*!"#$#'*9;(+!"#$#'*9;(;!"#$#'*9;((!"#$#'*9;(9!"#$#'*9;(%!"#$#'*9;('!"#$#'*9;(:!"#$#'*9;()

!"#$#'*9;(&!"#$#'*9;9*!"#$#'*9;9+!"#$#'*9;9;!"#$#'*9;9(!"#$#'*9;99!"#$#'*9;9%!"#$#'*9;9'!"#$#'*9;9:!"#$#'*9;9)!"#$#'*9;9&!"#$#'*9;%*!"#$#'*9;%+!"#$#'*9;%;!"#$#'*9;%(!"#$#'*9;%9!"#$#'*9;%%!"#$#'*9;%'!"#$#'*9;%:!"#$#'*9;%)!"#$#'*9;%&!"#$#'*9;'*!"#$#'*9;'+!"#$#'*9;';

!"#$#'*9;'(!"#$#'*9;'9!"#$#'*9;'%!"#$#'*9;''!"#$#'*9;':!"#$#'*9;')!"#$#'*9;'&!"#$#'*9;:*!"#$#'*9;:+!"#$#'*9;:;!"#$#'*9;:(!"#$#'*9;:9!"#$#'*9;:%!"#$#'*9;:'!"#$#'*9;::!"#$#'*9;:)!"#$#'*9;:&!"#$#'*9;)*!"#$#'*9;)+!"#$#'*9;);!"#$#'*9;)(!"#$#'*9;)9!"#$#'*9;)%!"#$#'*9;)'

!"#$#'*9;):!"#$#'*9;))!"#$#'*9;)&!"#$#'*9;&*!"#$#'*9;&+!"#$#'*9;&;!"#$#'*9;&(!"#$#'*9;&9!"#$#'*9;&%!"#$#'*9;&'!"#$#'*9;&:!"#$#'*9;&)!"#$#'*9;&&!"#$#'*9(**!"#$#'*9(*+!"#$#'*9(*;!"#$#'*9(*(!"#$#'*9(*9!"#$#'*9(*%!"#$#'*9(*'!"#$#'*9(*:!"#$#'*9(*)!"#$#'*9(*&!"#$#'*9(+*

!"#$#'*9(++!"#$#'*9(+;!"#$#'*9(+(!"#$#'*9(+9!"#$#'*9(+%!"#$#'*9(+'!"#$#'*9(+:!"#$#'*9(+)!"#$#'*9(+&!"#$#'*9(;*!"#$#'*9(;+!"#$#'*9(;;!"#$#'*9(;(!"#$#'*9(;9!"#$#'*9(;%!"#$#'*9(;'!"#$#'*9(;:!"#$#'*9(;)!"#$#'*9(;&!"#$#'*9((*!"#$#'*9((+!"#$#'*9((;!"#$#'*9(((!"#$#'*9((9

!"#$#'*9((%!"#$#'*9(('!"#$#'*9((:!"#$#'*9(()!"#$#'*9((&!"#$#'*9(9*!"#$#'*9(9+!"#$#'*9(9;!"#$#'*9(9(!"#$#'*9(99!"#$#'*9(9%!"#$#'*9(9'!"#$#'*9(9:!"#$#'*9(9)!"#$#'*9(9&!"#$#'*9(%*!"#$#'*9(%+!"#$#'*9(%;!"#$#'*9(%(!"#$#'*9(%9!"#$#'*9(%%!"#$#'*9(%'!"#$#'*9(%:!"#$#'*9(%) !"#$#'*9(%&!"#$#'*9('*!"#$#'*9('+!"#$#'*9(';!"#$#'*9('(!"#$#'*9('9!"#$#'*9('%!"#$#'*9(''!"#$#'*9(':!"#$#'*9(')!"#$#'*9('&!"#$#'*9(:*!"#$#'*9(:+!"#$#'*9(:;!"#$#'*9(:(!"#$#'*9(:9!"#$#'*9(:%!"#$#'*9(:'!"#$#'*9(::!"#$#'*9(:)!"#$#'*9(:&!"#$#'*9()*!"#$#'*9()+!"#$#'*9(); !"#$#'*9()(!"#$#'*9()9!"#$#'*9()%!"#$#'*9()'!"#$#'*9():!"#$#'*9())!"#$#'*9()&!"#$#'*9(&*!"#$#'*9(&+!"#$#'*9(&;!"#$#'*9(&(!"#$#'*9(&9!"#$#'*9(&%!"#$#'*9(&'!"#$#'*9(&:!"#$#'*9(&)!"#$#'*9(&&!"#$#'*99**!"#$#'*99*+!"#$#'*99*;!"#$#'*99*(!"#$#'*99*9!"#$#'*99*%!"#$#'*99*'

!"#$#'*99*:!"#$#'*99*)!"#$#'*99*&!"#$#'*99+*!"#$#'*99++!"#$#'*99+;!"#$#'*99+(!"#$#'*99+9!"#$#'*99+%!"#$#'*99+'!"#$#'*99+:!"#$#'*99+)!"#$#'*99+&!"#$#'*99;*!"#$#'*99;+!"#$#'*99;;!"#$#'*99;(!"#$#'*99;9!"#$#'*99;%!"#$#'*99;'!"#$#'*99;:!"#$#'*99;)!"#$#'*99;&!"#$#'*99(*

!"#$#'*99(+!"#$#'*99(;!"#$#'*99((!"#$#'*99(9!"#$#'*99(%!"#$#'*99('!"#$#'*99(:!"#$#'*99()!"#$#'*99(&!"#$#'*999*!"#$#'*999+!"#$#'*999;!"#$#'*999(!"#$#'*9999!"#$#'*999%!"#$#'*999'!"#$#'*999:!"#$#'*999)!"#$#'*999&!"#$#'*99%*!"#$#'*99%+!"#$#'*99%;!"#$#'*99%(!"#$#'*99%9

!"#$#'*99%%!"#$#'*99%'!"#$#'*99%:!"#$#'*99%)!"#$#'*99%&!"#$#'*99'*!"#$#'*99'+!"#$#'*99';!"#$#'*99'(!"#$#'*99'9!"#$#'*99'%!"#$#'*99''!"#$#'*99':!"#$#'*99')!"#$#'*99'&!"#$#'*99:*!"#$#'*99:+!"#$#'*99:;!"#$#'*99:(!"#$#'*99:9!"#$#'*99:%!"#$#'*99:'!"#$#'*99::!"#$#'*99:) !"#$#'*99:&!"#$#'*99)*!"#$#'*99)+!"#$#'*99);!"#$#'*99)(!"#$#'*99)9!"#$#'*99)%!"#$#'*99)'!"#$#'*99):!"#$#'*99))!"#$#'*99)&!"#$#'*99&*!"#$#'*99&+!"#$#'*99&;!"#$#'*99&(!"#$#'*99&9!"#$#'*99&%!"#$#'*99&'!"#$#'*99&:!"#$#'*99&)!"#$#'*99&&!"#$#'*9%**!"#$#'*9%*+!"#$#'*9%*;

!"#$#'*9%*(!"#$#'*9%*9!"#$#'*9%*%!"#$#'*9%*'!"#$#'*9%*:!"#$#'*9%*)!"#$#'*9%*&!"#$#'*9%+*!"#$#'*9%++!"#$#'*9%+;!"#$#'*9%+(!"#$#'*9%+9!"#$#'*9%+%!"#$#'*9%+'!"#$#'*9%+:!"#$#'*9%+)!"#$#'*9%+&!"#$#'*9%;*!"#$#'*9%;+!"#$#'*9%;;!"#$#'*9%;(!"#$#'*9%;9!"#$#'*9%;%!"#$#'*9%;'

!"#$#'*9%;:!"#$#'*9%;)!"#$#'*9%;&!"#$#'*9%(*!"#$#'*9%(+!"#$#'*9%(;!"#$#'*9%((!"#$#'*9%(9!"#$#'*9%(%!"#$#'*9%('!"#$#'*9%(:!"#$#'*9%()!"#$#'*9%(&!"#$#'*9%9*!"#$#'*9%9+!"#$#'*9%9;!"#$#'*9%9(!"#$#'*9%99!"#$#'*9%9%!"#$#'*9%9'!"#$#'*9%9:!"#$#'*9%9)!"#$#'*9%9&!"#$#'*9%%*

!"#$#'*9%%+!"#$#'*9%%;!"#$#'*9%%(!"#$#'*9%%9!"#$#'*9%%%!"#$#'*9%%'!"#$#'*9%%:!"#$#'*9%%)!"#$#'*9%%&!"#$#'*9%'*!"#$#'*9%'+!"#$#'*9%';!"#$#'*9%'(!"#$#'*9%'9!"#$#'*9%'%!"#$#'*9%''!"#$#'*9%':!"#$#'*9%')!"#$#'*9%'&!"#$#'*9%:*!"#$#'*9%:+!"#$#'*9%:;!"#$#'*9%:(!"#$#'*9%:9

!"#$#'*9%:%!"#$#'*9%:'!"#$#'*9%::!"#$#'*9%:)!"#$#'*9%:&!"#$#'*9%)*!"#$#'*9%)+!"#$#'*9%);!"#$#'*9%)(!"#$#'*9%)9!"#$#'*9%)%!"#$#'*9%)'!"#$#'*9%):!"#$#'*9%))!"#$#'*9%)&!"#$#'*9%&*!"#$#'*9%&+!"#$#'*9%&;!"#$#'*9%&(!"#$#'*9%&9!"#$#'*9%&%!"#$#'*9%&'!"#$#'*9%&:!"#$#'*9%&)

!"#$#'*9%&&!"#$#'*9'**!"#$#'*9'*+!"#$#'*9'*;!"#$#'*9'*(!"#$#'*9'*9!"#$#'*9'*%!"#$#'*9'*'!"#$#'*9'*:!"#$#'*9'*)!"#$#'*9'*&!"#$#'*9'+*!"#$#'*9'++!"#$#'*9'+;!"#$#'*9'+(!"#$#'*9'+9!"#$#'*9'+%!"#$#'*9'+'!"#$#'*9'+:!"#$#'*9'+)!"#$#'*9'+&!"#$#'*9';*!"#$#'*9';+!"#$#'*9';;

!"#$#'*9';(!"#$#'*9';9!"#$#'*9';%!"#$#'*9';'!"#$#'*9';:!"#$#'*9';)!"#$#'*9';&!"#$#'*9'(*!"#$#'*9'(+!"#$#'*9'(;!"#$#'*9'((!"#$#'*9'(9!"#$#'*9'(%!"#$#'*9'('!"#$#'*9'(:!"#$#'*9'()!"#$#'*9'(&!"#$#'*9'9*!"#$#'*9'9+!"#$#'*9'9;!"#$#'*9'9(!"#$#'*9'99!"#$#'*9'9%!"#$#'*9'9'

!"#$#'*9'9:!"#$#'*9'9)!"#$#'*9'9&!"#$#'*9'%*!"#$#'*9'%+!"#$#'*9'%;!"#$#'*9'%(!"#$#'*9'%9!"#$#'*9'%%!"#$#'*9'%'!"#$#'*9'%:!"#$#'*9'%)!"#$#'*9'%&!"#$#'*9''*!"#$#'*9''+!"#$#'*9'';!"#$#'*9''(!"#$#'*9''9!"#$#'*9''%!"#$#'*9'''!"#$#'*9'':!"#$#'*9'')!"#$#'*9''&!"#$#'*9':*

!"#$#'*9':+!"#$#'*9':;!"#$#'*9':(!"#$#'*9':9!"#$#'*9':%!"#$#'*9':'!"#$#'*9'::!"#$#'*9':)!"#$#'*9':&!"#$#'*9')*!"#$#'*9')+!"#$#'*9');!"#$#'*9')(!"#$#'*9')9!"#$#'*9')%!"#$#'*9')'!"#$#'*9'):!"#$#'*9'))!"#$#'*9')&!"#$#'*9'&*!"#$#'*9'&+!"#$#'*9'&;!"#$#'*9'&(!"#$#'*9'&9

!"#$#'*9'&%!"#$#'*9'&'!"#$#'*9'&:!"#$#'*9'&)!"#$#'*9'&&!"#$#'*9:**!"#$#'*9:*+!"#$#'*9:*;!"#$#'*9:*(!"#$#'*9:*9!"#$#'*9:*%!"#$#'*9:*'!"#$#'*9:*:!"#$#'*9:*)!"#$#'*9:*&!"#$#'*9:+*!"#$#'*9:++!"#$#'*9:+;!"#$#'*9:+(!"#$#'*9:+9!"#$#'*9:+%!"#$#'*9:+'!"#$#'*9:+:!"#$#'*9:+)

!"#$#'*9:+&!"#$#'*9:;*!"#$#'*9:;+!"#$#'*9:;;!"#$#'*9:;(!"#$#'*9:;9!"#$#'*9:;%!"#$#'*9:;'!"#$#'*9:;:!"#$#'*9:;)!"#$#'*9:;&!"#$#'*9:(*!"#$#'*9:(+!"#$#'*9:(;!"#$#'*9:((!"#$#'*9:(9!"#$#'*9:(%!"#$#'*9:('!"#$#'*9:(:!"#$#'*9:()!"#$#'*9:(&!"#$#'*9:9*!"#$#'*9:9+!"#$#'*9:9; !"#$#'*9:9(!"#$#'*9:99!"#$#'*9:9%!"#$#'*9:9'!"#$#'*9:9:!"#$#'*9:9)!"#$#'*9:9&!"#$#'*9:%*!"#$#'*9:%+!"#$#'*9:%;!"#$#'*9:%(!"#$#'*9:%9!"#$#'*9:%%!"#$#'*9:%'!"#$#'*9:%:!"#$#'*9:%)!"#$#'*9:%&!"#$#'*9:'*!"#$#'*9:'+!"#$#'*9:';!"#$#'*9:'(!"#$#'*9:'9!"#$#'*9:'%!"#$#'*9:'' !"#$#'*9:':!"#$#'*9:')!"#$#'*9:'&!"#$#'*9::*!"#$#'*9::+!"#$#'*9::;!"#$#'*9::(!"#$#'*9::9!"#$#'*9::%!"#$#'*9::'!"#$#'*9:::!"#$#'*9::)!"#$#'*9::&!"#$#'*9:)*!"#$#'*9:)+!"#$#'*9:);!"#$#'*9:)(!"#$#'*9:)9!"#$#'*9:)%!"#$#'*9:)'!"#$#'*9:):!"#$#'*9:))!"#$#'*9:)&!"#$#'*9:&*

!"#$#'*9:&+!"#$#'*9:&;!"#$#'*9:&(!"#$#'*9:&9!"#$#'*9:&%!"#$#'*9:&'!"#$#'*9:&:!"#$#'*9:&)!"#$#'*9:&&!"#$#'*9)**!"#$#'*9)*+!"#$#'*9)*;!"#$#'*9)*(!"#$#'*9)*9!"#$#'*9)*%!"#$#'*9)*'!"#$#'*9)*:!"#$#'*9)*)!"#$#'*9)*&!"#$#'*9)+*!"#$#'*9)++!"#$#'*9)+;!"#$#'*9)+(!"#$#'*9)+9

!"#$#'*9)+%!"#$#'*9)+'!"#$#'*9)+:!"#$#'*9)+)!"#$#'*9)+&!"#$#'*9);*!"#$#'*9);+!"#$#'*9);;!"#$#'*9);(!"#$#'*9);9!"#$#'*9);%!"#$#'*9);'!"#$#'*9);:!"#$#'*9);)!"#$#'*9);&!"#$#'*9)(*!"#$#'*9)(+!"#$#'*9)(;!"#$#'*9)((!"#$#'*9)(9!"#$#'*9)(%!"#$#'*9)('!"#$#'*9)(:!"#$#'*9)()

!"#$#'*9)(&!"#$#'*9)9*!"#$#'*9)9+!"#$#'*9)9;!"#$#'*9)9(!"#$#'*9)99!"#$#'*9)9%!"#$#'*9)9'!"#$#'*9)9:!"#$#'*9)9)!"#$#'*9)9&!"#$#'*9)%*!"#$#'*9)%+!"#$#'*9)%;!"#$#'*9)%(!"#$#'*9)%9!"#$#'*9)%%!"#$#'*9)%'!"#$#'*9)%:!"#$#'*9)%)!"#$#'*9)%&!"#$#'*9)'*!"#$#'*9)'+!"#$#'*9)';

!"#$#'*9)'(!"#$#'*9)'9!"#$#'*9)'%!"#$#'*9)''!"#$#'*9)':!"#$#'*9)')!"#$#'*9)'&!"#$#'*9):*!"#$#'*9):+!"#$#'*9):;!"#$#'*9):(!"#$#'*9):9!"#$#'*9):%!"#$#'*9):'!"#$#'*9)::!"#$#'*9):)!"#$#'*9):&!"#$#'*9))*!"#$#'*9))+!"#$#'*9));!"#$#'*9))(!"#$#'*9))9!"#$#'*9))%!"#$#'*9))'

!"#$#'*9)):!"#$#'*9)))!"#$#'*9))&!"#$#'*9)&*!"#$#'*9)&+!"#$#'*9)&;!"#$#'*9)&(!"#$#'*9)&9!"#$#'*9)&%!"#$#'*9)&'!"#$#'*9)&:!"#$#'*9)&)!"#$#'*9)&&!"#$#'*9&**!"#$#'*9&*+!"#$#'*9&*;!"#$#'*9&*(!"#$#'*9&*9!"#$#'*9&*%!"#$#'*9&*'!"#$#'*9&*:!"#$#'*9&*)!"#$#'*9&*&!"#$#'*9&+*

!"#$#'*9&++!"#$#'*9&+;!"#$#'*9&+(!"#$#'*9&+9!"#$#'*9&+%!"#$#'*9&+'!"#$#'*9&+:!"#$#'*9&+)!"#$#'*9&+&!"#$#'*9&;*!"#$#'*9&;+!"#$#'*9&;;!"#$#'*9&;(!"#$#'*9&;9!"#$#'*9&;%!"#$#'*9&;'!"#$#'*9&;:!"#$#'*9&;)!"#$#'*9&;&!"#$#'*9&(*!"#$#'*9&(+!"#$#'*9&(;!"#$#'*9&((!"#$#'*9&(9 !"#$#'*9&(%!"#$#'*9&('!"#$#'*9&(:!"#$#'*9&()!"#$#'*9&(&!"#$#'*9&9*!"#$#'*9&9+!"#$#'*9&9;!"#$#'*9&9(!"#$#'*9&99!"#$#'*9&9%!"#$#'*9&9'!"#$#'*9&9:!"#$#'*9&9)!"#$#'*9&9&!"#$#'*9&%*!"#$#'*9&%+!"#$#'*9&%;!"#$#'*9&%(!"#$#'*9&%9!"#$#'*9&%%!"#$#'*9&%'!"#$#'*9&%:!"#$#'*9&%)

!"#$#'*9&%&!"#$#'*9&'*!"#$#'*9&'+!"#$#'*9&';!"#$#'*9&'(!"#$#'*9&'9!"#$#'*9&'%!"#$#'*9&''!"#$#'*9&':!"#$#'*9&')!"#$#'*9&'&!"#$#'*9&:*!"#$#'*9&:+!"#$#'*9&:;!"#$#'*9&:(!"#$#'*9&:9!"#$#'*9&:%!"#$#'*9&:'!"#$#'*9&::!"#$#'*9&:)!"#$#'*9&:&!"#$#'*9&)*!"#$#'*9&)+!"#$#'*9&);

!"#$#'*9&)(!"#$#'*9&)9!"#$#'*9&)%!"#$#'*9&)'!"#$#'*9&):!"#$#'*9&))!"#$#'*9&)&!"#$#'*9&&*!"#$#'*9&&+!"#$#'*9&&;!"#$#'*9&&(!"#$#'*9&&9!"#$#'*9&&%!"#$#'*9&&'!"#$#'*9&&:!"#$#'*9&&)!"#$#'*9&&&!"#$#'*%***!"#$#'*%**+!"#$#'*%**;!"#$#'*%**(!"#$#'*%**9!"#$#'*%**%!"#$#'*%**' !"#$#'*%**:!"#$#'*%**)!"#$#'*%**&!"#$#'*%*+*!"#$#'*%*++!"#$#'*%*+;!"#$#'*%*+(!"#$#'*%*+9!"#$#'*%*+%!"#$#'*%*+'!"#$#'*%*+:!"#$#'*%*+)!"#$#'*%*+&!"#$#'*%*;*!"#$#'*%*;+!"#$#'*%*;;!"#$#'*%*;(!"#$#'*%*;9!"#$#'*%*;%!"#$#'*%*;'!"#$#'*%*;:!"#$#'*%*;)!"#$#'*%*;&!"#$#'*%*(* !"#$#'*%*(+!"#$#'*%*(;!"#$#'*%*((!"#$#'*%*(9!"#$#'*%*(%!"#$#'*%*('!"#$#'*%*(:!"#$#'*%*()!"#$#'*%*(&!"#$#'*%*9*!"#$#'*%*9+!"#$#'*%*9;!"#$#'*%*9(!"#$#'*%*99!"#$#'*%*9%!"#$#'*%*9'!"#$#'*%*9:!"#$#'*%*9)!"#$#'*%*9&!"#$#'*%*%*!"#$#'*%*%+!"#$#'*%*%;!"#$#'*%*%(!"#$#'*%*%9 !"#$#'*%*%%!"#$#'*%*%'!"#$#'*%*%:

@ @ 4 5 64 3 1 $6 ! 5 3 $ 8 , 4 $,

!"#$#'*%*%)!"#$#'*%*%&!"#$#'*%*'*!"#$#'*%*'+!"#$#'*%*';!"#$#'*%*'(!"#$#'*%*'9!"#$#'*%*'%!"#$#'*%*''!"#$#'*%*':!"#$#'*%*')!"#$#'*%*'&!"#$#'*%*:*!"#$#'*%*:+!"#$#'*%*:;!"#$#'*%*:(!"#$#'*%*:9!"#$#'*%*:%!"#$#'*%*:'!"#$#'*%*:)!"#$#'*%*:&!"#$#'*%*)*!"#$#'*%*)+!"#$#'*%*); !"#$#'*%*)(!"#$#'*%*)9!"#$#'*%*)%!"#$#'*%*)'!"#$#'*%*))!"#$#'*%*)&!"#$#'*%*&*!"#$#'*%*&;!"#$#'*%*&(!"#$#'*%*&9!"#$#'*%*&%!"#$#'*%*&:!"#$#'*%*&&!"#$#'*%+**!"#$#'*%+*+!"#$#'*%+*;!"#$#'*%+*(!"#$#'*%+*9!"#$#'*%+*%!"#$#'*%+*'!"#$#'*%+*:!"#$#'*%+*)!"#$#'*%+*&!"#$#'*%+++

!"#$#'*%++;!"#$#'*%++9!"#$#'*%++%!"#$#'*%++'!"#$#'*%++)!"#$#'*%++&!"#$#'*%+;*!"#$#'*%+;+!"#$#'*%+;;!"#$#'*%+;(!"#$#'*%+;9!"#$#'*%+;%!"#$#'*%+;'!"#$#'*%+;:!"#$#'*%+;)!"#$#'*%+(*!"#$#'*%+(;!"#$#'*%+((!"#$#'*%+(9!"#$#'*%+(%!"#$#'*%+('!"#$#'*%+(&!"#$#'*%+9*!"#$#'*%+9+

!"#$#'*%+9;!"#$#'*%+9(!"#$#'*%+99!"#$#'*%+9%!"#$#'*%+9'

|  | - A 4$ | . B . =5 < | . ..-" |
| --- | --- | --- | --- |
| !"#$#'*%+9: | ?3 5 $ | ?3 5  $ | ! 3 =. "" 4 /3" |

!"#$#'*%+9)!"#$#'*%+%*!"#$#'*%+%+!"#$#'*%+%(!"#$#'*%+%%!"#$#'*%+%'!"#$#'*%+%:!"#$#'*%+%)!"#$#'*%+%&!"#$#'*%+'*!"#$#'*%+'+!"#$#'*%+';!"#$#'*%+'9!"#$#'*%+'%!"#$#'*%+''!"#$#'*%+:*!"#$#'*%+:+!"#$#'*%+:;!"#$#'*%+:(!"#$#'*%+:'!"#$#'*%+::!"#$#'*%+)*!"#$#'*%+)+!"#$#'*%+); !"#$#'*%+)(!"#$#'*%+)9!"#$#'*%+)'!"#$#'*%+):!"#$#'*%+))!"#$#'*%+&;!"#$#'*%+&(!"#$#'*%+&9!"#$#'*%+&:!"#$#'*%+&)!"#$#'*%+&&!"#$#'*%;**!"#$#'*%;*+!"#$#'*%;*;!"#$#'*%;*9!"#$#'*%;*%!"#$#'*%;*'!"#$#'*%;*:!"#$#'*%;*)!"#$#'*%;*&!"#$#'*%;+*!"#$#'*%;++!"#$#'*%;+;!"#$#'*%;+(

!"#$#'*%;+%!"#$#'*%;+'!"#$#'*%;+:!"#$#'*%;+)!"#$#'*%;;*!"#$#'*%;;+!"#$#'*%;;(!"#$#'*%;;%!"#$#'*%;;'!"#$#'*%;;:!"#$#'*%;;&!"#$#'*%;(*!"#$#'*%;(+!"#$#'*%;(;!"#$#'*%;(9!"#$#'*%;(%!"#$#'*%;('!"#$#'*%;(:!"#$#'*%;()!"#$#'*%;(&!"#$#'*%;9*!"#$#'*%;9+!"#$#'*%;9;!"#$#'*%;9(

!"#$#'*%;99!"#$#'*%;9%!"#$#'*%;9:!"#$#'*%;9)!"#$#'*%;9&!"#$#'*%;%*!"#$#'*%;%+!"#$#'*%;%;!"#$#'*%;%(!"#$#'*%;%9!"#$#'*%;%%!"#$#'*%;%'!"#$#'*%;%:!"#$#'*%;%)!"#$#'*%;%&!"#$#'*%;'*!"#$#'*%;'+!"#$#'*%;';!"#$#'*%;'(!"#$#'*%;'9!"#$#'*%;'%!"#$#'*%;''!"#$#'*%;':!"#$#'*%;')

!"#$#'*%;'&!"#$#'*%;:*!"#$#'*%;:+!"#$#'*%;:(!"#$#'*%;:9!"#$#'*%;:%!"#$#'*%;:'!"#$#'*%;::!"#$#'*%;:)!"#$#'*%;:&!"#$#'*%;)*!"#$#'*%;)+!"#$#'*%;);!"#$#'*%;)(!"#$#'*%;)9!"#$#'*%;)%!"#$#'*%;)'!"#$#'*%;):!"#$#'*%;))!"#$#'*%;)&!"#$#'*%;&*!"#$#'*%;&+!"#$#'*%;&;!"#$#'*%;&(

!"#$#'*%;&9!"#$#'*%;&%!"#$#'*%;&'!"#$#'*%;&:!"#$#'*%;&)!"#$#'*%(**!"#$#'*%(*+!"#$#'*%(*;!"#$#'*%(*(!"#$#'*%(*9!"#$#'*%(*%!"#$#'*%(*'!"#$#'*%(*:!"#$#'*%(*)!"#$#'*%(*&!"#$#'*%(+*!"#$#'*%(++!"#$#'*%(+;!"#$#'*%(+(!"#$#'*%(+9!"#$#'*%(+%!"#$#'*%(+'!"#$#'*%(+:!"#$#'*%(+)

!"#$#'*%(+&!"#$#'*%(;*!"#$#'*%(;+!"#$#'*%(;;!"#$#'*%(;(!"#$#'*%(;9!"#$#'*%(;%!"#$#'*%(;'!"#$#'*%(;:!"#$#'*%(;)!"#$#'*%((+!"#$#'*%((;!"#$#'*%((%!"#$#'*%(('!"#$#'*%((:!"#$#'*%(()!"#$#'*%((&!"#$#'*%(9+!"#$#'*%(9(!"#$#'*%(99!"#$#'*%(9%!"#$#'*%(9:!"#$#'*%(9)!"#$#'*%(9&

!"#$#'*%(%*!"#$#'*%(%+!"#$#'*%(%;!"#$#'*%(%9!"#$#'*%(%%!"#$#'*%(%'!"#$#'*%(%:!"#$#'*%(%)!"#$#'*%(%&!"#$#'*%('*!"#$#'*%('+!"#$#'*%(';!"#$#'*%('(!"#$#'*%('9!"#$#'*%('%!"#$#'*%(''!"#$#'*%(':!"#$#'*%(')!"#$#'*%('&!"#$#'*%(:*!"#$#'*%(:;!"#$#'*%(:(!"#$#'*%(:9!"#$#'*%(:%

!"#$#'*%(:'!"#$#'*%(::!"#$#'*%(:)!"#$#'*%(:&!"#$#'*%()*!"#$#'*%();!"#$#'*%()(!"#$#'*%()9!"#$#'*%()%!"#$#'*%()'!"#$#'*%():!"#$#'*%())!"#$#'*%()&!"#$#'*%(&*!"#$#'*%(&+!"#$#'*%(&;!"#$#'*%(&(!"#$#'*%(&9!"#$#'*%(&%!"#$#'*%(&'!"#$#'*%(&:!"#$#'*%(&)!"#$#'*%(&&!"#$#'*%9** !"#$#'*%9*+!"#$#'*%9*;!"#$#'*%9*(

?" 5 $ ?" 5 $ !$ 8 6 7 3 7 "! 3 3 01 3 4 5

!"#$#'*%9*%!"#$#'*%9*'!"#$#'*%9*:!"#$#'*%9*)!"#$#'*%9*&!"#$#'*%9+*!"#$#'*%9++!"#$#'*%9+;!"#$#'*%9+(!"#$#'*%9+9!"#$#'*%9+%!"#$#'*%9+'!"#$#'*%9+:!"#$#'*%9+)!"#$#'*%9+&!"#$#'*%9;*!"#$#'*%9;+!"#$#'*%9;;!"#$#'*%9;(!"#$#'*%9;9!"#$#'*%9;%!"#$#'*%9;'!"#$#'*%9;:!"#$#'*%9;) !"#$#'*%9;&!"#$#'*%9(*!"#$#'*%9(+!"#$#'*%9(;!"#$#'*%9((!"#$#'*%9(9!"#$#'*%9(%!"#$#'*%9('!"#$#'*%9(:!"#$#'*%9()!"#$#'*%9(&!"#$#'*%99*!"#$#'*%99+!"#$#'*%99;!"#$#'*%99(!"#$#'*%999!"#$#'*%99%!"#$#'*%99'!"#$#'*%99:!"#$#'*%99)!"#$#'*%99&!"#$#'*%9%*!"#$#'*%9%+!"#$#'*%9%;

!"#$#'*%9%9!"#$#'*%9%%!"#$#'*%9%'!"#$#'*%9%:!"#$#'*%9%)!"#$#'*%9%&!"#$#'*%9'*!"#$#'*%9'+!"#$#'*%9';!"#$#'*%9'(!"#$#'*%9'9!"#$#'*%9'%!"#$#'*%9''!"#$#'*%9':!"#$#'*%9')!"#$#'*%9'&!"#$#'*%9:*!"#$#'*%9:+!"#$#'*%9:;!"#$#'*%9:(!"#$#'*%9:9!"#$#'*%9:%!"#$#'*%9:'!"#$#'*%9::

!"#$#'*%9:)!"#$#'*%9:&!"#$#'*%9)*!"#$#'*%9)+!"#$#'*%9);!"#$#'*%9)(!"#$#'*%9)9!"#$#'*%9)%!"#$#'*%9)'!"#$#'*%9):!"#$#'*%9))!"#$#'*%9)&!"#$#'*%9&*!"#$#'*%9&+!"#$#'*%9&;!"#$#'*%9&(!"#$#'*%9&9!"#$#'*%9&%!"#$#'*%9&'!"#$#'*%9&:!"#$#'*%9&)!"#$#'*%9&&!"#$#'*%%**!"#$#'*%%*+

!"#$#'*%%*;!"#$#'*%%*(!"#$#'*%%*%!"#$#'*%%*'!"#$#'*%%*:!"#$#'*%%*)!"#$#'*%%*&!"#$#'*%%+*!"#$#'*%%++!"#$#'*%%+;!"#$#'*%%+(!"#$#'*%%+9!"#$#'*%%+%!"#$#'*%%+'!"#$#'*%%+:!"#$#'*%%+)!"#$#'*%%+&!"#$#'*%%;*!"#$#'*%%;+!"#$#'*%%;;!"#$#'*%%;(!"#$#'*%%;9!"#$#'*%%;%!"#$#'*%%;'

!"#$#'*%%;:!"#$#'*%%;)!"#$#'*%%;&!"#$#'*%%(*!"#$#'*%%(+!"#$#'*%%(;!"#$#'*%%((!"#$#'*%%(9!"#$#'*%%(%!"#$#'*%%('!"#$#'*%%(:!"#$#'*%%()!"#$#'*%%(&!"#$#'*%%9*!"#$#'*%%9+!"#$#'*%%9;!"#$#'*%%9(!"#$#'*%%99!"#$#'*%%9%!"#$#'*%%9'!"#$#'*%%9:!"#$#'*%%9)!"#$#'*%%9&!"#$#'*%%%*

!"#$#'*%%%+!"#$#'*%%%;!"#$#'*%%%9!"#$#'*%%%%!"#$#'*%%%'!"#$#'*%%%:!"#$#'*%%%)!"#$#'*%%%&!"#$#'*%%'*!"#$#'*%%'+!"#$#'*%%';!"#$#'*%%'(!"#$#'*%%'9!"#$#'*%%'%!"#$#'*%%''!"#$#'*%%':!"#$#'*%%')!"#$#'*%%'&!"#$#'*%%:*!"#$#'*%%:+!"#$#'*%%:;!"#$#'*%%:(!"#$#'*%%:9!"#$#'*%%:%

!"#$#'*%%:'!"#$#'*%%::!"#$#'*%%:)!"#$#'*%%:&!"#$#'*%%)*!"#$#'*%%)+!"#$#'*%%);!"#$#'*%%)(!"#$#'*%%)9!"#$#'*%%)%!"#$#'*%%)'!"#$#'*%%):!"#$#'*%%))!"#$#'*%%)&!"#$#'*%%&*!"#$#'*%%&+!"#$#'*%%&;!"#$#'*%%&(!"#$#'*%%&9!"#$#'*%%&%!"#$#'*%%&'!"#$#'*%%&)!"#$#'*%%&&!"#$#'*%'**

!"#$#'*%'*+!"#$#'*%'*;!"#$#'*%'*(!"#$#'*%'*9!"#$#'*%'*%!"#$#'*%'*'!"#$#'*%'*:!"#$#'*%'*)!"#$#'*%'*&!"#$#'*%'+*!"#$#'*%'++!"#$#'*%'+;!"#$#'*%'+(!"#$#'*%'+9!"#$#'*%'+%!"#$#'*%'+'!"#$#'*%'+:!"#$#'*%'+)!"#$#'*%'+&!"#$#'*%';*!"#$#'*%';+!"#$#'*%';;!"#$#'*%';(!"#$#'*%';9

!"#$#'*%';%!"#$#'*%';'!"#$#'*%';:!"#$#'*%';)!"#$#'*%';&!"#$#'*%'(*!"#$#'*%'(+!"#$#'*%'(;!"#$#'*%'((!"#$#'*%'(9!"#$#'*%'(%!"#$#'*%'('!"#$#'*%'(:!"#$#'*%'()!"#$#'*%'(&!"#$#'*%'9*!"#$#'*%'9+!"#$#'*%'9;!"#$#'*%'9(!"#$#'*%'99!"#$#'*%'9%!"#$#'*%'9'!"#$#'*%'9:!"#$#'*%'9)

!"#$#'*%'9&!"#$#'*%'%*!"#$#'*%'%+!"#$#'*%'%;!"#$#'*%'%(!"#$#'*%'%9!"#$#'*%'%%!"#$#'*%'%'!"#$#'*%'%:!"#$#'*%'%)!"#$#'*%'%&!"#$#'*%''*!"#$#'*%''+!"#$#'*%'';!"#$#'*%''(!"#$#'*%''9!"#$#'*%''%!"#$#'*%'''!"#$#'*%'':!"#$#'*%'')!"#$#'*%''&!"#$#'*%':*!"#$#'*%':+!"#$#'*%':;

!"#$#'*%':(!"#$#'*%':9!"#$#'*%':%!"#$#'*%':'!"#$#'*%'::!"#$#'*%':)!"#$#'*%':&!"#$#'*%')*!"#$#'*%')+!"#$#'*%');!"#$#'*%')(!"#$#'*%')9!"#$#'*%')%!"#$#'*%')'!"#$#'*%'):!"#$#'*%'))!"#$#'*%')&!"#$#'*%'&*!"#$#'*%'&+!"#$#'*%'&(!"#$#'*%'&9!"#$#'*%'&%!"#$#'*%'&'!"#$#'*%'&:

!"#$#'*%'&)!"#$#'*%'&&!"#$#'*%:**!"#$#'*%:*+!"#$#'*%:*;!"#$#'*%:*(!"#$#'*%:*9!"#$#'*%:*%!"#$#'*%:*'!"#$#'*%:*:!"#$#'*%:*)!"#$#'*%:*&!"#$#'*%:+*!"#$#'*%:++!"#$#'*%:+;!"#$#'*%:+(!"#$#'*%:+9!"#$#'*%:+%!"#$#'*%:+'!"#$#'*%:+:!"#$#'*%:+)!"#$#'*%:+&!"#$#'*%:;*!"#$#'*%:;+

!"#$#'*%:;;!"#$#'*%:;(!"#$#'*%:;9!"#$#'*%:;%!"#$#'*%:;'!"#$#'*%:;:!"#$#'*%:;)!"#$#'*%:;&!"#$#'*%:(*!"#$#'*%:(+!"#$#'*%:(;!"#$#'*%:((!"#$#'*%:(9!"#$#'*%:(%!"#$#'*%:('!"#$#'*%:(:!"#$#'*%:()!"#$#'*%:(&!"#$#'*%:9*!"#$#'*%:9+!"#$#'*%:9;!"#$#'*%:9(!"#$#'*%:9%!"#$#'*%:9'

!"#$#'*%:9:!"#$#'*%:9)!"#$#'*%:9&!"#$#'*%:%*!"#$#'*%:%+!"#$#'*%:%;!"#$#'*%:%(!"#$#'*%:%9!"#$#'*%:%%!"#$#'*%:%'!"#$#'*%:%:!"#$#'*%:%)!"#$#'*%:%&!"#$#'*%:'*!"#$#'*%:'+!"#$#'*%:';!"#$#'*%:'(!"#$#'*%:'9!"#$#'*%:'%!"#$#'*%:''!"#$#'*%:':!"#$#'*%:')!"#$#'*%:'&!"#$#'*%::*

!"#$#'*%::+!"#$#'*%::;!"#$#'*%::(!"#$#'*%::9!"#$#'*%::%!"#$#'*%::'!"#$#'*%:::!"#$#'*%::)!"#$#'*%::&

?=3A4 ?=3A4 361 A4$

$ $

!"#$#'*%:)*!"#$#'*%:)+!"#$#'*%:); .!4".. 0 . .!4".. 0 4 5 6 ! - 6 3 - ! =7 ! =4 6 3 3 3 3 5 38 6 3 - ! =3

!< /4 E.!4H .!< /4 ! = - 3 4 6 3

E.!4H

!"#$#'+*+'(!"#$#'+*+'9!"#$#'+*+'%!"#$#'+*+''!"#$#'+*+':!"#$#'+*+')!"#$#'+*+'&!"#$#'+*+:*!"#$#'+*+:+!"#$#'+*+:;!"#$#'+*+:(!"#$#'+*+:9!"#$#'+*+:%!"#$#'+*+:'!"#$#'+*+::!"#$#'+*+:)!"#$#'+*+:&!"#$#'+*+)*!"#$#'+*+)+!"#$#'+*+);!"#$#'+*+)(!"#$#'+*+)9!"#$#'+*+)%!"#$#'+*+)' !"#$#'+*+):!"#$#'+*+))!"#$#'+*+)&!"#$#'+*+&*!"#$#'+*+&+!"#$#'+*+&;!"#$#'+*+&(!"#$#'+*+&9!"#$#'+*+&%!"#$#'+*+&:!"#$#'+*+&)!"#$#'+*+&&!"#$#'+*;**!"#$#'+*;*+!"#$#'+*;*;!"#$#'+*;*(!"#$#'+*;*9!"#$#'+*;*%!"#$#'+*;*'!"#$#'+*;*:!"#$#'+*;*)!"#$#'+*;*&!"#$#'+*;+*!"#$#'+*;++ !"#$#'+*;+;!"#$#'+*;+(!"#$#'+*;+9!"#$#'+*;+%!"#$#'+*;+'!"#$#'+*;+:!"#$#'+*;+)!"#$#'+*;+&!"#$#'+*;;*!"#$#'+*;;+!"#$#'+*;;;!"#$#'+*;;(!"#$#'+*;;9!"#$#'+*;;%

5 7 756 5 7 7 6 =5 $$ =6 $ 6 = $ 0 =$ -7 7 =$ .. 7 =3 76 6 = $5 =8 6 =$ 8 3 . =8 $8 =3 , 6 =. . 6 =8 1 6 =. 7 =6 $ 6 5 8 =

" ? 56" ? 3 @ 7 =$ 86 =7 8 . 5 =6 8 "


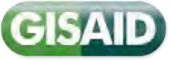


|  |  | | |  |  |
| --- | --- | --- | --- | --- | --- |
| !"#$#%&'%() | * + , - - | | | .* /- | " 0,"1" 1 /2 2 3 2 " $"4 1 0"2 +5.2 " +/ 3" , /6,27* -+,+ |
| !"#$#%&'%(8 | * + , - - | | | .* /- | ,"1" 1 /2 2 3 2 " $"4 1 0"2 +5.2 " +/ 3" , " 0/6,27* -+,+ |
| !"#$#%&'%(% | * + , - - | | | .* /- | 1" 1 /2 2 3 2 " $"4 1 0"2 +5.2 " +/ 3 " , " 0,"/6,27* -+,+ |
| !"#$#%&'%(' | * + , - - | | | .* /- | 1 /2 2 3 2 " $"4 1 0"2 +5.2 " +/ 3" , " 0,"1" /6,27* -+,+ |
| !"#$#%&'%(9 | * + , - - | | | .* /- | /2 2 3 2 " $"4 1 0"2 +5.2 " +/ 3" , " 0,"1" 1 /6,27* -+,+ |
| !"#$#%&'%(& | * + , - - | | | .* /- | 2 3 2 " $"4 1 0"2 +5.2 " +/ 3" , " 0, "1" 1 /2 /6,27* -+,+ |
| !"#$#%&'%(: | * + , - - | | | .* /- | 3 2 " $"4 1 0"2 +5.2 " +/ 3" , " 0,"1 " 1 /2 2 /6,27* -+,+ |
| !"#$#%&'%;< | * + , - - | | | .* /- | 2 " $"4 1 0"2 +5.2 " +/ 3" , " 0,"1" 1 /2 2 3 /6,27* -+,+ |
| !"#$#%&'%;( | * + , - - | | | .* /- | $"4 1 0"2 +5.2 " +/ 3" , " 0,"1" 1  /2 2 3 2 " /6,27* -+,+ |
| !"#$#%&'%;; | * + , - - | | | .* /- | 4 1 0"2 +5.2 " +/ 3" , " 0,"1" 1 /2 2 3 2 " $"/6,27* -+,+ |
| !"#$#%&'%;) | * + , - - | | | .* /- | 1 0"2 +5.2 " +/ 3" , " 0,"1" 1 /2 2 3 2 " $"4 /6,27* -+,+ |
| !"#$#%&'%;8 | * + , - - | | | .* /- | "2 +5.2 " +/ 3" , " 0,"1" 1 /2 2 3 2 " $"4 1 0/6,27* -+,+ |
| !"#$#%&'%;% | * + , - - | | | .* /- | 2 +5.2 " +/ 3" , " 0,"1" 1 /2 2 3 2 " $"4 1 0"/6,27* -+,+ |
| !"#$#%&'%;' | * + , - - | | | .* /- | .2 " +/ 3" , " 0,"1" 1 /2 2 3 2 " $"4 1 0"2 +5/6,27* -+,+ |
| !"#$#%&'%;9 | * + , - - | | | .* /- | " +/ 3" , " 0,"1" 1 /2 2 3 2 " $"4 1 0"2 +5.2/6,27* -+,+ |
| !"#$#%&'%;& | * + , - - | | | .* /- | 3" , " 0,"1" 1 /2 2 3 2 " $"4 1 0"2 + 5.2 " +/ /6,27* -+,+ |
| !"#$#%&'%;: | * + , - - | | | .* /- | , " 0,"1" 1 /2 2 3 2 " $"4 1 0"2 +5 .2 " +/ 3" /6,27* -+,+ |
| !"#$#%&'%)< | * + , - - | | | .* /- | " 0,"1" 1 /2 2 3 2 " $"4 1 0"2 +5.2 " +/ 3" , /6,27* -+,+ |
| !"#$#%&'%)( | 7 - | | | .* /- | ="0 ","1" 1 /2 2 3 2 " $"4 1 0 " + / 3" , " 0/6,27* -+,+ |
| !"#$#%&'%); | 7 - | | | .* /- | 0 ","1" 1 /2 2 3 2 " $"4 1 0 " +/ 3 " , " 0="/6,27* -+,+ |
| !"#$#%&'%)) | 7 - | | | .* /- | ","1" 1 /2 2 3 2 " $"4 1 0 " +/ 3" , " 0="0 /6,27* -+,+ |
| !"#$#%&'%)8 | 7 - | | | .* /- | ,"1" 1 /2 2 3 2 " $"4 1 0 " +/ 3" , " 0="0 "/6,27* -+,+ |
| !"#$#%&'%)% | 7 - | | | .* /- | 1" 1 /2 2 3 2 " $"4 1 0 " +/ 3" , " 0=" 0 ","/6,27* -+,+ |
| !"#$#%&'%)' | 3 , 7 | | | .* /- | 2 $,1 /2 2 3 2 " $"4 1 0 " +/ 3" , " 0,"1" /6,27* -+,+ |
| !"#$#%&'%)9 | 3 , 7 | | | .* /- | ,1 /2 2 3 2 " $"4 1 0 " +/ 3" , " 0, "1" 2 $/6,27* -+,+ |
| !"#$#%&'%)& | 3 , 7 | | | .* /- | 1 /2 2 3 2 " $"4 1 0 " +/ 3" , " 0,"1 " 2 $,/6,27* -+,+ |
| !"#$#%&'%): | 3 , 7 | | | .* /- | /2 2 3 2 " $"4 1 0 " +/ 3" , " 0,"1" 2 $,1 /6,27* -+,+ |
| !"#$#%&'%8< | 3 , 7 | | | .* /- | 2 3 2 " $"4 1 0 " +/ 3" , " 0,"1" 2 $,1 /2 /6,27* -+,+ |
| !"#$#%&'%8( | 3 , 7 | | | .* /- | 3 2 " $"4 1 0 " +/ 3" , " 0,"1" 2 $,1 /2 2 /6,27* -+,+ |
| !"#$#%&'%8; | 7 +2 | | | .* /- | 2 " $"4 1 07, " +/ 3" , " 0,"1" 1 /2 2 3 /6,27* -+,+ |
| !"#$#%&'%8) | 7 +2 | | | .* /- | $"4 1 07, " +/ 3" , " 0,"1" 1 /2 2 3 2 " /6,27* -+,+ |
| !"#$#%&'%88 | 7 +2 | | | .* /- | 4 1 07, " +/ 3" , " 0,"1" 1 /2 2 3 2 " $"/6,27* -+,+ |
| !"#$#%&'%8% | 7 +2 | | | .* /- | 1 07, " +/ 3" , " 0,"1" 1 /2 2 3 2 " $ "4 /6,27* -+,+ |
| !"#$#%&'%8' | 7 | | | .* /- | 1 2" 2 " +/ 3" , " 0,"1" 1 /2 2 3 2 " $"4 1 0/6,27* -+,+ |
| !"#$#%&'%89 | 7 | | | .* /- | " 2 " +/ 3" , " 0,"1" 1 /2 2 3 2 " $ "4 1 01 2/6,27* -+,+ |
| !"#$#%&'%8& | 7 | | | .* /- | 2 " +/ 3" , " 0,"1" 1 /2 2 3 2 " $"4 1 01 2" /6,27* -+,+ |
| !"#$#%&'%8: | ---> | | | .* /- | +"+* " +/ 3" , " 0,"1" 1 /2 2 3 2 " $"4 1 0/6,27* -+,+ |
| !"#$#%&'%%< | ---> | | | .* /- | +* " +/ 3" , " 0,"1" 1 /2 2 3 2 " $"4 1 0+"/6,27* -+,+ |
| !"#$#%&'%%( | 7 > | | | .* /- | , " 0,"1" 1 /2 2 3 2 " $"4 1 0+"+* " +/ 3" /6,27* -+,+ |
| !"#$#%&'%%; | | | 7 > | .* /- | | " 0,"1" 1 /2 2 3 2 " $"4 1 0+"+* " +/ 3" , /6,27* -+,+ |
| !"#$#%&'%%) | | | 7 > | .* /- | | ,"1" 1 /2 2 3 2 " $"4 1 0+"+* " +/ 3" , " 0/6,27* -+,+ |
| !"#$#%&'%%8 | | | 7 > | .* /- | | 1" 1 /2 2 3 2 " $"4 1 0+"+* " +/ 3" , " 0,"/6,27* -+,+ |
| !"#$#%&'%%% | | | ---> | .* /- | | 1 /2 2 3 2 " $"4 1 0+"+* " +/ 3" , " 0,"1" /6,27* -+,+ |
| !"#$#%&'%%' | | | ---> | .* /- | | /2 2 3 2 " $"4 1 0+"+* " +/ 3" , " 0 ,"1" 1 /6,27* -+,+ |
| !"#$#%&'%%9 | | | ---> | .* /- | | 2 3 2 " $"4 1 0+"+* " +/ 3" , " 0,"1" 1 /2 /6,27* -+,+ |
| !"#$#%&'%%& | | | ---> | .* /- | | 3 2 " $"4 1 0+"+* " +/ 3" , " 0,"1" 1 /2 2 /6,27* -+,+ |
| !"#$#%&'%%: | | | ---> | .* /- | | 2 " $"4 1 0+"+* " +/ 3" , " 0,"1" 1 / 2 2 3 /6,27* -+,+ |
| !"#$#%&'%'< | | | ---> | .* /- | | $"4 1 0+"+* " +/ 3" , " 0,"1" 1 /2 2 3 2 " /6,27* -+,+ |
| !"#$#%&'%'( | | | ---> | .* /- | | 4 1 0+"+* " +/ 3" , " 0,"1" 1 /2 2 3 2 " $"/6,27* -+,+ |
| !"#$#%&'%'; | | | ,!/,!/, - 7 + | .* /- | | 1 0**/ " +/ 3" , " 0,"1" 1 /2 2 3 2 " $"4 /6,27* -+,+ |
| !"#$#%&'%') | | | ,!/,!/, - 7 + | .* /- | | **/ " +/ 3" , " 0,"1" 1 /2 2 3 2 " $ "4 1 0/6,27* -+,+ |
| !"#$#%&'%'8 | | | ,!/,!/, - 7 + | .* /- | | / " +/ 3" , " 0,"1" 1 /2 2 3 2 " $"4 1 0**/6,27* -+,+ |
| !"#$#%&'%'% | | | ,!/,!/, - 7 + | .* /- | | " +/ 3" , " 0,"1" 1 /2 2 3 2 " $"4 1 0**//6,27* -+,+ |
| !"#$#%&'%'' | | | ,!/,!/, - 7 + | .* /- | | +/ 3" , " 0,"1" 1 /2 2 3 2 " $"4 1 0** / " /6,27* -+,+ |
| !"#$#%&'%'9!"#$#%&'%'& | | | 7 , | .* /- | | 3" , " 0,"1" 1 /2 2 3 2 " $"4 1 07" " +/ /6,27* -+,+ |
| !"#$#%&'%': | | | -/ | .* /- | | "7" " +/ 3" , " 0,"1" 1 /2 2 3 2 " $"4 1 0/6,27* -+,+ |
| !"#$#%&'%9< | | | -/ | .* /- | | 7" " +/ 3" , " 0,"1" 1 /2 2 3 2 " $"4 1 0 "/6,27* -+,+ |
| !"#$#%&'%9( | | | > > ?>@1  7 " / | > >  ?>@1 7 " / | | / * / |

!"#$#%&'%9;!"#$#%&'%9)!"#$#%&'%98!"#$#%&'%9%!"#$#%&'%9'!"#$#%&'%99!"#$#%&'%9&!"#$#%&'%9:!"#$#%&'%&<!"#$#%&'%&(!"#$#%&'%&;!"#$#%&'%&)!"#$#%&'%&8!"#$#%&'%&%!"#$#%&'%&'!"#$#%&'%&9!"#$#%&'%&&!"#$#%&'%&:!"#$#%&'%:<!"#$#%&'%:(!"#$#%&'%:;!"#$#%&'%:)!"#$#%&'%:8!"#$#%&'%:% !"#$#%&'%:'!"#$#%&'%:9!"#$#%&'%:&!"#$#%&'%::!"#$#%&''<<!"#$#%&''<(!"#$#%&''<;!"#$#%&''<)!"#$#%&''<8!"#$#%&''<%!"#$#%&''<'!"#$#%&''<9!"#$#%&''<&!"#$#%&''<:!"#$#%&''(<!"#$#%&''((!"#$#%&''()!"#$#%&''(8!"#$#%&''(%!"#$#%&''('!"#$#%&''(&!"#$#%&''(:!"#$#%&'';<!"#$#%&'';;

!"#$#%&'';)!"#$#%&'';8!"#$#%&'';%!"#$#%&'';'!"#$#%&'';9!"#$#%&'';&!"#$#%&'';:!"#$#%&'')<!"#$#%&'')(!"#$#%&'');!"#$#%&''))!"#$#%&'')8!"#$#%&'')%!"#$#%&'')'!"#$#%&'')9!"#$#%&'')&!"#$#%&''):!"#$#%&''8<!"#$#%&''8(!"#$#%&''8;!"#$#%&''8)!"#$#%&''88!"#$#%&''8%!"#$#%&''8'

!"#$#%&''89!"#$#%&''8&!"#$#%&''8:!"#$#%&''%<!"#$#%&''%(!"#$#%&''%;!"#$#%&''%)!"#$#%&''%8!"#$#%&''%%!"#$#%&''%'!"#$#%&''%9!"#$#%&''%&!"#$#%&''%:!"#$#%&'''<!"#$#%&'''(!"#$#%&''';!"#$#%&''')!"#$#%&'''8!"#$#%&'''%!"#$#%&''''!"#$#%&'''9!"#$#%&'''&!"#$#%&''':!"#$#%&''9< !"#$#%&''9(!"#$#%&''9;!"#$#%&''9)!"#$#%&''98!"#$#%&''9%!"#$#%&''9'!"#$#%&''99!"#$#%&''9&!"#$#%&''9:!"#$#%&''&<!"#$#%&''&(!"#$#%&''&;!"#$#%&''&)!"#$#%&''&8!"#$#%&''&%!"#$#%&''&'!"#$#%&''&9!"#$#%&''&&!"#$#%&''&:!"#$#%&'':<!"#$#%&'':(!"#$#%&'':;!"#$#%&'':)!"#$#%&'':8 !"#$#%&'':%!"#$#%&'':'!"#$#%&'':9!"#$#%&'':&!"#$#%&''::!"#$#%&'9<<!"#$#%&'9<(!"#$#%&'9<;!"#$#%&'9<)!"#$#%&'9<8!"#$#%&'9<%!"#$#%&'9<'!"#$#%&'9<9!"#$#%&'9<&!"#$#%&'9(<!"#$#%&'9((!"#$#%&'9(;!"#$#%&'9()!"#$#%&'9(8!"#$#%&'9(%!"#$#%&'9('!"#$#%&'9(9!"#$#%&'9(&!"#$#%&'9(:

!"#$#%&'9;<!"#$#%&'9;(!"#$#%&'9;;!"#$#%&'9;)!"#$#%&'9;8!"#$#%&'9;%!"#$#%&'9;'!"#$#%&'9;9!"#$#%&'9;&!"#$#%&'9;:!"#$#%&'9)<!"#$#%&'9)(!"#$#%&'9);!"#$#%&'9))!"#$#%&'9)8!"#$#%&'9)%!"#$#%&'9)'!"#$#%&'9)9!"#$#%&'9)&!"#$#%&'9):!"#$#%&'98<!"#$#%&'98(!"#$#%&'98;!"#$#%&'98)

!"#$#%&'988!"#$#%&'98%!"#$#%&'98'!"#$#%&'989!"#$#%&'98&!"#$#%&'98:!"#$#%&'9%<!"#$#%&'9%(!"#$#%&'9%;!"#$#%&'9%)!"#$#%&'9%8!"#$#%&'9%%!"#$#%&'9%'!"#$#%&'9%9!"#$#%&'9%&!"#$#%&'9%:!"#$#%&'9'<!"#$#%&'9'(!"#$#%&'9';!"#$#%&'9')!"#$#%&'9'8!"#$#%&'9'%!"#$#%&'9''!"#$#%&'9'9

!"#$#%&'9'&!"#$#%&'9':!"#$#%&'99<!"#$#%&'99(!"#$#%&'99;!"#$#%&'99)!"#$#%&'998!"#$#%&'99%!"#$#%&'99'!"#$#%&'999!"#$#%&'99&!"#$#%&'99:!"#$#%&'9&<!"#$#%&'9&(!"#$#%&'9&;!"#$#%&'9&)!"#$#%&'9&8!"#$#%&'9&%!"#$#%&'9&'!"#$#%&'9&9!"#$#%&'9&&!"#$#%&'9&:!"#$#%&'9:<!"#$#%&'9:(

!"#$#%&'9:;!"#$#%&'9:)!"#$#%&'9:8!"#$#%&'9:%!"#$#%&'9:'!"#$#%&'9:9!"#$#%&'9:&!"#$#%&'9::!"#$#%&'&<<!"#$#%&'&<(!"#$#%&'&<;!"#$#%&'&<)!"#$#%&'&<8!"#$#%&'&<%!"#$#%&'&<'!"#$#%&'&<9!"#$#%&'&<&!"#$#%&'&<:!"#$#%&'&(<!"#$#%&'&((!"#$#%&'&(;!"#$#%&'&()!"#$#%&'&(8!"#$#%&'&(%

!"#$#%&'&('!"#$#%&'&(9!"#$#%&'&(&!"#$#%&'&(:!"#$#%&'&;<!"#$#%&'&;(!"#$#%&'&;;!"#$#%&'&;)!"#$#%&'&;8!"#$#%&'&;%!"#$#%&'&;'!"#$#%&'&;9!"#$#%&'&;&!"#$#%&'&;:!"#$#%&'&)<!"#$#%&'&)(!"#$#%&'&);!"#$#%&'&))!"#$#%&'&)8!"#$#%&'&)%!"#$#%&'&)'!"#$#%&'&)9!"#$#%&'&)&!"#$#%&'&):

!"#$#%&'&8<!"#$#%&'&8(!"#$#%&'&8;!"#$#%&'&8)!"#$#%&'&88!"#$#%&'&8%!"#$#%&'&8'!"#$#%&'&89!"#$#%&'&8&!"#$#%&'&8:!"#$#%&'&%<!"#$#%&'&%(!"#$#%&'&%;!"#$#%&'&%)!"#$#%&'&%8!"#$#%&'&%%!"#$#%&'&%'!"#$#%&'&%9!"#$#%&'&%&!"#$#%&'&%:!"#$#%&'&'<!"#$#%&'&'(!"#$#%&'&';!"#$#%&'&')

!"#$#%&'&'8!"#$#%&'&'%!"#$#%&'&''!"#$#%&'&'9!"#$#%&'&'&!"#$#%&'&':!"#$#%&'&9<!"#$#%&'&9(!"#$#%&'&9;!"#$#%&'&9)!"#$#%&'&98!"#$#%&'&9%!"#$#%&'&9'!"#$#%&'&99!"#$#%&'&9&!"#$#%&'&9:!"#$#%&'&&<!"#$#%&'&&(!"#$#%&'&&;!"#$#%&'&&)!"#$#%&'&&8!"#$#%&'&&%!"#$#%&'&&'!"#$#%&'&&9 !"#$#%&'&&&!"#$#%&'&&:!"#$#%&'&:<!"#$#%&'&:(!"#$#%&'&:;!"#$#%&'&:)!"#$#%&'&:8!"#$#%&'&:%!"#$#%&'&:'!"#$#%&'&:9!"#$#%&'&:&!"#$#%&'&::!"#$#%&':<<!"#$#%&':<(!"#$#%&':<;!"#$#%&':<)!"#$#%&':<8!"#$#%&':<%!"#$#%&':<'!"#$#%&':<&!"#$#%&':<:!"#$#%&':(<!"#$#%&':((!"#$#%&':(;

!"#$#%&':()!"#$#%&':(8!"#$#%&':(%!"#$#%&':('!"#$#%&':(9!"#$#%&':(&!"#$#%&':(:!"#$#%&':;<!"#$#%&':;(!"#$#%&':;;!"#$#%&':;)!"#$#%&':;8!"#$#%&':;%!"#$#%&':;'!"#$#%&':;9!"#$#%&':;&!"#$#%&':;:!"#$#%&':)<!"#$#%&':)(!"#$#%&':);!"#$#%&':))!"#$#%&':)8!"#$#%&':)%!"#$#%&':)'

!"#$#%&':)9!"#$#%&':)&!"#$#%&':):!"#$#%&':8<!"#$#%&':8(!"#$#%&':8;!"#$#%&':8)!"#$#%&':88!"#$#%&':8%!"#$#%&':8'!"#$#%&':89!"#$#%&':8&!"#$#%&':8:!"#$#%&':%<!"#$#%&':%(!"#$#%&':%;!"#$#%&':%)!"#$#%&':%8!"#$#%&':%%!"#$#%&':%'!"#$#%&':%9!"#$#%&':%&!"#$#%&':%:!"#$#%&':'<

!"#$#%&':'(!"#$#%&':';!"#$#%&':')!"#$#%&':'8!"#$#%&':'%!"#$#%&':''!"#$#%&':'9!"#$#%&':'&!"#$#%&':':!"#$#%&':9<!"#$#%&':9(!"#$#%&':9;!"#$#%&':9)!"#$#%&':98!"#$#%&':9%!"#$#%&':9'!"#$#%&':99!"#$#%&':9&!"#$#%&':9:!"#$#%&':&<!"#$#%&':&(!"#$#%&':&;!"#$#%&':&)!"#$#%&':&8

!"#$#%&':&%!"#$#%&':&'!"#$#%&':&9!"#$#%&':&&!"#$#%&':&:!"#$#%&'::<!"#$#%&'::(!"#$#%&'::;!"#$#%&'::)!"#$#%&'::8!"#$#%&'::%!"#$#%&'::'!"#$#%&'::9!"#$#%&'::&!"#$#%&':::!"#$#%&9<<<!"#$#%&9<<(!"#$#%&9<<;!"#$#%&9<<)!"#$#%&9<<8!"#$#%&9<<%!"#$#%&9<<'!"#$#%&9<<9!"#$#%&9<<&

!"#$#%&9<<:!"#$#%&9<(<!"#$#%&9<((!"#$#%&9<(;!"#$#%&9<()!"#$#%&9<(8!"#$#%&9<(%!"#$#%&9<('!"#$#%&9<(9!"#$#%&9<(&!"#$#%&9<(:!"#$#%&9<;<!"#$#%&9<;(!"#$#%&9<;;!"#$#%&9<;)!"#$#%&9<;8!"#$#%&9<;%!"#$#%&9<;'!"#$#%&9<;9!"#$#%&9<;&!"#$#%&9<;:!"#$#%&9<)<!"#$#%&9<)(!"#$#%&9<);

!"#$#%&9<))!"#$#%&9<)8!"#$#%&9<)%!"#$#%&9<)'!"#$#%&9<)9!"#$#%&9<)&!"#$#%&9<):!"#$#%&9<8<!"#$#%&9<8(!"#$#%&9<8;!"#$#%&9<8)!"#$#%&9<88!"#$#%&9<8%!"#$#%&9<8'!"#$#%&9<89!"#$#%&9<8&!"#$#%&9<8:!"#$#%&9<%<!"#$#%&9<%(!"#$#%&9<%;!"#$#%&9<%)!"#$#%&9<%8!"#$#%&9<%%!"#$#%&9<%'

!"#$#%&9<%9!"#$#%&9<%&!"#$#%&9<%:!"#$#%&9<'<!"#$#%&9<'(!"#$#%&9<';!"#$#%&9<')!"#$#%&9<'8!"#$#%&9<'%!"#$#%&9<''!"#$#%&9<'9!"#$#%&9<'&!"#$#%&9<':!"#$#%&9<9<!"#$#%&9<9(

$ $ " ->A(: +B ,7 0$ $ " 6 / !72 + +2 - $+ 52- ->A(: 0?CDD DA@

!"#$#%&9<9;!"#$#%&9<9)!"#$#%&9<98!"#$#%&9<9%!"#$#%&9<9'!"#$#%&9<99!"#$#%&9<9&!"#$#%&9<9:!"#$#%&9<&<!"#$#%&9<&(!"#$#%&9<&;!"#$#%&9<&)!"#$#%&9<&8!"#$#%&9<&'!"#$#%&9<&9!"#$#%&9<&&!"#$#%&9<&:!"#$#%&9<:<!"#$#%&9<:(!"#$#%&9<:;!"#$#%&9<:)!"#$#%&9<:8!"#$#%&9<:%!"#$#%&9<:' !"#$#%&9<:9!"#$#%&9<:&!"#$#%&9<::!"#$#%&9(<<!"#$#%&9(<(!"#$#%&9(<;!"#$#%&9(<)!"#$#%&9(<8!"#$#%&9(<%!"#$#%&9(<'!"#$#%&9(<9!"#$#%&9(<&!"#$#%&9(<:!"#$#%&9((<!"#$#%&9(((!"#$#%&9((;!"#$#%&9(()!"#$#%&9((8!"#$#%&9((%!"#$#%&9(('!"#$#%&9((9!"#$#%&9((&!"#$#%&9((:!"#$#%&9(;<

!"#$#%&9(;(!"#$#%&9(;;!"#$#%&9(;)!"#$#%&9(;8!"#$#%&9(;%!"#$#%&9(;'!"#$#%&9(;9!"#$#%&9(;&!"#$#%&9(;:!"#$#%&9()<!"#$#%&9()(!"#$#%&9();!"#$#%&9())!"#$#%&9()8!"#$#%&9()%!"#$#%&9()'!"#$#%&9()9!"#$#%&9()&!"#$#%&9():!"#$#%&9(8<!"#$#%&9(8(!"#$#%&9(8;!"#$#%&9(8)!"#$#%&9(88 !"#$#%&9(8%!"#$#%&9(8'!"#$#%&9(89!"#$#%&9(8&!"#$#%&9(8:!"#$#%&9(%<!"#$#%&9(%(!"#$#%&9(%;!"#$#%&9(%)!"#$#%&9(%8!"#$#%&9(%%!"#$#%&9(%'!"#$#%&9(%9!"#$#%&9(%&!"#$#%&9(%:!"#$#%&9('<!"#$#%&9('(!"#$#%&9(';!"#$#%&9(')!"#$#%&9('8!"#$#%&9('%!"#$#%&9(''!"#$#%&9('9!"#$#%&9('&

!"#$#%&9(':!"#$#%&9(9<!"#$#%&9(9(!"#$#%&9(9;!"#$#%&9(9)!"#$#%&9(98!"#$#%&9(9%!"#$#%&9(9'!"#$#%&9(99!"#$#%&9(9&!"#$#%&9(9:!"#$#%&9(&<!"#$#%&9(&(!"#$#%&9(&;!"#$#%&9(&)!"#$#%&9(&8!"#$#%&9(&%!"#$#%&9(&'!"#$#%&9(&9!"#$#%&9(&&!"#$#%&9(&:!"#$#%&9(:<!"#$#%&9(:(!"#$#%&9(:;

!"#$#%&9(:)!"#$#%&9(:8!"#$#%&9(:%!"#$#%&9(:'!"#$#%&9(:9!"#$#%&9(:&!"#$#%&9(::!"#$#%&9;<<!"#$#%&9;<(!"#$#%&9;<;!"#$#%&9;<)!"#$#%&9;<8!"#$#%&9;<%!"#$#%&9;<'!"#$#%&9;<9!"#$#%&9;<&!"#$#%&9;<:!"#$#%&9;(<!"#$#%&9;((!"#$#%&9;(;!"#$#%&9;()!"#$#%&9;(8!"#$#%&9;(%!"#$#%&9;('

!"#$#%&9;(9!"#$#%&9;(&!"#$#%&9;(:!"#$#%&9;;<!"#$#%&9;;(!"#$#%&9;;;!"#$#%&9;;)!"#$#%&9;;8!"#$#%&9;;%!"#$#%&9;;'!"#$#%&9;;9!"#$#%&9;;&!"#$#%&9;;:!"#$#%&9;)<!"#$#%&9;)(!"#$#%&9;);!"#$#%&9;))!"#$#%&9;)8!"#$#%&9;)%!"#$#%&9;)'!"#$#%&9;)9!"#$#%&9;)&!"#$#%&9;):!"#$#%&9;8<

!"#$#%&9;8(!"#$#%&9;8;!"#$#%&9;8)!"#$#%&9;88!"#$#%&9;8%!"#$#%&9;8'!"#$#%&9;89!"#$#%&9;8&!"#$#%&9;8:!"#$#%&9;%<!"#$#%&9;%(!"#$#%&9;%;!"#$#%&9;%)!"#$#%&9;%8!"#$#%&9;%%!"#$#%&9;%'!"#$#%&9;%9!"#$#%&9;%&!"#$#%&9;%:!"#$#%&9;'<!"#$#%&9;'(!"#$#%&9;';!"#$#%&9;')!"#$#%&9;'8 !"#$#%&9;''!"#$#%&9;'9!"#$#%&9;'&!"#$#%&9;':!"#$#%&9;9<!"#$#%&9;9(!"#$#%&9;9;!"#$#%&9;9)!"#$#%&9;98!"#$#%&9;9%!"#$#%&9;9'!"#$#%&9;99!"#$#%&9;9&!"#$#%&9;9:!"#$#%&9;&<!"#$#%&9;&(!"#$#%&9;&;!"#$#%&9;&)!"#$#%&9;&8!"#$#%&9;&%!"#$#%&9;&'!"#$#%&9;&9!"#$#%&9;&&!"#$#%&9;&:

!"#$#%&9;:<!"#$#%&9;:(!"#$#%&9;:;!"#$#%&9;:)!"#$#%&9;:8!"#$#%&9;:%!"#$#%&9;:'!"#$#%&9;:9!"#$#%&9;:&!"#$#%&9;::!"#$#%&9)<<!"#$#%&9)<(!"#$#%&9)<;

# $ $ " ->A(: +B ,7 0$ $ " 6 / !72 + +2 - $+

52- ->A(: 0

!"#$#%&9)<8!"#$#%&9)<%!"#$#%&9)<'!"#$#%&9)<9!"#$#%&9)<&!"#$#%&9)<:!"#$#%&9)(<!"#$#%&9)((!"#$#%&9)(;!"#$#%&9)()!"#$#%&9)(8!"#$#%&9)(%!"#$#%&9)('!"#$#%&9)(9!"#$#%&9)(&!"#$#%&9)(:!"#$#%&9);<!"#$#%&9);(!"#$#%&9);;!"#$#%&9);)!"#$#%&9);8!"#$#%&9);%!"#$#%&9);'!"#$#%&9);9 !"#$#%&9);&!"#$#%&9);:!"#$#%&9))<!"#$#%&9))(!"#$#%&9));!"#$#%&9)))!"#$#%&9))8!"#$#%&9))%!"#$#%&9))'!"#$#%&9))9!"#$#%&9))&!"#$#%&9)):!"#$#%&9)8<!"#$#%&9)8(!"#$#%&9)8;!"#$#%&9)8)!"#$#%&9)88!"#$#%&9)8%!"#$#%&9)8'!"#$#%&9)89!"#$#%&9)8&!"#$#%&9)8:!"#$#%&9)%<!"#$#%&9)%(

!"#$#%&9)%;!"#$#%&9)%)!"#$#%&9)%8!"#$#%&9)%%!"#$#%&9)%'!"#$#%&9)%9!"#$#%&9)%&!"#$#%&9)%:!"#$#%&9)'<!"#$#%&9)'(!"#$#%&9)';!"#$#%&9)')!"#$#%&9)'8!"#$#%&9)'%!"#$#%&9)''!"#$#%&9)'9!"#$#%&9)'&!"#$#%&9)':!"#$#%&9)9<!"#$#%&9)9(!"#$#%&9)9;!"#$#%&9)9)!"#$#%&9)98!"#$#%&9)9%

!"#$#%&9)9'!"#$#%&9)99!"#$#%&9)9&!"#$#%&9)9:!"#$#%&9)&(!"#$#%&9)&;!"#$#%&9)&)!"#$#%&9)&8!"#$#%&9)&%!"#$#%&9)&9!"#$#%&9)&&!"#$#%&9)&:!"#$#%&9):<!"#$#%&9):(!"#$#%&9):;!"#$#%&9):)!"#$#%&9):8!"#$#%&9):%!"#$#%&9):'!"#$#%&9):9!"#$#%&9):&!"#$#%&9)::!"#$#%&98<<!"#$#%&98<(

!"#$#%&98<;!"#$#%&98<)!"#$#%&98<8!"#$#%&98<%!"#$#%&98<'!"#$#%&98<9!"#$#%&98<&!"#$#%&98<:!"#$#%&98(<!"#$#%&98((!"#$#%&98(;!"#$#%&98()!"#$#%&98(8!"#$#%&98(%!"#$#%&98('!"#$#%&98(9!"#$#%&98(&!"#$#%&98(:!"#$#%&98;<!"#$#%&98;(!"#$#%&98;;!"#$#%&98;)!"#$#%&98;8!"#$#%&98;%

!"#$#%&98;'!"#$#%&98;9!"#$#%&98;&!"#$#%&98;:!"#$#%&98)<!"#$#%&98)(!"#$#%&98);!"#$#%&98))!"#$#%&98)8!"#$#%&98)%!"#$#%&98)'!"#$#%&98)9!"#$#%&98)&!"#$#%&98):!"#$#%&988<!"#$#%&988(!"#$#%&988;!"#$#%&988)!"#$#%&9888!"#$#%&988%!"#$#%&988'!"#$#%&9889!"#$#%&988&!"#$#%&988:

!"#$#%&98%<!"#$#%&98%(!"#$#%&98%;!"#$#%&98%)!"#$#%&98%8!"#$#%&98%%!"#$#%&98%'!"#$#%&98%9!"#$#%&98%&!"#$#%&98%:!"#$#%&98'<!"#$#%&98'(!"#$#%&98';!"#$#%&98')!"#$#%&98'8!"#$#%&98'%!"#$#%&98''!"#$#%&98'9!"#$#%&98'&!"#$#%&98':!"#$#%&989<!"#$#%&989(!"#$#%&989;!"#$#%&989)

!"#$#%&9898!"#$#%&989%!"#$#%&989'!"#$#%&989&!"#$#%&989:!"#$#%&98&<!"#$#%&98&(!"#$#%&98&)!"#$#%&98&8!"#$#%&98&%!"#$#%&98&'!"#$#%&98&9!"#$#%&98&&!"#$#%&98&:!"#$#%&98:<!"#$#%&98:(!"#$#%&98:;!"#$#%&98:)!"#$#%&98:8!"#$#%&98:%!"#$#%&98:'!"#$#%&98:9!"#$#%&98:&!"#$#%&98:: !"#$#%&9%<<!"#$#%&9%<(!"#$#%&9%<;!"#$#%&9%<)!"#$#%&9%<8!"#$#%&9%<%!"#$#%&9%<'!"#$#%&9%<9!"#$#%&9%<&!"#$#%&9%<:!"#$#%&9%(<!"#$#%&9%((!"#$#%&9%(;!"#$#%&9%()!"#$#%&9%(8!"#$#%&9%('!"#$#%&9%(9!"#$#%&9%(&!"#$#%&9%(:!"#$#%&9%;<!"#$#%&9%;(!"#$#%&9%;;!"#$#%&9%;)!"#$#%&9%;8 !"#$#%&9%;%!"#$#%&9%;'!"#$#%&9%;9!"#$#%&9%;&!"#$#%&9%;:!"#$#%&9%)<!"#$#%&9%)(!"#$#%&9%);!"#$#%&9%))!"#$#%&9%)8!"#$#%&9%)%!"#$#%&9%)'!"#$#%&9%)&!"#$#%&9%):!"#$#%&9%8<!"#$#%&9%8(!"#$#%&9%8;!"#$#%&9%8)!"#$#%&9%88!"#$#%&9%8%!"#$#%&9%8'!"#$#%&9%89!"#$#%&9%8&!"#$#%&9%8:

!"#$#%&9%%<!"#$#%&9%%(!"#$#%&9%%;!"#$#%&9%%)!"#$#%&9%%8!"#$#%&9%%%!"#$#%&9%%'!"#$#%&9%%9!"#$#%&9%%&!"#$#%&9%%:!"#$#%&9%'<!"#$#%&9%'(!"#$#%&9%';!"#$#%&9%')!"#$#%&9%'8!"#$#%&9%'%!"#$#%&9%''!"#$#%&9%'9!"#$#%&9%'&!"#$#%&9%':!"#$#%&9%9<!"#$#%&9%9(!"#$#%&9%9;!"#$#%&9%9)

!"#$#%&9%98!"#$#%&9%9%!"#$#%&9%9'!"#$#%&9%99!"#$#%&9%9&!"#$#%&9%9:!"#$#%&9%&<!"#$#%&9%&(!"#$#%&9%&;!"#$#%&9%&)!"#$#%&9%&8!"#$#%&9%&%!"#$#%&9%&'!"#$#%&9%&9!"#$#%&9%&&!"#$#%&9%&:!"#$#%&9%:(!"#$#%&9%:;!"#$#%&9%:)!"#$#%&9%:8!"#$#%&9%:%!"#$#%&9%:'!"#$#%&9%:9!"#$#%&9%:& !"#$#%&9%::!"#$#%&9'<<!"#$#%&9'<(!"#$#%&9'<;!"#$#%&9'<)!"#$#%&9'<8!"#$#%&9'<%!"#$#%&9'<'!"#$#%&9'<9!"#$#%&9'<&!"#$#%&9'<:!"#$#%&9'(<!"#$#%&9'((!"#$#%&9'(;!"#$#%&9'()!"#$#%&9'(8!"#$#%&9'(%!"#$#%&9'('!"#$#%&9'(9!"#$#%&9'(&!"#$#%&9'(:!"#$#%&9';<!"#$#%&9';(!"#$#%&9';;

!"#$#%&9';)!"#$#%&9';8!"#$#%&9';%!"#$#%&9';'!"#$#%&9';9!"#$#%&9';&!"#$#%&9';:!"#$#%&9')<!"#$#%&9')(!"#$#%&9');!"#$#%&9'))!"#$#%&9')8!"#$#%&9')%!"#$#%&9')'!"#$#%&9')9!"#$#%&9')&!"#$#%&9'):!"#$#%&9'8<!"#$#%&9'8(!"#$#%&9'8;!"#$#%&9'8)!"#$#%&9'88!"#$#%&9'8%!"#$#%&9'8'

!"#$#%&9'89!"#$#%&9'8&!"#$#%&9'8:!"#$#%&9'%<!"#$#%&9'%(!"#$#%&9'%;!"#$#%&9'%)!"#$#%&9'%8!"#$#%&9'%%!"#$#%&9'%'!"#$#%&9'%9!"#$#%&9'%&!"#$#%&9'%:!"#$#%&9''<!"#$#%&9''(!"#$#%&9'';!"#$#%&9'')!"#$#%&9''8!"#$#%&9''%!"#$#%&9'''!"#$#%&9''9!"#$#%&9''&!"#$#%&9'':!"#$#%&9'9<

!"#$#%&9'9(!"#$#%&9'9;!"#$#%&9'9)!"#$#%&9'98!"#$#%&9'9%!"#$#%&9'9'!"#$#%&9'99!"#$#%&9'9&!"#$#%&9'9:!"#$#%&9'&<!"#$#%&9'&(!"#$#%&9'&;!"#$#%&9'&)!"#$#%&9'&8!"#$#%&9'&%!"#$#%&9'&'!"#$#%&9'&9!"#$#%&9'&&!"#$#%&9'&:!"#$#%&9':<!"#$#%&9':(!"#$#%&9':;!"#$#%&9':)!"#$#%&9':8

!"#$#%&9':%!"#$#%&9':'!"#$#%&9':9!"#$#%&9':&!"#$#%&9'::!"#$#%&99<<!"#$#%&99<(!"#$#%&99<;!"#$#%&99<)!"#$#%&99<8!"#$#%&99<%!"#$#%&99<'!"#$#%&99<9!"#$#%&99<&!"#$#%&99<:!"#$#%&99(<

# $ $ ->A(: 7> = 2- - 4 6 / !72 + +2 - $

52- + ->A(: 0

!"#$#%&99((!"#$#%&99(;!"#$#%&99()!"#$#%&99(8!"#$#%&99(%!"#$#%&99('!"#$#%&99(9!"#$#%&99(&!"#$#%&99(:!"#$#%&99;<!"#$#%&99;(!"#$#%&99;;!"#$#%&99;)!"#$#%&99;8!"#$#%&99;%!"#$#%&99;'!"#$#%&99;9!"#$#%&99;&!"#$#%&99;:!"#$#%&99)<!"#$#%&99)(!"#$#%&99);!"#$#%&99))!"#$#%&99)8 !"#$#%&99)%!"#$#%&99)'!"#$#%&99)9!"#$#%&99)&!"#$#%&99):!"#$#%&998<!"#$#%&998(!"#$#%&998;!"#$#%&998)!"#$#%&9988!"#$#%&998%!"#$#%&998'!"#$#%&9989!"#$#%&998&!"#$#%&998:!"#$#%&99%<!"#$#%&99%(!"#$#%&99%;!"#$#%&99%)!"#$#%&99%8!"#$#%&99%%!"#$#%&99%'!"#$#%&99%9!"#$#%&99%& !"#$#%&99%:!"#$#%&99'<!"#$#%&99'(!"#$#%&99';!"#$#%&99')!"#$#%&99'8!"#$#%&99'%!"#$#%&99''!"#$#%&99'9!"#$#%&99'&!"#$#%&99':!"#$#%&999<!"#$#%&999(!"#$#%&999;!"#$#%&999)!"#$#%&9998!"#$#%&999%!"#$#%&999'!"#$#%&9999!"#$#%&999&!"#$#%&999:!"#$#%&99&<!"#$#%&99&(!"#$#%&99&; !"#$#%&99&)!"#$#%&99&8!"#$#%&99&%!"#$#%&99&'!"#$#%&99&9!"#$#%&99&&!"#$#%&99&:!"#$#%&99:<!"#$#%&99:(!"#$#%&99:;!"#$#%&99:)!"#$#%&99:8!"#$#%&99:%!"#$#%&99:'!"#$#%&99:&!"#$#%&99::!"#$#%&9&<<!"#$#%&9&<(!"#$#%&9&<;!"#$#%&9&<)!"#$#%&9&<8!"#$#%&9&<%!"#$#%&9&<'!"#$#%&9&<9

!"#$#%&9&<&!"#$#%&9&<:!"#$#%&9&(<!"#$#%&9&((!"#$#%&9&(;!"#$#%&9&()!"#$#%&9&(8!"#$#%&9&(%!"#$#%&9&('!"#$#%&9&(9!"#$#%&9&(&!"#$#%&9&(:!"#$#%&9&;<!"#$#%&9&;(!"#$#%&9&;;!"#$#%&9&;)!"#$#%&9&;8!"#$#%&9&;%!"#$#%&9&;'!"#$#%&9&;9!"#$#%&9&;&!"#$#%&9&;:!"#$#%&9&)<!"#$#%&9&)(

!"#$#%&9&);!"#$#%&9&))!"#$#%&9&)8!"#$#%&9&)%!"#$#%&9&)'!"#$#%&9&)9!"#$#%&9&)&!"#$#%&9&):!"#$#%&9&8<!"#$#%&9&8(!"#$#%&9&8;!"#$#%&9&8)!"#$#%&9&88!"#$#%&9&8%!"#$#%&9&8'!"#$#%&9&89!"#$#%&9&8&!"#$#%&9&8:!"#$#%&9&%<!"#$#%&9&%(!"#$#%&9&%;!"#$#%&9&%)!"#$#%&9&%8!"#$#%&9&%% !"#$#%&9&%'!"#$#%&9&%9!"#$#%&9&%:!"#$#%&9&'<!"#$#%&9&'(!"#$#%&9&';!"#$#%&9&')!"#$#%&9&'8!"#$#%&9&'%!"#$#%&9&''!"#$#%&9&'9!"#$#%&9&'&!"#$#%&9&':!"#$#%&9&9<!"#$#%&9&9(!"#$#%&9&9;!"#$#%&9&9)!"#$#%&9&98!"#$#%&9&9%!"#$#%&9&9'!"#$#%&9&99!"#$#%&9&9&!"#$#%&9&9:!"#$#%&9&&< !"#$#%&9&&(!"#$#%&9&&;!"#$#%&9&&)!"#$#%&9&&8!"#$#%&9&&%!"#$#%&9&&'!"#$#%&9&&9!"#$#%&9&&&!"#$#%&9&:<!"#$#%&9&:(!"#$#%&9&:;!"#$#%&9&:)!"#$#%&9&:8!"#$#%&9&:%!"#$#%&9&:'!"#$#%&9&:9!"#$#%&9&:&!"#$#%&9&::!"#$#%&9:<<!"#$#%&9:<(!"#$#%&9:<;!"#$#%&9:<)!"#$#%&9:<8!"#$#%&9:<%

!"#$#%&9:<'!"#$#%&9:<9!"#$#%&9:<&!"#$#%&9:<:!"#$#%&9:(<!"#$#%&9:((!"#$#%&9:(;!"#$#%&9:()!"#$#%&9:(8!"#$#%&9:(%!"#$#%&9:('!"#$#%&9:(9!"#$#%&9:(&!"#$#%&9:(:!"#$#%&9:;<!"#$#%&9:;(!"#$#%&9:;;!"#$#%&9:;)!"#$#%&9:;8!"#$#%&9:;%!"#$#%&9:;'!"#$#%&9:;9!"#$#%&9:;&!"#$#%&9:;:

!"#$#%&9:)<!"#$#%&9:)(!"#$#%&9:);!"#$#%&9:))!"#$#%&9:)8!"#$#%&9:)%!"#$#%&9:)'!"#$#%&9:)9!"#$#%&9:)&!"#$#%&9:):!"#$#%&9:8<!"#$#%&9:8(!"#$#%&9:8;!"#$#%&9:8)!"#$#%&9:88!"#$#%&9:8%!"#$#%&9:8'!"#$#%&9:89!"#$#%&9:8&!"#$#%&9:8:!"#$#%&9:%<!"#$#%&9:%(!"#$#%&9:%;!"#$#%&9:%)

!"#$#%&9:%8!"#$#%&9:%%!"#$#%&9:%'!"#$#%&9:%9!"#$#%&9:%&!"#$#%&9:%:!"#$#%&9:'(!"#$#%&9:';!"#$#%&9:')!"#$#%&9:'8!"#$#%&9:'%!"#$#%&9:''!"#$#%&9:'9!"#$#%&9:'&!"#$#%&9:':!"#$#%&9:9<!"#$#%&9:9(!"#$#%&9:9;!"#$#%&9:9)!"#$#%&9:98!"#$#%&9:9%!"#$#%&9:9'!"#$#%&9:9&!"#$#%&9:9:

!"#$#%&9:&<!"#$#%&9:&(!"#$#%&9:&;!"#$#%&9:&)!"#$#%&9:&8!"#$#%&9:&%!"#$#%&9:&'!"#$#%&9:&9!"#$#%&9:&&!"#$#%&9:&:!"#$#%&9::<!"#$#%&9::(!"#$#%&9::;!"#$#%&9::)!"#$#%&9::8!"#$#%&9::%!"#$#%&9::'!"#$#%&9::9!"#$#%&9::&!"#$#%&&<<<!"#$#%&&<<(!"#$#%&&<<;!"#$#%&&<<)!"#$#%&&<<8

!"#$#%&&<<%!"#$#%&&<<'!"#$#%&&<<&!"#$#%&&<<:!"#$#%&&<(<!"#$#%&&<((!"#$#%&&<(;!"#$#%&&<()!"#$#%&&<(8!"#$#%&&<(%!"#$#%&&<('!"#$#%&&<(9!"#$#%&&<(&!"#$#%&&<(:!"#$#%&&<;<!"#$#%&&<;(!"#$#%&&<;;!"#$#%&&<;)!"#$#%&&<;8!"#$#%&&<;%!"#$#%&&<;9!"#$#%&&<;&!"#$#%&&<;:!"#$#%&&<)<

!"#$#%&&<)(!"#$#%&&<);!"#$#%&&<))!"#$#%&&<)8!"#$#%&&<)%!"#$#%&&<)'!"#$#%&&<)9!"#$#%&&<)&!"#$#%&&<):!"#$#%&&<8<!"#$#%&&<8(!"#$#%&&<8;!"#$#%&&<8)!"#$#%&&<88!"#$#%&&<8%!"#$#%&&<8'!"#$#%&&<89!"#$#%&&<8&!"#$#%&&<8:!"#$#%&&<%(!"#$#%&&<%;!"#$#%&&<%)!"#$#%&&<%8!"#$#%&&<%% !"#$#%&&<%'!"#$#%&&<%9!"#$#%&&<%&!"#$#%&&<%:

# $ $ " ->A(: +B ,7 0$ $ " 6 / !72 + +2 - $+

52- ->A(: 0

!"#$#%&&<'<!"#$#%&&<'(!"#$#%&&<';!"#$#%&&<')!"#$#%&&<'8!"#$#%&&<'%!"#$#%&&<''!"#$#%&&<'9!"#$#%&&<'&!"#$#%&&<':!"#$#%&&<9<!"#$#%&&<9(!"#$#%&&<9;!"#$#%&&<9)!"#$#%&&<98!"#$#%&&<9'!"#$#%&&<99!"#$#%&&<9&!"#$#%&&<9:!"#$#%&&<&<!"#$#%&&<&(!"#$#%&&<&;!"#$#%&&<&)!"#$#%&&<&8

!"#$#%&&<&%!"#$#%&&<&'!"#$#%&&<&9!"#$#%&&<&&!"#$#%&&<&:!"#$#%&&<:<!"#$#%&&<:(!"#$#%&&<:;!"#$#%&&<:)!"#$#%&&<:8!"#$#%&&<:%!"#$#%&&<:'!"#$#%&&<:9!"#$#%&&<:&!"#$#%&&<::!"#$#%&&(<<!"#$#%&&(<(!"#$#%&&(<;!"#$#%&&(<)!"#$#%&&(<8!"#$#%&&(<%!"#$#%&&(<'!"#$#%&&(<9!"#$#%&&(<&

!"#$#%&&(<:!"#$#%&&((<!"#$#%&&(((!"#$#%&&((;!"#$#%&&(()!"#$#%&&((8!"#$#%&&((%!"#$#%&&(('!"#$#%&&((9!"#$#%&&((&!"#$#%&&((:!"#$#%&&(;<!"#$#%&&(;(!"#$#%&&(;;!"#$#%&&(;)!"#$#%&&(;8!"#$#%&&(;'!"#$#%&&(;9!"#$#%&&(;:!"#$#%&&()<!"#$#%&&()(!"#$#%&&();!"#$#%&&())!"#$#%&&()8

!"#$#%&&()%!"#$#%&&()'!"#$#%&&()9!"#$#%&&()&!"#$#%&&():!"#$#%&&(8<!"#$#%&&(8(!"#$#%&&(8;!"#$#%&&(8)!"#$#%&&(88!"#$#%&&(8%!"#$#%&&(8'!"#$#%&&(89!"#$#%&&(8&!"#$#%&&(8:!"#$#%&&(%<!"#$#%&&(%(!"#$#%&&(%;!"#$#%&&(%)!"#$#%&&(%8!"#$#%&&(%%!"#$#%&&(%'!"#$#%&&(%9!"#$#%&&(%&

!"#$#%&&(%:!"#$#%&&('<!"#$#%&&('(!"#$#%&&(';!"#$#%&&(')!"#$#%&&('8!"#$#%&&('%!"#$#%&&(''!"#$#%&&('9!"#$#%&&('&!"#$#%&&(':!"#$#%&&(9<!"#$#%&&(9(!"#$#%&&(9;!"#$#%&&(9)!"#$#%&&(98!"#$#%&&(9'!"#$#%&&(99!"#$#%&&(9&!"#$#%&&(9:!"#$#%&&(&<!"#$#%&&(&(!"#$#%&&(&;!"#$#%&&(&)

!"#$#%&&(&8!"#$#%&&(&%!"#$#%&&(&'!"#$#%&&(&9!"#$#%&&(&&!"#$#%&&(&:!"#$#%&&(:<!"#$#%&&(:(!"#$#%&&(:;!"#$#%&&(:)!"#$#%&&(:8!"#$#%&&(:%!"#$#%&&(:'!"#$#%&&(:9!"#$#%&&(:&!"#$#%&&(::!"#$#%&&;<<!"#$#%&&;<(!"#$#%&&;<;!"#$#%&&;<)!"#$#%&&;<8!"#$#%&&;<%!"#$#%&&;<'!"#$#%&&;<9

!"#$#%&&;<&!"#$#%&&;<:!"#$#%&&;(<!"#$#%&&;(;!"#$#%&&;()!"#$#%&&;(8!"#$#%&&;(%!"#$#%&&;('!"#$#%&&;(9!"#$#%&&;(&!"#$#%&&;(:!"#$#%&&;;<!"#$#%&&;;(!"#$#%&&;;;!"#$#%&&;;)!"#$#%&&;;8!"#$#%&&;;%!"#$#%&&;;'!"#$#%&&;;9!"#$#%&&;;:!"#$#%&&;)<!"#$#%&&;)(!"#$#%&&;);!"#$#%&&;))

!"#$#%&&;)8!"#$#%&&;)%!"#$#%&&;)'!"#$#%&&;)9!"#$#%&&;)&!"#$#%&&;):!"#$#%&&;8<!"#$#%&&;8(!"#$#%&&;8;!"#$#%&&;8)!"#$#%&&;88!"#$#%&&;8%!"#$#%&&;89!"#$#%&&;8&!"#$#%&&;8:!"#$#%&&;%<!"#$#%&&;%(!"#$#%&&;%;!"#$#%&&;%)!"#$#%&&;%8!"#$#%&&;%%!"#$#%&&;%'!"#$#%&&;%9!"#$#%&&;%&

!"#$#%&&;%:!"#$#%&&;'<!"#$#%&&;'(!"#$#%&&;';!"#$#%&&;')!"#$#%&&;'8!"#$#%&&;'%!"#$#%&&;''!"#$#%&&;'9!"#$#%&&;'&!"#$#%&&;':!"#$#%&&;9<!"#$#%&&;9(!"#$#%&&;9;!"#$#%&&;9)!"#$#%&&;98!"#$#%&&;9%!"#$#%&&;9'!"#$#%&&;99!"#$#%&&;9&!"#$#%&&;9:!"#$#%&&;&<!"#$#%&&;&(!"#$#%&&;&; !"#$#%&&;&)!"#$#%&&;&8!"#$#%&&;&'!"#$#%&&;&9!"#$#%&&;&&!"#$#%&&;&:!"#$#%&&;:<!"#$#%&&;:(!"#$#%&&;:;!"#$#%&&;:)!"#$#%&&;:8!"#$#%&&;:%!"#$#%&&;:'!"#$#%&&;:9!"#$#%&&;::!"#$#%&&)<<!"#$#%&&)<(!"#$#%&&)<;!"#$#%&&)<)!"#$#%&&)<8!"#$#%&&)<'!"#$#%&&)<9!"#$#%&&)<&!"#$#%&&)<: !"#$#%&&)(<!"#$#%&&)((!"#$#%&&)(;!"#$#%&&)()!"#$#%&&)(8!"#$#%&&)(%!"#$#%&&)('!"#$#%&&)(9!"#$#%&&)(&!"#$#%&&)(:!"#$#%&&);<!"#$#%&&);(!"#$#%&&);;!"#$#%&&);)!"#$#%&&);8!"#$#%&&);%!"#$#%&&);'!"#$#%&&);9!"#$#%&&);&!"#$#%&&);:!"#$#%&&))<!"#$#%&&))(!"#$#%&&));!"#$#%&&)))

!"#$#%&&))8!"#$#%&&))%!"#$#%&&))'!"#$#%&&))9!"#$#%&&))&!"#$#%&&)):!"#$#%&&)8<!"#$#%&&)8(!"#$#%&&)8;!"#$#%&&)8)!"#$#%&&)88!"#$#%&&)8%!"#$#%&&)8'!"#$#%&&)89!"#$#%&&)8&!"#$#%&&)%<!"#$#%&&)%(!"#$#%&&)%;!"#$#%&&)%)!"#$#%&&)%8!"#$#%&&)%%!"#$#%&&)%'!"#$#%&&)%9!"#$#%&&)%&

!"#$#%&&)%:!"#$#%&&)'<!"#$#%&&)'(!"#$#%&&)';!"#$#%&&)')!"#$#%&&)'8!"#$#%&&)'%!"#$#%&&)''!"#$#%&&)'9!"#$#%&&)'&!"#$#%&&)':!"#$#%&&)9<!"#$#%&&)9(!"#$#%&&)9;!"#$#%&&)9)!"#$#%&&)98!"#$#%&&)9%!"#$#%&&)9'!"#$#%&&)99!"#$#%&&)9&!"#$#%&&)9:!"#$#%&&)&<!"#$#%&&)&(!"#$#%&&)&;

!"#$#%&&)&)!"#$#%&&)&8!"#$#%&&)&%!"#$#%&&)&'!"#$#%&&)&9!"#$#%&&)&&!"#$#%&&)&:!"#$#%&&):<!"#$#%&&):(!"#$#%&&):;!"#$#%&&):)!"#$#%&&):8!"#$#%&&):%!"#$#%&&):'!"#$#%&&):9!"#$#%&&):&!"#$#%&&)::!"#$#%&&8<<!"#$#%&&8<;!"#$#%&&8<)!"#$#%&&8<8!"#$#%&&8<%!"#$#%&&8<'!"#$#%&&8<9

!"#$#%&&8<&!"#$#%&&8<:!"#$#%&&8(<!"#$#%&&8((!"#$#%&&8(;!"#$#%&&8()!"#$#%&&8(8!"#$#%&&8(%!"#$#%&&8('!"#$#%&&8(9!"#$#%&&8(&!"#$#%&&8(:!"#$#%&&8;<!"#$#%&&8;(!"#$#%&&8;;!"#$#%&&8;)!"#$#%&&8;8!"#$#%&&8;%!"#$#%&&8;'!"#$#%&&8;9!"#$#%&&8;&!"#$#%&&8;:!"#$#%&&8)<!"#$#%&&8)(

!"#$#%&&8);!"#$#%&&8))!"#$#%&&8)8!"#$#%&&8)%!"#$#%&&8)'!"#$#%&&8)9!"#$#%&&8):!"#$#%&&88<!"#$#%&&88(!"#$#%&&88;!"#$#%&&88)!"#$#%&&888!"#$#%&&88%!"#$#%&&88'!"#$#%&&889!"#$#%&&88&!"#$#%&&88:!"#$#%&&8%<!"#$#%&&8%(!"#$#%&&8%;!"#$#%&&8%)!"#$#%&&8%8!"#$#%&&8%'!"#$#%&&8%9

!"#$#%&&8%&!"#$#%&&8%:!"#$#%&&8'<!"#$#%&&8'(!"#$#%&&8';!"#$#%&&8')!"#$#%&&8'8!"#$#%&&8'%!"#$#%&&8''!"#$#%&&8'9!"#$#%&&8'&!"#$#%&&8':!"#$#%&&89<!"#$#%&&89(!"#$#%&&89;!"#$#%&&89)!"#$#%&&898!"#$#%&&89%!"#$#%&&89'!"#$#%&&899!"#$#%&&89&!"#$#%&&89:!"#$#%&&8&<!"#$#%&&8&( !"#$#%&&8&;!"#$#%&&8&)!"#$#%&&8&8!"#$#%&&8&%!"#$#%&&8&'!"#$#%&&8&9!"#$#%&&8&&!"#$#%&&8&:!"#$#%&&8:<!"#$#%&&8:(!"#$#%&&8:;!"#$#%&&8:)!"#$#%&&8:8!"#$#%&&8:%!"#$#%&&8:9!"#$#%&&8:&!"#$#%&&8::!"#$#%&&%<<!"#$#%&&%<(!"#$#%&&%<;!"#$#%&&%<)!"#$#%&&%<8!"#$#%&&%<%!"#$#%&&%<' !"#$#%&&%<9!"#$#%&&%<&!"#$#%&&%<:!"#$#%&&%(<!"#$#%&&%((!"#$#%&&%(;!"#$#%&&%()!"#$#%&&%(8!"#$#%&&%(%!"#$#%&&%('!"#$#%&&%(9!"#$#%&&%(&!"#$#%&&%(:!"#$#%&&%;<!"#$#%&&%;(!"#$#%&&%;;!"#$#%&&%;)!"#$#%&&%;8!"#$#%&&%;%!"#$#%&&%;'!"#$#%&&%;9!"#$#%&&%;&!"#$#%&&%;:!"#$#%&&%)< !"#$#%&&%)(!"#$#%&&%);!"#$#%&&%))!"#$#%&&%)8!"#$#%&&%)%!"#$#%&&%)'!"#$#%&&%)9!"#$#%&&%)&!"#$#%&&%):!"#$#%&&%8<!"#$#%&&%8;!"#$#%&&%8)!"#$#%&&%88!"#$#%&&%8%!"#$#%&&%8'!"#$#%&&%89!"#$#%&&%8&!"#$#%&&%8:!"#$#%&&%%<!"#$#%&&%%(!"#$#%&&%%;!"#$#%&&%%)!"#$#%&&%%%!"#$#%&&%%'

!"#$#%&&%%9!"#$#%&&%%&!"#$#%&&%%:!"#$#%&&%'<!"#$#%&&%'(!"#$#%&&%';!"#$#%&&%')!"#$#%&&%'8!"#$#%&&%'%!"#$#%&&%''!"#$#%&&%'9!"#$#%&&%'&!"#$#%&&%':!"#$#%&&%9<!"#$#%&&%9(!"#$#%&&%9)!"#$#%&&%98!"#$#%&&%9%!"#$#%&&%9'!"#$#%&&%99!"#$#%&&%9&!"#$#%&&%9:!"#$#%&&%&<!"#$#%&&%&(

!"#$#%&&%&;!"#$#%&&%&)!"#$#%&&%&8!"#$#%&&%&%!"#$#%&&%&'!"#$#%&&%&9!"#$#%&&%&&!"#$#%&&%&:!"#$#%&&%:<!"#$#%&&%:(!"#$#%&&%:;!"#$#%&&%:)!"#$#%&&%:8!"#$#%&&%:%!"#$#%&&%:'!"#$#%&&%:9!"#$#%&&%:&!"#$#%&&%::!"#$#%&&'<<!"#$#%&&'<(!"#$#%&&'<;!"#$#%&&'<)!"#$#%&&'<8!"#$#%&&'<%

!"#$#%&&'<'!"#$#%&&'<9!"#$#%&&'<&!"#$#%&&'<:!"#$#%&&'(<!"#$#%&&'((!"#$#%&&'(;!"#$#%&&'()!"#$#%&&'(8!"#$#%&&'(%!"#$#%&&'('!"#$#%&&'(9!"#$#%&&'(&!"#$#%&&'(:!"#$#%&&';<!"#$#%&&';(!"#$#%&&';;!"#$#%&&';)!"#$#%&&';8!"#$#%&&';%!"#$#%&&';'!"#$#%&&';9!"#$#%&&';&!"#$#%&&';:

!"#$#%&&')<!"#$#%&&')(!"#$#%&&');!"#$#%&&'))!"#$#%&&')8!"#$#%&&')%!"#$#%&&')'!"#$#%&&')9!"#$#%&&')&!"#$#%&&'):!"#$#%&&'8<!"#$#%&&'8(!"#$#%&&'8;!"#$#%&&'8)!"#$#%&&'88!"#$#%&&'8%!"#$#%&&'8'!"#$#%&&'89!"#$#%&&'8&!"#$#%&&'8:!"#$#%&&'%<!"#$#%&&'%(!"#$#%&&'%;!"#$#%&&'%)

!"#$#%&&'%8!"#$#%&&'%%!"#$#%&&'%'!"#$#%&&'%9!"#$#%&&'%&!"#$#%&&'%:!"#$#%&&''<!"#$#%&&''(!"#$#%&&'';!"#$#%&&'')!"#$#%&&''8!"#$#%&&''%!"#$#%&&'''!"#$#%&&''9!"#$#%&&''&!"#$#%&&'':!"#$#%&&'9<!"#$#%&&'9(!"#$#%&&'9;!"#$#%&&'9)!"#$#%&&'9%!"#$#%&&'9'!"#$#%&&'99!"#$#%&&'9& !"#$#%&&'9:!"#$#%&&'&<!"#$#%&&'&(!"#$#%&&'&;!"#$#%&&'&)!"#$#%&&'&8!"#$#%&&'&%!"#$#%&&'&'!"#$#%&&'&9!"#$#%&&'&&!"#$#%&&'&:!"#$#%&&':<!"#$#%&&':(!"#$#%&&':;!"#$#%&&':)!"#$#%&&':8!"#$#%&&':'!"#$#%&&':9!"#$#%&&':&!"#$#%&&'::!"#$#%&&9<<!"#$#%&&9<(!"#$#%&&9<;!"#$#%&&9<) !"#$#%&&9<8!"#$#%&&9<%!"#$#%&&9<'!"#$#%&&9<9!"#$#%&&9<&!"#$#%&&9<:!"#$#%&&9(<!"#$#%&&9((!"#$#%&&9(;!"#$#%&&9()!"#$#%&&9(8!"#$#%&&9('!"#$#%&&9(&!"#$#%&&9(:!"#$#%&&9;<!"#$#%&&9;(!"#$#%&&9;;!"#$#%&&9;)!"#$#%&&9;8!"#$#%&&9;%!"#$#%&&9;'!"#$#%&&9;9!"#$#%&&9;&!"#$#%&&9;:

!"#$#%&&9)<!"#$#%&&9)(!"#$#%&&9);!"#$#%&&9))!"#$#%&&9)8!"#$#%&&9)%!"#$#%&&9)'!"#$#%&&9)9!"#$#%&&9)&!"#$#%&&9):!"#$#%&&98<!"#$#%&&98(!"#$#%&&98;!"#$#%&&98)!"#$#%&&988!"#$#%&&98%!"#$#%&&98'!"#$#%&&989!"#$#%&&98&!"#$#%&&98:!"#$#%&&9%<!"#$#%&&9%(!"#$#%&&9%;!"#$#%&&9%)

!"#$#%&&9%8!"#$#%&&9%%!"#$#%&&9%'!"#$#%&&9%9!"#$#%&&9%&!"#$#%&&9%:!"#$#%&&9'<!"#$#%&&9'(!"#$#%&&9';!"#$#%&&9')!"#$#%&&9'8!"#$#%&&9'%!"#$#%&&9''!"#$#%&&9'9!"#$#%&&9'&!"#$#%&&9':!"#$#%&&99<!"#$#%&&99(!"#$#%&&99;!"#$#%&&99)!"#$#%&&998!"#$#%&&99%!"#$#%&&99'!"#$#%&&999

!"#$#%&&99&!"#$#%&&99:!"#$#%&&9&<!"#$#%&&9&(!"#$#%&&9&;!"#$#%&&9&)!"#$#%&&9&8!"#$#%&&9&%!"#$#%&&9&'!"#$#%&&9&9!"#$#%&&9&&!"#$#%&&9&:!"#$#%&&9:<!"#$#%&&9:(!"#$#%&&9:)!"#$#%&&9:8!"#$#%&&9:%!"#$#%&&9:'!"#$#%&&9:9!"#$#%&&9:&!"#$#%&&9::!"#$#%&&&<<!"#$#%&&&<(!"#$#%&&&<;

!"#$#%&&&<)!"#$#%&&&<8!"#$#%&&&<%!"#$#%&&&<'!"#$#%&&&<9!"#$#%&&&<&!"#$#%&&&<:!"#$#%&&&(<!"#$#%&&&((!"#$#%&&&(;!"#$#%&&&()!"#$#%&&&(8!"#$#%&&&(%!"#$#%&&&('!"#$#%&&&(9!"#$#%&&&(&!"#$#%&&&(:!"#$#%&&&;<!"#$#%&&&;(!"#$#%&&&;;!"#$#%&&&;)!"#$#%&&&;8!"#$#%&&&;%!"#$#%&&&;'

!"#$#%&&&;9!"#$#%&&&;&!"#$#%&&&;:!"#$#%&&&)<!"#$#%&&&)(!"#$#%&&&);!"#$#%&&&))!"#$#%&&&)8!"#$#%&&&)%!"#$#%&&&)'!"#$#%&&&)9!"#$#%&&&)&!"#$#%&&&):!"#$#%&&&8<!"#$#%&&&8(!"#$#%&&&8;!"#$#%&&&8)!"#$#%&&&8'!"#$#%&&&89!"#$#%&&&8&!"#$#%&&&8:!"#$#%&&&%<!"#$#%&&&%(!"#$#%&&&%;

!"#$#%&&&%)!"#$#%&&&%8!"#$#%&&&%%!"#$#%&&&%'!"#$#%&&&%9!"#$#%&&&%&!"#$#%&&&%:!"#$#%&&&'<!"#$#%&&&'(!"#$#%&&&';!"#$#%&&&')!"#$#%&&&'8!"#$#%&&&'%!"#$#%&&&''!"#$#%&&&'&!"#$#%&&&':!"#$#%&&&9<!"#$#%&&&9(!"#$#%&&&9;!"#$#%&&&9)!"#$#%&&&98!"#$#%&&&9%!"#$#%&&&9'!"#$#%&&&99

!"#$#%&&&9&!"#$#%&&&9:!"#$#%&&&&<!"#$#%&&&&(!"#$#%&&&&)!"#$#%&&&&8!"#$#%&&&&%!"#$#%&&&&'!"#$#%&&&&9!"#$#%&&&&&!"#$#%&&&&:!"#$#%&&&:<!"#$#%&&&:(!"#$#%&&&:;!"#$#%&&&:)!"#$#%&&&:8!"#$#%&&&:%!"#$#%&&&:'!"#$#%&&&:9!"#$#%&&&:&!"#$#%&&&::!"#$#%&&:<<!"#$#%&&:<(!"#$#%&&:<;

!"#$#%&&:<)!"#$#%&&:<8!"#$#%&&:<%!"#$#%&&:<'!"#$#%&&:<9!"#$#%&&:<&!"#$#%&&:<:!"#$#%&&:(<!"#$#%&&:((!"#$#%&&:(;!"#$#%&&:()!"#$#%&&:(8!"#$#%&&:(%!"#$#%&&:('!"#$#%&&:(9!"#$#%&&:(&!"#$#%&&:(:!"#$#%&&:;<!"#$#%&&:;(!"#$#%&&:;;!"#$#%&&:;)!"#$#%&&:;8!"#$#%&&:;%!"#$#%&&:;'

!"#$#%&&:;&!"#$#%&&:;:!"#$#%&&:)<!"#$#%&&:)(!"#$#%&&:);!"#$#%&&:))!"#$#%&&:)8!"#$#%&&:)%!"#$#%&&:)'!"#$#%&&:)9!"#$#%&&:)&!"#$#%&&:):!"#$#%&&:8(!"#$#%&&:8;!"#$#%&&:8)!"#$#%&&:88!"#$#%&&:8%!"#$#%&&:8'!"#$#%&&:89!"#$#%&&:8&!"#$#%&&:8:!"#$#%&&:%<!"#$#%&&:%(!"#$#%&&:%;

!"#$#%&&:%)!"#$#%&&:%8!"#$#%&&:%%!"#$#%&&:%'!"#$#%&&:%9!"#$#%&&:%&!"#$#%&&:%:!"#$#%&&:'<!"#$#%&&:'(!"#$#%&&:')!"#$#%&&:'8!"#$#%&&:'%!"#$#%&&:''!"#$#%&&:'9!"#$#%&&:'&!"#$#%&&:':!"#$#%&&:9<!"#$#%&&:9(!"#$#%&&:9;!"#$#%&&:9)!"#$#%&&:98!"#$#%&&:9%!"#$#%&&:9'!"#$#%&&:99

!"#$#%&&:9&!"#$#%&&:9:!"#$#%&&:&<!"#$#%&&:&(!"#$#%&&:&;!"#$#%&&:&)!"#$#%&&:&8!"#$#%&&:&%!"#$#%&&:&'!"#$#%&&:&9!"#$#%&&:&&!"#$#%&&:&:!"#$#%&&::<!"#$#%&&::(!"#$#%&&::;!"#$#%&&::)!"#$#%&&::8!"#$#%&&::%!"#$#%&&::'!"#$#%&&::9!"#$#%&&::&!"#$#%&&:::!"#$#%&:<<<!"#$#%&:<<( !"#$#%&:<<;!"#$#%&:<<)!"#$#%&:<<8!"#$#%&:<<%!"#$#%&:<<'!"#$#%&:<<9!"#$#%&:<<&!"#$#%&:<<:!"#$#%&:<(<!"#$#%&:<((!"#$#%&:<()!"#$#%&:<(8!"#$#%&:<('

|  | $ $ | ->A(: 52- | 7> = 2- - 4 6 / !72 + +2 - $ + ->A(: 0 |
| --- | --- | --- | --- |
| !"#$#%&:<(9!"#$#%&:<(&!"#$#%&:<(: | $ $, 2 | ->A(: 52- | 0$ $, 2 6 / !72 + +2 - $+  ->A(: 0 |
| !"#$#%&:<;< | $ $ | ->A(: 52- | 7> = 2- - 4 6 / !72 + +2 - $ + ->A(: 0 |
| !"#$#%&:<;( | $ $, 2 | ->A(: 52- | 0$ $, 2 6 / !72 + +2 - $+  ->A(: 0 |
| !"#$#%&:<;;!"#$#%&:<;) | $ $ | ->A(: 52- | 7> = 2- - 4 6 / !72 + +2 - $ + ->A(: 0 |
| !"#$#%&:<;8 | $ $, 2 | ->A(: 52- | 0$ $, 2 6 / !72 + +2 - $+  ->A(: 0 |
| !"#$#%&:<;% | $ $ | ->A(: 52- | 7> = 2- - 4 6 / !72 + +2 - $ + ->A(: 0 |
| !"#$#%&:<;' | $ $, 2 | ->A(: 52- | 0$ $, 2 6 / !72 + +2 - $+  ->A(: 0 |
| !"#$#%&:<;9 | $ $ | ->A(: 52- | 7> = 2- - 4 6 / !72 + +2 - $ + ->A(: 0 |
| !"#$#%&:<;&!"#$#%&:<;:!"#$#%&:<)< | $ $, 2 | ->A(: 52- | 0$ $, 2 6 / !72 + +2 - $+  ->A(: 0 |
| !"#$#%&:<)(!"#$#%&:<);!"#$#%&:<)) | $ $ | ->A(: 52- | 7> = 2- - 4 6 / !72 + +2 - $ + ->A(: 0 |
| !"#$#%&:<)8 | $ $, 2 | ->A(: 52- | 0$ $, 2 6 / !72 + +2 - $+  ->A(: 0 |
| !"#$#%&:<)%!"#$#%&:<)'!"#$#%&:<)& | $ $ | ->A(: 52- | 7> = 2- - 4 6 / !72 + +2 - $ + ->A(: 0 |
| !"#$#%&:<):!"#$#%&:<8<!"#$#%&:<8( | $ $, 2 | ->A(: 52- | 0$ $, 2 6 / !72 + +2 - $+  ->A(: 0 |
| !"#$#%&:<8; | $ $ | ->A(: 52- | 7> = 2- - 4 6 / !72 + +2 - $ + ->A(: 0 |
| !"#$#%&:<8) | $ $, 2 | ->A(: 52- | 0$ $, 2 6 / !72 + +2 - $+  ->A(: 0 |
| !"#$#%&:<88!"#$#%&:<8%!"#$#%&:<8' !"#$#%&:<89!"#$#%&:<8&!"#$#%&:<8: | $ $ | ->A(: 52- | 7> = 2- - 4 6 / !72 + +2 - $ + ->A(: 0 |
| !"#$#%&:<%( | $ $, 2 | ->A(: 52- | 0$ $, 2 6 / !72 + +2 - $+  ->A(: 0 |
| !"#$#%&:<%;!"#$#%&:<%)!"#$#%&:<%8 !"#$#%&:<%%!"#$#%&:<%' | $ $ | ->A(: 52- | 7> = 2- - 4 6 / !72 + +2 - $ + ->A(: 0 |
| !"#$#%&:<%9 | $ $, 2 | ->A(: 52- | 0$ $, 2 6 / !72 + +2 - $+  ->A(: 0 |
| !"#$#%&:<%&!"#$#%&:<%:!"#$#%&:<'<  !"#$#%&:<'( | $ $ | ->A(: 52- | 7> = 2- - 4 6 / !72 + +2 - $ + ->A(: 0 |
| !"#$#%&:<') | $ $, 2 | ->A(: 52- | 0$ $, 2 6 / !72 + +2 - $+  ->A(: 0 |
| !"#$#%&:<'8!"#$#%&:<'%!"#$#%&:<'' | $ $ | ->A(: 52- | 7> = 2- - 4 6 / !72 + +2 - $ + ->A(: 0 |
| !"#$#%&:<'9 | $ $, 2 | ->A(: 52- | 0$ $, 2 6 / !72 + +2 - $+  ->A(: 0 |

| !"#$#%&:<'&!"#$#%&:<': | $ $ |  | 7> = 2- - 4 6 / !72 + +2 - $ + ->A(: 0 |
| --- | --- | --- | --- |
| !"#$#%&:<9< | $ $, 2 | ->A(: 52- | 0$ $, 2 6 / !72 + +2 - $+  ->A(: 0 |
| !"#$#%&:<9(!"#$#%&:<9;!"#$#%&:<9) | $ $ | ->A(: 52- | 7> = 2- - 4 6 / !72 + +2 - $ + ->A(: 0 |
| !"#$#%&:<98!"#$#%&:<9% | $ $, 2 | ->A(: 52- | 0$ $, 2 6 / !72 + +2 - $+  ->A(: 0 |
| !"#$#%&:<9' | $ $ | ->A(: 52- | 7> = 2- - 4 6 / !72 + +2 - $ + ->A(: 0 |
| !"#$#%&:<99 | $ $, 2 | ->A(: 52- | 0$ $, 2 6 / !72 + +2 - $+  ->A(: 0 |
| !"#$#%&:<9& | $ $ | ->A(: 52- | 7> = 2- - 4 6 / !72 + +2 - $ + ->A(: 0 |
| !"#$#%&:<9:!"#$#%&:<&<!"#$#%&:<&( | $ $, 2 | ->A(: 52- | 0$ $, 2 6 / !72 + +2 - $+  ->A(: 0 |
| !"#$#%&:<&; | $ $ | ->A(: 52- | 7> = 2- - 4 6 / !72 + +2 - $ + ->A(: 0 |
| !"#$#%&:<&) | $ $, 2 | ->A(: 52- | 0$ $, 2 6 / !72 + +2 - $+  ->A(: 0 |
| !"#$#%&:<&8!"#$#%&:<&% | $ $ | ->A(: 52- | 7> = 2- - 4 6 / !72 + +2 - $ + ->A(: 0 |
| !"#$#%&:<&' | $ $, 2 | ->A(: 52- | 0$ $, 2 6 / !72 + +2 - $+  ->A(: 0 |
| !"#$#%&:<&9!"#$#%&:<&&!"#$#%&:<&: | $ $ | ->A(: 52- | 7> = 2- - 4 6 / !72 + +2 - $ + ->A(: 0 |
| !"#$#%&:<:<!"#$#%&:<:( | $ $, 2 | ->A(: 52- | 0$ $, 2 6 / !72 + +2 - $+  ->A(: 0 |
| !"#$#%&:<:;!"#$#%&:<:)!"#$#%&:<:8 | $ $ | ->A(: 52- | 7> = 2- - 4 6 / !72 + +2 - $ + ->A(: 0 |
| !"#$#%&:<:% | $ $, 2 | ->A(: 52- | 0$ $, 2 6 / !72 + +2 - $+  ->A(: 0 |
| !"#$#%&:<:' | $ $ | ->A(: 52- | 7> = 2- - 4 6 / !72 + +2 - $ + ->A(: 0 |
| !"#$#%&:<:9!"#$#%&:<:&!"#$#%&:<:: | $ $, 2 | ->A(: 52- | 0$ $, 2 6 / !72 + +2 - $+  ->A(: 0 |
| !"#$#%&:(<< | $ $ | ->A(: 52- | 7> = 2- - 4 6 / !72 + +2 - $ + ->A(: 0 |
| !"#$#%&:(<( | $ $, 2 | ->A(: 52- | 0$ $, 2 6 / !72 + +2 - $+  ->A(: 0 |
| !"#$#%&:(<; | $ $ | ->A(: 52- | 7> = 2- - 4 6 / !72 + +2 - $ + ->A(: 0 |
| !"#$#%&:(<)!"#$#%&:(<8 | $ $, 2 | ->A(: 52- | 0$ $, 2 6 / !72 + +2 - $+  ->A(: 0 |
| !"#$#%&:(<%!"#$#%&:(<'!"#$#%&:(<9 | $ $ | ->A(: 52- | 7> = 2- - 4 6 / !72 + +2 - $ + ->A(: 0 |
| !"#$#%&:(<&!"#$#%&:(<:!"#$#%&:((<  !"#$#%&:((( | $ $, 2 | ->A(: 52- | 0$ $, 2 6 / !72 + +2 - $+  ->A(: 0 |
| !"#$#%&:((;!"#$#%&:(() | $ $ | ->A(: 52- | 7> = 2- - 4 6 / !72 + +2 - $ + ->A(: 0 |
| !"#$#%&:((8!"#$#%&:((% | $ $, 2 | ->A(: 52- | 0$ $, 2 6 / !72 + +2 - $+  ->A(: 0 |
| !"#$#%&:((' | $ $ | ->A(: 52- | 7> = 2- - 4 6 / !72 + +2 - $ + ->A(: 0 |
| !"#$#%&:((9!"#$#%&:((& | $ $, 2 | ->A(: 52- | 0$ $, 2 6 / !72 + +2 - $+  ->A(: 0 |
| !"#$#%&:((:!"#$#%&:(;< | $ $ | ->A(: 52- | 7> = 2- - 4 6 / !72 + +2 - $ + ->A(: 0 |
| !"#$#%&:(;(!"#$#%&:(;;!"#$#%&:(;) | $ $, 2 | ->A(: 52- | 0$ $, 2 6 / !72 + +2 - $+  ->A(: 0 |
| !"#$#%&:(;8!"#$#%&:(;%!"#$#%&:(;' | $ $ | ->A(: 52- | 7> = 2- - 4 6 / !72 + +2 - $ + ->A(: 0 |
| !"#$#%&:(;9 | $ $, 2 | ->A(: 52- | 0$ $, 2 6 / !72 + +2 - $+  ->A(: 0 |
| !"#$#%&:(;&!"#$#%&:(;:!"#$#%&:()(  !"#$#%&:(); | $ $ | ->A(: 52- | 7> = 2- - 4 6 / !72 + +2 - $ + ->A(: 0 |
| !"#$#%&:()) | $ $, 2 | ->A(: 52- | 0$ $, 2 6 / !72 + +2 - $+  ->A(: 0 |
| !"#$#%&:()8!"#$#%&:()' | $ $ | ->A(: 52- | 7> = 2- - 4 6 / !72 + +2 - $ + ->A(: 0 |
| !"#$#%&:()9 | $ $, 2 | ->A(: 52- | 0$ $, 2 6 / !72 + +2 - $+  ->A(: 0 |
| !"#$#%&:()&!"#$#%&:():!"#$#%&:(8<  !"#$#%&:(8(!"#$#%&:(8;!"#$#%&:(8)  !"#$#%&:(88!"#$#%&:(8%!"#$#%&:(8' | $ $ | ->A(: 52- | 7> = 2- - 4 6 / !72 + +2 - $ + ->A(: 0 |
| !"#$#%&:(89 | $ $, 2 | ->A(: 52- | 0$ $, 2 6 / !72 + +2 - $+  ->A(: 0 |
| !"#$#%&:(8&!"#$#%&:(%<!"#$#%&:(%(  !"#$#%&:(%; | $ $ | ->A(: 52- | 7> = 2- - 4 6 / !72 + +2 - $ + ->A(: 0 |
| !"#$#%&:(%) | $ $, 2 | ->A(: 52- | 0$ $, 2 6 / !72 + +2 - $+  ->A(: 0 |
| !"#$#%&:(%8!"#$#%&:(%%!"#$#%&:(%' | $ $ | ->A(: 52- | 7> = 2- - 4 6 / !72 + +2 - $ + ->A(: 0 |
| !"#$#%&:(%9 | $ $, 2 | ->A(: 52- | 0$ $, 2 6 / !72 + +2 - $+  ->A(: 0 |
| !"#$#%&:(%&!"#$#%&:(%: | $ $ | ->A(: 52- | 7> = 2- - 4 6 / !72 + +2 - $ + ->A(: 0 |
| !"#$#%&:('<!"#$#%&:('( | $ $, 2 | ->A(: 52- | 0$ $, 2 6 / !72 + +2 - $+  ->A(: 0 |
| !"#$#%&:(';!"#$#%&:(')!"#$#%&:('8 | $ $ | ->A(: | 7> = 2- - 4 6 / !72 + +2 - $ |

!"#$#%&:;') $ $ ->A(:

# 52-

| !"#$#%&:('% |  | 52- | + ->A(: 0 |
| --- | --- | --- | --- |
| !"#$#%&:(''!"#$#%&:('9!"#$#%&:('&  !"#$#%&:(':!"#$#%&:(9<!"#$#%&:(9(  !"#$#%&:(9;!"#$#%&:(9)!"#$#%&:(98 | $ $, 2 | ->A(: 52- | 0$ $, 2 6 / !72 + +2 - $+  ->A(: 0 |
| !"#$#%&:(9% | $ $ | ->A(: 52- | 7> = 2- - 4 6 / !72 + +2 - $ + ->A(: 0 |
| !"#$#%&:(9' | $ $, 2 | ->A(: 52- | 0$ $, 2 6 / !72 + +2 - $+  ->A(: 0 |
| !"#$#%&:(99!"#$#%&:(9& | $ $ | ->A(: 52- | 7> = 2- - 4 6 / !72 + +2 - $ + ->A(: 0 |
| !"#$#%&:(9: | $ $, 2 | ->A(: 52- | 0$ $, 2 6 / !72 + +2 - $+  ->A(: 0 |
| !"#$#%&:(&<!"#$#%&:(&( | $ $ | ->A(: 52- | 7> = 2- - 4 6 / !72 + +2 - $ + ->A(: 0 |
| !"#$#%&:(&;!"#$#%&:(&)!"#$#%&:(&% | $ $, 2 | ->A(: 52- | 0$ $, 2 6 / !72 + +2 - $+  ->A(: 0 |
| !"#$#%&:(&' | $ $ | ->A(: 52- | 7> = 2- - 4 6 / !72 + +2 - $ + ->A(: 0 |
| !"#$#%&:(&9 | $ $, 2 | ->A(: 52- | 0$ $, 2 6 / !72 + +2 - $+  ->A(: 0 |
| !"#$#%&:(&&!"#$#%&:(&:!"#$#%&:(:< !"#$#%&:(:(!"#$#%&:(:;!"#$#%&:(:8 | $ $ | ->A(: 52- | 7> = 2- - 4 6 / !72 + +2 - $ + ->A(: 0 |
| !"#$#%&:(:%!"#$#%&:(:' | $ $, 2 | ->A(: 52- | 0$ $, 2 6 / !72 + +2 - $+  ->A(: 0 |
| !"#$#%&:(:9 | $ $ | ->A(: 52- | 7> = 2- - 4 6 / !72 + +2 - $ + ->A(: 0 |
| !"#$#%&:(:&!"#$#%&:(::!"#$#%&:;<< | $ $, 2 | ->A(: 52- | 0$ $, 2 6 / !72 + +2 - $+  ->A(: 0 |
| !"#$#%&:;<(!"#$#%&:;<;!"#$#%&:;<)  !"#$#%&:;<8 | $ $ | ->A(: 52- | 7> = 2- - 4 6 / !72 + +2 - $ + ->A(: 0 |
| !"#$#%&:;<% | $ $, 2 | ->A(: 52- | 0$ $, 2 6 / !72 + +2 - $+  ->A(: 0 |
| !"#$#%&:;<'!"#$#%&:;<9!"#$#%&:;<& !"#$#%&:;<:!"#$#%&:;(< | $ $ | ->A(: 52- | 7> = 2- - 4 6 / !72 + +2 - $ + ->A(: 0 |
| !"#$#%&:;((!"#$#%&:;(; | $ $, 2 | ->A(: 52- | 0$ $, 2 6 / !72 + +2 - $+  ->A(: 0 |
| !"#$#%&:;()!"#$#%&:;(%!"#$#%&:;(' !"#$#%&:;(9!"#$#%&:;(&!"#$#%&:;(: !"#$#%&:;;< | $ $ | ->A(: 52- | 7> = 2- - 4 6 / !72 + +2 - $ + ->A(: 0 |
| !"#$#%&:;;( | $ $, 2 | ->A(: 52- | 0$ $, 2 6 / !72 + +2 - $+  ->A(: 0 |
| !"#$#%&:;;;!"#$#%&:;;) | $ $ | ->A(: 52- | 7> = 2- - 4 6 / !72 + +2 - $ + ->A(: 0 |
| !"#$#%&:;;8!"#$#%&:;;%!"#$#%&:;;'  !"#$#%&:;;9 | $ $, 2 | ->A(: 52- | 0$ $, 2 6 / !72 + +2 - $+  ->A(: 0 |
| !"#$#%&:;;& | $ $ | ->A(: 52- | 7> = 2- - 4 6 / !72 + +2 - $ + ->A(: 0 |
| !"#$#%&:;;: | $ $, 2 | ->A(: 52- | 0$ $, 2 6 / !72 + +2 - $+  ->A(: 0 |
| !"#$#%&:;)< | $ $ | ->A(: 52- | 7> = 2- - 4 6 / !72 + +2 - $ + ->A(: 0 |
| !"#$#%&:;)( | $ $, 2 | ->A(: 52- | 0$ $, 2 6 / !72 + +2 - $+  ->A(: 0 |
| !"#$#%&:;); | $ $ | ->A(: 52- | 7> = 2- - 4 6 / !72 + +2 - $ + ->A(: 0 |
| !"#$#%&:;)) | $ $, 2 | ->A(: 52- | 0$ $, 2 6 / !72 + +2 - $+  ->A(: 0 |
| !"#$#%&:;)8 | $ $ | ->A(: 52- | 7> = 2- - 4 6 / !72 + +2 - $ + ->A(: 0 |
| !"#$#%&:;)% | $ $, 2 | ->A(: 52- | 0$ $, 2 6 / !72 + +2 - $+  ->A(: 0 |
| !"#$#%&:;)' | $ $ | ->A(: 52- | 7> = 2- - 4 6 / !72 + +2 - $ + ->A(: 0 |
| !"#$#%&:;)9 | $ $, 2 | ->A(: 52- | 0$ $, 2 6 / !72 + +2 - $+  ->A(: 0 |
| !"#$#%&:;)&!"#$#%&:;):!"#$#%&:;8<  !"#$#%&:;8( | $ $ | ->A(: 52- | 7> = 2- - 4 6 / !72 + +2 - $ + ->A(: 0 |
| !"#$#%&:;8;!"#$#%&:;8) | $ $, 2 | ->A(: 52- | 0$ $, 2 6 / !72 + +2 - $+  ->A(: 0 |
| !"#$#%&:;88!"#$#%&:;8%!"#$#%&:;8' | $ $ | ->A(: 52- | 7> = 2- - 4 6 / !72 + +2 - $ + ->A(: 0 |
| !"#$#%&:;89 | $ $, 2 | ->A(: 52- | 0$ $, 2 6 / !72 + +2 - $+  ->A(: 0 |
| !"#$#%&:;8:!"#$#%&:;%< | $ $ | ->A(: 52- | 7> = 2- - 4 6 / !72 + +2 - $ + ->A(: 0 |
| !"#$#%&:;%( | $ $, 2 | ->A(: 52- | 0$ $, 2 6 / !72 + +2 - $+  ->A(: 0 |
| !"#$#%&:;%; | $ $ | ->A(: 52- | 7> = 2- - 4 6 / !72 + +2 - $ + ->A(: 0 |
| !"#$#%&:;%) | $ $, 2 | ->A(: 52- | 0$ $, 2 6 / !72 + +2 - $+  ->A(: 0 |
| !"#$#%&:;%8!"#$#%&:;%% | $ $ | ->A(: 52- | 7> = 2- - 4 6 / !72 + +2 - $ + ->A(: 0 |
| !"#$#%&:;%' | $ $, 2 | ->A(: 52- | 0$ $, 2 6 / !72 + +2 - $+  ->A(: 0 |
| !"#$#%&:;%9!"#$#%&:;%& | $ $ | ->A(: 52- | 7> = 2- - 4 6 / !72 + +2 - $ + ->A(: 0 |
| !"#$#%&:;%:!"#$#%&:;'<!"#$#%&:;'( | $ $, 2 | ->A(: 52- | 0$ $, 2 6 / !72 + +2 - $+  ->A(: 0 |

7> = 2- - 4 6 / !72 + +2 - $

+ ->A(: 0

!"#$#%&:;'%!"#$#%&:;'' $ $, 2 0$ $, 2 6 / !72 + +2 - $+

->A(: 0

!"#$#%&:;'&!"#$#%&:;':!"#$#%&:;9<!"#$#%&:;9(!"#$#%&:;9;!"#$#%&:;9)!"#$#%&:;98!"#$#%&:;9%!"#$#%&:;9'!"#$#%&:;99!"#$#%&:;9&!"#$#%&:;9:!"#$#%&:;&<!"#$#%&:;&(!"#$#%&:;&;!"#$#%&:;&)!"#$#%&:;&8!"#$#%&:;&%!"#$#%&:;&'!"#$#%&:;&9!"#$#%&:;&&!"#$#%&:;:<!"#$#%&:;:(!"#$#%&:;:;

!"#$#%&:;:)!"#$#%&:;:8!"#$#%&:;:%!"#$#%&:;:'

|  | $ $ " | ->A(: 52- | +B ,7 0$ $ " 6 / !72 + +2 - $+ ->A(: 0 |
| --- | --- | --- | --- |
| !"#$#%&:;:9 | $ $, 2 | ->A(: 52- | 0$ $, 2 6 / !72 + +2 - $+  ->A(: 0 |
| !"#$#%&:;:&!"#$#%&:;::!"#$#%&:)<<  !"#$#%&:)<( | $ $ " | ->A(: 52- | +B ,7 0$ $ " 6 / !72 + +2 - $+ ->A(: 0 |
| !"#$#%&:)<8 | $ $, 2 | ->A(: 52- | 0$ $, 2 6 / !72 + +2 - $+  ->A(: 0 |

!"#$#%&:)<%!"#$#%&:)<'!"#$#%&:)<9!"#$#%&:)<&!"#$#%&:)<:!"#$#%&:)(<!"#$#%&:)((!"#$#%&:)(;!"#$#%&:)()!"#$#%&:)(8!"#$#%&:)(%!"#$#%&:)('!"#$#%&:)(9!"#$#%&:)(&!"#$#%&:)(:!"#$#%&:);<!"#$#%&:);(!"#$#%&:);;!"#$#%&:);)!"#$#%&:);8!"#$#%&:);%!"#$#%&:);'!"#$#%&:);9!"#$#%&:);&

!"#$#%&:))<!"#$#%&:))(!"#$#%&:));!"#$#%&:)))

|  | $ $ " | ->A(: 52- | +B ,7 0$ $ " 6 / !72 + +2 - $+ ->A(: 0 |
| --- | --- | --- | --- |
| !"#$#%&:))% | $ $, 2 | ->A(: | 0$ $, 2 6 / !72 + +2 - $+ |

52- ->A(: 0

!"#$#%&:))'!"#$#%&:))9!"#$#%&:))&!"#$#%&:)):!"#$#%&:)8<!"#$#%&:)8(!"#$#%&:)8)!"#$#%&:)8%!"#$#%&:)8'!"#$#%&:)89!"#$#%&:)8&!"#$#%&:)8:!"#$#%&:)%<!"#$#%&:)%;!"#$#%&:)%)!"#$#%&:)%8

|  | $ $ " | ->A(: 52- | +B ,7 0$ $ " 6 / !72 + +2 - $+ ->A(: 0 |
| --- | --- | --- | --- |
| !"#$#%&:)%% | $ $, 2 | ->A(: | 0$ $, 2 6 / !72 + +2 - $+ |

52- ->A(: 0

!"#$#%&:)%'!"#$#%&:)%9!"#$#%&:)%&!"#$#%&:)%:!"#$#%&:)'<!"#$#%&:)'(!"#$#%&:)';!"#$#%&:)')!"#$#%&:)'8!"#$#%&:)'%!"#$#%&:)''!"#$#%&:)'9!"#$#%&:)'&!"#$#%&:)':!"#$#%&:)9<!"#$#%&:)9(!"#$#%&:)9;!"#$#%&:)9)!"#$#%&:)98!"#$#%&:)9%

|  | $ $ " | ->A(: 52- | +B ,7 0$ $ " 6 / !72 + +2 - $+ ->A(: 0 |
| --- | --- | --- | --- |
| !"#$#%&:)99 | $ $, 2 | ->A(: | 0$ $, 2 6 / !72 + +2 - $+ |

52- ->A(: 0

!"#$#%&:)9&!"#$#%&:)9:!"#$#%&:)&<!"#$#%&:)&(!"#$#%&:)&;!"#$#%&:)&)!"#$#%&:)&8!"#$#%&:)&%!"#$#%&:)&'!"#$#%&:)&9!"#$#%&:)&&!"#$#%&:)&:!"#$#%&:):(!"#$#%&:):;!"#$#%&:):)!"#$#%&:):8!"#$#%&:):%!"#$#%&:):'!"#$#%&:):9!"#$#%&:):&!"#$#%&:)::!"#$#%&:8<<!"#$#%&:8<(!"#$#%&:8<;

!"#$#%&:8<)!"#$#%&:8<8!"#$#%&:8<%!"#$#%&:8<'

|  | $ $ " | ->A(: 52- | +B ,7 0$ $ " 6 / !72 + +2 - $+ ->A(: 0 |
| --- | --- | --- | --- |
| !"#$#%&:8<9 | $ $, 2 | ->A(: 52- | 0$ $, 2 6 / !72 + +2 - $+  ->A(: 0 |
| !"#$#%&:8<&!"#$#%&:8<:!"#$#%&:8(<  !"#$#%&:8((!"#$#%&:8(;!"#$#%&:8()  !"#$#%&:8(8!"#$#%&:8(%!"#$#%&:8(' | $ $ " | ->A(: 52- | +B ,7 0$ $ " 6 / !72 + +2 - $+ ->A(: 0 |
| !"#$#%&:8(9 | $ $, 2 | ->A(: 52- | 0$ $, 2 6 / !72 + +2 - $+  ->A(: 0 |
| !"#$#%&:8(&!"#$#%&:8(:!"#$#%&:8;< !"#$#%&:8;(!"#$#%&:8;; | $ $ " | ->A(: 52- | +B ,7 0$ $ " 6 / !72 + +2 - $+ ->A(: 0 |
| !"#$#%&:8;) | $ $, 2 | ->A(: 52- | 0$ $, 2 6 / !72 + +2 - $+  ->A(: 0 |
| !"#$#%&:8;8!"#$#%&:8;%!"#$#%&:8;' !"#$#%&:8;9!"#$#%&:8;&!"#$#%&:8;: | $ $ " | ->A(: 52- | +B ,7 0$ $ " 6 / !72 + +2 - $+ ->A(: 0 |
| !"#$#%&:8)< | $ $, 2 | ->A(: 52- | 0$ $, 2 6 / !72 + +2 - $+  ->A(: 0 |
| !"#$#%&:8)(!"#$#%&:8);!"#$#%&:8))  !"#$#%&:8)8!"#$#%&:8)%!"#$#%&:8)' !"#$#%&:8)9!"#$#%&:8)&!"#$#%&:8): | $ $ " | ->A(: 52- | +B ,7 0$ $ " 6 / !72 + +2 - $+ ->A(: 0 |
| !"#$#%&:88< | $ $, 2 | ->A(: 52- | 0$ $, 2 6 / !72 + +2 - $+  ->A(: 0 |

!"#$#%&:88(!"#$#%&:88;!"#$#%&:88)!"#$#%&:888!"#$#%&:88%!"#$#%&:88'!"#$#%&:889!"#$#%&:88&!"#$#%&:88:!"#$#%&:8%<!"#$#%&:8%(!"#$#%&:8%;!"#$#%&:8%)!"#$#%&:8%8!"#$#%&:8%%!"#$#%&:8%'!"#$#%&:8%9!"#$#%&:8%&!"#$#%&:8%:!"#$#%&:8'<!"#$#%&:8'(!"#$#%&:8';!"#$#%&:8')!"#$#%&:8'8 !"#$#%&:8'%!"#$#%&:8''!"#$#%&:8'9!"#$#%&:8'&!"#$#%&:8':!"#$#%&:89<!"#$#%&:89(!"#$#%&:89;!"#$#%&:89)!"#$#%&:898!"#$#%&:89%!"#$#%&:89'!"#$#%&:899!"#$#%&:89&!"#$#%&:89:!"#$#%&:8&<!"#$#%&:8&(!"#$#%&:8&;!"#$#%&:8&)!"#$#%&:8&8!"#$#%&:8&%!"#$#%&:8&'!"#$#%&:8&9!"#$#%&:8&&

!"#$#%&:8&:!"#$#%&:8:<!"#$#%&:8:(!"#$#%&:8:;

|  | $ $ " | ->A(: 52- | +B ,7 0$ $ " 6 / !72 + +2 - $+ ->A(: 0 |
| --- | --- | --- | --- |
| !"#$#%&:8:) | $ $, 2 | ->A(: | 0$ $, 2 6 / !72 + +2 - $+ |

52- ->A(: 0

!"#$#%&:8:8!"#$#%&:8:%!"#$#%&:8:'!"#$#%&:8:9!"#$#%&:8:&!"#$#%&:8::!"#$#%&:%<<!"#$#%&:%<(!"#$#%&:%<;!"#$#%&:%<)!"#$#%&:%<8!"#$#%&:%<%!"#$#%&:%<'!"#$#%&:%<9!"#$#%&:%<&!"#$#%&:%<:!"#$#%&:%(<!"#$#%&:%((!"#$#%&:%(;!"#$#%&:%()!"#$#%&:%(8!"#$#%&:%(%!"#$#%&:%('!"#$#%&:%(9

!"#$#%&:%(&

|  | $ $ " | ->A(: 52- | +B ,7 0$ $ " 6 / !72 + +2 - $+ ->A(: 0 |
| --- | --- | --- | --- |
| !"#$#%&:%(:!"#$#%&:%;<!"#$#%&:%;( | $ $, 2 | ->A(: 52- | 0$ $, 2 6 / !72 + +2 - $+  ->A(: 0 |
| !"#$#%&:%;;!"#$#%&:%;)!"#$#%&:%;8  !"#$#%&:%;% | $ $ " | ->A(: 52- | +B ,7 0$ $ " 6 / !72 + +2 - $+ ->A(: 0 |
| !"#$#%&:%;' | $ $, 2 | ->A(: 52- | 0$ $, 2 6 / !72 + +2 - $+  ->A(: 0 |

!"#$#%&:%;9!"#$#%&:%;&!"#$#%&:%;:!"#$#%&:%)<!"#$#%&:%)(!"#$#%&:%);!"#$#%&:%))!"#$#%&:%)8!"#$#%&:%)%!"#$#%&:%)'!"#$#%&:%)9!"#$#%&:%)&!"#$#%&:%):!"#$#%&:%8<!"#$#%&:%8(!"#$#%&:%8;!"#$#%&:%8)!"#$#%&:%88!"#$#%&:%8%!"#$#%&:%8'!"#$#%&:%89!"#$#%&:%8&!"#$#%&:%8:!"#$#%&:%%<

!"#$#%&:%%(!"#$#%&:%%;!"#$#%&:%%)!"#$#%&:%%8!"#$#%&:%%%!"#$#%&:%%'!"#$#%&:%%9!"#$#%&:%%&!"#$#%&:%%:!"#$#%&:%'<!"#$#%&:%'(!"#$#%&:%';!"#$#%&:%')!"#$#%&:%'8!"#$#%&:%'%!"#$#%&:%''!"#$#%&:%'&!"#$#%&:%':!"#$#%&:%9<!"#$#%&:%9(

# $ $ " ->A(: +B ,7 0$ $ " 6 / !72 + +2 - $+

52- ->A(: 0

!"#$#%&:%9;!"#$#%&:%9)!"#$#%&:%98!"#$#%&:%9%!"#$#%&:%9'!"#$#%&:%99!"#$#%&:%9&!"#$#%&:%9:!"#$#%&:%&<!"#$#%&:%&(!"#$#%&:%&;!"#$#%&:%&)!"#$#%&:%&8!"#$#%&:%&%!"#$#%&:%&'!"#$#%&:%&9!"#$#%&:%&&!"#$#%&:%&:!"#$#%&:%:(!"#$#%&:%:;!"#$#%&:%:)!"#$#%&:%:8!"#$#%&:%:%!"#$#%&:%:' !"#$#%&:%:9!"#$#%&:%:&!"#$#%&:%::!"#$#%&:'<<!"#$#%&:'<(!"#$#%&:'<;!"#$#%&:'<)!"#$#%&:'<8!"#$#%&:'<%!"#$#%&:'<'!"#$#%&:'<9!"#$#%&:'<&!"#$#%&:'<:!"#$#%&:'(<!"#$#%&:'((!"#$#%&:'(;!"#$#%&:'()!"#$#%&:'(8!"#$#%&:'(%!"#$#%&:'('!"#$#%&:'(&!"#$#%&:';<!"#$#%&:';(!"#$#%&:';;

!"#$#%&:';)!"#$#%&:';8!"#$#%&:';%!"#$#%&:';'!"#$#%&:';9!"#$#%&:';&!"#$#%&:';:!"#$#%&:')<!"#$#%&:')(!"#$#%&:');!"#$#%&:'))!"#$#%&:')8!"#$#%&:')%!"#$#%&:')'!"#$#%&:')9!"#$#%&:')&!"#$#%&:'):!"#$#%&:'8<!"#$#%&:'8(!"#$#%&:'8;!"#$#%&:'8)!"#$#%&:'88!"#$#%&:'8%!"#$#%&:'8'

!"#$#%&:'89!"#$#%&:'8&!"#$#%&:'8:!"#$#%&:'%<!"#$#%&:'%(!"#$#%&:'%;!"#$#%&:'%)!"#$#%&:'%8!"#$#%&:'%%!"#$#%&:'%'!"#$#%&:'%9!"#$#%&:'%&!"#$#%&:'%:!"#$#%&:''<!"#$#%&:''(!"#$#%&:'';!"#$#%&:'')!"#$#%&:''8!"#$#%&:''%!"#$#%&:'''!"#$#%&:''9!"#$#%&:''&!"#$#%&:'':!"#$#%&:'9<

!"#$#%&:'9(!"#$#%&:'9;!"#$#%&:'9)!"#$#%&:'98!"#$#%&:'9%!"#$#%&:'9'!"#$#%&:'99!"#$#%&:'9&!"#$#%&:'9:!"#$#%&:'&<!"#$#%&:'&(!"#$#%&:'&;!"#$#%&:'&)!"#$#%&:'&8!"#$#%&:'&%!"#$#%&:'&'!"#$#%&:'&9!"#$#%&:'&&!"#$#%&:'&:!"#$#%&:':<!"#$#%&:':(!"#$#%&:':;!"#$#%&:':)!"#$#%&:':8

!"#$#%&:':%!"#$#%&:':'!"#$#%&:':9!"#$#%&:':&!"#$#%&:'::!"#$#%&:9<<!"#$#%&:9<;!"#$#%&:9<)!"#$#%&:9<8!"#$#%&:9<%!"#$#%&:9<'!"#$#%&:9<9!"#$#%&:9<&!"#$#%&:9<:!"#$#%&:9(<!"#$#%&:9((!"#$#%&:9(;!"#$#%&:9()!"#$#%&:9(8!"#$#%&:9(%!"#$#%&:9('!"#$#%&:9(9!"#$#%&:9(&!"#$#%&:9(:

!"#$#%&:9;<!"#$#%&:9;(!"#$#%&:9;;!"#$#%&:9;)!"#$#%&:9;8!"#$#%&:9;%!"#$#%&:9;'!"#$#%&:9;9!"#$#%&:9;&!"#$#%&:9;:!"#$#%&:9)<!"#$#%&:9)(!"#$#%&:9);!"#$#%&:9))!"#$#%&:9)8!"#$#%&:9)%!"#$#%&:9)'!"#$#%&:9)9!"#$#%&:9)&!"#$#%&:9):!"#$#%&:98<!"#$#%&:98(!"#$#%&:98;!"#$#%&:98)

!"#$#%&:988!"#$#%&:98%!"#$#%&:98'!"#$#%&:989!"#$#%&:98&!"#$#%&:98:!"#$#%&:9%<!"#$#%&:9%(!"#$#%&:9%;!"#$#%&:9%)!"#$#%&:9%8!"#$#%&:9%%!"#$#%&:9%'!"#$#%&:9%9!"#$#%&:9%:!"#$#%&:9'<!"#$#%&:9'(

|  | $ $, 2 | ->A(: 52- | 0$ $, 2 6 / !72 + +2 - $+ ->A(: 0?CDD DA@ |
| --- | --- | --- | --- |
| !"#$#%&:9'; | $ $ " | ->A(: 52- | +B ,7 0$ $ " 6 / !72 + +2 - $+ ->A(: 0?CDD DA@ |
| !"#$#%&:9')!"#$#%&:9'8 | $ $, 2 | ->A(: 52- | 0$ $, 2 6 / !72 + +2 - $+ ->A(: 0?CDD DA@ |
| !"#$#%&:9'% | $ $ " |  | +B ,7 0$ $ " 6 / !72 + +2 - $+ ->A(: 0?CDD DA@ |
| !"#$#%&:9''!"#$#%&:9'9!"#$#%&:9'&  !"#$#%&:9':!"#$#%&:99<!"#$#%&:99( !"#$#%&:99;!"#$#%&:99) | $ $, 2 |  | 0$ $, 2 6 / !72 + +2 - $+ ->A(: 0?CDD DA@ |

| !"#$#%&:998!"#$#%&:99%!"#$#%&:99'  !"#$#%&:999 | $ $ |  | 7> = 2- - 4 6 / !72 + +2 - $ + ->A(: 0?CDD DA@ |
| --- | --- | --- | --- |
| !"#$#%&:99&!"#$#%&:99:!"#$#%&:9&<  !"#$#%&:9&(!"#$#%&:9&;!"#$#%&:9&)  !"#$#%&:9&8!"#$#%&:9&%!"#$#%&:9&' | $ $ " |  | +B ,7 0$ $ " 6 / !72 + +2 - $+ ->A(: 0?CDD DA@ |
| !"#$#%&:9&9 | $ $, 2 | ->A(: 52- | 0$ $, 2 6 / !72 + +2 - $+ ->A(: 0?CDD DA@ |
| !"#$#%&:9&& | $ $ | ->A(: 52- | 7> = 2- - 4 6 / !72 + +2 - $ + ->A(: 0?CDD DA@ |
| !"#$#%&:9&: | $ $, 2 | ->A(: 52- | 0$ $, 2 6 / !72 + +2 - $+ ->A(: 0?CDD DA@ |
| !"#$#%&:9:< | $ $ | ->A(: 52- | 7> = 2- - 4 6 / !72 + +2 - $ + ->A(: 0?CDD DA@ |
| !"#$#%&:9:(!"#$#%&:9:; | $ $, 2 | ->A(: 52- | 0$ $, 2 6 / !72 + +2 - $+ ->A(: 0?CDD DA@ |
| !"#$#%&:9:) | $ $ | ->A(: 52- | 7> = 2- - 4 6 / !72 + +2 - $ + ->A(: 0?CDD DA@ |
| !"#$#%&:9:8!"#$#%&:9:%!"#$#%&:9:'  !"#$#%&:9:9!"#$#%&:9:&!"#$#%&:9:: !"#$#%&:&<<!"#$#%&:&<( | $ $, 2 | ->A(: 52- | 0$ $, 2 6 / !72 + +2 - $+ ->A(: 0?CDD DA@ |
| !"#$#%&:&<; | $ $ " | ->A(: 52- | +B ,7 0$ $ " 6 / !72 + +2 - $+ ->A(: 0?CDD DA@ |

!"#$#%&:&<)!"#$#%&:&<8!"#$#%&:&<%!"#$#%&:&<'!"#$#%&:&<9!"#$#%&:&<&!"#$#%&:&<:!"#$#%&:&(<!"#$#%&:&((!"#$#%&:&(;!"#$#%&:&()!"#$#%&:&(8!"#$#%&:&(%!"#$#%&:&('!"#$#%&:&(9!"#$#%&:&(&!"#$#%&:&(:!"#$#%&:&;<!"#$#%&:&;(!"#$#%&:&;;!"#$#%&:&;)!"#$#%&:&;8!"#$#%&:&;%!"#$#%&:&;' !"#$#%&:&;9!"#$#%&:&;&!"#$#%&:&;:!"#$#%&:&)<!"#$#%&:&)(!"#$#%&:&);!"#$#%&:&))!"#$#%&:&)8!"#$#%&:&)'!"#$#%&:&)9!"#$#%&:&)&!"#$#%&:&):!"#$#%&:&8<!"#$#%&:&8(!"#$#%&:&8;!"#$#%&:&8)!"#$#%&:&88!"#$#%&:&8%!"#$#%&:&8'!"#$#%&:&89!"#$#%&:&8&!"#$#%&:&8:!"#$#%&:&%<!"#$#%&:&%( !"#$#%&:&%;!"#$#%&:&%)!"#$#%&:&%8!"#$#%&:&%%!"#$#%&:&%'!"#$#%&:&%9!"#$#%&:&%&!"#$#%&:&%:!"#$#%&:&'<!"#$#%&:&'(!"#$#%&:&';!"#$#%&:&')!"#$#%&:&'8!"#$#%&:&'%!"#$#%&:&''!"#$#%&:&'9!"#$#%&:&'&!"#$#%&:&':!"#$#%&:&9<!"#$#%&:&9(!"#$#%&:&9;!"#$#%&:&9)!"#$#%&:&98!"#$#%&:&9%

!"#$#%&:&9'!"#$#%&:&99!"#$#%&:&9&!"#$#%&:&9:!"#$#%&:&&<!"#$#%&:&&(!"#$#%&:&&;!"#$#%&:&&)!"#$#%&:&&8!"#$#%&:&&%!"#$#%&:&&'!"#$#%&:&&9!"#$#%&:&&&!"#$#%&:&&:!"#$#%&:&:<!"#$#%&:&:;!"#$#%&:&:)!"#$#%&:&:8!"#$#%&:&:%!"#$#%&:&:'!"#$#%&:&:9!"#$#%&:&:&!"#$#%&:&::!"#$#%&::<<

!"#$#%&::<(!"#$#%&::<;!"#$#%&::<)!"#$#%&::<%!"#$#%&::<9!"#$#%&::<&!"#$#%&::<:!"#$#%&::(<!"#$#%&::((!"#$#%&::(;!"#$#%&::(8!"#$#%&::(%!"#$#%&::('!"#$#%&::(9!"#$#%&::(&!"#$#%&::(:!"#$#%&::;<!"#$#%&::;(!"#$#%&::;;!"#$#%&::;)!"#$#%&::;8!"#$#%&::;%!"#$#%&::;'!"#$#%&::;9

!"#$#%&::;&!"#$#%&::;:!"#$#%&::)<!"#$#%&::)(!"#$#%&::);!"#$#%&::))!"#$#%&::)8!"#$#%&::)%!"#$#%&::)9!"#$#%&::)&!"#$#%&::):!"#$#%&::8<!"#$#%&::8(!"#$#%&::8;!"#$#%&::8)!"#$#%&::88!"#$#%&::8%!"#$#%&::8'!"#$#%&::89!"#$#%&::8&!"#$#%&::8:!"#$#%&::%<!"#$#%&::%(!"#$#%&::%;

!"#$#%&::%)!"#$#%&::%8!"#$#%&::%%!"#$#%&::%'!"#$#%&::%9!"#$#%&::%&!"#$#%&::%:!"#$#%&::'<!"#$#%&::'(!"#$#%&::';!"#$#%&::')!"#$#%&::'%!"#$#%&::''!"#$#%&::'9!"#$#%&::'&!"#$#%&::':!"#$#%&::9<!"#$#%&::9(!"#$#%&::9;!"#$#%&::9)!"#$#%&::9%!"#$#%&::9'!"#$#%&::99!"#$#%&::9&

!"#$#%&::9:!"#$#%&::&<!"#$#%&::&(!"#$#%&::&;!"#$#%&::&)!"#$#%&::&8!"#$#%&::&%!"#$#%&::&'!"#$#%&::&9!"#$#%&::&&!"#$#%&::&:!"#$#%&:::<!"#$#%&:::(!"#$#%&:::;!"#$#%&:::)!"#$#%&:::8!"#$#%&:::%!"#$#%&:::'!"#$#%&:::9!"#$#%&:::&!"#$#%&::::!"#$#%:<<<<!"#$#%:<<<(!"#$#%:<<<; !"#$#%:<<<)!"#$#%:<<<8!"#$#%:<<<%!"#$#%:<<<'!"#$#%:<<<9!"#$#%:<<<:!"#$#%:<<(<!"#$#%:<<((!"#$#%:<<(;!"#$#%:<<()!"#$#%:<<(8!"#$#%:<<(%!"#$#%:<<('!"#$#%:<<(9!"#$#%:<<(&!"#$#%:<<(:!"#$#%:<<;<!"#$#%:<<;(!"#$#%:<<;;!"#$#%:<<;)!"#$#%:<<;8!"#$#%:<<;%!"#$#%:<<;'!"#$#%:<<;9

!"#$#%:<<;&!"#$#%:<<;:!"#$#%:<<)<!"#$#%:<<)(!"#$#%:<<);!"#$#%:<<))!"#$#%:<<)8!"#$#%:<<)%!"#$#%:<<)'!"#$#%:<<)9!"#$#%:<<)&!"#$#%:<<):!"#$#%:<<8<!"#$#%:<<8(!"#$#%:<<8;!"#$#%:<<8)!"#$#%:<<88!"#$#%:<<8%!"#$#%:<<8&!"#$#%:<<8:!"#$#%:<<%<!"#$#%:<<%(!"#$#%:<<%;!"#$#%:<<%) !"#$#%:<<%8!"#$#%:<<%%!"#$#%:<<%'!"#$#%:<<%9!"#$#%:<<%&!"#$#%:<<%:!"#$#%:<<'<!"#$#%:<<'(!"#$#%:<<';!"#$#%:<<'8!"#$#%:<<'%!"#$#%:<<''!"#$#%:<<'9!"#$#%:<<'&!"#$#%:<<':!"#$#%:<<9<!"#$#%:<<9(!"#$#%:<<9;!"#$#%:<<9)!"#$#%:<<98!"#$#%:<<9%!"#$#%:<<9'!"#$#%:<<99!"#$#%:<<9& !"#$#%:<<9:!"#$#%:<<&<!"#$#%:<<&(!"#$#%:<<&;!"#$#%:<<&)!"#$#%:<<&8!"#$#%:<<&%!"#$#%:<<&'!"#$#%:<<&9!"#$#%:<<&&!"#$#%:<<&:!"#$#%:<<:<!"#$#%:<<:(!"#$#%:<<:;!"#$#%:<<:)!"#$#%:<<:8!"#$#%:<<:%!"#$#%:<<:'!"#$#%:<<:9!"#$#%:<<:&!"#$#%:<<::!"#$#%:<(<<!"#$#%:<(<(!"#$#%:<(<;

!"#$#%:<(<)!"#$#%:<(<8!"#$#%:<(<%!"#$#%:<(<9!"#$#%:<(<&!"#$#%:<(<:!"#$#%:<((<!"#$#%:<(((!"#$#%:<((;!"#$#%:<(()!"#$#%:<((8!"#$#%:<((%!"#$#%:<(('!"#$#%:<((9!"#$#%:<((&!"#$#%:<((:!"#$#%:<(;<!"#$#%:<(;(!"#$#%:<(;;!"#$#%:<(;)!"#$#%:<(;8!"#$#%:<(;%!"#$#%:<(;'!"#$#%:<(;9

!"#$#%:<(;&!"#$#%:<(;:!"#$#%:<()<!"#$#%:<()(!"#$#%:<();!"#$#%:<())!"#$#%:<()8!"#$#%:<()%!"#$#%:<()'!"#$#%:<()9!"#$#%:<()&!"#$#%:<():!"#$#%:<(8<!"#$#%:<(8(!"#$#%:<(8;!"#$#%:<(88!"#$#%:<(8%!"#$#%:<(8'!"#$#%:<(89!"#$#%:<(8&!"#$#%:<(8:!"#$#%:<(%<!"#$#%:<(%(!"#$#%:<(%; !"#$#%:<(%)!"#$#%:<(%8!"#$#%:<(%%!"#$#%:<(%'!"#$#%:<(%9!"#$#%:<(%&!"#$#%:<(%:!"#$#%:<('<!"#$#%:<('(!"#$#%:<(';!"#$#%:<(')!"#$#%:<('8!"#$#%:<('%!"#$#%:<(''!"#$#%:<('9!"#$#%:<('&!"#$#%:<(':!"#$#%:<(9<!"#$#%:<(9(

|  | $ $ | ->A(: 52- | 7> = 2- - 4 6 / !72 + +2 - $ + ->A(: 0?CDD DA@ |
| --- | --- | --- | --- |
| !"#$#%:<(9; | $ $, 2 | ->A(: 52- | 0$ $, 2 6 / !72 + +2 - $+ ->A(: 0?CDD DA@ |
| !"#$#%:<(9) | $ $- | ->A(: 52- | /70$ $- 6 / !72 + +2 - $+ ->A(: 0?CDD DA@ |
| !"#$#%:<(98!"#$#%:<(9% | $ $, 2 | ->A(: 52- | 0$ $, 2 6 / !72 + +2 - $+ ->A(: 0?CDD DA@ |
| !"#$#%:<(9'!"#$#%:<(99!"#$#%:<(9& !"#$#%:<(9:!"#$#%:<(&< | $ $ | ->A(: 52- | 7> = 2- - 4 6 / !72 + +2 - $ + ->A(: 0?CDD DA@ |
| !"#$#%:<(&( | $ $ " | ->A(: 52- | +B ,7 0$ $ " 6 / !72 + +2 - $+ ->A(: 0?CDD DA@ |
| !"#$#%:<(&;!"#$#%:<(&)!"#$#%:<(&8  !"#$#%:<(&% | $ $ | ->A(: 52- | 7> = 2- - 4 6 / !72 + +2 - $ + ->A(: 0?CDD DA@ |
| !"#$#%:<(&' | $ $ " | ->A(: 52- | +B ,7 0$ $ " 6 / !72 + +2 - $+ ->A(: 0?CDD DA@ |
| !"#$#%:<(&9!"#$#%:<(&&!"#$#%:<(&: !"#$#%:<(:<!"#$#%:<(:(!"#$#%:<(:; | $ $ | ->A(: 52- | 7> = 2- - 4 6 / !72 + +2 - $ + ->A(: 0?CDD DA@ |
| !"#$#%:<(:)!"#$#%:<(:8 | $ $ " | ->A(: 52- | +B ,7 0$ $ " 6 / !72 + +2 - $+ ->A(: 0?CDD DA@ |
| !"#$#%:<(:%!"#$#%:<(:'!"#$#%:<(:9 | $ $ | ->A(: 52- | 7> = 2- - 4 6 / !72 + +2 - $ + ->A(: 0?CDD DA@ |
| !"#$#%:<(:& | $ $ " | ->A(: 52- | +B ,7 0$ $ " 6 / !72 + +2 - $+ ->A(: 0?CDD DA@ |
| !"#$#%:<(:: | $ $ | ->A(: 52- | 7> = 2- - 4 6 / !72 + +2 - $ + ->A(: 0?CDD DA@ |
| !"#$#%:<;<< | $ $ " | ->A(: 52- | +B ,7 0$ $ " 6 / !72 + +2 - $+ ->A(: 0?CDD DA@ |
| !"#$#%:<;<(!"#$#%:<;<;!"#$#%:<;<) !"#$#%:<;<8!"#$#%:<;<% | $ $ | ->A(: 52- | 7> = 2- - 4 6 / !72 + +2 - $ + ->A(: 0?CDD DA@ |
| !"#$#%:<;<' | $ $ " | ->A(: 52- | +B ,7 0$ $ " 6 / !72 + +2 - $+ ->A(: 0?CDD DA@ |
| !"#$#%:<;<9!"#$#%:<;<&!"#$#%:<;<: | $ $ | ->A(: 52- | 7> = 2- - 4 6 / !72 + +2 - $ + ->A(: 0?CDD DA@ |
| !"#$#%:<;(< | $ $ " | ->A(: 52- | +B ,7 0$ $ " 6 / !72 + +2 - $+ ->A(: 0?CDD DA@ |
| !"#$#%:<;((!"#$#%:<;(; | $ $ | ->A(: 52- | 7> = 2- - 4 6 / !72 + +2 - $ + ->A(: 0?CDD DA@ |
| !"#$#%:<;()!"#$#%:<;(8!"#$#%:<;(% | $ $ " | ->A(: 52- | +B ,7 0$ $ " 6 / !72 + +2 - $+ ->A(: 0?CDD DA@ |
| !"#$#%:<;('!"#$#%:<;(9!"#$#%:<;(& !"#$#%:<;(: | $ $ | ->A(: 52- | 7> = 2- - 4 6 / !72 + +2 - $ + ->A(: 0?CDD DA@ |
| !"#$#%:<;;< | $ $ " | ->A(: 52- | +B ,7 0$ $ " 6 / !72 + +2 - $+ ->A(: 0?CDD DA@ |
| !"#$#%:<;;(!"#$#%:<;;;!"#$#%:<;;) | $ $ | ->A(: 52- | 7> = 2- - 4 6 / !72 + +2 - $ + ->A(: 0?CDD DA@ |
| !"#$#%:<;;8!"#$#%:<;;%!"#$#%:<;;' | $ $ " | ->A(: 52- | +B ,7 0$ $ " 6 / !72 + +2 - $+ ->A(: 0?CDD DA@ |
| !"#$#%:<;;9 | $ $ | ->A(: 52- | 7> = 2- - 4 6 / !72 + +2 - $ + ->A(: 0?CDD DA@ |
| !"#$#%:<;;& | $ $ " | ->A(: 52- | +B ,7 0$ $ " 6 / !72 + +2 - $+ ->A(: 0?CDD DA@ |
| !"#$#%:<;;:!"#$#%:<;)<!"#$#%:<;)( !"#$#%:<;);!"#$#%:<;)) | $ $ |  | 7> = 2- - 4 6 / !72 + +2 - $ + ->A(: 0?CDD DA@ |

!"#$#%:<;)8!"#$#%:<;)% $ $ "

+B ,7 0$ $ " 6 / !72 + +2 - $+

->A(: 0?CDD DA@

!"#$#%:<))' $ $

| !"#$#%:<;)'!"#$#%:<;)9!"#$#%:<;)& | $ $ |  | 7> = 2- - 4 6 / !72 + +2 - $ + ->A(: 0?CDD DA@ |
| --- | --- | --- | --- |
| !"#$#%:<;): | $ $ " |  | +B ,7 0$ $ " 6 / !72 + +2 - $+ ->A(: 0?CDD DA@ |
| !"#$#%:<;8<!"#$#%:<;8(!"#$#%:<;8; !"#$#%:<;8)!"#$#%:<;88!"#$#%:<;8% | $ $ | ->A(: 52- | 7> = 2- - 4 6 / !72 + +2 - $ + ->A(: 0?CDD DA@ |
| !"#$#%:<;8' | $ $ " | ->A(: 52- | +B ,7 0$ $ " 6 / !72 + +2 - $+ ->A(: 0?CDD DA@ |
| !"#$#%:<;89!"#$#%:<;8&!"#$#%:<;8: | $ $ | ->A(: 52- | 7> = 2- - 4 6 / !72 + +2 - $ + ->A(: 0?CDD DA@ |
| !"#$#%:<;%< | $ $ " | ->A(: 52- | +B ,7 0$ $ " 6 / !72 + +2 - $+ ->A(: 0?CDD DA@ |
| !"#$#%:<;%(!"#$#%:<;%;!"#$#%:<;%)  !"#$#%:<;%8 | $ $ | ->A(: 52- | 7> = 2- - 4 6 / !72 + +2 - $ + ->A(: 0?CDD DA@ |
| !"#$#%:<;%% | $ $ " | ->A(: 52- | +B ,7 0$ $ " 6 / !72 + +2 - $+ ->A(: 0?CDD DA@ |
| !"#$#%:<;%' | $ $ | ->A(: 52- | 7> = 2- - 4 6 / !72 + +2 - $ + ->A(: 0?CDD DA@ |
| !"#$#%:<;%9 | $ $ " | ->A(: 52- | +B ,7 0$ $ " 6 / !72 + +2 - $+ ->A(: 0?CDD DA@ |
| !"#$#%:<;%& | $ $ | ->A(: 52- | 7> = 2- - 4 6 / !72 + +2 - $ + ->A(: 0?CDD DA@ |
| !"#$#%:<;%:!"#$#%:<;'<!"#$#%:<;'( | $ $ " | ->A(: 52- | +B ,7 0$ $ " 6 / !72 + +2 - $+ ->A(: 0?CDD DA@ |
| !"#$#%:<;'; | $ $ | ->A(: 52- | 7> = 2- - 4 6 / !72 + +2 - $ + ->A(: 0?CDD DA@ |
| !"#$#%:<;') | $ $ " | ->A(: 52- | +B ,7 0$ $ " 6 / !72 + +2 - $+ ->A(: 0?CDD DA@ |
| !"#$#%:<;'8 | $ $ | ->A(: 52- | 7> = 2- - 4 6 / !72 + +2 - $ + ->A(: 0?CDD DA@ |
| !"#$#%:<;'% | $ $ " | ->A(: 52- | +B ,7 0$ $ " 6 / !72 + +2 - $+ ->A(: 0?CDD DA@ |
| !"#$#%:<;''!"#$#%:<;'9!"#$#%:<;'& !"#$#%:<;':!"#$#%:<;9<!"#$#%:<;9( | $ $ | ->A(: 52- | 7> = 2- - 4 6 / !72 + +2 - $ + ->A(: 0?CDD DA@ |
| !"#$#%:<;9; | $ $ " | ->A(: 52- | +B ,7 0$ $ " 6 / !72 + +2 - $+ ->A(: 0?CDD DA@ |
| !"#$#%:<;9) | $ $ | ->A(: 52- | 7> = 2- - 4 6 / !72 + +2 - $ + ->A(: 0?CDD DA@ |
| !"#$#%:<;98!"#$#%:<;9%!"#$#%:<;9'  !"#$#%:<;99 | $ $ " | ->A(: 52- | +B ,7 0$ $ " 6 / !72 + +2 - $+ ->A(: 0?CDD DA@ |
| !"#$#%:<;9&!"#$#%:<;9:!"#$#%:<;&<  !"#$#%:<;&( | $ $ | ->A(: 52- | 7> = 2- - 4 6 / !72 + +2 - $ + ->A(: 0?CDD DA@ |
| !"#$#%:<;&;!"#$#%:<;&)!"#$#%:<;&8 | $ $ " | ->A(: 52- | +B ,7 0$ $ " 6 / !72 + +2 - $+ ->A(: 0?CDD DA@ |
| !"#$#%:<;&% | $ $ | ->A(: 52- | 7> = 2- - 4 6 / !72 + +2 - $ + ->A(: 0?CDD DA@ |
| !"#$#%:<;&' | $ $ " | ->A(: 52- | +B ,7 0$ $ " 6 / !72 + +2 - $+ ->A(: 0?CDD DA@ |
| !"#$#%:<;&9 | $ $ | ->A(: 52- | 7> = 2- - 4 6 / !72 + +2 - $ + ->A(: 0?CDD DA@ |
| !"#$#%:<;&& | $ $ " | ->A(: 52- | +B ,7 0$ $ " 6 / !72 + +2 - $+ ->A(: 0?CDD DA@ |
| !"#$#%:<;&:!"#$#%:<;:<!"#$#%:<;:( !"#$#%:<;:;!"#$#%:<;:)!"#$#%:<;:8 | $ $ | ->A(: 52- | 7> = 2- - 4 6 / !72 + +2 - $ + ->A(: 0?CDD DA@ |
| !"#$#%:<;:%!"#$#%:<;:' | $ $ " | ->A(: 52- | +B ,7 0$ $ " 6 / !72 + +2 - $+ ->A(: 0?CDD DA@ |
| !"#$#%:<;:9!"#$#%:<;:&!"#$#%:<;:: | $ $ | ->A(: 52- | 7> = 2- - 4 6 / !72 + +2 - $ + ->A(: 0?CDD DA@ |
| !"#$#%:<)<< | $ $ " | ->A(: 52- | +B ,7 0$ $ " 6 / !72 + +2 - $+ ->A(: 0?CDD DA@ |
| !"#$#%:<)<(!"#$#%:<)<;!"#$#%:<)<) !"#$#%:<)<8!"#$#%:<)<% | $ $ | ->A(: 52- | 7> = 2- - 4 6 / !72 + +2 - $ + ->A(: 0?CDD DA@ |
| !"#$#%:<)<'!"#$#%:<)<9 | $ $ " | ->A(: 52- | +B ,7 0$ $ " 6 / !72 + +2 - $+ ->A(: 0?CDD DA@ |
| !"#$#%:<)<& | $ $ | ->A(: 52- | 7> = 2- - 4 6 / !72 + +2 - $ + ->A(: 0?CDD DA@ |
| !"#$#%:<)<: | $ $ " | ->A(: 52- | +B ,7 0$ $ " 6 / !72 + +2 - $+ ->A(: 0?CDD DA@ |
| !"#$#%:<)(<!"#$#%:<)((!"#$#%:<)(;  !"#$#%:<)() | $ $ | ->A(: 52- | 7> = 2- - 4 6 / !72 + +2 - $ + ->A(: 0?CDD DA@ |
| !"#$#%:<)(8 | $ $ " | ->A(: 52- | +B ,7 0$ $ " 6 / !72 + +2 - $+ ->A(: 0?CDD DA@ |
| !"#$#%:<)(%!"#$#%:<)('!"#$#%:<)(9  !"#$#%:<)(& | $ $ | ->A(: 52- | 7> = 2- - 4 6 / !72 + +2 - $ + ->A(: 0?CDD DA@ |
| !"#$#%:<)(: | $ $ " | ->A(: 52- | +B ,7 0$ $ " 6 / !72 + +2 - $+ ->A(: 0?CDD DA@ |
| !"#$#%:<);< | $ $ | ->A(: 52- | 7> = 2- - 4 6 / !72 + +2 - $ + ->A(: 0?CDD DA@ |
| !"#$#%:<);( | $ $ " | ->A(: 52- | +B ,7 0$ $ " 6 / !72 + +2 - $+ ->A(: 0?CDD DA@ |
| !"#$#%:<);;!"#$#%:<);)!"#$#%:<);8 !"#$#%:<);%!"#$#%:<);' | $ $ | ->A(: 52- | 7> = 2- - 4 6 / !72 + +2 - $ + ->A(: 0?CDD DA@ |
| !"#$#%:<);9!"#$#%:<);&!"#$#%:<);: | $ $ " | ->A(: 52- | +B ,7 0$ $ " 6 / !72 + +2 - $+ ->A(: 0?CDD DA@ |
| !"#$#%:<))<!"#$#%:<))(!"#$#%:<)); !"#$#%:<)))!"#$#%:<))8 | $ $ | ->A(: 52- | 7> = 2- - 4 6 / !72 + +2 - $ + ->A(: 0?CDD DA@ |
| !"#$#%:<))% | $ $ " |  | +B ,7 0$ $ " 6 / !72 + +2 - $+ |

->A(: 0?CDD DA@

7> = 2- - 4 6 / !72 + +2 - $

+ ->A(: 0?CDD DA@

| !"#$#%:<))9 | $ $ " |  | +B ,7 0$ $ " 6 / !72 + +2 - $+ ->A(: 0?CDD DA@ |
| --- | --- | --- | --- |
| !"#$#%:<))&!"#$#%:<)):!"#$#%:<)8<  !"#$#%:<)8(!"#$#%:<)8;!"#$#%:<)8) !"#$#%:<)88!"#$#%:<)8% | $ $ |  | 7> = 2- - 4 6 / !72 + +2 - $ + ->A(: 0?CDD DA@ |
| !"#$#%:<)8'!"#$#%:<)89 | $ $ " | ->A(: 52- | +B ,7 0$ $ " 6 / !72 + +2 - $+ ->A(: 0?CDD DA@ |
| !"#$#%:<)8&!"#$#%:<)8:!"#$#%:<)%< | $ $ | ->A(: 52- | 7> = 2- - 4 6 / !72 + +2 - $ + ->A(: 0?CDD DA@ |
| !"#$#%:<)%(!"#$#%:<)%; | $ $ " | ->A(: 52- | +B ,7 0$ $ " 6 / !72 + +2 - $+ ->A(: 0?CDD DA@ |
| !"#$#%:<)%)!"#$#%:<)%8 | $ $ | ->A(: 52- | 7> = 2- - 4 6 / !72 + +2 - $ + ->A(: 0?CDD DA@ |
| !"#$#%:<)%% | $ $ " | ->A(: 52- | +B ,7 0$ $ " 6 / !72 + +2 - $+ ->A(: 0?CDD DA@ |
| !"#$#%:<)%' | $ $ | ->A(: 52- | 7> = 2- - 4 6 / !72 + +2 - $ + ->A(: 0?CDD DA@ |
| !"#$#%:<)%9 | $ $ " | ->A(: 52- | +B ,7 0$ $ " 6 / !72 + +2 - $+ ->A(: 0?CDD DA@ |
| !"#$#%:<)%& | $ $ | ->A(: 52- | 7> = 2- - 4 6 / !72 + +2 - $ + ->A(: 0?CDD DA@ |
| !"#$#%:<)%: | $ $ " | ->A(: 52- | +B ,7 0$ $ " 6 / !72 + +2 - $+ ->A(: 0?CDD DA@ |
| !"#$#%:<)'< | $ $ | ->A(: 52- | 7> = 2- - 4 6 / !72 + +2 - $ + ->A(: 0?CDD DA@ |
| !"#$#%:<)'( | $ $ " | ->A(: 52- | +B ,7 0$ $ " 6 / !72 + +2 - $+ ->A(: 0?CDD DA@ |
| !"#$#%:<)';!"#$#%:<)') | $ $ | ->A(: 52- | 7> = 2- - 4 6 / !72 + +2 - $ + ->A(: 0?CDD DA@ |
| !"#$#%:<)'8 | $ $ " | ->A(: 52- | +B ,7 0$ $ " 6 / !72 + +2 - $+ ->A(: 0?CDD DA@ |
| !"#$#%:<)'% | $ $ | ->A(: 52- | 7> = 2- - 4 6 / !72 + +2 - $ + ->A(: 0?CDD DA@ |
| !"#$#%:<)''!"#$#%:<)'9 | $ $ " | ->A(: 52- | +B ,7 0$ $ " 6 / !72 + +2 - $+ ->A(: 0?CDD DA@ |
| !"#$#%:<)'& | $ $ | ->A(: 52- | 7> = 2- - 4 6 / !72 + +2 - $ + ->A(: 0?CDD DA@ |
| !"#$#%:<)': | $ $ " | ->A(: 52- | +B ,7 0$ $ " 6 / !72 + +2 - $+ ->A(: 0?CDD DA@ |
| !"#$#%:<)9<!"#$#%:<)9(!"#$#%:<)9; | $ $ | ->A(: 52- | 7> = 2- - 4 6 / !72 + +2 - $ + ->A(: 0?CDD DA@ |
| !"#$#%:<)9) | $ $ " | ->A(: 52- | +B ,7 0$ $ " 6 / !72 + +2 - $+ ->A(: 0?CDD DA@ |
| !"#$#%:<)98!"#$#%:<)9%!"#$#%:<)9' !"#$#%:<)99!"#$#%:<)9& | $ $ | ->A(: 52- | 7> = 2- - 4 6 / !72 + +2 - $ + ->A(: 0?CDD DA@ |
| !"#$#%:<)9:!"#$#%:<)&< | $ $ " | ->A(: 52- | +B ,7 0$ $ " 6 / !72 + +2 - $+ ->A(: 0?CDD DA@ |
| !"#$#%:<)&(!"#$#%:<)&; | $ $ | ->A(: 52- | 7> = 2- - 4 6 / !72 + +2 - $ + ->A(: 0?CDD DA@ |
| !"#$#%:<)&) | $ $ " | ->A(: 52- | +B ,7 0$ $ " 6 / !72 + +2 - $+ ->A(: 0?CDD DA@ |
| !"#$#%:<)&8!"#$#%:<)&% | $ $ | ->A(: 52- | 7> = 2- - 4 6 / !72 + +2 - $ + ->A(: 0?CDD DA@ |
| !"#$#%:<)&' | $ $ " | ->A(: 52- | +B ,7 0$ $ " 6 / !72 + +2 - $+ ->A(: 0?CDD DA@ |
| !"#$#%:<)&9 | $ $ | ->A(: 52- | 7> = 2- - 4 6 / !72 + +2 - $ + ->A(: 0?CDD DA@ |
| !"#$#%:<)&& | $ $ " | ->A(: 52- | +B ,7 0$ $ " 6 / !72 + +2 - $+ ->A(: 0?CDD DA@ |
| !"#$#%:<)&: | $ $ | ->A(: 52- | 7> = 2- - 4 6 / !72 + +2 - $ + ->A(: 0?CDD DA@ |
| !"#$#%:<):< | $ $ " | ->A(: 52- | +B ,7 0$ $ " 6 / !72 + +2 - $+ ->A(: 0?CDD DA@ |
| !"#$#%:<):( | $ $ | ->A(: 52- | 7> = 2- - 4 6 / !72 + +2 - $ + ->A(: 0?CDD DA@ |
| !"#$#%:<):; | $ $ " | ->A(: 52- | +B ,7 0$ $ " 6 / !72 + +2 - $+ ->A(: 0?CDD DA@ |
| !"#$#%:<):)!"#$#%:<):8!"#$#%:<):%  !"#$#%:<):' | $ $ | ->A(: 52- | 7> = 2- - 4 6 / !72 + +2 - $ + ->A(: 0?CDD DA@ |
| !"#$#%:<):9!"#$#%:<):&!"#$#%:<):: | $ $ " | ->A(: 52- | +B ,7 0$ $ " 6 / !72 + +2 - $+ ->A(: 0?CDD DA@ |
| !"#$#%:<8<<!"#$#%:<8<(!"#$#%:<8<; | $ $ | ->A(: 52- | 7> = 2- - 4 6 / !72 + +2 - $ + ->A(: 0?CDD DA@ |
| !"#$#%:<8<) | $ $ " | ->A(: 52- | +B ,7 0$ $ " 6 / !72 + +2 - $+ ->A(: 0?CDD DA@ |
| !"#$#%:<8<8 | $ $ | ->A(: 52- | 7> = 2- - 4 6 / !72 + +2 - $ + ->A(: 0?CDD DA@ |
| !"#$#%:<8<% | $ $ " | ->A(: 52- | +B ,7 0$ $ " 6 / !72 + +2 - $+ ->A(: 0?CDD DA@ |

|  | $ $ | ->A(: 52- | 7> = 2- - 4 6 / !72 + +2 - $ + ->A(: 0?CDD DA@ |
| --- | --- | --- | --- |
| !"#$#%:<8)& | $ $ " | ->A(: 52- | +B ,7 0$ $ " 6 / !72 + +2 - $+ ->A(: 0?CDD DA@ |
| !"#$#%:<8):!"#$#%:<88< | $ $ | ->A(: | 7> = 2- - 4 6 / !72 + +2 - $ |

!"#$#%:<8<'!"#$#%:<8<9!"#$#%:<8<&!"#$#%:<8<:!"#$#%:<8(<!"#$#%:<8((!"#$#%:<8(;!"#$#%:<8()!"#$#%:<8(8!"#$#%:<8(%!"#$#%:<8('!"#$#%:<8(9!"#$#%:<8(&!"#$#%:<8;<!"#$#%:<8;(

|  | $ $ | ->A(: 52- | 7> = 2- - 4 6 / !72 + +2 - $ + ->A(: 0?CDD DA@ |
| --- | --- | --- | --- |
| !"#$#%:<8;;!"#$#%:<8;) | $ $ " | ->A(: | +B ,7 0$ $ " 6 / !72 + +2 - $+ |

52- ->A(: 0?CDD DA@

!"#$#%:<8;8!"#$#%:<8;%!"#$#%:<8;'!"#$#%:<8;9!"#$#%:<8;&!"#$#%:<8;:!"#$#%:<8)<!"#$#%:<8)(!"#$#%:<8);!"#$#%:<8))!"#$#%:<8)8!"#$#%:<8)%!"#$#%:<8)'!"#$#%:<8)9

|  |  | 52- | + ->A(: 0?CDD DA@ |
| --- | --- | --- | --- |
| !"#$#%:<88(!"#$#%:<88; | $ $ " | ->A(: 52- | +B ,7 0$ $ " 6 / !72 + +2 - $+ ->A(: 0?CDD DA@ |
| !"#$#%:<88)!"#$#%:<888!"#$#%:<88% | $ $ | ->A(: 52- | 7> = 2- - 4 6 / !72 + +2 - $ + ->A(: 0?CDD DA@ |
| !"#$#%:<88' | $ $ " | ->A(: 52- | +B ,7 0$ $ " 6 / !72 + +2 - $+ ->A(: 0?CDD DA@ |
| !"#$#%:<889 | $ $ | ->A(: 52- | 7> = 2- - 4 6 / !72 + +2 - $ + ->A(: 0?CDD DA@ |
| !"#$#%:<88& | $ $ " | ->A(: 52- | +B ,7 0$ $ " 6 / !72 + +2 - $+ ->A(: 0?CDD DA@ |
| !"#$#%:<88:!"#$#%:<8%<!"#$#%:<8%( | $ $ | ->A(: 52- | 7> = 2- - 4 6 / !72 + +2 - $ + ->A(: 0?CDD DA@ |
| !"#$#%:<8%;!"#$#%:<8%) | $ $ " | ->A(: 52- | +B ,7 0$ $ " 6 / !72 + +2 - $+ ->A(: 0?CDD DA@ |
| !"#$#%:<8%8!"#$#%:<8%%!"#$#%:<8%'  !"#$#%:<8%9!"#$#%:<8%&!"#$#%:<8%: !"#$#%:<8'<!"#$#%:<8'( | $ $ | ->A(: 52- | 7> = 2- - 4 6 / !72 + +2 - $ + ->A(: 0?CDD DA@ |
| !"#$#%:<8'; | $ $ " | ->A(: 52- | +B ,7 0$ $ " 6 / !72 + +2 - $+ ->A(: 0?CDD DA@ |

!"#$#%:<8')!"#$#%:<8'8!"#$#%:<8'%!"#$#%:<8''!"#$#%:<8'9!"#$#%:<8'&!"#$#%:<8':!"#$#%:<89<!"#$#%:<89(!"#$#%:<89;!"#$#%:<89)!"#$#%:<898!"#$#%:<89%!"#$#%:<89'!"#$#%:<899!"#$#%:<89&!"#$#%:<89:!"#$#%:<8&<!"#$#%:<8&(!"#$#%:<8&;!"#$#%:<8&)!"#$#%:<8&8!"#$#%:<8&%!"#$#%:<8&'

!"#$#%:<8&9!"#$#%:<8&&!"#$#%:<8&:!"#$#%:<8:<!"#$#%:<8:(!"#$#%:<8:;!"#$#%:<8:)!"#$#%:<8:8!"#$#%:<8:%!"#$#%:<8:'!"#$#%:<8:&!"#$#%:<8::!"#$#%:<%<<!"#$#%:<%<(!"#$#%:<%<;!"#$#%:<%<)!"#$#%:<%<8!"#$#%:<%<%!"#$#%:<%<'!"#$#%:<%<9!"#$#%:<%<&!"#$#%:<%<:!"#$#%:<%(<!"#$#%:<%((

!"#$#%:<%(;!"#$#%:<%()!"#$#%:<%(8!"#$#%:<%(%!"#$#%:<%('!"#$#%:<%(9!"#$#%:<%(&!"#$#%:<%(:!"#$#%:<%;<!"#$#%:<%;(!"#$#%:<%;;!"#$#%:<%;)!"#$#%:<%;8!"#$#%:<%;%!"#$#%:<%;'!"#$#%:<%;9!"#$#%:<%;&!"#$#%:<%;:!"#$#%:<%)<!"#$#%:<%)(!"#$#%:<%);!"#$#%:<%))!"#$#%:<%)8!"#$#%:<%)%

!"#$#%:<%)'!"#$#%:<%)9!"#$#%:<%)&!"#$#%:<%):!"#$#%:<%8<!"#$#%:<%8(!"#$#%:<%8;!"#$#%:<%8)!"#$#%:<%88!"#$#%:<%8%!"#$#%:<%8'!"#$#%:<%89!"#$#%:<%8&!"#$#%:<%8:!"#$#%:<%%<!"#$#%:<%%(!"#$#%:<%%;!"#$#%:<%%)!"#$#%:<%%8!"#$#%:<%%%!"#$#%:<%%'!"#$#%:<%%9!"#$#%:<%%&!"#$#%:<%%:

!"#$#%:<%'<!"#$#%:<%'(!"#$#%:<%';!"#$#%:<%')!"#$#%:<%'8!"#$#%:<%'%!"#$#%:<%''!"#$#%:<%'9!"#$#%:<%'&!"#$#%:<%':!"#$#%:<%9<!"#$#%:<%9(!"#$#%:<%9;!"#$#%:<%9)!"#$#%:<%98!"#$#%:<%9%!"#$#%:<%9'!"#$#%:<%99!"#$#%:<%9&!"#$#%:<%9:!"#$#%:<%&<!"#$#%:<%&(!"#$#%:<%&;!"#$#%:<%&)

!"#$#%:<%&8!"#$#%:<%&%!"#$#%:<%&'!"#$#%:<%&9!"#$#%:<%&&!"#$#%:<%&:!"#$#%:<%:<!"#$#%:<%:(!"#$#%:<%:;!"#$#%:<%:)!"#$#%:<%:8!"#$#%:<%:%!"#$#%:<%:'!"#$#%:<%:9!"#$#%:<%:&!"#$#%:<%::!"#$#%:<'<<!"#$#%:<'<(!"#$#%:<'<;!"#$#%:<'<)!"#$#%:<'<8!"#$#%:<'<%!"#$#%:<'<'!"#$#%:<'<9

!"#$#%:<'<&!"#$#%:<'<:!"#$#%:<'(<!"#$#%:<'((!"#$#%:<'(;!"#$#%:<'()!"#$#%:<'(8!"#$#%:<'(%!"#$#%:<'('!"#$#%:<'(9!"#$#%:<'(&!"#$#%:<'(:!"#$#%:<';<!"#$#%:<';(!"#$#%:<';;!"#$#%:<';)!"#$#%:<';8!"#$#%:<';%!"#$#%:<';'!"#$#%:<';9!"#$#%:<';&!"#$#%:<';:!"#$#%:<')<!"#$#%:<')(

!"#$#%:<');!"#$#%:<'))!"#$#%:<')8!"#$#%:<')%!"#$#%:<')'!"#$#%:<')9!"#$#%:<')&!"#$#%:<'):!"#$#%:<'8<!"#$#%:<'8(!"#$#%:<'8;!"#$#%:<'8)!"#$#%:<'88!"#$#%:<'8%!"#$#%:<'8'!"#$#%:<'89!"#$#%:<'8&!"#$#%:<'8:!"#$#%:<'%<!"#$#%:<'%(!"#$#%:<'%;!"#$#%:<'%)!"#$#%:<'%8!"#$#%:<'%%

!"#$#%:<'%'!"#$#%:<'%9!"#$#%:<'%&!"#$#%:<'%:!"#$#%:<''<!"#$#%:<''(!"#$#%:<'';!"#$#%:<'')!"#$#%:<''8!"#$#%:<''%!"#$#%:<'''!"#$#%:<''9!"#$#%:<''&!"#$#%:<'':!"#$#%:<'9<!"#$#%:<'9(!"#$#%:<'9;!"#$#%:<'9)!"#$#%:<'98!"#$#%:<'9%!"#$#%:<'9'!"#$#%:<'99!"#$#%:<'9&!"#$#%:<'9:

!"#$#%:<'&(!"#$#%:<'&;!"#$#%:<'&)!"#$#%:<'&8!"#$#%:<'&%!"#$#%:<'&'!"#$#%:<'&9!"#$#%:<'&&

|  | $ $ | ->A(: 52- | 7> = 2- - 4 6 / !72 + +2 - $ + ->A(: 0?CDD DA@ |
| --- | --- | --- | --- |
| !"#$#%:<'&: | * + , - - | .* /- | +/ 3" , " 0,"1" 1 /2 2 3 2 " $"4 1 0" 2 +5.2 " /6,27* -+,+ |
| !"#$#%:<':< | ---> | .* /- | " +/ 3" , " 0,"1" 1 /2 2 3 2 " $"4 1 0+"+*/6,27* -+,+ |
| !"#$#%:<':( | 7 > | .* /- | +/ 3" , " 0,"1" 1 /2 2 3 2 " $"4 1 0+" +* " /6,27* -+,+ |
| !"#$#%:<':; | 7 > | .* /- | 3" , " 0,"1" 1 /2 2 3 2 " $"4 1 0+"+ * " +/ /6,27* -+,+ |
| !"#$#%:<':) | 1,$44 4/-- | 1,$44 4/-- | ,/ ** -! , E,! *> ! $ $E>,/ -- |
| !"#$#%:<':8 | 1,$44 4/-- | 1,$44 4/-- | ** ,/ E,-! , ! *> ! $ $E>,/ -- |
| !"#$#%:<':% | 1,$44 4/-- | 1,$44 4/-- | -! , E,** ,/ ! *> ! $ $E>-,/ - |
| !"#$#%:<':' | 1,$44 4/-- | 1,$44 4/-- | -! , ** E,,/ ! *> ! $ $E>-,/ - |
| !"#$#%:<':9 | 1,$44 4/-- | 1,$44 4/-- | ,/ -! , ** E,! *> ! $ $E>-,/ - |
| !"#$#%:<':& | 1,$44 4/-- | 1,$44 4/-- | ** E,-! , ,/ ! *> ! $ $E>,/ -- |

!"#$#%:<'::!"#$#%:<9<<!"#$#%:<9<(!"#$#%:<9<;!"#$#%:<9<)!"#$#%:<9<8!"#$#%:<9<%!"#$#%:<9<'!"#$#%:<9<9!"#$#%:<9<&!"#$#%:<9<:!"#$#%:<9(<!"#$#%:<9((!"#$#%:<9(;!"#$#%:<9()!"#$#%:<9(8!"#$#%:<9(%!"#$#%:<9('!"#$#%:<9(9!"#$#%:<9(&!"#$#%:<9(:!"#$#%:<9;<!"#$#%:<9;(!"#$#%:<9;; !"#$#%:<9;)!"#$#%:<9;8!"#$#%:<9;%!"#$#%:<9;'!"#$#%:<9;9!"#$#%:<9;&!"#$#%:<9;:!"#$#%:<9)<!"#$#%:<9)(!"#$#%:<9);!"#$#%:<9))!"#$#%:<9)8!"#$#%:<9)%!"#$#%:<9)'!"#$#%:<9)9!"#$#%:<9)&!"#$#%:<9):!"#$#%:<98<!"#$#%:<98(!"#$#%:<98;!"#$#%:<98)!"#$#%:<988!"#$#%:<98%!"#$#%:<98'

!"#$#%:<989!"#$#%:<98&!"#$#%:<98:!"#$#%:<9%<!"#$#%:<9%(!"#$#%:<9%;

|  | 5,- , $ | $ $5, , | > |
| --- | --- | --- | --- |

!"#$#%:<9%)!"#$#%:<9%8!"#$#%:<9%%!"#$#%:<9%'!"#$#%:<9%9!"#$#%:<9%&!"#$#%:<9%:!"#$#%:<9'<!"#$#%:<9'(!"#$#%:<9';!"#$#%:<9')!"#$#%:<9'8!"#$#%:<9'%!"#$#%:<9''!"#$#%:<9'9!"#$#%:<9'&!"#$#%:<9':!"#$#%:<99<!"#$#%:<99(!"#$#%:<99;!"#$#%:<99)!"#$#%:<998!"#$#%:<99%!"#$#%:<99' !"#$#%:<999!"#$#%:<99&!"#$#%:<99:!"#$#%:<9&<!"#$#%:<9&(!"#$#%:<9&;!"#$#%:<9&)!"#$#%:<9&8!"#$#%:<9&%!"#$#%:<9&'!"#$#%:<9&9!"#$#%:<9&&!"#$#%:<9&:!"#$#%:<9:<!"#$#%:<9:(!"#$#%:<9:;!"#$#%:<9:)!"#$#%:<9:8!"#$#%:<9:%!"#$#%:<9:'!"#$#%:<9:9!"#$#%:<9:&!"#$#%:<9::!"#$#%:<&<< !"#$#%:<&<(!"#$#%:<&<;!"#$#%:<&<)!"#$#%:<&<8!"#$#%:<&<%!"#$#%:<&<'!"#$#%:<&<9!"#$#%:<&<&!"#$#%:<&<:!"#$#%:<&(<!"#$#%:<&((!"#$#%:<&(;!"#$#%:<&()!"#$#%:<&(8!"#$#%:<&(%!"#$#%:<&('!"#$#%:<&(9!"#$#%:<&(&!"#$#%:<&(:!"#$#%:<&;<!"#$#%:<&;(!"#$#%:<&;;

,7 " 7 $ ,7 " 7 ," + 6$4F

$

!"#$#%:<&;)!"#$#%:<&;8!"#$#%:<&;%!"#$#%:<&;'!"#$#%:<&;9!"#$#%:<&;&!"#$#%:<&;:!"#$#%:<&)<!"#$#%:<&)(!"#$#%:<&);!"#$#%:<&))!"#$#%:<&)8!"#$#%:<&)%!"#$#%:<&)'

|  | , > 53 | , > 53 | , " * >2 4,3 60 , 7 4*. 7 |
| --- | --- | --- | --- |
| !"#$#%:<&)9 | ,7 " 7 $ | ,7 " 7  $ | ," + 6$4F |

!"#$#%:<&):!"#$#%:<&8<!"#$#%:<&8)!"#$#%:<&%(!"#$#%:<&%;!"#$#%:<&%)!"#$#%:<&%8!"#$#%:<&'<!"#$#%:<&'%!"#$#%:<&'9!"#$#%:<&':!"#$#%:<&9(!"#$#%:<&9;!"#$#%:<&98

|  | 0F7 | 0F7 | /0 *+3, /" ,2- / $2 , " 4 | | |
| --- | --- | --- | --- | --- | --- |
| !"#$#%:<&99 | , , 57 7  7E | 1 " 7  > | 2A+2 7 .7 ! ," ,/ /2 7 > 2 * 7 | | |
| !"#$#%:<&9& | 57 , , | 1 " 7  > | 2A+2 7 .7 ! / /2 7 > 2 * 7 | | |
| !"#$#%:<&9:!"#$#%:<&&< | , , 5$, 7 >> 7 0 | 1 " 7  > | 2A+2 7 .7 ! / /2 7 > 2 * 7 | | |
| !"#$#%:<&&( | E , $ | 1 " 7  > | 2A+2 7 .7 ! / /2 7 > 2 * 7 | | |
| !"#$#%:<&&; | 5 $, | 1 " 7  > | 2A+2 7 .7 ! / /2 7 > 2 * 7 | | |
| !"#$#%:<&&)!"#$#%:<&&8 | E7 , | 1 " 7  > | 2A+2 7 .7 ! / /2 7 > 2 * 7 | | |
| !"#$#%:<&&%!"#$#%:<&&'!"#$#%:<&&9  !"#$#%:<&&& | > 7 0, | 1 " 7  > | 2A+2 7 .7 ! / /2 7 > 2 * 7 | | |
| !"#$#%:<&&:!"#$#%:<&:<!"#$#%:<&:(  !"#$#%:<&:; | 57 ,  - | 1 " 7  > | 2A+2 7 .7 ! / /2 7 > 2 * 7 | | |
| !"#$#%:<&:) | , , 5$, 7 >> 7 0 | 1 " 7  > | 2A+2 7 .7 ! / /2 7 > 2 * 7 | | |
| !"#$#%:<&:8!"#$#%:<&:%!"#$#%:<&:'  !"#$#%:<&:9 | 7 0 A2 -$, | 1 " 7  > | 2A+2 7 .7 ! / /2 7 > 2 * 7 | | |
| !"#$#%:<&:& | E , $ | 1 " 7  > | 2A+2 7 .7 ! / /2 7 > 2 * 7 | | |
| !"#$#%:<&:: | E7 , | 1 " 7  > | 2A+2 7 .7 ! / /2 7 > 2 * 7 | | |
| !"#$#%:<:<< | 57 , , | 1 " 7  > | 2A+2 7 .7 ! / /2 7 > 2 * 7 | | |
| !"#$#%:<:<(!"#$#%:<:<;!"#$#%:<:<) | E , $ | 1 " 7  > | 2A+2 7 .7 ! / /2 7 > 2 * 7 | | |
| !"#$#%:<:<8!"#$#%:<:<%!"#$#%:<:<'  !"#$#%:<:<9 | 7 1A2,  , | 1 " 7  > | | 2A+2 7 .7 ! / /2 7 > 2 * 7 |
| !"#$#%:<:<&!"#$#%:<:<:!"#$#%:<:(< | 57 , , | 1 " 7  > | | 2A+2 7 .7 ! / /2 7 > 2 * 7 |
| !"#$#%:<:(( | 5 $, | 1 " 7  > | | 2A+2 7 .7 ! / /2 7 > 2 * 7 |
| !"#$#%:<:(; | E , $ | 1 " 7  > | | 2A+2 7 .7 ! / /2 7 > 2 * 7 |
| !"#$#%:<:()!"#$#%:<:(8 | 57 , , | 1 " 7  > | | 2A+2 7 .7 ! / /2 7 > 2 * 7 |
| !"#$#%:<:(% | 7 1A2,  , | 1 " 7  > | | 2A+2 7 .7 ! / /2 7 > 2 * 7 |
| !"#$#%:<:(' | 57 , , | 1 " 7  > | | 2A+2 7 .7 ! / /2 7 > 2 * 7 |
| !"#$#%:<:(9 | 5 $, | 1 " 7  > | | 2A+2 7 .7 ! / /2 7 > 2 * 7 |
| !"#$#%:<:(& | 57 , , | 1 " 7  > | | 2A+2 7 .7 ! / /2 7 > 2 * 7 |
| !"#$#%:<:(:!"#$#%:<:;< | > 7 0, | 1 " 7  > | | 2A+2 7 .7 ! / /2 7 > 2 * 7 |
| !"#$#%:<:;(!"#$#%:<:;; | 7 0 A2 -$, | 1 " 7  > | | 2A+2 7 .7 ! / /2 7 > 2 * 7 |
| !"#$#%:<:;) | 57 , , | 1 " 7  > | | 2A+2 7 .7 ! / /2 7 > 2 * 7 |
| !"#$#%:<:;8!"#$#%:<:;%!"#$#%:<:;' | 57 11,  - | 1 " 7  > | | 2A+2 7 .7 ! / /2 7 > 2 * 7 |
| !"#$#%:<:;9!"#$#%:<:;& | 57 , , | 1 " 7  > | | 2A+2 7 .7 ! / /2 7 > 2 * 7 |
| !"#$#%:<:;: | , , 5$, 7 >> 7 0 | 1 " 7  > | | 2A+2 7 .7 ! / /2 7 > 2 * 7 |
| !"#$#%:<:)< | 57 , , | 1 " 7  > | | 2A+2 7 .7 ! / /2 7 > 2 * 7 |
| !"#$#%:<:)( | 7 0 A2 -$, | 1 " 7  > | | 2A+2 7 .7 ! / /2 7 > 2 * 7 |
| !"#$#%:<:);!"#$#%:<:)) | , , 5$, 7 >> 7 0 | 1 " 7  > | | 2A+2 7 .7 ! / /2 7 > 2 * 7 |
| !"#$#%:<:)8!"#$#%:<:)% | 57 , , | 1 " 7  > | | 2A+2 7 .7 ! / /2 7 > 2 * 7 |
| !"#$#%:<:)'!"#$#%:<:)9 | 7 1A2,  , | 1 " 7  > | | 2A+2 7 .7 ! / /2 7 > 2 * 7 |
| !"#$#%:<:)&!"#$#%:<:):!"#$#%:<:8< !"#$#%:<:8(!"#$#%:<:8;!"#$#%:<:8) !"#$#%:<:88 | 57 11,  - | 1 " 7  > | | 2A+2 7 .7 ! / /2 7 > 2 * 7 |
| !"#$#%:<:8% | , , 5$, 7 >> 7 0 | 1 " 7  > | | 2A+2 7 .7 ! / /2 7 > 2 * 7 |
| !"#$#%:<:8'!"#$#%:<:89 | 57 11,  - | 1 " 7  > | | 2A+2 7 .7 ! / /2 7 > 2 * 7 |
| !"#$#%:<:8& | 7 0 A2 -$, | 1 " 7  > | | 2A+2 7 .7 ! / /2 7 > 2 * 7 |
| !"#$#%:<:8:!"#$#%:<:%< | 57 11,  - | 1 " 7  > | | 2A+2 7 .7 ! / /2 7 > 2 * 7 |
| !"#$#%:<:%(!"#$#%:<:%; | 57 ,  - | 1 " 7  > | | 2A+2 7 .7 ! / /2 7 > 2 * 7 |

!"#$#%:<:%)!"#$#%:<:%8!"#$#%:<:%%!"#$#%:<:%'!"#$#%:<:%9!"#$#%:<:%&!"#$#%:<:%:!"#$#%:<:'<!"#$#%:<:'(!"#$#%:<:';!"#$#%:<:')!"#$#%:<:'8!"#$#%:<:'%!"#$#%:<:''!"#$#%:<:'9!"#$#%:<:'&!"#$#%:<:':!"#$#%:<:9<!"#$#%:<:9(!"#$#%:<:9;!"#$#%:<:9)!"#$#%:<:98!"#$#%:<:9%

|  | , , 57 7  7E | 1 " 7  > | 2A+$G2 7 .7 ! / /2 7 > 2 * 7 |
| --- | --- | --- | --- |
| !"#$#%:<:9'!"#$#%:<:99 | , , 57 7  7E | 1 " 7  > | 2A+2 7 .7 ! / /2 7 > 2 * 7 |
| !"#$#%:<:9&!"#$#%:<:9:!"#$#%:<:&< | 57 , , | 1 " 7  > | 2A+2 7 .7 ! / /2 7 > 2 * 7 |
| !"#$#%:<:&( | E7 , | 1 " 7  > | 2A+2 7 .7 ! / /2 7 > 2 * 7 |
| !"#$#%:<:&;!"#$#%:<:&) | > 7 0, | 1 " 7  > | 2A+2 7 .7 ! / /2 7 > 2 * 7 |
| !"#$#%:<:&8 | 7 1A2,  , | 1 " 7  > | 2A+2 7 .7 ! / /2 7 > 2 * 7 |
| !"#$#%:<:&% | E7 , | 1 " 7  > | 2A+2 7 .7 ! / /2 7 > 2 * 7 |

!"#$#%:<:&'!"#$#%:<:&9!"#$#%:<:&&!"#$#%:<:&:!"#$#%:<::<!"#$#%:<::(!"#$#%:<::;!"#$#%:<::)!"#$#%:<::8!"#$#%:<::%!"#$#%:<::'!"#$#%:<::9!"#$#%:<::&!"#$#%:<:::!"#$#%:(<<<!"#$#%:(<<(!"#$#%:(<<;!"#$#%:(<<)!"#$#%:(<<8!"#$#%:(<<%!"#$#%:(<<'

|  | | 7 0 A2 -$, | | 1 " 7  > | 2A+2 7 .7 ! / /2 7 > 2 * 7 |
| --- | --- | --- | --- | --- | --- |
| !"#$#%:(<<9 | | , , | | 1 " 7  > | 2A+2 7 .7 ! / /2 7 > 2 * 7 |
| !"#$#%:(<<& | | E7 , | | 1 " 7  > | 2A+2 7 .7 ! / /2 7 > 2 * 7 |
| !"#$#%:(<<: | | 7 0 A2 -$, | | 1 " 7  > | 2A+2 7 .7 ! / /2 7 > 2 * 7 |
| !"#$#%:(<(< | | 5 $, | | 1 " 7  > | 2A+2 7 .7 ! / /2 7 > 2 * 7 |
| !"#$#%:(<(( | | 57 , , | | 1 " 7  > | 2A+2 7 .7 ! / /2 7 > 2 * 7 |
| !"#$#%:(<(; | | , , A, , 7 | | 1 " 7  > | 2A+2 7 .7 ! / /2 7 > 2 * 7 |
| !"#$#%:(<() | | 5 $, | | 1 " 7  > | 2A+2 7 .7 ! / /2 7 > 2 * 7 |
| !"#$#%:(<(8 | | , , | | 1 " 7  > | 2A+2 7 .7 ! / /2 7 > 2 * 7 |
| !"#$#%:(<(%!"#$#%:(<('!"#$#%:(<(9 | | 7 0 A2 -$, | | 1 " 7  > | 2A+2 7 .7 ! / /2 7 > 2 * 7 |
| !"#$#%:(<(&!"#$#%:(<(: | | , , A, , 7 | | 1 " 7 | 2A+2 7 .7 ! / /2 7 > 2 * 7 |
|  | |  | > |  |
| !"#$#%:(<;< | | 57 , , | 1 " 7  > | 2A+2 7 .7 ! / /2 7 > 2 * 7 |
| !"#$#%:(<;( | | , , 57 7  7E | 1 " 7  > | 2A+2 7 .7 ! / /2 7 > 2 * 7 |

!"#$#%:(<;;!"#$#%:(<;)!"#$#%:(<;8!"#$#%:(<;%!"#$#%:(<;'!"#$#%:(<;9!"#$#%:(<;&!"#$#%:(<;:!"#$#%:(<)<!"#$#%:(<)(!"#$#%:(<);!"#$#%:(<))!"#$#%:(<)8!"#$#%:(<)%!"#$#%:(<)'

|  | ,"7$ | ,"7$ | , 7 $ |
| --- | --- | --- | --- |
| !"#$#%:(<&)!"#$#%:(<&8!"#$#%:(<&% !"#$#%:(<&'!"#$#%:(<&9!"#$#%:(<&& !"#$#%:(<&: | > /$1 * /?1/*@ | "B $1 * /?1/*@ | " , A2!1 !2A$ 44E, ,! $ $ +- , -"," +H *  +7 0* / 1 $2, A, +A+B , 0 |
| !"#$#%:(<:(!"#$#%:(<:) | " 5- | ->A(: 52- | $ ,, !I0GG ,7 $ 7 , - 0E 7 = E2 - + 7 $ - + $ - J 6 / !72 + +2 - $+ ->A(: 0 |
| !"#$#%:(<:: | -75" A$> A EII* | -75" A$> A | $ + /1 , -/ 7 *" 0 6" 4+ |

EII*

!"#$#%:((<<!"#$#%:((<(!"#$#%:((<)!"#$#%:((<8!"#$#%:((<%!"#$#%:((<'!"#$#%:((<9!"#$#%:((<&!"#$#%:((<:!"#$#%:(((<!"#$#%:((((!"#$#%:(((;!"#$#%:((()!"#$#%:(((8!"#$#%:(((%!"#$#%:((('!"#$#%:(((9!"#$#%:(((&!"#$#%:(((:!"#$#%:((;(!"#$#%:((;;!"#$#%:((;)!"#$#%:((;8!"#$#%:((;% !"#$#%:((;&!"#$#%:(()<!"#$#%:(()(!"#$#%:(();!"#$#%:(())!"#$#%:(()%!"#$#%:(()9!"#$#%:((8<!"#$#%:((8(!"#$#%:((8;!"#$#%:((88!"#$#%:((8%!"#$#%:((89!"#$#%:((8&!"#$#%:((8:!"#$#%:((%<!"#$#%:((%(!"#$#%:((%;!"#$#%:((%)!"#$#%:((%8!"#$#%:((%%!"#$#%:((%'!"#$#%:((%9!"#$#%:((%&

!"#$#%:((%:!"#$#%:(('<!"#$#%:(('(!"#$#%:((';!"#$#%:((')!"#$#%:(('8!"#$#%:(('%!"#$#%:((''!"#$#%:(('9!"#$#%:(('&!"#$#%:((':!"#$#%:((9<!"#$#%:((9(!"#$#%:((9;!"#$#%:((9)!"#$#%:((98!"#$#%:((9%!"#$#%:((9'!"#$#%:((99!"#$#%:((9&!"#$#%:((9:!"#$#%:((&<!"#$#%:((&(!"#$#%:((&;

!"#$#%:((&)!"#$#%:((&8!"#$#%:((&%!"#$#%:((&'!"#$#%:((&9!"#$#%:((&&!"#$#%:((&:!"#$#%:((:<!"#$#%:((:(!"#$#%:((:;!"#$#%:((:)!"#$#%:((:8!"#$#%:((:%!"#$#%:((:'!"#$#%:((:9!"#$#%:((:&!"#$#%:((::!"#$#%:(;<(!"#$#%:(;<;!"#$#%:(;<)!"#$#%:(;<8!"#$#%:(;<%!"#$#%:(;<9!"#$#%:(;<&

!"#$#%:(;<:!"#$#%:(;(<!"#$#%:(;((!"#$#%:(;(;!"#$#%:(;()!"#$#%:(;(8!"#$#%:(;(%!"#$#%:(;('!"#$#%:(;(9!"#$#%:(;(&!"#$#%:(;(:!"#$#%:(;;<!"#$#%:(;;(!"#$#%:(;;;!"#$#%:(;;)!"#$#%:(;;8!"#$#%:(;;%!"#$#%:(;;'!"#$#%:(;;9!"#$#%:(;;&!"#$#%:(;;:!"#$#%:(;)<!"#$#%:(;)(!"#$#%:(;);

!"#$#%:(;))!"#$#%:(;)8!"#$#%:(;)%!"#$#%:(;)'!"#$#%:(;)9!"#$#%:(;)&!"#$#%:(;):!"#$#%:(;8<!"#$#%:(;8(!"#$#%:(;8;!"#$#%:(;8)!"#$#%:(;88!"#$#%:(;8%!"#$#%:(;8'!"#$#%:(;89!"#$#%:(;8&!"#$#%:(;8:!"#$#%:(;%<!"#$#%:(;%(!"#$#%:(;%;!"#$#%:(;%)!"#$#%:(;%8!"#$#%:(;%%!"#$#%:(;%' !"#$#%:(;%9!"#$#%:(;%&!"#$#%:(;%:!"#$#%:(;'<!"#$#%:(;'(!"#$#%:(;';!"#$#%:(;')!"#$#%:(;'8!"#$#%:(;'%!"#$#%:(;''!"#$#%:(;'9!"#$#%:(;'&!"#$#%:(;':

| 0* 1 ,,5  !"#$#%:(;9<!"#$#%:(;9(!"#$#%:(;9;!"#$#%:(;9)!"#$#%:(;98!"#$#%:(;9%!"#$#%:(;9'!"#$#%:(;99!"#$#%:(;9&!"#$#%:(;9:!"#$#%:(;&< | ," 7 $2 1 .! " + 1, , , |
| --- | --- |
| 1 > - "--- 1 > - | 7 ,3 F3+ 7=37--++=,= 36-/ BE F |

"---

!"#$#%:(;&(!"#$#%:(;&;!"#$#%:(;&8!"#$#%:(;&%!"#$#%:(;&9!"#$#%:(;&&!"#$#%:(;&:!"#$#%:(;:<!"#$#%:(;:(!"#$#%:(;:;!"#$#%:(;:)!"#$#%:(;:8!"#$#%:(;:%!"#$#%:(;:9!"#$#%:(;:&!"#$#%:()<<!"#$#%:()<(!"#$#%:()<;!"#$#%:()<)!"#$#%:()<8!"#$#%:()<%!"#$#%:()<'!"#$#%:()<9!"#$#%:()<&

!"#$#%:()<:!"#$#%:()(<!"#$#%:()(;!"#$#%:()(%

5" 7 $ 5" 7 $ != 2

!"#$#%:();'!"#$#%:();9!"#$#%:();&!"#$#%:();:!"#$#%:())<!"#$#%:())(!"#$#%:());!"#$#%:()))!"#$#%:())8!"#$#%:())%!"#$#%:())9!"#$#%:())&!"#$#%:()8<

* ,5 * , - , "4 - "44 E

5

!"#$#%:()89!"#$#%:()8&!"#$#%:()8:!"#$#%:()%<!"#$#%:()%(!"#$#%:()%;!"#$#%:()%)!"#$#%:()%8!"#$#%:()%%!"#$#%:()%'!"#$#%:()%9!"#$#%:()%&!"#$#%:()%:!"#$#%:()'<!"#$#%:()'(!"#$#%:()';!"#$#%:()')!"#$#%:()'8!"#$#%:()'%!"#$#%:()''!"#$#%:()'9!"#$#%:()'&!"#$#%:()':!"#$#%:()9< !"#$#%:()9(!"#$#%:()9;!"#$#%:()9)!"#$#%:()98!"#$#%:()9%!"#$#%:()9'!"#$#%:()99!"#$#%:()9&!"#$#%:()9:!"#$#%:()&<!"#$#%:()&(!"#$#%:()&;!"#$#%:()&)!"#$#%:()&8!"#$#%:()&%!"#$#%:()&'!"#$#%:()&9!"#$#%:()&&!"#$#%:()&:!"#$#%:():<!"#$#%:():(!"#$#%:():;!"#$#%:():)!"#$#%:():8

!"#$#%:():%!"#$#%:():'!"#$#%:():9!"#$#%:():&!"#$#%:()::!"#$#%:(8<<!"#$#%:(8<(!"#$#%:(8<;!"#$#%:(8<)!"#$#%:(8<8!"#$#%:(8<%!"#$#%:(8<'!"#$#%:(8<9!"#$#%:(8<&!"#$#%:(8<:!"#$#%:(8(<!"#$#%:(8((!"#$#%:(8(;!"#$#%:(8()!"#$#%:(8(8!"#$#%:(8(%!"#$#%:(8('!"#$#%:(8(9!"#$#%:(8(&

!"#$#%:(8(:!"#$#%:(8;<!"#$#%:(8;(!"#$#%:(8;;!"#$#%:(8;)!"#$#%:(8;8!"#$#%:(8;%!"#$#%:(8;'!"#$#%:(8;9!"#$#%:(8;&!"#$#%:(8;:!"#$#%:(8)<!"#$#%:(8)(!"#$#%:(8);!"#$#%:(8))!"#$#%:(8)8!"#$#%:(8)%!"#$#%:(8)'!"#$#%:(8)9!"#$#%:(8)&!"#$#%:(8):!"#$#%:(88<!"#$#%:(88(!"#$#%:(88;

!"#$#%:(88)!"#$#%:(888!"#$#%:(88%!"#$#%:(88'!"#$#%:(889!"#$#%:(88&!"#$#%:(88:!"#$#%:(8%<!"#$#%:(8%(!"#$#%:(8%;!"#$#%:(8%)!"#$#%:(8%8!"#$#%:(8%%!"#$#%:(8%'!"#$#%:(8%9!"#$#%:(8%&!"#$#%:(8%:!"#$#%:(8'<!"#$#%:(8'(!"#$#%:(8';!"#$#%:(8')!"#$#%:(8'8!"#$#%:(8'%!"#$#%:(8'' !"#$#%:(8'9!"#$#%:(8'&!"#$#%:(8':!"#$#%:(89<

| "-1 "-1    !"#$#%:(89(!"#$#%:(89;!"#$#%:(89)!"#$#%:(898!"#$#%:(89%!"#$#%:(89'!"#$#%:(899!"#$#%:(89&!"#$#%:(89:!"#$#%:(8&<!"#$#%:(8&(!"#$#%:(8&;!"#$#%:(8&) | | | 0 4 2 /0 ,7, 2 |
| --- | --- | --- | --- |
|  | "-1 | "-1 | 0 4 2 /0 ,77.20, 2 |
| !"#$#%:(8&8 | - $ | 17 " A -  " , /J 7 J5 | -,A"7 |
| !"#$#%:(8&%!"#$#%:(8&' | 7 | 17 " A -  " , /J 7 J5 | -,A"7 |
| !"#$#%:(8&9!"#$#%:(8&&!"#$#%:(8&: !"#$#%:(8:<!"#$#%:(8:( | $" | 17 " A -  " , /J 7 J5 | -,A"7 |
| !"#$#%:(8:; | , " | 17 " A -  " , /J 7 J5 | -,A"7 |
| !"#$#%:(8:) | " 1A/ 17 A17 " | 17 " A -  " , /J 7 J5 | -,A"7 |
| !"#$#%:(8:8!"#$#%:(8:%!"#$#%:(8:'  !"#$#%:(8:9!"#$#%:(8:&!"#$#%:(8:: !"#$#%:(%<<!"#$#%:(%<( | " A17 " | 17 " A -  " , /J 7 J5 | -,A"7 |
| !"#$#%:(%<;!"#$#%:(%<)!"#$#%:(%<8 | !$?!$@ | 17 " A -  " , /J 7 J5 | -,A"7 |
| !"#$#%:(%<%!"#$#%:(%<' | >K" ?"@ | 17 " A -  " , /J 7 J5 | -,A"7 |
| !"#$#%:(%<9 | " ?"@A-/  7 A17 " | 17 " A -  " , /J 7 J5 | -,A"7 |
| !"#$#%:(%<&!"#$#%:(%<:!"#$#%:(%(<  !"#$#%:(%((!"#$#%:(%(;!"#$#%:(%()  !"#$#%:(%(8!"#$#%:(%(%!"#$#%:(%(' | " ?"@A$ 7 A  17 " | 17 " A -  " , /J 7 J5 | -,A"7 |
| !"#$#%:(%(9 | " ?"@A/ "  7 A17 " | 17 " A -  " , /J 7 J5 | -,A"7 |
| !"#$#%:(%(& | 0- K7 | 17 " A -  " , /J 7 J5 | -,A"7 |
| !"#$#%:(%(:!"#$#%:(%;<!"#$#%:(%;( | "> LE ,5- | -, , - / - | > E0" /5->, ,$,4M 4,E, |
| !"#$#%:(%;;!"#$#%:(%;)!"#$#%:(%;8  !"#$#%:(%;%!"#$#%:(%;'!"#$#%:(%;9  !"#$#%:(%;&!"#$#%:(%;:!"#$#%:(%)< | ,15- A,$ | -, , - / - | > E0" /5->, ,$,4M 4,E, |
| !"#$#%:(%)(!"#$#%:(%);!"#$#%:(%))  !"#$#%:(%)8 | $ | -, , - / - | > E0" /5->, ,$,4M 4,E, |
| !"#$#%:(%)%!"#$#%:(%)'!"#$#%:(%)9 !"#$#%:(%)&!"#$#%:(%): | "-1 | "-1 | 0 4 2 /0 ,7, 2 |
| !"#$#%:(%8(!"#$#%:(%8;!"#$#%:(%8) !"#$#%:(%88!"#$#%:(%8%!"#$#%:(%8' !"#$#%:(%89 | -75" A$> A EII* | -75" A$> A  EII* | $ + /1 , -/ 7 *" 0 6" 4+ |
| !"#$#%:(%%<!"#$#%:(%%(!"#$#%:(%%;  !"#$#%:(%%)!"#$#%:(%%8!"#$#%:(%%% !"#$#%:(%%'!"#$#%:(%%9 | , 5A" 7 $?,5A"7$@ | ,5A"7$ | 0 4, * , 1 |

| !"#$#%:(%%& | | > /$?>/$@ | >/$,5A"7$ | - $ 0 , 4, * + 1 |
| --- | --- | --- | --- | --- |
| !"#$#%:(%%:!"#$#%:(%'< | | , 5A" 7 $?,5A"7$@ | ,5A"7$ | 0 4, * , 1 |
| !"#$#%:(%'(!"#$#%:(%'; | | > /$?>/$@ | >/$,5A"7$ | - $ 0 , 4, * + 1 |
| !"#$#%:(%')!"#$#%:(%'8 | | , 5A" 7 $?,5A"7$@ | ,5A"7$ | 0 4, * , 1 |
| !"#$#%:(%'% | | > /$?>/$@ | >/$,5A"7$ | - $ 0 , 4, * + 1 |
| !"#$#%:(%''!"#$#%:(%'9!"#$#%:(%'& !"#$#%:(%':!"#$#%:(%9< | | , 5A" 7 $?,5A"7$@ | ,5A"7$ | 0 4, * , 1 |
| !"#$#%:(%9( | | > /$?>/$@ | >/$,5A"7$ | - $ 0 , 4, * + 1 |
| !"#$#%:(%9; | | , 5A" 7 $?,5A"7$@ | ,5A"7$ | 0 4, * , 1 |
| !"#$#%:(%9)!"#$#%:(%98 | | > /$?>/$@ | >/$,5A"7$ | - $ 0 , 4, * + 1 |
| !"#$#%:(%9%!"#$#%:(%9'!"#$#%:(%99 | | , 5A" 7 $?,5A"7$@ | ,5A"7$ | 0 4, * , 1 |
| !"#$#%:(%9& | | > /$?>/$@ | >/$,5A"7$ | - $ 0 , 4, * + 1 |
| !"#$#%:(%9: | | , 5A" 7 $?,5A"7$@ | ,5A"7$ | 0 4, * , 1 |
| !"#$#%:(%&< | | > /$?>/$@ | >/$,5A"7$ | - $ 0 , 4, * + 1 |
| !"#$#%:(%&(!"#$#%:(%&;!"#$#%:(%&) | | , 5A" 7 $?,5A"7$@ | ,5A"7$ | 0 4, * , 1 |
| !"#$#%:(%&8!"#$#%:(%&% | | > /$?>/$@ | >/$,5A"7$ | - $ 0 , 4, * + 1 |
| !"#$#%:(%&' | | , 5A" 7 $?,5A"7$@ | ,5A"7$ | 0 4, * , 1 |
| !"#$#%:(%&9 | | > /$?>/$@ | >/$,5A"7$ | - $ 0 , 4, * + 1 |
| !"#$#%:(%&&!"#$#%:(%&: | | , 5A" 7 $?,5A"7$@ | ,5A"7$ | 0 4, * , 1 |
| !"#$#%:(%:<!"#$#%:(%:(!"#$#%:(%:; | | > /$?>/$@ | >/$,5A"7$ | - $ 0 , 4, * + 1 |
| !"#$#%:(%:)!"#$#%:(%:8 | | , 5A" 7 $?,5A"7$@ | ,5A"7$ | 0 4, * , 1 |
| !"#$#%:(%:% | | > /$?>/$@ | >/$,5A"7$ | - $ 0 , 4, * + 1 |
| !"#$#%:(%:'!"#$#%:(%:9!"#$#%:(%:& !"#$#%:(%::!"#$#%:('<<!"#$#%:('<( !"#$#%:('<; | | , 5A" 7 $?,5A"7$@ | ,5A"7$ | 0 4, * , 1 |
| !"#$#%:('<) | | > /$?>/$@ | >/$,5A"7$ | - $ 0 , 4, * + 1 |
| !"#$#%:('<8!"#$#%:('<%!"#$#%:('<' !"#$#%:('<9!"#$#%:('<& | | , 5A" 7 $?,5A"7$@ | ,5A"7$ | 0 4, * , 1 |
| !"#$#%:('<: | | > /$?>/$@ | >/$,5A"7$ | - $ 0 , 4, * + 1 |
| !"#$#%:('(<!"#$#%:('((!"#$#%:('(; | | , 5A" 7 $?,5A"7$@ | ,5A"7$ | 0 4, * , 1 |
| !"#$#%:('() | | > /$?>/$@ | >/$,5A"7$ | - $ 0 , 4, * + 1 |
| !"#$#%:('(8 | | , 5A" 7 $?,5A"7$@ | ,5A"7$ | 0 4, * , 1 |
| !"#$#%:('(% | | > /$?>/$@ | >/$,5A"7$ | - $ 0 , 4, * + 1 |
| !"#$#%:('('!"#$#%:('(9!"#$#%:('(& | | , 5A" 7 $?,5A"7$@ | ,5A"7$ | 0 4, * , 1 |
| !"#$#%:('(: | | > /$?>/$@ | >/$,5A"7$ | - $ 0 , 4, * + 1 |
| !"#$#%:(';< | | , 5A" 7 $?,5A"7$@ | ,5A"7$ | 0 4, * , 1 |
| !"#$#%:(';( | | > /$?>/$@ | >/$,5A"7$ | - $ 0 , 4, * + 1 |
| !"#$#%:(';; | | , 5A" 7 $?,5A"7$@ | ,5A"7$ | 0 4, * , 1 |
| !"#$#%:(';) | | > /$?>/$@ | >/$,5A"7$ | - $ 0 , 4, * + 1 |
| !"#$#%:(';8!"#$#%:(';% | | , 5A" 7 $?,5A"7$@ | ,5A"7$ | 0 4, * , 1 |
| !"#$#%:(';' | | > /$?>/$@ | >/$,5A"7$ | - $ 0 , 4, * + 1 |
| !"#$#%:(';9!"#$#%:(';&!"#$#%:(';:!"#$#%:(')<!"#$#%:(')(!"#$#%:(');!"#$#%:('))!"#$#%:(')8!"#$#%:(')%!"#$#%:(')'!"#$#%:(')9 | | | |
| , 5A" 7 $?,5A"7$@ ,5A"7$ | | | | 0 4, * , 1 |
| !"#$#%:(')& > /$?>/$@ >/$,5A"7$ | | | | - $ 0 , 4, * + 1 |
| !"#$#%:('): , 5A" 7 $?,5A"7$@ ,5A"7$ | | | | 0 4, * , 1 |
| !"#$#%:('8< > /$?>/$@ >/$,5A"7$ | | | | - $ 0 , 4, * + 1 |
| !"#$#%:('8(!"#$#%:('8; , 5A" 7 $?,5A"7$@ ,5A"7$ | | | | 0 4, * , 1 |
| !"#$#%:('8) > /$?>/$@ >/$,5A"7$ | | | | - $ 0 , 4, * + 1 |
| !"#$#%:('88 , 5A" 7 $?,5A"7$@ ,5A"7$ | | | | 0 4, * , 1 |
| !"#$#%:('8% > /$?>/$@ >/$,5A"7$ | | | | - $ 0 , 4, * + 1 |
| !"#$#%:('8'!"#$#%:('89!"#$#%:('8& , 5A" 7 $?,5A"7$@ ,5A"7$  !"#$#%:('8:!"#$#%:('%<!"#$#%:('%( | | | | 0 4, * , 1 |
| !"#$#%:('%;!"#$#%:('%)!"#$#%:('%8 > /$?>/$@ >/$,5A"7$ | | | | - $ 0 , 4, * + 1 |
| !"#$#%:('%% , 5A" 7 $?,5A"7$@ ,5A"7$ | | | | 0 4, * , 1 |
| !"#$#%:('%'!"#$#%:('%9 > /$?>/$@ >/$,5A"7$ | | | | - $ 0 , 4, * + 1 |
| !"#$#%:('%& , 5A" 7 $?,5A"7$@ ,5A"7$ | | | | 0 4, * , 1 |
| !"#$#%:('%: > /$?>/$@ >/$,5A"7$ | | | | - $ 0 , 4, * + 1 |
| !"#$#%:(''<!"#$#%:(''(!"#$#%:(''; , 5A" 7 $?,5A"7$@ ,5A"7$  !"#$#%:('')!"#$#%:(''8!"#$#%:(''% | | | | 0 4, * , 1 |
| !"#$#%:('''!"#$#%:(''9 > /$?>/$@ >/$,5A"7$ | | | | - $ 0 , 4, * + 1 |
| !"#$#%:(''& , 5A" 7 $?,5A"7$@ ,5A"7$ | | | | 0 4, * , 1 |
| !"#$#%:('':!"#$#%:('9< > /$?>/$@ >/$,5A"7$ | | | | - $ 0 , 4, * + 1 |
| !"#$#%:('9( , 5A" 7 $?,5A"7$@ ,5A"7$ | | | | 0 4, * , 1 |
| !"#$#%:('9; > /$?>/$@ >/$,5A"7$ | | | | - $ 0 , 4, * + 1 |
| !"#$#%:('9)!"#$#%:('98!"#$#%:('9% , 5A" 7 $?,5A"7$@ ,5A"7$  !"#$#%:('9'!"#$#%:('99!"#$#%:('9& | | | | 0 4, * , 1 |
| !"#$#%:('9: > /$?>/$@ >/$,5A"7$ | | | | - $ 0 , 4, * + 1 |
| !"#$#%:('&< , 5A" 7 $?,5A"7$@ ,5A"7$ | | | | 0 4, * , 1 |
| !"#$#%:('&( > /$?>/$@ >/$,5A"7$ | | | | - $ 0 , 4, * + 1 |
| !"#$#%:('&;!"#$#%:('&) , 5A" 7 $?,5A"7$@ ,5A"7$ | | | | 0 4, * , 1 |
| !"#$#%:('&8 > /$?>/$@ >/$,5A"7$ | | | | - $ 0 , 4, * + 1 |
| !"#$#%:('&%!"#$#%:('&'!"#$#%:('&9 , 5A" 7 $?,5A"7$@ ,5A"7$ | | | | 0 4, * , 1 |
| !"#$#%:('&& > /$?>/$@ >/$,5A"7$ | | | | - $ 0 , 4, * + 1 |
| !"#$#%:('&:!"#$#%:(':<!"#$#%:(':( , 5A" 7 $?,5A"7$@ ,5A"7$ !"#$#%:(':;!"#$#%:(':)!"#$#%:(':8  !"#$#%:(':%!"#$#%:(':'!"#$#%:(':9 | | | | 0 4, * , 1 |
| !"#$#%:(':& > /$?>/$@ >/$,5A"7$ | | | | - $ 0 , 4, * + 1 |
| !"#$#%:(':: , 5A" 7 $?,5A"7$@ ,5A"7$ | | | | 0 4, * , 1 |
| !"#$#%:(9<< > /$?>/$@ >/$,5A"7$ | | | | - $ 0 , 4, * + 1 |
| !"#$#%:(9<(!"#$#%:(9<; | , 5A" 7 $?,5A"7$@ | ,5A"7$ | 0 4, * , 1 |
| !"#$#%:(9<)!"#$#%:(9<8 | > /$?>/$@ | >/$,5A"7$ | - $ 0 , 4, * + 1 |
| !"#$#%:(9<% | , 5A" 7 $?,5A"7$@ | ,5A"7$ | 0 4, * , 1 |
| !"#$#%:(9<'!"#$#%:(9<9!"#$#%:(9<& | > /$?>/$@ | >/$,5A"7$ | - $ 0 , 4, * + 1 |
| !"#$#%:(9<:!"#$#%:(9(<!"#$#%:(9(( !"#$#%:(9(;!"#$#%:(9()!"#$#%:(9(8 | , 5A" 7 $?,5A"7$@ | ,5A"7$ | 0 4, * , 1 |
| !"#$#%:(9(% | > /$?>/$@ | >/$,5A"7$ | - $ 0 , 4, * + 1 |
| !"#$#%:(9('!"#$#%:(9(9!"#$#%:(9(& | , 5A" 7 $?,5A"7$@ | ,5A"7$ | 0 4, * , 1 |
| !"#$#%:(9:(!"#$#%:(9:; | > /$?>/$@ | >/$,5A"7$ | - $ 0 , 4, * + 1 |
| !"#$#%:(9:)!"#$#%:(9:8!"#$#%:(9:% !"#$#%:(9:'!"#$#%:(9:9!"#$#%:(9:& | , 5A" 7 $?,5A"7$@ | ,5A"7$ | 0 4, * , 1 |
| !"#$#%:(9::!"#$#%:(&<< | > /$?>/$@ | >/$,5A"7$ | - $ 0 , 4, * + 1 |
| !"#$#%:(&<(!"#$#%:(&<;!"#$#%:(&<) !"#$#%:(&<8!"#$#%:(&<%!"#$#%:(&<' | , 5A" 7 $?,5A"7$@ | ,5A"7$ | 0 4, * , 1 |
| !"#$#%:(&<9 | > /$?>/$@ | >/$,5A"7$ | - $ 0 , 4, * + 1 |

!"#$#%:(&<&!"#$#%:(&<:!"#$#%:(&(<!"#$#%:(&((!"#$#%:(&(;!"#$#%:(&()!"#$#%:(&(8!"#$#%:(&(%!"#$#%:(&('!"#$#%:(&(9!"#$#%:(&(&!"#$#%:(&(:!"#$#%:(&;<!"#$#%:(&;(

|  | , 5A" 7 $?,5A"7$@ | ,5A"7$ | 0 4, * , 1 |
| --- | --- | --- | --- |
| !"#$#%:(&;; | > /$?>/$@ | >/$,5A"7$ | - $ 0 , 4, * + 1 |
| !"#$#%:(&;) | , 5A" 7 $?,5A"7$@ | ,5A"7$ | 0 4, * , 1 |
| !"#$#%:(&;8!"#$#%:(&;% | > /$?>/$@ | >/$,5A"7$ | - $ 0 , 4, * + 1 |

!"#$#%:(&;'!"#$#%:(&;9!"#$#%:(&;&!"#$#%:(&;:!"#$#%:(&)<!"#$#%:(&)(!"#$#%:(&);!"#$#%:(&))!"#$#%:(&)8!"#$#%:(&)%!"#$#%:(&)'!"#$#%:(&)9!"#$#%:(&)&!"#$#%:(&):!"#$#%:(&8<!"#$#%:(&8(!"#$#%:(&8;!"#$#%:(&8)!"#$#%:(&88!"#$#%:(&8%!"#$#%:(&8'!"#$#%:(&89!"#$#%:(&8&

|  | , 5A" 7 $?,5A"7$@ | ,5A"7$ | 0 4, * , 1 |
| --- | --- | --- | --- |
| !"#$#%:(&8:!"#$#%:(&%<!"#$#%:(&%(  !"#$#%:(&%; | > /$?>/$@ | >/$,5A"7$ | - $ 0 , 4, * + 1 |
| !"#$#%:(&%) | , 5A" 7 $?,5A"7$@ | ,5A"7$ | 0 4, * , 1 |

!"#$#%:(&%8!"#$#%:(&%%!"#$#%:(&%'!"#$#%:(&%9!"#$#%:(&%&!"#$#%:(&%:!"#$#%:(&'<!"#$#%:(&'(!"#$#%:(&';!"#$#%:(&')!"#$#%:(&'8!"#$#%:(&'%!"#$#%:(&''!"#$#%:(&'9!"#$#%:(&'&!"#$#%:(&':!"#$#%:(&9<!"#$#%:(&9(!"#$#%:(&9;!"#$#%:(&9)!"#$#%:(&98!"#$#%:(&9%!"#$#%:(&9'!"#$#%:(&99 !"#$#%:(&9&!"#$#%:(&9:!"#$#%:(&&<!"#$#%:(&&(!"#$#%:(&&;!"#$#%:(&&)!"#$#%:(&&8!"#$#%:(&&%!"#$#%:(&&'!"#$#%:(&&9!"#$#%:(&&&!"#$#%:(&&:!"#$#%:(&:<!"#$#%:(&:(!"#$#%:(&:;!"#$#%:(&:)!"#$#%:(&:8!"#$#%:(&:%!"#$#%:(&:'!"#$#%:(&:9!"#$#%:(&:&!"#$#%:(&::!"#$#%:(:<<!"#$#%:(:<( !"#$#%:(:<;!"#$#%:(:<)!"#$#%:(:<8

|  | > /$?>/$@ | >/$,5A"7$ | - $ 0 , 4, * + 1 |
| --- | --- | --- | --- |
| !"#$#%:(:<%!"#$#%:(:<' | , 5A" 7 $?,5A"7$@ | ,5A"7$ | 0 4, * , 1 |
| !"#$#%:(:<9!"#$#%:(:<&!"#$#%:(:<: | > /$?>/$@ | >/$,5A"7$ | - $ 0 , 4, * + 1 |
| !"#$#%:(:(<!"#$#%:(:(( | , 5A" 7 $?,5A"7$@ | ,5A"7$ | 0 4, * , 1 |
| !"#$#%:(:(; | > /$?>/$@ | >/$,5A"7$ | - $ 0 , 4, * + 1 |
| !"#$#%:(:()!"#$#%:(:(8!"#$#%:(:(%  !"#$#%:(:(' | , 5A" 7 $?,5A"7$@ | ,5A"7$ | 0 4, * , 1 |
| !"#$#%:(:(9 | > /$?>/$@ | >/$,5A"7$ | - $ 0 , 4, * + 1 |
| !"#$#%:(:(& | , 5A" 7 $?,5A"7$@ | ,5A"7$ | 0 4, * , 1 |
| !"#$#%:(:(: | > /$?>/$@ | >/$,5A"7$ | - $ 0 , 4, * + 1 |
| !"#$#%:(:;<!"#$#%:(:;(!"#$#%:(:;; !"#$#%:(:;)!"#$#%:(:;8 | , 5A" 7 $?,5A"7$@ | ,5A"7$ | 0 4, * , 1 |
| !"#$#%:(:;% | > /$?>/$@ | >/$,5A"7$ | - $ 0 , 4, * + 1 |
| !"#$#%:(:;'!"#$#%:(:;9 | , 5A" 7 $?,5A"7$@ | ,5A"7$ | 0 4, * , 1 |
| !"#$#%:(:;& | > /$?>/$@ | >/$,5A"7$ | - $ 0 , 4, * + 1 |
| !"#$#%:(:;:!"#$#%:(:)<!"#$#%:(:)( | , 5A" 7 $?,5A"7$@ | ,5A"7$ | 0 4, * , 1 |
| !"#$#%:(:); | > /$?>/$@ | >/$,5A"7$ | - $ 0 , 4, * + 1 |
| !"#$#%:(:))!"#$#%:(:)8!"#$#%:(:)% | , 5A" 7 $?,5A"7$@ | ,5A"7$ | 0 4, * , 1 |

!"#$#%:(:)'!"#$#%:(:)9!"#$#%:(:)& !"#$#%:(:):

!"#$#%:(:8<!"#$#%:(:8(!"#$#%:(:8;!"#$#%:(:8)!"#$#%:(:88!"#$#%:(:8%!"#$#%:(:8'!"#$#%:(:89!"#$#%:(:8&!"#$#%:(:8:!"#$#%:(:%<!"#$#%:(:%(!"#$#%:(:%;!"#$#%:(:%)!"#$#%:(:%8!"#$#%:(:%%!"#$#%:(:%'!"#$#%:(:%9!"#$#%:(:%&!"#$#%:(:%:!"#$#%:(:'<

> /$?>/$@ >/$,5A"7$ - $ 0 , 4, * + 1

!"#$#%:(:'(!"#$#%:(:';!"#$#%:(:')!"#$#%:(:'8!"#$#%:(:'%!"#$#%:(:''!"#$#%:(:'9!"#$#%:(:':!"#$#%:(:9<!"#$#%:(:9(!"#$#%:(:9;!"#$#%:(:9)!"#$#%:(:98!"#$#%:(:9%!"#$#%:(:9'!"#$#%:(:99!"#$#%:(:9&!"#$#%:(:9:!"#$#%:(:&<!"#$#%:(:&(!"#$#%:(:&;!"#$#%:(:&)!"#$#%:(:&8!"#$#%:(:&% !"#$#%:(:&'!"#$#%:(:&9!"#$#%:(:&&!"#$#%:(:&:!"#$#%:(::<!"#$#%:(::(!"#$#%:(::;!"#$#%:(::)!"#$#%:(::8!"#$#%:(::%!"#$#%:(::'!"#$#%:(::9!"#$#%:(::&!"#$#%:(:::!"#$#%:;<<<!"#$#%:;<<(!"#$#%:;<<;!"#$#%:;<<)!"#$#%:;<<8!"#$#%:;<<%!"#$#%:;<<'!"#$#%:;<<9!"#$#%:;<<&!"#$#%:;<<:

!"#$#%:;<(<!"#$#%:;<((!"#$#%:;<(;!"#$#%:;<()!"#$#%:;<(8!"#$#%:;<(%!"#$#%:;<('!"#$#%:;<(9!"#$#%:;<(&!"#$#%:;<(:!"#$#%:;<;<!"#$#%:;<;(!"#$#%:;<;;!"#$#%:;<;)!"#$#%:;<;8!"#$#%:;<;%!"#$#%:;<;'!"#$#%:;<;9!"#$#%:;<;&!"#$#%:;<;:!"#$#%:;<)<!"#$#%:;<)(!"#$#%:;<);!"#$#%:;<))

!"#$#%:;<)8!"#$#%:;<)%!"#$#%:;<)'!"#$#%:;<)9!"#$#%:;<)&!"#$#%:;<):!"#$#%:;<8<!"#$#%:;<8(!"#$#%:;<8;!"#$#%:;<8)!"#$#%:;<88!"#$#%:;<8%!"#$#%:;<8'!"#$#%:;<89!"#$#%:;<8&!"#$#%:;<8:!"#$#%:;<%<!"#$#%:;<%(!"#$#%:;<%;!"#$#%:;<%)!"#$#%:;<%8!"#$#%:;<%%!"#$#%:;<%'!"#$#%:;<%9

!"#$#%:;<%&!"#$#%:;<%:!"#$#%:;<'<!"#$#%:;<'(!"#$#%:;<';!"#$#%:;<')!"#$#%:;<'8!"#$#%:;<'%!"#$#%:;<''!"#$#%:;<'9!"#$#%:;<'&!"#$#%:;<':!"#$#%:;<9<!"#$#%:;<9(!"#$#%:;<9;!"#$#%:;<9)!"#$#%:;<98!"#$#%:;<9%!"#$#%:;<9'!"#$#%:;<99!"#$#%:;<9&!"#$#%:;<9:!"#$#%:;<&<!"#$#%:;<&(

!"#$#%:;<&;!"#$#%:;<&)!"#$#%:;<&8!"#$#%:;<&%!"#$#%:;<&'!"#$#%:;<&9!"#$#%:;<&&!"#$#%:;<&:!"#$#%:;<:<!"#$#%:;<:(!"#$#%:;<:;!"#$#%:;<:)!"#$#%:;<:8!"#$#%:;<:%!"#$#%:;<:'!"#$#%:;<:9!"#$#%:;<:&!"#$#%:;<::!"#$#%:;(<<!"#$#%:;(<(!"#$#%:;(<;!"#$#%:;(<)!"#$#%:;(<8!"#$#%:;(<%

!"#$#%:;(<'!"#$#%:;(<9!"#$#%:;(<&!"#$#%:;(<:!"#$#%:;((<!"#$#%:;(((!"#$#%:;((;!"#$#%:;(()!"#$#%:;((8!"#$#%:;((%!"#$#%:;(('!"#$#%:;((9!"#$#%:;((&!"#$#%:;((:!"#$#%:;(;<!"#$#%:;(;(!"#$#%:;(;;!"#$#%:;(;)!"#$#%:;(;8!"#$#%:;(;%!"#$#%:;(;'!"#$#%:;(;9!"#$#%:;(;&!"#$#%:;(;:

!"#$#%:;()<!"#$#%:;()(!"#$#%:;();!"#$#%:;())!"#$#%:;()8!"#$#%:;()%

, 5A" 7 $?,5A"7$@ ,5A"7$ 0 4, * , 1

!"#$#%:;()'!"#$#%:;()9!"#$#%:;()&!"#$#%:;():!"#$#%:;(8<!"#$#%:;(8(!"#$#%:;(8;!"#$#%:;(8)!"#$#%:;(88!"#$#%:;(8%!"#$#%:;(8'!"#$#%:;(89!"#$#%:;(8&!"#$#%:;(8:!"#$#%:;(%<!"#$#%:;(%(!"#$#%:;(%;!"#$#%:;(%)!"#$#%:;(%8!"#$#%:;(%%!"#$#%:;(%'!"#$#%:;(%9!"#$#%:;(%&!"#$#%:;(%: !"#$#%:;('<!"#$#%:;('(!"#$#%:;(';!"#$#%:;(')!"#$#%:;('8!"#$#%:;('%!"#$#%:;(''!"#$#%:;('9!"#$#%:;('&!"#$#%:;(':!"#$#%:;(9<!"#$#%:;(9(!"#$#%:;(9;!"#$#%:;(9)!"#$#%:;(98!"#$#%:;(9%!"#$#%:;(9'!"#$#%:;(99!"#$#%:;(9&!"#$#%:;(9:!"#$#%:;(&<!"#$#%:;(&(!"#$#%:;(&;!"#$#%:;(&)

!"#$#%:;(&8!"#$#%:;(&%!"#$#%:;(&'!"#$#%:;(&9!"#$#%:;(&&!"#$#%:;(&:!"#$#%:;(:<!"#$#%:;(:(!"#$#%:;(:;!"#$#%:;(:)!"#$#%:;(:8!"#$#%:;(:%

> /$?>/$@ >/$,5A"7$ - $ 0 , 4, * + 1

!"#$#%:;(:'!"#$#%:;(:9!"#$#%:;(:&!"#$#%:;(::!"#$#%:;;<<!"#$#%:;;<(!"#$#%:;;<;!"#$#%:;;<)!"#$#%:;;<8!"#$#%:;;<%!"#$#%:;;<'!"#$#%:;;<9!"#$#%:;;<&!"#$#%:;;<:!"#$#%:;;(<!"#$#%:;;((!"#$#%:;;(;!"#$#%:;;()!"#$#%:;;(8!"#$#%:;;(%!"#$#%:;;('!"#$#%:;;(9!"#$#%:;;(&!"#$#%:;;(: !"#$#%:;;;<!"#$#%:;;;(!"#$#%:;;;;!"#$#%:;;;)!"#$#%:;;;8!"#$#%:;;;%!"#$#%:;;;'!"#$#%:;;;9!"#$#%:;;;&!"#$#%:;;;:!"#$#%:;;)<!"#$#%:;;)(!"#$#%:;;);!"#$#%:;;))!"#$#%:;;)8!"#$#%:;;)%!"#$#%:;;)'!"#$#%:;;)9!"#$#%:;;)&!"#$#%:;;):!"#$#%:;;8<!"#$#%:;;8(!"#$#%:;;8;!"#$#%:;;8)

!"#$#%:;;88!"#$#%:;;8%!"#$#%:;;8'!"#$#%:;;89!"#$#%:;;8&!"#$#%:;;8:!"#$#%:;;%<!"#$#%:;;%(!"#$#%:;;%;!"#$#%:;;%)!"#$#%:;;%8!"#$#%:;;%%!"#$#%:;;%'!"#$#%:;;%9!"#$#%:;;%&!"#$#%:;;%:!"#$#%:;;'<!"#$#%:;;'(!"#$#%:;;';!"#$#%:;;')!"#$#%:;;'8!"#$#%:;;'%!"#$#%:;;''!"#$#%:;;'9

!"#$#%:;;'&!"#$#%:;;':!"#$#%:;;9<!"#$#%:;;9(!"#$#%:;;9;!"#$#%:;;9)!"#$#%:;;98!"#$#%:;;9%!"#$#%:;;9'!"#$#%:;;99!"#$#%:;;9&!"#$#%:;;9:!"#$#%:;;&<!"#$#%:;;&(!"#$#%:;;&;!"#$#%:;;&)!"#$#%:;;&8!"#$#%:;;&%!"#$#%:;;&'!"#$#%:;;&9!"#$#%:;;&&!"#$#%:;;&:!"#$#%:;;:<!"#$#%:;;:(

!"#$#%:;;:;!"#$#%:;;:)!"#$#%:;;:8!"#$#%:;;:%!"#$#%:;;:'!"#$#%:;;:9!"#$#%:;;:&!"#$#%:;;::!"#$#%:;)<<!"#$#%:;)<(!"#$#%:;)<;!"#$#%:;)<)!"#$#%:;)<8!"#$#%:;)<%!"#$#%:;)<'!"#$#%:;)<9!"#$#%:;)<&!"#$#%:;)<:!"#$#%:;)(<!"#$#%:;)((!"#$#%:;)(;!"#$#%:;)()!"#$#%:;)(8!"#$#%:;)(% !"#$#%:;)('!"#$#%:;)(9!"#$#%:;)(&!"#$#%:;)(:!"#$#%:;);<!"#$#%:;);(!"#$#%:;);;!"#$#%:;);)!"#$#%:;);8!"#$#%:;);%!"#$#%:;);'!"#$#%:;);9!"#$#%:;);&!"#$#%:;);:!"#$#%:;))<!"#$#%:;))(!"#$#%:;));!"#$#%:;)))!"#$#%:;))8!"#$#%:;))%!"#$#%:;))'!"#$#%:;))9!"#$#%:;))&!"#$#%:;)):

!"#$#%:;)8<!"#$#%:;)8(!"#$#%:;)8;!"#$#%:;)8)!"#$#%:;)88!"#$#%:;)8%!"#$#%:;)8'!"#$#%:;)89!"#$#%:;)8&!"#$#%:;)8:!"#$#%:;)%<!"#$#%:;)%(!"#$#%:;)%;!"#$#%:;)%)!"#$#%:;)%8!"#$#%:;)%%!"#$#%:;)%'!"#$#%:;)%9!"#$#%:;)%&!"#$#%:;)%:!"#$#%:;)'<!"#$#%:;)'(!"#$#%:;)';!"#$#%:;)')

!"#$#%:;)'8!"#$#%:;)'%!"#$#%:;)''!"#$#%:;)'9!"#$#%:;)'&!"#$#%:;)':!"#$#%:;)9<!"#$#%:;)9(!"#$#%:;)9;!"#$#%:;)9)!"#$#%:;)98!"#$#%:;)9%!"#$#%:;)9'!"#$#%:;)99!"#$#%:;)9&!"#$#%:;)9:!"#$#%:;)&<!"#$#%:;)&(!"#$#%:;)&;!"#$#%:;)&)!"#$#%:;)&8!"#$#%:;)&%!"#$#%:;)&'!"#$#%:;)&9 !"#$#%:;)&&!"#$#%:;)&:!"#$#%:;):<!"#$#%:;):(!"#$#%:;):;!"#$#%:;):)!"#$#%:;):8!"#$#%:;):%!"#$#%:;):'!"#$#%:;):9!"#$#%:;):&!"#$#%:;)::!"#$#%:;8<<!"#$#%:;8<(!"#$#%:;8<;!"#$#%:;8<)!"#$#%:;8<8!"#$#%:;8<%!"#$#%:;8<'!"#$#%:;8<9!"#$#%:;8<&!"#$#%:;8<:!"#$#%:;8(<!"#$#%:;8(( !"#$#%:;8(;!"#$#%:;8()!"#$#%:;8(8!"#$#%:;8(%!"#$#%:;8('!"#$#%:;8(9!"#$#%:;8(&!"#$#%:;8(:!"#$#%:;8;<!"#$#%:;8;(!"#$#%:;8;;!"#$#%:;8;)!"#$#%:;8;8!"#$#%:;8;%!"#$#%:;8;'!"#$#%:;8;9!"#$#%:;8;&!"#$#%:;8;:!"#$#%:;8)<!"#$#%:;8)(!"#$#%:;8);!"#$#%:;8))!"#$#%:;8)8!"#$#%:;8)% !"#$#%:;8)'!"#$#%:;8)9!"#$#%:;8)&!"#$#%:;8):!"#$#%:;88<!"#$#%:;88(!"#$#%:;88;!"#$#%:;88)!"#$#%:;888!"#$#%:;88%!"#$#%:;88'!"#$#%:;889!"#$#%:;88&!"#$#%:;88:!"#$#%:;8%<!"#$#%:;8%(!"#$#%:;8%;!"#$#%:;8%)!"#$#%:;8%8!"#$#%:;8%%!"#$#%:;8%'!"#$#%:;8%9!"#$#%:;8%&!"#$#%:;8%:

!"#$#%:;8'<!"#$#%:;8'(!"#$#%:;8';!"#$#%:;8')!"#$#%:;8'8!"#$#%:;8'%!"#$#%:;8''!"#$#%:;8'9!"#$#%:;8'&!"#$#%:;8':!"#$#%:;89<!"#$#%:;89(!"#$#%:;89;!"#$#%:;89)!"#$#%:;898!"#$#%:;89%!"#$#%:;89'!"#$#%:;899!"#$#%:;89&!"#$#%:;89:!"#$#%:;8&<!"#$#%:;8&(!"#$#%:;8&;!"#$#%:;8&)

!"#$#%:;8&8

|  | , 5A" 7 $?,5A"7$@ | ,5A"7$ | 0 4, * , 1 |
| --- | --- | --- | --- |
| !"#$#%:;8&%!"#$#%:;8&' | > /$?>/$@ | >/$,5A"7$ | - $ 0 , 4, * + 1 |
| !"#$#%:;8&9 | , 5A" 7 $?,5A"7$@ | ,5A"7$ | 0 4, * , 1 |

!"#$#%:;8&&!"#$#%:;8&:!"#$#%:;8:<!"#$#%:;8:(!"#$#%:;8:;!"#$#%:;8:)!"#$#%:;8:8!"#$#%:;8:%!"#$#%:;8:'!"#$#%:;8:9!"#$#%:;8:&!"#$#%:;8::!"#$#%:;%<<!"#$#%:;%<(!"#$#%:;%<;!"#$#%:;%<)!"#$#%:;%<8!"#$#%:;%<%!"#$#%:;%<'!"#$#%:;%<9!"#$#%:;%<:

| > /$?>/$@ >/$,5A"7$ | | | | - $ 0 , 4, * + 1 |
| --- | --- | --- | --- | --- |
| !"#$#%:;%(< , 5A" 7 $?,5A"7$@ ,5A"7$ | | | | 0 4, * , 1 |
| !"#$#%:;%((!"#$#%:;%(;!"#$#%:;%() > /$?>/$@ >/$,5A"7$  !"#$#%:;%(8!"#$#%:;%(%!"#$#%:;%('  !"#$#%:;%(9!"#$#%:;%(&!"#$#%:;%(:!"#$#%:;%;<!"#$#%:;%;(!"#$#%:;%;;!"#$#%:;%;)!"#$#%:;%;8!"#$#%:;%;%!"#$#%:;%;'!"#$#%:;%;9!"#$#%:;%;&!"#$#%:;%;:!"#$#%:;%)<!"#$#%:;%)' | | | | - $ 0 , 4, * + 1 |
| , 5A" 7 $?,5A"7$@ ,5A"7$ | | | | 0 4, * , 1 |
|  | !"#$#%:;%)9 | > /$?>/$@ | >/$,5A"7$ | - $ 0 , 4, * + 1 |
|  | !"#$#%:;%)&!"#$#%:;%):!"#$#%:;%8< !"#$#%:;%8(!"#$#%:;%8;!"#$#%:;%8) !"#$#%:;%88!"#$#%:;%8%!"#$#%:;%8' !"#$#%:;%89 | , 5A" 7 $?,5A"7$@ | ,5A"7$ | 0 4, * , 1 |
|  | !"#$#%:;%8& | > /$?>/$@ | >/$,5A"7$ | - $ 0 , 4, * + 1 |

!"#$#%:;%8:!"#$#%:;%%<!"#$#%:;%%(!"#$#%:;%%;!"#$#%:;%%)!"#$#%:;%%8!"#$#%:;%%%!"#$#%:;%%'!"#$#%:;%%9!"#$#%:;%%&!"#$#%:;%%:!"#$#%:;%'<!"#$#%:;%'(!"#$#%:;%';!"#$#%:;%')

| , 5A" 7 $?,5A"7$@ ,5A"7$ | 0 4, * , 1 |
| --- | --- |
| !"#$#%:;%'8 > /$?>/$@ >/$,5A"7$ | - $ 0 , 4, * + 1 |
| !"#$#%:;%'%!"#$#%:;%''!"#$#%:;%'9 , 5A" 7 $?,5A"7$@ ,5A"7$ | 0 4, * , 1 |
| !"#$#%:;%'& > /$?>/$@ >/$,5A"7$ | - $ 0 , 4, * + 1 |
| !"#$#%:;%':!"#$#%:;%9<!"#$#%:;%9( , 5A" 7 $?,5A"7$@ ,5A"7$ !"#$#%:;%9;!"#$#%:;%9)!"#$#%:;%98 !"#$#%:;%9%!"#$#%:;%9' | 0 4, * , 1 |
| !"#$#%:;%99 > /$?>/$@ >/$,5A"7$  !"#$#%:;%9&!"#$#%:;%9:!"#$#%:;%&<!"#$#%:;%&(!"#$#%:;%&;!"#$#%:;%&)!"#$#%:;%&8!"#$#%:;%&%!"#$#%:;%&'!"#$#%:;%&9!"#$#%:;%&&!"#$#%:;%&: | - $ 0 , 4, * + 1 |
| , 5A" 7 $?,5A"7$@ ,5A"7$ | 0 4, * , 1 |
| !"#$#%:;%:< > /$?>/$@ >/$,5A"7$ | - $ 0 , 4, * + 1 |
| !"#$#%:;%:(!"#$#%:;%:;!"#$#%:;%:) , 5A" 7 $?,5A"7$@ ,5A"7$  !"#$#%:;%:8!"#$#%:;%:%!"#$#%:;%:' | 0 4, * , 1 |
| !"#$#%:;%:9 > /$?>/$@ >/$,5A"7$ | - $ 0 , 4, * + 1 |
| !"#$#%:;%:&!"#$#%:;%:: , 5A" 7 $?,5A"7$@ ,5A"7$ | 0 4, * , 1 |
| !"#$#%:;'<< > /$?>/$@ >/$,5A"7$ | - $ 0 , 4, * + 1 |

!"#$#%:;'<(!"#$#%:;'<;!"#$#%:;'<)!"#$#%:;'<8!"#$#%:;'<%!"#$#%:;'<'!"#$#%:;'<9!"#$#%:;'<&!"#$#%:;'<:!"#$#%:;'(<!"#$#%:;'((!"#$#%:;'(;!"#$#%:;'()!"#$#%:;'(8!"#$#%:;'(%

, 5A" 7 $?,5A"7$@ ,5A"7$ 0 4, * , 1

!"#$#%:;'(' > /$?>/$@ >/$,5A"7$ - $ 0 , 4, * + 1

!"#$#%:;'(9!"#$#%:;'(&!"#$#%:;'(:!"#$#%:;';<!"#$#%:;';(!"#$#%:;';;!"#$#%:;';)!"#$#%:;';8!"#$#%:;';%!"#$#%:;';'!"#$#%:;';9!"#$#%:;';&!"#$#%:;';:!"#$#%:;')<!"#$#%:;')(!"#$#%:;');!"#$#%:;'))!"#$#%:;')8!"#$#%:;')%!"#$#%:;')'!"#$#%:;')9!"#$#%:;')&!"#$#%:;'):!"#$#%:;'8< !"#$#%:;'8(!"#$#%:;'8;!"#$#%:;'8)!"#$#%:;'88!"#$#%:;'8%!"#$#%:;'8'!"#$#%:;'89!"#$#%:;'8&!"#$#%:;'8:!"#$#%:;'%<!"#$#%:;'%(!"#$#%:;'%;!"#$#%:;'%)!"#$#%:;'%8!"#$#%:;'%%!"#$#%:;'%'!"#$#%:;'%9!"#$#%:;'%&!"#$#%:;'%:

|  | , 5A" 7 $?,5A"7$@ | ,5A"7$ | 0 4, * , 1 |
| --- | --- | --- | --- |
| !"#$#%:;''<!"#$#%:;''( | > /$?>/$@ | >/$,5A"7$ | - $ 0 , 4, * + 1 |
| !"#$#%:;'';!"#$#%:;'')!"#$#%:;''8 !"#$#%:;''%!"#$#%:;'''!"#$#%:;''9 | , 5A" 7 $?,5A"7$@ | ,5A"7$ | 0 4, * , 1 |
| !"#$#%:;''& | > /$?>/$@ | >/$,5A"7$ | - $ 0 , 4, * + 1 |

!"#$#%:;'':!"#$#%:;'9<!"#$#%:;'9(!"#$#%:;'9;!"#$#%:;'9)!"#$#%:;'98!"#$#%:;'9%!"#$#%:;'9'!"#$#%:;'99!"#$#%:;'9&!"#$#%:;'9:!"#$#%:;'&<!"#$#%:;'&(!"#$#%:;'&;!"#$#%:;'&)!"#$#%:;'&8!"#$#%:;'&%!"#$#%:;'&'!"#$#%:;'&9!"#$#%:;'&&!"#$#%:;'&:!"#$#%:;':<!"#$#%:;':(!"#$#%:;':;

| !"#$#%:;':)!"#$#%:;':8!"#$#%:;':%!"#$#%:;':'!"#$#%:;':9!"#$#%:;':&!"#$#%:;'::!"#$#%:;9<<!"#$#%:;9<(!"#$#%:;9<;!"#$#%:;9<)!"#$#%:;9<8!"#$#%:;&(: | | |  |
| --- | --- | --- | --- |
|  | , 5A" 7 $?,5A"7$@ | ,5A"7$ | 0 4, * , 1 |
| !"#$#%:;&;<!"#$#%:;&;( | > /$?>/$@ | >/$,5A"7$ | - $ 0 , 4, * + 1 |
| !"#$#%:;&;;!"#$#%:;&;) | , 5A" 7 $?,5A"7$@ | ,5A"7$ | 0 4, * , 1 |
| !"#$#%:;&;8 | > /$?>/$@ | >/$,5A"7$ | - $ 0 , 4, * + 1 |
| !"#$#%:;&;%!"#$#%:;&;'!"#$#%:;&;9 | , 5A" 7 $?,5A"7$@ | ,5A"7$ | 0 4, * , 1 |
| !"#$#%:;&;& | > /$?>/$@ | >/$,5A"7$ | - $ 0 , 4, * + 1 |
| !"#$#%:;&;:!"#$#%:;&)<!"#$#%:;&)( !"#$#%:;&);!"#$#%:;&))!"#$#%:;&)8 !"#$#%:;&)% | , 5A" 7 $?,5A"7$@ | ,5A"7$ | 0 4, * , 1 |
| !"#$#%:;&)'!"#$#%:;&)9 | > /$?>/$@ | >/$,5A"7$ | - $ 0 , 4, * + 1 |

!"#$#%:;&)&!"#$#%:;&):!"#$#%:;&8<!"#$#%:;&8(!"#$#%:;&8;!"#$#%:;&8)!"#$#%:;&88!"#$#%:;&8%!"#$#%:;&8'!"#$#%:;&89!"#$#%:;&8&!"#$#%:;&8:!"#$#%:;&%<!"#$#%:;&%(!"#$#%:;&%;!"#$#%:;&%)!"#$#%:;&%8!"#$#%:;&%%!"#$#%:;&%'!"#$#%:;&%9

| , 5A" 7 $?,5A"7$@ ,5A"7$ | 0 4, * , 1 |
| --- | --- |
| !"#$#%:;&%&!"#$#%:;&%:!"#$#%:;&'< > /$?>/$@ >/$,5A"7$ | - $ 0 , 4, * + 1 |
| !"#$#%:;&'( , 5A" 7 $?,5A"7$@ ,5A"7$ | 0 4, * , 1 |
| !"#$#%:;&';!"#$#%:;&')!"#$#%:;&'8 > /$?>/$@ >/$,5A"7$ !"#$#%:;&'%  !"#$#%:;&''!"#$#%:;&'9!"#$#%:;&'&!"#$#%:;&':!"#$#%:;&9<!"#$#%:;&9(!"#$#%:;&9;!"#$#%:;&9)!"#$#%:;&98!"#$#%:;&9%!"#$#%:;&9'!"#$#%:;&99 | - $ 0 , 4, * + 1 |
| , 5A" 7 $?,5A"7$@ ,5A"7$ | 0 4, * , 1 |
| !"#$#%:;&9&!"#$#%:;&9:!"#$#%:;&&< > /$?>/$@ >/$,5A"7$ | - $ 0 , 4, * + 1 |

!"#$#%:;&&(

!"#$#%:;&&;!"#$#%:;&&)!"#$#%:;&&8!"#$#%:;&&%!"#$#%:;&&'!"#$#%:;&&9!"#$#%:;&&&!"#$#%:;&&:!"#$#%:;&:<!"#$#%:;&:(!"#$#%:;&:;!"#$#%:;&:)!"#$#%:;&:8!"#$#%:;&:%!"#$#%:;&:'!"#$#%:;&:9!"#$#%:;&:&!"#$#%:;&::!"#$#%:;:<<!"#$#%:;:<(!"#$#%:;:<;!"#$#%:;:<)!"#$#%:;:<8!"#$#%:;:<% !"#$#%:;:<'!"#$#%:;:<9!"#$#%:;:<&!"#$#%:;:<:!"#$#%:;:(<!"#$#%:;:((!"#$#%:;:(;!"#$#%:;:()!"#$#%:;:(8!"#$#%:;:(%!"#$#%:;:('!"#$#%:;:(9!"#$#%:;:(&!"#$#%:;:(:!"#$#%:;:;<!"#$#%:;:;(!"#$#%:;:;;!"#$#%:;:;)!"#$#%:;:;8!"#$#%:;:;%!"#$#%:;:;'!"#$#%:;:;9!"#$#%:;:;&!"#$#%:;:;:

!"#$#%:;:)<!"#$#%:;:)(!"#$#%:;:);!"#$#%:;:))!"#$#%:;:)8!"#$#%:;:)%!"#$#%:;:)'!"#$#%:;:)9!"#$#%:;:)&!"#$#%:;:):!"#$#%:;:8<!"#$#%:;:8(!"#$#%:;:8;!"#$#%:;:8)!"#$#%:;:88!"#$#%:;:8%!"#$#%:;:8'!"#$#%:;:89!"#$#%:;:8&!"#$#%:;:8:!"#$#%:;:%<!"#$#%:;:%(!"#$#%:;:%;!"#$#%:;:%) !"#$#%:;:%8!"#$#%:;:%%!"#$#%:;:%'!"#$#%:;:%9

|  | , 5A" 7 $?,5A"7$@ | ,5A"7$ | 0 4, * , 1 |
| --- | --- | --- | --- |
| !"#$#%:;:%& | > /$?>/$@ | >/$,5A"7$ | - $ 0 , 4, * + 1 |
| !"#$#%:;:%:!"#$#%:;:'<!"#$#%:;:'( | , 5A" 7 $?,5A"7$@ | ,5A"7$ | 0 4, * , 1 |

!"#$#%:;:';!"#$#%:;:')!"#$#%:;:'8 !"#$#%:;:'%!"#$#%:;:''!"#$#%:;:'9

!"#$#%:;:'&

!"#$#%:;:':!"#$#%:;:9<!"#$#%:;:9(!"#$#%:;:9;!"#$#%:;:9)!"#$#%:;:98!"#$#%:;:9%!"#$#%:;:9'!"#$#%:;:99!"#$#%:;:9&!"#$#%:;:9:!"#$#%:;:&<!"#$#%:;:&(!"#$#%:;:&;!"#$#%:;:&)!"#$#%:;:&8!"#$#%:;:&%

> /$?>/$@ >/$,5A"7$ - $ 0 , 4, * + 1

!"#$#%:;:&'!"#$#%:;:&9!"#$#%:;:&&!"#$#%:;:&:!"#$#%:;::<!"#$#%:;::(!"#$#%:;::;!"#$#%:;::)!"#$#%:;::8!"#$#%:;::%!"#$#%:;::'!"#$#%:;::9!"#$#%:;::&!"#$#%:;:::!"#$#%:)<<<!"#$#%:)<<(!"#$#%:)<<;

|  | , 5A" 7 $?,5A"7$@ | ,5A"7$ | 0 4, * , 1 |
| --- | --- | --- | --- |
| !"#$#%:)<<)!"#$#%:)<<8!"#$#%:)<<% | > /$?>/$@ | >/$,5A"7$ | - $ 0 , 4, * + 1 |

!"#$#%:)<<'!"#$#%:)<<9

!"#$#%:)<<&!"#$#%:)<<:!"#$#%:)<(<!"#$#%:)<((!"#$#%:)<(;!"#$#%:)<()!"#$#%:)<(8!"#$#%:)<(%!"#$#%:)<('!"#$#%:)<(9!"#$#%:)<(&!"#$#%:)<(:!"#$#%:)<;<!"#$#%:)<;(!"#$#%:)<;;!"#$#%:)<;)!"#$#%:)<;8!"#$#%:)<;%!"#$#%:)<;'!"#$#%:)<;9!"#$#%:)<;&!"#$#%:)<;:!"#$#%:)<)<!"#$#%:)<)(

| !"#$#%:)<); |  |
| --- | --- |
| , 5A" 7 $?,5A"7$@ ,5A"7$ | 0 4, * , 1 |
| !"#$#%:)<)) > /$?>/$@ >/$,5A"7$ | - $ 0 , 4, * + 1 |
| !"#$#%:)<)8 , 5A" 7 $?,5A"7$@ ,5A"7$ | 0 4, * , 1 |
| !"#$#%:)<)% > /$?>/$@ >/$,5A"7$  !"#$#%:)<)'!"#$#%:)<)9!"#$#%:)<)&!"#$#%:)<):!"#$#%:)<8<!"#$#%:)<8(!"#$#%:)<8;!"#$#%:)<8)!"#$#%:)<88!"#$#%:)<8%!"#$#%:)<8'!"#$#%:)<89 | - $ 0 , 4, * + 1 |
| , 5A" 7 $?,5A"7$@ ,5A"7$ | 0 4, * , 1 |
| !"#$#%:)<8& > /$?>/$@ >/$,5A"7$ | - $ 0 , 4, * + 1 |

!"#$#%:)<8:!"#$#%:)<%<!"#$#%:)<%(!"#$#%:)<%;!"#$#%:)<%)!"#$#%:)<%8!"#$#%:)<%%!"#$#%:)<%'!"#$#%:)<%9!"#$#%:)<%&!"#$#%:)<%:!"#$#%:)<'<!"#$#%:)<'(!"#$#%:)<';!"#$#%:)<')!"#$#%:)<'8!"#$#%:)<'%!"#$#%:)<''!"#$#%:)<'9!"#$#%:)<'&!"#$#%:)<':!"#$#%:)<9<!"#$#%:)<9(!"#$#%:)<9; !"#$#%:)<9)!"#$#%:)<98!"#$#%:)<9%!"#$#%:)<9'!"#$#%:)<99!"#$#%:)<9&!"#$#%:)<9:!"#$#%:)<&<!"#$#%:)<&(!"#$#%:)<&;!"#$#%:)<&)!"#$#%:)<&8!"#$#%:)<&%!"#$#%:)<&'!"#$#%:)<&9!"#$#%:)<&&!"#$#%:)<&:!"#$#%:)<:<!"#$#%:)<:(!"#$#%:)<:;!"#$#%:)<:)!"#$#%:)<:8!"#$#%:)<:%!"#$#%:)<:' !"#$#%:)<:9!"#$#%:)<:&!"#$#%:)<::!"#$#%:)(<<!"#$#%:)(<(!"#$#%:)(<;!"#$#%:)(<)!"#$#%:)(<8!"#$#%:)(<%!"#$#%:)(<'!"#$#%:)(<9!"#$#%:)(<&!"#$#%:)(<:!"#$#%:)((<!"#$#%:)(((!"#$#%:)((;!"#$#%:)(()!"#$#%:)((8!"#$#%:)((%!"#$#%:)(('!"#$#%:)((9!"#$#%:)((&!"#$#%:)((:!"#$#%:)(;< !"#$#%:)(;(!"#$#%:)(;;!"#$#%:)(;)!"#$#%:)(;8

|  | , 5A" 7 $?,5A"7$@ | ,5A"7$ | 0 4, * , 1 |
| --- | --- | --- | --- |
| !"#$#%:)(;%!"#$#%:)(;'!"#$#%:)(;9 | > /$?>/$@ | >/$,5A"7$ | - $ 0 , 4, * + 1 |

!"#$#%:)(;&

!"#$#%:)(;:!"#$#%:)()<!"#$#%:)()(!"#$#%:)();!"#$#%:)())!"#$#%:)()8!"#$#%:)()%!"#$#%:)()'!"#$#%:)()9!"#$#%:)()&!"#$#%:)():!"#$#%:)(8<!"#$#%:)(8(!"#$#%:)(8;!"#$#%:)(8)!"#$#%:)(88!"#$#%:)(8%!"#$#%:)(8'!"#$#%:)(89!"#$#%:)(8&!"#$#%:)(8:!"#$#%:)(%<!"#$#%:)(%(!"#$#%:)(%; !"#$#%:)(%)!"#$#%:)(%8!"#$#%:)(%%!"#$#%:)(%'!"#$#%:)(%9!"#$#%:)(%&!"#$#%:)(%:!"#$#%:)('<!"#$#%:)('(!"#$#%:)(';!"#$#%:)(')!"#$#%:)('8!"#$#%:)('%!"#$#%:)(''!"#$#%:)('9!"#$#%:)('&!"#$#%:)(':!"#$#%:)(9<!"#$#%:)(9(!"#$#%:)(9;!"#$#%:)(9)!"#$#%:)(98!"#$#%:)(9%!"#$#%:)(9'

!"#$#%:)(99!"#$#%:)(9&!"#$#%:)(9:!"#$#%:)(&<!"#$#%:)(&(!"#$#%:)(&;!"#$#%:)(&)!"#$#%:)(&8!"#$#%:)(&%!"#$#%:)(&'!"#$#%:)(&9!"#$#%:)(&&!"#$#%:)(&:!"#$#%:)(:<!"#$#%:)(:(!"#$#%:)(:;!"#$#%:)(:)!"#$#%:)(:8!"#$#%:)(:%!"#$#%:)(:'!"#$#%:)(:9!"#$#%:)(:&!"#$#%:)(::!"#$#%:);<<

!"#$#%:);<(!"#$#%:);<;!"#$#%:);<)!"#$#%:);<8!"#$#%:);<%!"#$#%:);<'!"#$#%:);<9!"#$#%:);<&!"#$#%:);<:!"#$#%:);(<!"#$#%:);((!"#$#%:);(;!"#$#%:);()!"#$#%:);(8

, 5A" 7 $?,5A"7$@ ,5A"7$ 0 4, * , 1

!"#$#%:);(%!"#$#%:);(' > /$?>/$@ >/$,5A"7$ - $ 0 , 4, * + 1

!"#$#%:);(9!"#$#%:);(&!"#$#%:);(:!"#$#%:);;<!"#$#%:);;(!"#$#%:);;;!"#$#%:);;)!"#$#%:);;8!"#$#%:);;%!"#$#%:);;'!"#$#%:);;9!"#$#%:);;&!"#$#%:);;:!"#$#%:);)<!"#$#%:);)(!"#$#%:););!"#$#%:);))!"#$#%:);)8!"#$#%:);)%!"#$#%:);)'!"#$#%:);)9!"#$#%:);)&!"#$#%:);):!"#$#%:);8<

!"#$#%:);8(!"#$#%:);8;!"#$#%:);8)!"#$#%:);88!"#$#%:);8%!"#$#%:);8'!"#$#%:);89!"#$#%:);8&!"#$#%:);8:!"#$#%:);%<!"#$#%:);%(!"#$#%:);%;!"#$#%:);%)!"#$#%:);%8!"#$#%:);%%

, 5A" 7 $?,5A"7$@ ,5A"7$ 0 4, * , 1

!"#$#%:);%' > /$?>/$@ >/$,5A"7$ - $ 0 , 4, * + 1

!"#$#%:);%9!"#$#%:);%&!"#$#%:);%:!"#$#%:);'<!"#$#%:);'(!"#$#%:);';!"#$#%:);')!"#$#%:);'8!"#$#%:);'%!"#$#%:);''!"#$#%:);'9!"#$#%:);'&!"#$#%:);':!"#$#%:);9<!"#$#%:);9(!"#$#%:);9;!"#$#%:);9)!"#$#%:);98!"#$#%:);9%!"#$#%:);9'!"#$#%:);99!"#$#%:);9&!"#$#%:);9:!"#$#%:);&< !"#$#%:);&(!"#$#%:);&;!"#$#%:);&)!"#$#%:);&8!"#$#%:);&%!"#$#%:);&'!"#$#%:);&9!"#$#%:);&&!"#$#%:);&:!"#$#%:);:<!"#$#%:);:(!"#$#%:);:;!"#$#%:);:)!"#$#%:);:8!"#$#%:);:%!"#$#%:);:'!"#$#%:);:9!"#$#%:);:&!"#$#%:);::!"#$#%:))<<!"#$#%:))<(!"#$#%:))<;!"#$#%:))<)!"#$#%:))<8 !"#$#%:))<%!"#$#%:))<'!"#$#%:))<9!"#$#%:))<&!"#$#%:))<:!"#$#%:))(<!"#$#%:))((!"#$#%:))(;!"#$#%:))()!"#$#%:))(8!"#$#%:))(%!"#$#%:))('!"#$#%:))(9!"#$#%:))(&!"#$#%:))(:!"#$#%:));<!"#$#%:));(!"#$#%:));;!"#$#%:));)!"#$#%:));8!"#$#%:));%!"#$#%:));'!"#$#%:));9!"#$#%:));& !"#$#%:));:!"#$#%:)))<!"#$#%:)))(!"#$#%:)));!"#$#%:))))!"#$#%:)))8!"#$#%:)))%!"#$#%:)))'!"#$#%:)))9!"#$#%:)))&!"#$#%:))):!"#$#%:))8<!"#$#%:))8(!"#$#%:))8;!"#$#%:))8)!"#$#%:))88!"#$#%:))8%!"#$#%:))8'!"#$#%:))89!"#$#%:))8&!"#$#%:))8:!"#$#%:))%<!"#$#%:))%(!"#$#%:))%;

!"#$#%:))%)!"#$#%:))%8!"#$#%:))%%!"#$#%:))%'!"#$#%:))%9!"#$#%:))%&!"#$#%:))%:!"#$#%:))'<!"#$#%:))'(!"#$#%:))';!"#$#%:))')!"#$#%:))'8!"#$#%:))'%!"#$#%:))''!"#$#%:))'9!"#$#%:))'&!"#$#%:))':!"#$#%:))9<!"#$#%:))9(!"#$#%:))9;!"#$#%:))9)!"#$#%:))98!"#$#%:))9%!"#$#%:))9'

!"#$#%:))99!"#$#%:))9&!"#$#%:))9:!"#$#%:))&<!"#$#%:))&(!"#$#%:))&;!"#$#%:))&)!"#$#%:))&8!"#$#%:))&%!"#$#%:))&'!"#$#%:))&9!"#$#%:))&&!"#$#%:))&:!"#$#%:)):<!"#$#%:)):(!"#$#%:)):;!"#$#%:)):)!"#$#%:)):8!"#$#%:)):%!"#$#%:)):'!"#$#%:)):9!"#$#%:)):&!"#$#%:))::!"#$#%:)8<<

!"#$#%:)8<(!"#$#%:)8<;!"#$#%:)8<)!"#$#%:)8<8!"#$#%:)8<%!"#$#%:)8<'!"#$#%:)8<9!"#$#%:)8<&!"#$#%:)8<:!"#$#%:)8(<

, 5A" 7 $?,5A"7$@ ,5A"7$ 0 4, * , 1

!"#$#%:)8((!"#$#%:)8(; > /$?>/$@ >/$,5A"7$ - $ 0 , 4, * + 1

!"#$#%:)8()!"#$#%:)8(8!"#$#%:)8(%!"#$#%:)8('!"#$#%:)8(9!"#$#%:)8(&!"#$#%:)8(:!"#$#%:)8;<!"#$#%:)8;(!"#$#%:)8;;!"#$#%:)8;)!"#$#%:)8;8!"#$#%:)8;%!"#$#%:)8;'!"#$#%:)8;9!"#$#%:)8;&!"#$#%:)8;:!"#$#%:)8)<!"#$#%:)8)(!"#$#%:)8);!"#$#%:)8))!"#$#%:)8)8!"#$#%:)8)%!"#$#%:)8)' !"#$#%:)8)9!"#$#%:)8)&!"#$#%:)8):!"#$#%:)88<!"#$#%:)88(!"#$#%:)88;!"#$#%:)88)!"#$#%:)888!"#$#%:)88%!"#$#%:)88'!"#$#%:)889!"#$#%:)88&!"#$#%:)88:!"#$#%:)8%<!"#$#%:)8%(!"#$#%:)8%;!"#$#%:)8%)!"#$#%:)8%8!"#$#%:)8%%!"#$#%:)8%'!"#$#%:)8%9!"#$#%:)8%&!"#$#%:)8%:!"#$#%:)8'<

!"#$#%:)8'(!"#$#%:)8';!"#$#%:)8')!"#$#%:)8'8!"#$#%:)8'%!"#$#%:)8''!"#$#%:)8'9!"#$#%:)8'&!"#$#%:)8':!"#$#%:)89<!"#$#%:)89(!"#$#%:)89;!"#$#%:)89)!"#$#%:)898!"#$#%:)89%

|  | , 5A" 7 $?,5A"7$@ | ,5A"7$ | 0 4, * , 1 |
| --- | --- | --- | --- |
| !"#$#%:)89'!"#$#%:)899 | > /$?>/$@ | >/$,5A"7$ | - $ 0 , 4, * + 1 |
| !"#$#%:)89&!"#$#%:)89:!"#$#%:)8&< | *K7 | +$$ | ,- - +/+3 $ |

!"#$#%:)8&(!"#$#%:)8&)!"#$#%:)8&'!"#$#%:)8&9!"#$#%:)8&:!"#$#%:)8:<!"#$#%:)8:(!"#$#%:)8:;!"#$#%:)8:)!"#$#%:)8:8!"#$#%:)8:%!"#$#%:)8:'!"#$#%:)8:9!"#$#%:)8:&!"#$#%:)8::!"#$#%:)%<(!"#$#%:)%<;!"#$#%:)%<)!"#$#%:)%<8!"#$#%:)%<'!"#$#%:)%<9!"#$#%:)%<&!"#$#%:)%<:!"#$#%:)%(( !"#$#%:)%(;!"#$#%:)%()!"#$#%:)%(8!"#$#%:)%(%!"#$#%:)%(9!"#$#%:)%(&!"#$#%:)%(:!"#$#%:)%;)!"#$#%:)%;8!"#$#%:)%;%!"#$#%:)%;'!"#$#%:)%;9!"#$#%:)%;&!"#$#%:)%;:!"#$#%:)%)(!"#$#%:)%);!"#$#%:)%))!"#$#%:)%)8!"#$#%:)%)'!"#$#%:)%)9!"#$#%:)%)&!"#$#%:)%):!"#$#%:)%8<!"#$#%:)%8(

!"#$#%:)%8)!"#$#%:)%88!"#$#%:)%8%!"#$#%:)%8'!"#$#%:)%89!"#$#%:)%8&!"#$#%:)%8:!"#$#%:)%%<!"#$#%:)%%(

|  | !/ $ | ,,5 | $,7 ! " + 1/ , |
| --- | --- | --- | --- |
| !"#$#%:)%%)!"#$#%:)%%8!"#$#%:)%%% | *K7 | +$$ | ,- - +/+3 $ |

!"#$#%:)%%'!"#$#%:)%%9!"#$#%:)%%&

!"#$#%:)%')!"#$#%:)%'8!"#$#%:)%'%!"#$#%:)%''!"#$#%:)%'&!"#$#%:)%':!"#$#%:)%9(!"#$#%:)%9;!"#$#%:)%98!"#$#%:)%9%!"#$#%:)%9'!"#$#%:)%99!"#$#%:)%9&!"#$#%:)%9:!"#$#%:)%&(!"#$#%:)%&;!"#$#%:)%&8!"#$#%:)%&9!"#$#%:)%&&!"#$#%:)%&:!"#$#%:)%:(!"#$#%:)%:;!"#$#%:)%:)!"#$#%:)%:8 !"#$#%:)%:%

|  | 0F7 | 0F7 | /0 *+3, /" ,2- / $2 , " 4 |
| --- | --- | --- | --- |
| !"#$#%:)%:' | , - 5 | * | + 22/G - 1 +- /1 1E= E 42! =+A= $2 - 0-+ " $ |

E " , ,+ 7 /12, , = +7 + +. / *" "0 1 6$ 0 + " *+/ 7

!"#$#%:)%:9!"#$#%:)%:&!"#$#%:)%::!"#$#%:)'<<!"#$#%:)'<(!"#$#%:)'<;!"#$#%:)'<)!"#$#%:)'<8!"#$#%:)'<%!"#$#%:)'<'!"#$#%:)'<9!"#$#%:)'<&!"#$#%:)'<:!"#$#%:)'(<!"#$#%:)'((!"#$#%:)'(;!"#$#%:)'()!"#$#%:)'(8!"#$#%:)'(%!"#$#%:)'('!"#$#%:)'(9!"#$#%:)'(&!"#$#%:)'(:!"#$#%:)';< !"#$#%:)';(!"#$#%:)';;!"#$#%:)';)!"#$#%:)';8!"#$#%:)';%!"#$#%:)';'!"#$#%:)';9!"#$#%:)';&!"#$#%:)';:!"#$#%:)')<!"#$#%:)')(!"#$#%:)');!"#$#%:)'))!"#$#%:)')8!"#$#%:)')%!"#$#%:)')'!"#$#%:)')9!"#$#%:)')&!"#$#%:)'):!"#$#%:)'8<!"#$#%:)'8(!"#$#%:)'8;!"#$#%:)'8)!"#$#%:)'88

!"#$#%:)'8%

|  |  | " 7 > $E ?"7>AE@ | 1 |
| --- | --- | --- | --- |
| !"#$#%:)'8'!"#$#%:)'89!"#$#%:)'8& | 8-" | 17 " A -  " , /J 7 J5 | -,A"7 |
| !"#$#%:)'8: | , $ | 17 " A -  " , /J 7 J5 | -,A"7 |
| !"#$#%:)'%<!"#$#%:)'%( | 7 ," | 17 " A -  " , /J 7 J5 | -,A"7 |
| !"#$#%:)'%; | 7 | 17 " A -  " , /J 7 J5 | -,A"7 |
| !"#$#%:)'%) | $" | 17 " A -  " , /J 7 J5 | -,A"7 |
| !"#$#%:)'%8 | , " | 17 " A -  " , /J 7 J5 | -,A"7 |
| !"#$#%:)'%%!"#$#%:)'%'!"#$#%:)'%9  !"#$#%:)'%& | " 1A7 A17 " | 17 " A -  " , /J 7 J5 | -,A"7 |
| !"#$#%:)'%: | " 1A/ 17 A17 " | 17 " A - | -,A"7 |

" , /J

7 J5

!"#$#%:)''<!"#$#%:)''(!"#$#%:)'';!"#$#%:)'')!"#$#%:)''8!"#$#%:)''%!"#$#%:)'''!"#$#%:)''9!"#$#%:)''&!"#$#%:)'':!"#$#%:)'9<!"#$#%:)'9(!"#$#%:)'9;!"#$#%:)'9)!"#$#%:)'98!"#$#%:)'9%!"#$#%:)'9'!"#$#%:)'99!"#$#%:)'9&!"#$#%:)'9:!"#$#%:)'&<!"#$#%:)'&(!"#$#%:)'&;

" A17 " 17 " A - -,A"7

" , /J

7 J5

!"#$#%:)'&)!"#$#%:)'&8!"#$#%:)'&%!"#$#%:)'&'!"#$#%:)'&9!"#$#%:)'&&!"#$#%:)'&:!"#$#%:)':<!"#$#%:)':(!"#$#%:)':;!"#$#%:)':)!"#$#%:)':8!"#$#%:)':%!"#$#%:)':'!"#$#%:)':9!"#$#%:)':&!"#$#%:)'::!"#$#%:)9<<!"#$#%:)9<(!"#$#%:)9<;!"#$#%:)9<)!"#$#%:)9<8!"#$#%:)9<%!"#$#%:)9<' !"#$#%:)9<9!"#$#%:)9<&!"#$#%:)9<:!"#$#%:)9(<!"#$#%:)9((!"#$#%:)9(;!"#$#%:)9()!"#$#%:)9(8!"#$#%:)9(%!"#$#%:)9('!"#$#%:)9(9!"#$#%:)9(&!"#$#%:)9(:!"#$#%:)9;<!"#$#%:)9;(!"#$#%:)9;;!"#$#%:)9;)!"#$#%:)9;8!"#$#%:)9;%!"#$#%:)9;'!"#$#%:)9;9!"#$#%:)9;&!"#$#%:)9;:!"#$#%:)9)<

!"#$#%:)9)(!"#$#%:)9);!"#$#%:)9))!"#$#%:)9)8!"#$#%:)9)%!"#$#%:)9)'!"#$#%:)9)9!"#$#%:)9)&!"#$#%:)9):!"#$#%:)98<!"#$#%:)98(!"#$#%:)98;!"#$#%:)98)!"#$#%:)988!"#$#%:)98%!"#$#%:)98'!"#$#%:)989!"#$#%:)98&!"#$#%:)98:!"#$#%:)9%<!"#$#%:)9%(!"#$#%:)9%;!"#$#%:)9%)!"#$#%:)9%8

| !$?!$@ 17 " A -  " , /J  7 J5  !"#$#%:)9%%!"#$#%:)9%'!"#$#%:)9%9!"#$#%:)9%&!"#$#%:)9%:!"#$#%:)9'<!"#$#%:)9'(!"#$#%:)9';!"#$#%:)9')!"#$#%:)9'8!"#$#%:)9'% | | | | | | -,A"7 |
| --- | --- | --- | --- | --- | --- | --- |
|  | | " ?"@A$ 7 A  17 " | | 17 " A -  " , /J 7 J5 | | -,A"7 |
| !"#$#%:)9''!"#$#%:)9'9!"#$#%:)9'& | | " ?"@A/ "  7 A17 " | | 17 " A -  " , /J 7 J5 | | -,A"7 |
| !"#$#%:)9': | | 0- K7 | | 17 " A -  " , /J 7 J5 | | -,A"7 |
| !"#$#%:)99< | | " A17 " | | 17 " A -  " , /J 7 J5 | | -,A"7 |
| !"#$#%:)99( | | !$?!$@ | 17 " A -  " , /J 7 J5 | -,A"7 | | |
| !"#$#%:)99;!"#$#%:)99)!"#$#%:)998 !"#$#%:)99%!"#$#%:)99' | | 7"0$/!1$$,*=!H5! | !1,,=/ | E / A$ , $N4AB I7 AL4" 0 A>$ ,.A /I A,! ,MA$ + " -M | | |

!"#$#%:)9&9!"#$#%:)9&&!"#$#%:)9:<!"#$#%:)9:(!"#$#%:)9:;!"#$#%:)9:)!"#$#%:)9:8!"#$#%:)9:%!"#$#%:)9:'!"#$#%:)9:9!"#$#%:)9:&!"#$#%:)9::!"#$#%:)&<<!"#$#%:)&<(!"#$#%:)&<;!"#$#%:)&<)!"#$#%:)&<8!"#$#%:)&<'!"#$#%:)&<9!"#$#%:)&<&!"#$#%:)&<:!"#$#%:)&(<!"#$#%:)&((!"#$#%:)&() !"#$#%:)&(8!"#$#%:)&(%!"#$#%:)&('!"#$#%:)&(9!"#$#%:)&(&!"#$#%:)&(:!"#$#%:)&;<!"#$#%:)&;(!"#$#%:)&;;!"#$#%:)&;)!"#$#%:)&;8!"#$#%:)&;%!"#$#%:)&;'!"#$#%:)&;9!"#$#%:)&;&!"#$#%:)&;:!"#$#%:)&);!"#$#%:)&))!"#$#%:)&)8!"#$#%:)&)%!"#$#%:)&)'!"#$#%:)&)9!"#$#%:)&)&!"#$#%:)&):

!"#$#%:)&8<!"#$#%:)&8(!"#$#%:)&8;!"#$#%:)&8)!"#$#%:)&88!"#$#%:)&8%!"#$#%:)&8'!"#$#%:)&89!"#$#%:)&8&!"#$#%:)&8:!"#$#%:)&%<!"#$#%:)&%(!"#$#%:)&%)!"#$#%:)&%8

/> 5, - " 7 /> 5, "7!-B 0 ! - " 7 !

!"#$#%:)&%%!"#$#%:)&%'!"#$#%:)&%9!"#$#%:)&%&!"#$#%:)&%:!"#$#%:)&'<!"#$#%:)&'(!"#$#%:)&';!"#$#%:)&')!"#$#%:)&'8!"#$#%:)&'%!"#$#%:)&''!"#$#%:)&'9!"#$#%:)&'&!"#$#%:)&':!"#$#%:)&9<!"#$#%:)&9(!"#$#%:)&9;!"#$#%:)&9)!"#$#%:)&98!"#$#%:)&9%!"#$#%:)&9'!"#$#%:)&99!"#$#%:)&9& !"#$#%:)&9:!"#$#%:)&&<!"#$#%:)&&(!"#$#%:)&&;!"#$#%:)&&)!"#$#%:)&&8!"#$#%:)&&%!"#$#%:)&&'!"#$#%:)&&9!"#$#%:)&&&!"#$#%:)&&:!"#$#%:)&:<!"#$#%:)&:(!"#$#%:)&:;!"#$#%:)&:)!"#$#%:)&:8!"#$#%:)&:%!"#$#%:)&:'!"#$#%:)&:9!"#$#%:)&:&!"#$#%:)&::!"#$#%:):<<!"#$#%:):<(

|  | -75" A$> A EII* | -75" A$> A  EII* | $ + /1 , -/ 7 *" 0 6" 4+ |
| --- | --- | --- | --- |
| !"#$#%:):<; | $ | 1 /->  / " " | * E!A$O>! , > |
| !"#$#%:):<) | 7 | 1 /->  / " " | * E!A$O>! , > |
| !"#$#%:):<8!"#$#%:):<%!"#$#%:):<' !"#$#%:):<9!"#$#%:):<&!"#$#%:):<: | $ , | 1 /->  / " " | * E!A$O>! , > |
| !"#$#%:):(< | 7 * A* | 1 /->  / " " | * E!A$O>! , > |
| !"#$#%:):(( | 7 $--I6 | 1 /->  / " " | * E!A$O>! , > |
| !"#$#%:):(; | 7 , | 1 /->  / " " | * E!A$O>! , > |
| !"#$#%:):() | 7 0 | 1 /->  / " " | * E!A$O>! , > |
| !"#$#%:):(8 | 7 ,4 | 1 /->  / " " | * E!A$O>! , > |
| !"#$#%:):(% | 7 | 1 /->  / " " | * E!A$O>! , > |
| !"#$#%:):(' | 7 | 1 /->  / " " | * E!A$O>! , > |
| !"#$#%:):(9 | 7 , ,4 | 1 /->  / " " | * E!A$O>! , > |
| !"#$#%:):(& | 7 + | 1 /->  / " " | * E!A$O>! , > |
| !"#$#%:):(: | 7 | 1 /->  / " " | * E!A$O>! , > |
| !"#$#%:):;< | 7 ,4 | 1 /->  / " " | * E!A$O>! , > |
| !"#$#%:):;( | 7 E | 1 /->  / " " | * E!A$O>! , > |
| !"#$#%:):;;!"#$#%:):;) | 7 ,4O ,4 | 1 /->  / " " | * E!A$O>! , > |
| !"#$#%:):;8!"#$#%:):;%!"#$#%:):;'  !"#$#%:):;9!"#$#%:):;&!"#$#%:):;: !"#$#%:):)<!"#$#%:):)(!"#$#%:):); !"#$#%:):)) | $ ," 6 | 1 /->  / " " | * E!A$O>! , > |
| !"#$#%:):)8 | - A!A* | 1 /->  / " " | * E!A$O>! , > |
| !"#$#%:):)% | " A0 | 1 /->  / " " | * E!A$O>! , > |
| !"#$#%:):)' | E | 1 /->  / " " | * E!A$O>! , > |

!"#$#%:):)&!"#$#%:):):!"#$#%:):8<!"#$#%:):8(!"#$#%:):8;!"#$#%:):8)!"#$#%:):88!"#$#%:):8%!"#$#%:):89!"#$#%:):8&!"#$#%:):8:!"#$#%:):%<!"#$#%:):%(!"#$#%:):%;!"#$#%:):%)!"#$#%:):%8!"#$#%:):%%!"#$#%:):%'!"#$#%:):%9!"#$#%:):%&!"#$#%:):'<!"#$#%:):'(!"#$#%:):';!"#$#%:):') !"#$#%:):'8!"#$#%:):'%!"#$#%:):''!"#$#%:):'9!"#$#%:):'&!"#$#%:):':!"#$#%:):9<!"#$#%:):9(!"#$#%:):9;!"#$#%:):9)!"#$#%:):98!"#$#%:):9%!"#$#%:):9'!"#$#%:):99!"#$#%:):9&!"#$#%:):9:!"#$#%:):&<!"#$#%:):&(

" 7 $ " 7 $ 7

!"#$#%:):&;!"#$#%:):&8!"#$#%:):&%!"#$#%:):&'!"#$#%:):&9!"#$#%:):&&!"#$#%:):&:!"#$#%:)::<!"#$#%:)::(!"#$#%:)::;!"#$#%:)::)!"#$#%:)::8!"#$#%:)::%!"#$#%:)::'!"#$#%:)::9!"#$#%:)::&

/> 5, - " 7 /> 5, "7!-B 0 ! - " 7 !

!"#$#%:):::!"#$#%:8<<;!"#$#%:8<<%!"#$#%:8<<9!"#$#%:8<<&!"#$#%:8<<:!"#$#%:8<(<!"#$#%:8<((!"#$#%:8<(;!"#$#%:8<()!"#$#%:8<(8!"#$#%:8<(%!"#$#%:8<('!"#$#%:8<(9!"#$#%:8<(&!"#$#%:8<(:!"#$#%:8<;<!"#$#%:8<;(!"#$#%:8<;)!"#$#%:8<;'!"#$#%:8<;9!"#$#%:8<;&!"#$#%:8<)<!"#$#%:8<)( !"#$#%:8<);!"#$#%:8<)8!"#$#%:8<)%!"#$#%:8<)&!"#$#%:8<):!"#$#%:8<8<!"#$#%:8<8(!"#$#%:8<8;!"#$#%:8<8)!"#$#%:8<88!"#$#%:8<8%!"#$#%:8<8'!"#$#%:8<89!"#$#%:8<8&!"#$#%:8<8:!"#$#%:8<%<!"#$#%:8<%(!"#$#%:8<%;!"#$#%:8<%)!"#$#%:8<%8!"#$#%:8<%%!"#$#%:8<%'!"#$#%:8<%9!"#$#%:8<%&

!"#$#%:8<%:!"#$#%:8<'<!"#$#%:8<'(!"#$#%:8<';!"#$#%:8<')!"#$#%:8<'8!"#$#%:8<'%!"#$#%:8<''!"#$#%:8<'9!"#$#%:8<'&!"#$#%:8<':!"#$#%:8<9<!"#$#%:8<9(!"#$#%:8<9;!"#$#%:8<9)!"#$#%:8<98!"#$#%:8<9%!"#$#%:8<9'!"#$#%:8<9:!"#$#%:8<&<!"#$#%:8<&(!"#$#%:8<&)!"#$#%:8<&8!"#$#%:8<&'

!"#$#%:8<&9!"#$#%:8<&&!"#$#%:8<&:!"#$#%:8<:<!"#$#%:8<:(!"#$#%:8<:)!"#$#%:8<:8!"#$#%:8<:%!"#$#%:8<:9!"#$#%:8<:&!"#$#%:8<::!"#$#%:8(<(!"#$#%:8(<;!"#$#%:8(<)!"#$#%:8(<8!"#$#%:8(<%!"#$#%:8(<'!"#$#%:8(<9!"#$#%:8(<&!"#$#%:8((<!"#$#%:8(((!"#$#%:8((;!"#$#%:8(()!"#$#%:8((8

!"#$#%:8((%!"#$#%:8(('

|  | 5" 7 $ | 5" 7 $ | != 2 |
| --- | --- | --- | --- |
| !"#$#%:8((& | -5! !L | , 5  EH | + - E4* I"A> ,MB 4+ +I ,*A* I4 ,/ +"*E!4!$4 >N*M"/.A 0 , "P -M |
| !"#$#%:8(;(!"#$#%:8(;8!"#$#%:8(;9 !"#$#%:8(;&!"#$#%:8()(!"#$#%:8()) | = ->A(:* | $A= " 7 | +E 0 * - > ,"-2 --, , $ ,0 " "$ /, 1 E - - 4 21 |
| !"#$#%:8(8& | 7 | 0" 7 | A, $,$2 ,7 /4 2 $.*"!B ,*/20 A |
| !"#$#%:8(8:!"#$#%:8(%<!"#$#%:8(%( !"#$#%:8(%;!"#$#%:8(%) | 2 $ | 0" 7 | A, $,$2 ,7 /4 2 $.*"!B ,*/20 A |
| !"#$#%:8(%8 | 5 ! | 0" 7 | A, $,$2 ,7 /4 2 $.*"!B ,*/20 A |
| !"#$#%:8(%%!"#$#%:8(%'!"#$#%:8(%9  !"#$#%:8(%&!"#$#%:8(%:!"#$#%:8('< !"#$#%:8('(!"#$#%:8('; | * / | * / | 3-A $ = $! , *A3 |
| !"#$#%:8(')!"#$#%:8('8!"#$#%:8('% |  | 1 /->  / " " | * E!A$O>! , > |
| !"#$#%:8('9!"#$#%:8(9<!"#$#%:8(9( !"#$#%:8(98!"#$#%:8(9%!"#$#%:8(9: | "$, | "$,,  5 | "$,, 5 |
| !"#$#%:8(&%!"#$#%:8(&'!"#$#%:8(&9  !"#$#%:8(&& | " ,27  05, | /-5 / | 3E,4,2.+ 44 ,4 + 47 4 + 44 ,4 /4 1. 224,+471. |

!"#$#%:8;8&!"#$#%:8;8:!"#$#%:8;%(!"#$#%:8;%;!"#$#%:8;%)!"#$#%:8;%%!"#$#%:8;%'!"#$#%:8;%&!"#$#%:8;%:!"#$#%:8;'<!"#$#%:8;';!"#$#%:8;')!"#$#%:8;'%!"#$#%:8;''!"#$#%:8;'9!"#$#%:8;'&!"#$#%:8;':!"#$#%:8;9<!"#$#%:8;9(!"#$#%:8;9;!"#$#%:8;9)!"#$#%:8;98!"#$#%:8;9%

5" 7 $ 5" 7 $ != 2

!"#$#%:8;9:!"#$#%:8;&<!"#$#%:8;&(!"#$#%:8;&;!"#$#%:8;&)!"#$#%:8;&8!"#$#%:8;&'!"#$#%:8;&9!"#$#%:8;&&!"#$#%:8;&:!"#$#%:8;:<!"#$#%:8;:(!"#$#%:8;:;!"#$#%:8;:)!"#$#%:8;:8!"#$#%:8;:%!"#$#%:8;:'!"#$#%:8;:9!"#$#%:8;:&!"#$#%:8;::!"#$#%:8)<<!"#$#%:8)<(!"#$#%:8)<;!"#$#%:8)<) !"#$#%:8)<8!"#$#%:8)<%!"#$#%:8)<'!"#$#%:8)<9!"#$#%:8)<&!"#$#%:8)<:!"#$#%:8)(<!"#$#%:8)((!"#$#%:8)(;!"#$#%:8)()!"#$#%:8)(8!"#$#%:8)(%!"#$#%:8)('!"#$#%:8)(9!"#$#%:8)(&!"#$#%:8)(:!"#$#%:8);<!"#$#%:8);(!"#$#%:8);;!"#$#%:8);8!"#$#%:8);%!"#$#%:8);'!"#$#%:8);9!"#$#%:8);& !"#$#%:8);:!"#$#%:8))<!"#$#%:8))(!"#$#%:8));!"#$#%:8)))!"#$#%:8))8!"#$#%:8))%!"#$#%:8))'!"#$#%:8))9!"#$#%:8))&!"#$#%:8)):!"#$#%:8)8<!"#$#%:8)8(!"#$#%:8)8;!"#$#%:8)8)!"#$#%:8)88!"#$#%:8)8%!"#$#%:8)8'!"#$#%:8)89!"#$#%:8)8&!"#$#%:8)8:!"#$#%:8)%<!"#$#%:8)%(!"#$#%:8)%; !"#$#%:8)%)!"#$#%:8)%8!"#$#%:8)%%!"#$#%:8)%'!"#$#%:8)%9!"#$#%:8)%&!"#$#%:8)%:!"#$#%:8)'<!"#$#%:8)'(!"#$#%:8)';!"#$#%:8)')!"#$#%:8)'8!"#$#%:8)'%!"#$#%:8)''!"#$#%:8)'9!"#$#%:8)'&!"#$#%:8)':!"#$#%:8)9<!"#$#%:8)9(!"#$#%:8)9)!"#$#%:8)98!"#$#%:8)9%!"#$#%:8)9'!"#$#%:8)99

!"#$#%:8)9&!"#$#%:8)9:!"#$#%:8)&<!"#$#%:8)&(!"#$#%:8)&;!"#$#%:8)&)!"#$#%:8)&8!"#$#%:8)&%!"#$#%:8)&'!"#$#%:8)&&!"#$#%:8)&:!"#$#%:8):(!"#$#%:8):)

E * " 7 $ E * " 7 $ +*

!"#$#%:8):8!"#$#%:8):%!"#$#%:8):'!"#$#%:8):9!"#$#%:8):&!"#$#%:8)::!"#$#%:88<<!"#$#%:88<(!"#$#%:88<;!"#$#%:88<)!"#$#%:88<8!"#$#%:88<'!"#$#%:88<9!"#$#%:88<:!"#$#%:88(<!"#$#%:88((!"#$#%:88(;!"#$#%:88()!"#$#%:88(8!"#$#%:88(%!"#$#%:88('!"#$#%:88(9!"#$#%:88(&!"#$#%:88(:

!"#$#%:88;<!"#$#%:88;(!"#$#%:88;;!"#$#%:88;)!"#$#%:88;%!"#$#%:88;9!"#$#%:88;&!"#$#%:88;:!"#$#%:88)<!"#$#%:88)(!"#$#%:88);!"#$#%:88))!"#$#%:88)&!"#$#%:88):!"#$#%:888(!"#$#%:888;!"#$#%:888)!"#$#%:8888

|  | 5" 7 $ | 5" 7 $ | != 2 |
| --- | --- | --- | --- |
| !"#$#%:8889!"#$#%:888&!"#$#%:888:  !"#$#%:88%<!"#$#%:88%(!"#$#%:88%; !"#$#%:88%)!"#$#%:88%8 | " 7 $ | "/> *  > - - " | =0=$- "+32H 57+ * 60 |
| !"#$#%:88%% | 067 " 7 $ | "/> *  > - - " | =0=$- "+32H 57+ * 60 |
| !"#$#%:88%' | " 7 $ | "/> *  > - - " | =0=$- "+32H 57+ * 60 |
| !"#$#%:88%9 | 1=7 | "/> *  > - - " | =0=$- "+32H 57+ * 60 |
| !"#$#%:88%& | - " 7 | "/> *  > - - " | =0=$- "+32H 57+ * 60 |
| !"#$#%:88%: | " 7 $ | "/> *  > - - " | =0=$- "+32H 57+ * 60 |
| !"#$#%:88'< | -E | "/> *  > - - " | =0=$- "+32H 57+ * 60 |
| !"#$#%:88'( | E$* " 7 $ | "/> *  > - - " | =0=$- "+32H 57+ * 60 |
| !"#$#%:88'; | 1,7 | "/> *  > - - " | =0=$- "+32H 57+ * 60 |
| !"#$#%:88') | ,17 | "/> *  > - - " | =0=$- "+32H 57+ * 60 |
| !"#$#%:88'8 | 067 " 7 $ | "/> *  > - - " | =0=$- "+32H 57+ * 60 |
| !"#$#%:88'% | E$* " 7 $ | "/> *  > - - " | =0=$- "+32H 57+ * 60 |

!"#$#%:88'9!"#$#%:88'&!"#$#%:88':!"#$#%:889<!"#$#%:889(!"#$#%:889;!"#$#%:889)!"#$#%:8898!"#$#%:889%!"#$#%:889'!"#$#%:8899!"#$#%:889&!"#$#%:889:!"#$#%:88&<!"#$#%:88&(!"#$#%:88&;!"#$#%:88&)

6>1,56J65 ->A(:52?-A52@- 0 * ,0,--, 6,+ /,B 0"!1,+$

7 J* 177 1 -, ,2* +0-E

!"#$#%:88&8!"#$#%:88&%!"#$#%:88&'!"#$#%:88&&!"#$#%:88&:!"#$#%:88:<!"#$#%:88:(!"#$#%:88:;!"#$#%:88:)!"#$#%:88:8!"#$#%:88:%!"#$#%:88:'!"#$#%:88:9!"#$#%:88:&!"#$#%:88::!"#$#%:8%<<!"#$#%:8%<;!"#$#%:8%<)!"#$#%:8%<8!"#$#%:8%<%!"#$#%:8%<'!"#$#%:8%<&!"#$#%:8%<:!"#$#%:8%(< !"#$#%:8%((!"#$#%:8%(;!"#$#%:8%()!"#$#%:8%(8!"#$#%:8%(%!"#$#%:8%('!"#$#%:8%(9!"#$#%:8%(&!"#$#%:8%(:!"#$#%:8%;(!"#$#%:8%;;!"#$#%:8%;)!"#$#%:8%;8!"#$#%:8%;%!"#$#%:8%;'!"#$#%:8%;9!"#$#%:8%;&!"#$#%:8%;:!"#$#%:8%)<!"#$#%:8%)(!"#$#%:8%);!"#$#%:8%))!"#$#%:8%)%!"#$#%:8%)' !"#$#%:8%)9!"#$#%:8%)&!"#$#%:8%):!"#$#%:8%8<!"#$#%:8%8(!"#$#%:8%8;!"#$#%:8%8)!"#$#%:8%88!"#$#%:8%8%!"#$#%:8%8'!"#$#%:8%89!"#$#%:8%8&!"#$#%:8%8:!"#$#%:8%%<!"#$#%:8%%(!"#$#%:8%%;!"#$#%:8%%)!"#$#%:8%%8!"#$#%:8%%%!"#$#%:8%%'!"#$#%:8%%9!"#$#%:8%%&!"#$#%:8%%:!"#$#%:8%'<

!"#$#%:8%';!"#$#%:8%')!"#$#%:8%'8!"#$#%:8%'%!"#$#%:8%''!"#$#%:8%'9!"#$#%:8%'&!"#$#%:8%':!"#$#%:8%9<!"#$#%:8%9(!"#$#%:8%9)!"#$#%:8%98!"#$#%:8%9%!"#$#%:8%9'!"#$#%:8%99!"#$#%:8%9&!"#$#%:8%9:!"#$#%:8%&<!"#$#%:8%&(!"#$#%:8%&;!"#$#%:8%&)!"#$#%:8%&8!"#$#%:8%&%!"#$#%:8%&'

!"#$#%:8%&9!"#$#%:8%&&!"#$#%:8%&:!"#$#%:8%:<!"#$#%:8%:(!"#$#%:8%:;!"#$#%:8%:)!"#$#%:8%:8!"#$#%:8%:%!"#$#%:8%:'!"#$#%:8%:9!"#$#%:8%:&!"#$#%:8%::!"#$#%:8'<<!"#$#%:8'<;!"#$#%:8'<)!"#$#%:8'<8!"#$#%:8'<%!"#$#%:8'<'!"#$#%:8'<9!"#$#%:8'<&!"#$#%:8'<:!"#$#%:8'(<!"#$#%:8'((

!"#$#%:8'(;!"#$#%:8'()!"#$#%:8'(8!"#$#%:8'(%!"#$#%:8'('

5* ->A(:52?-A52@- , 5*C- ,, + 1 / " /+H 1 $ 5*0$C- ,

# *- "62 " , 2 6/*"7!7 $C7 * H ! 47 C-

!"#$#%:8'(9!"#$#%:8'(&!"#$#%:8'(:!"#$#%:8';<!"#$#%:8';(!"#$#%:8';;!"#$#%:8';)!"#$#%:8';8!"#$#%:8';%!"#$#%:8';'!"#$#%:8';9!"#$#%:8';&!"#$#%:8';:!"#$#%:8')<!"#$#%:8')(!"#$#%:8');!"#$#%:8'))!"#$#%:8')8!"#$#%:8')%!"#$#%:8')'!"#$#%:8')9!"#$#%:8')&!"#$#%:8'):!"#$#%:8'8<

| !"#$#%:8'8(!"#$#%:8'8;!"#$#%:8'8)!"#$#%:8'88 |  |  |
| --- | --- | --- |
| 6>1,56J65  7 J* 177 | ->A(:52?-A52@- | 0 * ,0,--, 6,+ /,B 0"!1,+$ 1 -, ,2* +0-E |

!"#$#%:8'8%!"#$#%:8'8'!"#$#%:8'89!"#$#%:8'8&!"#$#%:8'8:!"#$#%:8'%<!"#$#%:8'%(!"#$#%:8'%;!"#$#%:8'%)!"#$#%:8'%8!"#$#%:8'%%!"#$#%:8'%'!"#$#%:8'%9!"#$#%:8'%&!"#$#%:8'%:!"#$#%:8''<!"#$#%:8''(!"#$#%:8'';!"#$#%:8'')!"#$#%:8''8!"#$#%:8''%!"#$#%:8'''!"#$#%:8''9!"#$#%:8''& !"#$#%:8'':!"#$#%:8'9<!"#$#%:8'9(!"#$#%:8'9;!"#$#%:8'9)!"#$#%:8'98!"#$#%:8'9%!"#$#%:8'9'!"#$#%:8'99!"#$#%:8'9&!"#$#%:8'9:!"#$#%:8'&<!"#$#%:8'&(!"#$#%:8'&;!"#$#%:8'&)!"#$#%:8'&8!"#$#%:8'&%!"#$#%:8'&'!"#$#%:8'&9!"#$#%:8'&&!"#$#%:8'&:!"#$#%:8':<!"#$#%:8':(!"#$#%:8':;

!"#$#%:8':)!"#$#%:8':8!"#$#%:8':%!"#$#%:8':'!"#$#%:8':9!"#$#%:8':&!"#$#%:8'::!"#$#%:89<<!"#$#%:89<(!"#$#%:89<;!"#$#%:89<)!"#$#%:89<8!"#$#%:89<%!"#$#%:89<'!"#$#%:89<9!"#$#%:89<&!"#$#%:89<:!"#$#%:89((!"#$#%:89(;!"#$#%:89()!"#$#%:89(8!"#$#%:89(%!"#$#%:89('!"#$#%:89(9

!"#$#%:89(&!"#$#%:89(:!"#$#%:89;<!"#$#%:89;(!"#$#%:89;;!"#$#%:89;)!"#$#%:89;8!"#$#%:89;%!"#$#%:89;'!"#$#%:89;9!"#$#%:89;&!"#$#%:89;:!"#$#%:89)<!"#$#%:89)(!"#$#%:89)8!"#$#%:89)%!"#$#%:89)'!"#$#%:89)9!"#$#%:89)&!"#$#%:89):!"#$#%:898<!"#$#%:898(!"#$#%:898;!"#$#%:898) !"#$#%:8988!"#$#%:898%!"#$#%:898'!"#$#%:898:!"#$#%:89%<!"#$#%:89%(!"#$#%:89%;!"#$#%:89%)!"#$#%:89%8!"#$#%:89%%!"#$#%:89%'!"#$#%:89%9!"#$#%:89%&!"#$#%:89%:!"#$#%:89'<!"#$#%:89'(!"#$#%:89';!"#$#%:89')!"#$#%:89'8!"#$#%:89'%!"#$#%:89''!"#$#%:89'9!"#$#%:89'&!"#$#%:89': !"#$#%:899<!"#$#%:899(!"#$#%:899;!"#$#%:899)!"#$#%:8998!"#$#%:899%!"#$#%:899'!"#$#%:8999!"#$#%:899&!"#$#%:899:!"#$#%:89&<!"#$#%:89&(!"#$#%:89&;!"#$#%:89&)!"#$#%:89&8!"#$#%:89&%!"#$#%:89&'!"#$#%:89&9!"#$#%:89&&!"#$#%:89&:!"#$#%:89:<!"#$#%:89:(!"#$#%:89:;!"#$#%:89:)

!"#$#%:89:8!"#$#%:89:%!"#$#%:89:'!"#$#%:89:9!"#$#%:89:&!"#$#%:89::!"#$#%:8&<<!"#$#%:8&<(!"#$#%:8&<;!"#$#%:8&<)!"#$#%:8&<8!"#$#%:8&<%!"#$#%:8&<'!"#$#%:8&<9!"#$#%:8&<&!"#$#%:8&<:!"#$#%:8&(<!"#$#%:8&((!"#$#%:8&(;!"#$#%:8&()!"#$#%:8&(8!"#$#%:8&(%

> -17-D,/-A5 ->A(:52?-A52@- E 1+2 ,- $ 0+1 ! A-2 1 J, ,1 "J/

-> / +7 > $/J ,$/ J2$ .1+ /.+7!0

!"#$#%:8&('!"#$#%:8&(9!"#$#%:8&(:!"#$#%:8&;<!"#$#%:8&;(!"#$#%:8&;;!"#$#%:8&;)!"#$#%:8&;8!"#$#%:8&;%!"#$#%:8&;'!"#$#%:8&;&!"#$#%:8&;:!"#$#%:8&)<!"#$#%:8&)(!"#$#%:8&);!"#$#%:8&))!"#$#%:8&)8!"#$#%:8&)%!"#$#%:8&)'!"#$#%:8&)9!"#$#%:8&)&!"#$#%:8&):!"#$#%:8&8<!"#$#%:8&8( !"#$#%:8&8;!"#$#%:8&8)!"#$#%:8&88!"#$#%:8&8%!"#$#%:8&8'!"#$#%:8&89

|  | > / ! 17$D  * 5! D , ,5! | ->A(:52?-A52@- | ,7 ,// ,* 4-Q0 R!7 >,-+0- B /= F+ */ 0-0 2 |
| --- | --- | --- | --- |
| !"#$#%:8&%(!"#$#%:8&'< | $ - $ | ->A(:52?-A52@- | 7 $ "+ 76 , 4, +*.! * - ++- - |

# /! LA , "2E /F7-7 ,7 , 4A++$$ +,

- 1 ! Q0 - . / "A/ $ /1 "/ 0/" 0 +"1 !>+ ,

!"#$#%:8&'(!"#$#%:8&';!"#$#%:8&')!"#$#%:8&'8!"#$#%:8&'%!"#$#%:8&'9!"#$#%:8&':!"#$#%:8&9<!"#$#%:8&9(!"#$#%:8&9;!"#$#%:8&9)!"#$#%:8&98!"#$#%:8&9%!"#$#%:8&9'!"#$#%:8&9&!"#$#%:8&9:!"#$#%:8&&(!"#$#%:8&&;!"#$#%:8&&8!"#$#%:8&&%!"#$#%:8&&'!"#$#%:8&&9

5- $7 - ->A(:52?-A52@- - / , 2" / /0* 7 0 " 1 " - $E="+B E

17E 0 - 7 170 + *27" / +" 7 + *

!"#$#%:8&&:!"#$#%:8&:<!"#$#%:8&:(!"#$#%:8&:;!"#$#%:8&:)!"#$#%:8&:8!"#$#%:8&:%!"#$#%:8&:'!"#$#%:8&:9!"#$#%:8&:&!"#$#%:8&::!"#$#%:8:<<!"#$#%:8:<(!"#$#%:8:<;!"#$#%:8:<)!"#$#%:8:<8!"#$#%:8:<%!"#$#%:8:<'!"#$#%:8:<9!"#$#%:8:<&!"#$#%:8:<:!"#$#%:8:(<!"#$#%:8:((!"#$#%:8:(;

!"#$#%:8:()!"#$#%:8:(8!"#$#%:8:(%!"#$#%:8:('!"#$#%:8:(9!"#$#%:8:(&!"#$#%:8:(:!"#$#%:8:;<!"#$#%:8:;(!"#$#%:8:;;

6>1,56J65 ->A(:52?-A52@- 0 * ,0,--, 6,+ /,B 0"!1,+$

7 J* 177 1 -, ,2* +0-E

!"#$#%:8:;)!"#$#%:8:;8!"#$#%:8:;'!"#$#%:8:;9!"#$#%:8:;&!"#$#%:8:;:!"#$#%:8:)<!"#$#%:8:)(!"#$#%:8:);!"#$#%:8:))!"#$#%:8:)8!"#$#%:8:)%!"#$#%:8:)'!"#$#%:8:)9!"#$#%:8:)&!"#$#%:8:):!"#$#%:8:8<!"#$#%:8:8(!"#$#%:8:8;!"#$#%:8:8)!"#$#%:8:88!"#$#%:8:8%!"#$#%:8:8'!"#$#%:8:89 !"#$#%:8:8&!"#$#%:8:8:!"#$#%:8:%<!"#$#%:8:%(!"#$#%:8:%;!"#$#%:8:%)!"#$#%:8:%8!"#$#%:8:%%!"#$#%:8:%'!"#$#%:8:%9!"#$#%:8:%&!"#$#%:8:%:!"#$#%:8:'<!"#$#%:8:'(!"#$#%:8:';!"#$#%:8:')!"#$#%:8:'8!"#$#%:8:'%!"#$#%:8:''!"#$#%:8:'9!"#$#%:8:'&!"#$#%:8:':!"#$#%:8:9<!"#$#%:8:9(

!"#$#%:8:9;!"#$#%:8:9)!"#$#%:8:98!"#$#%:8:9%!"#$#%:8:9'!"#$#%:8:99!"#$#%:8:9&!"#$#%:8:9:!"#$#%:8:&<!"#$#%:8:&(!"#$#%:8:&)!"#$#%:8:&8!"#$#%:8:&%!"#$#%:8:&'

H , -- , DB ->A(:52?-A52@- - ,.2 > ,E , ,$77A +* " ,- +- 00 17 ,- -

1 ,E+>,$

!"#$#%:8:&9!"#$#%:8:&&!"#$#%:8:&:!"#$#%:8::<!"#$#%:8::(!"#$#%:8::;!"#$#%:8::)!"#$#%:8::8!"#$#%:8::%!"#$#%:8::9!"#$#%:8::&!"#$#%:8:::!"#$#%:%<<<!"#$#%:%<<(!"#$#%:%<<;!"#$#%:%<<)!"#$#%:%<<8!"#$#%:%<<%!"#$#%:%<<'!"#$#%:%<<&!"#$#%:%<<:!"#$#%:%<(<!"#$#%:%<((!"#$#%:%<(; !"#$#%:%<(8!"#$#%:%<('!"#$#%:%<(9!"#$#%:%<(:!"#$#%:%<;<!"#$#%:%<;(!"#$#%:%<;)!"#$#%:%<;%!"#$#%:%<;'!"#$#%:%<;9!"#$#%:%<;:!"#$#%:%<)<!"#$#%:%<)(!"#$#%:%<);!"#$#%:%<))!"#$#%:%<)8!"#$#%:%<)%!"#$#%:%<)'!"#$#%:%<)9!"#$#%:%<)&!"#$#%:%<):!"#$#%:%<8(!"#$#%:%<8;!"#$#%:%<8)

!"#$#%:%<88!"#$#%:%<8%

1 5D 07 17E 0 D ->A(:52?-A52@- $1 ,*/= + $+ ,0B 7 * -=$" * $!6+

1- -17E 0 D10 "- !*! *"+- = 0!

7 17E 0 D1 7 17E

0 !"#$#%:%<8'!"#$#%:%<89!"#$#%:%<8&!"#$#%:%<8:!"#$#%:%<%<!"#$#%:%<%(!"#$#%:%<%;!"#$#%:%<%)!"#$#%:%<%8!"#$#%:%<%%!"#$#%:%<%'!"#$#%:%<%&!"#$#%:%<%:!"#$#%:%<'<!"#$#%:%<'(!"#$#%:%<';!"#$#%:%<')!"#$#%:%<'8!"#$#%:%<'%!"#$#%:%<''!"#$#%:%<'9!"#$#%:%<'&!"#$#%:%<':!"#$#%:%<9< !"#$#%:%<9(!"#$#%:%<9;!"#$#%:%<9)!"#$#%:%<98!"#$#%:%<9%!"#$#%:%<9'!"#$#%:%<99!"#$#%:%<9&!"#$#%:%<9:!"#$#%:%<&<!"#$#%:%<&(!"#$#%:%<&;!"#$#%:%<&)!"#$#%:%<&8!"#$#%:%<&%!"#$#%:%<&'!"#$#%:%<&9!"#$#%:%<&&!"#$#%:%<&:!"#$#%:%<:<!"#$#%:%<:(!"#$#%:%<:;!"#$#%:%<:)!"#$#%:%<:8 !"#$#%:%<:%!"#$#%:%<:'!"#$#%:%<:&!"#$#%:%<::

H , -- , DB ->A(:52?-A52@- - ,.2 > ,E , ,$77A +* " ,- +- 00 17 ,- -

1 ,E+>,$

!"#$#%:%(<<!"#$#%:%(<(!"#$#%:%(<;!"#$#%:%(<8!"#$#%:%(<%!"#$#%:%(<'!"#$#%:%(<9!"#$#%:%(<&!"#$#%:%(<:!"#$#%:%((<!"#$#%:%(((!"#$#%:%((;!"#$#%:%(()!"#$#%:%((8

-!45" D0 ->A(:52?-A52@- * =* ! 2 * / $ !* - / /$" 7 170

!"#$#%:%((%!"#$#%:%(('!"#$#%:%((9!"#$#%:%((&!"#$#%:%((:!"#$#%:%(;<!"#$#%:%(;(!"#$#%:%(;;!"#$#%:%(;)!"#$#%:%(;8!"#$#%:%(;%!"#$#%:%(;'!"#$#%:%(;9!"#$#%:%(;&!"#$#%:%(;:!"#$#%:%()<!"#$#%:%()(!"#$#%:%();!"#$#%:%())!"#$#%:%()8!"#$#%:%()%!"#$#%:%()'!"#$#%:%()9!"#$#%:%()& !"#$#%:%():!"#$#%:%(8<!"#$#%:%(8(!"#$#%:%(8;!"#$#%:%(8)!"#$#%:%(88!"#$#%:%(8%!"#$#%:%(8'!"#$#%:%(89!"#$#%:%(8&!"#$#%:%(8:!"#$#%:%(%<!"#$#%:%(%(!"#$#%:%(%;!"#$#%:%(%)!"#$#%:%(%8!"#$#%:%(%%!"#$#%:%(%'!"#$#%:%(%9!"#$#%:%(%&!"#$#%:%(%:!"#$#%:%('<!"#$#%:%('(!"#$#%:%(';

!"#$#%:%(')!"#$#%:%('8!"#$#%:%('%!"#$#%:%('9!"#$#%:%('&!"#$#%:%(':!"#$#%:%(9<!"#$#%:%(9(!"#$#%:%(9;!"#$#%:%(9)!"#$#%:%(98!"#$#%:%(9%!"#$#%:%(9'!"#$#%:%(99!"#$#%:%(9&!"#$#%:%(9:!"#$#%:%(&<!"#$#%:%(&;!"#$#%:%(&)!"#$#%:%(&8!"#$#%:%(&%!"#$#%:%(&'!"#$#%:%(&9!"#$#%:%(&:

!"#$#%:%(:<!"#$#%:%(:(!"#$#%:%(:;!"#$#%:%(:)!"#$#%:%(:8!"#$#%:%(:'!"#$#%:%(:9!"#$#%:%(:&!"#$#%:%(::!"#$#%:%;<<!"#$#%:%;<(!"#$#%:%;<;!"#$#%:%;<)!"#$#%:%;<8!"#$#%:%;<%!"#$#%:%;<'!"#$#%:%;<9!"#$#%:%;<&!"#$#%:%;<:!"#$#%:%;(<!"#$#%:%;(;!"#$#%:%;()!"#$#%:%;(8!"#$#%:%;(%

!"#$#%:%;('!"#$#%:%;(9!"#$#%:%;(&!"#$#%:%;(:!"#$#%:%;;<!"#$#%:%;;(!"#$#%:%;;;!"#$#%:%;;8!"#$#%:%;;%!"#$#%:%;;'!"#$#%:%;;9!"#$#%:%;;&!"#$#%:%;;:!"#$#%:%;)<!"#$#%:%;)(

H * ->A(:52?-A52@- + * $ 2 0$A>/ " 02 ,4$ ,1 AE $44,/ 14!

, =1 , 0 6+0/ * - * /" ! ,+, + "+ Q

!"#$#%:%;);!"#$#%:%;))!"#$#%:%;)8!"#$#%:%;)%!"#$#%:%;)'!"#$#%:%;)9!"#$#%:%;)&!"#$#%:%;):!"#$#%:%;8<!"#$#%:%;8(!"#$#%:%;8;!"#$#%:%;8)!"#$#%:%;88!"#$#%:%;8%!"#$#%:%;8'!"#$#%:%;89!"#$#%:%;8&!"#$#%:%;8:!"#$#%:%;%<!"#$#%:%;%(!"#$#%:%;%;!"#$#%:%;%)!"#$#%:%;%8!"#$#%:%;%% !"#$#%:%;%'!"#$#%:%;%9!"#$#%:%;%&!"#$#%:%;%:!"#$#%:%;'<!"#$#%:%;'(!"#$#%:%;';!"#$#%:%;')!"#$#%:%;'8!"#$#%:%;'%!"#$#%:%;''!"#$#%:%;'9!"#$#%:%;'&!"#$#%:%;':!"#$#%:%;9<!"#$#%:%;9(!"#$#%:%;9;!"#$#%:%;9)!"#$#%:%;98!"#$#%:%;9%!"#$#%:%;9'!"#$#%:%;99!"#$#%:%;9&!"#$#%:%;9: !"#$#%:%;&<!"#$#%:%;&(!"#$#%:%;&;!"#$#%:%;&)!"#$#%:%;&8!"#$#%:%;&%!"#$#%:%;&'!"#$#%:%;&9!"#$#%:%;&&!"#$#%:%;&:!"#$#%:%;:<!"#$#%:%;:(!"#$#%:%;:;!"#$#%:%;:)!"#$#%:%;:8!"#$#%:%;:%!"#$#%:%;:'!"#$#%:%;:9!"#$#%:%;:&!"#$#%:%;::!"#$#%:%)<<!"#$#%:%)<(!"#$#%:%)<;!"#$#%:%)<) !"#$#%:%)<8!"#$#%:%)<%!"#$#%:%)<'!"#$#%:%)<9!"#$#%:%)<&!"#$#%:%)<:!"#$#%:%)(<!"#$#%:%)((!"#$#%:%)(;!"#$#%:%)()!"#$#%:%)(8!"#$#%:%)(%!"#$#%:%)('!"#$#%:%)(9!"#$#%:%)(&!"#$#%:%)(:!"#$#%:%);<!"#$#%:%);(!"#$#%:%);;!"#$#%:%);)!"#$#%:%);8!"#$#%:%);%

|  | H , -- , DB  1 | ->A(:52?-A52@- | - ,.2 > ,E , ,$77A +* " ,- +- 00 17 ,- - ,E+>,$ |
| --- | --- | --- | --- |
| !"#$#%:%);'!"#$#%:%);9!"#$#%:%);& | 6>1,56J657 J* 177 | ->A(:52?-A52@- | 0 * ,0,--, 6,+ /,B 0"!1,+$ 1 -, ,2* +0-E |

!"#$#%:%);:!"#$#%:%))<!"#$#%:%))(!"#$#%:%));!"#$#%:%)))!"#$#%:%))8!"#$#%:%))%!"#$#%:%))'!"#$#%:%))9!"#$#%:%))&!"#$#%:%)):!"#$#%:%)8<!"#$#%:%)8(!"#$#%:%)8;!"#$#%:%)8)!"#$#%:%)88!"#$#%:%)8%!"#$#%:%)8'!"#$#%:%)89!"#$#%:%)8&!"#$#%:%)8:!"#$#%:%)%<!"#$#%:%)%(!"#$#%:%)%; !"#$#%:%)%)!"#$#%:%)%8!"#$#%:%)%%!"#$#%:%)%'!"#$#%:%)%9!"#$#%:%)%&!"#$#%:%)%:!"#$#%:%)'<!"#$#%:%)'(!"#$#%:%)')!"#$#%:%)'8!"#$#%:%)'%!"#$#%:%)''!"#$#%:%)'9!"#$#%:%)'&!"#$#%:%)':!"#$#%:%)9<!"#$#%:%)9(!"#$#%:%)9;!"#$#%:%)9)!"#$#%:%)98!"#$#%:%)9%!"#$#%:%)9'!"#$#%:%)99 !"#$#%:%)9&!"#$#%:%)&<!"#$#%:%)&(!"#$#%:%)&;!"#$#%:%)&)!"#$#%:%)&8!"#$#%:%)&%!"#$#%:%)&'!"#$#%:%)&9!"#$#%:%)&&!"#$#%:%)&:!"#$#%:%):<!"#$#%:%):(!"#$#%:%):;!"#$#%:%):)!"#$#%:%):8!"#$#%:%):'!"#$#%:%):9!"#$#%:%):&!"#$#%:%)::!"#$#%:%8<<!"#$#%:%8<(!"#$#%:%8<;!"#$#%:%8<)

!"#$#%:%8<8!"#$#%:%8<%!"#$#%:%8<'!"#$#%:%8<9!"#$#%:%8<&!"#$#%:%8<:!"#$#%:%8(<!"#$#%:%8((!"#$#%:%8(;!"#$#%:%8()!"#$#%:%8(8!"#$#%:%8(%!"#$#%:%8('!"#$#%:%8(9!"#$#%:%8(&!"#$#%:%8(:!"#$#%:%8;<!"#$#%:%8;(!"#$#%:%8;;!"#$#%:%8;)!"#$#%:%8;8!"#$#%:%8;%!"#$#%:%8;'!"#$#%:%8;9

!"#$#%:%8;&!"#$#%:%8;:!"#$#%:%8)<!"#$#%:%8)(!"#$#%:%8);!"#$#%:%8))!"#$#%:%8)8!"#$#%:%8)%!"#$#%:%8)'!"#$#%:%8)9!"#$#%:%8)&!"#$#%:%8):!"#$#%:%88<!"#$#%:%88(!"#$#%:%88)!"#$#%:%888!"#$#%:%88%!"#$#%:%88'!"#$#%:%889!"#$#%:%88&!"#$#%:%88:!"#$#%:%8%<!"#$#%:%8%(!"#$#%:%8%; !"#$#%:%8%)!"#$#%:%8%8!"#$#%:%8%%!"#$#%:%8%9!"#$#%:%8%&!"#$#%:%8%:!"#$#%:%8'<!"#$#%:%8'(!"#$#%:%8';!"#$#%:%8')!"#$#%:%8'8!"#$#%:%8'%!"#$#%:%8''!"#$#%:%8'9!"#$#%:%8'&!"#$#%:%8':!"#$#%:%89<!"#$#%:%89(!"#$#%:%89;!"#$#%:%89)!"#$#%:%898!"#$#%:%89%!"#$#%:%89'!"#$#%:%899

!"#$#%:%89&!"#$#%:%8&<!"#$#%:%8&(!"#$#%:%8&;!"#$#%:%8&)!"#$#%:%8&8!"#$#%:%8&%!"#$#%:%8&'!"#$#%:%8&9!"#$#%:%8&&!"#$#%:%8&:!"#$#%:%8:<!"#$#%:%8:(!"#$#%:%8:;!"#$#%:%8:)!"#$#%:%8:8!"#$#%:%8:%!"#$#%:%8:'!"#$#%:%8:9!"#$#%:%8:&!"#$#%:%%<<!"#$#%:%%<(!"#$#%:%%<;!"#$#%:%%<8

!"#$#%:%%<%!"#$#%:%%<'!"#$#%:%%<9!"#$#%:%%<&!"#$#%:%%<:!"#$#%:%%(<!"#$#%:%%((!"#$#%:%%(;!"#$#%:%%()!"#$#%:%%(8!"#$#%:%%(%!"#$#%:%%('!"#$#%:%%(9!"#$#%:%%(&!"#$#%:%%(:!"#$#%:%%;<!"#$#%:%%;;!"#$#%:%%;)!"#$#%:%%;8!"#$#%:%%;%!"#$#%:%%;'!"#$#%:%%;9!"#$#%:%%;&!"#$#%:%%;:

!"#$#%:%%)<!"#$#%:%%)(!"#$#%:%%);!"#$#%:%%))!"#$#%:%%)8!"#$#%:%%)%!"#$#%:%%)'!"#$#%:%%)9!"#$#%:%%)&!"#$#%:%%):!"#$#%:%%8<!"#$#%:%%8(!"#$#%:%%8;!"#$#%:%%8)!"#$#%:%%88!"#$#%:%%8%!"#$#%:%%8'!"#$#%:%%89!"#$#%:%%8&!"#$#%:%%8:!"#$#%:%%%<!"#$#%:%%%(!"#$#%:%%%;!"#$#%:%%%)

!"#$#%:%%%8!"#$#%:%%%%!"#$#%:%%%'!"#$#%:%%%9!"#$#%:%%%&!"#$#%:%%%:!"#$#%:%%'<!"#$#%:%%'(!"#$#%:%%';!"#$#%:%%')!"#$#%:%%'8!"#$#%:%%'%!"#$#%:%%''!"#$#%:%%'9!"#$#%:%%'&!"#$#%:%%':!"#$#%:%%9<!"#$#%:%%9(!"#$#%:%%9;!"#$#%:%%9)!"#$#%:%%98!"#$#%:%%9%!"#$#%:%%9'!"#$#%:%%99

!"#$#%:%%9&!"#$#%:%%9:!"#$#%:%%&<!"#$#%:%%&(!"#$#%:%%&;!"#$#%:%%&)!"#$#%:%%&8!"#$#%:%%&%!"#$#%:%%&'!"#$#%:%%&9!"#$#%:%%&&!"#$#%:%%&:!"#$#%:%%:<!"#$#%:%%:(!"#$#%:%%:;!"#$#%:%%:)!"#$#%:%%:8!"#$#%:%%:%!"#$#%:%%:'!"#$#%:%%:9!"#$#%:%%:&!"#$#%:%%::!"#$#%:%'<<!"#$#%:%'<(

!"#$#%:%'<;!"#$#%:%'<)!"#$#%:%'<8!"#$#%:%'<%!"#$#%:%'<'!"#$#%:%'<9!"#$#%:%'<&!"#$#%:%'<:!"#$#%:%'(<!"#$#%:%'((!"#$#%:%'(;!"#$#%:%'()!"#$#%:%'(8!"#$#%:%'(%!"#$#%:%'('

C > -B C ->A(:52?-A52@- -,+!$ , "- , * 6 *A 6+-+ $ $

"5 1 "2 4A 0 +/,* + -0-

!"#$#%:%'(9!"#$#%:%'(&!"#$#%:%'(:!"#$#%:%';<!"#$#%:%';(!"#$#%:%';;!"#$#%:%';)!"#$#%:%';8!"#$#%:%';%!"#$#%:%';'!"#$#%:%';9!"#$#%:%';&!"#$#%:%';:!"#$#%:%')<!"#$#%:%')(!"#$#%:%');!"#$#%:%'))!"#$#%:%')8!"#$#%:%')%!"#$#%:%')'!"#$#%:%')9!"#$#%:%')&!"#$#%:%'):!"#$#%:%'8< !"#$#%:%'8(!"#$#%:%'8;!"#$#%:%'8)!"#$#%:%'88!"#$#%:%'8%!"#$#%:%'8'!"#$#%:%'89!"#$#%:%'8&!"#$#%:%'8:!"#$#%:%'%<!"#$#%:%'%(!"#$#%:%'%;!"#$#%:%'%)!"#$#%:%'%8!"#$#%:%'%%!"#$#%:%'%'!"#$#%:%'%9!"#$#%:%'%&!"#$#%:%'%:!"#$#%:%''<!"#$#%:%''(!"#$#%:%'';!"#$#%:%'')!"#$#%:%''8

!"#$#%:%''%!"#$#%:%'''!"#$#%:%''9!"#$#%:%''&!"#$#%:%'':!"#$#%:%'9<!"#$#%:%'9(!"#$#%:%'9;!"#$#%:%'9)!"#$#%:%'98!"#$#%:%'9%!"#$#%:%'9'!"#$#%:%'99!"#$#%:%'9&!"#$#%:%'9:!"#$#%:%'&<!"#$#%:%'&(!"#$#%:%'&;!"#$#%:%'&)!"#$#%:%'&8!"#$#%:%'&%!"#$#%:%'&'!"#$#%:%'&9!"#$#%:%'&& !"#$#%:%'&:!"#$#%:%':<!"#$#%:%':(!"#$#%:%':;!"#$#%:%':)!"#$#%:%':8!"#$#%:%':%!"#$#%:%':'!"#$#%:%':9!"#$#%:%':&!"#$#%:%'::!"#$#%:%9<<!"#$#%:%9<(!"#$#%:%9<;!"#$#%:%9<)!"#$#%:%9<8!"#$#%:%9<%!"#$#%:%9<'!"#$#%:%9<9!"#$#%:%9<&!"#$#%:%9<:!"#$#%:%9(<!"#$#%:%9((!"#$#%:%9(; !"#$#%:%9()!"#$#%:%9(8!"#$#%:%9(%!"#$#%:%9('!"#$#%:%9(9!"#$#%:%9(&!"#$#%:%9(:!"#$#%:%9;<!"#$#%:%9;(!"#$#%:%9;;!"#$#%:%9;)!"#$#%:%9;8!"#$#%:%9;%!"#$#%:%9;'!"#$#%:%9;9!"#$#%:%9;&!"#$#%:%9;:!"#$#%:%9)<!"#$#%:%9)(!"#$#%:%9);!"#$#%:%9))!"#$#%:%9)8!"#$#%:%9)%!"#$#%:%9)'

!"#$#%:%9)9!"#$#%:%9)&!"#$#%:%9):!"#$#%:%98<!"#$#%:%98(!"#$#%:%98;!"#$#%:%98)!"#$#%:%988!"#$#%:%98%!"#$#%:%98'!"#$#%:%989!"#$#%:%98&!"#$#%:%98:!"#$#%:%9%<!"#$#%:%9%(!"#$#%:%9%;!"#$#%:%9%)!"#$#%:%9%8!"#$#%:%9%%!"#$#%:%9%'!"#$#%:%9%9!"#$#%:%9%&!"#$#%:%9%:!"#$#%:%9'<

!"#$#%:%9'(!"#$#%:%9';!"#$#%:%9')!"#$#%:%9'8!"#$#%:%9'%!"#$#%:%9''!"#$#%:%9'9!"#$#%:%9'&!"#$#%:%9':!"#$#%:%99<!"#$#%:%99(!"#$#%:%99;!"#$#%:%99)!"#$#%:%998!"#$#%:%99%!"#$#%:%99'!"#$#%:%999!"#$#%:%99&!"#$#%:%99:!"#$#%:%9&<!"#$#%:%9&(!"#$#%:%9&;!"#$#%:%9&)!"#$#%:%9&8

!"#$#%:%9&%!"#$#%:%9&'!"#$#%:%9&9!"#$#%:%9&&!"#$#%:%9&:!"#$#%:%9:<!"#$#%:%9:(!"#$#%:%9:;!"#$#%:%9:)!"#$#%:%9:8!"#$#%:%9:%!"#$#%:%9:'!"#$#%:%9:9!"#$#%:%9:&

6>1,56J65 ->A(:52?-A52@- 0 * ,0,--, 6,+ /,B 0"!1,+$

7 J* 177 1 -, ,2* +0-E

!"#$#%:%&<;!"#$#%:%&<)!"#$#%:%&<8!"#$#%:%&<%!"#$#%:%&<9!"#$#%:%&<&!"#$#%:%&<:!"#$#%:%&(<!"#$#%:%&((!"#$#%:%&(;!"#$#%:%&()!"#$#%:%&(8!"#$#%:%&(%!"#$#%:%&('!"#$#%:%&(9!"#$#%:%&(&!"#$#%:%&(:!"#$#%:%&;<!"#$#%:%&;(!"#$#%:%&;)!"#$#%:%&;8!"#$#%:%&;%!"#$#%:%&;'!"#$#%:%&;&

!"#$#%:%&;:!"#$#%:%&)<!"#$#%:%&)(!"#$#%:%&);!"#$#%:%&))!"#$#%:%&)8!"#$#%:%&)%!"#$#%:%&)'

> 07 17E ->A(:52?-A52@- 0 ," 1 /*$ / 0 " " 2+$ - 62 ", *.

0 D - $,= 4,/4-!

0, 5

!"#$#%:%&)9!"#$#%:%&)&!"#$#%:%&):!"#$#%:%&8<!"#$#%:%&8(!"#$#%:%&8)!"#$#%:%&88!"#$#%:%&8%!"#$#%:%&8'!"#$#%:%&89!"#$#%:%&8&!"#$#%:%&8:!"#$#%:%&%(!"#$#%:%&%;!"#$#%:%&%)!"#$#%:%&%8!"#$#%:%&%%!"#$#%:%&%'!"#$#%:%&%9!"#$#%:%&%&!"#$#%:%&%:!"#$#%:%&'<!"#$#%:%&'(!"#$#%:%&'; !"#$#%:%&')!"#$#%:%&'8!"#$#%:%&'%!"#$#%:%&''!"#$#%:%&'9!"#$#%:%&'&!"#$#%:%&':!"#$#%:%&9<!"#$#%:%&9(!"#$#%:%&9;!"#$#%:%&9%!"#$#%:%&9'!"#$#%:%&99!"#$#%:%&9&!"#$#%:%&9:!"#$#%:%&&<!"#$#%:%&&;!"#$#%:%&&)!"#$#%:%&&8!"#$#%:%&&%!"#$#%:%&&'!"#$#%:%&&9!"#$#%:%&&&!"#$#%:%&&:

!"#$#%:%&:<!"#$#%:%&:(!"#$#%:%&:;!"#$#%:%&:)!"#$#%:%&:8!"#$#%:%&:%!"#$#%:%&:'!"#$#%:%&:9!"#$#%:%&:&!"#$#%:%&::!"#$#%:%:<<!"#$#%:%:<(!"#$#%:%:<;!"#$#%:%:<)!"#$#%:%:<8!"#$#%:%:<%!"#$#%:%:<'!"#$#%:%:<9!"#$#%:%:<&!"#$#%:%:<:!"#$#%:%:(<!"#$#%:%:((!"#$#%:%:(;!"#$#%:%:()

!"#$#%:%:(8!"#$#%:%:(%!"#$#%:%:('!"#$#%:%:(9!"#$#%:%:(&!"#$#%:%:(:!"#$#%:%:;<!"#$#%:%:;;!"#$#%:%:;8!"#$#%:%:;%!"#$#%:%:;'!"#$#%:%:;9!"#$#%:%:;&!"#$#%:%:;:!"#$#%:%:)<!"#$#%:%:)(!"#$#%:%:);!"#$#%:%:)8!"#$#%:%:)%!"#$#%:%:)'!"#$#%:%:)9!"#$#%:%:)&!"#$#%:%:8<!"#$#%:%:8(

!"#$#%:%:8;!"#$#%:%:8)!"#$#%:%:88!"#$#%:%:8%!"#$#%:%:8'!"#$#%:%:89!"#$#%:%:8&!"#$#%:%:%<!"#$#%:%:%(!"#$#%:%:%;!"#$#%:%:%)!"#$#%:%:%8!"#$#%:%:%'!"#$#%:%:%9!"#$#%:%:%&!"#$#%:%:%:!"#$#%:%:'<!"#$#%:%:'(!"#$#%:%:';!"#$#%:%:')!"#$#%:%:'8!"#$#%:%:'%!"#$#%:%:''!"#$#%:%:'9

!"#$#%:%:'&!"#$#%:%:':!"#$#%:%:9<!"#$#%:%:9(!"#$#%:%:9;!"#$#%:%:9)!"#$#%:%:98!"#$#%:%:9%!"#$#%:%:9'!"#$#%:%:99!"#$#%:%:9&!"#$#%:%:9:!"#$#%:%:&<!"#$#%:%:&(!"#$#%:%:&;!"#$#%:%:&)!"#$#%:%:&8!"#$#%:%:&%!"#$#%:%:&'!"#$#%:%:&9!"#$#%:%:&&!"#$#%:%:&:!"#$#%:%::<!"#$#%:%::( !"#$#%:%::;!"#$#%:%::)!"#$#%:%::8!"#$#%:%::%!"#$#%:%::'!"#$#%:%::9!"#$#%:%::&!"#$#%:%:::!"#$#%:'<<<!"#$#%:'<<(!"#$#%:'<<;!"#$#%:'<<)!"#$#%:'<<8!"#$#%:'<<%!"#$#%:'<<'!"#$#%:'<<9!"#$#%:'<<&!"#$#%:'<<:!"#$#%:'<(<!"#$#%:'<((!"#$#%:'<(;!"#$#%:'<(8!"#$#%:'<('!"#$#%:'<(9 !"#$#%:'<(&!"#$#%:'<(:!"#$#%:'<;<!"#$#%:'<;(!"#$#%:'<;;!"#$#%:'<;)!"#$#%:'<;8!"#$#%:'<;%!"#$#%:'<;'!"#$#%:'<;9!"#$#%:'<;&!"#$#%:'<;:!"#$#%:'<)<!"#$#%:'<)(!"#$#%:'<);!"#$#%:'<))!"#$#%:'<)8!"#$#%:'<)%!"#$#%:'<)'!"#$#%:'<)9!"#$#%:'<)&!"#$#%:'<):!"#$#%:'<8<!"#$#%:'<8(

!"#$#%:'<8;!"#$#%:'<8)!"#$#%:'<88!"#$#%:'<8%!"#$#%:'<8'!"#$#%:'<89!"#$#%:'<8&!"#$#%:'<8:!"#$#%:'<%<!"#$#%:'<%(!"#$#%:'<%;!"#$#%:'<%)!"#$#%:'<%8!"#$#%:'<%'!"#$#%:'<%9!"#$#%:'<%&!"#$#%:'<%:!"#$#%:'<'<!"#$#%:'<';!"#$#%:'<')!"#$#%:'<'8!"#$#%:'<'%!"#$#%:'<'9!"#$#%:'<'&

!"#$#%:'<':!"#$#%:'<9<!"#$#%:'<9(!"#$#%:'<9;!"#$#%:'<9)!"#$#%:'<98!"#$#%:'<9%!"#$#%:'<9'!"#$#%:'<99!"#$#%:'<9&!"#$#%:'<9:!"#$#%:'<&<!"#$#%:'<&(!"#$#%:'<&;!"#$#%:'<&)!"#$#%:'<&8!"#$#%:'<&%!"#$#%:'<&9!"#$#%:'<&&!"#$#%:'<&:!"#$#%:'<:<!"#$#%:'<:(!"#$#%:'<:;!"#$#%:'<:)

!"#$#%:'<:8!"#$#%:'<:%!"#$#%:'<:'!"#$#%:'<:9!"#$#%:'<::!"#$#%:'(<<!"#$#%:'(<(!"#$#%:'(<;!"#$#%:'(<)!"#$#%:'(<8!"#$#%:'(<%!"#$#%:'(<'!"#$#%:'(<9!"#$#%:'(<&!"#$#%:'(<:!"#$#%:'((<!"#$#%:'(((!"#$#%:'((;!"#$#%:'(()!"#$#%:'((8!"#$#%:'((%!"#$#%:'(('!"#$#%:'((9!"#$#%:'((& !"#$#%:'((:!"#$#%:'(;<!"#$#%:'(;(!"#$#%:'(;;!"#$#%:'(;)!"#$#%:'(;8!"#$#%:'(;%!"#$#%:'(;'!"#$#%:'(;9!"#$#%:'(;&!"#$#%:'(;:!"#$#%:'()<!"#$#%:'()(!"#$#%:'();!"#$#%:'())!"#$#%:'()8!"#$#%:'()%!"#$#%:'()'!"#$#%:'()9!"#$#%:'()&!"#$#%:'():!"#$#%:'(8<!"#$#%:'(8(!"#$#%:'(8;

!"#$#%:'(8)!"#$#%:'(88!"#$#%:'(8%!"#$#%:'(8'!"#$#%:'(89!"#$#%:'(8:!"#$#%:'(%<!"#$#%:'(%(!"#$#%:'(%;!"#$#%:'(%)!"#$#%:'(%8!"#$#%:'(%%!"#$#%:'(%'!"#$#%:'(%9!"#$#%:'(%:!"#$#%:'('<!"#$#%:'('(!"#$#%:'(';!"#$#%:'(')!"#$#%:'('8!"#$#%:'('%!"#$#%:'(''!"#$#%:'('9!"#$#%:'('&

!"#$#%:'(':!"#$#%:'(9<!"#$#%:'(9(!"#$#%:'(9;!"#$#%:'(9)!"#$#%:'(98!"#$#%:'(9'!"#$#%:'(99!"#$#%:'(9&!"#$#%:'(9:!"#$#%:'(&<!"#$#%:'(&(!"#$#%:'(&;!"#$#%:'(&)!"#$#%:'(&8!"#$#%:'(&%!"#$#%:'(&'!"#$#%:'(&&!"#$#%:'(&:!"#$#%:'(:<

H * ->A(:52?-A52@- + * $ 2 0$A>/ " 02 ,4$ ,1 AE $44,/ 14!

, =1 , 0 6+0/ * - * /" ! ,+, + "+ Q

!"#$#%:'(:(!"#$#%:'(:;!"#$#%:'(:)!"#$#%:'(:8!"#$#%:'(:%!"#$#%:'(:'!"#$#%:'(:9!"#$#%:'(:&!"#$#%:'(::!"#$#%:';<<!"#$#%:';<;!"#$#%:';<)!"#$#%:';<8!"#$#%:';<%!"#$#%:';<'!"#$#%:';<9!"#$#%:';<&!"#$#%:';<:!"#$#%:';(<!"#$#%:';((!"#$#%:';(;!"#$#%:';()!"#$#%:';(8!"#$#%:';(%

!"#$#%:';('!"#$#%:';(&!"#$#%:';(:!"#$#%:';;<!"#$#%:';;(!"#$#%:';;;!"#$#%:';;)!"#$#%:';;8!"#$#%:';;%!"#$#%:';;'!"#$#%:';;9

|  | > 07 17E  0 D - 0, 5 | ->A(:52?-A52@- | 0 ," 1 /*$ / 0 " " 2+$ - 62 ", *. $,= 4,/4-! |
| --- | --- | --- | --- |
| !"#$#%:';;&!"#$#%:';;:!"#$#%:';)<  !"#$#%:';)( | 71 4-/ E | 71 4-/  E | 2E22*4 |
| !"#$#%:';); | 71 4-/ E | 71 4-/  E | 2E,22*4 |

!"#$#%:';))!"#$#%:';)8!"#$#%:';)%!"#$#%:';)'!"#$#%:';)9!"#$#%:';)&!"#$#%:';):!"#$#%:';8<!"#$#%:';8(!"#$#%:';8;!"#$#%:';8)!"#$#%:';88!"#$#%:';8%!"#$#%:';8'!"#$#%:';89!"#$#%:';8&!"#$#%:';8:

71 4-/ E 71 4-/ 2E22*4

E

!"#$#%:';%<!"#$#%:';%(!"#$#%:';%;!"#$#%:';%)!"#$#%:';%8!"#$#%:';%%!"#$#%:';%'!"#$#%:';%9!"#$#%:';%&!"#$#%:';%:!"#$#%:';'<!"#$#%:';'(!"#$#%:';';!"#$#%:';')

|  | 7!$F$-- | 71 4-/  E | 2E22*4 |
| --- | --- | --- | --- |
| !"#$#%:';'8!"#$#%:';'% | 71 4-/ E | 71 4-/  E | 2E,22*4 |
| !"#$#%:';''!"#$#%:';'9 | 71 4-/ E | 71 4-/  E | 2E22*4 |

!"#$#%:';'&!"#$#%:';':!"#$#%:';9<!"#$#%:';9(!"#$#%:';9;!"#$#%:';9)!"#$#%:';98!"#$#%:';9%!"#$#%:';9'!"#$#%:';99!"#$#%:';9&!"#$#%:';9:!"#$#%:';&<!"#$#%:';&(!"#$#%:';&;!"#$#%:';&)!"#$#%:';&8!"#$#%:';&%!"#$#%:';&'!"#$#%:';&9!"#$#%:';&&!"#$#%:';&:!"#$#%:';:<!"#$#%:';:( !"#$#%:';:;!"#$#%:';:)!"#$#%:';:8!"#$#%:';:%!"#$#%:';:'!"#$#%:';:9!"#$#%:';:&!"#$#%:';::!"#$#%:')<<!"#$#%:')<(!"#$#%:')<;!"#$#%:')<)!"#$#%:')<8!"#$#%:')<%!"#$#%:')<'!"#$#%:')<9!"#$#%:')<&!"#$#%:')<:!"#$#%:')(<!"#$#%:')((!"#$#%:')(;!"#$#%:')()!"#$#%:')(8!"#$#%:')(%

!"#$#%:')('!"#$#%:')(9

7!$F$-- 71 4-/ 2E22*4

E

!"#$#%:')(&!"#$#%:')(:!"#$#%:');<!"#$#%:');(!"#$#%:');;!"#$#%:');)!"#$#%:');8!"#$#%:');%!"#$#%:');'!"#$#%:');9!"#$#%:');&!"#$#%:');:!"#$#%:'))<!"#$#%:'))(!"#$#%:'));!"#$#%:')))!"#$#%:'))8!"#$#%:'))%!"#$#%:'))'!"#$#%:'))9!"#$#%:'))&!"#$#%:')):!"#$#%:')8<!"#$#%:')8(

!"#$#%:')8;!"#$#%:')8)!"#$#%:')88!"#$#%:')8%!"#$#%:')8'!"#$#%:')89!"#$#%:')8&!"#$#%:')8:!"#$#%:')%<!"#$#%:')%(!"#$#%:')%;

",> $ 71 4-/ 2E22*4 $4142 4! ! 4 ! , 2

|  |  | E | 2 !, 0 6,1 "> * !5 * $ 2 ! * 0 > 6 |
| --- | --- | --- | --- |
| !"#$#%:')%)!"#$#%:')%8!"#$#%:')%%  !"#$#%:')%' | 71 4-/ E | 71 4-/  E | 2E22*4 |

!"#$#%:')%9!"#$#%:')%&!"#$#%:')%:!"#$#%:')'<!"#$#%:')'(!"#$#%:')';!"#$#%:')')!"#$#%:')'8!"#$#%:')'%!"#$#%:')''!"#$#%:')'9!"#$#%:')'&!"#$#%:')':!"#$#%:')9<!"#$#%:')9(!"#$#%:')9;!"#$#%:')9)!"#$#%:')98!"#$#%:')9%!"#$#%:')9'!"#$#%:')99

|  | 7!$F$-- | 71 4-/  E | 2E22*4 |
| --- | --- | --- | --- |
| !"#$#%:')&' | 1  /E AE ?1/EAE @1 7 ?17@ | 1  /E AE  ?1/EAE @1 7 ?17@ | 2 2 + / *4 * " |
| !"#$#'<;%%:!"#$#'<;%'<!"#$#'<;%'( | * > $ | *  > $ | / 7 -+ |


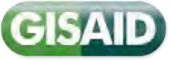


|  |  |
| --- | --- |
| !"#$#%&'()* +," - ." - /0 1" 2 3 ,-1.045414,1/6  71 61 045414,16$ 8 9- 1: ".1"/8 79 1045414,16+7045414,16 7 ! 01",1 | 21 1 2 ! -$ " +; ! ! 9 3 9  3 4 3 9 ! < <+2+ 1  1 3 ++! 48 " , ==! ;22" 3 02 " 4 |
| !"#$#%&'(>? +," - ." - /0 1" 2 3 ,-1.045414,1/6  71 61 045414,16$ 8 9- 1: ".1"/8 79 1045414,16+7045414,16 7 ! 01",1  !"#$#%&'(>(!"#$#%&'(>)!"#$#%&'(>>!"#$#%&'(>@!"#$#%&'(>%!"#$#%&'(>'!"#$#%&'(>&!"#$#%&'(>*!"#$#%&'(>A!"#$#%&'(@?!"#$#%&'(@(!"#$#%&'(@)!"#$#%&'(@>!"#$#%&'(@@ | 21 1 2 ! -$ " +; ! ! 9 3 9  3 4 3 +9 $ 7 9! 2+! 48 " , ==! ;22" 3 02 4 -1 |
| " 2 B $0."2B50/ | 3 |
| !"#$#%&'(@% +,-1 ." - /0 1" 2 3 ,-1.045414,1/6  71 61 045414,16$ 8 9- 1: ".1"/8 79 1045414,16+7045414,16 7 ! 01",1 | 21 1 2 ! -$ " +; ! ! 9 3 9  3 4 3 -0 0 |
| !"#$#%&'(@'!"#$#%&'(@& +CB +CB $ B ! C 31,  !"#$#%&'(@* $ B ! C D7E0 E  31, | 1 4 $ < $+ 3 9 |
| !"#$#%&'(@A +CB +CB $ B ! C 31,  $ B ! C D7E0 E | 1 4 $ < $+ 3 9 |

# 31, !"#$#%&'(%?!"#$#%&'(%(!"#$#%&'(%)!"#$#%&'(%>!"#$#%&'(%@!"#$#%&'(%%!"#$#%&'(%'!"#$#%&'(%&!"#$#%&'(%*!"#$#%&'(%A!"#$#%&'('?!"#$#%&'('(!"#$#%&'(')!"#$#%&'('>!"#$#%&'('@!"#$#%&'('%!"#$#%&'(''!"#$#%&'('&!"#$#%&'('*!"#$#%&'('A

|  | " 2 $ | " 2 $ | 2 |
| --- | --- | --- | --- |
| !"#$#%&'(&?!"#$#%&'(&( | "2 < $ | "+B <B 77 " | 8$1 <$DF4G 89,+ 17 + ""7 2 H9 |
| !"#$#%&'(&) | 93$ | "+B <B 77 " | 8$1 <$DF4G 89,+ 17 + ""7 2 H9 |
| !"#$#%&'(&> | 0$2 5< ! | "+B <B 77 " | 89DF<$8$4G ,7 + ""7 2 H9 |
| !"#$#%&'(&@ | B57 $ | "+B <B 77 " | 89DF<$8$4G ,7 + ""7 2 H9 |
| !"#$#%&'(&%!"#$#%&'(&' | 93$ | "+B <B 77 " | 8$1 <$DF4G 89,+ 17 + ""7 2 H9 |
| !"#$#%&'(&& | 77"2B + $ | "+B <B 77 " | 89DF<$8$4G ,7 + ""7 2 H9 |
| !"#$#%&'(&* | 7" 2 $I 0 | "+B <B 77 " | 89DF<$8$4G ,7 + ""7 2 H9 |
| !"#$#%&'(&A!"#$#%&'(*? !"#$#%&'(*(!"#$#%&'(*) !"#$#%&'(*>!"#$#%&'(*@  !"#$#%&'(*%!"#$#%&'(*'  !"#$#%&'(*&!"#$#%&'(** | $2 | "+B <B 77 " | 89DF<$8$4G ,7 + ""7 2 H9 |
| !"#$#%&'(*A!"#$#%&'(A? !"#$#%&'(A(!"#$#%&'(A)  !"#$#%&'(A>!"#$#%&'(A@ !"#$#%&'(A% | " 2 $ | "+B <B 77 " | 89DF<$8$4G ,7 + ""7 2 H9 |
| !"#$#%&'(A'!"#$#%&'(A& | + 2 5" 2 $ | "+B <B 77 " | 89DF<$8$4G ,7 + ""7 2 H9 |

# !"#$#%&'(A*!"#$#%&'(AA!"#$#%&')??!"#$#%&')?(!"#$#%&')?)!"#$#%&')?>!"#$#%&')?@!"#$#%&')?%!"#$#%&')?'!"#$#%&')?&!"#$#%&')?*!"#$#%&')?A!"#$#%&')(?!"#$#%&')((!"#$#%&')()!"#$#%&')(>!"#$#%&')(@!"#$#%&')(%!"#$#%&')('!"#$#%&')(&!"#$#%&')(*!"#$#%&')(A!"#$#%&'))?!"#$#%&'))( 12 2 12 2 < $ < 21+ 14 < $ !"#$#%&'))>!"#$#%&'))@ " 2 $ " 2 $ 2 !"#$#%&'))%!"#$#%&'))' !"#$#%&'))& !"#$#%&'))*!"#$#%&'))A!"#$#%&')>?!"#$#%&')>(!"#$#%&')>)!"#$#%&')>>!"#$#%&')>@!"#$#%&')>%!"#$#%&')>'!"#$#%&')>&!"#$#%&')>*!"#$#%&')>A!"#$#%&')@?!"#$#%&')@(!"#$#%&')@)!"#$#%&')@>!"#$#%&')@@!"#$#%&')@%!"#$#%&')@'!"#$#%&')@&!"#$#%&')@*!"#$#%&')@A!"#$#%&')%?!"#$#%&')%( !"#$#%&')%)!"#$#%&')%>!"#$#%&')%@!"#$#%&')%%!"#$#%&')%'

|  | 12 " 2  $ | 12 " 2 $ | 1" D H$;J |
| --- | --- | --- | --- |
| !"#$#%&')%&!"#$#%&')%* !"#$#%&')%A!"#$#%&')'? | +!  .3+!/ | +! .3+!/ | <5< + ;51 7 5 37 ;5;93 ;5 5 ;0 5  !157$ 2;5+$;51;!+;5; ; |
| !"#$#%&')'(!"#$#%&')') !"#$#%&')'> | +!  .3+!/ | +! .3+!/ | <5< + ;51 7 5 37 ;5;93 ;5 5 ;;5  + ;!157$ 2;5+$;51;!+;5; ; |
| !"#$#%&')'@!"#$#%&')'% | +!  .3+!/ | +! .3+!/ | !+;5; ; + ;51 7 5 37 ;5;93 ;5 5 ;;5  + ;!157$ 2;5+$;51; <5< |
| !"#$#%&')''!"#$#%&')'&  !"#$#%&')'*!"#$#%&')'A  !"#$#%&')&?!"#$#%&')&( | +!  .3+!/ | +! .3+!/ | !+;5; ; + ;51 7 5 37 ;5;93 ;5 5 ;;5  + ;0 5 $ 2;5+$;51; <5< |
| !"#$#%&')&) | +!  .3+!/ | +! .3+!/ | <5< + ;51 7 5 37 ;5;93 ;5 5 ;0 5  !157$ 2;5+$;51;!+;5; ; |
| !"#$#%&')&> | +!  .3+!/ | +! .3+!/ | !+;5; ; + ;51 7 5 37 ;5;93 ;5 5 ;;5  + ;0 5 $ 2;5+$;51; <5< |
| !"#$#%&')&@!"#$#%&')&% !"#$#%&')&' | +!  .3+!/ | +! .3+!/ | <5< + ;51 7 5 37 ;5;93 ;5 5 ;;5  + ;!157$ 2;5+$;51;!+;5; ; |
| !"#$#%&')&&!"#$#%&')&* | +!  .3+!/ | +! .3+!/ | !+;5; ; + ;51 7 5 37 ;5;93 ;5 5 ;;5  + ;!157$ 2;5+$;51; <5< |
| !"#$#%&')&A | +!  .3+!/ | +! .3+!/ | !+;5; ; + ;51 7 5 37 ;5;93 ;5 5 ;;5  + ;0 5 $ 2;5+$;51; <5< |

# !"#$#%&')*>!"#$#%&')*%!"#$#%&')*'!"#$#%&')*&!"#$#%&')**!"#$#%&')*A!"#$#%&')A?!"#$#%&')A)!"#$#%&')A>!"#$#%&')A&!"#$#%&')A*!"#$#%&')AA!"#$#%&'>??!"#$#%&'>?(!"#$#%&'>?)!"#$#%&'>?@!"#$#%&'>?%!"#$#%&'>?'!"#$#%&'>?&!"#$#%&'>?*!"#$#%&'>?A!"#$#%&'>(?!"#$#%&'>((!"#$#%&'>() !"#$#%&'>(>!"#$#%&'>(%!"#$#%&'>('!"#$#%&'>(&!"#$#%&'>(*!"#$#%&'>(A!"#$#%&'>)?!"#$#%&'>)(!"#$#%&'>)@!"#$#%&'>)% 9H2 .9J2/ 9H2 .9J2/ +9 <DF1 +" 147 + $4 !"#$#%&'>>(!"#$#%&'>>)!"#$#%&'>>>!"#$#%&'>>@!"#$#%&'>@&!"#$#%&'>@*!"#$#%&'>@A!"#$#%&'>%(!"#$#%&'>%>!"#$#%&'>%@!"#$#%&'>%%!"#$#%&'>%'!"#$#%&'>%*!"#$#%&'>%A!"#$#%&'>'?!"#$#%&'>'(

|  | 9H2 .9J2/ | 9H2 .9J2/ | +9 <1 " ;DF1 +" 147 + $4 |
| --- | --- | --- | --- |
| !"#$#%&'>*> | +,< + 41 | ." - /0 1" 2 3 ,-1.045414,1/6  71 61 045414,16$ 8  9- 1: ".1"/8 79 1045414,16+7045414,16 7 ! 01",16< <9 4$ " " .<<94$""/8 | 21 1 2 ! -$ " +; ! ! 9 3 9  3 4 3 21 1 49-+:0 ; |
| !"#$#%&'>*% | 91 7<-4 | 3 2 + | "26 6" 463 6 26"436 96" 6B |

# !"#$#%&'>*'!"#$#%&'>*& 3 2 + 3 2 + "26 6" 463 6 26" 436 96 3462 363 ,61 6062+6 36260963 16 " 6++6 64 D64 3$61 06 $64626+; 6" 36B

!"#$#%&'>AA!"#$#%&'@??!"#$#%&'@?(!"#$#%&'@?)!"#$#%&'@?>!"#$#%&'@?@!"#$#%&'@?%!"#$#%&'@?'!"#$#%&'@?&!"#$#%&'@?*!"#$#%&'@?A!"#$#%&'@(?!"#$#%&'@((!"#$#%&'@()!"#$#%&'@(>!"#$#%&'@(@!"#$#%&'@(%!"#$#%&'@('!"#$#%&'@(&!"#$#%&'@(*!"#$#%&'@(A!"#$#%&'@)?!"#$#%&'@)(!"#$#%&'@)) !"#$#%&'@)>!"#$#%&'@)@!"#$#%&'@)%!"#$#%&'@)'!"#$#%&'@)&!"#$#%&'@)*!"#$#%&'@)A!"#$#%&'@>?!"#$#%&'@>(!"#$#%&'@>)!"#$#%&'@>>!"#$#%&'@>@!"#$#%&'@>%!"#$#%&'@>'!"#$#%&'@>&!"#$#%&'@>*!"#$#%&'@>A!"#$#%&'@@?!"#$#%&'@@(!"#$#%&'@@)!"#$#%&'@@>!"#$#%&'@@@!"#$#%&'@@%!"#$#%&'@@'

!"#$#%&'@@&!"#$#%&'@@*!"#$#%&'@@A!"#$#%&'@%?!"#$#%&'@%(!"#$#%&'@%)!"#$#%&'@%>!"#$#%&'@%@!"#$#%&'@%%!"#$#%&'@%'!"#$#%&'@%&!"#$#%&'@%*!"#$#%&'@%A!"#$#%&'@'?!"#$#%&'@'(!"#$#%&'@')!"#$#%&'@'>!"#$#%&'@'@!"#$#%&'@'%!"#$#%&'@''!"#$#%&'@'&!"#$#%&'@'*!"#$#%&'@'A!"#$#%&'@&? !"#$#%&'@&(!"#$#%&'@&)!"#$#%&'@&>!"#$#%&'@&@!"#$#%&'@&%!"#$#%&'@&'!"#$#%&'@&&!"#$#%&'@&*!"#$#%&'@&A!"#$#%&'@*?!"#$#%&'@*(!"#$#%&'@*)!"#$#%&'@*>!"#$#%&'@*@!"#$#%&'@*%!"#$#%&'@*'!"#$#%&'@*&!"#$#%&'@**!"#$#%&'@*A!"#$#%&'@A?!"#$#%&'@A(!"#$#%&'@A)!"#$#%&'@A>!"#$#%&'@A@

# !"#$#%&'@A%!"#$#%&'@A'!"#$#%&'@A&!"#$#%&'@A*!"#$#%&'@AA!"#$#%&'%??!"#$#%&'%?(!"#$#%&'%?)!"#$#%&'%?>!"#$#%&'%?@!"#$#%&'%?%!"#$#%&'%?'!"#$#%&'%?&!"#$#%&'%?*!"#$#%&'%?A!"#$#%&'%(?!"#$#%&'%((!"#$#%&'%()!"#$#%&'%(>!"#$#%&'%(@!"#$#%&'%(%!"#$#%&'%('!"#$#%&'%(& ,B $ ,B $ "+ 2J$B1+ 15$2 4+D H !"#$#%&'%(*!"#$#%&'%(A!"#$#%&'%)?!"#$#%&'%)(!"#$#%&'%))!"#$#%&'%)>!"#$#%&'%)@!"#$#%&'%)%!"#$#%&'%)'!"#$#%&'%)&!"#$#%&'%)*!"#$#%&'%)A!"#$#%&'%>?!"#$#%&'%>(!"#$#%&'%>)!"#$#%&'%>>!"#$#%&'%>@!"#$#%&'%>%!"#$#%&'%>'!"#$#%&'%>&!"#$#%&'%>*!"#$#%&'%>A!"#$#%&'%@?!"#$#%&'%@( !"#$#%&'%@)!"#$#%&'%@>!"#$#%&'%@@!"#$#%&'%@%!"#$#%&'%@'!"#$#%&'%@&!"#$#%&'%@*!"#$#%&'%@A!"#$#%&'%%?!"#$#%&'%%(!"#$#%&'%%)!"#$#%&'%%>!"#$#%&'%%@!"#$#%&'%%%!"#$#%&'%%'

|  | ,7< | ,7< | 2" 0 $$ 1D 7 |
| --- | --- | --- | --- |
| !"#$#%&'%%&!"#$#%&'%%* | ,B $ | ,B $ | "+ 2J$B1+ 15$2 4+D H |

# !"#$#%&'%%A!"#$#%&'%'? !"#$#%&'%'(!"#$#%&'%') !"#$#%&'%'>!"#$#%&'%'@

!"#$#%&'%'A!"#$#%&'%&?!"#$#%&'%&(!"#$#%&'%&)!"#$#%&'%&>!"#$#%&'%&@!"#$#%&'%&%!"#$#%&'%&'!"#$#%&'%&&!"#$#%&'%&*!"#$#%&'%&A!"#$#%&'%*?!"#$#%&'%*(!"#$#%&'%*)!"#$#%&'%*>!"#$#%&'%*@!"#$#%&'%*%!"#$#%&'%*'!"#$#%&'%*&!"#$#%&'%**!"#$#%&'%*A!"#$#%&'%A?!"#$#%&'%A(!"#$#%&'%A) !"#$#%&'%A>!"#$#%&'%A@!"#$#%&'%A%!"#$#%&'%A'!"#$#%&'%A&!"#$#%&'%A*!"#$#%&'%AA!"#$#%&''??!"#$#%&''?(!"#$#%&''?)!"#$#%&''?>!"#$#%&''?@!"#$#%&''?%!"#$#%&''?'!"#$#%&''?&!"#$#%&''?*!"#$#%&''?A!"#$#%&''(?!"#$#%&''((!"#$#%&''()!"#$#%&''(>!"#$#%&''(@!"#$#%&''(%!"#$#%&''('

# !"#$#%&''(&!"#$#%&''(*!"#$#%&''(A!"#$#%&'')?!"#$#%&'')(!"#$#%&''))!"#$#%&'')>!"#$#%&'')@!"#$#%&'')%!"#$#%&'')'!"#$#%&'')&!"#$#%&'')*!"#$#%&'')A!"#$#%&''>?!"#$#%&''>(!"#$#%&''>)!"#$#%&''>>!"#$#%&''>@!"#$#%&''>%!"#$#%&''>'!"#$#%&''>&!"#$#%&''>*!"#$#%&''>A!"#$#%&''@? !"#$#%&''@(!"#$#%&''@)!"#$#%&''@>!"#$#%&''@@!"#$#%&''@%!"#$#%&''@'!"#$#%&''@&!"#$#%&''@*!"#$#%&''@A!"#$#%&''%?!"#$#%&''%(!"#$#%&''%)!"#$#%&''%>!"#$#%&''%@!"#$#%&''%%!"#$#%&''%'!"#$#%&''%&!"#$#%&''%*!"#$#%&''%A!"#$#%&'''?!"#$#%&'''(!"#$#%&''')!"#$#%&'''>!"#$#%&'''@ !"#$#%&'''%!"#$#%&''''!"#$#%&'''&!"#$#%&'''*!"#$#%&'''A!"#$#%&''&?!"#$#%&''&(!"#$#%&''&)!"#$#%&''&>!"#$#%&''&@!"#$#%&''&%!"#$#%&''&'!"#$#%&''&&!"#$#%&''&*!"#$#%&''&A!"#$#%&''*?!"#$#%&''*(!"#$#%&''*)!"#$#%&''*>!"#$#%&''*@!"#$#%&''*%!"#$#%&''*'!"#$#%&''*&!"#$#%&''** !"#$#%&''*A!"#$#%&''A?!"#$#%&''A(!"#$#%&''A)!"#$#%&''A>!"#$#%&''A@!"#$#%&''A%!"#$#%&''A'!"#$#%&''A&!"#$#%&''A*!"#$#%&''AA!"#$#%&'&??!"#$#%&'&?(!"#$#%&'&?)!"#$#%&'&?>!"#$#%&'&?@!"#$#%&'&?%!"#$#%&'&?'!"#$#%&'&?&!"#$#%&'&?*!"#$#%&'&?A!"#$#%&'&(?!"#$#%&'&((!"#$#%&'&() !"#$#%&'&(>!"#$#%&'&(@!"#$#%&'&(%!"#$#%&'&('!"#$#%&'&(&!"#$#%&'&(*!"#$#%&'&(A!"#$#%&'&)?!"#$#%&'&)(!"#$#%&'&))!"#$#%&'&)>!"#$#%&'&)@!"#$#%&'&)%!"#$#%&'&)'!"#$#%&'&)&!"#$#%&'&)*!"#$#%&'&)A!"#$#%&'&>?!"#$#%&'&>(!"#$#%&'&>)!"#$#%&'&>>!"#$#%&'&>@!"#$#%&'&>%!"#$#%&'&>' !"#$#%&'&>&!"#$#%&'&>*!"#$#%&'&>A!"#$#%&'&@?!"#$#%&'&@(!"#$#%&'&@)!"#$#%&'&@>!"#$#%&'&@@!"#$#%&'&@%!"#$#%&'&@'!"#$#%&'&@&!"#$#%&'&@*!"#$#%&'&@A!"#$#%&'&%?!"#$#%&'&%(!"#$#%&'&%)!"#$#%&'&%>!"#$#%&'&%@!"#$#%&'&%%!"#$#%&'&%'!"#$#%&'&%&!"#$#%&'&%*!"#$#%&'&%A!"#$#%&'&'? !"#$#%&'&'(!"#$#%&'&')!"#$#%&'&'>!"#$#%&'&'@!"#$#%&'&'%!"#$#%&'&''!"#$#%&'&'&!"#$#%&'&'*!"#$#%&'&'A!"#$#%&'&&?!"#$#%&'&&(!"#$#%&'&&)!"#$#%&'&&>!"#$#%&'&&@!"#$#%&'&&%!"#$#%&'&&'!"#$#%&'&&&!"#$#%&'&&*!"#$#%&'&&A!"#$#%&'&*?!"#$#%&'&*(!"#$#%&'&*)!"#$#%&'&*>!"#$#%&'&*@ !"#$#%&'&*%!"#$#%&'&*'!"#$#%&'&*&!"#$#%&'&**!"#$#%&'&*A!"#$#%&'&A?!"#$#%&'&A(!"#$#%&'&A)!"#$#%&'&A>!"#$#%&'&A@!"#$#%&'&A%!"#$#%&'&A'!"#$#%&'&A&!"#$#%&'&A*!"#$#%&'&AA!"#$#%&'*??!"#$#%&'*?(!"#$#%&'*?)!"#$#%&'*?>!"#$#%&'*?@!"#$#%&'*?%!"#$#%&'*?'!"#$#%&'*?&!"#$#%&'*?* !"#$#%&'*?A!"#$#%&'*(?!"#$#%&'*((!"#$#%&'*()!"#$#%&'*(>!"#$#%&'*(@!"#$#%&'*(%!"#$#%&'*('!"#$#%&'*(&!"#$#%&'*(*!"#$#%&'*(A!"#$#%&'*)?!"#$#%&'*)(!"#$#%&'*))!"#$#%&'*)>!"#$#%&'*)@!"#$#%&'*)%!"#$#%&'*)'!"#$#%&'*)&!"#$#%&'*)*!"#$#%&'*)A!"#$#%&'*>?!"#$#%&'*>(!"#$#%&'*>) !"#$#%&'*>>!"#$#%&'*>@!"#$#%&'*>%!"#$#%&'*>'!"#$#%&'*>&!"#$#%&'*>*!"#$#%&'*>A!"#$#%&'*@?!"#$#%&'*@(!"#$#%&'*@)!"#$#%&'*@>!"#$#%&'*@@!"#$#%&'*@%!"#$#%&'*@'!"#$#%&'*@&!"#$#%&'*@*!"#$#%&'*@A!"#$#%&'*%?!"#$#%&'*%(!"#$#%&'*%)!"#$#%&'*%>!"#$#%&'*%@!"#$#%&'*%%!"#$#%&'*%' !"#$#%&'*%&!"#$#%&'*%*!"#$#%&'*%A!"#$#%&'*'?!"#$#%&'*'(!"#$#%&'*')!"#$#%&'*'>!"#$#%&'*'@!"#$#%&'*'%!"#$#%&'*''!"#$#%&'*'&!"#$#%&'*'*!"#$#%&'*'A!"#$#%&'*&?!"#$#%&'*&(!"#$#%&'*&)!"#$#%&'*&>!"#$#%&'*&@!"#$#%&'*&%!"#$#%&'*&' HB31,H6H 7B5(A,4.75,4/7 9 < 191771 H1D +1: ,2 6< 3 9"!31D$3 71 14< D970 22 !"#$#%&'*&&!"#$#%&'*&*!"#$#%&'*&A!"#$#%&'**?!"#$#%&'**(!"#$#%&'**)!"#$#%&'**>!"#$#%&'**@!"#$#%&'**%!"#$#%&'**&!"#$#%&'***!"#$#%&'**A!"#$#%&'*A?!"#$#%&'*A(!"#$#%&'*A)!"#$#%&'*A>!"#$#%&'*A@!"#$#%&'*A%!"#$#%&'*A' ,< 7B5(A,4.75,4/7 1 ,<K7 11 D 3 + " +DG 3 $ ,<9$K7 1 <7 "H4 " 1 4 H+ <"2!2 $K2 < G ! ;2 K7!"#$#%&'*A&!"#$#%&'*A*!"#$#%&'*AA!"#$#%&'A??!"#$#%&'A?(!"#$#%&'A?)!"#$#%&'A?>!"#$#%&'A?@!"#$#%&'A?'!"#$#%&'A?*!"#$#%&'A?A!"#$#%&'A(?!"#$#%&'A()!"#$#%&'A(>!"#$#%&'A(@!"#$#%&'A(%!"#$#%&'A('!"#$#%&'A(&!"#$#%&'A(* " ,7 7B5(A,4.75,4/7 D 87 2 12 1 7 1 " "

!"#$#%&'A)?!"#$#%&'A)(!"#$#%&'A))!"#$#%&'A)>!"#$#%&'A)@!"#$#%&'A)%!"#$#%&'A)'!"#$#%&'A)&!"#$#%&'A)*!"#$#%&'A)A!"#$#%&'A>?!"#$#%&'A>(!"#$#%&'A>)!"#$#%&'A>>!"#$#%&'A>@!"#$#%&'A>%!"#$#%&'A>'!"#$#%&'A>&!"#$#%&'A>*!"#$#%&'A>A!"#$#%&'A@?!"#$#%&'A@(!"#$#%&'A@)!"#$#%&'A@> !"#$#%&'A@@!"#$#%&'A@%!"#$#%&'A@'!"#$#%&'A@&!"#$#%&'A@*!"#$#%&'A@A!"#$#%&'A%?!"#$#%&'A%(!"#$#%&'A%)!"#$#%&'A%>!"#$#%&'A%@!"#$#%&'A%%!"#$#%&'A%'!"#$#%&'A%&!"#$#%&'A%*!"#$#%&'A%A!"#$#%&'A'?

|  | HB31,H6H  ,2 6< 3 22 | 7B5(A,4.75,4/7 | 9 < 191771 H1D +1:  9"!31D$3 71 14< D970 |
| --- | --- | --- | --- |
| !"#$#%&'A'@!"#$#%&'A'%  !"#$#%&'A'*!"#$#%&'A&'  !"#$#%&'A&&!"#$#%&'A*) | $ 7 $ | 7B5(A,4.75,4/7 | 2 $ "D 2H 1 ;1 D<-!  < 7 DD7 7 +! L5 1 "40 +  J272 12 1 ;5DD$$ D17 3 ! M9 7 -  + "5+ $ +3 "+ 9+" 9 D"3 !BD 1 |
| !"#$#%&'A*@!"#$#%&'A*% | ,7 $  2 7 320 9 7 2 329 | 7B5(A,4.75,4/7 | 7 + 1 4" + +9< 2 9 " 3 " 7 $ 08"D: 0 D <42" + D" 2 D < |

!"#$#%&'A*'!"#$#%&'A*&!"#$#%&'A**!"#$#%&'A*A!"#$#%&'AA?!"#$#%&'AA(!"#$#%&'AA)!"#$#%&'AA>!"#$#%&'AA@!"#$#%&'AA%!"#$#%&'AA'!"#$#%&'AA&!"#$#%&'AA*!"#$#%&'AAA!"#$#%&&???!"#$#%&&??(!"#$#%&&??)!"#$#%&&??>!"#$#%&&??@!"#$#%&&??%!"#$#%&&??'!"#$#%&&??&!"#$#%&&??*!"#$#%&&??A !"#$#%&&?(?!"#$#%&&?((!"#$#%&&?()!"#$#%&&?(>!"#$#%&&?(@!"#$#%&&?(%!"#$#%&&?('!"#$#%&&?(&!"#$#%&&?(*!"#$#%&&?(A!"#$#%&&?)?!"#$#%&&?)(!"#$#%&&?))!"#$#%&&?)>!"#$#%&&?)@!"#$#%&&?)%!"#$#%&&?)'!"#$#%&&?)&!"#$#%&&?)*!"#$#%&&?)A!"#$#%&&?>?!"#$#%&&?>(!"#$#%&&?>)!"#$#%&&?>>

# !"#$#%&&?>@!"#$#%&&?>%!"#$#%&&?>'!"#$#%&&?>&!"#$#%&&?>*!"#$#%&&?>A

|  | 3 ,I 92 32  0 9 I37 7  320 9 I392  320 9 I3 2 32 0 9 | 7B5(A,4.75,4/7 | $3 1<+8 D $D 19: 2 < 78$"  < $!HD"7 !<! <"D7 8 9! |
| --- | --- | --- | --- |

# !"#$#%&&?@(!"#$#%&&?@)!"#$#%&&?@>!"#$#%&&?@@!"#$#%&&?@%!"#$#%&&?@'!"#$#%&&?@&!"#$#%&&?@A!"#$#%&&?%?!"#$#%&&?%(!"#$#%&&?%)!"#$#%&&?%>!"#$#%&&?%@!"#$#%&&?%%!"#$#%&&?%'!"#$#%&&?%&!"#$#%&&?%*!"#$#%&&?'?!"#$#%&&?'(!"#$#%&&?')!"#$#%&&?'>!"#$#%&&?'@!"#$#%&&?'%!"#$#%&&?'' !"#$#%&&?'& 7!;, 7B5(A,4.75,4/7 < 8< ! 4 < + $ !< " I9 +$ 7 + " 2 329 !"#$#%&&?'*!"#$#%&&?'A!"#$#%&&?&?!"#$#%&&?&(!"#$#%&&?&)!"#$#%&&?&>!"#$#%&&?&@!"#$#%&&?&%!"#$#%&&?&'!"#$#%&&?&&!"#$#%&&?&*!"#$#%&&?&A!"#$#%&&?*?!"#$#%&&?*(!"#$#%&&?*)!"#$#%&&?*>!"#$#%&&?*@!"#$#%&&?*%!"#$#%&&?*'!"#$#%&&?*&!"#$#%&&?**!"#$#%&&?*A!"#$#%&&?A?!"#$#%&&?A( !"#$#%&&?A)!"#$#%&&?A>!"#$#%&&?A@!"#$#%&&?A%

|  | HB31,H6H  ,2 6< 3 22 | 7B5(A,4.75,4/7 | 9 < 191771 H1D +1:  9"!31D$3 71 14< D970 |
| --- | --- | --- | --- |

# !"#$#%&&?A'!"#$#%&&?A&!"#$#%&&?A*!"#$#%&&?AA!"#$#%&&(??!"#$#%&&(?(!"#$#%&&(?)!"#$#%&&(?>!"#$#%&&(?@!"#$#%&&(?%!"#$#%&&(?'!"#$#%&&(?&!"#$#%&&(?*!"#$#%&&(?A G < 7B5(A,4.75,4/7 D < $ 4 9$5B+ " 94 1;$ 13 50 $;;1+ 3;! 1 83 1 9 HD9+ < 7 < +" ! 1D1 D "D M

!"#$#%&&((?!"#$#%&&(((!"#$#%&&((>!"#$#%&&((@!"#$#%&&((%!"#$#%&&(('!"#$#%&&((&!"#$#%&&((*!"#$#%&&((A!"#$#%&&()?!"#$#%&&()(!"#$#%&&())!"#$#%&&()>!"#$#%&&()@!"#$#%&&()%!"#$#%&&()'!"#$#%&&()&!"#$#%&&()*!"#$#%&&()A!"#$#%&&(>?!"#$#%&&(>(!"#$#%&&(>)!"#$#%&&(>>!"#$#%&&(>@ !"#$#%&&(>%!"#$#%&&(>'!"#$#%&&(>&!"#$#%&&(>*!"#$#%&&(>A!"#$#%&&(@?!"#$#%&&(@(!"#$#%&&(@)!"#$#%&&(@>!"#$#%&&(@@!"#$#%&&(@%!"#$#%&&(@'!"#$#%&&(@&!"#$#%&&(@*!"#$#%&&(@A!"#$#%&&(%?!"#$#%&&(%(!"#$#%&&(%)!"#$#%&&(%>!"#$#%&&(%@!"#$#%&&(%'!"#$#%&&(%&!"#$#%&&(%*!"#$#%&&(%A !"#$#%&&('?!"#$#%&&('(!"#$#%&&(')!"#$#%&&('>!"#$#%&&('@!"#$#%&&('%!"#$#%&&(''!"#$#%&&('&!"#$#%&&('*!"#$#%&&('A!"#$#%&&(&?!"#$#%&&(&(!"#$#%&&(&)!"#$#%&&(&>!"#$#%&&(&@!"#$#%&&(&%!"#$#%&&(&'!"#$#%&&(&&!"#$#%&&(&*!"#$#%&&(&A!"#$#%&&(*?!"#$#%&&(*(!"#$#%&&(*)!"#$#%&&(*>

# !"#$#%&&(*@!"#$#%&&(*%!"#$#%&&(*'!"#$#%&&(*&!"#$#%&&(**!"#$#%&&(*A!"#$#%&&(A?!"#$#%&&(A(!"#$#%&&(A) G 1 77 1 7B5(A,4.75,4/7 7 1-4 B 10 1 1$225 D< " 17 D7 9 I:3 9 32 17 710DB1$ !"#$#%&&(A>!"#$#%&&(A@!"#$#%&&(A&!"#$#%&&)??!"#$#%&&)?(!"#$#%&&)?>!"#$#%&&)?@!"#$#%&&)?%!"#$#%&&)?'!"#$#%&&)?&!"#$#%&&)?*!"#$#%&&)?A!"#$#%&&)(?!"#$#%&&)(>!"#$#%&&)(@ 7!;, 7B5(A,4.75,4/7 < 8< ! 4 < + $ !< " I9 +$ 7 + " 2 329

!"#$#%&&))?!"#$#%&&))(!"#$#%&&))>!"#$#%&&))@!"#$#%&&))%!"#$#%&&))&!"#$#%&&))*!"#$#%&&))A!"#$#%&&)>?!"#$#%&&)>(!"#$#%&&)>)!"#$#%&&)>>!"#$#%&&)>@!"#$#%&&)>%!"#$#%&&)>'!"#$#%&&)>&!"#$#%&&)>*!"#$#%&&)>A!"#$#%&&)@?!"#$#%&&)@(!"#$#%&&)@)!"#$#%&&)@>!"#$#%&&)@@!"#$#%&&)@' !"#$#%&&)@&!"#$#%&&)@*!"#$#%&&)%?!"#$#%&&)%(!"#$#%&&)%)!"#$#%&&)%>!"#$#%&&)%@!"#$#%&&)%%!"#$#%&&)%'!"#$#%&&)%&!"#$#%&&)%*!"#$#%&&)%A!"#$#%&&)'?!"#$#%&&)'(!"#$#%&&)')!"#$#%&&)'>!"#$#%&&)'@!"#$#%&&)'%!"#$#%&&)''!"#$#%&&)'&!"#$#%&&)'A!"#$#%&&)&?!"#$#%&&)&(!"#$#%&&)&)

# !"#$#%&&)&>!"#$#%&&)&@!"#$#%&&)&'!"#$#%&&)&&!"#$#%&&)&*!"#$#%&&)&A!"#$#%&&)*?!"#$#%&&)*(!"#$#%&&)*)!"#$#%&&)*>!"#$#%&&)*%!"#$#%&&)*'!"#$#%&&)*&!"#$#%&&)**!"#$#%&&)*A!"#$#%&&)A?!"#$#%&&)A(!"#$#%&&)A>!"#$#%&&)A@!"#$#%&&)A%!"#$#%&&)A'!"#$#%&&)A&!"#$#%&&)A* ,!H 7B5(A,4.75,4/7 <9D1 1 D 5 0+1 1 D1 !"#$#%&&)AA!"#$#%&&>??!"#$#%&&>?)!"#$#%&&>?@!"#$#%&&>?%!"#$#%&&>?'!"#$#%&&>?&!"#$#%&&>?A!"#$#%&&>(?!"#$#%&&>((!"#$#%&&>(>!"#$#%&&>(@!"#$#%&&>(%!"#$#%&&>('!"#$#%&&>(&!"#$#%&&>(*!"#$#%&&>(A!"#$#%&&>)?!"#$#%&&>)(!"#$#%&&>))!"#$#%&&>)@!"#$#%&&>)%!"#$#%&&>)'!"#$#%&&>)& !"#$#%&&>)*!"#$#%&&>)A!"#$#%&&>>?!"#$#%&&>>(!"#$#%&&>>>!"#$#%&&>>@!"#$#%&&>>%!"#$#%&&>>'!"#$#%&&>>&!"#$#%&&>>A!"#$#%&&>@?!"#$#%&&>@(!"#$#%&&>@)!"#$#%&&>@>!"#$#%&&>@@!"#$#%&&>@%

|  | 7!;,  " I9 +$ " 2 329 | | | 7B5(A,4.75,4/7 | < 8< ! 4 < + $ !< 7 + |
| --- | --- | --- | --- | --- | --- |
| !"#$#%&&>@' | ,!H | | | 7B5(A,4.75,4/7 | <9D1 1 D 5 0+1 1 D1 |
| !"#$#%&&>@&!"#$#%&&>@* !"#$#%&&>@A!"#$#%&&>%?  !"#$#%&&>%)!"#$#%&&>%> | B + !  32$I <  ,! I | | | 7B5(A,4.75,4/7 | 12 1++ 1< ;7M9 N!2 B17D97 : +8 JD <+ 979 4 |
| !"#$#%&&>%@!"#$#%&&>%' !"#$#%&&>%&!"#$#%&&>%* | | 1 1,! |

!"#$#%&&>%A!"#$#%&&>'?!"#$#%&&>'(!"#$#%&&>')!"#$#%&&>'>!"#$#%&&>'@!"#$#%&&>'%!"#$#%&&>''!"#$#%&&>'&!"#$#%&&>'*!"#$#%&&>'A!"#$#%&&>&?!"#$#%&&>&(!"#$#%&&>&)!"#$#%&&>&>!"#$#%&&>&@!"#$#%&&>&%!"#$#%&&>&'!"#$#%&&>&&!"#$#%&&>&*!"#$#%&&>&A!"#$#%&&>*?

| ,!H 7B5(A,4.75,4/7 | <9D1 1 D 5 0+1 1 D1 |
| --- | --- |
| !"#$#%&&>*(!"#$#%&&>*) 7!;, 7B5(A,4.75,4/7  " I9 +$ " 2 329 | < 8< ! 4 < + $ !< 7 + |
| !"#$#%&&>*>!"#$#%&&>*@ HB31,H6H 7B5(A,4.75,4/7  ,2 6< 3  22  !"#$#%&&>*&!"#$#%&&>**!"#$#%&&>*A!"#$#%&&>A?!"#$#%&&>A(!"#$#%&&>A>!"#$#%&&>A@!"#$#%&&>A%!"#$#%&&>A'!"#$#%&&>A&!"#$#%&&>A*!"#$#%&&@?? | 9 < 191771 H1D +1:  9"!31D$3 71 14< D970 |
| 7!;, 7B5(A,4.75,4/7  " I9 +$ | < 8< ! 4 < + $ !< 7 + |

# " 2 329 !"#$#%&&@?(!"#$#%&&@?)!"#$#%&&@?>!"#$#%&&@?@!"#$#%&&@?%!"#$#%&&@?'!"#$#%&&@?&!"#$#%&&@?*!"#$#%&&@?A!"#$#%&&@(?!"#$#%&&@((!"#$#%&&@()!"#$#%&&@(>!"#$#%&&@(@!"#$#%&&@(%!"#$#%&&@(' HB31,H6H 7B5(A,4.75,4/7 9 < 191771 H1D +1: ,2 6< 3 9"!31D$3 71 14< D970 22 !"#$#%&&@(&!"#$#%&&@(*!"#$#%&&@(A!"#$#%&&@)?!"#$#%&&@)(!"#$#%&&@))!"#$#%&&@)>!"#$#%&&@)@!"#$#%&&@)%!"#$#%&&@)'!"#$#%&&@)&!"#$#%&&@)*!"#$#%&&@)A!"#$#%&&@>?!"#$#%&&@>(!"#$#%&&@>)!"#$#%&&@>>!"#$#%&&@>@!"#$#%&&@>'!"#$#%&&@>&!"#$#%&&@>*!"#$#%&&@>A!"#$#%&&@@?!"#$#%&&@@( !"#$#%&&@@)!"#$#%&&@@>!"#$#%&&@@@!"#$#%&&@@%!"#$#%&&@@'!"#$#%&&@@&!"#$#%&&@@*!"#$#%&&@@A!"#$#%&&@%?!"#$#%&&@%(!"#$#%&&@%)!"#$#%&&@%>!"#$#%&&@%@!"#$#%&&@%%!"#$#%&&@%'!"#$#%&&@%&!"#$#%&&@%*!"#$#%&&@%A!"#$#%&&@'?!"#$#%&&@'(!"#$#%&&@')!"#$#%&&@'>!"#$#%&&@'@!"#$#%&&@'% !"#$#%&&@''!"#$#%&&@'&!"#$#%&&@'*!"#$#%&&@'A!"#$#%&&@&?!"#$#%&&@&(!"#$#%&&@&)!"#$#%&&@&>!"#$#%&&@&@!"#$#%&&@&%!"#$#%&&@&'!"#$#%&&@&&!"#$#%&&@&*!"#$#%&&@&A!"#$#%&&@*?!"#$#%&&@*(!"#$#%&&@*)!"#$#%&&@*>!"#$#%&&@*@!"#$#%&&@*%!"#$#%&&@*'!"#$#%&&@*&!"#$#%&&@**!"#$#%&&@*A !"#$#%&&@A?!"#$#%&&@A(!"#$#%&&@A)!"#$#%&&@A>!"#$#%&&@A@!"#$#%&&@A%!"#$#%&&@A'!"#$#%&&@A&!"#$#%&&@A*!"#$#%&&@AA!"#$#%&&%??!"#$#%&&%?(!"#$#%&&%?)!"#$#%&&%?>!"#$#%&&%?@!"#$#%&&%?%!"#$#%&&%?'!"#$#%&&%?&!"#$#%&&%?*!"#$#%&&%?A!"#$#%&&%(?!"#$#%&&%((!"#$#%&&%()!"#$#%&&%(> !"#$#%&&%(%!"#$#%&&%('!"#$#%&&%(&!"#$#%&&%(*!"#$#%&&%(A!"#$#%&&%)?!"#$#%&&%)(!"#$#%&&%))!"#$#%&&%)>!"#$#%&&%)@!"#$#%&&%)%!"#$#%&&%)'!"#$#%&&%)&!"#$#%&&%)*!"#$#%&&%)A!"#$#%&&%>?!"#$#%&&%>(!"#$#%&&%>)!"#$#%&&%>>!"#$#%&&%>@!"#$#%&&%>%!"#$#%&&%>'!"#$#%&&%>&!"#$#%&&%>* !"#$#%&&%>A!"#$#%&&%@?!"#$#%&&%@(!"#$#%&&%@)!"#$#%&&%@>

|  | B 7: K  ", | 7B5(A,4.75,4/7 | 71D!$ 1 "7 1 < H <5 H D7D $ $3 "4 ;5 9 D+1< D 797 |
| --- | --- | --- | --- |
| !"#$#%&&%@%!"#$#%&&%@' | 7!;,  " I9 +$ " 2 329 | 7B5(A,4.75,4/7 | < 8< ! 4 < + $ !< 7 + |

# !"#$#%&&%@&!"#$#%&&%@*!"#$#%&&%@A!"#$#%&&%%?!"#$#%&&%%(!"#$#%&&%%)!"#$#%&&%%>!"#$#%&&%%@!"#$#%&&%%%!"#$#%&&%%'!"#$#%&&%%&!"#$#%&&%%*!"#$#%&&%%A!"#$#%&&%'?!"#$#%&&%'(!"#$#%&&%')!"#$#%&&%'>!"#$#%&&%'%!"#$#%&&%''!"#$#%&&%'&!"#$#%&&%'*!"#$#%&&%'A!"#$#%&&%&?!"#$#%&&%&( !"#$#%&&%&)!"#$#%&&%&>!"#$#%&&%&@!"#$#%&&%&%!"#$#%&&%&'!"#$#%&&%&&!"#$#%&&%&*!"#$#%&&%&A!"#$#%&&%*?!"#$#%&&%*(!"#$#%&&%*)!"#$#%&&%*>!"#$#%&&%*@!"#$#%&&%*%!"#$#%&&%*'!"#$#%&&%*&!"#$#%&&%**!"#$#%&&%*A!"#$#%&&%A?!"#$#%&&%A(!"#$#%&&%A)!"#$#%&&%A>!"#$#%&&%A@!"#$#%&&%A% !"#$#%&&%A'

| 12 2 12 2 < $  < $ | < 21+ 14 |
| --- | --- |
| !"#$#%&&%A& B B B B .B/3 2 " +  .B/3 2 " + | + < + |
| !"#$#%&&'?@ B B B B .B/3 2 " +  .B/3 2 " + | + < + B2 |
| !"#$#%&&'?& + 22 + 22  !"#$#%&&')>!"#$#%&&')@!"#$#%&&')%!"#$#%&&')'!"#$#%&&')&!"#$#%&&')*!"#$#%&&')A!"#$#%&&'>?!"#$#%&&'>(!"#$#%&&'>)!"#$#%&&'>>!"#$#%&&'>@!"#$#%&&'>%!"#$#%&&'>'!"#$#%&&'>&!"#$#%&&'>* | 7 $ 2+ |
| 93 " 2 B " | 36D263 $69 6BD |

# !"#$#%&&'>A!"#$#%&&'@?!"#$#%&&'@(!"#$#%&&'@)!"#$#%&&'@>!"#$#%&&'@@!"#$#%&&'@%!"#$#%&&'@'!"#$#%&&'@&!"#$#%&&'@*!"#$#%&&'@A!"#$#%&&'%?!"#$#%&&'%(!"#$#%&&'%)!"#$#%&&'%>!"#$#%&&'%%!"#$#%&&'%'!"#$#%&&'%&!"#$#%&&'%*!"#$#%&&'%A!"#$#%&&''?!"#$#%&&''(!"#$#%&&'')!"#$#%&&''> !"#$#%&&''@!"#$#%&&''%!"#$#%&&'''!"#$#%&&''&!"#$#%&&''*!"#$#%&&''A!"#$#%&&'&?!"#$#%&&'&)!"#$#%&&'&%!"#$#%&&'&'!"#$#%&&'&&!"#$#%&&'&*!"#$#%&&'&A!"#$#%&&'*?!"#$#%&&'*(!"#$#%&&'*)!"#$#%&&'*>!"#$#%&&'*@!"#$#%&&'*%!"#$#%&&'*'!"#$#%&&'*&!"#$#%&&'**!"#$#%&&'*A!"#$#%&&'A? !"#$#%&&'A(!"#$#%&&'A)!"#$#%&&'A>!"#$#%&&'A@!"#$#%&&'A%!"#$#%&&'A'!"#$#%&&'A&!"#$#%&&'A*!"#$#%&&'AA!"#$#%&&&??!"#$#%&&&?(!"#$#%&&&?)!"#$#%&&&?@!"#$#%&&&?%!"#$#%&&&?'!"#$#%&&&?&!"#$#%&&&?*!"#$#%&&&?A!"#$#%&&&(?!"#$#%&&&((!"#$#%&&&()!"#$#%&&&(>!"#$#%&&&(@!"#$#%&&&(% !"#$#%&&&('!"#$#%&&&(&!"#$#%&&&(*!"#$#%&&&(A!"#$#%&&&)?!"#$#%&&&)(!"#$#%&&&))!"#$#%&&&)>!"#$#%&&&)@!"#$#%&&&)%!"#$#%&&&)'!"#$#%&&&)&!"#$#%&&&)*!"#$#%&&&)A!"#$#%&&&>?!"#$#%&&&>(!"#$#%&&&>)!"#$#%&&&>>

|  | 3B ; | 3B ; | "B |
| --- | --- | --- | --- |
| !"#$#%&&&>@ | B < +7 < | 0 3 7 ,< | B O2P4L<QP<R<-PB OSP 0TP0 TP2 L PD -4PT 1$T PU L  $ PT P13PTP1 P P!RP! 9P9PQBVD;3 <4 |
| !"#$#%&&&>% | B < +7 < | 0 3 7 ,< | 4L<QPB O2P<R<-PB OSP 0TP0 TP2 L PD -4PT 1$T PU L  $ PT P13PTP1 P P!RP! 9P9PQBV<4 D;3 |
| !"#$#%&&&>' | B < +7 < | 0 3 7 ,< | B O2P4L<QP<R<-PB OSP 0TP0 TP2 L PD -4PT 1$T PU L  $ PT P13PTP1 P P!RP! 9P9PQBVD;3 <4 |
| !"#$#%&&&>& | B < +7 < | 0 3 7 ,< | 4L<QPB O2P<R<-PB OSP 0TP0 TP2 L PD -4PT 1$T PU L  $ PT P13PTP1 P P!RP! 9P9PQBV<4 D;3 |
| !"#$#%&&&>* | B < +7 < | 0 3 7 ,< | B O2P4L<QP<R<-PB OSP 0TP0 TP2 L PD -4PT 1$T PU L  $ PT P13PTP1 P P!RP! 9P9PQBVD;3 <4 |
| !"#$#%&&&>A | B < +7 < | 0 3 7 ,< | 4L<QPB O2P<R<-PB OSP 0TP0 TP2 L PD -4PT 1$T PU L  $ PT P13PTP1 P P!RP! 9P9PQBV<4 D;3 |
| !"#$#%&&&@?!"#$#%&&&@( !"#$#%&&&@) | B < +7 < | 0 3 7 ,< | <R<-PB O2P4L<QPB OSP 0TP0 TP2 L PD -4PT 1$T PU L  $ PT P13PTP1 P P!RP! 9P9PQBVD;3 <4 |
| !"#$#%&&&@>!"#$#%&&&@@  !"#$#%&&&@%!"#$#%&&&@'  !"#$#%&&&@&!"#$#%&&&@* | 7B5(A | ! 1 7 | < 1 + 3 -7 $1-<! 1 7 4  !- +1 14 7B5(A |

# !"#$#%&&&@A!"#$#%&&&%?!"#$#%&&&%)!"#$#%&&&%>!"#$#%&&&%@!"#$#%&&&%%!"#$#%&&&%'!"#$#%&&&%*!"#$#%&&&'(!"#$#%&&&'>!"#$#%&&&'@!"#$#%&&&'%!"#$#%&&&''!"#$#%&&&'&!"#$#%&&&'*!"#$#%&&&'A!"#$#%&&&&?!"#$#%&&&&(!"#$#%&&&&)!"#$#%&&&&>!"#$#%&&&&@!"#$#%&&&&%!"#$#%&&&&'!"#$#%&&&&& !"#$#%&&&&*!"#$#%&&&&A!"#$#%&&&*(!"#$#%&&&*>!"#$#%&&&*@!"#$#%&&&*%!"#$#%&&&*'!"#$#%&&&*&!"#$#%&&&*A!"#$#%&&&A?!"#$#%&&&A(!"#$#%&&&A)!"#$#%&&&A>!"#$#%&&&A@!"#$#%&&&A%!"#$#%&&&A'!"#$#%&&&A&!"#$#%&&&A*!"#$#%&&&AA!"#$#%&&*??!"#$#%&&*?(!"#$#%&&*?)!"#$#%&&*?>!"#$#%&&*?% !"#$#%&&*?&!"#$#%&&*?*!"#$#%&&*?A!"#$#%&&*(?!"#$#%&&*((!"#$#%&&*()!"#$#%&&*(>!"#$#%&&*(@!"#$#%&&*('!"#$#%&&*(&!"#$#%&&*)?!"#$#%&&*)(!"#$#%&&*)@!"#$#%&&*)%!"#$#%&&*)'!"#$#%&&*)&!"#$#%&&*)* 7B5(A ! 1 7 2 !"#$#%&&*)A!"#$#%&&*>?!"#$#%&&*>(!"#$#%&&*>)!"#$#%&&*>>!"#$#%&&*>@!"#$#%&&*>%!"#$#%&&*>&!"#$#%&&*>*!"#$#%&&*>A!"#$#%&&*@?!"#$#%&&*@(!"#$#%&&*@)!"#$#%&&*@>!"#$#%&&*@@!"#$#%&&*@%!"#$#%&&*@'!"#$#%&&*@&!"#$#%&&*@*!"#$#%&&*@A!"#$#%&&*%?!"#$#%&&*%(!"#$#%&&*%)!"#$#%&&*%> !"#$#%&&*%@!"#$#%&&*%%!"#$#%&&*%'!"#$#%&&*%&!"#$#%&&*%*!"#$#%&&*%A!"#$#%&&*'(!"#$#%&&*')!"#$#%&&*'>!"#$#%&&*'@!"#$#%&&*'%!"#$#%&&*''!"#$#%&&*'*!"#$#%&&*'A!"#$#%&&*&?!"#$#%&&*&(!"#$#%&&*&)!"#$#%&&*&>!"#$#%&&*&@!"#$#%&&*&%!"#$#%&&*&&!"#$#%&&*&A!"#$#%&&**?!"#$#%&&**( !"#$#%&&**)!"#$#%&&**>!"#$#%&&**@!"#$#%&&**%!"#$#%&&**'!"#$#%&&**&!"#$#%&&***!"#$#%&&**A!"#$#%&&*A?!"#$#%&&*A(!"#$#%&&*A)!"#$#%&&*A>!"#$#%&&*A@!"#$#%&&*A%!"#$#%&&*A'!"#$#%&&*A&!"#$#%&&*A*!"#$#%&&*AA!"#$#%&&A??!"#$#%&&A?(!"#$#%&&A?)!"#$#%&&A?>!"#$#%&&A?@!"#$#%&&A?% !"#$#%&&A?'!"#$#%&&A?*!"#$#%&&A?A!"#$#%&&A(?!"#$#%&&A((!"#$#%&&A()!"#$#%&&A(>!"#$#%&&A(@!"#$#%&&A(%!"#$#%&&A('!"#$#%&&A(*!"#$#%&&A(A!"#$#%&&A)?!"#$#%&&A)(!"#$#%&&A))!"#$#%&&A)>!"#$#%&&A)@!"#$#%&&A)%!"#$#%&&A)'!"#$#%&&A)&!"#$#%&&A)*!"#$#%&&A)A!"#$#%&&A>?!"#$#%&&A>( !"#$#%&&A>)!"#$#%&&A>>!"#$#%&&A>@!"#$#%&&A>%!"#$#%&&A>&!"#$#%&&A>*!"#$#%&&A>A!"#$#%&&A@?!"#$#%&&A@(!"#$#%&&A@)!"#$#%&&A@>!"#$#%&&A@@!"#$#%&&A@'!"#$#%&&A@&!"#$#%&&A@*!"#$#%&&A@A!"#$#%&&A%?!"#$#%&&A%(!"#$#%&&A%)!"#$#%&&A%>!"#$#%&&A%@!"#$#%&&A%%!"#$#%&&A%'!"#$#%&&A%& !"#$#%&&A%*!"#$#%&&A%A!"#$#%&&A'?!"#$#%&&A'(!"#$#%&&A')!"#$#%&&A'>!"#$#%&&A'@!"#$#%&&A'%!"#$#%&&A''!"#$#%&&A'&!"#$#%&&A'*!"#$#%&&A'A!"#$#%&&A&?!"#$#%&&A&(!"#$#%&&A&)!"#$#%&&A&>!"#$#%&&A&@!"#$#%&&A&%!"#$#%&&A&'!"#$#%&&A&&!"#$#%&&A&*!"#$#%&&A&A!"#$#%&&A*?!"#$#%&&A*( !"#$#%&&A*)!"#$#%&&A*>!"#$#%&&A*%!"#$#%&&A*A!"#$#%&&AA?!"#$#%&&AA(!"#$#%&&AA)!"#$#%&&AA>!"#$#%&&AA@!"#$#%&&AA'!"#$#%&&AA&!"#$#%&&AA*!"#$#%&&AAA!"#$#%&*???!"#$#%&*??(!"#$#%&*??)!"#$#%&*??>!"#$#%&*??@!"#$#%&*??%!"#$#%&*??'!"#$#%&*??&!"#$#%&*??*!"#$#%&*??A!"#$#%&*?(( !"#$#%&*?(>!"#$#%&*?(@!"#$#%&*?(%!"#$#%&*?('!"#$#%&*?(&!"#$#%&*?(*!"#$#%&*?(A!"#$#%&*?)?!"#$#%&*?)(!"#$#%&*?))!"#$#%&*?)>!"#$#%&*?)@!"#$#%&*?)%!"#$#%&*?)'!"#$#%&*?)&!"#$#%&*?)*!"#$#%&*?)A!"#$#%&*?>?!"#$#%&*?>(!"#$#%&*?>)!"#$#%&*?>>!"#$#%&*?>@!"#$#%&*?>%!"#$#%&*?>' !"#$#%&*?>&!"#$#%&*?>*!"#$#%&*?>A!"#$#%&*?@?!"#$#%&*?@(!"#$#%&*?@)!"#$#%&*?@>!"#$#%&*?@@!"#$#%&*?@%!"#$#%&*?@'!"#$#%&*?@&!"#$#%&*?@*!"#$#%&*?%?!"#$#%&*?%(!"#$#%&*?%>!"#$#%&*?%@!"#$#%&*?%%!"#$#%&*?%&!"#$#%&*?%*!"#$#%&*?%A!"#$#%&*?'?!"#$#%&*?'(!"#$#%&*?')!"#$#%&*?'> !"#$#%&*?'@!"#$#%&*?'%!"#$#%&*?''!"#$#%&*?'&!"#$#%&*?'*!"#$#%&*?'A!"#$#%&*?&?!"#$#%&*?&(!"#$#%&*?&)!"#$#%&*?&>!"#$#%&*?&@!"#$#%&*?&%!"#$#%&*?&'!"#$#%&*?&&!"#$#%&*?&*!"#$#%&*?&A

|  | 7B5(A | ! 1 7 | < 1 + 3 -7 $1-<! 1 7 4  !- +1 14 7B5(A |
| --- | --- | --- | --- |
| !"#$#%&*?*? | 7+5 7 < 1!7 2 4 | 7+5 7 < 1!7 2 4 | -41" 1 < $ + 1 +-" " "7 7 |
| !"#$#%&*?*( | 7+5 7 < 1!7 2 4 | 7+5 7 < 1!7 2 4 | -41" 1 < $ + 1 +-" " "7 7 |

# !"#$#%&*?*)!"#$#%&*?*>!"#$#%&*?*@!"#$#%&*?*%!"#$#%&*?*'!"#$#%&*?*&!"#$#%&*?**!"#$#%&*?*A!"#$#%&*?A?!"#$#%&*?A(!"#$#%&*?A)!"#$#%&*?A>!"#$#%&*?A@!"#$#%&*?A%!"#$#%&*?A'!"#$#%&*?A&!"#$#%&*?A*!"#$#%&*?AA!"#$#%&*(??!"#$#%&*(?(!"#$#%&*(?)!"#$#%&*(?>!"#$#%&*(?@!"#$#%&*(?% !"#$#%&*(?'!"#$#%&*(?&!"#$#%&*(?*!"#$#%&*(?A!"#$#%&*((?!"#$#%&*(((!"#$#%&*(()!"#$#%&*((>!"#$#%&*((@!"#$#%&*((%!"#$#%&*(('!"#$#%&*((&!"#$#%&*((*!"#$#%&*((A!"#$#%&*()?!"#$#%&*()(!"#$#%&*())!"#$#%&*()>!"#$#%&*()@!"#$#%&*()%!"#$#%&*()'!"#$#%&*()&!"#$#%&*()*!"#$#%&*()A !"#$#%&*(>?!"#$#%&*(>(!"#$#%&*(>)!"#$#%&*(>>!"#$#%&*(>@!"#$#%&*(>%!"#$#%&*(>'!"#$#%&*(>&!"#$#%&*(>*!"#$#%&*(>A!"#$#%&*(@?!"#$#%&*(@(!"#$#%&*(@)!"#$#%&*(@>!"#$#%&*(@@!"#$#%&*(@%!"#$#%&*(@'!"#$#%&*(@&!"#$#%&*(@*!"#$#%&*(@A!"#$#%&*(%?!"#$#%&*(%(!"#$#%&*(%)!"#$#%&*(%> !"#$#%&*(%@!"#$#%&*(%%!"#$#%&*(%'!"#$#%&*(%&!"#$#%&*(%*!"#$#%&*(%A!"#$#%&*('?!"#$#%&*('(!"#$#%&*(')!"#$#%&*('>!"#$#%&*('@!"#$#%&*('%!"#$#%&*(''!"#$#%&*('&

|  | | ,17 1  $ | $ $,11 | | B |
| --- | --- | --- | --- | --- | --- |
| !"#$#%&*('* | | 7+5 7 < 1!7 2 4 | 7+5 7 < 1!7 2 4 | | -41" 1 < $ + 1 +-" " "7 7 |
| !"#$#%&*('A!"#$#%&*(&? | | ,17 1 | $ $,11 | | B |
| !"#$#%&*(&(!"#$#%&*(&) !"#$#%&*(&>!"#$#%&*(&@ | $ | |  |  | | |
| !"#$#%&*(&% | 7+5 7 < 1!7 2 4 | | 7+5 7 < 1!7 2 4 | -41" 1 < $ + 1 +-" " "7 7 | | |
| !"#$#%&*(&'!"#$#%&*(&& | 73+B +50, | | 73+B +50, | < G ! 0 < < H1 < 5 0 151B < $$ D | | |
| !"#$#%&*(&*!"#$#%&*(&A !"#$#%&*(*?!"#$#%&*(*(  !"#$#%&*(*)!"#$#%&*(*> !"#$#%&*(*@ | 7+5 7 < 1!7 2 4 | | 7+5 7 < 1!7 2 4 | -41" 1 < $ + 1 +-" " "7 7 | | |
| !"#$#%&*(*% | ,17 1  $ | | $ $,11 | B | | |
| !"#$#%&*(*' | 2 D +ODW; | | 7 | 57 1 1 7 1 ;P ;5! 1 7 ";0 7 DW;" DW;1 F 1;O B D P1 7 D | | |
| !"#$#%&*(*&!"#$#%&*(** | 2 B 3 | | 7 | 57 1 1 7 1 ;P ;5! 1 7 ";0 7 DW;" DW;1 F 1;O B D P1 7 D 13 | | |
| !"#$#%&*(*A | 2 ,1 | | 7 | 57 1 1 7 1 ;P ;5! 1 7 ";0 7 DW;" DW;1 F 1;O B D P1 7 +; | | |
| !"#$#%&*(A?!"#$#%&*(A( | 2 7 L,$;< | | 7 | 57 1 1 7 1 ;P ;5! 1 7 ";0 7 DW;" DW;1 F 1;O B D P1  7 + < | | |
| !"#$#%&*(A)!"#$#%&*(A> | 2 ,1 | | 7 | 57 1 1 7 1 ;P ;5! 1 7 ";0 7 DW;" DW;1 F 1;O B D P1 7 +; | | |
| !"#$#%&*(A@ | 7 -2 ,$7 | | 7 | 57 1 1 7 1 ;P ;5! 1 7 ";0 7 DW;" DW;1 F 1;O B D P1  7 D $O; | | |
| !"#$#%&*(A% | 7 -2 | | 7 | 57 1 1 7 1 ;P ;5! 1 7 ";0 7 DW;" DW;1 F 1;O B D P1  7 1 L | | |
| !"#$#%&*(A' | 2 " | | 7 | 57 1 1 7 1 ;P ;5! 1 7 ";0 7 DW;" DW;1 F 1;O B D P1 7 D 1; | | |
| !"#$#%&*(A& | 2 " | | 7 | 57 1 1 7 1 ;P ;5! 1 7 ";0 7 DW;" DW;1 F 1;O B D P1  7 7 | | |
| !"#$#%&*(A*!"#$#%&*(AA | 2 " | | 7 | 57 1 1 7 1 ;P ;5! 1 7 ";0 7 DW;" DW;1 F 1;O B D P1 7 1 < | | |
| !"#$#%&*)??!"#$#%&*)?( | 2 " | | 7 | 57 1 1 7 1 ;P ;5! 1 7 ";0 7 DW;" DW;1 F 1;O B D P1 | | |

# 7 7

!"#$#%&*)?)!"#$#%&*)?>!"#$#%&*)?@!"#$#%&*)?%!"#$#%&*)?'!"#$#%&*)?&!"#$#%&*)?*!"#$#%&*)?A!"#$#%&*)(?!"#$#%&*)((!"#$#%&*)()!"#$#%&*)(>!"#$#%&*)(@!"#$#%&*)(%!"#$#%&*)('!"#$#%&*)(&!"#$#%&*)(*!"#$#%&*)(A!"#$#%&*))?!"#$#%&*))(!"#$#%&*)))!"#$#%&*))>!"#$#%&*))@!"#$#%&*))% !"#$#%&*))'!"#$#%&*))&!"#$#%&*))*!"#$#%&*))A!"#$#%&*)>?!"#$#%&*)@(!"#$#%&*)@)!"#$#%&*)@>!"#$#%&*)@@!"#$#%&*)@%!"#$#%&*)@'!"#$#%&*)@&!"#$#%&*)@*!"#$#%&*)@A!"#$#%&*)%?!"#$#%&*)%(!"#$#%&*)%)!"#$#%&*)%>!"#$#%&*)%@!"#$#%&*)%%!"#$#%&*)%'!"#$#%&*)%&!"#$#%&*)%*!"#$#%&*)%A

# !"#$#%&*)'?!"#$#%&*)'(!"#$#%&*)')!"#$#%&*)'>!"#$#%&*)'@!"#$#%&*)'%!"#$#%&*)''!"#$#%&*)'&!"#$#%&*)'*!"#$#%&*)'A!"#$#%&*)&?!"#$#%&*)&(!"#$#%&*)&)!"#$#%&*)&>!"#$#%&*)&@!"#$#%&*)&%!"#$#%&*)&'!"#$#%&*)&&!"#$#%&*)&*!"#$#%&*)&A!"#$#%&*)*?!"#$#%&*)*(!"#$#%&*)*)!"#$#%&*)*> !"#$#%&*)*@!"#$#%&*)*%!"#$#%&*)*'!"#$#%&*)*&!"#$#%&*)**!"#$#%&*)*A!"#$#%&*)A?!"#$#%&*)A(!"#$#%&*)A)!"#$#%&*)A>!"#$#%&*)A@!"#$#%&*)A%!"#$#%&*)A'!"#$#%&*)A&!"#$#%&*)A*!"#$#%&*)AA!"#$#%&*>??!"#$#%&*>?(!"#$#%&*>?)!"#$#%&*>?>!"#$#%&*>?@!"#$#%&*>?%!"#$#%&*>?'!"#$#%&*>?& !"#$#%&*>?*!"#$#%&*>?A!"#$#%&*>(?!"#$#%&*>((!"#$#%&*>()!"#$#%&*>(>!"#$#%&*>(@!"#$#%&*>(%!"#$#%&*>('!"#$#%&*>(&!"#$#%&*>(*!"#$#%&*>(A!"#$#%&*>)?!"#$#%&*>)(!"#$#%&*>))!"#$#%&*>)>!"#$#%&*>)@!"#$#%&*>)%!"#$#%&*>)'!"#$#%&*>)&!"#$#%&*>)*!"#$#%&*>)A!"#$#%&*>>?!"#$#%&*>>( !"#$#%&*>>)!"#$#%&*>>>!"#$#%&*>>@ 3 B +$ 3 B +$ 1 7 ; ;D ;7 7 0

!"#$#%&*>>%!"#$#%&*>>'!"#$#%&*>>&!"#$#%&*>>*!"#$#%&*>>A!"#$#%&*>@?!"#$#%&*>@(!"#$#%&*>@)!"#$#%&*>@>!"#$#%&*>@@!"#$#%&*>@%!"#$#%&*>@'!"#$#%&*>@&!"#$#%&*>@*!"#$#%&*>@A!"#$#%&*>%?!"#$#%&*>%(!"#$#%&*>%)!"#$#%&*>%>!"#$#%&*>%@!"#$#%&*>%%!"#$#%&*>%'!"#$#%&*>%&!"#$#%&*>%* !"#$#%&*>%A!"#$#%&*>'?!"#$#%&*>'(!"#$#%&*>')!"#$#%&*>'>!"#$#%&*>'@!"#$#%&*>'%!"#$#%&*>''!"#$#%&*>'&!"#$#%&*>'*!"#$#%&*>'A!"#$#%&*>&?!"#$#%&*>&(!"#$#%&*>&)!"#$#%&*>&>!"#$#%&*>&@!"#$#%&*>&%!"#$#%&*>&'!"#$#%&*>&&!"#$#%&*>&*!"#$#%&*>&A!"#$#%&*>*?!"#$#%&*>*(!"#$#%&*>*)

!"#$#%&*>*>!"#$#%&*>*@!"#$#%&*>*%!"#$#%&*>*'!"#$#%&*>*&!"#$#%&*>**!"#$#%&*>*A!"#$#%&*>A?!"#$#%&*>A(!"#$#%&*>A)!"#$#%&*>A>!"#$#%&*>A@!"#$#%&*>A%!"#$#%&*>A'!"#$#%&*>A&!"#$#%&*>A*!"#$#%&*>AA!"#$#%&*@??!"#$#%&*@?(!"#$#%&*@?)!"#$#%&*@?>!"#$#%&*@?@!"#$#%&*@?%!"#$#%&*@?' !"#$#%&*@?&!"#$#%&*@?*!"#$#%&*@?A!"#$#%&*@(?!"#$#%&*@((!"#$#%&*@()!"#$#%&*@(>!"#$#%&*@(@!"#$#%&*@(%!"#$#%&*@('!"#$#%&*@(&!"#$#%&*@(*!"#$#%&*@(A!"#$#%&*@)?!"#$#%&*@)(!"#$#%&*@))!"#$#%&*@)>!"#$#%&*@)@!"#$#%&*@)%!"#$#%&*@)'!"#$#%&*@)&!"#$#%&*@)*!"#$#%&*@)A!"#$#%&*@>? !"#$#%&*@>(!"#$#%&*@>)!"#$#%&*@>>!"#$#%&*@>@!"#$#%&*@>%!"#$#%&*@>'!"#$#%&*@>&!"#$#%&*@>*!"#$#%&*@>A!"#$#%&*@@?!"#$#%&*@@(!"#$#%&*@@)!"#$#%&*@@>!"#$#%&*@@@!"#$#%&*@@%!"#$#%&*@@'!"#$#%&*@@&!"#$#%&*@@*!"#$#%&*@@A!"#$#%&*@%?!"#$#%&*@%(!"#$#%&*@%)!"#$#%&*@%>!"#$#%&*@%@

# !"#$#%&*@%%!"#$#%&*@%'!"#$#%&*@%&!"#$#%&*@%*!"#$#%&*@%A!"#$#%&*@'?!"#$#%&*@'(!"#$#%&*@')!"#$#%&*@'>!"#$#%&*@'@!"#$#%&*@'%!"#$#%&*@''!"#$#%&*@'&!"#$#%&*@'*!"#$#%&*@'A!"#$#%&*@&?!"#$#%&*@&(!"#$#%&*@&)!"#$#%&*@&>!"#$#%&*@&@!"#$#%&*@&%!"#$#%&*@&'!"#$#%&*@&&!"#$#%&*@&*

!"#$#%&*@&A!"#$#%&*@*?!"#$#%&*@*(!"#$#%&*@*)!"#$#%&*@*>!"#$#%&*@*@!"#$#%&*@*%!"#$#%&*@*'!"#$#%&*@*&!"#$#%&*@**!"#$#%&*@*A!"#$#%&*@A?!"#$#%&*@A(!"#$#%&*@A)!"#$#%&*@A>!"#$#%&*@A@!"#$#%&*@A%!"#$#%&*@A'!"#$#%&*@A&!"#$#%&*@A*!"#$#%&*@AA!"#$#%&*%??!"#$#%&*%?(!"#$#%&*%?) !"#$#%&*%?>!"#$#%&*%?@!"#$#%&*%?%!"#$#%&*%?'!"#$#%&*%?&!"#$#%&*%?*!"#$#%&*%?A!"#$#%&*%(?!"#$#%&*%((!"#$#%&*%()!"#$#%&*%(>!"#$#%&*%(@!"#$#%&*%(%!"#$#%&*%('!"#$#%&*%(&!"#$#%&*%(*!"#$#%&*%(A!"#$#%&*%)?!"#$#%&*%)(!"#$#%&*%))!"#$#%&*%)>!"#$#%&*%)@!"#$#%&*%)%!"#$#%&*%)'

!"#$#%&*%)&!"#$#%&*%)*!"#$#%&*%)A!"#$#%&*%>?!"#$#%&*%>(!"#$#%&*%>)!"#$#%&*%>>!"#$#%&*%>@!"#$#%&*%>%!"#$#%&*%>'!"#$#%&*%>&!"#$#%&*%>*!"#$#%&*%>A!"#$#%&*%@?!"#$#%&*%@(!"#$#%&*%@)!"#$#%&*%@>!"#$#%&*%@@!"#$#%&*%@%!"#$#%&*%@'!"#$#%&*%@&!"#$#%&*%@*!"#$#%&*%@A!"#$#%&*%%? !"#$#%&*%%(!"#$#%&*%%)!"#$#%&*%%>!"#$#%&*%%@!"#$#%&*%%%!"#$#%&*%%'!"#$#%&*%%&!"#$#%&*%%*!"#$#%&*%%A!"#$#%&*%'?!"#$#%&*%'(!"#$#%&*%')!"#$#%&*%'>!"#$#%&*%'@!"#$#%&*%'%!"#$#%&*%''!"#$#%&*%'&!"#$#%&*%'*!"#$#%&*%'A!"#$#%&*%&?!"#$#%&*%&(!"#$#%&*%&)!"#$#%&*%&>!"#$#%&*%&@

# !"#$#%&*%&%!"#$#%&*%&'!"#$#%&*%&&!"#$#%&*%&*!"#$#%&*%&A!"#$#%&*%*?!"#$#%&*%*(!"#$#%&*%*)!"#$#%&*%*>!"#$#%&*%*@!"#$#%&*%*%!"#$#%&*%*'!"#$#%&*%*&!"#$#%&*%**!"#$#%&*%*A!"#$#%&*%A?!"#$#%&*%A(!"#$#%&*%A)!"#$#%&*%A>!"#$#%&*%A@!"#$#%&*%A%!"#$#%&*%A'!"#$#%&*%A&!"#$#%&*%A* !"#$#%&*%AA!"#$#%&*'??!"#$#%&*'?(!"#$#%&*'?)!"#$#%&*'?>!"#$#%&*'?@!"#$#%&*'?%!"#$#%&*'?'!"#$#%&*'?&!"#$#%&*'?*!"#$#%&*'?A!"#$#%&*'(?!"#$#%&*'((!"#$#%&*'()!"#$#%&*'(>!"#$#%&*'(@!"#$#%&*'(%!"#$#%&*'('!"#$#%&*'(&!"#$#%&*'(*!"#$#%&*'(A!"#$#%&*')?!"#$#%&*')(!"#$#%&*')) !"#$#%&*')>!"#$#%&*')@!"#$#%&*')%!"#$#%&*')'!"#$#%&*')&!"#$#%&*')*!"#$#%&*')A!"#$#%&*'>?!"#$#%&*'>(!"#$#%&*'>)!"#$#%&*'>>!"#$#%&*'>@!"#$#%&*'>%!"#$#%&*'>'!"#$#%&*'>&!"#$#%&*'>*!"#$#%&*'>A!"#$#%&*'@?!"#$#%&*'@(!"#$#%&*'@)!"#$#%&*'@>!"#$#%&*'@@!"#$#%&*'@%!"#$#%&*'@' !"#$#%&*'@&!"#$#%&*'@*!"#$#%&*'@A!"#$#%&*'%?!"#$#%&*'%(!"#$#%&*'%)!"#$#%&*'%>!"#$#%&*'%@!"#$#%&*'%%!"#$#%&*'%'!"#$#%&*'%&!"#$#%&*'%*!"#$#%&*'%A!"#$#%&*''?!"#$#%&*''(!"#$#%&*'')!"#$#%&*''>!"#$#%&*''@!"#$#%&*''%!"#$#%&*'''!"#$#%&*''&!"#$#%&*''*!"#$#%&*''A!"#$#%&*'&? !"#$#%&*'&(!"#$#%&*'&)!"#$#%&*'&>!"#$#%&*'&@!"#$#%&*'&%!"#$#%&*'&'!"#$#%&*'&&!"#$#%&*'&*!"#$#%&*'&A!"#$#%&*'*?!"#$#%&*'*(!"#$#%&*'*)!"#$#%&*'*>!"#$#%&*'*@!"#$#%&*'*%!"#$#%&*'*'!"#$#%&*'*&!"#$#%&*'**!"#$#%&*'*A!"#$#%&*'A?!"#$#%&*'A(!"#$#%&*'A)!"#$#%&*'A>!"#$#%&*'A@ !"#$#%&*'A%

|  | $2 7 | $27 | 4 + 0 7 |
| --- | --- | --- | --- |
| !"#$#%&*'A& | 7 B $ $  < | $<5< | ! + "+ D1 B |
| !"#$#%&*'A* | $,2!B 9$ | 1 : 7 | 1BD" 4 + 1 ;< 524;F 8 7 4 1D D B 7D B |
| !"#$#%&*'AA!"#$#%&*&?? | $,2!B 9$ | 1 : 7 | D" 4 + 1B1 ;< 524;F 8 7 4 1D D B 7D B |
| !"#$#%&*&?(!"#$#%&*&?) | 1 "  " 24 C2 | 1 " " 24 C2 | 7 2,72328$13 28 1 $1D 9 F!!9 $ 723! 4 1 |

# !"#$#%&*&?@!"#$#%&*&?%!"#$#%&*&?'!"#$#%&*&?&!"#$#%&*&?*!"#$#%&*&?A!"#$#%&*&(?!"#$#%&*&((!"#$#%&*&()!"#$#%&*&(>!"#$#%&*&(@!"#$#%&*&(%!"#$#%&*&('!"#$#%&*&(&!"#$#%&*&(*!"#$#%&*&(A!"#$#%&*&)?!"#$#%&*&)(!"#$#%&*&))!"#$#%&*&)>!"#$#%&*&)@!"#$#%&*&)%!"#$#%&*&)'!"#$#%&*&)& $,2!B 9$ 1 : 7 1BD" 4 + 1 ;< 524;F 8 7 4 1D D B 7D B !"#$#%&*&)*!"#$#%&*&)A!"#$#%&*&>?!"#$#%&*&>(!"#$#%&*&>)!"#$#%&*&>>!"#$#%&*&>@!"#$#%&*&>%!"#$#%&*&>'!"#$#%&*&>&!"#$#%&*&>*!"#$#%&*&>A!"#$#%&*&@?!"#$#%&*&@(!"#$#%&*&@)!"#$#%&*&@>!"#$#%&*&@@!"#$#%&*&@%!"#$#%&*&@'!"#$#%&*&@&!"#$#%&*&@*!"#$#%&*&@A!"#$#%&*&%?!"#$#%&*&%( !"#$#%&*&%) $,2!B 9$ 1 : 7 + D" 4 1B1 ;< 524;F 8 7 4 1D D B 7D B

!"#$#%&*&%>!"#$#%&*&%@!"#$#%&*&%%!"#$#%&*&%'!"#$#%&*&%&!"#$#%&*&%*!"#$#%&*&%A!"#$#%&*&'?!"#$#%&*&'(!"#$#%&*&')!"#$#%&*&'>!"#$#%&*&'@!"#$#%&*&'%!"#$#%&*&''!"#$#%&*&'&!"#$#%&*&'*!"#$#%&*&'A!"#$#%&*&&?!"#$#%&*&&(!"#$#%&*&&)!"#$#%&*&&>!"#$#%&*&&@!"#$#%&*&&%!"#$#%&*&&' !"#$#%&*&&&!"#$#%&*&&*!"#$#%&*&&A!"#$#%&*&*?!"#$#%&*&*(!"#$#%&*&*)!"#$#%&*&*>!"#$#%&*&*@!"#$#%&*&*%!"#$#%&*&*'!"#$#%&*&*&!"#$#%&*&**!"#$#%&*&*A!"#$#%&*&A?!"#$#%&*&A(!"#$#%&*&A)!"#$#%&*&A>!"#$#%&*&A@!"#$#%&*&A%!"#$#%&*&A'!"#$#%&*&A&!"#$#%&*&A*!"#$#%&*&AA!"#$#%&**??

# !"#$#%&**?(!"#$#%&**?)!"#$#%&**?>!"#$#%&**?@!"#$#%&**?%!"#$#%&**?'!"#$#%&**?&!"#$#%&**?*!"#$#%&**?A!"#$#%&**(?!"#$#%&**((!"#$#%&**()!"#$#%&**(>!"#$#%&**(@!"#$#%&**(%!"#$#%&**('!"#$#%&**(&!"#$#%&**(*!"#$#%&**(A!"#$#%&**)?!"#$#%&**)(!"#$#%&**))!"#$#%&**)>!"#$#%&**)@ !"#$#%&**)%!"#$#%&**)'!"#$#%&**)&!"#$#%&**)*!"#$#%&**)A!"#$#%&**>?!"#$#%&**>(!"#$#%&**>)!"#$#%&**>>!"#$#%&**>@!"#$#%&**>%!"#$#%&**>'!"#$#%&**>&!"#$#%&**>*!"#$#%&**>A!"#$#%&**@?!"#$#%&**@(!"#$#%&**@)!"#$#%&**@>!"#$#%&**@@!"#$#%&**@%!"#$#%&**@'!"#$#%&**@&!"#$#%&**@* !"#$#%&**@A!"#$#%&**%?!"#$#%&**%(!"#$#%&**%)!"#$#%&**%>!"#$#%&**%@!"#$#%&**%%!"#$#%&**%'!"#$#%&**%&!"#$#%&**%*!"#$#%&**%A!"#$#%&**'?!"#$#%&**'(!"#$#%&**')!"#$#%&**'>!"#$#%&**'@!"#$#%&**'%!"#$#%&**''!"#$#%&**'&!"#$#%&**'*!"#$#%&**'A!"#$#%&**&?!"#$#%&**&(!"#$#%&**&) !"#$#%&**&>!"#$#%&**&@!"#$#%&**&%!"#$#%&**&'!"#$#%&**&&!"#$#%&**&*!"#$#%&**&A!"#$#%&***?!"#$#%&***(!"#$#%&***)!"#$#%&***>!"#$#%&***@!"#$#%&***%!"#$#%&***'!"#$#%&***&!"#$#%&****!"#$#%&***A!"#$#%&**A?!"#$#%&**A(!"#$#%&**A)!"#$#%&**A>!"#$#%&**A@!"#$#%&**A%!"#$#%&**A'

!"#$#%&**A&!"#$#%&**A*!"#$#%&**AA!"#$#%&*A??!"#$#%&*A?(!"#$#%&*A?)!"#$#%&*A?>!"#$#%&*A?@!"#$#%&*A?%!"#$#%&*A?'!"#$#%&*A?&!"#$#%&*A?*!"#$#%&*A?A!"#$#%&*A(?!"#$#%&*A((!"#$#%&*A()!"#$#%&*A(>!"#$#%&*A(@!"#$#%&*A(%!"#$#%&*A('!"#$#%&*A(&!"#$#%&*A(*!"#$#%&*A(A!"#$#%&*A)? !"#$#%&*A)(!"#$#%&*A))!"#$#%&*A)>!"#$#%&*A)@!"#$#%&*A)%!"#$#%&*A)'!"#$#%&*A)&!"#$#%&*A)*!"#$#%&*A)A!"#$#%&*A>?!"#$#%&*A>(!"#$#%&*A>)!"#$#%&*A>>!"#$#%&*A>@!"#$#%&*A>%!"#$#%&*A>'!"#$#%&*A>&!"#$#%&*A>*!"#$#%&*A>A!"#$#%&*A@?!"#$#%&*A@(!"#$#%&*A@)!"#$#%&*A@>!"#$#%&*A@@ !"#$#%&*A@%!"#$#%&*A@'!"#$#%&*A@&!"#$#%&*A@*!"#$#%&*A@A!"#$#%&*A%?!"#$#%&*A%(!"#$#%&*A%)!"#$#%&*A%>!"#$#%&*A%@!"#$#%&*A%%!"#$#%&*A%'!"#$#%&*A%&!"#$#%&*A%*!"#$#%&*A%A!"#$#%&*A'?!"#$#%&*A'(!"#$#%&*A')!"#$#%&*A'>!"#$#%&*A'@!"#$#%&*A'%!"#$#%&*A''!"#$#%&*A'&!"#$#%&*A'*

# !"#$#%&*A'A!"#$#%&*A&?!"#$#%&*A&(!"#$#%&*A&)!"#$#%&*A&>!"#$#%&*A&@!"#$#%&*A&%!"#$#%&*A&'!"#$#%&*A&&!"#$#%&*A&*!"#$#%&*A&A!"#$#%&*A*?!"#$#%&*A*(!"#$#%&*A*)!"#$#%&*A*>!"#$#%&*A*@!"#$#%&*A*%!"#$#%&*A*'!"#$#%&*A*&!"#$#%&*A**!"#$#%&*A*A!"#$#%&*AA?!"#$#%&*AA(!"#$#%&*AA)

!"#$#%&*AA>!"#$#%&*AA@!"#$#%&*AA%!"#$#%&*AA'!"#$#%&*AA&!"#$#%&*AA*!"#$#%&*AAA!"#$#%&A???!"#$#%&A??(!"#$#%&A??)!"#$#%&A??>!"#$#%&A??@!"#$#%&A??%!"#$#%&A??'!"#$#%&A??&!"#$#%&A??*!"#$#%&A??A!"#$#%&A?(?!"#$#%&A?((!"#$#%&A?()!"#$#%&A?(>!"#$#%&A?(@!"#$#%&A?(%!"#$#%&A?(' !"#$#%&A?(&!"#$#%&A?(*!"#$#%&A?(A!"#$#%&A?)?!"#$#%&A?)(!"#$#%&A?))!"#$#%&A?)>!"#$#%&A?)@!"#$#%&A?)%!"#$#%&A?)'!"#$#%&A?)&!"#$#%&A?)*!"#$#%&A?)A!"#$#%&A?>?!"#$#%&A?>(!"#$#%&A?>)!"#$#%&A?>>!"#$#%&A?>@!"#$#%&A?>%!"#$#%&A?>'!"#$#%&A?>&!"#$#%&A?>*!"#$#%&A?>A!"#$#%&A?@? !"#$#%&A?@(!"#$#%&A?@)!"#$#%&A?@>!"#$#%&A?@@!"#$#%&A?@%!"#$#%&A?@'!"#$#%&A?@&!"#$#%&A?@*!"#$#%&A?@A!"#$#%&A?%?!"#$#%&A?%(!"#$#%&A?%)!"#$#%&A?%>!"#$#%&A?%@!"#$#%&A?%%!"#$#%&A?%'!"#$#%&A?%&

|  | $,2!B 9$ | 1 : 7 | D" 4 + 1B1 ;< 524;F 8 7 4 1D D B 7D B |
| --- | --- | --- | --- |
| !"#$#%&A?%* | $9 | ! +.!+/ | J +13 01 0 D2";--$ ,71 2  1 2D,D D0D 2D ! 1 1<  1+1H< 1 1 < +D1 1 9 9< BB" B2! $ D D$ |
| !"#$#%&A?%A | $"$, | ! +.!+/ | J +13 01 0 D2";--$ ,71 2  1 2D,D D0D 2D ! 1 1<  1+1H< 1 1 < +D1 1 9 9< BB" B2! $ D D$ |

# !"#$#%&A?'?!"#$#%&A?'(!"#$#%&A?')!"#$#%&A?'>!"#$#%&A?'@!"#$#%&A?'%!"#$#%&A?''!"#$#%&A?'&!"#$#%&A?'*!"#$#%&A?'A!"#$#%&A?&?!"#$#%&A?&(!"#$#%&A?&)!"#$#%&A?&>!"#$#%&A?&@!"#$#%&A?&%!"#$#%&A?&&!"#$#%&A?&*!"#$#%&A?&A!"#$#%&A?*?!"#$#%&A?*(!"#$#%&A?*)!"#$#%&A?*>!"#$#%&A?*@ !"#$#%&A?*%!"#$#%&A?*'!"#$#%&A?*&!"#$#%&A?**!"#$#%&A?*A!"#$#%&A?A?!"#$#%&A?A)

|  | | 7 2 $ | ! +.!+/ | J +13 01 0 D2";--$ ,71 2  1 2D,D D0D 2D ! 1 1<  1+1H< 1 1 < +D1 1 9 9< BB" B2! $ D D$ |
| --- | --- | --- | --- | --- |
| !"#$#%&A?A>!"#$#%&A?A@ !"#$#%&A?A%!"#$#%&A?A' | | 32 | ! +.!+/ | J +13 01 0 D2";--$ ,71 2  1 2D,D D0D 2D ! 1 1<  1+1H< 1 1 < +D1 1 9 9< BB" B2! $ D D$ |
| !"#$#%&A?A&!"#$#%&A?A* | | $"$, | ! +.!+/ | J +13 01 0 D2";--$ ,71 2  1 2D,D D0D 2D ! 1 1<  1+1H< 1 1 < +D1 1 9 9< BB" B2! $ D D$ |
| !"#$#%&A?AA | | $9 | ! +.!+/ | J +13 01 0 D2";--$ ,71 2  1 2D,D D0D 2D ! 1 1<  1+1H< 1 1 < +D1 1 9 9< BB" B2! $ D D$ |
| !"#$#%&A(??!"#$#%&A(?(  !"#$#%&A(?)!"#$#%&A(?> !"#$#%&A(?@ | | 1 2 | ! +.!+/ | J +13 01 0 D2";--$ ,71 2  1 2D,D D0D 2D ! 1 1<  1+1H< 1 1 < +D1 1 9 9< BB" B2! $ D D$ |
| !"#$#%&A(?%!"#$#%&A(?'!"#$#%&A(?&!"#$#%&A(?*!"#$#%&A(?A!"#$#%&A((?!"#$#%&A(((!"#$#%&A(()!"#$#%&A((>!"#$#%&A((%!"#$#%&A(('!"#$#%&A((& | | | |
|  | $"$, | | ! +.!+/ | J +13 01 0 D2";--$ ,71 2  1 2D,D D0D 2D ! 1 1<  1+1H< 1 1 < +D1 1 9 9< BB" B2! $ D D$ |
| !"#$#%&A((* | 2 | | ! +.!+/ | J +13 01 0 D2";--$ ,71 2  1 2D,D D0D 2D ! 1 1<  1+1H< 1 1 < +D1 1 9 9< BB" B2! $ D D$ |
| !"#$#%&A((A!"#$#%&A()? | $"$, | | ! +.!+/ | J +13 01 0 D2";--$ ,71 2  1 2D,D D0D 2D ! 1 1<  1+1H< 1 1 < +D1 1 9 9< BB" B2! $ D D$ |
| !"#$#%&A()( | 2 | | ! +.!+/ | J +13 01 0 D2";--$ ,71 2  1 2D,D D0D 2D ! 1 1<  1+1H< 1 1 < +D1 1 9 9< BB" B2! $ D D$ |
| !"#$#%&A())!"#$#%&A()> | $"$, | | ! +.!+/ | J +13 01 0 D2";--$ ,71 2  1 2D,D D0D 2D ! 1 1<  1+1H< 1 1 < +D1 1 9 9< BB" B2! $ D D$ |
| !"#$#%&A()%!"#$#%&A()' !"#$#%&A()&!"#$#%&A()* | 7 $ | | ! +.!+/ | J +13 01 0 D2";--$ ,71 2  1 2D,D D0D 2D ! 1 1<  1+1H< 1 1 < +D1 1 9 9< BB" B2! $ D D$ |
| !"#$#%&A()A!"#$#%&A(>? !"#$#%&A(>(!"#$#%&A(>) | 1 2 | | ! +.!+/ | J +13 01 0 D2";--$ ,71 2 1 2D,D D0D 2D ! 1 1< |

# 1+1H< 1 1 < +D1 1 9 9< BB" B2! $ D D$ !"#$#%&A(>>!"#$#%&A(>@!"#$#%&A(>%!"#$#%&A(>'!"#$#%&A(>*!"#$#%&A(>A!"#$#%&A(@?!"#$#%&A(@(!"#$#%&A(@)!"#$#%&A(@>!"#$#%&A(@@!"#$#%&A(@%!"#$#%&A(@'!"#$#%&A(@&!"#$#%&A(@*!"#$#%&A(@A!"#$#%&A(%?!"#$#%&A(%(!"#$#%&A(%)!"#$#%&A(%>!"#$#%&A(%@!"#$#%&A(%%

|  | 7 $ | ! +.!+/ | J +13 01 0 D2";--$ ,71 2  1 2D,D D0D 2D ! 1 1<  1+1H< 1 1 < +D1 1 9 9< BB" B2! $ D D$ |
| --- | --- | --- | --- |
| !"#$#%&A(%' | $"$, | ! +.!+/ | J +13 01 0 D2";--$ ,71 2 |

# 1 2D,D D0D 2D ! 1 1< 1+1H< 1 1 < +D1 1 9 9< BB" B2! $ D D$ !"#$#%&A(%&!"#$#%&A(%*!"#$#%&A(%A!"#$#%&A('?!"#$#%&A('(!"#$#%&A(')!"#$#%&A('>!"#$#%&A('%!"#$#%&A(''!"#$#%&A('&!"#$#%&A('*!"#$#%&A('A!"#$#%&A(&?!"#$#%&A(&(!"#$#%&A(&)!"#$#%&A(&>!"#$#%&A(&@!"#$#%&A(&%!"#$#%&A(&'!"#$#%&A(&&!"#$#%&A(&*!"#$#%&A(&A!"#$#%&A(*?!"#$#%&A(*( !"#$#%&A(*)!"#$#%&A(*>!"#$#%&A(*@!"#$#%&A(*%!"#$#%&A(*'!"#$#%&A(*&!"#$#%&A(**!"#$#%&A(*A 7 $ ! +.!+/ J +13 01 0 D2";--$ ,71 2 1 2D,D D0D 2D ! 1 1< 1+1H< 1 1 < +D1 1 9 9< BB" B2! $ D D$ !"#$#%&A(A?!"#$#%&A(A)!"#$#%&A(A>!"#$#%&A(A@!"#$#%&A(A%!"#$#%&A(A'!"#$#%&A(A&!"#$#%&A(A*!"#$#%&A(AA!"#$#%&A)??!"#$#%&A)?(!"#$#%&A)?)!"#$#%&A)?>!"#$#%&A)?@!"#$#%&A)?%!"#$#%&A)?'!"#$#%&A)?&!"#$#%&A)?*!"#$#%&A)?A!"#$#%&A)(?!"#$#%&A)((!"#$#%&A)()!"#$#%&A)(>!"#$#%&A)(@ !"#$#%&A)(%!"#$#%&A)('!"#$#%&A)(&!"#$#%&A)(*

|  | 7$.3/ | ! +.!+/ | J +13 01 0 D2";--$ ,71 2  1 2D,D D0D 2D ! 1 1<  1+1H< 1 1 < +D1 1 9 9< BB" B2! $ D D$ |
| --- | --- | --- | --- |
| !"#$#%&A))?!"#$#%&A))>  !"#$#%&A))%!"#$#%&A))' !"#$#%&A))& | 7 2 $ | ! +.!+/ | J +13 01 0 D2";--$ ,71 2  1 2D,D D0D 2D ! 1 1<  1+1H< 1 1 < +D1 1 9 9< BB" B2! $ D D$ |
| !"#$#%&A))*!"#$#%&A))A  !"#$#%&A)>?!"#$#%&A)>( !"#$#%&A)>) | 1 2 | ! +.!+/ | J +13 01 0 D2";--$ ,71 2  1 2D,D D0D 2D ! 1 1<  1+1H< 1 1 < +D1 1 9 9 |

# < BB" B2! $ D D$

!"#$#%&A)>>!"#$#%&A)>@!"#$#%&A)>'!"#$#%&A)>&!"#$#%&A)>*!"#$#%&A)>A!"#$#%&A)@?!"#$#%&A)@(!"#$#%&A)@)!"#$#%&A)@>!"#$#%&A)@@!"#$#%&A)@%!"#$#%&A)@'!"#$#%&A)@&!"#$#%&A)@*!"#$#%&A)@A!"#$#%&A)%?!"#$#%&A)%(!"#$#%&A)%)!"#$#%&A)%>!"#$#%&A)%@!"#$#%&A)%'!"#$#%&A)%*!"#$#%&A)%A !"#$#%&A)'?!"#$#%&A)'(!"#$#%&A)')!"#$#%&A)'>!"#$#%&A)'@!"#$#%&A)'%!"#$#%&A)''!"#$#%&A)'&!"#$#%&A)'*!"#$#%&A)'A!"#$#%&A)&?!"#$#%&A)&(!"#$#%&A)&)!"#$#%&A)&>!"#$#%&A)&@!"#$#%&A)&'!"#$#%&A)&&!"#$#%&A)&*!"#$#%&A)&A!"#$#%&A)*?!"#$#%&A)*(!"#$#%&A)*)!"#$#%&A)*>!"#$#%&A)*@

# !"#$#%&A)*'!"#$#%&A)*&!"#$#%&A)**!"#$#%&A)*A!"#$#%&A)A?!"#$#%&A)A(!"#$#%&A)A)!"#$#%&A)A> $"$, ! +.!+/ J +13 01 0 D2";--$ ,71 2 1 2D,D D0D 2D ! 1 1< 1+1H< 1 1 < +D1 1 9 9< BB" B2! $ D D$ !"#$#%&A)A@!"#$#%&A)A%!"#$#%&A)A'!"#$#%&A)A&!"#$#%&A)A*!"#$#%&A)AA!"#$#%&A>??!"#$#%&A>?(!"#$#%&A>?)!"#$#%&A>?>!"#$#%&A>?@!"#$#%&A>?%!"#$#%&A>?'!"#$#%&A>?&!"#$#%&A>?*!"#$#%&A>?A!"#$#%&A>(?!"#$#%&A>(>!"#$#%&A>(@ 1 2 ! +.!+/ J +13 01 0 D2";--$ ,71 2 1 2D,D D0D 2D ! 1 1< 1+1H< 1 1 < +D1 1 9 9< BB" B2! $ D D$

!"#$#%&A>(%!"#$#%&A>('!"#$#%&A>(&!"#$#%&A>(*!"#$#%&A>(A!"#$#%&A>)?!"#$#%&A>)(!"#$#%&A>))!"#$#%&A>)>!"#$#%&A>)%!"#$#%&A>)'!"#$#%&A>)*!"#$#%&A>)A!"#$#%&A>>?!"#$#%&A>>(!"#$#%&A>>)!"#$#%&A>>>!"#$#%&A>>@!"#$#%&A>>%!"#$#%&A>>'!"#$#%&A>>&!"#$#%&A>>*!"#$#%&A>@?!"#$#%&A>@( !"#$#%&A>@@!"#$#%&A>@%!"#$#%&A>@'!"#$#%&A>@&!"#$#%&A>@*!"#$#%&A>@A!"#$#%&A>%?!"#$#%&A>%(!"#$#%&A>%)!"#$#%&A>%>!"#$#%&A>%@!"#$#%&A>%%!"#$#%&A>%'!"#$#%&A>%&!"#$#%&A>%*!"#$#%&A>%A!"#$#%&A>'?!"#$#%&A>'(!"#$#%&A>')!"#$#%&A>'>!"#$#%&A>'@!"#$#%&A>'%!"#$#%&A>''!"#$#%&A>'&

!"#$#%&A>'*!"#$#%&A>&?!"#$#%&A>&(!"#$#%&A>&)!"#$#%&A>&@!"#$#%&A>&%!"#$#%&A>&'!"#$#%&A>&&!"#$#%&A>&* $"$, ! +.!+/ J +13 01 0 D2";--$ ,71 2

# 1 2D,D D0D 2D ! 1 1< 1+1H< 1 1 < +D1 1 9 9< BB" B2! $ D D$ !"#$#%&A>&A!"#$#%&A>*?!"#$#%&A>*(!"#$#%&A>*)!"#$#%&A>*>!"#$#%&A>*@!"#$#%&A>*%!"#$#%&A>*&!"#$#%&A>**!"#$#%&A>*A!"#$#%&A>A?!"#$#%&A>A(!"#$#%&A>A)!"#$#%&A>A@!"#$#%&A>A%!"#$#%&A>A'!"#$#%&A>A&!"#$#%&A>A*!"#$#%&A>AA!"#$#%&A@??!"#$#%&A@?)

|  | 7$.3/ | ! +.!+/ | J +13 01 0 D2";--$ ,71 2  1 2D,D D0D 2D ! 1 1<  1+1H< 1 1 < +D1 1 9 9< BB" B2! $ D D$ |
| --- | --- | --- | --- |
| !"#$#%&A@?> | $"$, | ! +.!+/ | J +13 01 0 D2";--$ ,71 2  1 2D,D D0D 2D ! 1 1<  1+1H< 1 1 < +D1 1 9 9< BB" B2! $ D D$ |
| !"#$#%&A@?@ | 1 2 | ! +.!+/ | J +13 01 0 D2";--$ ,71 2  1 2D,D D0D 2D ! 1 1<  1+1H< 1 1 < +D1 1 9 9< BB" B2! $ D D$ |
| !"#$#%&A@?% | $"$, | ! +.!+/ | J +13 01 0 D2";--$ ,71 2  1 2D,D D0D 2D ! 1 1<  1+1H< 1 1 < +D1 1 9 9< BB" B2! $ D D$ |
| !"#$#%&A@?'!"#$#%&A@?& | 32 | ! +.!+/ | J +13 01 0 D2";--$ ,71 2  1 2D,D D0D 2D ! 1 1<  1+1H< 1 1 < +D1 1 9 9< BB" B2! $ D D$ |
| !"#$#%&A@?*!"#$#%&A@?A !"#$#%&A@(?!"#$#%&A@((  !"#$#%&A@()!"#$#%&A@(> !"#$#%&A@(@ | 7 2 $ | ! +.!+/ | J +13 01 0 D2";--$ ,71 2  1 2D,D D0D 2D ! 1 1<  1+1H< 1 1 < +D1 1 9 9< BB" B2! $ D D$ |
| !"#$#%&A@(%!"#$#%&A@(' !"#$#%&A@(& | $"$, | ! +.!+/ | J +13 01 0 D2";--$ ,71 2  1 2D,D D0D 2D ! 1 1<  1+1H< 1 1 < +D1 1 9 9< BB" B2! $ D D$ |
| !"#$#%&A@(*!"#$#%&A@(A !"#$#%&A@)? | 7$.3/ | ! +.!+/ | J +13 01 0 D2";--$ ,71 2  1 2D,D D0D 2D ! 1 1<  1+1H< 1 1 < +D1 1 9 9< BB" B2! $ D D$ |
| !"#$#%&A@)(!"#$#%&A@)) !"#$#%&A@)>!"#$#%&A@)@ | $9 | ! +.!+/ | J +13 01 0 D2";--$ ,71 2  1 2D,D D0D 2D ! 1 1<  1+1H< 1 1 < +D1 1 9 9< BB" B2! $ D D$ |
| !"#$#%&A@)% | $"$, | ! +.!+/ | J +13 01 0 D2";--$ ,71 2 |

# 1 2D,D D0D 2D ! 1 1< 1+1H< 1 1 < +D1 1 9 9< BB" B2! $ D D$

!"#$#%&A@)'!"#$#%&A@)&!"#$#%&A@)*!"#$#%&A@)A!"#$#%&A@>?!"#$#%&A@>(!"#$#%&A@>)!"#$#%&A@>>!"#$#%&A@>@!"#$#%&A@>%!"#$#%&A@>'!"#$#%&A@>&!"#$#%&A@>*!"#$#%&A@>A!"#$#%&A@@?!"#$#%&A@@(!"#$#%&A@@)!"#$#%&A@@>!"#$#%&A@@@!"#$#%&A@@%!"#$#%&A@@'!"#$#%&A@@&!"#$#%&A@@*!"#$#%&A@%? !"#$#%&A@%(!"#$#%&A@%)!"#$#%&A@%>!"#$#%&A@%@!"#$#%&A@%%!"#$#%&A@%'!"#$#%&A@%&!"#$#%&A@%*!"#$#%&A@%A!"#$#%&A@'?!"#$#%&A@'(!"#$#%&A@')!"#$#%&A@'>!"#$#%&A@'@!"#$#%&A@'%!"#$#%&A@''!"#$#%&A@'&!"#$#%&A@'*!"#$#%&A@'A!"#$#%&A@&?!"#$#%&A@&(!"#$#%&A@&>!"#$#%&A@&@!"#$#%&A@&%

# !"#$#%&A@&'!"#$#%&A@&&!"#$#%&A@&*!"#$#%&A@&A!"#$#%&A@*?!"#$#%&A@*(!"#$#%&A@*)!"#$#%&A@*>!"#$#%&A@*@!"#$#%&A@*%!"#$#%&A@*'!"#$#%&A@*&!"#$#%&A@**!"#$#%&A@*A!"#$#%&A@A(!"#$#%&A@A)!"#$#%&A@A>!"#$#%&A@A@!"#$#%&A@A%!"#$#%&A@A'!"#$#%&A@A&!"#$#%&A@A*!"#$#%&A@AA!"#$#%&A%?? !"#$#%&A%?(!"#$#%&A%?>!"#$#%&A%?@!"#$#%&A%?%!"#$#%&A%?'

|  | 7 2 $ | ! +.!+/ | J +13 01 0 D2";--$ ,71 2  1 2D,D D0D 2D ! 1 1<  1+1H< 1 1 < +D1 1 9 9< BB" B2! $ D D$ |
| --- | --- | --- | --- |
| !"#$#%&A%?&!"#$#%&A%?*  !"#$#%&A%?A!"#$#%&A%(?  !"#$#%&A%()!"#$#%&A%(> | 7$.3/ | ! +.!+/ | J +13 01 0 D2";--$ ,71 2  1 2D,D D0D 2D ! 1 1<  1+1H< 1 1 < +D1 1 9 9< BB" B2! $ D D$ |
| !"#$#%&A%(@ | $"$, | ! +.!+/ | J +13 01 0 D2";--$ ,71 2 |

# 1 2D,D D0D 2D ! 1 1< 1+1H< 1 1 < +D1 1 9 9< BB" B2! $ D D$

!"#$#%&A%(%!"#$#%&A%('!"#$#%&A%(&!"#$#%&A%(*!"#$#%&A%(A!"#$#%&A%)?!"#$#%&A%)(!"#$#%&A%))!"#$#%&A%)>!"#$#%&A%)@!"#$#%&A%)%!"#$#%&A%)'!"#$#%&A%)&!"#$#%&A%)*!"#$#%&A%)A!"#$#%&A%>?!"#$#%&A%>(!"#$#%&A%>)!"#$#%&A%>>!"#$#%&A%>@!"#$#%&A%>%!"#$#%&A%>'!"#$#%&A%>&!"#$#%&A%>* !"#$#%&A%>A!"#$#%&A%@?!"#$#%&A%@(!"#$#%&A%@)!"#$#%&A%@>!"#$#%&A%@@!"#$#%&A%@%!"#$#%&A%@'!"#$#%&A%@&!"#$#%&A%@*!"#$#%&A%@A!"#$#%&A%%?!"#$#%&A%%(!"#$#%&A%%)!"#$#%&A%%>!"#$#%&A%%@!"#$#%&A%%%!"#$#%&A%%'!"#$#%&A%%&!"#$#%&A%%*!"#$#%&A%%A!"#$#%&A%'?!"#$#%&A%'(!"#$#%&A%')

!"#$#%&A%'>!"#$#%&A%'@!"#$#%&A%'%!"#$#%&A%''!"#$#%&A%'&!"#$#%&A%'*!"#$#%&A%'A!"#$#%&A%&?!"#$#%&A%&(!"#$#%&A%&)!"#$#%&A%&>!"#$#%&A%&@!"#$#%&A%&%!"#$#%&A%&'!"#$#%&A%&&!"#$#%&A%&*!"#$#%&A%&A!"#$#%&A%*?!"#$#%&A%*(!"#$#%&A%*)!"#$#%&A%*>!"#$#%&A%*@!"#$#%&A%*%!"#$#%&A%*' !"#$#%&A%*&!"#$#%&A%**!"#$#%&A%*A!"#$#%&A%A?!"#$#%&A%A(!"#$#%&A%A)!"#$#%&A%A>!"#$#%&A%A@!"#$#%&A%A%!"#$#%&A%A'!"#$#%&A%A&!"#$#%&A%A*!"#$#%&A%AA!"#$#%&A'??!"#$#%&A'?(!"#$#%&A'?)!"#$#%&A'?>!"#$#%&A'?@!"#$#%&A'?%!"#$#%&A'?'!"#$#%&A'?&!"#$#%&A'?*!"#$#%&A'?A!"#$#%&A'(?

!"#$#%&A'((!"#$#%&A'()!"#$#%&A'(>!"#$#%&A'(@!"#$#%&A'(%!"#$#%&A'('!"#$#%&A'(&!"#$#%&A'(*!"#$#%&A'(A!"#$#%&A')?!"#$#%&A')(!"#$#%&A'))!"#$#%&A')>!"#$#%&A')@!"#$#%&A')%!"#$#%&A')'!"#$#%&A')&!"#$#%&A')*!"#$#%&A')A!"#$#%&A'>?!"#$#%&A'>(!"#$#%&A'>)!"#$#%&A'>>!"#$#%&A'>@ !"#$#%&A'>%!"#$#%&A'>'!"#$#%&A'>&!"#$#%&A'>*!"#$#%&A'>A!"#$#%&A'@?!"#$#%&A'@(!"#$#%&A'@)!"#$#%&A'@>!"#$#%&A'@@!"#$#%&A'@%!"#$#%&A'@'!"#$#%&A'@&!"#$#%&A'@*!"#$#%&A'@A!"#$#%&A'%?!"#$#%&A'%(!"#$#%&A'%)!"#$#%&A'%>!"#$#%&A'%@!"#$#%&A'%%!"#$#%&A'%'!"#$#%&A'%&!"#$#%&A'%* !"#$#%&A'%A!"#$#%&A''?!"#$#%&A''(!"#$#%&A'')!"#$#%&A''>!"#$#%&A''@!"#$#%&A''%!"#$#%&A'''!"#$#%&A''&!"#$#%&A''*!"#$#%&A''A!"#$#%&A'&?!"#$#%&A'&(!"#$#%&A'&)!"#$#%&A'&>!"#$#%&A'&@!"#$#%&A'&%!"#$#%&A'&'!"#$#%&A'&&!"#$#%&A'&*!"#$#%&A'&A!"#$#%&A'*?!"#$#%&A'*(!"#$#%&A'*)

!"#$#%&A'*>!"#$#%&A'*@!"#$#%&A'*%!"#$#%&A'*'!"#$#%&A'*&!"#$#%&A'**!"#$#%&A'*A!"#$#%&A'A?!"#$#%&A'A(!"#$#%&A'A)!"#$#%&A'A>!"#$#%&A'A@!"#$#%&A'A%!"#$#%&A'A'!"#$#%&A'A&!"#$#%&A'A*!"#$#%&A'AA!"#$#%&A&??!"#$#%&A&?(!"#$#%&A&?)!"#$#%&A&?>!"#$#%&A&?@!"#$#%&A&?%!"#$#%&A&?' !"#$#%&A&?&!"#$#%&A&?*!"#$#%&A&?A!"#$#%&A&(?!"#$#%&A&((!"#$#%&A&()!"#$#%&A&(>!"#$#%&A&(@!"#$#%&A&(%!"#$#%&A&('!"#$#%&A&(&!"#$#%&A&(*!"#$#%&A&(A!"#$#%&A&)?!"#$#%&A&)(!"#$#%&A&))!"#$#%&A&)>!"#$#%&A&)@!"#$#%&A&)%!"#$#%&A&)'!"#$#%&A&)&!"#$#%&A&)*!"#$#%&A&)A!"#$#%&A&>? !"#$#%&A&>(!"#$#%&A&>)!"#$#%&A&>>!"#$#%&A&>@!"#$#%&A&>%!"#$#%&A&>'!"#$#%&A&>&!"#$#%&A&>*!"#$#%&A&>A!"#$#%&A&@?!"#$#%&A&@(!"#$#%&A&@)!"#$#%&A&@>!"#$#%&A&@@!"#$#%&A&@%!"#$#%&A&@'!"#$#%&A&@&!"#$#%&A&@*!"#$#%&A&@A!"#$#%&A&%?!"#$#%&A&%(!"#$#%&A&%)!"#$#%&A&%>!"#$#%&A&%@ !"#$#%&A&%%!"#$#%&A&%'!"#$#%&A&%&!"#$#%&A&%*

# G!2 7 3 1 $.31$/ 1-9" 12 ! +2 ;+ 1 $D93 4H1B 92D$< 3 <8$9<773X " 2 7X773 !"#$#%&A&%A!"#$#%&A&'?!"#$#%&A&'(!"#$#%&A&')!"#$#%&A&'>!"#$#%&A&'@!"#$#%&A&'%!"#$#%&A&''!"#$#%&A&'&!"#$#%&A&'*!"#$#%&A&'A!"#$#%&A&&?!"#$#%&A&&(!"#$#%&A&&)!"#$#%&A&&>!"#$#%&A&&@!"#$#%&A&&%!"#$#%&A&&'!"#$#%&A&&&!"#$#%&A&&*!"#$#%&A&&A!"#$#%&A&*?!"#$#%&A&*(!"#$#%&A&*) !"#$#%&A&*>!"#$#%&A&*@!"#$#%&A&*%!"#$#%&A&*'!"#$#%&A&*&!"#$#%&A&**!"#$#%&A&*A!"#$#%&A&A?!"#$#%&A&A(!"#$#%&A&A)!"#$#%&A&A>!"#$#%&A&A%!"#$#%&A&A'!"#$#%&A&A*!"#$#%&A&AA!"#$#%&A*??!"#$#%&A*?(!"#$#%&A*?)!"#$#%&A*?>!"#$#%&A*?@!"#$#%&A*?%!"#$#%&A*?'!"#$#%&A*?&!"#$#%&A*?* !"#$#%&A*?A!"#$#%&A*(?!"#$#%&A*((!"#$#%&A*()!"#$#%&A*(>!"#$#%&A*(@!"#$#%&A*(%!"#$#%&A*('!"#$#%&A*(&!"#$#%&A*(*!"#$#%&A*(A!"#$#%&A*)?!"#$#%&A*)(!"#$#%&A*))!"#$#%&A*)>!"#$#%&A*)@!"#$#%&A*)%!"#$#%&A*)'!"#$#%&A*)&!"#$#%&A*)*!"#$#%&A*)A!"#$#%&A*>?!"#$#%&A*>(!"#$#%&A*>) !"#$#%&A*>>!"#$#%&A*>@!"#$#%&A*>'!"#$#%&A*>&!"#$#%&A*>*!"#$#%&A*>A!"#$#%&A*@?!"#$#%&A*@(!"#$#%&A*@)!"#$#%&A*@@!"#$#%&A*@%!"#$#%&A*@'!"#$#%&A*@&!"#$#%&A*@*!"#$#%&A*@A!"#$#%&A*%?!"#$#%&A*%(!"#$#%&A*%)!"#$#%&A*%>!"#$#%&A*%@!"#$#%&A*%%!"#$#%&A*%'!"#$#%&A*%&!"#$#%&A*%* !"#$#%&A*%A!"#$#%&A*'?!"#$#%&A*'(!"#$#%&A*')!"#$#%&A*'>!"#$#%&A*'@!"#$#%&A*'%!"#$#%&A*''!"#$#%&A*'&!"#$#%&A*'*!"#$#%&A*'A!"#$#%&A*&?!"#$#%&A*&(!"#$#%&A*&)!"#$#%&A*&>!"#$#%&A*&@!"#$#%&A*&%!"#$#%&A*&'!"#$#%&A*&&!"#$#%&A*&*!"#$#%&A*&A!"#$#%&A**?!"#$#%&A**(!"#$#%&A**) !"#$#%&A**>!"#$#%&A**@!"#$#%&A**%!"#$#%&A**'!"#$#%&A**&!"#$#%&A***!"#$#%&A**A!"#$#%&A*A?!"#$#%&A*A(!"#$#%&A*A)!"#$#%&A*A>!"#$#%&A*A@!"#$#%&A*A%!"#$#%&A*A'!"#$#%&A*A&!"#$#%&A*A*!"#$#%&A*AA!"#$#%&AA??!"#$#%&AA?(!"#$#%&AA?)!"#$#%&AA?>!"#$#%&AA?@!"#$#%&AA?%!"#$#%&AA?' !"#$#%&AA?&!"#$#%&AA?*!"#$#%&AA?A!"#$#%&AA(?!"#$#%&AA((!"#$#%&AA()!"#$#%&AA(>!"#$#%&AA(@!"#$#%&AA(%!"#$#%&AA('!"#$#%&AA(&!"#$#%&AA(* $ $ 7B5(A,47 2B 8 47 7 ; H + !24 D D4 7 $D 7B5(A 9

!"#$#%&AA(A!"#$#%&AA)(!"#$#%&AA))!"#$#%&AA)>!"#$#%&AA)@!"#$#%&AA)%!"#$#%&AA)'!"#$#%&AA)&!"#$#%&AA)*!"#$#%&AA)A!"#$#%&AA>?!"#$#%&AA>(!"#$#%&AA>)!"#$#%&AA>>!"#$#%&AA>@!"#$#%&AA>%!"#$#%&AA>'!"#$#%&AA>&!"#$#%&AA>*!"#$#%&AA>A!"#$#%&AA@?!"#$#%&AA@(!"#$#%&AA@)!"#$#%&AA@> !"#$#%&AA@@!"#$#%&AA@%!"#$#%&AA@'!"#$#%&AA@&!"#$#%&AA@*!"#$#%&AA@A!"#$#%&AA%?!"#$#%&AA%)!"#$#%&AA%>!"#$#%&AA%@!"#$#%&AA%%!"#$#%&AA%'!"#$#%&AA%&!"#$#%&AA%*!"#$#%&AA%A!"#$#%&AA'?!"#$#%&AA'(!"#$#%&AA')!"#$#%&AA'>!"#$#%&AA'@!"#$#%&AA'%!"#$#%&AA''!"#$#%&AA'&!"#$#%&AA'*

# !"#$#%&AA'A!"#$#%&AA&?!"#$#%&AA&)!"#$#%&AA&>!"#$#%&AA&@!"#$#%&AA&%!"#$#%&AA&'!"#$#%&AA&&!"#$#%&AA&*!"#$#%&AA&A!"#$#%&AA*?!"#$#%&AA*)!"#$#%&AA*>!"#$#%&AA*@!"#$#%&AA*%!"#$#%&AA*'!"#$#%&AA*&!"#$#%&AA**!"#$#%&AA*A!"#$#%&AAA?!"#$#%&AAA(!"#$#%&AAA)!"#$#%&AAA>!"#$#%&AAA@ !"#$#%&AAA%!"#$#%&AAA'!"#$#%&AAA&!"#$#%&AAA*!"#$#%&AAAA!"#$#%*????!"#$#%*???(!"#$#%*???)!"#$#%*???>!"#$#%*???@!"#$#%*???%!"#$#%*???'!"#$#%*???&!"#$#%*???*!"#$#%*???A!"#$#%*??(?!"#$#%*??((!"#$#%*??()!"#$#%*??(>!"#$#%*??(@!"#$#%*??(%!"#$#%*??('!"#$#%*??(&!"#$#%*??(*

!"#$#%*??(A!"#$#%*??)?!"#$#%*??)(!"#$#%*??))!"#$#%*??)>!"#$#%*??)@!"#$#%*??)%!"#$#%*??)'!"#$#%*??)&!"#$#%*??)*!"#$#%*??)A!"#$#%*??>?!"#$#%*??>(!"#$#%*??>)!"#$#%*??>>!"#$#%*??>@!"#$#%*??>%!"#$#%*??>'!"#$#%*??>&!"#$#%*??>*!"#$#%*??>A!"#$#%*??@?!"#$#%*??@(!"#$#%*??@) !"#$#%*??@>!"#$#%*??@@!"#$#%*??@%!"#$#%*??@'!"#$#%*??@&!"#$#%*??@*!"#$#%*??@A!"#$#%*??%?!"#$#%*??%(!"#$#%*??%)!"#$#%*??%>!"#$#%*??%@!"#$#%*??%%!"#$#%*??%'!"#$#%*??%&!"#$#%*??%*!"#$#%*??%A!"#$#%*??'?!"#$#%*??'(!"#$#%*??'>!"#$#%*??'@!"#$#%*??'%!"#$#%*??''!"#$#%*??'& !"#$#%*??'*!"#$#%*??'A!"#$#%*??&?!"#$#%*??&(!"#$#%*??&)!"#$#%*??&@!"#$#%*??&%!"#$#%*??&'!"#$#%*??&&!"#$#%*??&*!"#$#%*??&A!"#$#%*??*?!"#$#%*??*(!"#$#%*??*)!"#$#%*??*>!"#$#%*??*@!"#$#%*??*%!"#$#%*??*'!"#$#%*??*&!"#$#%*??**!"#$#%*??*A!"#$#%*??A?!"#$#%*??A(!"#$#%*??A)

# !"#$#%*??A>!"#$#%*??A@!"#$#%*??A%!"#$#%*??A'!"#$#%*??A&!"#$#%*??A*!"#$#%*??AA!"#$#%*?(??!"#$#%*?(?(!"#$#%*?(?)!"#$#%*?(?>!"#$#%*?(?@!"#$#%*?(?%!"#$#%*?(?'!"#$#%*?(?&!"#$#%*?(?*!"#$#%*?(?A!"#$#%*?((?!"#$#%*?(((!"#$#%*?(()!"#$#%*?((>!"#$#%*?((@!"#$#%*?((%!"#$#%*?((' !"#$#%*?((&!"#$#%*?((*!"#$#%*?((A!"#$#%*?()?!"#$#%*?()(!"#$#%*?())!"#$#%*?()>!"#$#%*?()@!"#$#%*?()%!"#$#%*?()'!"#$#%*?()&!"#$#%*?()*!"#$#%*?()A!"#$#%*?(>?!"#$#%*?(>(!"#$#%*?(>)!"#$#%*?(>>!"#$#%*?(>@!"#$#%*?(>%!"#$#%*?(>'!"#$#%*?(>&!"#$#%*?(>A!"#$#%*?(@?!"#$#%*?(@( !"#$#%*?(@)!"#$#%*?(@>!"#$#%*?(@@!"#$#%*?(@%!"#$#%*?(@'!"#$#%*?(@&!"#$#%*?(@*!"#$#%*?(@A!"#$#%*?(%?!"#$#%*?(%(!"#$#%*?(%)!"#$#%*?(%>!"#$#%*?(%@!"#$#%*?(%%!"#$#%*?(%'!"#$#%*?(%&!"#$#%*?(%*!"#$#%*?(%A!"#$#%*?('?!"#$#%*?('(!"#$#%*?(')!"#$#%*?('>!"#$#%*?('@!"#$#%*?('' !"#$#%*?('&!"#$#%*?('*!"#$#%*?('A!"#$#%*?(&?!"#$#%*?(&(!"#$#%*?(&)!"#$#%*?(&>!"#$#%*?(&@!"#$#%*?(&%!"#$#%*?(&'!"#$#%*?(&&!"#$#%*?(&*!"#$#%*?(&A!"#$#%*?(*?!"#$#%*?(*(!"#$#%*?(*)!"#$#%*?(*>!"#$#%*?(*@!"#$#%*?(*%!"#$#%*?(*'!"#$#%*?(**!"#$#%*?(*A!"#$#%*?(A?!"#$#%*?(A( !"#$#%*?(A)!"#$#%*?(A>!"#$#%*?(A@!"#$#%*?(A%!"#$#%*?(A'!"#$#%*?(A&!"#$#%*?(A*!"#$#%*?(AA!"#$#%*?)??!"#$#%*?)?(!"#$#%*?)?)!"#$#%*?)?>!"#$#%*?)?@!"#$#%*?)?%!"#$#%*?)?'!"#$#%*?)?&!"#$#%*?)?*!"#$#%*?)?A!"#$#%*?)((!"#$#%*?)()!"#$#%*?)(>!"#$#%*?)(@!"#$#%*?)(%!"#$#%*?)(' !"#$#%*?)(&!"#$#%*?)(*!"#$#%*?)(A!"#$#%*?))?!"#$#%*?))(!"#$#%*?)))!"#$#%*?))>!"#$#%*?))@!"#$#%*?))%!"#$#%*?))'!"#$#%*?))&!"#$#%*?))*!"#$#%*?))A!"#$#%*?)>?!"#$#%*?)>(!"#$#%*?)>)!"#$#%*?)>>!"#$#%*?)>%!"#$#%*?)>'!"#$#%*?)>&!"#$#%*?)>*!"#$#%*?)>A!"#$#%*?)@?!"#$#%*?)@( !"#$#%*?)@)!"#$#%*?)@>!"#$#%*?)@@!"#$#%*?)@%!"#$#%*?)@'!"#$#%*?)@&!"#$#%*?)@*!"#$#%*?)@A!"#$#%*?)%?!"#$#%*?)%(!"#$#%*?)%)!"#$#%*?)%>!"#$#%*?)%@!"#$#%*?)%%!"#$#%*?)%&!"#$#%*?)%*!"#$#%*?)%A!"#$#%*?)'?!"#$#%*?)'(!"#$#%*?)')!"#$#%*?)'>!"#$#%*?)'@!"#$#%*?)'%!"#$#%*?)'' !"#$#%*?)'&!"#$#%*?)'*!"#$#%*?)'A!"#$#%*?)&?!"#$#%*?)&(!"#$#%*?)&)!"#$#%*?)&>!"#$#%*?)&@!"#$#%*?)&%!"#$#%*?)&'!"#$#%*?)&&!"#$#%*?)&*!"#$#%*?)&A!"#$#%*?)*?!"#$#%*?)*( $ $ " 7B5(A,47 D: 12 9$ $ " H + !24 D D4 7 $D 7B5(A 9

| !"#$#%*?)*)!"#$#%*?)*> $ $7 7B5(A,47 | | | +29$ $7 H + !24 D D  4 7 $D 7B5(A 9 |
| --- | --- | --- | --- |
| !"#$#%*?)*@ $ $ " 7B5(A,47 | | | D: 12 9$ $ " H + !24 D  D4 7 $D 7B5(A 9 |
| !"#$#%*?)*'!"#$#%*?)*& $ $7 7B5(A,47  !"#$#%*?)**!"#$#%*?)*A!"#$#%*?)A?!"#$#%*?)A(!"#$#%*?)A)!"#$#%*?)A>!"#$#%*?)A@!"#$#%*?)A%!"#$#%*?)A'!"#$#%*?)A&!"#$#%*?)A* | | | +29$ $7 H + !24 D D  4 7 $D 7B5(A 9 |
|  | $ $ " | 7B5(A,47 | D: 12 9$ $ " H + !24 D  D4 7 $D 7B5(A 9 |
| !"#$#%*?)AA!"#$#%*?>?? !"#$#%*?>?( | $ $7 | 7B5(A,47 | +29$ $7 H + !24 D D  4 7 $D 7B5(A 9 |
| !"#$#%*?>?)!"#$#%*?>?> !"#$#%*?>?@ | $ $ " | 7B5(A,47 | D: 12 9$ $ " H + !24 D  D4 7 $D 7B5(A 9 |
| !"#$#%*?>?% | $ $7 | 7B5(A,47 | +29$ $7 H + !24 D D  4 7 $D 7B5(A 9 |
| !"#$#%*?>?'!"#$#%*?>?& !"#$#%*?>?*!"#$#%*?>?A  !"#$#%*?>(?!"#$#%*?>(( !"#$#%*?>() | $ $ " | 7B5(A,47 | D: 12 9$ $ " H + !24 D  D4 7 $D 7B5(A 9 |
| !"#$#%*?>(> | $ $7 | 7B5(A,47 | +29$ $7 H + !24 D D  4 7 $D 7B5(A 9 |
| !"#$#%*?>(@!"#$#%*?>(% !"#$#%*?>('!"#$#%*?>(&  !"#$#%*?>(*!"#$#%*?>(A  !"#$#%*?>)?!"#$#%*?>)( | $ $ " | 7B5(A,47 | D: 12 9$ $ " H + !24 D  D4 7 $D 7B5(A 9 |
| !"#$#%*?>)) | $ $7 | 7B5(A,47 | +29$ $7 H + !24 D D  4 7 $D 7B5(A 9 |
| !"#$#%*?>)>!"#$#%*?>)@ | $ $ " | 7B5(A,47 | D: 12 9$ $ " H + !24 D  D4 7 $D 7B5(A 9 |
| !"#$#%*?>)% | $ $7 | 7B5(A,47 | +29$ $7 H + !24 D D  4 7 $D 7B5(A 9 |
| !"#$#%*?>)' | $ $ " | 7B5(A,47 | D: 12 9$ $ " H + !24 D  D4 7 $D 7B5(A 9 |
| !"#$#%*?>)& | $ $7 | 7B5(A,47 | +29$ $7 H + !24 D D  4 7 $D 7B5(A 9 |
| !"#$#%*?>)*!"#$#%*?>)A !"#$#%*?>>?!"#$#%*?>>( | $ $ " | 7B5(A,47 | D: 12 9$ $ " H + !24 D  D4 7 $D 7B5(A 9 |
| !"#$#%*?>>> | $ $7 | 7B5(A,47 | +29$ $7 H + !24 D D  4 7 $D 7B5(A 9 |
| !"#$#%*?>>@!"#$#%*?>>% !"#$#%*?>>'!"#$#%*?>>& | $ $ " | 7B5(A,47 | D: 12 9$ $ " H + !24 D  D4 7 $D 7B5(A 9 |
| !"#$#%*?>>* | $ $7 | 7B5(A,47 | +29$ $7 H + !24 D D  4 7 $D 7B5(A 9 |
| !"#$#%*?>>A | $ $ " | 7B5(A,47 | D: 12 9$ $ " H + !24 D  D4 7 $D 7B5(A 9 |
| !"#$#%*?>@?!"#$#%*?>@( | $ $7 | 7B5(A,47 | +29$ $7 H + !24 D D  4 7 $D 7B5(A 9 |
| !"#$#%*?>@)!"#$#%*?>@>!"#$#%*?>@@!"#$#%*?>@%!"#$#%*?>@'!"#$#%*?>@&!"#$#%*?>@*!"#$#%*?>@A!"#$#%*?>%?!"#$#%*?>%(!"#$#%*?>%) | | |
| $ $ " 7B5(A,47 | | | D: 12 9$ $ " H + !24 D  D4 7 $D 7B5(A 9 |
| !"#$#%*?>%>!"#$#%*?>%@ $ $7 7B5(A,47 | | | +29$ $7 H + !24 D D  4 7 $D 7B5(A 9 |
| !"#$#%*?>%%!"#$#%*?>%' $ $ " 7B5(A,47  !"#$#%*?>%&!"#$#%*?>%*  !"#$#%*?>%A!"#$#%*?>'? !"#$#%*?>'( | | | D: 12 9$ $ " H + !24 D  D4 7 $D 7B5(A 9 |
| !"#$#%*?>') $ $7 7B5(A,47 | | | +29$ $7 H + !24 D D  4 7 $D 7B5(A 9 |
| !"#$#%*?>'> $ $ " 7B5(A,47 | | | D: 12 9$ $ " H + !24 D  D4 7 $D 7B5(A 9 |
| !"#$#%*?>'@ $ $7 7B5(A,47 | | | +29$ $7 H + !24 D D  4 7 $D 7B5(A 9 |
| !"#$#%*?>'%!"#$#%*?>'' $ $ " 7B5(A,47 | | | D: 12 9$ $ " H + !24 D  D4 7 $D 7B5(A 9 |
| !"#$#%*?>'&!"#$#%*?>'* $ $7 7B5(A,47  !"#$#%*?>'A | | | +29$ $7 H + !24 D D  4 7 $D 7B5(A 9 |
| !"#$#%*?>&? $ $ " 7B5(A,47 | | | D: 12 9$ $ " H + !24 D  D4 7 $D 7B5(A 9 |
| !"#$#%*?>&( $ $7 7B5(A,47 | | | +29$ $7 H + !24 D D  4 7 $D 7B5(A 9 |
| !"#$#%*?>&) $ $ " 7B5(A,47 | | | D: 12 9$ $ " H + !24 D  D4 7 $D 7B5(A 9 |
| !"#$#%*?>&> $ $7 7B5(A,47  !"#$#%*?>&@!"#$#%*?>&%!"#$#%*?>&'!"#$#%*?>&&!"#$#%*?>&*!"#$#%*?>&A!"#$#%*?>*?!"#$#%*?>*(!"#$#%*?>*)!"#$#%*?>*>!"#$#%*?>*@ | | | +29$ $7 H + !24 D D  4 7 $D 7B5(A 9 |
| $ $ " 7B5(A,47 | | | D: 12 9$ $ " H + !24 D  D4 7 $D 7B5(A 9 |
| !"#$#%*?>*% $ $7 7B5(A,47 | | | +29$ $7 H + !24 D D  4 7 $D 7B5(A 9 |
| !"#$#%*?>*'!"#$#%*?>*& $ $ " 7B5(A,47  !"#$#%*?>**!"#$#%*?>*A !"#$#%*?>A? | | | D: 12 9$ $ " H + !24 D  D4 7 $D 7B5(A 9 |
| !"#$#%*?>A( $ $7 7B5(A,47 | | | +29$ $7 H + !24 D D  4 7 $D 7B5(A 9 |
| !"#$#%*?>A) $ $ " 7B5(A,47 | | | D: 12 9$ $ " H + !24 D  D4 7 $D 7B5(A 9 |
| !"#$#%*?>A>!"#$#%*?>A@ $ $7 7B5(A,47 | | | +29$ $7 H + !24 D D  4 7 $D 7B5(A 9 |
| !"#$#%*?>A% $ $ " 7B5(A,47 | | | D: 12 9$ $ " H + !24 D  D4 7 $D 7B5(A 9 |

| !"#$#%*?>A' $ $7 | | | +29$ $7 H + !24 D D  4 7 $D 7B5(A 9 |
| --- | --- | --- | --- |
| !"#$#%*?>A&!"#$#%*?>A* $ $ " 7B5(A,47  !"#$#%*?>AA | | | D: 12 9$ $ " H + !24 D  D4 7 $D 7B5(A 9 |
| !"#$#%*?@?? $ $7 7B5(A,47  !"#$#%*?@?(!"#$#%*?@?)!"#$#%*?@?>!"#$#%*?@?@!"#$#%*?@?%!"#$#%*?@?'!"#$#%*?@?&!"#$#%*?@?*!"#$#%*?@?A!"#$#%*?@(?!"#$#%*?@((!"#$#%*?@()!"#$#%*?@(> | | | +29$ $7 H + !24 D D  4 7 $D 7B5(A 9 |
| $ $ " 7B5(A,47 | | | D: 12 9$ $ " H + !24 D  D4 7 $D 7B5(A 9 |
| !"#$#%*?@(@ $ $7 7B5(A,47 | | | +29$ $7 H + !24 D D  4 7 $D 7B5(A 9 |
| !"#$#%*?@(%!"#$#%*?@(' $ $ " 7B5(A,47  !"#$#%*?@(& | | | D: 12 9$ $ " H + !24 D  D4 7 $D 7B5(A 9 |
| !"#$#%*?@(* $ $7 7B5(A,47 | | | +29$ $7 H + !24 D D  4 7 $D 7B5(A 9 |
| !"#$#%*?@(A!"#$#%*?@)? $ $ " 7B5(A,47 | | | D: 12 9$ $ " H + !24 D  D4 7 $D 7B5(A 9 |
| !"#$#%*?@)( $ $7 7B5(A,47  !"#$#%*?@))!"#$#%*?@)>!"#$#%*?@)@!"#$#%*?@)%!"#$#%*?@)'!"#$#%*?@)&!"#$#%*?@)*!"#$#%*?@)A!"#$#%*?@>?!"#$#%*?@>(!"#$#%*?@>)!"#$#%*?@>>!"#$#%*?@>@ | | | +29$ $7 H + !24 D D  4 7 $D 7B5(A 9 |
|  | $ $ " | 7B5(A,47 | D: 12 9$ $ " H + !24 D  D4 7 $D 7B5(A 9 |
| !"#$#%*?@>% | $ $7 | 7B5(A,47 | +29$ $7 H + !24 D D  4 7 $D 7B5(A 9 |
| !"#$#%*?@>'!"#$#%*?@>& !"#$#%*?@>*!"#$#%*?@>A  !"#$#%*?@@?!"#$#%*?@@(  !"#$#%*?@@)!"#$#%*?@@> | $ $ " | 7B5(A,47 | D: 12 9$ $ " H + !24 D  D4 7 $D 7B5(A 9 |
| !"#$#%*?@@@ | $ $7 | 7B5(A,47 | +29$ $7 H + !24 D D  4 7 $D 7B5(A 9 |
| !"#$#%*?@@%!"#$#%*?@@' !"#$#%*?@@&!"#$#%*?@@* | $ $ " | 7B5(A,47 | D: 12 9$ $ " H + !24 D  D4 7 $D 7B5(A 9 |
| !"#$#%*?@@A | $ $7 | 7B5(A,47 | +29$ $7 H + !24 D D  4 7 $D 7B5(A 9 |
| !"#$#%*?@%?!"#$#%*?@%( !"#$#%*?@%) | $ $ " | 7B5(A,47 | D: 12 9$ $ " H + !24 D  D4 7 $D 7B5(A 9 |
| !"#$#%*?@%> | $ $7 | 7B5(A,47 | +29$ $7 H + !24 D D  4 7 $D 7B5(A 9 |
| !"#$#%*?@%@!"#$#%*?@%%  !"#$#%*?@%'!"#$#%*?@%& !"#$#%*?@%* | $ $ " | 7B5(A,47 | D: 12 9$ $ " H + !24 D  D4 7 $D 7B5(A 9 |
| !"#$#%*?@%A | $ $7 | 7B5(A,47 | +29$ $7 H + !24 D D  4 7 $D 7B5(A 9 |
| !"#$#%*?@'?!"#$#%*?@'(  !"#$#%*?@')!"#$#%*?@'> !"#$#%*?@'@ | $ $ " | 7B5(A,47 | D: 12 9$ $ " H + !24 D  D4 7 $D 7B5(A 9 |
| !"#$#%*?@'% | $ $7 | 7B5(A,47 | +29$ $7 H + !24 D D  4 7 $D 7B5(A 9 |
| !"#$#%*?@'' | $ $ " | 7B5(A,47 | D: 12 9$ $ " H + !24 D  D4 7 $D 7B5(A 9 |
| !"#$#%*?@'& | $ $7 | 7B5(A,47 | +29$ $7 H + !24 D D  4 7 $D 7B5(A 9 |
| !"#$#%*?@'*!"#$#%*?@'A !"#$#%*?@&? | $ $ " | 7B5(A,47 | D: 12 9$ $ " H + !24 D  D4 7 $D 7B5(A 9 |
| !"#$#%*?@&( | $ $7 | 7B5(A,47 | +29$ $7 H + !24 D D  4 7 $D 7B5(A 9 |
| !"#$#%*?@&)!"#$#%*?@&>  !"#$#%*?@&@!"#$#%*?@&%  !"#$#%*?@&'!"#$#%*?@&& | $ $ " | 7B5(A,47 | D: 12 9$ $ " H + !24 D  D4 7 $D 7B5(A 9 |
| !"#$#%*?@&*!"#$#%*?@&A !"#$#%*?@*?!"#$#%*?@*( | $ $7 | 7B5(A,47 | +29$ $7 H + !24 D D  4 7 $D 7B5(A 9 |
| !"#$#%*?@*)!"#$#%*?@*> !"#$#%*?@*@ | $ $ " | 7B5(A,47 | D: 12 9$ $ " H + !24 D  D4 7 $D 7B5(A 9 |
| !"#$#%*?@*%!"#$#%*?@*' | $ $7 | 7B5(A,47 | +29$ $7 H + !24 D D  4 7 $D 7B5(A 9 |
| !"#$#%*?@*&!"#$#%*?@** !"#$#%*?@*A!"#$#%*?@A?  !"#$#%*?@A(!"#$#%*?@A)  !"#$#%*?@A>!"#$#%*?@A@ | $ $ " | 7B5(A,47 | D: 12 9$ $ " H + !24 D  D4 7 $D 7B5(A 9 |
| !"#$#%*?@A% | $ $7 | 7B5(A,47 | +29$ $7 H + !24 D D  4 7 $D 7B5(A 9 |
| !"#$#%*?@A'!"#$#%*?@A& | $ $ " | 7B5(A,47 | D: 12 9$ $ " H + !24 D  D4 7 $D 7B5(A 9 |
| !"#$#%*?@AA | $ $7 | 7B5(A,47 | +29$ $7 H + !24 D D  4 7 $D 7B5(A 9 |
| !"#$#%*?%??!"#$#%*?%?( | $ $ " | 7B5(A,47 | D: 12 9$ $ " H + !24 D  D4 7 $D 7B5(A 9 |
| !"#$#%*?%?) | $ $7 | 7B5(A,47 | +29$ $7 H + !24 D D  4 7 $D 7B5(A 9 |
| !"#$#%*?%?>!"#$#%*?%?@  !"#$#%*?%?%!"#$#%*?%?' !"#$#%*?%?& | $ $ " | 7B5(A,47 | D: 12 9$ $ " H + !24 D  D4 7 $D 7B5(A 9 |
| !"#$#%*?%?*!"#$#%*?%?A | $ $7 | 7B5(A,47 | +29$ $7 H + !24 D D  4 7 $D 7B5(A 9 |
| !"#$#%*?%(? | $ $ " | 7B5(A,47 | D: 12 9$ $ " H + !24 D  D4 7 $D 7B5(A 9 |
| !"#$#%*?%(( | $ $7 | 7B5(A,47 | +29$ $7 H + !24 D D  4 7 $D 7B5(A 9 |
| !"#$#%*?%()!"#$#%*?%(> !"#$#%*?%(@ | $ $ " | 7B5(A,47 | D: 12 9$ $ " H + !24 D  D4 7 $D 7B5(A 9 |
| !"#$#%*?%(% | $ $7 | 7B5(A,47 | +29$ $7 H + !24 D D  4 7 $D 7B5(A 9 |

# !"#$#%*?%('!"#$#%*?%(&!"#$#%*?%(*!"#$#%*?%(A!"#$#%*?%)?!"#$#%*?%)(!"#$#%*?%))!"#$#%*?%)>!"#$#%*?%)@!"#$#%*?%)%!"#$#%*?%)'!"#$#%*?%)&!"#$#%*?%)*!"#$#%*?%)A!"#$#%*?%>?!"#$#%*?%>(

|  | $ $ " | 7B5(A,47 | D: 12 9$ $ " H + !24 D  D4 7 $D 7B5(A 9 |
| --- | --- | --- | --- |
| !"#$#%*?%>)!"#$#%*?%>> | $ $7 | 7B5(A,47 | +29$ $7 H + !24 D D  4 7 $D 7B5(A 9 |
| !"#$#%*?%>@!"#$#%*?%>% | $ $ " | 7B5(A,47 | D: 12 9$ $ " H + !24 D  D4 7 $D 7B5(A 9 |
| !"#$#%*?%>' | $ $7 | 7B5(A,47 | +29$ $7 H + !24 D D |

# 4 7 $D 7B5(A 9 !"#$#%*?%>&!"#$#%*?%>*!"#$#%*?%>A!"#$#%*?%@?!"#$#%*?%@(!"#$#%*?%@)!"#$#%*?%@>!"#$#%*?%@@!"#$#%*?%@%!"#$#%*?%@'!"#$#%*?%@&!"#$#%*?%@*!"#$#%*?%@A!"#$#%*?%%(!"#$#%*?%%)!"#$#%*?%%>!"#$#%*?%%@!"#$#%*?%%%!"#$#%*?%%'

|  | $ $ " | 7B5(A,47 | D: 12 9$ $ " H + !24 D  D4 7 $D 7B5(A 9 |
| --- | --- | --- | --- |
| !"#$#%*?%%& | $ $7 | 7B5(A,47 | +29$ $7 H + !24 D D  4 7 $D 7B5(A 9 |
| !"#$#%*?%%* | $ $ " | 7B5(A,47 | D: 12 9$ $ " H + !24 D  D4 7 $D 7B5(A 9 |
| !"#$#%*?%%A | $ $7 | 7B5(A,47 | +29$ $7 H + !24 D D  4 7 $D 7B5(A 9 |
| !"#$#%*?%'?!"#$#%*?%'( !"#$#%*?%') | $ $ " | 7B5(A,47 | D: 12 9$ $ " H + !24 D  D4 7 $D 7B5(A 9 |
| !"#$#%*?%'@!"#$#%*?%'% | $ $7 | 7B5(A,47 | +29$ $7 H + !24 D D  4 7 $D 7B5(A 9 |
| !"#$#%*?%'' | $ $ " | 7B5(A,47 | D: 12 9$ $ " H + !24 D  D4 7 $D 7B5(A 9 |
| !"#$#%*?%'& | $ $7 | 7B5(A,47 | +29$ $7 H + !24 D D  4 7 $D 7B5(A 9 |
| !"#$#%*?%'* | $ $ " | 7B5(A,47 | D: 12 9$ $ " H + !24 D  D4 7 $D 7B5(A 9 |
| !"#$#%*?%'A | $ $7 | 7B5(A,47 | +29$ $7 H + !24 D D  4 7 $D 7B5(A 9 |
| !"#$#%*?%&?!"#$#%*?%&( !"#$#%*?%&) | $ $ " | 7B5(A,47 | D: 12 9$ $ " H + !24 D  D4 7 $D 7B5(A 9 |
| !"#$#%*?%&> | $ $7 | 7B5(A,47 | +29$ $7 H + !24 D D  4 7 $D 7B5(A 9 |
| !"#$#%*?%&@!"#$#%*?%&% | $ $ " | 7B5(A,47 | D: 12 9$ $ " H + !24 D  D4 7 $D 7B5(A 9 |
| !"#$#%*?%&'!"#$#%*?%&& | $ $7 | 7B5(A,47 | +29$ $7 H + !24 D D  4 7 $D 7B5(A 9 |
| !"#$#%*?%&* | $ $ " | 7B5(A,47 | D: 12 9$ $ " H + !24 D  D4 7 $D 7B5(A 9 |
| !"#$#%*?%&A | $ $7 | 7B5(A,47 | +29$ $7 H + !24 D D  4 7 $D 7B5(A 9 |
| !"#$#%*?%*?!"#$#%*?%*( | $ $ " | 7B5(A,47 | D: 12 9$ $ " H + !24 D  D4 7 $D 7B5(A 9 |
| !"#$#%*?%*) | $ $7 | 7B5(A,47 | +29$ $7 H + !24 D D  4 7 $D 7B5(A 9 |
| !"#$#%*?%*>!"#$#%*?%*@ !"#$#%*?%*% | $ $ " | 7B5(A,47 | D: 12 9$ $ " H + !24 D  D4 7 $D 7B5(A 9 |
| !"#$#%*?%*'!"#$#%*?%*& | $ $7 | 7B5(A,47 | +29$ $7 H + !24 D D  4 7 $D 7B5(A 9 |
| !"#$#%*?%**!"#$#%*?%*A  !"#$#%*?%A?!"#$#%*?%A( !"#$#%*?%A) | $ $ " | 7B5(A,47 | D: 12 9$ $ " H + !24 D  D4 7 $D 7B5(A 9 |
| !"#$#%*?%A> | $ $7 | 7B5(A,47 | +29$ $7 H + !24 D D  4 7 $D 7B5(A 9 |
| !"#$#%*?%A%!"#$#%*?%A' !"#$#%*?%A&!"#$#%*?%A*  !"#$#%*?%AA!"#$#%*?'??  !"#$#%*?'?(!"#$#%*?'?) | $ $ " | 7B5(A,47 | D: 12 9$ $ " H + !24 D  D4 7 $D 7B5(A 9 |
| !"#$#%*?'?> | $ $7 | 7B5(A,47 | +29$ $7 H + !24 D D  4 7 $D 7B5(A 9 |
| !"#$#%*?'?@!"#$#%*?'?% !"#$#%*?'?' | $ $ " | 7B5(A,47 | D: 12 9$ $ " H + !24 D  D4 7 $D 7B5(A 9 |
| !"#$#%*?'?& | $ $7 | 7B5(A,47 | +29$ $7 H + !24 D D  4 7 $D 7B5(A 9 |
| !"#$#%*?'?*!"#$#%*?'?A | $ $ " | 7B5(A,47 | D: 12 9$ $ " H + !24 D  D4 7 $D 7B5(A 9 |
| !"#$#%*?'(? | $ $7 | 7B5(A,47 | +29$ $7 H + !24 D D  4 7 $D 7B5(A 9 |
| !"#$#%*?'((!"#$#%*?'() !"#$#%*?'(>!"#$#%*?'(@  !"#$#%*?'(%!"#$#%*?'(' !"#$#%*?'(& | $ $ " | 7B5(A,47 | D: 12 9$ $ " H + !24 D  D4 7 $D 7B5(A 9 |
| !"#$#%*?'(* | $ $7 | 7B5(A,47 | +29$ $7 H + !24 D D  4 7 $D 7B5(A 9 |
| !"#$#%*?'(A!"#$#%*?')? | $ $ " | 7B5(A,47 | D: 12 9$ $ " H + !24 D  D4 7 $D 7B5(A 9 |
| !"#$#%*?')( | $ $7 | 7B5(A,47 | +29$ $7 H + !24 D D  4 7 $D 7B5(A 9 |
| !"#$#%*?'))!"#$#%*?')> !"#$#%*?')@ | $ $ " | 7B5(A,47 | D: 12 9$ $ " H + !24 D  D4 7 $D 7B5(A 9 |
| !"#$#%*?')%!"#$#%*?')' | $ $7 | 7B5(A,47 | +29$ $7 H + !24 D D  4 7 $D 7B5(A 9 |
| !"#$#%*?')&!"#$#%*?')* !"#$#%*?')A!"#$#%*?'>? | $ $ " | 7B5(A,47 | D: 12 9$ $ " H + !24 D  D4 7 $D 7B5(A 9 |
| !"#$#%*?'>(!"#$#%*?'>) | $ $1 4 | 7B5(A,47 | 9$ $1 4 H + !24 D D4  7 $D 7B5(A 9 |
| !"#$#%*?'>> | $ $ " | 7B5(A,47 | D: 12 9$ $ " H + !24 D  D4 7 $D 7B5(A 9 |
| !"#$#%*?'>@ | $ $1 4 | 7B5(A,47 | 9$ $1 4 H + !24 D D4  7 $D 7B5(A 9 |

| !"#$#%*?'>%!"#$#%*?'>' | $ $ " |  | D: 12 9$ $ " H + !24 D  D4 7 $D 7B5(A 9 |
| --- | --- | --- | --- |
| !"#$#%*?'>&!"#$#%*?'>* !"#$#%*?'>A | $ $1 4 | 7B5(A,47 | 9$ $1 4 H + !24 D D4  7 $D 7B5(A 9 |
| !"#$#%*?'@? | $ $ " | 7B5(A,47 | D: 12 9$ $ " H + !24 D  D4 7 $D 7B5(A 9 |
| !"#$#%*?'@(!"#$#%*?'@) !"#$#%*?'@>!"#$#%*?'@@ | $ $1 4 | 7B5(A,47 | 9$ $1 4 H + !24 D D4  7 $D 7B5(A 9 |
| !"#$#%*?'@% | $ $ " | 7B5(A,47 | D: 12 9$ $ " H + !24 D  D4 7 $D 7B5(A 9 |
| !"#$#%*?'@'!"#$#%*?'@& | $ $1 4 | 7B5(A,47 | 9$ $1 4 H + !24 D D4  7 $D 7B5(A 9 |
| !"#$#%*?'@* | $ $ " | 7B5(A,47 | D: 12 9$ $ " H + !24 D  D4 7 $D 7B5(A 9 |
| !"#$#%*?'@A | $ $1 4 | 7B5(A,47 | 9$ $1 4 H + !24 D D4  7 $D 7B5(A 9 |
| !"#$#%*?'%?!"#$#%*?'%( | $ $ " | 7B5(A,47 | D: 12 9$ $ " H + !24 D  D4 7 $D 7B5(A 9 |
| !"#$#%*?'%)!"#$#%*?'%> | $ $1 4 | 7B5(A,47 | 9$ $1 4 H + !24 D D4  7 $D 7B5(A 9 |
| !"#$#%*?'%% | $ $ " | 7B5(A,47 | D: 12 9$ $ " H + !24 D  D4 7 $D 7B5(A 9 |
| !"#$#%*?'%'!"#$#%*?'%& !"#$#%*?'%*!"#$#%*?'%A | $ $1 4 | 7B5(A,47 | 9$ $1 4 H + !24 D D4  7 $D 7B5(A 9 |
| !"#$#%*?''? | $ $ " | 7B5(A,47 | D: 12 9$ $ " H + !24 D  D4 7 $D 7B5(A 9 |
| !"#$#%*?''(!"#$#%*?'') | $ $1 4 | 7B5(A,47 | 9$ $1 4 H + !24 D D4  7 $D 7B5(A 9 |
| !"#$#%*?''> | $ $ " | 7B5(A,47 | D: 12 9$ $ " H + !24 D  D4 7 $D 7B5(A 9 |
| !"#$#%*?''@!"#$#%*?''%  !"#$#%*?'''!"#$#%*?''& !"#$#%*?''*!"#$#%*?''A  !"#$#%*?'&?!"#$#%*?'&(  !"#$#%*?'&)!"#$#%*?'&> | $ $1 4 | 7B5(A,47 | 9$ $1 4 H + !24 D D4  7 $D 7B5(A 9 |
| !"#$#%*?'&@!"#$#%*?'&% !"#$#%*?'&&!"#$#%*?'&*  !"#$#%*?'&A!"#$#%*?'*?  !"#$#%*?'*(!"#$#%*?'*) | $ $ " | 7B5(A,47 | D: 12 9$ $ " H + !24 D  D4 7 $D 7B5(A 9 |
| !"#$#%*?'*>!"#$#%*?'*@  !"#$#%*?'*%!"#$#%*?'*& !"#$#%*?'** | $ $1 4 | 7B5(A,47 | 9$ $1 4 H + !24 D D4  7 $D 7B5(A 9 |
| !"#$#%*?'*A!"#$#%*?'A? !"#$#%*?'A(!"#$#%*?'A) | $ $ " | 7B5(A,47 | D: 12 9$ $ " H + !24 D  D4 7 $D 7B5(A 9 |
| !"#$#%*?'A@ | $ $1 4 | 7B5(A,47 | 9$ $1 4 H + !24 D D4  7 $D 7B5(A 9 |
| !"#$#%*?'A%!"#$#%*?'A' !"#$#%*?'A& | $ $ " | 7B5(A,47 | D: 12 9$ $ " H + !24 D  D4 7 $D 7B5(A 9 |
| !"#$#%*?'A* | $ $1 4 | 7B5(A,47 | 9$ $1 4 H + !24 D D4  7 $D 7B5(A 9 |
| !"#$#%*?'AA | $ $ " | 7B5(A,47 | D: 12 9$ $ " H + !24 D  D4 7 $D 7B5(A 9 |
| !"#$#%*?&??!"#$#%*?&?( !"#$#%*?&?)!"#$#%*?&?@ | $ $1 4 | 7B5(A,47 | 9$ $1 4 H + !24 D D4  7 $D 7B5(A 9 |
| !"#$#%*?&?' | $ $ " | 7B5(A,47 | D: 12 9$ $ " H + !24 D  D4 7 $D 7B5(A 9 |
| !"#$#%*?&?&!"#$#%*?&?A | $ $1 4 | 7B5(A,47 | 9$ $1 4 H + !24 D D4  7 $D 7B5(A 9 |
| !"#$#%*?&(? | $ $ " | 7B5(A,47 | D: 12 9$ $ " H + !24 D  D4 7 $D 7B5(A 9 |
| !"#$#%*?&((!"#$#%*?&() !"#$#%*?&(>!"#$#%*?&(@ !"#$#%*?&(%!"#$#%*?&('  !"#$#%*?&(&!"#$#%*?&(* !"#$#%*?&(A | $ $1 4 | 7B5(A,47 | 9$ $1 4 H + !24 D D4  7 $D 7B5(A 9 |
| !"#$#%*?&)? | $ $ " | 7B5(A,47 | D: 12 9$ $ " H + !24 D  D4 7 $D 7B5(A 9 |
| !"#$#%*?&)( | $ $1 4 | 7B5(A,47 | 9$ $1 4 H + !24 D D4  7 $D 7B5(A 9 |
| !"#$#%*?&))!"#$#%*?&)> | $ $ " | 7B5(A,47 | D: 12 9$ $ " H + !24 D  D4 7 $D 7B5(A 9 |
| !"#$#%*?&)@!"#$#%*?&)%  !"#$#%*?&)'!"#$#%*?&)&  !"#$#%*?&)*!"#$#%*?&)A | $ $1 4 | 7B5(A,47 | 9$ $1 4 H + !24 D D4  7 $D 7B5(A 9 |
| !"#$#%*?&>? | $ $ " | 7B5(A,47 | D: 12 9$ $ " H + !24 D  D4 7 $D 7B5(A 9 |
| !"#$#%*?&>(!"#$#%*?&>) !"#$#%*?&>> | $ $1 4 | 7B5(A,47 | 9$ $1 4 H + !24 D D4  7 $D 7B5(A 9 |
| !"#$#%*?&>@ | $ $ " | 7B5(A,47 | D: 12 9$ $ " H + !24 D  D4 7 $D 7B5(A 9 |
| !"#$#%*?&>%!"#$#%*?&>' | $ $1 4 | 7B5(A,47 | 9$ $1 4 H + !24 D D4  7 $D 7B5(A 9 |
| !"#$#%*?&>&!"#$#%*?&>* | $ $ " | 7B5(A,47 | D: 12 9$ $ " H + !24 D  D4 7 $D 7B5(A 9 |
| !"#$#%*?&>A | $ $1 4 | 7B5(A,47 | 9$ $1 4 H + !24 D D4  7 $D 7B5(A 9 |
| !"#$#%*?&@? | $ $ " | 7B5(A,47 | D: 12 9$ $ " H + !24 D  D4 7 $D 7B5(A 9 |
| !"#$#%*?&@(!"#$#%*?&@)!"#$#%*?&@>!"#$#%*?&@@!"#$#%*?&@%!"#$#%*?&@'!"#$#%*?&@&!"#$#%*?&@*!"#$#%*?&@A!"#$#%*?&%?!"#$#%*?&%)!"#$#%*?&%> | | |
| $ $1 4 7B5(A,47 | | | 9$ $1 4 H + !24 D D4  7 $D 7B5(A 9 |
| !"#$#%*?&%@ $ $ " 7B5(A,47 | | | D: 12 9$ $ " H + !24 D |
|  |  |  | D4 7 $D 7B5(A 9 |
| !"#$#%*?&%%!"#$#%*?&%&  !"#$#%*?&%A!"#$#%*?&'?  !"#$#%*?&'(!"#$#%*?&') | $ $1 4 | 7B5(A,47 | 9$ $1 4 H + !24 D D4  7 $D 7B5(A 9 |
| !"#$#%*?&'>!"#$#%*?&'@ | $ $ " | 7B5(A,47 | D: 12 9$ $ " H + !24 D  D4 7 $D 7B5(A 9 |
| !"#$#%*?&''!"#$#%*?&'& !"#$#%*?&'* | $ $1 4 | 7B5(A,47 | 9$ $1 4 H + !24 D D4  7 $D 7B5(A 9 |
| !"#$#%*?&'A | $ $ " | 7B5(A,47 | D: 12 9$ $ " H + !24 D  D4 7 $D 7B5(A 9 |
| !"#$#%*?&&? | $ $1 4 | 7B5(A,47 | 9$ $1 4 H + !24 D D4  7 $D 7B5(A 9 |
| !"#$#%*?&&( | $ $ " | 7B5(A,47 | D: 12 9$ $ " H + !24 D  D4 7 $D 7B5(A 9 |
| !"#$#%*?&&)!"#$#%*?&&>!"#$#%*?&&@!"#$#%*?&&%!"#$#%*?&&'!"#$#%*?&&&!"#$#%*?&&*!"#$#%*?&&A!"#$#%*?&*(!"#$#%*?&*)!"#$#%*?&*> | | |
| $ $1 4 7B5(A,47 | | | 9$ $1 4 H + !24 D D4  7 $D 7B5(A 9 |
| !"#$#%*?&*@ $ $ " 7B5(A,47 | | | D: 12 9$ $ " H + !24 D  D4 7 $D 7B5(A 9 |
| !"#$#%*?&*%!"#$#%*?&*' $ $1 4 7B5(A,47 | | | 9$ $1 4 H + !24 D D4  7 $D 7B5(A 9 |
| !"#$#%*?&*&!"#$#%*?&** $ $ " 7B5(A,47  !"#$#%*?&*A | | | D: 12 9$ $ " H + !24 D  D4 7 $D 7B5(A 9 |
| !"#$#%*?&A? $ $1 4 7B5(A,47 | | | 9$ $1 4 H + !24 D D4  7 $D 7B5(A 9 |
| !"#$#%*?&A( $ $ " 7B5(A,47 | | | D: 12 9$ $ " H + !24 D |

# D4 7 $D 7B5(A 9

|  | $ $1 4 | 7B5(A,47 | 9$ $1 4 H + !24 D D4  7 $D 7B5(A 9 |
| --- | --- | --- | --- |
| !"#$#%*?*)(!"#$#%*?*)) !"#$#%*?*)>!"#$#%*?*)% | $ $ " | 7B5(A,47 | D: 12 9$ $ " H + !24 D  D4 7 $D 7B5(A 9 |
| !"#$#%*?*)'!"#$#%*?*)& | $ $1 4 | 7B5(A,47 | 9$ $1 4 H + !24 D D4  7 $D 7B5(A 9 |
| !"#$#%*?*)* | $ $ " | 7B5(A,47 | D: 12 9$ $ " H + !24 D  D4 7 $D 7B5(A 9 |
| !"#$#%*?*)A!"#$#%*?*>? !"#$#%*?*>(!"#$#%*?*>) | $ $1 4 | 7B5(A,47 | 9$ $1 4 H + !24 D D4  7 $D 7B5(A 9 |
| !"#$#%*?*>> | $ $ " | 7B5(A,47 | D: 12 9$ $ " H + !24 D  D4 7 $D 7B5(A 9 |
| !"#$#%*?*>@ | $ $1 4 | 7B5(A,47 | 9$ $1 4 H + !24 D D4  7 $D 7B5(A 9 |
| !"#$#%*?*>% | $ $ " | 7B5(A,47 | D: 12 9$ $ " H + !24 D  D4 7 $D 7B5(A 9 |
| !"#$#%*?*>'!"#$#%*?*>&!"#$#%*?*>*!"#$#%*?*>A!"#$#%*?*@(!"#$#%*?*@)!"#$#%*?*@>!"#$#%*?*@@!"#$#%*?*@%!"#$#%*?*@'!"#$#%*?*@&!"#$#%*?*@* | | |
|  | $ $1 4 | 7B5(A,47 | 9$ $1 4 H + !24 D D4  7 $D 7B5(A 9 |
| !"#$#%*?*@A | $ $ " | 7B5(A,47 | D: 12 9$ $ " H + !24 D  D4 7 $D 7B5(A 9 |
| !"#$#%*?*%? | $ $1 4 | 7B5(A,47 | 9$ $1 4 H + !24 D D4  7 $D 7B5(A 9 |
| !"#$#%*?*%( | $ $ " | 7B5(A,47 | D: 12 9$ $ " H + !24 D  D4 7 $D 7B5(A 9 |
| !"#$#%*?*%) | $ $1 4 | 7B5(A,47 | 9$ $1 4 H + !24 D D4  7 $D 7B5(A 9 |
| !"#$#%*?*%> | $ $ " | 7B5(A,47 | D: 12 9$ $ " H + !24 D  D4 7 $D 7B5(A 9 |
| !"#$#%*?*%@!"#$#%*?*%' !"#$#%*?*%& | $ $1 4 | 7B5(A,47 | 9$ $1 4 H + !24 D D4  7 $D 7B5(A 9 |
| !"#$#%*?*%*!"#$#%*?*%A !"#$#%*?*'? | $ $ | 7B5(A,47 | 2B 8 47 7 ; H + !24 D D4 7 $D 7B5(A 9 |
| !"#$#%*?*'( | $ $1 4 | 7B5(A,47 | 9$ $1 4 H + !24 D D4  7 $D 7B5(A 9 |
| !"#$#%*?*')!"#$#%*?*'> | $ $ | 7B5(A,47 | 2B 8 47 7 ; H + !24 D D4 7 $D 7B5(A 9 |
| !"#$#%*?*'@ | $ $1 4 | 7B5(A,47 | 9$ $1 4 H + !24 D D4  7 $D 7B5(A 9 |
| !"#$#%*?*'%!"#$#%*?*''  !"#$#%*?*'&!"#$#%*?*'*  !"#$#%*?*'A!"#$#%*?*&?  !"#$#%*?*&(!"#$#%*?*&) | $ $ | 7B5(A,47 | 2B 8 47 7 ; H + !24 D D4 7 $D 7B5(A 9 |
| !"#$#%*?*&> | $ $1 4 | 7B5(A,47 | 9$ $1 4 H + !24 D D4  7 $D 7B5(A 9 |
| !"#$#%*?*&@ | $ $ | 7B5(A,47 | 2B 8 47 7 ; H + !24 D D4 7 $D 7B5(A 9 |
| !"#$#%*?*&% | $ $1 4 | 7B5(A,47 | 9$ $1 4 H + !24 D D4  7 $D 7B5(A 9 |
| !"#$#%*?*&' | $ $ | 7B5(A,47 | 2B 8 47 7 ; H + !24 D D4 7 $D 7B5(A 9 |
| !"#$#%*?*&& | $ $1 4 | 7B5(A,47 | 9$ $1 4 H + !24 D D4  7 $D 7B5(A 9 |
| !"#$#%*?*&*!"#$#%*?*&A !"#$#%*?**?!"#$#%*?**( | $ $ | 7B5(A,47 | 2B 8 47 7 ; H + !24 D D4 7 $D 7B5(A 9 |
| !"#$#%*?**) | $ $1 4 | 7B5(A,47 | 9$ $1 4 H + !24 D D4  7 $D 7B5(A 9 |
| !"#$#%*?**>!"#$#%*?**@ | $ $ | 7B5(A,47 | 2B 8 47 7 ; H + !24 D D4 7 $D 7B5(A 9 |
| !"#$#%*?**% | $ $1 4 | 7B5(A,47 | 9$ $1 4 H + !24 D D4  7 $D 7B5(A 9 |

!"#$#%*?&A)!"#$#%*?&A>!"#$#%*?&A@!"#$#%*?&A%!"#$#%*?&A'!"#$#%*?&A&!"#$#%*?&A*!"#$#%*?&AA!"#$#%*?*??!"#$#%*?*?(!"#$#%*?*?)!"#$#%*?*?>!"#$#%*?*?@!"#$#%*?*?%!"#$#%*?*?'!"#$#%*?*?&!"#$#%*?*?*!"#$#%*?*?A!"#$#%*?*(?!"#$#%*?*((!"#$#%*?*()!"#$#%*?*(>!"#$#%*?*(@!"#$#%*?*(% !"#$#%*?*('!"#$#%*?*(&!"#$#%*?*(*!"#$#%*?*(A!"#$#%*?*)?

| !"#$#%*?**'!"#$#%*?**&  !"#$#%*?***!"#$#%*?**A  !"#$#%*?*A?!"#$#%*?*A( | $ $ |  | 2B 8 47 7 ; H + !24 D D4 7 $D 7B5(A 9 |
| --- | --- | --- | --- |
| !"#$#%*?*A) | $ $1 4 |  | 9$ $1 4 H + !24 D D4  7 $D 7B5(A 9 |
| !"#$#%*?*A>!"#$#%*?*A@ !"#$#%*?*A% | $ $ | 7B5(A,47 | 2B 8 47 7 ; H + !24 D D4 7 $D 7B5(A 9 |
| !"#$#%*?*A'!"#$#%*?*A& !"#$#%*?*A* | $ $1 4 | 7B5(A,47 | 9$ $1 4 H + !24 D D4  7 $D 7B5(A 9 |
| !"#$#%*?*AA | $ $ | 7B5(A,47 | 2B 8 47 7 ; H + !24 D D4 7 $D 7B5(A 9 |
| !"#$#%*?A?? | $ $1 4 | 7B5(A,47 | 9$ $1 4 H + !24 D D4  7 $D 7B5(A 9 |
| !"#$#%*?A?( | $ $ | 7B5(A,47 | 2B 8 47 7 ; H + !24 D D4 7 $D 7B5(A 9 |
| !"#$#%*?A?)!"#$#%*?A?> !"#$#%*?A?@ | $ $1 4 | 7B5(A,47 | 9$ $1 4 H + !24 D D4  7 $D 7B5(A 9 |
| !"#$#%*?A?%!"#$#%*?A?' !"#$#%*?A?&!"#$#%*?A?* | $ $ | 7B5(A,47 | 2B 8 47 7 ; H + !24 D D4 7 $D 7B5(A 9 |
| !"#$#%*?A(? | $ $1 4 | 7B5(A,47 | 9$ $1 4 H + !24 D D4  7 $D 7B5(A 9 |
| !"#$#%*?A((!"#$#%*?A() | $ $ | 7B5(A,47 | 2B 8 47 7 ; H + !24 D D4 7 $D 7B5(A 9 |
| !"#$#%*?A(> | $ $1 4 | 7B5(A,47 | 9$ $1 4 H + !24 D D4  7 $D 7B5(A 9 |
| !"#$#%*?A(@ | $ $ | 7B5(A,47 | 2B 8 47 7 ; H + !24 D D4 7 $D 7B5(A 9 |
| !"#$#%*?A(% | $ $1 4 | 7B5(A,47 | 9$ $1 4 H + !24 D D4  7 $D 7B5(A 9 |
| !"#$#%*?A(' | $ $ | 7B5(A,47 | 2B 8 47 7 ; H + !24 D D4 7 $D 7B5(A 9 |
| !"#$#%*?A(&!"#$#%*?A(* | $ $1 4 | 7B5(A,47 | 9$ $1 4 H + !24 D D4  7 $D 7B5(A 9 |
| !"#$#%*?A(A!"#$#%*?A)? | $ $ | 7B5(A,47 | 2B 8 47 7 ; H + !24 D D4 7 $D 7B5(A 9 |
| !"#$#%*?A)( | $ $1 4 | 7B5(A,47 | 9$ $1 4 H + !24 D D4  7 $D 7B5(A 9 |
| !"#$#%*?A))!"#$#%*?A)> !"#$#%*?A)@!"#$#%*?A)%  !"#$#%*?A)'!"#$#%*?A)& !"#$#%*?A)* | $ $ | 7B5(A,47 | 2B 8 47 7 ; H + !24 D D4 7 $D 7B5(A 9 |
| !"#$#%*?A)A | $ $1 4 | 7B5(A,47 | 9$ $1 4 H + !24 D D4  7 $D 7B5(A 9 |
| !"#$#%*?A>?!"#$#%*?A>(  !"#$#%*?A>)!"#$#%*?A>> !"#$#%*?A>@ | $ $ | 7B5(A,47 | 2B 8 47 7 ; H + !24 D D4 7 $D 7B5(A 9 |
| !"#$#%*?A>% | $ $1 4 | 7B5(A,47 | 9$ $1 4 H + !24 D D4  7 $D 7B5(A 9 |
| !"#$#%*?A>'!"#$#%*?A>& !"#$#%*?A>*!"#$#%*?A>A | $ $ | 7B5(A,47 | 2B 8 47 7 ; H + !24 D D4 7 $D 7B5(A 9 |
| !"#$#%*?A@? | $ $1 4 | 7B5(A,47 | 9$ $1 4 H + !24 D D4  7 $D 7B5(A 9 |
| !"#$#%*?A@(!"#$#%*?A@) !"#$#%*?A@>!"#$#%*?A@@  !"#$#%*?A@%!"#$#%*?A@' !"#$#%*?A@& | $ $ | 7B5(A,47 | 2B 8 47 7 ; H + !24 D D4 7 $D 7B5(A 9 |
| !"#$#%*?A@* | $ $1 4 | 7B5(A,47 | 9$ $1 4 H + !24 D D4  7 $D 7B5(A 9 |
| !"#$#%*?A@A | $ $ | 7B5(A,47 | 2B 8 47 7 ; H + !24 D D4 7 $D 7B5(A 9 |
| !"#$#%*?A%?!"#$#%*?A%( | $ $1 4 | 7B5(A,47 | 9$ $1 4 H + !24 D D4  7 $D 7B5(A 9 |
| !"#$#%*?A%)!"#$#%*?A%> !"#$#%*?A%@!"#$#%*?A%%  !"#$#%*?A%'!"#$#%*?A%& !"#$#%*?A%* | $ $ | 7B5(A,47 | 2B 8 47 7 ; H + !24 D D4 7 $D 7B5(A 9 |
| !"#$#%*?A%A | $ $1 4 | 7B5(A,47 | 9$ $1 4 H + !24 D D4  7 $D 7B5(A 9 |
| !"#$#%*?A'? | $ $ | 7B5(A,47 | 2B 8 47 7 ; H + !24 D D4 7 $D 7B5(A 9 |
| !"#$#%*?A'( | $ $1 4 | 7B5(A,47 | 9$ $1 4 H + !24 D D4 |

|  | $ $ | 7B5(A,47 | 2B 8 47 7 ; H + !24 D D4 7 $D 7B5(A 9 |
| --- | --- | --- | --- |
| !"#$#%*?A&' | $ $1 4 | 7B5(A,47 | 9$ $1 4 H + !24 D D4  7 $D 7B5(A 9 |
| !"#$#%*?A&&!"#$#%*?A&* | $ $ | 7B5(A,47 | 2B 8 47 7 ; H + !24 D D4 7 $D 7B5(A 9 |
| !"#$#%*?A&A!"#$#%*?A*? | $ $1 4 | 7B5(A,47 | 9$ $1 4 H + !24 D D4  7 $D 7B5(A 9 |
| !"#$#%*?A*(!"#$#%*?A*) !"#$#%*?A*>!"#$#%*?A*@ !"#$#%*?A*%!"#$#%*?A*'  !"#$#%*?A*&!"#$#%*?A** !"#$#%*?A*A | $ $ | 7B5(A,47 | 2B 8 47 7 ; H + !24 D D4 7 $D 7B5(A 9 |
| !"#$#%*?AA?!"#$#%*?AA( | $ $1 4 | 7B5(A,47 | 9$ $1 4 H + !24 D D4  7 $D 7B5(A 9 |
| !"#$#%*?AA) | $ $ | 7B5(A,47 | 2B 8 47 7 ; H + !24 D D4 7 $D 7B5(A 9 |
| !"#$#%*?AA> | $ $1 4 | 7B5(A,47 | 9$ $1 4 H + !24 D D4  7 $D 7B5(A 9 |

# 7 $D 7B5(A 9 !"#$#%*?A')!"#$#%*?A'>!"#$#%*?A'@!"#$#%*?A'%!"#$#%*?A''!"#$#%*?A'&!"#$#%*?A'*!"#$#%*?A'A!"#$#%*?A&?!"#$#%*?A&(!"#$#%*?A&)!"#$#%*?A&>!"#$#%*?A&@!"#$#%*?A&%

| !"#$#%*?AA@ | $ $ |  | 2B 8 47 7 ; H + !24 D D4 7 $D 7B5(A 9 |
| --- | --- | --- | --- |
| !"#$#%*?AA%!"#$#%*?AA' | $ $1 4 | 7B5(A,47 | 9$ $1 4 H + !24 D D4  7 $D 7B5(A 9 |
| !"#$#%*?AA& | $ $ | 7B5(A,47 | 2B 8 47 7 ; H + !24 D D4 7 $D 7B5(A 9 |
| !"#$#%*?AA* | $ $1 4 | 7B5(A,47 | 9$ $1 4 H + !24 D D4  7 $D 7B5(A 9 |
| !"#$#%*?AAA | $ $ | 7B5(A,47 | 2B 8 47 7 ; H + !24 D D4 7 $D 7B5(A 9 |
| !"#$#%*(??? | $ $1 4 | 7B5(A,47 | 9$ $1 4 H + !24 D D4  7 $D 7B5(A 9 |
| !"#$#%*(??(!"#$#%*(??) !"#$#%*(??> | $ $ | 7B5(A,47 | 2B 8 47 7 ; H + !24 D D4 7 $D 7B5(A 9 |
| !"#$#%*(??@ | $ $1 4 | 7B5(A,47 | 9$ $1 4 H + !24 D D4  7 $D 7B5(A 9 |
| !"#$#%*(??%!"#$#%*(??' !"#$#%*(??& | $ $ | 7B5(A,47 | 2B 8 47 7 ; H + !24 D D4 7 $D 7B5(A 9 |
| !"#$#%*(??*!"#$#%*(??A | $ $1 4 | 7B5(A,47 | 9$ $1 4 H + !24 D D4  7 $D 7B5(A 9 |
| !"#$#%*(?(?!"#$#%*(?((  !"#$#%*(?()!"#$#%*(?(> !"#$#%*(?(@!"#$#%*(?(%  !"#$#%*(?('!"#$#%*(?(& | $ $ | 7B5(A,47 | 2B 8 47 7 ; H + !24 D D4 7 $D 7B5(A 9 |
| !"#$#%*(?(* | $ $1 4 | 7B5(A,47 | 9$ $1 4 H + !24 D D4  7 $D 7B5(A 9 |
| !"#$#%*(?(A!"#$#%*(?)? !"#$#%*(?)(!"#$#%*(?)) | $ $ | 7B5(A,47 | 2B 8 47 7 ; H + !24 D D4 7 $D 7B5(A 9 |
| !"#$#%*(?)> | $ $1 4 | 7B5(A,47 | 9$ $1 4 H + !24 D D4  7 $D 7B5(A 9 |
| !"#$#%*(?)@ | $ $ | 7B5(A,47 | 2B 8 47 7 ; H + !24 D D4 7 $D 7B5(A 9 |
| !"#$#%*(?)%!"#$#%*(?)' | $ $1 4 | 7B5(A,47 | 9$ $1 4 H + !24 D D4  7 $D 7B5(A 9 |
| !"#$#%*(?)& | $ $ | 7B5(A,47 | 2B 8 47 7 ; H + !24 D D4 7 $D 7B5(A 9 |
| !"#$#%*(?)*!"#$#%*(?)A | $ $1 4 | 7B5(A,47 | 9$ $1 4 H + !24 D D4  7 $D 7B5(A 9 |
| !"#$#%*(?>?!"#$#%*(?>( | $ $ | 7B5(A,47 | 2B 8 47 7 ; H + !24 D D4 7 $D 7B5(A 9 |
| !"#$#%*(?>) | $ $1 4 | 7B5(A,47 | 9$ $1 4 H + !24 D D4  7 $D 7B5(A 9 |
| !"#$#%*(?>>!"#$#%*(?>@ | $ $ | 7B5(A,47 | 2B 8 47 7 ; H + !24 D D4 7 $D 7B5(A 9 |
| !"#$#%*(?>% | $ $1 4 | 7B5(A,47 | 9$ $1 4 H + !24 D D4  7 $D 7B5(A 9 |
| !"#$#%*(?>'!"#$#%*(?>& | $ $ | 7B5(A,47 | 2B 8 47 7 ; H + !24 D D4 7 $D 7B5(A 9 |
| !"#$#%*(?>* | $ $1 4 | 7B5(A,47 | 9$ $1 4 H + !24 D D4  7 $D 7B5(A 9 |
| !"#$#%*(?>A | $ $ | 7B5(A,47 | 2B 8 47 7 ; H + !24 D D4 7 $D 7B5(A 9 |
| !"#$#%*(?@?!"#$#%*(?@( | $ $1 4 | 7B5(A,47 | 9$ $1 4 H + !24 D D4  7 $D 7B5(A 9 |
| !"#$#%*(?@)!"#$#%*(?@> !"#$#%*(?@@ | $ $ | 7B5(A,47 | 2B 8 47 7 ; H + !24 D D4 7 $D 7B5(A 9 |
| !"#$#%*(?@' | $ $1 4 | 7B5(A,47 | 9$ $1 4 H + !24 D D4  7 $D 7B5(A 9 |
| !"#$#%*(?@&!"#$#%*(?@*  !"#$#%*(?@A!"#$#%*(?%?  !"#$#%*(?%(!"#$#%*(?%) !"#$#%*(?%> | $ $ | 7B5(A,47 | 2B 8 47 7 ; H + !24 D D4 7 $D 7B5(A 9 |
| !"#$#%*(?%@ | $ $1 4 | 7B5(A,47 | 9$ $1 4 H + !24 D D4  7 $D 7B5(A 9 |
| !"#$#%*(?%% | $ $ | 7B5(A,47 | 2B 8 47 7 ; H + !24 D D4 7 $D 7B5(A 9 |
| !"#$#%*(?%' | $ $1 4 | 7B5(A,47 | 9$ $1 4 H + !24 D D4  7 $D 7B5(A 9 |
| !"#$#%*(?%& | $ $ | 7B5(A,47 | 2B 8 47 7 ; H + !24 D D4 7 $D 7B5(A 9 |
| !"#$#%*(?%* | $ $1 4 | 7B5(A,47 | 9$ $1 4 H + !24 D D4  7 $D 7B5(A 9 |
| !"#$#%*(?%A!"#$#%*(?'?  !"#$#%*(?'(!"#$#%*(?') !"#$#%*(?'> | $ $ | 7B5(A,47 | 2B 8 47 7 ; H + !24 D D4 7 $D 7B5(A 9 |
| !"#$#%*(?'@!"#$#%*(?'% | $ $1 4 | 7B5(A,47 | 9$ $1 4 H + !24 D D4  7 $D 7B5(A 9 |
| !"#$#%*(?'' | $ $ | 7B5(A,47 | 2B 8 47 7 ; H + !24 D D4 7 $D 7B5(A 9 |
| !"#$#%*(?'& | $ $1 4 | 7B5(A,47 | 9$ $1 4 H + !24 D D4  7 $D 7B5(A 9 |
| !"#$#%*(?'* | $ $ | 7B5(A,47 | 2B 8 47 7 ; H + !24 D D4 7 $D 7B5(A 9 |
| !"#$#%*(?'A | $ $1 4 | 7B5(A,47 | 9$ $1 4 H + !24 D D4  7 $D 7B5(A 9 |
| !"#$#%*(?&?!"#$#%*(?&( !"#$#%*(?&)!"#$#%*(?&> | $ $ | 7B5(A,47 | 2B 8 47 7 ; H + !24 D D4 7 $D 7B5(A 9 |
| !"#$#%*(?&@ | $ $1 4 | 7B5(A,47 | 9$ $1 4 H + !24 D D4  7 $D 7B5(A 9 |
| !"#$#%*(?&% | $ $ | 7B5(A,47 | 2B 8 47 7 ; H + !24 D D4 7 $D 7B5(A 9 |
| !"#$#%*(?&' | $ $1 4 |  | 9$ $1 4 H + !24 D D4  7 $D 7B5(A 9 |
| !"#$#%*(?&& | $ $ |  | 2B 8 47 7 ; H + !24 D D4 7 $D 7B5(A 9 |
| !"#$#%*(?&* | $ $1 4 | 7B5(A,47 | 9$ $1 4 H + !24 D D4  7 $D 7B5(A 9 |
| !"#$#%*(?&A!"#$#%*(?*?  !"#$#%*(?*(!"#$#%*(?*) !"#$#%*(?*> | $ $ | 7B5(A,47 | 2B 8 47 7 ; H + !24 D D4 7 $D 7B5(A 9 |
| !"#$#%*(?*@ | $ $1 4 | 7B5(A,47 | 9$ $1 4 H + !24 D D4  7 $D 7B5(A 9 |
| !"#$#%*(?*% | $ $ | 7B5(A,47 | 2B 8 47 7 ; H + !24 D D4 7 $D 7B5(A 9 |
| !"#$#%*(?*' | $ $1 4 | 7B5(A,47 | 9$ $1 4 H + !24 D D4  7 $D 7B5(A 9 |
| !"#$#%*(?*&!"#$#%*(?**  !"#$#%*(?*A!"#$#%*(?A?  !"#$#%*(?A(!"#$#%*(?A) | $ $ | 7B5(A,47 | 2B 8 47 7 ; H + !24 D D4 7 $D 7B5(A 9 |
| !"#$#%*(?A> | $ $1 4 | 7B5(A,47 | 9$ $1 4 H + !24 D D4  7 $D 7B5(A 9 |
| !"#$#%*(?A@!"#$#%*(?A% !"#$#%*(?A' | $ $ | 7B5(A,47 | 2B 8 47 7 ; H + !24 D D4 7 $D 7B5(A 9 |
| !"#$#%*(?A& | $ $1 4 | 7B5(A,47 | 9$ $1 4 H + !24 D D4  7 $D 7B5(A 9 |
| !"#$#%*(?A* | $ $ | 7B5(A,47 | 2B 8 47 7 ; H + !24 D D4 7 $D 7B5(A 9 |
| !"#$#%*(?AA | $ $1 4 | 7B5(A,47 | 9$ $1 4 H + !24 D D4  7 $D 7B5(A 9 |
| !"#$#%*((??!"#$#%*((?( !"#$#%*((?) | $ $ | 7B5(A,47 | 2B 8 47 7 ; H + !24 D D4 7 $D 7B5(A 9 |
| !"#$#%*((?> | $ $1 4 | 7B5(A,47 | 9$ $1 4 H + !24 D D4  7 $D 7B5(A 9 |
| !"#$#%*((?@!"#$#%*((?% | $ $ | 7B5(A,47 | 2B 8 47 7 ; H + !24 D D4 7 $D 7B5(A 9 |
| !"#$#%*((?'!"#$#%*((?& | $ $1 4 | 7B5(A,47 | 9$ $1 4 H + !24 D D4  7 $D 7B5(A 9 |
| !"#$#%*((?*!"#$#%*((?A !"#$#%*(((? | $ $ | 7B5(A,47 | 2B 8 47 7 ; H + !24 D D4 7 $D 7B5(A 9 |
| !"#$#%*(((( | $ $1 4 | 7B5(A,47 | 9$ $1 4 H + !24 D D4  7 $D 7B5(A 9 |
| !"#$#%*((() | $ $ | 7B5(A,47 | 2B 8 47 7 ; H + !24 |

# D D4 7 $D 7B5(A 9 !"#$#%*(((>!"#$#%*(((@!"#$#%*(((%!"#$#%*((('!"#$#%*(((&!"#$#%*(((*!"#$#%*(((A!"#$#%*(()?!"#$#%*(()(!"#$#%*(())!"#$#%*(()>!"#$#%*(()@!"#$#%*(()%!"#$#%*(()'

|  | $ $1 4 | 7B5(A,47 | 9$ $1 4 H + !24 D D4  7 $D 7B5(A 9 |
| --- | --- | --- | --- |
| !"#$#%*(()& | $ $ | 7B5(A,47 | 2B 8 47 7 ; H + !24 D D4 7 $D 7B5(A 9 |
| !"#$#%*(()*!"#$#%*(()A !"#$#%*((>?!"#$#%*((>( | $ $1 4 | 7B5(A,47 | 9$ $1 4 H + !24 D D4  7 $D 7B5(A 9 |
| !"#$#%*((>)!"#$#%*((>> | $ $ | 7B5(A,47 | 2B 8 47 7 ; H + !24 D D4 7 $D 7B5(A 9 |
| !"#$#%*((>@!"#$#%*((>% !"#$#%*((>'!"#$#%*((>&  !"#$#%*((>*!"#$#%*((>A !"#$#%*((@? | $ $1 4 | 7B5(A,47 | 9$ $1 4 H + !24 D D4  7 $D 7B5(A 9 |
| !"#$#%*((@( | $ $ | 7B5(A,47 | 2B 8 47 7 ; H + !24 D D4 7 $D 7B5(A 9 |
| !"#$#%*((@)!"#$#%*((@> !"#$#%*((@@!"#$#%*((@% | $ $1 4 | 7B5(A,47 | 9$ $1 4 H + !24 D D4  7 $D 7B5(A 9 |
| !"#$#%*((@' | $ $ | 7B5(A,47 | 2B 8 47 7 ; H + !24 D D4 7 $D 7B5(A 9 |
| !"#$#%*((@&!"#$#%*((@*  !"#$#%*((%?!"#$#%*((%(  !"#$#%*((%)!"#$#%*((%> | $ $1 4 | 7B5(A,47 | 9$ $1 4 H + !24 D D4  7 $D 7B5(A 9 |
| !"#$#%*((%@ | $ $ | 7B5(A,47 | 2B 8 47 7 ; H + !24 |

# D D4 7 $D 7B5(A 9 !"#$#%*((%%!"#$#%*((%'!"#$#%*((%&!"#$#%*((%*!"#$#%*((%A!"#$#%*(('?!"#$#%*(('(!"#$#%*((')!"#$#%*(('>!"#$#%*(('@!"#$#%*(('%

|  | $ $1 4 | 7B5(A,47 | 9$ $1 4 H + !24 D D4  7 $D 7B5(A 9 |
| --- | --- | --- | --- |
| !"#$#%*(('' | $ $ | 7B5(A,47 | 2B 8 47 7 ; H + !24 D D4 7 $D 7B5(A 9 |
| !"#$#%*(('&!"#$#%*(('* !"#$#%*(('A!"#$#%*((&?  !"#$#%*((&(!"#$#%*((&) !"#$#%*((&> | $ $1 4 | 7B5(A,47 | 9$ $1 4 H + !24 D D4  7 $D 7B5(A 9 |
| !"#$#%*((&@ | $ $ | 7B5(A,47 | 2B 8 47 7 ; H + !24 D D4 7 $D 7B5(A 9 |
| !"#$#%*((&%!"#$#%*((&' !"#$#%*((&&!"#$#%*((&*  !"#$#%*((&A!"#$#%*((*? !"#$#%*((*( | $ $1 4 | 7B5(A,47 | 9$ $1 4 H + !24 D D4  7 $D 7B5(A 9 |
| !"#$#%*((*)!"#$#%*((*> | $ $ | 7B5(A,47 | 2B 8 47 7 ; H + !24 D D4 7 $D 7B5(A 9 |
| !"#$#%*((*@!"#$#%*((*%  !"#$#%*((*'!"#$#%*((*& !"#$#%*((** | $ $1 4 | 7B5(A,47 | 9$ $1 4 H + !24 D D4  7 $D 7B5(A 9 |
| !"#$#%*((*A | $ $ | 7B5(A,47 | 2B 8 47 7 ; H + !24 D D4 7 $D 7B5(A 9 |
| !"#$#%*((A?!"#$#%*((A( !"#$#%*((A) | $ $1 4 | 7B5(A,47 | 9$ $1 4 H + !24 D D4  7 $D 7B5(A 9 |
| !"#$#%*((A>!"#$#%*((A@ | $ $ | 7B5(A,47 | 2B 8 47 7 ; H + !24 D D4 7 $D 7B5(A 9 |

| !"#$#%*((A% | $ $1 4 |  | 9$ $1 4 H + !24 D D4  7 $D 7B5(A 9 |
| --- | --- | --- | --- |
| !"#$#%*((A' | $ $ | 7B5(A,47 | 2B 8 47 7 ; H + !24 |

# D D4 7 $D 7B5(A 9 !"#$#%*((A&!"#$#%*((A*!"#$#%*((AA!"#$#%*()??!"#$#%*()?(!"#$#%*()?)!"#$#%*()?>!"#$#%*()?@!"#$#%*()?%!"#$#%*()?'!"#$#%*()?&!"#$#%*()?*!"#$#%*()?A!"#$#%*()(?!"#$#%*()((!"#$#%*()()!"#$#%*()(>

|  | $ $1 4 | 7B5(A,47 | 9$ $1 4 H + !24 D D4  7 $D 7B5(A 9 |
| --- | --- | --- | --- |
| !"#$#%*()(@ | $ $ | 7B5(A,47 | 2B 8 47 7 ; H + !24 D D4 7 $D 7B5(A 9 |
| !"#$#%*()(%!"#$#%*()(' | $ $1 4 | 7B5(A,47 | 9$ $1 4 H + !24 D D4  7 $D 7B5(A 9 |
| !"#$#%*()(& | $ $ | 7B5(A,47 | 2B 8 47 7 ; H + !24 D D4 7 $D 7B5(A 9 |
| !"#$#%*()(*!"#$#%*()(A  !"#$#%*())?!"#$#%*())( !"#$#%*())) | $ $1 4 | 7B5(A,47 | 9$ $1 4 H + !24 D D4  7 $D 7B5(A 9 |
| !"#$#%*())> | $ $ | 7B5(A,47 | 2B 8 47 7 ; H + !24 D D4 7 $D 7B5(A 9 |
| !"#$#%*())@!"#$#%*())% | $ $1 4 | 7B5(A,47 | 9$ $1 4 H + !24 D D4  7 $D 7B5(A 9 |
| !"#$#%*())' | $ $ | 7B5(A,47 | 2B 8 47 7 ; H + !24 D D4 7 $D 7B5(A 9 |
| !"#$#%*())&!"#$#%*())* | $ $1 4 | 7B5(A,47 | 9$ $1 4 H + !24 D D4  7 $D 7B5(A 9 |
| !"#$#%*())A!"#$#%*()>? | $ $ | 7B5(A,47 | 2B 8 47 7 ; H + !24 D D4 7 $D 7B5(A 9 |
| !"#$#%*()>(!"#$#%*()>)!"#$#%*()>>!"#$#%*()>@!"#$#%*()>%!"#$#%*()>'!"#$#%*()>&!"#$#%*()>*!"#$#%*()>A!"#$#%*()@?!"#$#%*()@(!"#$#%*()@>!"#$#%*()@@ | | |
| $ $1 4 7B5(A,47 | | | 9$ $1 4 H + !24 D D4  7 $D 7B5(A 9 |
| !"#$#%*()@% $ $ 7B5(A,47 | | | 2B 8 47 7 ; H + !24 D D4 7 $D 7B5(A 9 |
| !"#$#%*()@' $ $1 4 7B5(A,47 | | | 9$ $1 4 H + !24 D D4  7 $D 7B5(A 9 |
| !"#$#%*()@& $ $ 7B5(A,47  !"#$#%*()@*!"#$#%*()@A!"#$#%*()%?!"#$#%*()%(!"#$#%*()%)!"#$#%*()%>!"#$#%*()%@!"#$#%*()%%!"#$#%*()%'!"#$#%*()%&!"#$#%*()%*!"#$#%*()%A!"#$#%*()'? | | | 2B 8 47 7 ; H + !24 D D4 7 $D 7B5(A 9 |
| $ $1 4 7B5(A,47 | | | 9$ $1 4 H + !24 D D4  7 $D 7B5(A 9 |
| !"#$#%*()') $ $ 7B5(A,47 | | | 2B 8 47 7 ; H + !24 D D4 7 $D 7B5(A 9 |
| !"#$#%*()'>!"#$#%*()'@ $ $1 4 7B5(A,47  !"#$#%*()'%!"#$#%*()'' | | | 9$ $1 4 H + !24 D D4  7 $D 7B5(A 9 |
| !"#$#%*()'& $ $ 7B5(A,47 | | | 2B 8 47 7 ; H + !24 |

# D D4 7 $D 7B5(A 9 !"#$#%*()'*!"#$#%*()'A!"#$#%*()&?!"#$#%*()&(!"#$#%*()&)!"#$#%*()&>!"#$#%*()&@!"#$#%*()&%!"#$#%*()&'!"#$#%*()&&!"#$#%*()&*!"#$#%*()&A!"#$#%*()*?!"#$#%*()*(!"#$#%*()*)!"#$#%*()*>!"#$#%*()*@

|  | $ $1 4 | 7B5(A,47 | 9$ $1 4 H + !24 D D4  7 $D 7B5(A 9 |
| --- | --- | --- | --- |
| !"#$#%*()*% | $ $ | 7B5(A,47 | 2B 8 47 7 ; H + !24 D D4 7 $D 7B5(A 9 |
| !"#$#%*()*'!"#$#%*()*&  !"#$#%*()**!"#$#%*()*A !"#$#%*()A? | $ $1 4 | 7B5(A,47 | 9$ $1 4 H + !24 D D4  7 $D 7B5(A 9 |
| !"#$#%*()A( | $ $ | 7B5(A,47 | 2B 8 47 7 ; H + !24 |

# D D4 7 $D 7B5(A 9 !"#$#%*()A)!"#$#%*()A>!"#$#%*()A@!"#$#%*()A%!"#$#%*()A'!"#$#%*()A&!"#$#%*()A*!"#$#%*()AA!"#$#%*(>??!"#$#%*(>?(!"#$#%*(>?)!"#$#%*(>?>!"#$#%*(>?@!"#$#%*(>?%!"#$#%*(>?'!"#$#%*(>?&!"#$#%*(>?*!"#$#%*(>?A

| $ $1 4 7B5(A,47 | | | 9$ $1 4 H + !24 D D4  7 $D 7B5(A 9 |
| --- | --- | --- | --- |
| !"#$#%*(>(? $ $ 7B5(A,47  !"#$#%*(>((!"#$#%*(>()!"#$#%*(>(>!"#$#%*(>(@!"#$#%*(>(%!"#$#%*(>('!"#$#%*(>(&!"#$#%*(>(*!"#$#%*(>(A!"#$#%*(>)?!"#$#%*(>)(!"#$#%*(>)) | | | 2B 8 47 7 ; H + !24 D D4 7 $D 7B5(A 9 |
|  | $ $1 4 | 7B5(A,47 | 9$ $1 4 H + !24 D D4  7 $D 7B5(A 9 |
| !"#$#%*(>)>!"#$#%*(>)@ | $ $ | 7B5(A,47 | 2B 8 47 7 ; H + !24 D D4 7 $D 7B5(A 9 |
| !"#$#%*(>)%!"#$#%*(>)' !"#$#%*(>)&!"#$#%*(>)* | $ $1 4 | 7B5(A,47 | 9$ $1 4 H + !24 D D4  7 $D 7B5(A 9 |
| !"#$#%*(>)A | $ $ | 7B5(A,47 | 2B 8 47 7 ; H + !24 D D4 7 $D 7B5(A 9 |
| !"#$#%*(>>?!"#$#%*(>>( !"#$#%*(>>)!"#$#%*(>>> | $ $1 4 | 7B5(A,47 | 9$ $1 4 H + !24 D D4  7 $D 7B5(A 9 |
| !"#$#%*(>>@ | $ $ | 7B5(A,47 | 2B 8 47 7 ; H + !24 D D4 7 $D 7B5(A 9 |
| !"#$#%*(>>%!"#$#%*(>>' | $ $1 4 | 7B5(A,47 | 9$ $1 4 H + !24 D D4  7 $D 7B5(A 9 |
| !"#$#%*(>>& | $ $ | 7B5(A,47 | 2B 8 47 7 ; H + !24 D D4 7 $D 7B5(A 9 |
| !"#$#%*(>>*!"#$#%*(>>A !"#$#%*(>@? | $ $1 4 | 7B5(A,47 | 9$ $1 4 H + !24 D D4  7 $D 7B5(A 9 |
| !"#$#%*(>@( | $ $ | 7B5(A,47 | 2B 8 47 7 ; H + !24 D D4 7 $D 7B5(A 9 |
| !"#$#%*(>@)!"#$#%*(>@> | $ $1 4 | 7B5(A,47 | 9$ $1 4 H + !24 D D4  7 $D 7B5(A 9 |
| !"#$#%*(>@@ | $ $ | 7B5(A,47 | 2B 8 47 7 ; H + !24 D D4 7 $D 7B5(A 9 |
| !"#$#%*(>@% | $ $1 4 | 7B5(A,47 | 9$ $1 4 H + !24 D D4  7 $D 7B5(A 9 |
| !"#$#%*(>@' | $ $ | 7B5(A,47 | 2B 8 47 7 ; H + !24 D D4 7 $D 7B5(A 9 |
| !"#$#%*(>@&!"#$#%*(>@* !"#$#%*(>@A | $ $1 4 | 7B5(A,47 | 9$ $1 4 H + !24 D D4  7 $D 7B5(A 9 |

| !"#$#%*(>%? | $ $ |  | 2B 8 47 7 ; H + !24 D D4 7 $D 7B5(A 9 |
| --- | --- | --- | --- |
| !"#$#%*(>%(!"#$#%*(>%) !"#$#%*(>%> | $ $1 4 |  | 9$ $1 4 H + !24 D D4  7 $D 7B5(A 9 |
| !"#$#%*(>%@ | $ $ | 7B5(A,47 | 2B 8 47 7 ; H + !24 D D4 7 $D 7B5(A 9 |
| !"#$#%*(>%%!"#$#%*(>%' !"#$#%*(>%& | $ $1 4 | 7B5(A,47 | 9$ $1 4 H + !24 D D4  7 $D 7B5(A 9 |
| !"#$#%*(>%* | $ $ | 7B5(A,47 | 2B 8 47 7 ; H + !24 D D4 7 $D 7B5(A 9 |
| !"#$#%*(>'?!"#$#%*(>'(  !"#$#%*(>')!"#$#%*(>'>  !"#$#%*(>'@!"#$#%*(>'% | $ $1 4 | 7B5(A,47 | 9$ $1 4 H + !24 D D4  7 $D 7B5(A 9 |
| !"#$#%*(>'& | $ $ " | 7B5(A,47 | D: 12 9$ $ " H + !24 D  D4 7 $D 7B5(A 9 |
| !"#$#%*(>'*!"#$#%*(>'A !"#$#%*(>&?!"#$#%*(>&( !"#$#%*(>&)!"#$#%*(>&>  !"#$#%*(>&@!"#$#%*(>&% !"#$#%*(>&' | $ $1 4 | 7B5(A,47 | 9$ $1 4 H + !24 D D4  7 $D 7B5(A 9 |
| !"#$#%*(>&& | $ $ | 7B5(A,47 | 2B 8 47 7 ; H + !24 D D4 7 $D 7B5(A 9 |
| !"#$#%*(>&*!"#$#%*(>&A !"#$#%*(>*? | $ $ " | 7B5(A,47 | D: 12 9$ $ " H + !24 D  D4 7 $D 7B5(A 9 |
| !"#$#%*(>*( | $ $ | 7B5(A,47 | 2B 8 47 7 ; H + !24 D D4 7 $D 7B5(A 9 |
| !"#$#%*(>*)!"#$#%*(>*> | $ $1 4 | 7B5(A,47 | 9$ $1 4 H + !24 D D4  7 $D 7B5(A 9 |
| !"#$#%*(>*@ | $ $ | 7B5(A,47 | 2B 8 47 7 ; H + !24 D D4 7 $D 7B5(A 9 |
| !"#$#%*(>*%!"#$#%*(>*' | $ $1 4 | 7B5(A,47 | 9$ $1 4 H + !24 D D4  7 $D 7B5(A 9 |
| !"#$#%*(>*& | $ $ | 7B5(A,47 | 2B 8 47 7 ; H + !24 D D4 7 $D 7B5(A 9 |
| !"#$#%*(>*A!"#$#%*(>A? | $ $1 4 | 7B5(A,47 | 9$ $1 4 H + !24 D D4  7 $D 7B5(A 9 |
| !"#$#%*(>A( | $ $ " | 7B5(A,47 | D: 12 9$ $ " H + !24 D  D4 7 $D 7B5(A 9 |
| !"#$#%*(>A) | $ $ | 7B5(A,47 | 2B 8 47 7 ; H + !24 D D4 7 $D 7B5(A 9 |
| !"#$#%*(>A> | $ $1 4 | 7B5(A,47 | 9$ $1 4 H + !24 D D4  7 $D 7B5(A 9 |
| !"#$#%*(>A@ | $ $7 | 7B5(A,47 | +29$ $7 H + !24 D D  4 7 $D 7B5(A 9 |
| !"#$#%*(>A% | $ $ | 7B5(A,47 | 2B 8 47 7 ; H + !24 D D4 7 $D 7B5(A 9 |
| !"#$#%*(>A' | $ $7 | 7B5(A,47 | +29$ $7 H + !24 D D  4 7 $D 7B5(A 9 |
| !"#$#%*(>A&!"#$#%*(>A*  !"#$#%*(>AA!"#$#%*(@?? !"#$#%*(@?( | $ $1 4 | 7B5(A,47 | 9$ $1 4 H + !24 D D4  7 $D 7B5(A 9 |
| !"#$#%*(@?)!"#$#%*(@?> | $ $ | 7B5(A,47 | 2B 8 47 7 ; H + !24 D D4 7 $D 7B5(A 9 |
| !"#$#%*(@?@ | $ $ " | 7B5(A,47 | D: 12 9$ $ " H + !24 D  D4 7 $D 7B5(A 9 |
| !"#$#%*(@?%!"#$#%*(@?' !"#$#%*(@?&!"#$#%*(@?* | $ $ | 7B5(A,47 | 2B 8 47 7 ; H + !24 D D4 7 $D 7B5(A 9 |
| !"#$#%*(@?A | $ $ " | 7B5(A,47 | D: 12 9$ $ " H + !24 D  D4 7 $D 7B5(A 9 |
| !"#$#%*(@(? | $ $ | 7B5(A,47 | 2B 8 47 7 ; H + !24 D D4 7 $D 7B5(A 9 |
| !"#$#%*(@((!"#$#%*(@() !"#$#%*(@(>!"#$#%*(@(@ | $ $ " | 7B5(A,47 | D: 12 9$ $ " H + !24 D  D4 7 $D 7B5(A 9 |
| !"#$#%*(@(% | $ $1 4 | 7B5(A,47 | 9$ $1 4 H + !24 D D4  7 $D 7B5(A 9 |
| !"#$#%*(@('!"#$#%*(@(& | $ $ " | 7B5(A,47 | D: 12 9$ $ " H + !24 D  D4 7 $D 7B5(A 9 |
| !"#$#%*(@(*!"#$#%*(@(A !"#$#%*(@)? | $ $ | 7B5(A,47 | 2B 8 47 7 ; H + !24 D D4 7 $D 7B5(A 9 |
| !"#$#%*(@)(!"#$#%*(@)) | $ $1 4 | 7B5(A,47 | 9$ $1 4 H + !24 D D4  7 $D 7B5(A 9 |
| !"#$#%*(@)@!"#$#%*(@)' !"#$#%*(@)& | $ $ " | 7B5(A,47 | D: 12 9$ $ " H + !24 D  D4 7 $D 7B5(A 9 |
| !"#$#%*(@)* | $ $1 4 | 7B5(A,47 | 9$ $1 4 H + !24 D D4  7 $D 7B5(A 9 |
| !"#$#%*(@)A!"#$#%*(@>?  !"#$#%*(@>(!"#$#%*(@>) !"#$#%*(@>> | $ $ " | 7B5(A,47 | D: 12 9$ $ " H + !24 D  D4 7 $D 7B5(A 9 |
| !"#$#%*(@>@ | $ $1 4 | 7B5(A,47 | 9$ $1 4 H + !24 D D4  7 $D 7B5(A 9 |
| !"#$#%*(@>%!"#$#%*(@>' | $ $ " | 7B5(A,47 | D: 12 9$ $ " H + !24 D  D4 7 $D 7B5(A 9 |
| !"#$#%*(@>& | $ $ | 7B5(A,47 | 2B 8 47 7 ; H + !24 D D4 7 $D 7B5(A 9 |
| !"#$#%*(@>* | $ $1 4 | 7B5(A,47 | 9$ $1 4 H + !24 D D4  7 $D 7B5(A 9 |
| !"#$#%*(@>A | $ $ | 7B5(A,47 | 2B 8 47 7 ; H + !24 D D4 7 $D 7B5(A 9 |
| !"#$#%*(@@? | $ $1 4 | 7B5(A,47 | 9$ $1 4 H + !24 D D4  7 $D 7B5(A 9 |
| !"#$#%*(@@( | $ $ | 7B5(A,47 | 2B 8 47 7 ; H + !24 |

|  |  |  | D D4 7 $D 7B5(A 9 |
| --- | --- | --- | --- |
| !"#$#%*(@@) | $ $ " | 7B5(A,47 | D: 12 9$ $ " H + !24 D  D4 7 $D 7B5(A 9 |
| !"#$#%*(@@> | $ $ | 7B5(A,47 | 2B 8 47 7 ; H + !24 D D4 7 $D 7B5(A 9 |
| !"#$#%*(@@@!"#$#%*(@@% !"#$#%*(@@' | $ $ " | 7B5(A,47 | D: 12 9$ $ " H + !24 D  D4 7 $D 7B5(A 9 |
| !"#$#%*(@@& | $ $1 4 | 7B5(A,47 | 9$ $1 4 H + !24 D D4  7 $D 7B5(A 9 |
| !"#$#%*(@@* | $ $ " | 7B5(A,47 | D: 12 9$ $ " H + !24 D  D4 7 $D 7B5(A 9 |
| !"#$#%*(@@A!"#$#%*(@%? !"#$#%*(@%( | 7+5 7 < 1!7 2 4 | 7+5 7 < 1!7 2 4 | -41" 1 < $ + 1 +-" " "7 7 |
| !"#$#%*(@%)!"#$#%*(@%> !"#$#%*(@%@ | 1;4 1; ,Y 9Z | 3779Z Z1;1 2,Y 9Z |  |
| !"#$#%*(@%% | 07  .07+1/ | 3779Z Z1;1 2,Y 9Z |  |
| !"#$#%*(@%'!"#$#%*(@%&  !"#$#%*(@%*!"#$#%*(@%A  !"#$#%*(@'?!"#$#%*(@'( | 1;4 1; ,Y 9Z | 3779Z Z1;1 2,Y 9Z |  |
| !"#$#%*(@') | 07  .07+1/ | 3779Z Z1;1 2,Y 9Z |  |
| !"#$#%*(@'>!"#$#%*(@'@!"#$#%*(@'%!"#$#%*(@''!"#$#%*(@&*!"#$#%*(@&A!"#$#%*(@*?!"#$#%*(@*(!"#$#%*(@*)!"#$#%*(@*>!"#$#%*(@*@!"#$#%*(@*% | | |
| 1;4 1; 3779Z Z1;1 2,Y 9Z  ,Y 9Z | | |  |
| !"#$#%*(@*'!"#$#%*(@*& 07 3779Z Z1;1 2,Y 9Z  !"#$#%*(@**!"#$#%*(@*A .07+1/  !"#$#%*(@A?!"#$#%*(@A(  !"#$#%*(@A)!"#$#%*(@A> | | |  |
| !"#$#%*(@A@!"#$#%*(@A% 7+5 7 < 1!7 7+5 7 < 1!7 2 4  !"#$#%*(@A'!"#$#%*(@A& 2 4  !"#$#%*(@AA!"#$#%*(%??  !"#$#%*(%?(!"#$#%*(%?) | | | -41" 1 < $ + 1 +-" " "7 7 |
| !"#$#%*(%?>!"#$#%*(%?@ 7+5 7 < 1!7 7+5 7 < 1!7 2 4  2 4 | | | -41" 1 < $ + 1 +-" " "7 7 |
| !"#$#%*(%?%!"#$#%*(%?' 377I< 377I<  !"#$#%*(%?& | | | B 1 <, 3 9"- 4 9+4B+1 ; 4-  9 3 +-"410 : - "+ |

# !"#$#%*(%?*!"#$#%*(%?A!"#$#%*(%(?!"#$#%*(%((!"#$#%*(%()!"#$#%*(%(>!"#$#%*(%(@!"#$#%*(%(%!"#$#%*(%('!"#$#%*(%(&!"#$#%*(%(*!"#$#%*(%)?!"#$#%*(%))!"#$#%*(%)>!"#$#%*(%)@!"#$#%*(%)%!"#$#%*(%)'!"#$#%*(%)&!"#$#%*(%)*!"#$#%*(%)A!"#$#%*(%>?!"#$#%*(%>(!"#$#%*(%>)!"#$#%*(%>> !"#$#%*(%>@!"#$#%*(%>%!"#$#%*(%>'!"#$#%*(%>&!"#$#%*(%>*!"#$#%*(%>A!"#$#%*(%@?!"#$#%*(%@(!"#$#%*(%@)!"#$#%*(%@>!"#$#%*(%@@!"#$#%*(%@%!"#$#%*(%@'!"#$#%*(%@&!"#$#%*(%@*!"#$#%*(%@A!"#$#%*(%%?!"#$#%*(%%(!"#$#%*(%%)!"#$#%*(%%>!"#$#%*(%%@!"#$#%*(%%%!"#$#%*(%%'!"#$#%*(%%& !"#$#%*(%%*!"#$#%*(%%A!"#$#%*(%'?!"#$#%*(%'(!"#$#%*(%')!"#$#%*(%'>!"#$#%*(%'@!"#$#%*(%'%!"#$#%*(%''!"#$#%*(%'&!"#$#%*(%'*!"#$#%*(%'A!"#$#%*(%&?!"#$#%*(%&(!"#$#%*(%&)!"#$#%*(%&>!"#$#%*(%&@ B7$ B7$ B7$ !"#$#%*(%&%!"#$#%*(%&'!"#$#%*(%&&!"#$#%*(%&*!"#$#%*(%&A!"#$#%*(%*?!"#$#%*(%*(!"#$#%*(%*)!"#$#%*(%*>!"#$#%*(%*@!"#$#%*(%*%!"#$#%*(%*'!"#$#%*(%A?!"#$#%*(%A(!"#$#%*(%A)!"#$#%*(%A>!"#$#%*(%A@!"#$#%*(%A%!"#$#%*(%A'!"#$#%*(%A&!"#$#%*(%A*!"#$#%*(%AA!"#$#%*('??!"#$#%*('?( !"#$#%*('?)!"#$#%*('?>!"#$#%*('?@!"#$#%*('?%!"#$#%*('?'!"#$#%*('?&!"#$#%*('?*!"#$#%*('?A!"#$#%*('(?!"#$#%*('((!"#$#%*('()!"#$#%*('(>!"#$#%*('(@!"#$#%*('(%!"#$#%*('('!"#$#%*('(&!"#$#%*('(*!"#$#%*('(A!"#$#%*(')?!"#$#%*(')(!"#$#%*('))!"#$#%*(')>!"#$#%*(')@!"#$#%*(')% !"#$#%*(')'!"#$#%*(')&!"#$#%*(')*!"#$#%*(')A!"#$#%*('>?!"#$#%*('>(!"#$#%*('>)!"#$#%*('>>!"#$#%*('>@!"#$#%*('>%!"#$#%*('>'!"#$#%*('>&!"#$#%*('>*!"#$#%*('>A!"#$#%*('@?!"#$#%*('@(!"#$#%*('@)!"#$#%*('@>!"#$#%*('@@!"#$#%*('@%!"#$#%*('@'!"#$#%*('@&!"#$#%*('@*!"#$#%*('@A !"#$#%*('%?!"#$#%*('%(!"#$#%*('%)!"#$#%*('%>!"#$#%*('%@!"#$#%*('%%!"#$#%*('%'!"#$#%*('%&!"#$#%*('%*!"#$#%*('%A!"#$#%*(''?!"#$#%*(''(!"#$#%*('')!"#$#%*(''>!"#$#%*(''@!"#$#%*(''%!"#$#%*('''!"#$#%*(''& 7 1 1 4 1W<+[ < H< < - 7W 1H"1 15"2B< !"#$#%*(''*!"#$#%*(''A!"#$#%*('&?!"#$#%*('&(!"#$#%*('&)!"#$#%*('&>!"#$#%*('&@!"#$#%*('&%!"#$#%*('&'!"#$#%*('&&!"#$#%*('&*!"#$#%*('&A!"#$#%*('*(!"#$#%*('*)!"#$#%*('*>!"#$#%*('*@!"#$#%*('*%!"#$#%*('*'!"#$#%*('*&!"#$#%*('*A!"#$#%*('A?!"#$#%*('A(!"#$#%*('A>!"#$#%*('A@ !"#$#%*('A%!"#$#%*('A'!"#$#%*('A&!"#$#%*('A*!"#$#%*('AA!"#$#%*(&??!"#$#%*(&?(!"#$#%*(&?)!"#$#%*(&?>!"#$#%*(&?%!"#$#%*(&?'!"#$#%*(&?&!"#$#%*(&?*!"#$#%*(&?A!"#$#%*(&(?!"#$#%*(&((!"#$#%*(&()!"#$#%*(&(@!"#$#%*(&(%!"#$#%*(&('!"#$#%*(&(&!"#$#%*(&(*!"#$#%*(&(A!"#$#%*(&)? !"#$#%*(&)(!"#$#%*(&)>!"#$#%*(&)'!"#$#%*(&)&!"#$#%*(&)*!"#$#%*(&)A!"#$#%*(&>?!"#$#%*(&>(!"#$#%*(&>)!"#$#%*(&>@!"#$#%*(&>%!"#$#%*(&>'!"#$#%*(&>&!"#$#%*(&>*!"#$#%*(&>A!"#$#%*(&@(!"#$#%*(&@)!"#$#%*(&@>!"#$#%*(&@@!"#$#%*(&@%!"#$#%*(&@'!"#$#%*(&@&!"#$#%*(&@*!"#$#%*(&@A !"#$#%*(&%?!"#$#%*(&%(!"#$#%*(&%)!"#$#%*(&%>!"#$#%*(&%@!"#$#%*(&%'!"#$#%*(&%&!"#$#%*(&%*!"#$#%*(&%A!"#$#%*(&'?!"#$#%*(&'(!"#$#%*(&')!"#$#%*(&'>!"#$#%*(&'@!"#$#%*(&'%!"#$#%*(&''!"#$#%*(&'&!"#$#%*(&'*!"#$#%*(&'A!"#$#%*(&&?!"#$#%*(&&)!"#$#%*(&&>!"#$#%*(&&%!"#$#%*(&&' !"#$#%*(&&&!"#$#%*(&&*!"#$#%*(&&A!"#$#%*(&*?!"#$#%*(&*(!"#$#%*(&*)!"#$#%*(&*>!"#$#%*(&*@!"#$#%*(&*%!"#$#%*(&*'!"#$#%*(&*&!"#$#%*(&**!"#$#%*(&*A!"#$#%*(&A(!"#$#%*(&A)!"#$#%*(&A>!"#$#%*(&A@!"#$#%*(&A%!"#$#%*(&A'!"#$#%*(&A&!"#$#%*(&A*!"#$#%*(&AA!"#$#%*(*??!"#$#%*(*?( !"#$#%*(*?)!"#$#%*(*?>!"#$#%*(*?@!"#$#%*(*?%!"#$#%*(*?'!"#$#%*(*?&!"#$#%*(*?*!"#$#%*(*?A!"#$#%*(*(?!"#$#%*(*((!"#$#%*(*()!"#$#%*(*(>!"#$#%*(*(@!"#$#%*(*(%!"#$#%*(*('!"#$#%*(*(*!"#$#%*(*(A!"#$#%*(*)?!"#$#%*(*)(!"#$#%*(*))!"#$#%*(*)>!"#$#%*(*)@!"#$#%*(*)%!"#$#%*(*)' !"#$#%*(*)&!"#$#%*(*)*!"#$#%*(*)A!"#$#%*(*>?!"#$#%*(*>)!"#$#%*(*>>!"#$#%*(*>@!"#$#%*(*>%!"#$#%*(*>'!"#$#%*(*>&!"#$#%*(*>*!"#$#%*(*>A!"#$#%*(*@?!"#$#%*(*@(!"#$#%*(*@)!"#$#%*(*@>!"#$#%*(*@@!"#$#%*(*@'!"#$#%*(*@&!"#$#%*(*@*!"#$#%*(*@A!"#$#%*(*%?!"#$#%*(*%(!"#$#%*(*%) !"#$#%*(*%>!"#$#%*(*%@!"#$#%*(*%%!"#$#%*(*%'!"#$#%*(*%&!"#$#%*(*%A!"#$#%*(*'?!"#$#%*(*'(!"#$#%*(*'>!"#$#%*(*'@!"#$#%*(*'%!"#$#%*(*''!"#$#%*(*'&!"#$#%*(*'*!"#$#%*(*'A!"#$#%*(*&?!"#$#%*(*&(!"#$#%*(*&)!"#$#%*(*&>!"#$#%*(*&@!"#$#%*(*&%!"#$#%*(*&'!"#$#%*(*&&!"#$#%*(*&* !"#$#%*(*&A!"#$#%*(**?!"#$#%*(**(!"#$#%*(**>!"#$#%*(**@!"#$#%*(**%!"#$#%*(**'!"#$#%*(***!"#$#%*(**A!"#$#%*(*A(!"#$#%*(*A)!"#$#%*(*A>!"#$#%*(*A@!"#$#%*(*A%!"#$#%*(*A'!"#$#%*(*A&!"#$#%*(*A*!"#$#%*(*AA!"#$#%*(A??!"#$#%*(A?(!"#$#%*(A?)!"#$#%*(A?>!"#$#%*(A?@!"#$#%*(A?% !"#$#%*(A?'!"#$#%*(A?&!"#$#%*(A?*!"#$#%*(A?A!"#$#%*(A(?!"#$#%*(A((!"#$#%*(A(>!"#$#%*(A(%!"#$#%*(A('!"#$#%*(A(&!"#$#%*(A(*!"#$#%*(A(A!"#$#%*(A)?!"#$#%*(A)(!"#$#%*(A))!"#$#%*(A)>!"#$#%*(A)@!"#$#%*(A)%!"#$#%*(A)'!"#$#%*(A)&!"#$#%*(A)*!"#$#%*(A)A!"#$#%*(A>?!"#$#%*(A>( !"#$#%*(A>)!"#$#%*(A>>!"#$#%*(A>@!"#$#%*(A>%!"#$#%*(A>'!"#$#%*(A>&!"#$#%*(A>*!"#$#%*(A>A!"#$#%*(A@?!"#$#%*(A@(!"#$#%*(A@)!"#$#%*(A@>!"#$#%*(A@@!"#$#%*(A@%!"#$#%*(A@'!"#$#%*(A@&!"#$#%*(A@*!"#$#%*(A@A!"#$#%*(A%?!"#$#%*(A%(!"#$#%*(A%)!"#$#%*(A%>!"#$#%*(A%@!"#$#%*(A%% !"#$#%*(A%'!"#$#%*(A%&!"#$#%*(A%*!"#$#%*(A%A!"#$#%*(A'?!"#$#%*(A'(!"#$#%*(A')!"#$#%*(A'>!"#$#%*(A'@!"#$#%*(A'%!"#$#%*(A''!"#$#%*(A'&!"#$#%*(A'*!"#$#%*(A'A!"#$#%*(A&?!"#$#%*(A&(!"#$#%*(A&)!"#$#%*(A&>!"#$#%*(A&%!"#$#%*(A&'!"#$#%*(A&&!"#$#%*(A&*!"#$#%*(A&A!"#$#%*(A*? !"#$#%*(A*(!"#$#%*(A*)!"#$#%*(A*>!"#$#%*(A*@!"#$#%*(A*%!"#$#%*(A*'!"#$#%*(A*&!"#$#%*(A**!"#$#%*(A*A!"#$#%*(AA?!"#$#%*(AA(!"#$#%*(AA)!"#$#%*(AA>!"#$#%*(AA@!"#$#%*(AA%!"#$#%*(AA'!"#$#%*(AA&!"#$#%*(AA*!"#$#%*(AAA!"#$#%*)???!"#$#%*)??(!"#$#%*)??)

|  | ,2 < 7 B | ,2 < 7 < | 1 19+ 2 1<51 ;1< 4 $ ;44 H 9 5 0 D < 2"1 73 + <1 <+ 5 1 <22! |
| --- | --- | --- | --- |
| !"#$#%*)?(& | $"$, | ! +.!+/ | J +13 01 0 D2";--$ ,71 2  1 2D,D D0D 2D ! 1 1<  1+1H< 1 1 < +D1 1 9 9< BB" B2! $ D D$ |
| !"#$#%*)?(*!"#$#%*)?(A !"#$#%*)?)?!"#$#%*)?)( | "$<" | ! +.!+/ | J +13 01 0 D2";--$ ,71 2  1 2D,D D0D 2D ! 1 1<  1+1H< 1 1 < +D1 1 9 9< BB" B2! $ D D$ |
| !"#$#%*)?))!"#$#%*)?)> | " $ | ! +.!+/ | J +13 01 0 D2";--$ ,71 2  1 2D,D D0D 2D ! 1 1<  1+1H< 1 1 < +D1 1 9 9< BB" B2! $ D D$ |
| !"#$#%*)?)& | 7 1 | 1 | 4 1W<+[ < H< < - 7W 1H"1 15"2B< |
| !"#$#%*)?)* | 7+5 7 < 1!7 2 4 | 7+5 7 < 1!7 2 4 | -41" 1 < $ + 1 +-" " "7 7 |
| !"#$#%*)?)A!"#$#%*)?>? | < 7 51  , | 7!< .7!</+$" | 3 5+ $-+- 1 1 |
| !"#$#%*)?>(!"#$#%*)?>) | 2 " | 2 " | ;2;,; 14; 5+\! $!F] ;1^;-"^ |
| !"#$#%*)?>> | " 1  42 9,1 | +7 , + | F01;14-D ;; 1; D ;2 ; D ;  ; 1; +;3- 44;1D;23- |

!"#$#%*)?>@!"#$#%*)?>%!"#$#%*)?>'!"#$#%*)?>&!"#$#%*)?>*!"#$#%*)?>A!"#$#%*)?@?!"#$#%*)?@(!"#$#%*)?@)!"#$#%*)?@>!"#$#%*)?@@!"#$#%*)?@%!"#$#%*)?@*!"#$#%*)?@A!"#$#%*)?%?!"#$#%*)?%(!"#$#%*)?%)!"#$#%*)?%>!"#$#%*)?%@!"#$#%*)?%%!"#$#%*)?%'!"#$#%*)?%&!"#$#%*)?%*!"#$#%*)?%A !"#$#%*)?'?!"#$#%*)?'(!"#$#%*)?')!"#$#%*)?'>!"#$#%*)?'@!"#$#%*)?'%!"#$#%*)?''!"#$#%*)?'&!"#$#%*)?'*!"#$#%*)?'A!"#$#%*)?&?!"#$#%*)?&(!"#$#%*)?&)!"#$#%*)?&>!"#$#%*)?&@!"#$#%*)?&%!"#$#%*)?&'!"#$#%*)?&&!"#$#%*)?&*!"#$#%*)?&A!"#$#%*)?*?!"#$#%*)?*(!"#$#%*)?*)!"#$#%*)?*> !"#$#%*)?*@!"#$#%*)?*%!"#$#%*)?*'!"#$#%*)?*&!"#$#%*)?**!"#$#%*)?*A!"#$#%*)?A?

# 1 L 2 , :7B5"3 I<B.77/ 7 1 1$ "_D11O:7B5"3 N! O++ O < !"#$#%*)?A(!"#$#%*)?A)!"#$#%*)?A>!"#$#%*)?A@!"#$#%*)?A&!"#$#%*)?A*!"#$#%*)?AA!"#$#%*)(??!"#$#%*)(?(!"#$#%*)(?)!"#$#%*)(?>!"#$#%*)(?@!"#$#%*)(?%!"#$#%*)(?'!"#$#%*)(?&!"#$#%*)(?*!"#$#%*)(?A 2 ,1: WB 5B$ :7B5"3 I<B.77/ 1L! 7L1O; 1Z D1 1W;$; " 1D`+L ;+L ;1L + ;:7B5

| .7/  !"#$#%*)((?!"#$#%*)(((!"#$#%*)(()!"#$#%*)((>!"#$#%*)((@!"#$#%*)((%!"#$#%*)(('!"#$#%*)((&!"#$#%*)((*!"#$#%*)((A!"#$#%*)()? | | | "3 |
| --- | --- | --- | --- |
|  | 73+B +50, | 73+B +50, | < < 2+a H1 < 5 0 151B < $$ D |
| !"#$#%*)()( | 72 < < | 73+B +50, | < < 2+a H1 < 5 0 151B < $$ D |
| !"#$#%*)()) | 73+B +50, | 73+B +50, | < < 2+a H1 < 5 0 151B < $$ D |
| !"#$#%*)()> | ,71 B ; , | < 1 | 1;7 "; 7 ";;0 |
| !"#$#%*)()@ | 1 | 1 | 131 8 1 33 ;1: 10; < !;;43 ;3;D 2F |
| !"#$#%*)()%!"#$#%*)()' | 4 1 7 | 1 4 1 7 | -0- 2 ;:2 |
| !"#$#%*)()&!"#$#%*)()*  !"#$#%*)()A!"#$#%*)(>?  !"#$#%*)(>(!"#$#%*)(>) | ,2 | 9 1 | " 7 |
| !"#$#%*)(>> | " 2 $ | " 2 $ | 2 |
| !"#$#%*)(>@ | ";$ | ";$ | , ";2! 9 1H4 H4 D |

# !"#$#%*)(@*!"#$#%*)(@A!"#$#%*)(%?!"#$#%*)(%(!"#$#%*)(%)!"#$#%*)(%>!"#$#%*)(%%!"#$#%*)(%'!"#$#%*)(%&!"#$#%*)(%*!"#$#%*)('(!"#$#%*)('>!"#$#%*)(''!"#$#%*)('&!"#$#%*)('*!"#$#%*)(&?!"#$#%*)(&(!"#$#%*)(&*!"#$#%*)(*>!"#$#%*)(*&

| 97+$ 7 2 ,31H2 7  !"#$#%*))))!"#$#%*)))>!"#$#%*)))@!"#$#%*)))%!"#$#%*)))'!"#$#%*)))*!"#$#%*)))A!"#$#%*))>?!"#$#%*))>>!"#$#%*))>%!"#$#%*))>'!"#$#%*))>&!"#$#%*))>* | 4 9 4 D2 1 ! |
| --- | --- |
| " 2 $ 7 2 ,31H2 7 | 4 +71 7 32 |
| !"#$#%*))@( 3B ; 3B ; | "B |

# !"#$#%*))@)!"#$#%*))@>!"#$#%*))@@!"#$#%*))@%!"#$#%*))@'!"#$#%*))@&!"#$#%*))@*!"#$#%*))@A!"#$#%*))%(!"#$#%*))%)!"#$#%*))%>!"#$#%*))%@!"#$#%*))%%!"#$#%*))%'!"#$#%*))%&!"#$#%*))%*!"#$#%*))%A!"#$#%*))'?!"#$#%*))'(!"#$#%*))')!"#$#%*))'>!"#$#%*))'@!"#$#%*))'%!"#$#%*))'' !"#$#%*))'&!"#$#%*))'*!"#$#%*))'A!"#$#%*))&?!"#$#%*))&(!"#$#%*))&)!"#$#%*))&>!"#$#%*))&@!"#$#%*))&%!"#$#%*))&'!"#$#%*))&&!"#$#%*))&*!"#$#%*))&A!"#$#%*))*?!"#$#%*))*(!"#$#%*))*)!"#$#%*))*>!"#$#%*))*@!"#$#%*))*%!"#$#%*))*'!"#$#%*))**!"#$#%*))*A!"#$#%*))A?!"#$#%*))A( !"#$#%*))A)!"#$#%*))A>!"#$#%*))A@!"#$#%*))A%!"#$#%*))A'!"#$#%*))A&!"#$#%*))A*!"#$#%*))AA!"#$#%*)>??!"#$#%*)>?(!"#$#%*)>?)!"#$#%*)>?>!"#$#%*)>?@!"#$#%*)>?%!"#$#%*)>?'!"#$#%*)>?*!"#$#%*)>((!"#$#%*)>()!"#$#%*)>(>!"#$#%*)>(@!"#$#%*)>(%!"#$#%*)>('!"#$#%*)>(&!"#$#%*)>(* !"#$#%*)>(A!"#$#%*)>)?!"#$#%*)>)(!"#$#%*)>))!"#$#%*)>)>!"#$#%*)>)@!"#$#%*)>)%!"#$#%*)>)'!"#$#%*)>)&!"#$#%*)>)*!"#$#%*)>)A!"#$#%*)>>?!"#$#%*)>>(!"#$#%*)>>)!"#$#%*)>>>!"#$#%*)>>@!"#$#%*)>>%!"#$#%*)>>'!"#$#%*)>>&!"#$#%*)>>*!"#$#%*)>>A!"#$#%*)>@?!"#$#%*)>@(!"#$#%*)>@) !"#$#%*)>@>!"#$#%*)>@%!"#$#%*)>@'!"#$#%*)>@&!"#$#%*)>@A!"#$#%*)>%?!"#$#%*)>%(!"#$#%*)>%)!"#$#%*)>%>!"#$#%*)>%@!"#$#%*)>%%!"#$#%*)>%'!"#$#%*)>%&!"#$#%*)>%*!"#$#%*)>%A!"#$#%*)>'?!"#$#%*)>'(!"#$#%*)>')!"#$#%*)>'>!"#$#%*)>'@!"#$#%*)>'%!"#$#%*)>''!"#$#%*)>'&!"#$#%*)>'* !"#$#%*)>'A!"#$#%*)>&?!"#$#%*)>&(!"#$#%*)>&)!"#$#%*)>&>!"#$#%*)>&@!"#$#%*)>&%!"#$#%*)>&'!"#$#%*)>&&!"#$#%*)>&*!"#$#%*)>&A!"#$#%*)>*?!"#$#%*)>*(!"#$#%*)>*)!"#$#%*)>*>!"#$#%*)>*@!"#$#%*)>*%!"#$#%*)>*'!"#$#%*)>*&!"#$#%*)>**!"#$#%*)>*A!"#$#%*)>A?!"#$#%*)>A(!"#$#%*)>A) !"#$#%*)>A>!"#$#%*)>A@!"#$#%*)>A%!"#$#%*)>A'!"#$#%*)>A&!"#$#%*)>AA!"#$#%*)@??!"#$#%*)@?(!"#$#%*)@?)!"#$#%*)@?>!"#$#%*)@?@!"#$#%*)@?%!"#$#%*)@?'!"#$#%*)@?&!"#$#%*)@?*!"#$#%*)@?A!"#$#%*)@(?!"#$#%*)@((!"#$#%*)@()!"#$#%*)@(>!"#$#%*)@(@!"#$#%*)@('!"#$#%*)@(&!"#$#%*)@(* !"#$#%*)@(A!"#$#%*)@)?!"#$#%*)@))!"#$#%*)@)>!"#$#%*)@)@!"#$#%*)@)%!"#$#%*)@)'!"#$#%*)@)&!"#$#%*)@)*!"#$#%*)@)A!"#$#%*)@>?!"#$#%*)@>(!"#$#%*)@>)!"#$#%*)@>>!"#$#%*)@>@!"#$#%*)@>%!"#$#%*)@>'!"#$#%*)@>&!"#$#%*)@>*!"#$#%*)@>A!"#$#%*)@@?!"#$#%*)@@(!"#$#%*)@@)!"#$#%*)@@> !"#$#%*)@@%!"#$#%*)@@'!"#$#%*)@@&!"#$#%*)@@*!"#$#%*)@@A!"#$#%*)@%?!"#$#%*)@%(!"#$#%*)@%)!"#$#%*)@%>!"#$#%*)@%@!"#$#%*)@%%!"#$#%*)@%'!"#$#%*)@%&!"#$#%*)@%*!"#$#%*)@%A!"#$#%*)@'?!"#$#%*)@'(!"#$#%*)@')!"#$#%*)@'>!"#$#%*)@'@!"#$#%*)@'%!"#$#%*)@''!"#$#%*)@'&!"#$#%*)@'* !"#$#%*)@'A!"#$#%*)@&?!"#$#%*)@&(!"#$#%*)@&)!"#$#%*)@&>!"#$#%*)@&@!"#$#%*)@&%!"#$#%*)@&'!"#$#%*)@&&!"#$#%*)@&*!"#$#%*)@&A!"#$#%*)@*?!"#$#%*)@*(!"#$#%*)@*)!"#$#%*)@*>!"#$#%*)@*@!"#$#%*)@*%!"#$#%*)@*'!"#$#%*)@*&!"#$#%*)@**!"#$#%*)@*A!"#$#%*)@A?!"#$#%*)@A(!"#$#%*)@A) !"#$#%*)@A>!"#$#%*)@A@!"#$#%*)@A%!"#$#%*)@A'!"#$#%*)@A&!"#$#%*)@A*!"#$#%*)@AA!"#$#%*)%??!"#$#%*)%?(!"#$#%*)%?)!"#$#%*)%?>!"#$#%*)%?@!"#$#%*)%?%!"#$#%*)%?'!"#$#%*)%?&

|  | 7" $ | 3 1 $.31$/ | 1-9" 1! +2 ;+ 1 $D93 4H1  B " B7 D< H4 3 <8$9<2 17 773X " 2 7773 |
| --- | --- | --- | --- |
| !"#$#%*)%?* | 73+B +50, | 73+B +50, | < < 2+a H1 < 5 0 151B < $$ D |
| !"#$#%*)%?A | +B  $ B ! C 31, | +B $ B ! C 31 , D7E0 E | 1 4 $ < $+ 3 9 |
| !"#$#%*)%(? | +B  $ B ! C 31, | +B $ B ! C 31 , bD7E0 E | 1 4 $ < $+ 3 9 |
| !"#$#%*)%((!"#$#%*)%() !"#$#%*)%(> | +B  $ B ! C 31, b | +B $ B ! C 31 , bD7E0 E | 1 4 $ < $+ 3 9 |
| !"#$#%*)%(@!"#$#%*)%(% |  | 3 .3/ 7.7/ | < |
| !"#$#%*)%('!"#$#%*)%(& !"#$#%*)%(*!"#$#%*)%(A |  | 3 .3/ | < |
| !"#$#%*)%))!"#$#%*)%)> |  | 3 .3/ 7.7/ | < |

# !"#$#%*)%)@!"#$#%*)%)%!"#$#%*)%)'!"#$#%*)%)&!"#$#%*)%)*!"#$#%*)%)A!"#$#%*)%>?!"#$#%*)%>(!"#$#%*)%>)!"#$#%*)%>>!"#$#%*)%>@ B ; E4 -E B ; E4 -E B 9< 4; D9 D 1 1 <-< "9 B D 7 !"#$#%*)'?*!"#$#%*)'?A!"#$#%*)'(?!"#$#%*)'((!"#$#%*)'()!"#$#%*)'(>!"#$#%*)'(@!"#$#%*)'(%!"#$#%*)'('!"#$#%*)'(&!"#$#%*)'(*!"#$#%*)'(A!"#$#%*)')?!"#$#%*)')(!"#$#%*)'))!"#$#%*)')>!"#$#%*)')@!"#$#%*)')%!"#$#%*)')'!"#$#%*)')&!"#$#%*)')*!"#$#%*)')A!"#$#%*)'>?!"#$#%*)'>( !"#$#%*)'>)!"#$#%*)'>>!"#$#%*)'>@!"#$#%*)'>%!"#$#%*)'>'!"#$#%*)'>&!"#$#%*)'>*!"#$#%*)'>A!"#$#%*)'@?!"#$#%*)'@(!"#$#%*)'@)!"#$#%*)'@>!"#$#%*)'@@!"#$#%*)'@%!"#$#%*)'@'!"#$#%*)'@&!"#$#%*)'@*!"#$#%*)'@A!"#$#%*)'%?!"#$#%*)'%(!"#$#%*)'%)!"#$#%*)'%>!"#$#%*)'%@!"#$#%*)'%% !"#$#%*)'%'!"#$#%*)'%&!"#$#%*)'%*!"#$#%*)'%A!"#$#%*)''?!"#$#%*)''(!"#$#%*)'')!"#$#%*)''>!"#$#%*)''@!"#$#%*)''%!"#$#%*)'''!"#$#%*)''&!"#$#%*)''*!"#$#%*)''A!"#$#%*)'&?!"#$#%*)'&(!"#$#%*)'&)!"#$#%*)'&>!"#$#%*)'&@!"#$#%*)'&%!"#$#%*)'&'!"#$#%*)'&&!"#$#%*)'&*!"#$#%*)'&A !"#$#%*)'*?!"#$#%*)'*(!"#$#%*)'*)!"#$#%*)'*>!"#$#%*)'*@!"#$#%*)'*%!"#$#%*)'*'!"#$#%*)'*&!"#$#%*)'**!"#$#%*)'*A!"#$#%*)'A?!"#$#%*)'A( 4 1 7 1 I 4 1 7 -0- 2 ;:2 !"#$#%*)'A)!"#$#%*)'A> $++ F 9 0+ $" B 0" 7 +;;B 0;" 17 1 !"#$#%*)'A@ X!;7B5(A F 1;; " !"#$#%*)'A%!"#$#%*)'A'!"#$#%*)&??!"#$#%*)&)@!"#$#%*)&)&!"#$#%*)&)*!"#$#%*)&>'!"#$#%*)&@)!"#$#%*)&%(!"#$#%*)&%>!"#$#%*)&%@!"#$#%*)&%'!"#$#%*)&'>!"#$#%*)&'%

|  | | 1 L2 ,  7 < | | :7B5"3 I<B.77/ | | 1;F :7B5"3 | |
| --- | --- | --- | --- | --- | --- | --- | --- |
| !"#$#%*)&'&!"#$#%*)&'* !"#$#%*)&'A!"#$#%*)&&? | | $++ X!;7B5(A | | F | | 9 0+ $" B 0" 7 +;;B 0;" 17 1 F 1;; " | |
| !"#$#%*)&&(!"#$#%*)&&)  !"#$#%*)&&>!"#$#%*)&&@ !"#$#%*)&&% | | 4 $ | | 9" 2 | | 51 $1$4 12 +; 4 $-<"!: 1< +49 5 | |
| !"#$#%*)&&'!"#$#%*)&&&  !"#$#%*)&&*!"#$#%*)&&A  !"#$#%*)&*?!"#$#%*)&*(  !"#$#%*)&*)!"#$#%*)&*> !"#$#%*)&*@ | | , | | 9" 2 | | 51 $1$4 12 +; 4 $-<"!: 1< +49 5 | |
| !"#$#%*)&*%!"#$#%*)&*'  !"#$#%*)&*&!"#$#%*)&** !"#$#%*)&*A!"#$#%*)&A(  !"#$#%*)&A)!"#$#%*)&A> | |  | | 9" 2 | | 51 $1$4 12 +; 4 $-<"!: 1< +49 5 | |
| !"#$#%*)&A@!"#$#%*)&A% !"#$#%*)&A' | | , ! | | 9" 2 | | 51 $1$4 12 +; 4 $-<"!: 1< +49 5 | |
| !"#$#%*)&A&!"#$#%*)&A* | | 4 B | | 9" 2 | | 51 $1$4 12 +; 4 $-<"!: 1< +49 5 | |
| !"#$#%*)&AA | | #4,$ | | 9" 2 | | 51 $1$4 12 +; 4 $-<"!: 1< +49 5 | |
| !"#$#%*)*?? | | 4 B | | 9" 2 | | 51 $1$4 12 +; 4 $-<"!: 1< +49 5 | |
| !"#$#%*)*?( | |  | | 9" 2 | | 51 $1$4 12 +; 4 $-<"!: 1< +49 5 | |
| !"#$#%*)*?) | | $++ X!;7B5(A | | F | | 9 0+ $" B 0" 7 +;;B 0;" 17 1 F 1;; " | |
| !"#$#%*)*?>!"#$#%*)*?@ !"#$#%*)*?% | | 2 | | 9" 2 | | 51 $1$4 12 +; 4 $-<"!: 1< +49 5 | |
| !"#$#%*)*?'!"#$#%*)*?& | | 4 < | | 9" 2 | | 51 $1$4 12 +; 4 $-<"!: 1< +49 5 | |
| !"#$#%*)*?* | | 4 1 | | 9" 2 | | 51 $1$4 12 +; 4 $-<"!: 1< +49 5 | |
| !"#$#%*)*?A | |  | | 9" 2 | | 51 $1$4 12 +; 4 $-<"!: 1< +49 5 | |
| !"#$#%*)*(?!"#$#%*)*(( | | $++ X!;7B5(A | | F | | 9 0+ $" B 0" 7 +;;B 0;" 17 1 F 1;; " | |

# !"#$#%*)*()!"#$#%*)*(>!"#$#%*)*(@!"#$#%*)*(%!"#$#%*)*(&!"#$#%*)*(*!"#$#%*)*)?!"#$#%*)*)(!"#$#%*)*))!"#$#%*)*)>!"#$#%*)*)@!"#$#%*)*)%!"#$#%*)*)'!"#$#%*)*)&!"#$#%*)*)*!"#$#%*)*)A!"#$#%*)*>?!"#$#%*)*>(!"#$#%*)*>)

|  | 2 ,1_O | :7B5"3 I<B.77/ | LLB$ "W;5$12;D D P;" 7 P"1 _;:7B5"3 |
| --- | --- | --- | --- |
| !"#$#%*)*>> | $++ X!;7B5(A | F | 9 0+ $" B 0" 7 +;;B 0;" 17 1 F 1;; " |
| !"#$#%*)*>@ |  | 9" 2 | 51 $1$4 12 +; 4 $-<"!: 1< +49 5 |
| !"#$#%*)*>%!"#$#%*)*>' !"#$#%*)*>&!"#$#%*)*>* | 4 B | 9" 2 | 51 $1$4 12 +; 4 $-<"!: 1< +49 5 |
| !"#$#%*)*>A | #4,$ | 9" 2 | 51 $1$4 12 +; 4 $-<"!: 1< +49 5 |
| !"#$#%*)*@? | 3 0- B7 | 9" 2 | 51 $1$4 12 +; 4 $-<"!: 1< +49 5 |
| !"#$#%*)*@( |  | 9" 2 | 51 $1$4 12 +; 4 $-<"!: 1< +49 5 |
| !"#$#%*)*@) | $++ X!;7B5(A | F | 9 0+ $" B 0" 7 +;;B 0;" 17 1 F 1;; " |
| !"#$#%*)*@>!"#$#%*)*@@ | 2 ,1_O | :7B5"3 I<B.77/ | LLB$ "W;5$12;D D P;" 7 P"1 _;:7B5"3 |
| !"#$#%*)*@%!"#$#%*)*@'  !"#$#%*)*@&!"#$#%*)*@* !"#$#%*)*@A!"#$#%*)*%? | $++ X!;7B5(A | F | 9 0+ $" B 0" 7 +;;B 0;" 17 1 F 1;; " |

# !"#$#%*)*%(!"#$#%*)*%)!"#$#%*)*%>!"#$#%*)*%@!"#$#%*)*%%!"#$#%*)*%'!"#$#%*)*%&!"#$#%*)*%*!"#$#%*)*%A!"#$#%*)*'?!"#$#%*)*'(!"#$#%*)*')!"#$#%*)*'>!"#$#%*)*'@!"#$#%*)*'%!"#$#%*)*''!"#$#%*)*'&!"#$#%*)*'*!"#$#%*)*'A!"#$#%*)*&?!"#$#%*)*&(!"#$#%*)*&)!"#$#%*)*&>!"#$#%*)*&@ !"#$#%*)*&%!"#$#%*)*&'!"#$#%*)*&&!"#$#%*)*&*!"#$#%*)*&A!"#$#%*)**?!"#$#%*)**(!"#$#%*)**)!"#$#%*)**>!"#$#%*)**@!"#$#%*)**%!"#$#%*)**'!"#$#%*)**&!"#$#%*)***!"#$#%*)**A!"#$#%*)*A?!"#$#%*)*A(!"#$#%*)*A)!"#$#%*)*A>!"#$#%*)*A@!"#$#%*)*A%!"#$#%*)*A'!"#$#%*)*A&!"#$#%*)*A* !"#$#%*)*AA!"#$#%*)A??!"#$#%*)A?(!"#$#%*)A?)!"#$#%*)A?>!"#$#%*)A?@!"#$#%*)A?%!"#$#%*)A?'!"#$#%*)A?&!"#$#%*)A?*!"#$#%*)A?A!"#$#%*)A(?!"#$#%*)A((!"#$#%*)A()!"#$#%*)A(>!"#$#%*)A(@!"#$#%*)A(%!"#$#%*)A('!"#$#%*)A(&!"#$#%*)A(*!"#$#%*)A(A!"#$#%*)A)?!"#$#%*)A)(!"#$#%*)A)) !"#$#%*)A)>!"#$#%*)A)@!"#$#%*)A)%!"#$#%*)A)'!"#$#%*)A)&!"#$#%*)A)*!"#$#%*)A)A!"#$#%*)A>?!"#$#%*)A>(!"#$#%*)A>)!"#$#%*)A>>!"#$#%*)A>@!"#$#%*)A>%!"#$#%*)A>'!"#$#%*)A>&!"#$#%*)A>*!"#$#%*)A>A!"#$#%*)A@?!"#$#%*)A@(!"#$#%*)A@)!"#$#%*)A@>!"#$#%*)A@@!"#$#%*)A@%!"#$#%*)A@' !"#$#%*)A@&!"#$#%*)A@*!"#$#%*)A@A!"#$#%*)A%?!"#$#%*)A%(!"#$#%*)A%)!"#$#%*)A%>!"#$#%*)A%@!"#$#%*)A%%!"#$#%*)A%'!"#$#%*)A%&!"#$#%*)A%*

| 7 7 " 2 75F <  !"#$#%*)A%A!"#$#%*)A'?!"#$#%*)A'(!"#$#%*)A')!"#$#%*)A'>!"#$#%*)A'@!"#$#%*)A'%!"#$#%*)A''!"#$#%*)A'&!"#$#%*)A'*!"#$#%*)A'A!"#$#%*)A&?!"#$#%*)A&(!"#$#%*)A&)!"#$#%*)A&>!"#$#%*)A&@ | 7F<7 7 |
| --- | --- |
| $ " 2 75F < | 7F<7 7 |

!"#$#%*)A&%!"#$#%*)A&'!"#$#%*)A&&!"#$#%*)A&*!"#$#%*)A&A!"#$#%*)A*?!"#$#%*)A*(!"#$#%*)A*)!"#$#%*)A*>!"#$#%*)A*@!"#$#%*)A*%!"#$#%*)A*'!"#$#%*)A*&!"#$#%*)A**!"#$#%*)A*A!"#$#%*)AA?!"#$#%*)AA(!"#$#%*)AA)!"#$#%*)AA>!"#$#%*)AA@!"#$#%*)AA%!"#$#%*)AA'!"#$#%*)AA&!"#$#%*)AA* !"#$#%*)AAA!"#$#%*>???!"#$#%*>??(!"#$#%*>??)!"#$#%*>??>!"#$#%*>??@!"#$#%*>??%!"#$#%*>??'!"#$#%*>??&!"#$#%*>??*!"#$#%*>??A!"#$#%*>?(?!"#$#%*>?((!"#$#%*>?()!"#$#%*>?(>!"#$#%*>?(@!"#$#%*>?(%!"#$#%*>?('!"#$#%*>?(&!"#$#%*>?(*!"#$#%*>?(A!"#$#%*>?)?!"#$#%*>?)(!"#$#%*>?)) !"#$#%*>?)>!"#$#%*>?)@!"#$#%*>?)%!"#$#%*>?)'!"#$#%*>?)&!"#$#%*>?)*!"#$#%*>?)A!"#$#%*>?>?!"#$#%*>?>(!"#$#%*>?>)!"#$#%*>?>>!"#$#%*>?>@!"#$#%*>?>%!"#$#%*>?>'!"#$#%*>?>&!"#$#%*>?>*!"#$#%*>?>A!"#$#%*>?@?!"#$#%*>?@(!"#$#%*>?@)!"#$#%*>?@>!"#$#%*>?@@!"#$#%*>?@%

# 7 " 2 $ 75F < 7F<7 7 !"#$#%*>?@'!"#$#%*>?@&!"#$#%*>?@*!"#$#%*>?@A!"#$#%*>?%?!"#$#%*>?%(!"#$#%*>?%)!"#$#%*>?%>!"#$#%*>?%@!"#$#%*>?%%!"#$#%*>?%'!"#$#%*>?%&!"#$#%*>?%*!"#$#%*>?%A!"#$#%*>?'?!"#$#%*>?'(!"#$#%*>?')!"#$#%*>?'>!"#$#%*>?'@!"#$#%*>?'%!"#$#%*>?''!"#$#%*>?'&!"#$#%*>?'*!"#$#%*>?'A !"#$#%*>?&?!"#$#%*>?&(!"#$#%*>?&)!"#$#%*>?&>!"#$#%*>?&@!"#$#%*>?&%!"#$#%*>?&'!"#$#%*>?&&!"#$#%*>?&*!"#$#%*>?&A!"#$#%*>?*?!"#$#%*>?*(!"#$#%*>?*)!"#$#%*>?*>!"#$#%*>?*@!"#$#%*>?*%!"#$#%*>?*'!"#$#%*>?*&!"#$#%*>?**!"#$#%*>?*A!"#$#%*>?A?!"#$#%*>?A(!"#$#%*>?A)!"#$#%*>?A> !"#$#%*>?A@!"#$#%*>?A%!"#$#%*>?A'!"#$#%*>?A&!"#$#%*>?A*!"#$#%*>?AA!"#$#%*>(??!"#$#%*>(?(!"#$#%*>(?)!"#$#%*>(?>!"#$#%*>(?%!"#$#%*>(?'!"#$#%*>(?&!"#$#%*>(?*!"#$#%*>(?A!"#$#%*>((?!"#$#%*>(((!"#$#%*>(()!"#$#%*>((>!"#$#%*>((@!"#$#%*>((%!"#$#%*>(('!"#$#%*>((&!"#$#%*>((* !"#$#%*>((A!"#$#%*>()?!"#$#%*>()(!"#$#%*>())!"#$#%*>()>!"#$#%*>()@!"#$#%*>()%!"#$#%*>()'!"#$#%*>()&!"#$#%*>()*!"#$#%*>()A!"#$#%*>(>?!"#$#%*>(>(!"#$#%*>(>)!"#$#%*>(>>!"#$#%*>(>@!"#$#%*>(>%!"#$#%*>(>'!"#$#%*>(>&!"#$#%*>(>*!"#$#%*>(>A!"#$#%*>(@?!"#$#%*>(@(!"#$#%*>(@)

!"#$#%*>(@>!"#$#%*>(@@!"#$#%*>(@%!"#$#%*>(@'!"#$#%*>(@&!"#$#%*>(@*!"#$#%*>(@A!"#$#%*>(%?!"#$#%*>(%(!"#$#%*>(%)!"#$#%*>(%>!"#$#%*>(%@!"#$#%*>(%%!"#$#%*>(%'!"#$#%*>(%&!"#$#%*>(%*!"#$#%*>(%A!"#$#%*>('?!"#$#%*>('(!"#$#%*>(')!"#$#%*>('>!"#$#%*>('@!"#$#%*>('%!"#$#%*>('' !"#$#%*>('&!"#$#%*>('*!"#$#%*>('A!"#$#%*>(&?

| 2 7 " 2 $ 75F < | 7F<7 7 |
| --- | --- |
| !"#$#%*>(&(!"#$#%*>(&) 9 7 " 2 $ 75F <  !"#$#%*>(&>!"#$#%*>(&@!"#$#%*>(&%!"#$#%*>(&'!"#$#%*>(&&!"#$#%*>(&*!"#$#%*>(&A!"#$#%*>(*?!"#$#%*>(*(!"#$#%*>(*)!"#$#%*>(*>!"#$#%*>(*@!"#$#%*>(*%!"#$#%*>(*'!"#$#%*>(*&!"#$#%*>(**!"#$#%*>(*A | 7F<7 7 |
| <7 " 2 $ 75F < | 7F<7 7 |
| !"#$#%*>(A?!"#$#%*>(A( 0" 2 $ 75F <  !"#$#%*>(A)!"#$#%*>(A>  !"#$#%*>(A@!"#$#%*>(A%  !"#$#%*>(A'!"#$#%*>(A& | 7F<7 7 |
| !"#$#%*>(A* 17 " 2 75F < | 7F<7 7 |

# !"#$#%*>(AA!"#$#%*>)??!"#$#%*>)?(!"#$#%*>)?)!"#$#%*>)?>!"#$#%*>)?@!"#$#%*>)?%!"#$#%*>)?'!"#$#%*>)?&!"#$#%*>)?*!"#$#%*>)?A!"#$#%*>)(?!"#$#%*>)((!"#$#%*>)()!"#$#%*>)(>!"#$#%*>)(@!"#$#%*>)(%!"#$#%*>)('!"#$#%*>)(&!"#$#%*>)(*!"#$#%*>)(A!"#$#%*>))?!"#$#%*>))(!"#$#%*>)))

| !"#$#%*>))>!"#$#%*>))@!"#$#%*>))%!"#$#%*>))'!"#$#%*>))&!"#$#%*>))*!"#$#%*>))A!"#$#%*>)>?!"#$#%*>)>( |  |  |
| --- | --- | --- |
| ,707 1 $ | 75F < | 7F<7 7 |
| !"#$#%*>)@'!"#$#%*>)@& " 2 $  !"#$#%*>)@* | " 2 $ | 2 |
[truncated: 2,548,586 more chars]
