# Supplementary material for: Loss of orf3b in the circulating SARS-CoV-2 strains
Source: Emerg Microbes Infect. 2020 Dec 24;9(1):2685–96. doi: 10.1080/22221751.2020.1852892 (PMC7782295; doi:10.1080/22221751.2020.1852892)
Supplement: Figure_S2_updated.docx [file TEMI_A_1852892_SM1185.docx]

**Figure S2**

**(For the point-to-point address only; Response to reviewer 1 comment 1)**


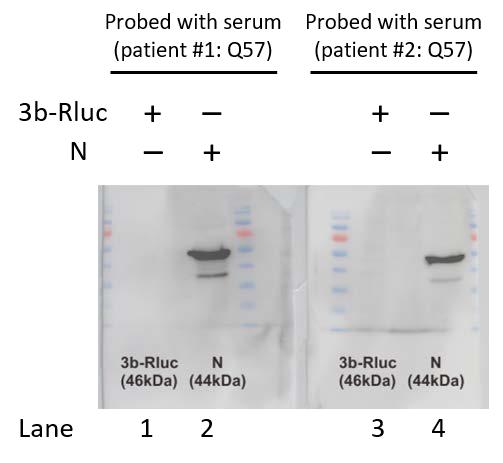


Fig. S2. Examination of the antibodies presented in the serum samples of COVID-19 patients using Western blotting method.

Cell lysates containing the orf3b protein (fused with Renilla luciferase) or nucleoprotein were loaded into the SDS -PAGE. Proteins were detected by the two serum samples obtained from patient 1 and 2. The bands (lane 2 and 4) represented the presence of anti-N antibody in the patients’ sera. However, no band was observed in lane 1 and 3, indicating that anti-orf3b antibody may not able to recognize the denatured orf3b protein on the membrane.

It is noted that anti-orf3b antibody can be detected in the serum samples of COVID-19 patients using LIPS assay (Nat Immunol, 2020. 21:1293-1301). We proposed that the denatured orf3b protein in the denaturing PAGE will loss the structure of immune epitope for the recognition by anti-orf3b antibody. It may happen when the protein is small. Therefore, we have repeated the detection of anti-orf3b antibody using the LIPS method described in the above-mentioned Nature Immunology paper. Data were shown in Response 2 and Fig. S1 in the revised manuscript.

**Reference:**

Hachim et al. ORF8 and ORF3b antibodies are accurate serological markers of early and late SARS-CoV-2 infection. Nat Immunol. 2020 21:1293-1301.
