# Supplementary material for: Loss of orf3b in the circulating SARS-CoV-2 strains
Source: Emerg Microbes Infect. 2020 Dec 24;9(1):2685–96. doi: 10.1080/22221751.2020.1852892 (PMC7782295; doi:10.1080/22221751.2020.1852892)
Supplement: Figure_S1_updated.docx [file TEMI_A_1852892_SM1184.docx]

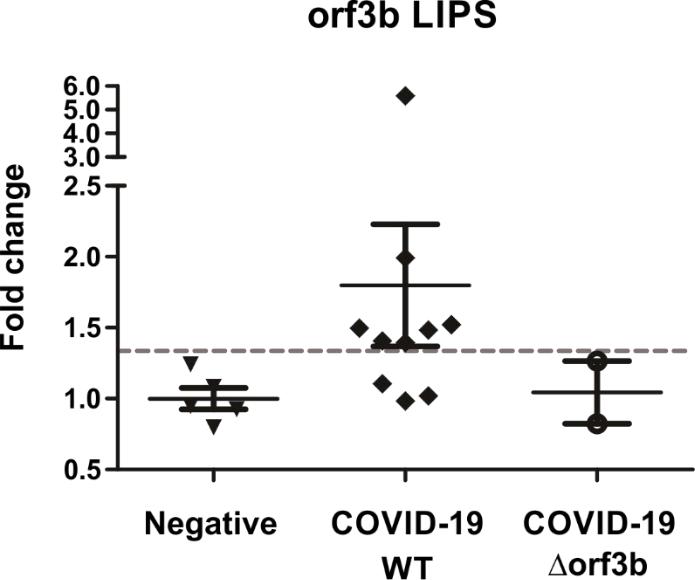


**Figure S1. LIPS assay for the determination of anti-orf3b antibody in Q57 and H57 patient serum samples.**

Antibodies against orf3b in serum samples of patients infected by orf3b intact SARS-CoV-2 or loss-of-orf3b SARS-CoV-2 were detected using orf3b Luciferase Immunoprecipitation System (LIPS) assay. Negative control group (n=5) were anonymous pre-pandemic serum samples. COVID-19 WT group (n=10) were sera from patients with intact orf3b reading frame (orf3a Q57 genotype). COVID-19 ∆orf3b (n=2) were the only two sera available in Hong Kong that have truncated orf3b open-reading frame (orf3a H57 genotype). The luciferase signal was normalized to the negative control group and represented as fold change. Cut-off value (grey dotted line) was set as determined by the mean plus two s.d. of the negative control group. Since two serum samples of H57 genotype were available in Hong Kong, no statistical analysis was performed.
